# Comparative Genomic Analysis of Food-Originated Coagulase- Negative *Staphylococcus*: Analysis of Conserved Core Genes and Diversity of the Pan-Genome

**DOI:** 10.4014/jmb.1910.10049

**Published:** 2019-12-09

**Authors:** Sojeong Heo, Jung-Sug Lee, Jong-Hoon Lee, Do-Won Jeong

**Affiliations:** 1 Department of Food and Nutrition, Dongduk Women’s University, Seoul 02748, Republic of Korea; 2 Department of Food and Nutrition, Kookmin University, Seoul 0707, Republic of Korea; 3 Department of Food Science and Biotechnology, Kyonggi University, Suwon 16227, Republic of Korea

**Keywords:** Coagulase-negative *Staphylococcus*, pan-genome, prophage, transposase

## Abstract

To shed light on the genetic differences among food-originated coagulase-negative *Staphylococcus* (CNS), we performed pan-genome analysis of five species: *Staphylococcus carnosus* (two strains), *Staphylococcus equorum* (two strains), *Staphylococcus succinus* (three strains), *Staphylococcus xylosus* (two strains), and *Staphylococcus saprophyticus* (one strain). The pan-genome size increases with each new strain and currently holds about 4,500 genes from 10 genomes. Specific genes were shown to be strain dependent but not species dependent. Most specific genes were of unknown function or encoded restriction-modification enzymes, transposases, or prophages. Our results indicate that unique genes have been acquired or lost by convergent evolution within individual strains.

## Introduction

Coagulase-negative staphylococci (CNS) have been detected from several niches including the skin and mucous membranes of mammals, the environment, and a variety of food stuffs such as meat, cheese, and raw milk [1-3]. Although some CNS, such as *Staphylococcus epidermidis*, *S. haemolyticus*, and *S. saprophyticus*, have been shown to cause occasional opportunistic infections [4-6], CNS species are frequently isolated from fermented foods and known as benign bacteria. Genome analyses revealed that food-originated CNS did not encode any of the virulence factors found in *S. aureus* [7-12].

Food-originated CNS have been reported to play a major role in the development of sensory properties in fermented foods by the reduction of nitrates to nitrite and then to nitrous oxide, as well as through proteolysis and lipolysis [13, 14]. In particular, *S. carnosus*, *S. equorum*, *S. succinus*, and *S. xylosus*, produce low-molecular-weight compounds, including esters, amino acids, aldehydes, amines, and free fatty acids, which have an impact on flavour [15-17]. These species have been detected as dominant bacteria in naturally-fermented meat products, and have been used as starter cultures in meat fermentation processes [18, 19]. Several food-originated CNS strains have been selected as starter candidates through safety assessments to check their benign properties [2, 20-24].

CNS have been recently identified as a dominant group of bacteria in jeotgal, a high-salt-fermented seafood, and doenjang, a high-salt-fermented soybean food, of Korea [25-29]. *S. equorum* KS1039 and *S. succinus* 14BME1 were selected as starter candidates for jeotgal and doenjang through safety assessments [23] and the lack of the virulence factors found in *S. aureus* was confirmed through complete genome sequencing [8, 12]. In addition, *S. succinus* as a starter produces distinguished volatile compound patterns compared with *S. saprophyticus* in soybean fermentation under laboratory conditions [30, 31]. Although the contribution of food-originated CNS in food fermentation has been shown, the specific effects of different species remained unclear. In the current study, we performed comparative genomic analysis of five CNS species that dominantly detected in fermented food, using 10 strains in total, to define the scale and scope of the pan-genome and the core genes, and to clarify the genetic background of species from different niches. This study introduced interspecific comprehensive comparative genome analysis to shed light on the genetic background of CNS species.

## Materials and Methods

### Habitat, Bacterial Strains, and Culture Conditions

Ten CNS comprising *S. carnosus* (two strains), *S. equorum* (two strains), *S. succinus* (three strains), *S. xylosus* (two strains), and *S. saprophyticus* (one strain) were subjected to genomic analysis (Table 1). Strains JCM 6069, TM300, KS1039, Mu2, and 14BME20 originated from fermented foods from various countries [8, 9, 11, 12, 32]. Strains CSM-77 and DSM 14617 originated from the environment [33, 34]. Strains C2a and ATCC 15305 originated from humans [35, 36]. Strain HKUOPL8 originated from animal faecal material [37]. CNS strains were cultured in tryptic soy broth (TSB; Difco, USA) at 30°C for 24 h to maintain their traits [38].

### Comparative Genomics

For comparative genomic analysis of the 10 CNS, genome sequence data from strains JCM 6069 (GenBank accession: NZ_CP016760), TM300 (NC_012121), KS1039 (NZ_CP01311), Mu2 (NZ_CAJL00000000), 14BME20 (NZ_CP018199), CSM-77 (NZ_LUJH01000000), DSM 14617 (NZ_LCSH00000000), C2a (NZ_LN554884), HKUOPL8 (NZ_CP007208), and ATCC 15305 (NC_007350) were obtained from the NCBI database (http://ncbi.nlm.nih.gov/genomes). The average nucleotide identity (ANI), which provides a robust measure of genetic distance among bacterial genomes, among the conserved genes of the genomes was used for comparative analysis [39]. The Efficient Database framework for comparative Genome Analyses using BLAST score Ratios (EDGAR) was used for core genome, pan-genome, and singleton analyses [40]; the genome of strain 14BME20 was used as a reference genome for Venn diagram construction for five genome analysis. Comparative analyses at the protein level were performed by an all-against-all comparison of the annotated genomes. The algorithm used was BLASTP and data were normalized according to the best score [41]. The score ratio value, which shows the quality of the hit, was calculated by dividing the scores of further hits by the best hit [42]. Two genes were considered orthologous when a bidirectional best BLAST hit with a single score ratio value threshold of at least 32% was obtained for orthology estimation.

Genome level differences between CNS genomes with other groups were analysed using BRIG-0.95 [43]. Circular genome maps were generated using reference and query genome sequences in a set of concentric rings coloured according to BLAST identity. BRIG (BLAST Ring Imager Generator)-generated regions of interest were re-annotated using the RAST pipeline and re-inspected for homology by BLASTP and assessment of function. Putative prophage DNA sequences were analysed using the PHASTER (Phage Search Tool Enhanced Release) method [44] and compared by the Easyfig program [45].

MLST of 10 CNS strains was performed according to a previously published *S. equorum* MLST protocol [46] using seven housekeeping genes: *aroE* (encoding shikimate 5-dehydrogenase), *dnaJ* (chaperone protein), *glpF* (glycerol 3-phosphate dehydrogenase), *gmk* (guanylate kinase), *hsp60* (heat shock protein 60), *mutS* (DNA mismatch repair protein), and *pta* (phosphotransacetylase). The phylogenetic tree was constructed using the maximum likelihood method.

### Bacteriophage Isolation and Plaque Formation

Strain 14BME20 cultured in TSB was normalized to 0.3 turbidity at OD_600_ and incubated for 2 h at 37oC after addition of mitomycin C (1 µg/ml). Culture was filtered using 0.45 µm filter (Millex syringe-driven filter, Milipore, USA) to remove bacteria and stored at 4°C until plaque assays. Phage sample (100 µl) were added to *S. aureus* RN4220 (100 µl) and incubated overnight at 37°C before adding to TSB top agar for plaque production. 100 µl of overnight cultures were prepared in 5 ml of TSB containing 0.7% agarose and overlaid onto tryptic soy agar (Difco). After incubation for 24 h at 37oC, plaques were checked to ensure phage lysis. All spot tests were repeated in triplicate to confirm results.

## Results and Discussion

### Genome Summary and General Features

The general features of the 10 CNS genomes are summarized in Table 1. The average genome sequence length of the 10 strains was 2,762,844 bp. *S. carnosus* TM300 exhibited the smallest genome (2,566,424 bp), while strain Mu2 possessed the largest (2,927,171 bp). Both of the *S. equorum* strains possessed genomes that were larger in size than the average of the 10 strains. The CNS strains displayed an average G+C content of 33.29% and *S. carnosus* showed the highest G+C content.

To facilitate a coherent comparative analysis, we performed consistent ORF prediction for 10 CNS (7 complete and 3 incomplete) genome sequences. In this way, comparable numbers of genes were obtained for each genome, with an average of 2,649 ORFs per genome (Table 1). Notably,(BLAST-based) functional *in silico* prediction could be performed for 91.9% of the identified ORFs, while the remaining 8.1% that were not assigned the Clusters of Orthologous Groups (COG) functional classification were predicted to encode hypothetical proteins.

**Fig. 1:**
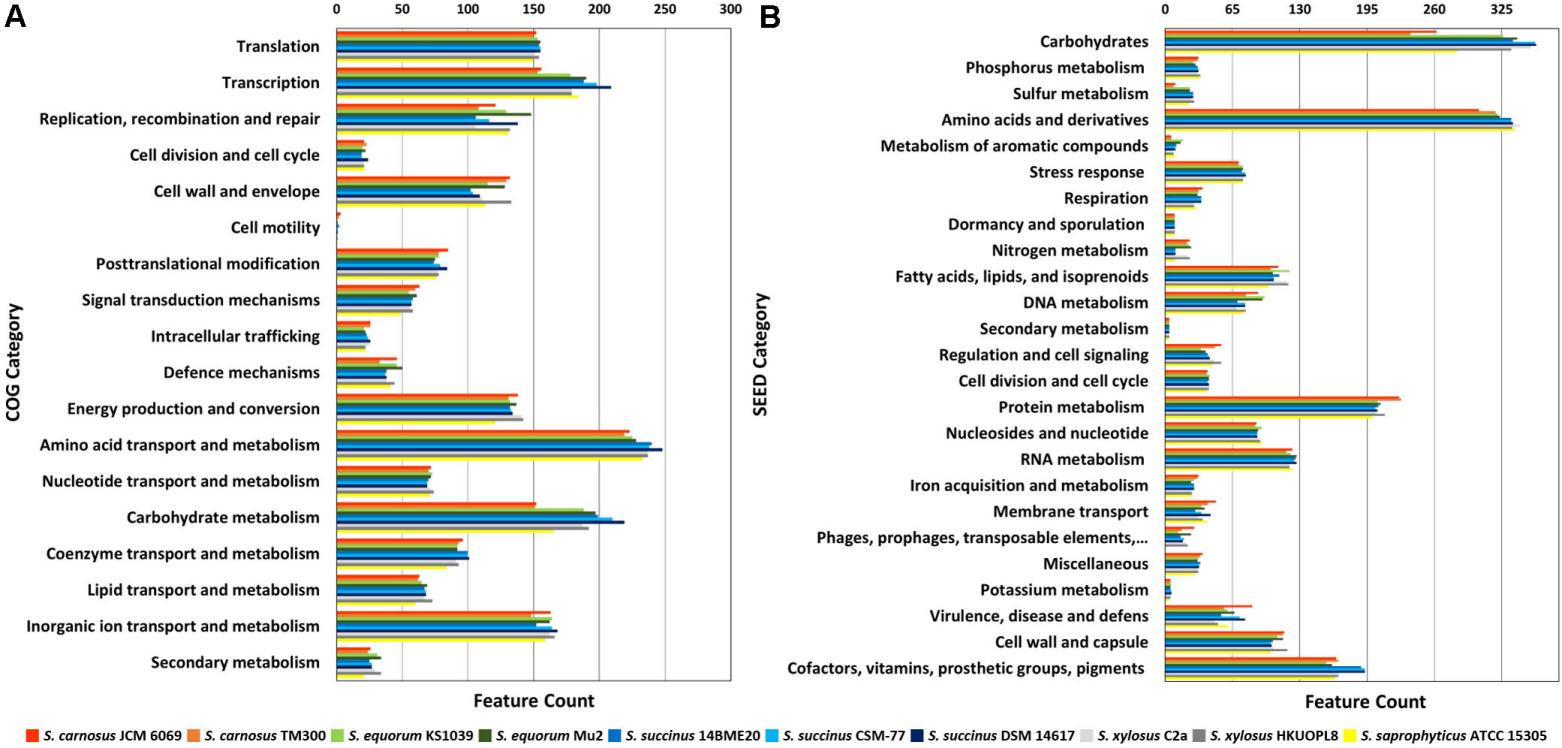
Comparison of functional categories in the 10 coagulase-negative *Staphylococcus* strain genomes based on COG (**A**) and SEED (**B**). Genome sequences of 10 strains JCM 6069, TM300, KS1039, Mu2, 14BME20, CSM-77, DSM 14617, C2a, HKUOPL8, and ATCC 15305 were uploaded to the COG and SEED viewer servers independently. Functional roles of the annotated genes were assigned and grouped in subsystem feature categories. Coloured bars indicate the number of genes assigned to each category.

Analysis using COG functional categorization and SEED subsystem categorization predicted the existence of an average of 2,434 and 2,012 coding sequences (CDSs) per genome, respectively (Table 1). Based on COG functional categorization of genes, amino acid transport and metabolism (8.9%–10.1%) was the most abundant category, followed by carbohydrate transport and metabolism (6.3%–8.6%). Based on the SEED subsystem, over 329 CDSs accounting for 15.1%–17.6% of the CNS genomes were allocated to genes for amino acid biosynthesis and utilization (Fig. 1). The next most abundant subsystem category was related to carbohydrate utilization (12.5%–17.5%), followed by protein metabolism. At the species level, the genomes of *S. succinus* possessed more of the first and second categories than the other CNS genomes based on the SEED subsystem. These major functional groups were related to protein and carbohydrate metabolism and thus appear to represent a key genetic background of the CNS in protein-rich fermented food.

### Pan-Genome and Core-Genome Analysis

**Fig. 2:**
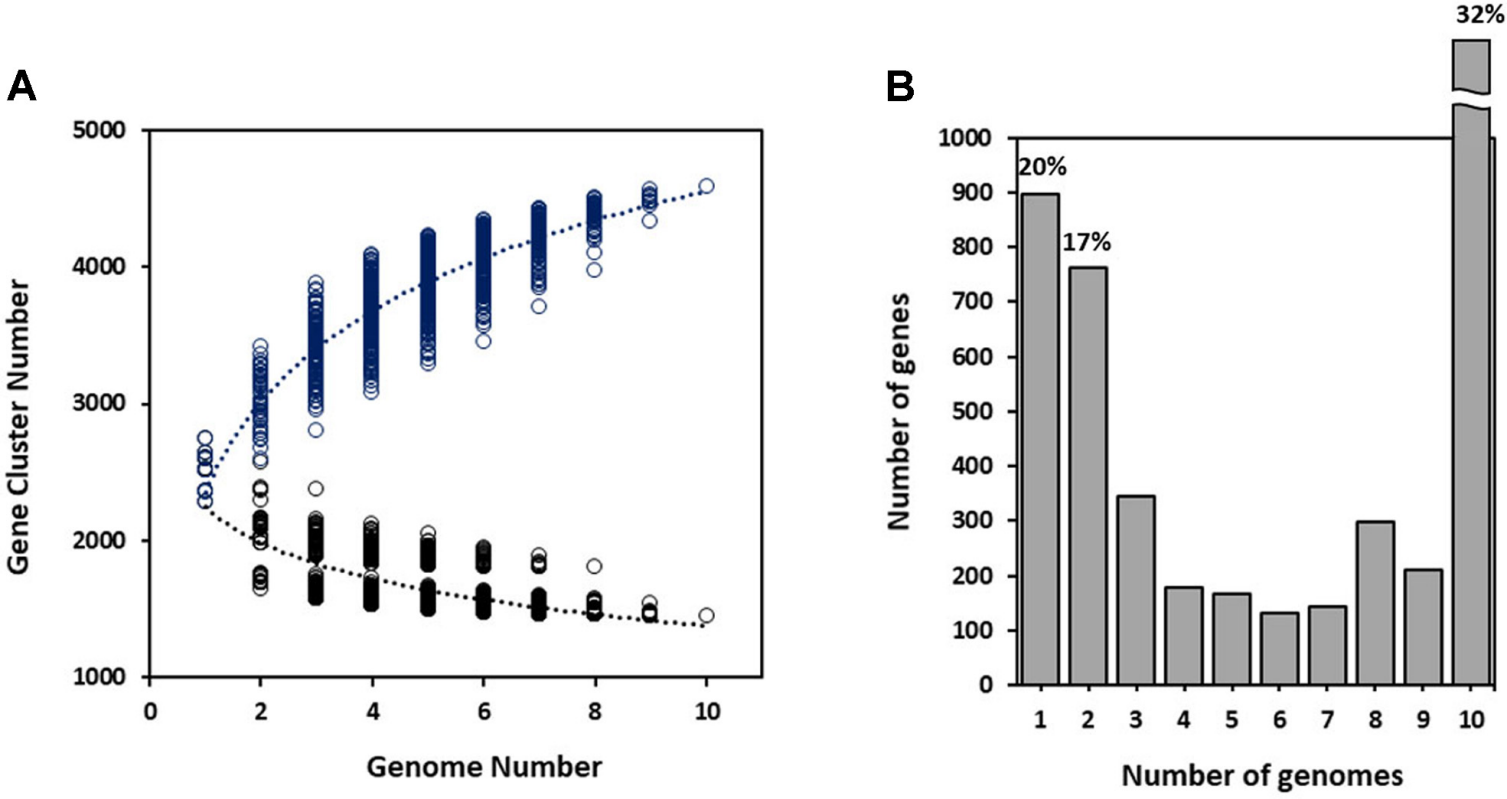
Sizes of the core and pan-genomes (**A**) and the gene frequency (**B**) of the 10 coagulase-negative *Staphylococcus* strain genomes. (**A**) The black curve (core genome) and blue curve (pan-genome) were fitted to the decay function (1096.318exp(-x/2.990)+1422.055) and Heap’s law function (2568.934x^0.254^), respectively. Each dot shows the gene cluster number of individual genome. (**B**) Genes present in a single genome represent lineage-specific genes, while at the opposite end of the scale, genes found in all 10 genomes represent the *Staphylococcus* core genome.

Estimation of the CNS pan-genome indicated that the gene pool should be increased with sequential addition of each new genome (Fig. 2A). In this study, we predicted that the CNS genome could hold at least 4,500 genes. There is a five-fold increase from the first addition (526 genes) to the tenth addition (99 genes), as the growth rate gradually lessens. The above analyses confirmed that five CNS species possessed an open pan-genome that increased in size with the addition of newly sequenced strains. This was consistent with previous studies on the unique genes of CNS [30].

In contrast to the pan-genome, estimation of the CNS core genome indicated that genes shared by all strains decreased with each addition, finally reaching a plateau of around 1,450 genes (Fig. 2A). The decrease dropped from 2,535 genes to 1,992 genes at the first addition, and dropped to 1,453 genes after the tenth addition. As a result, a final constant number of 1,453 shared genes was determined as the core genome size. The size of the CNS core genome decreased as a function of the number of genomes included, while the size of the pan-genome increased. Regression analysis of the shared genes was extrapolated by fitting a decaying function, which was considered to provide the best fit to the dataset. Medini *et al*. [47] reported that the core genes represent the essence of the species, while the unique genes represent the diversity of the species. We therefore conclude that the 1,453 core genes of the 10 CNS are essential components for these microorganisms to survive and thrive in nature.

The average gene content for the CNS genomes was found to be 2,434 ± 92 genes based COG and the 10 CNS share 1,453 genes in their core genomes as determined by EDGAR (Fig. 2B). The genome core genes were the most common genes (32%; Fig. 2B). The core genome accounts for approximately 56.5%–63.5% of the genes in each genome. It was surprising that only 20% of the genes in the pan-genome of CNS (based on these 10 genome sequences) were represented in only one lineage and most strain-specific genes were phage or transposon related, plasmid-encoded, or hypothetical (Table S1). These results suggested variability in gene content between species, as well as between strains of the same species. This again highlighted the genomic plasticity among CNS living in different habits and possessing diverse lifestyles. An open pan-genome is typical of those species that colonize multiple environments and exhibit multiple methods of exchanging genetic material.

### Comparative Analysis of the 10 CNS Genomes

**Fig. 3:**
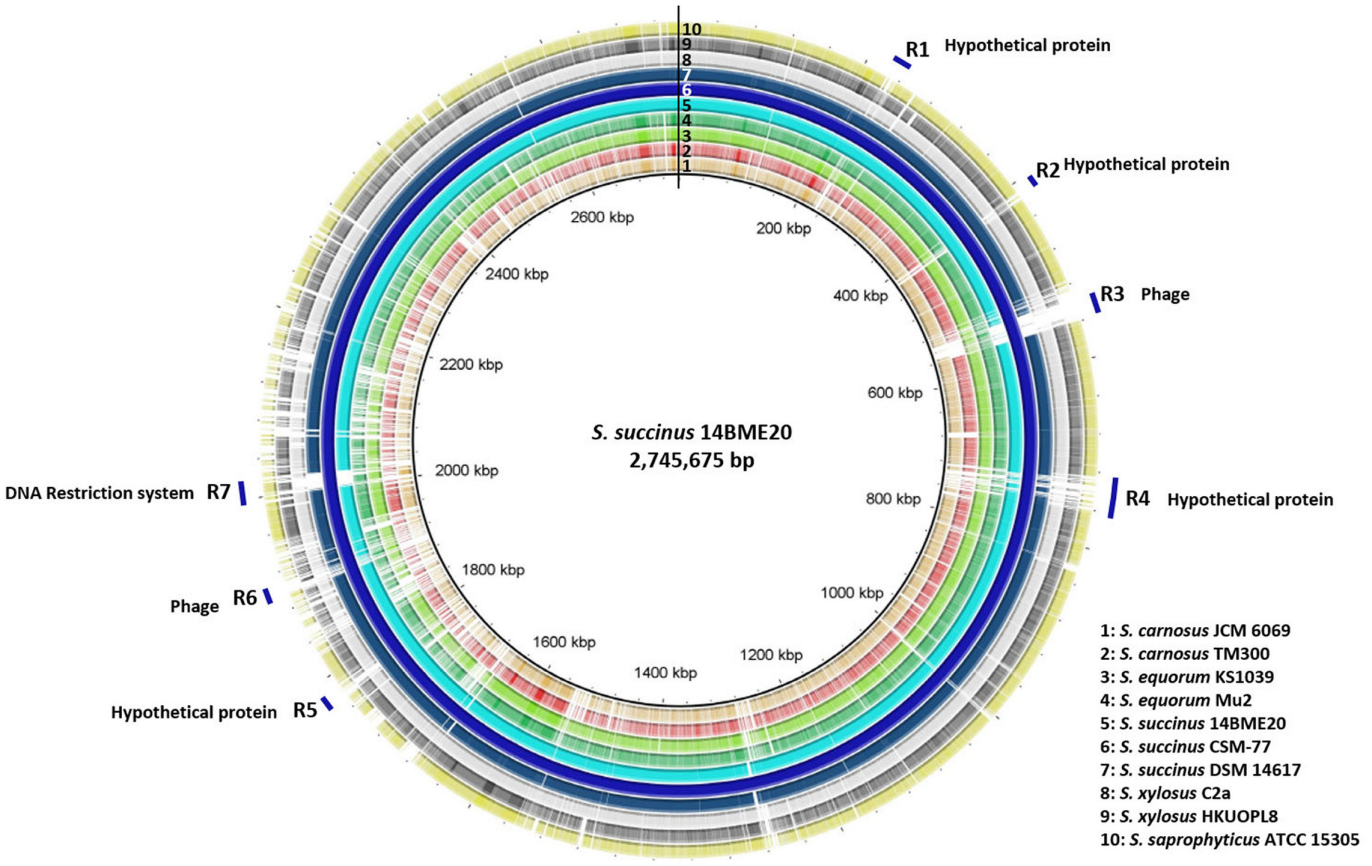
Circular representation of genomes of different lineages/sub-lineages in representative CNS strains, using *Staphylococcus succinus* 14BME20 as a reference. Coloured rings represent different strains. Unique regions of *S. succinus* are marked in blue in the outermost circle.

The genomes of the 10 CNS strains were analysed using BRIG, along with reference strain 14BME20, and seven regions were identified that corresponded to putative phage elements and DNA restriction systems (Fig. 3). Although whole-genome analysis using other reference strains is not shown, the most unique regions corresponded with phage elements and hypothetical proteins. These regions corresponded with the main genetic differences identified between different groups of strains. The unique genes in regions 1, 2, 4, and 5 were identified as hypothetical proteins. Regions 3 and 6 were identified as intact phages and phage elements, respectively, and region 7 comprised genes for a DNA restriction system. These regions correlated with the unique genes identified in strain 14BME20 and corresponded with the pan-genome results (Table S1).

**Fig. 4:**
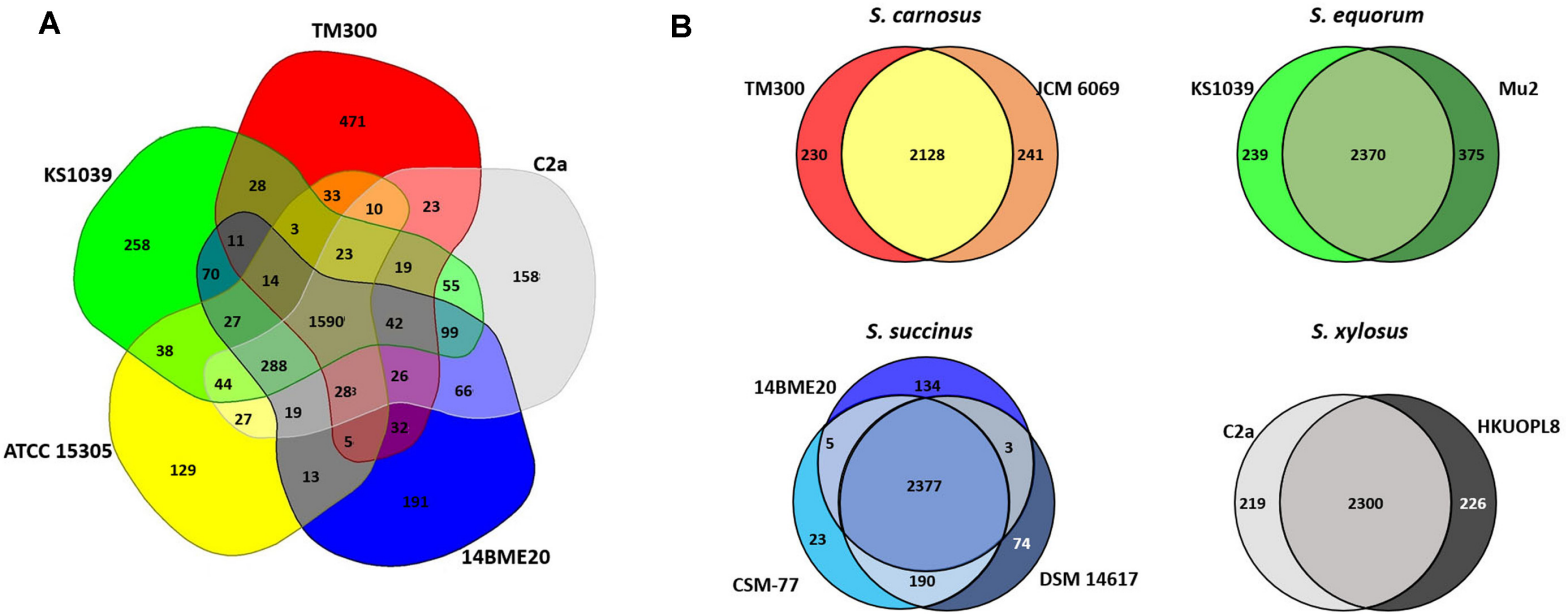
Venn diagram of five different coagulase-negative *Staphylococcus* genomes (**A**) and four sets of genomes from four species (**B**). Venn diagram generated using EDGAR. Overlapping regions represent CDSs shared between the CNS genomes. The numbers outside the overlapping regions indicate the numbers of CDSs in each genome without homologs in the other sequenced CNS genomes.

First, we analysed the genes shared between the genomes of the five different species, TM300, KS1039, ATCC 15305, 14BME20, and C2a, to identify genes that are unique to particular species. The gene pools shared by the genomes of the five different species are depicted in a Venn diagram (Fig. 4A). These five strains shared 1,590 CDSs in their core genome, corresponding to approximately 65.4%–69.4% of their ORFs. Many of the CDSs in the core genome were assigned via COG annotation to functions relating to metabolism and the transport of amino acids and carbohydrates. The genome of strain *S. saprophyticus* ATCC 15305 had the smallest proportion (5.2%) of unique CDSs that were absent from the four other CNS genomes. By contrast, the proportions of unique CDSs in the genomes of strains TM300, KS1039, 14BME20, and C2a were 19.4%, 11.3%, 7.5%, and 6.2%, respectively. The majority of singleton-specific genes were associated with hypothetical proteins (Table S2). Whereas, functional singletons in the genomes of strains TM300, KS1039, ATCC 15305, 14BME20, and C2a were allocated to a CRISPR-associated protein, an MFS transporter, phage-related genes, and transposase genes. Interestingly, most unique genes were not shared only with the same species, they were allocated as singletons or shared with other species (Fig. 4B and Table S1). These results implied that CNS related to fermentation might have originated from the same ancestor and have divided into different species through the loss and gain of genes allowing adaptations to new environments.

**Fig. 5:**
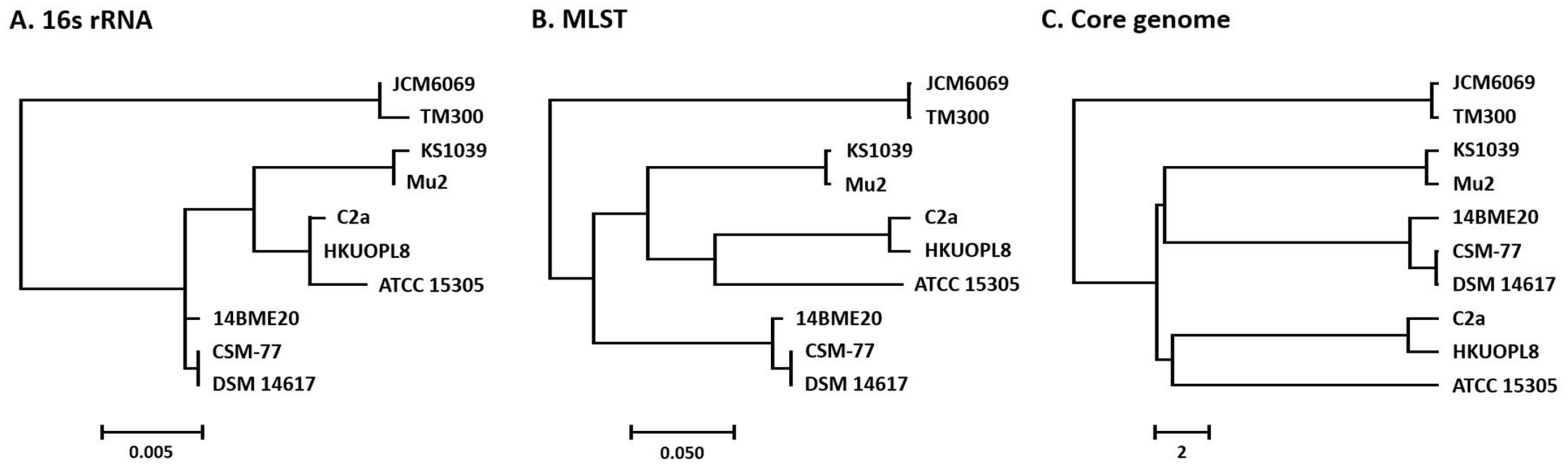
Phylogenetic relationship between the 10 different coagulase-negative *Staphylococcus* genomes as determined by multiple methods. 16S rRNA (**A**) and the multilocus sequence typing (**B**) sequences reconstructed with maximum likelihood algorithms. Consensus tree of all core genomes (**C**) generated using the unweighted pair-group method with the arithmetic mean.

In fact, it was difficult to differentiate biochemically between three species, *S. xylosus*, *S. equorum*, and *S. succinus*, due to them possessing highly similar properties [48]. The 16S rRNA genes showed high genetic identity (>96.8%) among the five species (Fig. 5A): *S. carnosus*, *S. equorum*, *S. succinus*, *S. xylosus*, and *S. saprophyticus*, detected frequently in fermented food. Our analysis of the pan-genome of 10 CNS did not show species-specific patterns and specific genes did not distinguish distinct species but showed strain-specific properties (Fig. 4 and Table S1).

### Mobile Genetic Elements

Mobile elements contribute to horizontal gene transfer including the acquisition and loss of genes. Mobile genes contribute to the unique properties of each isolate whether inherited from a common ancestor or acquired from another strain. Therefore, we analysed the mobile genetic elements among the CNS.

**Fig. 6:**
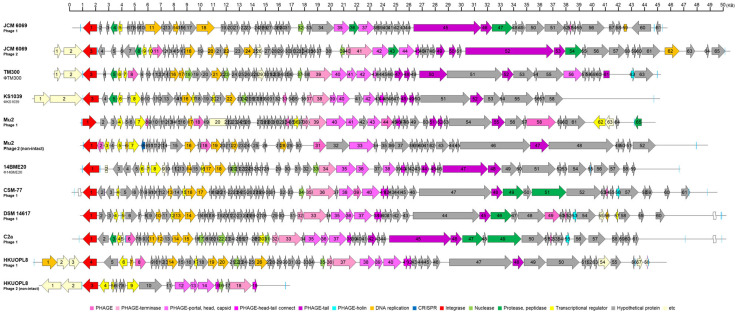
Phage gene clusters in the 10 CNS. Genomes were aligned using the BLASTn algorithm and regions with more than 65% identity are indicated. The positions and orientations of the coding regions are represented by arrows. Genome modules are color-coded according to the legend. Blue lines indicate the attachment site of phage.

*Prophage Φ* **14BME20**. Prophages are one type of mobile element involved in horizontal gene transfer between bacteria that account for a substantial amount of inter-strain genetic variability in several bacterial species. Indeed, prophages contribute a large part of strain-specific DNA. The *S. succinus* genome contains a 40.3 kb intact prophage, termed Φ14BME20 (Fig. 6), which is located at 0.4 Mbp (Fig. 3, R3 region). According to its size, Φ14BME20 belongs to the class II phages [49]. The prophage encodes 59 proteins, of which 38 have no assigned function. The remaining ORFs code for typical phage functions such as integrase, terminase subunits, structural proteins, and regulatory functions. Φ14BME20 does not possess the *holin* gene, which functions in bacteriophage lysis. Φ14BME20 was not formed plaque experimentally, and these result was matched the lack of *holin* gene. The lack of both the *holin* and *lysin* genes likely effects the mobility of Φ14BME20. Either side of BK815_RS02645 and BK815_RS02935, two-pair attachment sites for the bacteriophage were identified (Fig. 6, blue line). Φ14BME20 had no striking overall similarity to other staphylococcal prophages or published prophage sequences. In addition, as shown in Fig. 6, phage structures vary in different strains and these results confer the presence of unique genes in each CNS.

**Genomic islands.** Genomic islands exist in a genome as a result of horizontal gene transfer. Genomic islands can be identified by their GC content profile [50]. The average GC content of strain 14BME20 was 33.08% and five regions with distinct GC contents were identified (Fig. S1, regions 1 to 5). Five regions (1–5) were matched with tRNA operons and these regions were detected in the complete genomes of CNS, with the exception of the draft genomes of CSM-77, DSM 14617, and Mu2. The tRNA operons were highly conserved and possessed high GC contents. In addition, mobile genes such as transposase genes, which were generally detected in genomic islands [51], were not detected flanking the five regions indicating that these regions may not have been acquired. Genomic islands were therefore not detected in the genome of 14BME20 or the six complete CNS genomes.

**Transposase.** Transposases play an important role in horizontal gene transfer between phylogenetically distinct prokaryotes [52]. Transposase genes were not detected in the genome of strains TM300 and 14BME20, although several different groups of transposases were detected (Table 2 and Table S3). Some transposases belonging to six insertion sequence (IS) families were detected and in particular eight transposases of four different types were detected in strain ATCC 15305. IS30 and IS6-like transposases were identified as the most frequent IS element family in the genomes of staphylococci. Although transposases are highly related to the mobility of DNA among genomes, transposases were not generally observed in the 10 CNS species associated with fermented foods. Thus, we conclude that transposases are unlikely to play a crucial role in horizontal gene transfer.

**Plasmid.** Plasmids are vehicles for cross-species horizontal gene transfer. In our previous studies, the genetic elements in plasmids were shown to contribute strain-specific properties [30]. In the current study, two strains, HKUOPL8 and ATCC 15305, possessed one and two plasmids, respectively. The plasmid in strain HKUOPL8 encoded a hypothetical protein. In strain ATCC 15305, two plasmids encoded the water channel aquaporin for osmotic balancing [36]. Virulence genes were not detected in these plasmids. In general, plasmids involved in horizontal gene transfer were small in size. Unfortunately, this characteristic limits the effectiveness of plasmid prediction from whole-genome sequence data [53] [54]. Therefore, more in-depth analysis of plasmids is required to elucidate the acquisition of genes.

Conjugal transfer has been recognized as a key mechanism by which genes, such as antibiotic resistance genes, disseminate. For successful conjugal transfer, plasmids require *oriT*, *relaxase* for the recognition of plasmid, and a type IV secretion system for transfer of the plasmid or DNA into the recipient cell. The *relaxase* gene was detected in three CNS genomes, Mu2, CSM77, and DSM 14167 (Table S3). However, it was difficult to confirm the presence of plasmids due to incomplete genome sequences and difficulties detecting type IV secretion system genes.

### Food-Originated CNS Genomes Contain Few Mobile Elements

As shown in Table 2, mobile genetic elements such as genomic islands, plasmid, and the staphylococcal cassette chromosome were rarely detected in the 10 CNS genomes, with the exception of phage-related genes. Although phage-related genes were detected from one and more CNS, no further *lysin*, *holin*, or known virulence genes for a lytic lifestyle were detected among the 10 CNS genomes.

Compared with other mobile elements, transposase genes were more frequently detected in the 10 CNS genomes; however, associated virulence genes were not detected except in strain ATCC 15305. Most transposase genes (eight genes) were detected in ATCC 15305. However, no virulence genes were detected in the flanking regions of the transposase compared with *S. aureus*, which contained antibiotic resistance genes or virulence factors [55]. Indeed, genes related to mobility for conjugal transfer were detected in three strains, but the secretion system required for transfer was not detected in these strains. Therefore, the results imply that food-originating bacteria are benign and could be changed with acquired gene traits.

In this study, we analysed the genomes of food-originated CNS to determine the genetic basis for the division into species. The 16S rRNA gene sequences were determined for the five CNS species (Fig. 5A) but were too highly conserved to distinguish between species, and the MLST results (Fig. 5B) were similar to those of the16S rRNA phylogenetic tree. Meanwhile, our analysis of the pan-genome of 10 CNS did not show species-specific patterns but strain-specific properties. Based on these results, we postulated that identification of the core genome shared by all CNS isolates provides a new opportunity for determining the phylogenetic relationships among isolates. This phylogenomic analysis showed that *S. carnosus* was differentiated from the other four species (Fig. 5C) and two species, *S. equorum*/*S. succinus* and *S. saprophyticus*/*S. xylosus*, originated from the same ancestor. These results indicated substantial amino acid sequence differences in *S. succinus* compared with other CNS strains. This pan-comparative genomic analysis identified unique genes harbouring strain-specific determinants among the 10 CNS strains and known virulence genes were not detected among the 10 CNS genomes. In this context, this study strongly supports that the four CNS species are highly related and, in general, lack mobile genetic elements. The mobile genetic elements that were identified among the 10 CNS strains contributed to specific genetic properties. This supported the avirulent properties of food-originated CNS. This study supports the usefulness of comparative genomics in the safety assessment and functional analysis of industrial strains including starter candidates for food fermentation.

## Supplemental Materials



Supplementary data for this paper are available on-line only at http://jmb.or.kr.

## Figures and Tables

**Table 1:** General genomic and specific phenotypic features of the 10 coagulase-negative *Staphylococcus* strains.

Species	*S. carnosus*		*S. equorum*		*S. succinus*			*S. xylosus*		*S. saprophyticus*
				
Strain	JCM 6069	TM300	KS1039	Mu2	14BME20	CSM-77	DSM14617	C2a	HKUOPL8	ATCC 15305
Size	2,645,106	2,566,424	2,822,193	2,927,171	2,745,675	2,802,639	2,887,686	2,786,686	2,866,963	2,577,899
GC content %	34.56	34.63	33.07	32.80	33.08	32.95	32.94	32.90	32.73	33.19
No. of plasmids	0	0	0	-	0	-	-	-	1 ^a^	2 ^b^
Predicted CDS	2,676	2,492	2,675	2,745	2,589	2,652	2,831	2,613	2,717	2,503
COG	2,429	2,290	2,431	2,555	2,390	2,451	2,571	2,408	2,502	2,315
SEED	2,002	1,903	2,009	2,045	2,013	2,064	2,088	2,023	2,053	1,918
No. of rRNAs	18	15	22	4	19	15	19	19	13	19
No. of tRNAs	61	60	61	55	61	59	57	59	55	60
Contigs	1	1	1	30	1	11	162	1	2	3
Origin	Pla-chom, fermented fish products	Sausage, meat starter	Saeu-jeot, fermented shrimp	French smear-ripened cheese	Doenjang	Triassic salt mine	Plant and soil inclusions	Human skin	Healthy giant panda faecal material	Young female outpatients
Country	Thailand	France	South Korea	Germany	South Korea	UK	Dominican Republic	Unknown	Hong Kong	Japan
Accession no.	NZ_CP016760	NC_012121	NZ_CP01311	NZ_CAJL00000000	NZ_CP018199	NZ_LUJH01000000	NZ_LCSH00000000	NZ_LN554884	NZ_CP007208	NC_007350
Status	Complete	Complete	Complete	Contig	Complete	Contig	Contig	Complete	Complete	Complete
Reference	[32]	[9]	[12]	[11]	[8]	[33]	[34]	[35]	[37]	[36]

^a^Plasmids in strain HKUOPL8: unnamed, 30.1 kb.

^b^Plasmids in strain ATCC 15305: pSSP1, 38.4 kb; pSSP2, 22.9 kb.

**Table 2:** Mobile elements of 10 coagulase-negative *Staphylococcus* strains.

Species	*S. carnosus*		*S. equorum*		*S. succinus*			*S. xylosus*		*S. saprophyticus*
				
Strain	JCM 6069	TM300	KS1039	Mu2	14BME20	CSM-77	DSM14617	C2a	HKUOPL8	ATCC 15305
Phage	2	1	1	2	1	1	1	1	2	0
Genomic islands	0	0 (1^*^)	0	0	0	0	0	0	0	0 (1^*^)
Plasmid	0	0	0	- (contig)	0	- (contig)	- (contig)	0	1	2
Relaxsome				1		1	1			
Staphylococcal cassette chromosomes	0	0	0		0			0	0	0

^*^Suggested number from a previous study, but gene not detected in this study.
