# Supplementary material for: Comparative Genomic Analysis of Food-Originated Coagulase- Negative Staphylococcus: Analysis of Conserved Core Genes and Diversity of the Pan-Genome
Source: J Microbiol Biotechnol. 2019 Dec 9;30(3):341–51. doi: 10.4014/jmb.1910.10049 (PMC9728283; doi:10.4014/jmb.1910.10049)
Supplement: Supplementary file 1 [file JMB-30-3-341-supple.pdf]

J. Microbiol. Biotechnol.
https://doi.org/10.4014/jmb.1910.10049

Table S1. Fractional pan-genome of 10 CNS genomes

**Product**	***S. carnosus***		***S. equorum***		***S. succinus***			***S. xylosus***		***S. saprophyticus***
	**JCM 6069**	**TM300**	**KS1039**	**Mu2**	**14BME20**	**CSM 77**	**DSM 14617**	**C2a**	**HKUOPL8**	**ATCC 15305**
NAD(P)-dependent oxidoreductase	BEK99_RS00020	SCA_RS11190	SE1039_RS10910	SEQMU2_RS02790	BK815_RS11650	A6V26_RS07690	AA913_RS01395	SXYL_RS02225	BE24_RS09560	SSP_RS02040
FMN-binding glutamate synthase family protein	BEK99_RS00025	SCA_RS11185	SE1039_RS10810	SEQMU2_RS02690	BK815_RS11780	A6V26_RS07560	AA913_RS01265	SXYL_RS02330	BE24_RS09455	SSP_RS02140
drug:proton antiporter	BEK99_RS00030	SCA_RS11180	SE1039_RS10625	SEQMU2_RS02505	BK815_RS11940	A6V26_RS07400	AA913_RS01105	SXYL_RS02520	BE24_RS09255	SSP_RS02360
DUF1445 domain-containing protein	BEK99_RS00060	SCA_RS11150	SE1039_RS11350	SEQMU2_RS03225	BK815_RS11200	A6V26_RS08165	AA913_RS03945	SXYL_RS01685	BE24_RS10090	SSP_RS01500
TetR/AcrR family transcriptional regulator	BEK99_RS00265	SCA_RS10980	SE1039_RS10455	SEQMU2_RS02315	BK815_RS12060	A6V26_RS07280	AA913_RS00980	SXYL_RS02715	BE24_RS09060	SSP_RS02500
carbohydrate kinase	BEK99_RS00270	SCA_RS10975	SE1039_RS08810	SEQMU2_RS00700	BK815_RS00585	A6V26_RS12630	AA913_RS13215	SXYL_RS04375	BE24_RS07360	SSP_RS04170
MarR family transcriptional regulator	BEK99_RS00315	SCA_RS10930	SE1039_RS12135	SEQMU2_RS03980	BK815_RS10515	A6V26_RS08850	AA913_RS09765	SXYL_RS00900	BE24_RS10800	SSP_RS00840
pyruvate decarboxylase	BEK99_RS00320	SCA_RS10925	SE1039_RS12125	SEQMU2_RS03970	BK815_RS10525	A6V26_RS08840	AA913_RS09775	SXYL_RS00905	BE24_RS10795	SSP_RS00845
choline transporter	BEK99_RS00325	SCA_RS10920	SE1039_RS11945	SEQMU2_RS03790	BK815_RS10710	A6V26_RS08655	AA913_RS09960	SXYL_RS01085	BE24_RS10625	SSP_RS00955
GbsR/MarR family transcriptional regulator	BEK99_RS00330	SCA_RS10915	SE1039_RS11940	SEQMU2_RS03785	BK815_RS12615	A6V26_RS06730	AA913_RS00430	SXYL_RS01090	BE24_RS10620	SSP_RS00960
betaine-aldehyde dehydrogenase	BEK99_RS00335	SCA_RS10910	SE1039_RS11935	SEQMU2_RS03780	BK815_RS10720	A6V26_RS08645	AA913_RS09970	SXYL_RS01095	BE24_RS10615	SSP_RS00965
choline dehydrogenase	BEK99_RS00340	SCA_RS10905	SE1039_RS11930	SEQMU2_RS03775	BK815_RS10725	A6V26_RS08640	AA913_RS09975	SXYL_RS01100	BE24_RS10610	SSP_RS00970
hypothetical protein	BEK99_RS00390	SCA_RS10855	SE1039_RS11320	SEQMU2_RS03195	BK815_RS11225	A6V26_RS08140	AA913_RS01845	SXYL_RS01710	BE24_RS10065	SSP_RS01525
class I fructose-bisphosphate aldolase	BEK99_RS00430	SCA_RS10820	SE1039_RS11960	SEQMU2_RS03805	BK815_RS10700	A6V26_RS08665	AA913_RS09950	SXYL_RS01075	BE24_RS10635	SSP_RS00945
membrane protein	BEK99_RS00450	SCA_RS10800	SE1039_RS09270	SEQMU2_RS01145	BK815_RS00130	A6V26_RS11980	AA913_RS11560	SXYL_RS03925	BE24_RS07835	SSP_RS03705
N-acetylmuramic acid 6-phosphate etherase	BEK99_RS00460	SCA_RS10790	SE1039_RS10080	SEQMU2_RS01940	BK815_RS12435	A6V26_RS06910	AA913_RS00610	SXYL_RS03120	BE24_RS08685	SSP_RS02895
permease	BEK99_RS00465	SCA_RS10785	SE1039_RS10085	SEQMU2_RS01945	BK815_RS12430	A6V26_RS06915	AA913_RS00615	SXYL_RS03115	BE24_RS08690	SSP_RS02890
phage integrase	BEK99_RS00510	SCA_RS10740	SE1039_RS02295	SEQMU2_RS07080	BK815_RS07210	A6V26_RS03335	AA913_RS07600	SXYL_RS10900	BE24_RS00830	SSP_RS10495
monovalent cation/H+ antiporter subunit B	BEK99_RS00520	SCA_RS10730	SE1039_RS02305	SEQMU2_RS07090	BK815_RS07200	A6V26_RS03325	AA913_RS07610	SXYL_RS10890	BE24_RS00840	SSP_RS10485
cation:proton antiporter	BEK99_RS00525	SCA_RS10725	SE1039_RS02310	SEQMU2_RS07095	BK815_RS07195	A6V26_RS03320	AA913_RS07615	SXYL_RS10885	BE24_RS00845	SSP_RS10480
Na+/H+ antiporter subunit D	BEK99_RS00530	SCA_RS10720	SE1039_RS02315	SEQMU2_RS07100	BK815_RS07190	A6V26_RS03315	AA913_RS07620	SXYL_RS10880	BE24_RS00850	SSP_RS10475
Na+/H+ antiporter subunit E	BEK99_RS00535	SCA_RS10715	SE1039_RS02320	SEQMU2_RS07105	BK815_RS07185	A6V26_RS03310	AA913_RS07625	SXYL_RS10875	BE24_RS00855	SSP_RS10470
cation:proton antiporter	BEK99_RS00540	SCA_RS10710	SE1039_RS02325	SEQMU2_RS07110	BK815_RS07180	A6V26_RS03305	AA913_RS07630	SXYL_RS10870	BE24_RS00860	SSP_RS10465
cation:proton antiporter	BEK99_RS00545	SCA_RS10705	SE1039_RS02330	SEQMU2_RS07115	BK815_RS07175	A6V26_RS03300	AA913_RS07635	SXYL_RS10865	BE24_RS00865	SSP_RS10460
lactate dehydrogenase	BEK99_RS00555	SCA_RS10695	SE1039_RS11430	SEQMU2_RS03285	BK815_RS11140	A6V26_RS08225	AA913_RS04005	SXYL_RS01595	BE24_RS10150	SSP_RS01435
dihydroxyacetone kinase subunit DhaK	BEK99_RS00595	SCA_RS10660	SE1039_RS02430	SEQMU2_RS07215	BK815_RS07075	A6V26_RS03200	AA913_RS07735	SXYL_RS10765	BE24_RS00965	SSP_RS10360
dihydroxyacetone kinase subunit L	BEK99_RS00600	SCA_RS10655	SE1039_RS02435	SEQMU2_RS07220	BK815_RS07070	A6V26_RS03195	AA913_RS07740	SXYL_RS10760	BE24_RS00970	SSP_RS10355
PTS-dependent dihydroxyacetone kinase phosphotransferase subunit DhaM	BEK99_RS00605	SCA_RS10650	SE1039_RS02440	SEQMU2_RS07225	BK815_RS07065	A6V26_RS03190	AA913_RS07745	SXYL_RS10755	BE24_RS00975	SSP_RS10350

Product	*S. carnosus*		*S. equorum*		*S. succinus*			*S. xylosus*		*S. saprophyticus*
	**JCM 6069**	**TM300**	**KS1039**	**Mu2**	**14BME20**	**CSM 77**	**DSM 14617**	**C2a**	**HKUOPL8**	**ATCC 15305**
CDP-glycerol:glycerophosphate glycerophosphotransferase	BEK99_RS00615	SCA_RS12710	SE1039_RS13610	SEQMU2_RS03760	BK815_RS10745	A6V26_RS08620	AA913_RS14340	SXYL_RS13255	BE24_RS13640	SSP_RS12655
glycosyl transferase	BEK99_RS00650	SCA_RS10605	SE1039_RS02695	SEQMU2_RS07485	BK815_RS06800	A6V26_RS02925	AA913_RS08005	SXYL_RS10490	BE24_RS01245	SSP_RS10080
ABC transporter ATP-binding protein	BEK99_RS00670	SCA_RS10585	SE1039_RS12420	SEQMU2_RS04215	BK815_RS10930	A6V26_RS08435	AA913_RS04215	SXYL_RS00625	BE24_RS11065	SSP_RS00655
ABC transporter permease	BEK99_RS00675	SCA_RS10580	SE1039_RS12415	SEQMU2_RS04210	BK815_RS10935	A6V26_RS08430	AA913_RS04210	SXYL_RS00630	BE24_RS11060	SSP_RS00660
MFS transporter	BEK99_RS00685	SCA_RS10570	SE1039_RS00115	SEQMU2_RS05705	BK815_RS10275	A6V26_RS08555	AA913_RS10060	SXYL_RS01930	BE24_RS10500	SSP_RS10895
3-oxoacyl-ACP reductase	BEK99_RS00690	SCA_RS10565	SE1039_RS00120	SEQMU2_RS05710	BK815_RS12075	A6V26_RS07265	AA913_RS00965	SXYL_RS02730	BE24_RS09045	SSP_RS02515
2-haloalkanoic acid dehalogenase	BEK99_RS00700	SCA_RS10555	SE1039_RS11125	SEQMU2_RS03005	BK815_RS11420	A6V26_RS07940	AA913_RS01645	SXYL_RS01950	BE24_RS09835	SSP_RS01795
acetyl-CoA acetyltransferase	BEK99_RS00715	SCA_RS10540	SE1039_RS02055	SEQMU2_RS06840	BK815_RS07450	A6V26_RS03575	AA913_RS11195	SXYL_RS11135	BE24_RS00625	SSP_RS10725
pantoate--beta-alanine ligase	BEK99_RS00790	SCA_RS10465	SE1039_RS12200	SEQMU2_RS04060	BK815_RS10430	A6V26_RS06270	AA913_RS12655	SXYL_RS00825	BE24_RS10875	SSP_RS00760
2-dehydropantoate 2-reductase	BEK99_RS00800	SCA_RS10455	SE1039_RS12210	SEQMU2_RS04070	BK815_RS10420	A6V26_RS06260	AA913_RS12665	SXYL_RS00815	BE24_RS10885	SSP_RS00750
PTS transporter subunit IIC	BEK99_RS00855	SCA_RS10330	SE1039_RS11445	SEQMU2_RS03300	BK815_RS11125	A6V26_RS08240	AA913_RS04020	SXYL_RS01580	BE24_RS10165	SSP_RS01420
allophanate hydrolase subunit 1	BEK99_RS00860	SCA_RS10325	SE1039_RS07155	SEQMU2_RS12265	BK815_RS02335	A6V26_RS10045	AA913_RS05190	SXYL_RS06170	BE24_RS05470	SSP_RS05870
acetyl-CoA carboxylase biotin carboxyl carrier protein	BEK99_RS13030	SCA_RS10315	SE1039_RS07145	SEQMU2_RS12255	BK815_RS02345	A6V26_RS10035	AA913_RS05180	SXYL_RS06180	BE24_RS05460	SSP_RS05880
acetyl-CoA carboxylase biotin carboxylase subunit	BEK99_RS00870	SCA_RS10310	SE1039_RS07140	SEQMU2_RS12250	BK815_RS02350	A6V26_RS10030	AA913_RS05175	SXYL_RS06185	BE24_RS05455	SSP_RS05885
hypothetical protein	BEK99_RS00875	SCA_RS10305	SE1039_RS07135	SEQMU2_RS12245	BK815_RS02355	A6V26_RS10025	AA913_RS05170	SXYL_RS06190	BE24_RS05450	SSP_RS05890
divalent metal cation transporter	BEK99_RS00880	SCA_RS10300	SE1039_RS07130	SEQMU2_RS12240	BK815_RS02360	A6V26_RS10020	AA913_RS05165	SXYL_RS06195	BE24_RS05445	SSP_RS05895
xylulokinase	BEK99_RS00885	SCA_RS10295	SE1039_RS12305	SEQMU2_RS04165	BK815_RS10210	A6V26_RS06050	AA913_RS12615	SXYL_RS01935	BE24_RS10990	SSP_RS01780
universal stress protein	BEK99_RS00940	SCA_RS10240	SE1039_RS11505	SEQMU2_RS03360	BK815_RS11035	A6V26_RS08330	AA913_RS04110	SXYL_RS01515	BE24_RS10230	SSP_RS01350
DUF3021 domain-containing protein	BEK99_RS00960	SCA_RS10220	SE1039_RS00350	SEQMU2_RS05855	BK815_RS10025	A6V26_RS05865	AA913_RS10360	SXYL_RS12960	BE24_RS12270	SSP_RS12155
hypothetical protein	BEK99_RS00965	SCA_RS10215	SE1039_RS11455	SEQMU2_RS03310	BK815_RS11115	A6V26_RS08250	AA913_RS04030	SXYL_RS01570	BE24_RS10175	SSP_RS01410
SAM-dependent methyltransferase	BEK99_RS00980	SCA_RS10200	SE1039_RS00455	SEQMU2_RS05960	BK815_RS08730	A6V26_RS04565	AA913_RS06985	SXYL_RS12850	BE24_RS12375	SSP_RS12035
pyrimidine-nucleoside phosphorylase	BEK99_RS00995	SCA_RS10185	SE1039_RS09280	SEQMU2_RS01155	BK815_RS00120	A6V26_RS11990	AA913_RS11570	SXYL_RS03915	BE24_RS07845	SSP_RS03695
phosphopentomutase	BEK99_RS01000	SCA_RS10180	SE1039_RS09275	SEQMU2_RS01150	BK815_RS00125	A6V26_RS11985	AA913_RS11565	SXYL_RS03920	BE24_RS07840	SSP_RS03700
fructosamine-3-kinase	BEK99_RS01010	SCA_RS10170	SE1039_RS11495	SEQMU2_RS03350	BK815_RS11045	A6V26_RS08320	AA913_RS04100	SXYL_RS01525	BE24_RS10220	SSP_RS01360
class A sortase SrtA	BEK99_RS01040	SCA_RS10140	SE1039_RS11115	SEQMU2_RS02995	BK815_RS11440	A6V26_RS07920	AA913_RS01625	SXYL_RS01965	BE24_RS09820	SSP_RS01810
methylated-DNA--protein-cysteine methyltransferase	BEK99_RS01055	SCA_RS10125	SE1039_RS11280	SEQMU2_RS03155	BK815_RS11260	A6V26_RS08105	AA913_RS01810	SXYL_RS01750	BE24_RS10025	SSP_RS01565
peptide ABC transporter ATP-binding protein	BEK99_RS01075	SCA_RS10105	SE1039_RS11410	SEQMU2_RS03265	BK815_RS11160	A6V26_RS08205	AA913_RS03985	SXYL_RS12745	BE24_RS12485	SSP_RS01455
LysR family transcriptional regulator	BEK99_RS01085	SCA_RS10095	SE1039_RS11245	SEQMU2_RS03120	BK815_RS11295	A6V26_RS08070	AA913_RS01775	SXYL_RS01785	BE24_RS09980	SSP_RS01605
CidA/LrgA family protein	BEK99_RS01090	SCA_RS10090	SE1039_RS11240	SEQMU2_RS03115	BK815_RS11300	A6V26_RS08065	AA913_RS01770	SXYL_RS01790	BE24_RS09975	SSP_RS01610
LrgB family protein	BEK99_RS01095	SCA_RS10085	SE1039_RS11235	SEQMU2_RS03110	BK815_RS11305	A6V26_RS08060	AA913_RS01765	SXYL_RS01795	BE24_RS09970	SSP_RS01615
pyruvate oxidase	BEK99_RS01100	SCA_RS10080	SE1039_RS11230	SEQMU2_RS03105	BK815_RS11310	A6V26_RS08055	AA913_RS01760	SXYL_RS01800	BE24_RS09965	SSP_RS01620

Product	*S. carnosus*		*S. equorum*		*S. succinus*			*S. xylosus*		*S. saprophyticus*
	**JCM 6069**	**TM300**	**KS1039**	**Mu2**	**14BME20**	**CSM 77**	**DSM 14617**	**C2a**	**HKUOPL8**	**ATCC 15305**
GNAT family N-acetyltransferase	BEK99_RS01110	SCA_RS10070	SE1039_RS11215	SEQMU2_RS03090	BK815_RS11325	A6V26_RS08040	AA913_RS01745	SXYL_RS01815	BE24_RS09950	SSP_RS01640
L-serine ammonia-lyase iron-sulfur-dependent subunit alpha	BEK99_RS01160	SCA_RS10020	SE1039_RS05180	SEQMU2_RS10030	BK815_RS04700	A6V26_RS00860	AA913_RS03070	SXYL_RS12390	BE24_RS12800	SSP_RS07820
NAD(P)H-dependent oxidoreductase	BEK99_RS01170	SCA_RS10010	SE1039_RS11080	SEQMU2_RS02960	BK815_RS11480	A6V26_RS07880	AA913_RS01585	SXYL_RS02005	BE24_RS09780	SSP_RS01850
ABC transporter substrate-binding protein	BEK99_RS01195	SCA_RS09985	SE1039_RS11050	SEQMU2_RS02930	BK815_RS11500	A6V26_RS07840	AA913_RS01545	SXYL_RS02035	BE24_RS09750	SSP_RS01895
MarR family transcriptional regulator	BEK99_RS01200	SCA_RS09980	SE1039_RS11040	SEQMU2_RS02920	BK815_RS11510	A6V26_RS07830	AA913_RS01535	SXYL_RS02045	BE24_RS09740	SSP_RS01905
ring-cleaving dioxygenase mhqO	BEK99_RS01205	SCA_RS09975	SE1039_RS11030	SEQMU2_RS02910	BK815_RS11520	A6V26_RS07820	AA913_RS01525	SXYL_RS02055	BE24_RS09730	SSP_RS01915
melibiose:sodium transporter MelB	BEK99_RS01210	SCA_RS09970	SE1039_RS12750	SEQMU2_RS04625	BK815_RS11540	A6V26_RS07800	AA913_RS01505	SXYL_RS00405	BE24_RS11305	SSP_RS00520
AraC family transcriptional regulator	BEK99_RS01215	SCA_RS09965	SE1039_RS12760	SEQMU2_RS04630	BK815_RS11545	A6V26_RS07795	AA913_RS01500	SXYL_RS00400	BE24_RS11310	SSP_RS00515
fructose-bisphosphatase class III	BEK99_RS01225	SCA_RS09955	SE1039_RS11010	SEQMU2_RS02890	BK815_RS11560	A6V26_RS07780	AA913_RS01485	SXYL_RS02085	BE24_RS09700	SSP_RS01945
antibiotic biosynthesis monooxygenase	BEK99_RS01250	SCA_RS09930	SE1039_RS11000	SEQMU2_RS02880	BK815_RS11580	A6V26_RS07760	AA913_RS01465	SXYL_RS02105	BE24_RS09680	SSP_RS01965
alpha-acetolactate decarboxylase	BEK99_RS01255	SCA_RS09925	SE1039_RS10995	SEQMU2_RS02875	BK815_RS11585	A6V26_RS07755	AA913_RS01460	SXYL_RS02110	BE24_RS09675	SSP_RS00425
FUSC family protein	BEK99_RS01260	SCA_RS09920	SE1039_RS10990	SEQMU2_RS02870	BK815_RS11590	A6V26_RS07750	AA913_RS01455	SXYL_RS02115	BE24_RS09670	SSP_RS01970
GTP pyrophosphokinase	BEK99_RS01275	SCA_RS09905	SE1039_RS10970	SEQMU2_RS02850	BK815_RS11600	A6V26_RS07740	AA913_RS01890	SXYL_RS02135	BE24_RS09650	SSP_RS01990
GntR family transcriptional regulator	BEK99_RS01295	SCA_RS09885	SE1039_RS10965	SEQMU2_RS02845	BK815_RS11605	A6V26_RS07735	AA913_RS01440	SXYL_RS02145	BE24_RS09640	SSP_RS02000
gluconokinase	BEK99_RS01300	SCA_RS09880	SE1039_RS10960	SEQMU2_RS02840	BK815_RS11610	A6V26_RS07730	AA913_RS01435	SXYL_RS02150	BE24_RS09635	SSP_RS02005
gluconate permease	BEK99_RS01305	SCA_RS09875	SE1039_RS10955	SEQMU2_RS02835	BK815_RS11615	A6V26_RS07725	AA913_RS01430	SXYL_RS02155	BE24_RS09630	SSP_RS02010
RNA degradosome polyphosphate kinase	BEK99_RS01320	SCA_RS09860	SE1039_RS10835	SEQMU2_RS02715	BK815_RS11725	A6V26_RS07615	AA913_RS01320	SXYL_RS02300	BE24_RS09485	SSP_RS02110
methionine ABC transporter ATP-binding protein	BEK99_RS01345	SCA_RS09835	SE1039_RS10800	SEQMU2_RS02680	BK815_RS11790	A6V26_RS07550	AA913_RS01255	SXYL_RS02340	BE24_RS09445	SSP_RS02155
iron export ABC transporter permease subunit FetB	BEK99_RS01350	SCA_RS09830	SE1039_RS10795	SEQMU2_RS02675	BK815_RS11795	A6V26_RS07545	AA913_RS01250	SXYL_RS02345	BE24_RS09440	SSP_RS02160
MFS transporter	BEK99_RS01355	SCA_RS09825	SE1039_RS10790	SEQMU2_RS02670	BK815_RS11800	A6V26_RS07540	AA913_RS01245	SXYL_RS02350	BE24_RS09435	SSP_RS02165
choline ABC transporter permease	BEK99_RS01380	SCA_RS09800	SE1039_RS10750	SEQMU2_RS02630	BK815_RS11840	A6V26_RS07500	AA913_RS01205	SXYL_RS02395	BE24_RS09390	SSP_RS02205
osmoprotectant ABC transporter substrate-binding protein	BEK99_RS01385	SCA_RS09795	SE1039_RS10745	SEQMU2_RS02625	BK815_RS11845	A6V26_RS07495	AA913_RS01200	SXYL_RS02400	BE24_RS09385	SSP_RS02210
amino acid ABC transporter permease	BEK99_RS01390	SCA_RS09790	SE1039_RS10740	SEQMU2_RS02620	BK815_RS11850	A6V26_RS07490	AA913_RS01195	SXYL_RS02405	BE24_RS09380	SSP_RS02215
tributyrin esterase	BEK99_RS01395	SCA_RS09785	SE1039_RS10735	SEQMU2_RS02615	BK815_RS11855	A6V26_RS07485	AA913_RS01190	SXYL_RS02410	BE24_RS09375	SSP_RS02220
quinolone resistance protein	BEK99_RS01435	SCA_RS09745	SE1039_RS11310	SEQMU2_RS03165	BK815_RS11235	A6V26_RS08130	AA913_RS01835	SXYL_RS02435	BE24_RS09350	SSP_RS02270
hypothetical protein	BEK99_RS01465	SCA_RS09720	SE1039_RS10670	SEQMU2_RS02550	BK815_RS11910	A6V26_RS07430	AA913_RS01135	SXYL_RS02485	BE24_RS09300	SSP_RS02325
Bcr/CflA family drug resistance efflux transporter	BEK99_RS01470	SCA_RS09715	SE1039_RS10660	SEQMU2_RS02540	BK815_RS11915	A6V26_RS07425	AA913_RS01130	SXYL_RS02495	BE24_RS09290	SSP_RS02335
GtrA family protein	BEK99_RS01475	SCA_RS09710	SE1039_RS10655	SEQMU2_RS02535	BK815_RS11920	A6V26_RS07420	AA913_RS01125	SXYL_RS02500	BE24_RS09285	SSP_RS02340
cation transporter	BEK99_RS01480	SCA_RS09705	SE1039_RS10650	SEQMU2_RS02530	BK815_RS11925	A6V26_RS07415	AA913_RS01120	SXYL_RS02505	BE24_RS09280	SSP_RS02345
hypothetical protein	BEK99_RS01485	SCA_RS09700	SE1039_RS10645	SEQMU2_RS02525	BK815_RS11930	A6V26_RS07410	AA913_RS01115	SXYL_RS02510	BE24_RS09265	SSP_RS02350
2,3-bisphosphoglycerate-dependent phosphoglycerate mutase	BEK99_RS01490	SCA_RS09695	SE1039_RS10640	SEQMU2_RS02520	BK815_RS11935	A6V26_RS07405	AA913_RS01110	SXYL_RS02515	BE24_RS09260	SSP_RS02355

Product	*S. carnosus*		*S. equorum*		*S. succinus*			*S. xylosus*		*S. saprophyticus*
	**JCM 6069**	**TM300**	**KS1039**	**Mu2**	**14BME20**	**CSM 77**	**DSM 14617**	**C2a**	**HKUOPL8**	**ATCC 15305**
protein-disulfide isomerase	BEK99_RS01510	SCA_RS09675	SE1039_RS10610	SEQMU2_RS02490	BK815_RS11960	A6V26_RS07380	AA913_RS01085	SXYL_RS02535	BE24_RS09240	SSP_RS02385
polyisoprenoid-binding protein	BEK99_RS01530	SCA_RS09655	SE1039_RS10600	SEQMU2_RS02480	BK815_RS11970	A6V26_RS07370	AA913_RS01075	SXYL_RS02545	BE24_RS09230	SSP_RS02395
SRPBCC domain-containing protein	BEK99_RS01535	SCA_RS09650	SE1039_RS10580	SEQMU2_RS02460	BK815_RS11980	A6V26_RS07360	AA913_RS01065	SXYL_RS02565	BE24_RS09210	SSP_RS02415
formate/nitrite transporter	BEK99_RS01545	SCA_RS09640	SE1039_RS10570	SEQMU2_RS02430	BK815_RS12005	A6V26_RS07335	AA913_RS01035	SXYL_RS02590	BE24_RS09185	SSP_RS02435
hydroxymethylglutaryl-CoA synthase	BEK99_RS01620	SCA_RS09565	SE1039_RS11270	SEQMU2_RS03145	BK815_RS11265	A6V26_RS08100	AA913_RS01805	SXYL_RS01755	BE24_RS10010	SSP_RS01575
3-ketoacyl-CoA thiolase	BEK99_RS01625	SCA_RS09560	SE1039_RS11265	SEQMU2_RS03140	BK815_RS11270	A6V26_RS08095	AA913_RS01800	SXYL_RS01760	BE24_RS10005	SSP_RS01580
hydroxymethylglutaryl-CoA reductase degradative	BEK99_RS01630	SCA_RS09555	SE1039_RS11260	SEQMU2_RS03135	BK815_RS11275	A6V26_RS08090	AA913_RS01795	SXYL_RS01765	BE24_RS10000	SSP_RS01585
hsp20/alpha crystallin family protein	BEK99_RS01635	SCA_RS09550	SE1039_RS10495	SEQMU2_RS02355	BK815_RS12015	A6V26_RS07325	AA913_RS01025	SXYL_RS02670	BE24_RS09105	SSP_RS02450
MarR family transcriptional regulator	BEK99_RS01640	SCA_RS09545	SE1039_RS10490	SEQMU2_RS02350	BK815_RS12025	A6V26_RS07315	AA913_RS01015	SXYL_RS02680	BE24_RS09095	SSP_RS02460
hypothetical protein	BEK99_RS01645	SCA_RS09540	SE1039_RS10485	SEQMU2_RS02345	BK815_RS12030	A6V26_RS07310	AA913_RS01010	SXYL_RS02685	BE24_RS09090	SSP_RS02465
DUF4889 domain-containing protein	BEK99_RS01660	SCA_RS09525	SE1039_RS10480	SEQMU2_RS02340	BK815_RS12040	A6V26_RS07300	AA913_RS01000	SXYL_RS02690	BE24_RS09085	SSP_RS02475
magnesium transporter CorA	BEK99_RS01670	SCA_RS09515	SE1039_RS10460	SEQMU2_RS02320	BK815_RS12055	A6V26_RS07285	AA913_RS00985	SXYL_RS02710	BE24_RS09065	SSP_RS02495
membrane protein	BEK99_RS01680	SCA_RS09505	SE1039_RS10390	SEQMU2_RS02250	BK815_RS12130	A6V26_RS07215	AA913_RS00915	SXYL_RS02775	BE24_RS09000	SSP_RS02560
NAD(P)/FAD-dependent oxidoreductase	BEK99_RS01685	SCA_RS09500	SE1039_RS10385	SEQMU2_RS02245	BK815_RS12135	A6V26_RS07210	AA913_RS00910	SXYL_RS02780	BE24_RS08995	SSP_RS02570
MFS transporter	BEK99_RS01690	SCA_RS09495	SE1039_RS10375	SEQMU2_RS02235	BK815_RS12145	A6V26_RS07200	AA913_RS00900	SXYL_RS02790	BE24_RS08985	SSP_RS02580
quinone oxidoreductase	BEK99_RS01695	SCA_RS09490	SE1039_RS10370	SEQMU2_RS02230	BK815_RS12150	A6V26_RS07195	AA913_RS00895	SXYL_RS02795	BE24_RS08980	SSP_RS02585
hypothetical protein	BEK99_RS01700	SCA_RS09485	SE1039_RS10360	SEQMU2_RS02220	BK815_RS12165	A6V26_RS07180	AA913_RS00880	SXYL_RS02805	BE24_RS08970	SSP_RS02595
teichoic acid biosynthesis protein F	BEK99_RS01705	SCA_RS09480	SE1039_RS10355	SEQMU2_RS02215	BK815_RS12170	A6V26_RS07175	AA913_RS00875	SXYL_RS02810	BE24_RS08965	SSP_RS02600
L-lactate permease	BEK99_RS01710	SCA_RS09475	SE1039_RS10350	SEQMU2_RS02210	BK815_RS12175	A6V26_RS07170	AA913_RS00870	SXYL_RS02815	BE24_RS08960	SSP_RS02605
glycoside hydrolase	BEK99_RS01715	SCA_RS09470	SE1039_RS10345	SEQMU2_RS02205	BK815_RS12180	A6V26_RS07165	AA913_RS00865	SXYL_RS02820	BE24_RS08955	SSP_RS02610
DNA-binding response regulator	BEK99_RS01730	SCA_RS09455	SE1039_RS10330	SEQMU2_RS02190	BK815_RS12195	A6V26_RS07150	AA913_RS00850	SXYL_RS02850	BE24_RS08940	SSP_RS02625
ABC transporter permease	BEK99_RS01735	SCA_RS09450	SE1039_RS10325	SEQMU2_RS02185	BK815_RS12200	A6V26_RS07145	AA913_RS00845	SXYL_RS02855	BE24_RS08935	SSP_RS02630
ABC transporter ATP-binding protein	BEK99_RS01740	SCA_RS09445	SE1039_RS10320	SEQMU2_RS02180	BK815_RS12205	A6V26_RS07140	AA913_RS00840	SXYL_RS02860	BE24_RS08930	SSP_RS02635
hypothetical protein	BEK99_RS01745	SCA_RS09440	SE1039_RS10315	SEQMU2_RS02175	BK815_RS12210	A6V26_RS07135	AA913_RS00835	SXYL_RS02865	BE24_RS08925	SSP_RS02640
MarR family transcriptional regulator	BEK99_RS01750	SCA_RS09435	SE1039_RS10310	SEQMU2_RS02170	BK815_RS12215	A6V26_RS07130	AA913_RS00830	SXYL_RS02870	BE24_RS08920	SSP_RS02645
TcaA protein	BEK99_RS01755	SCA_RS09430	SE1039_RS10305	SEQMU2_RS02165	BK815_RS12220	A6V26_RS07125	AA913_RS00825	SXYL_RS02875	BE24_RS08915	SSP_RS02650
Bcr/CflA family drug resistance efflux transporter	BEK99_RS01760	SCA_RS09425	SE1039_RS10300	SEQMU2_RS02160	BK815_RS12225	A6V26_RS07120	AA913_RS00820	SXYL_RS02880	BE24_RS08910	SSP_RS02655
HlyD family secretion protein	BEK99_RS01775	SCA_RS09410	SE1039_RS10290	SEQMU2_RS02150	BK815_RS12235	A6V26_RS07110	AA913_RS00810	SXYL_RS02890	BE24_RS08900	SSP_RS02665
multidrug MFS transporter	BEK99_RS01780	SCA_RS09405	SE1039_RS10285	SEQMU2_RS02145	BK815_RS12240	A6V26_RS07105	AA913_RS00805	SXYL_RS02895	BE24_RS08895	SSP_RS02670
3-hydroxyacyl-CoA dehydrogenase	BEK99_RS01795	SCA_RS09390	SE1039_RS10265	SEQMU2_RS02125	BK815_RS12260	A6V26_RS07085	AA913_RS00785	SXYL_RS02915	BE24_RS08875	SSP_RS02690
magnesium and cobalt transport protein CorA	BEK99_RS01800	SCA_RS09385	SE1039_RS10260	SEQMU2_RS02120	BK815_RS12265	A6V26_RS07080	AA913_RS00780	SXYL_RS02920	BE24_RS08870	SSP_RS02695

**Product**	***S. carnosus***		***S. equorum***		***S. succinus***			***S. xylosus***		***S. saprophyticus***
	**JCM 6069**	**TM300**	**KS1039**	**Mu2**	**14BME20**	**CSM 77**	**DSM 14617**	**C2a**	**HKUOPL8**	**ATCC 15305**
type 2 isopentenyl-diphosphate Delta-isomerase	BEK99_RS01805	SCA_RS09380	SE1039_RS10255	SEQMU2_RS02115	BK815_RS12270	A6V26_RS07075	AA913_RS00775	SXYL_RS02925	BE24_RS08865	SSP_RS02700
DNA-3-methyladenine glycosylase	BEK99_RS01825	SCA_RS09360	SE1039_RS10240	SEQMU2_RS02100	BK815_RS12280	A6V26_RS07065	AA913_RS00765	SXYL_RS02955	BE24_RS08850	SSP_RS02725
sodium ABC transporter ATP-binding protein	BEK99_RS01855	SCA_RS09325	SE1039_RS10230	SEQMU2_RS02090	BK815_RS12295	A6V26_RS07050	AA913_RS00750	SXYL_RS02970	BE24_RS08835	SSP_RS02740
sodium ABC transporter permease	BEK99_RS01860	SCA_RS09320	SE1039_RS10225	SEQMU2_RS02085	BK815_RS12300	A6V26_RS07045	AA913_RS00745	SXYL_RS02975	BE24_RS08830	SSP_RS02745
galactose mutarotase	BEK99_RS01870	SCA_RS09310	SE1039_RS10220	SEQMU2_RS02080	BK815_RS12305	A6V26_RS07040	AA913_RS00740	SXYL_RS02980	BE24_RS08825	SSP_RS02750
ribose-5-phosphate isomerase	BEK99_RS01875	SCA_RS09305	SE1039_RS10210	SEQMU2_RS02070	BK815_RS12315	A6V26_RS07030	AA913_RS00730	SXYL_RS02990	BE24_RS08815	SSP_RS02760
CPBP family intramembrane metalloprotease	BEK99_RS01880	SCA_RS09300	SE1039_RS10205	SEQMU2_RS02065	BK815_RS12320	A6V26_RS07025	AA913_RS00725	SXYL_RS02995	BE24_RS08810	SSP_RS02765
LysR family transcriptional regulator	BEK99_RS01890	SCA_RS09290	SE1039_RS10195	SEQMU2_RS02055	BK815_RS12330	A6V26_RS07015	AA913_RS00715	SXYL_RS03005	BE24_RS08800	SSP_RS02775
urocanate hydratase	BEK99_RS01900	SCA_RS09280	SE1039_RS10185	SEQMU2_RS02045	BK815_RS12335	A6V26_RS07010	AA913_RS00710	SXYL_RS03015	BE24_RS08790	SSP_RS02780
SulP family inorganic anion transporter	BEK99_RS01910	SCA_RS09270	SE1039_RS10175	SEQMU2_RS02035	BK815_RS12345	A6V26_RS07000	AA913_RS00700	SXYL_RS03025	BE24_RS08780	SSP_RS02790
amidohydrolase	BEK99_RS01915	SCA_RS09265	SE1039_RS10170	SEQMU2_RS02030	BK815_RS12350	A6V26_RS06995	AA913_RS00695	SXYL_RS03030	BE24_RS08775	SSP_RS02795
oxidoreductase	BEK99_RS01920	SCA_RS09260	SE1039_RS10165	SEQMU2_RS03255	BK815_RS12355	A6V26_RS06990	AA913_RS00690	SXYL_RS03035	BE24_RS08770	SSP_RS02800
hypothetical protein	BEK99_RS01925	SCA_RS09255	SE1039_RS10160	SEQMU2_RS02020	BK815_RS10695	A6V26_RS08670	AA913_RS09945	SXYL_RS03040	BE24_RS08765	SSP_RS02805
sodium:proton antiporter	BEK99_RS01930	SCA_RS09250	SE1039_RS10155	SEQMU2_RS02015	BK815_RS12360	A6V26_RS06985	AA913_RS00685	SXYL_RS03045	BE24_RS08760	SSP_RS02810
SRPBCC domain-containing protein	BEK99_RS01935	SCA_RS09245	SE1039_RS10150	SEQMU2_RS02010	BK815_RS12365	A6V26_RS06980	AA913_RS00680	SXYL_RS03050	BE24_RS08755	SSP_RS02815
hypothetical protein	BEK99_RS01945	SCA_RS09235	SE1039_RS10135	SEQMU2_RS01995	BK815_RS12380	A6V26_RS06965	AA913_RS00665	SXYL_RS03065	BE24_RS08740	SSP_RS02840
haloacid dehalogenase	BEK99_RS01950	SCA_RS09230	SE1039_RS10125	SEQMU2_RS01985	BK815_RS12390	A6V26_RS06955	AA913_RS00655	SXYL_RS03075	BE24_RS08730	SSP_RS02850
hypothetical protein	BEK99_RS01955	SCA_RS09225	SE1039_RS10120	SEQMU2_RS01980	BK815_RS12395	A6V26_RS06950	AA913_RS00650	SXYL_RS03080	BE24_RS08725	SSP_RS02855
hypothetical protein	BEK99_RS01960	SCA_RS09220	SE1039_RS10115	SEQMU2_RS01975	BK815_RS12400	A6V26_RS06945	AA913_RS00645	SXYL_RS03085	BE24_RS08720	SSP_RS02860
hypothetical protein	BEK99_RS01965	SCA_RS09215	SE1039_RS10110	SEQMU2_RS01970	BK815_RS12405	A6V26_RS06940	AA913_RS00640	SXYL_RS03090	BE24_RS08715	SSP_RS02865
formate/nitrite transporter	BEK99_RS01970	SCA_RS09210	SE1039_RS10105	SEQMU2_RS01965	BK815_RS12410	A6V26_RS06935	AA913_RS00635	SXYL_RS03095	BE24_RS08710	SSP_RS02870
gamma-aminobutyrate permease	BEK99_RS01975	SCA_RS09205	SE1039_RS10100	SEQMU2_RS01960	BK815_RS12415	A6V26_RS06930	AA913_RS00630	SXYL_RS03100	BE24_RS08705	SSP_RS02875
DNA-binding transcriptional regulator	BEK99_RS01995	SCA_RS09185	SE1039_RS10065	SEQMU2_RS01925	BK815_RS12455	A6V26_RS06890	AA913_RS00590	SXYL_RS03135	BE24_RS08670	SSP_RS02910
inositol monophosphatase	BEK99_RS02000	SCA_RS09180	SE1039_RS10060	SEQMU2_RS01920	BK815_RS12460	A6V26_RS06885	AA913_RS00585	SXYL_RS03140	BE24_RS08665	SSP_RS02915
LytR family transcriptional regulator	BEK99_RS02005	SCA_RS09175	SE1039_RS10055	SEQMU2_RS01915	BK815_RS12465	A6V26_RS06880	AA913_RS00580	SXYL_RS03145	BE24_RS08660	SSP_RS02920
formate dehydrogenase subunit alpha	BEK99_RS02015	SCA_RS09165	SE1039_RS10050	SEQMU2_RS01910	BK815_RS12470	A6V26_RS06875	AA913_RS00575	SXYL_RS03150	BE24_RS08655	SSP_RS02925
hypothetical protein	BEK99_RS02020	SCA_RS09160	SE1039_RS10045	SEQMU2_RS01905	BK815_RS12475	A6V26_RS06870	AA913_RS00570	SXYL_RS03155	BE24_RS08650	SSP_RS02930
hypothetical protein	BEK99_RS02025	SCA_RS09155	SE1039_RS10040	SEQMU2_RS01900	BK815_RS12480	A6V26_RS06865	AA913_RS00565	SXYL_RS03160	BE24_RS08645	SSP_RS02935
autolysin	BEK99_RS02030	SCA_RS09150	SE1039_RS10035	SEQMU2_RS01895	BK815_RS12485	A6V26_RS06860	AA913_RS00560	SXYL_RS03165	BE24_RS08640	SSP_RS02940
hypothetical protein	BEK99_RS02035	SCA_RS09145	SE1039_RS10030	SEQMU2_RS01890	BK815_RS12490	A6V26_RS06855	AA913_RS00555	SXYL_RS03170	BE24_RS08635	SSP_RS02945
2-hydroxyacid dehydrogenase	BEK99_RS02040	SCA_RS09140	SE1039_RS10025	SEQMU2_RS01885	BK815_RS12495	A6V26_RS06850	AA913_RS00550	SXYL_RS03175	BE24_RS08630	SSP_RS02950

Product	*S. carnosus*		*S. equorum*		*S. succinus*			*S. xylosus*		*S. saprophyticus*
	**JCM 6069**	**TM300**	**KS1039**	**Mu2**	**14BME20**	**CSM 77**	**DSM 14617**	**C2a**	**HKUOPL8**	**ATCC 15305**
QacE family quaternary ammonium compound efflux SMR transporter	BEK99_RS02045	SCA_RS09135	SE1039_RS10020	SEQMU2_RS01880	BK815_RS12500	A6V26_RS06845	AA913_RS00545	SXYL_RS03180	BE24_RS08625	SSP_RS02955
multidrug resistance protein SMR	BEK99_RS02050	SCA_RS09130	SE1039_RS10015	SEQMU2_RS01875	BK815_RS12505	A6V26_RS06840	AA913_RS00540	SXYL_RS03185	BE24_RS08620	SSP_RS02960
DUF4870 domain-containing protein	BEK99_RS02055	SCA_RS09125	SE1039_RS10005	SEQMU2_RS01865	BK815_RS12515	A6V26_RS06830	AA913_RS00530	SXYL_RS03195	BE24_RS08610	SSP_RS02970
octopine dehydrogenase	BEK99_RS02060	SCA_RS09120	SE1039_RS10000	SEQMU2_RS01860	BK815_RS12520	A6V26_RS06825	AA913_RS00525	SXYL_RS03200	BE24_RS08605	SSP_RS02975
Na+/H+ antiporter NhaC	BEK99_RS02065	SCA_RS09115	SE1039_RS09995	SEQMU2_RS01855	BK815_RS12525	A6V26_RS06820	AA913_RS00520	SXYL_RS03205	BE24_RS08600	SSP_RS02980
CHAP domain-containing protein	BEK99_RS02075	SCA_RS09105	SE1039_RS09985	SEQMU2_RS01845	BK815_RS12530	A6V26_RS06815	AA913_RS00515	SXYL_RS03210	BE24_RS08595	SSP_RS02985
hypothetical protein	BEK99_RS02085	SCA_RS09095	SE1039_RS09975	SEQMU2_RS01835	BK815_RS12540	A6V26_RS06805	AA913_RS00505	SXYL_RS03220	BE24_RS08585	SSP_RS02995
accessory regulator A	BEK99_RS02090	SCA_RS09090	SE1039_RS09970	SEQMU2_RS01830	BK815_RS12545	A6V26_RS06800	AA913_RS00500	SXYL_RS03225	BE24_RS08580	SSP_RS03000
urease accessory protein UreG	BEK99_RS02100	SCA_RS09080	SE1039_RS11585	SEQMU2_RS03440	BK815_RS10955	A6V26_RS08410	AA913_RS04190	SXYL_RS01425	BE24_RS10320	SSP_RS01265
urease accessory protein UreF	BEK99_RS02105	SCA_RS09075	SE1039_RS11580	SEQMU2_RS03435	BK815_RS10960	A6V26_RS08405	AA913_RS04185	SXYL_RS01430	BE24_RS10315	SSP_RS01270
urease accessory protein UreE	BEK99_RS02110	SCA_RS09070	SE1039_RS11575	SEQMU2_RS03430	BK815_RS10965	A6V26_RS08400	AA913_RS04180	SXYL_RS01435	BE24_RS10310	SSP_RS01275
urease subunit alpha	BEK99_RS02115	SCA_RS09065	SE1039_RS11570	SEQMU2_RS03425	BK815_RS10970	A6V26_RS08395	AA913_RS04175	SXYL_RS01440	BE24_RS10305	SSP_RS01280
urea transporter	BEK99_RS02120	SCA_RS09050	SE1039_RS11555	SEQMU2_RS03410	BK815_RS10985	A6V26_RS08380	AA913_RS04160	SXYL_RS01455	BE24_RS10290	SSP_RS01295
ferrichrome ABC transporter substrate-binding protein	BEK99_RS02125	SCA_RS09045	SE1039_RS09935	SEQMU2_RS01795	BK815_RS12560	A6V26_RS06785	AA913_RS00485	SXYL_RS03260	BE24_RS08545	SSP_RS03035
acyl-CoA dehydrogenase	BEK99_RS02130	SCA_RS09040	SE1039_RS09940	SEQMU2_RS01800	BK815_RS12550	A6V26_RS06795	AA913_RS00495	SXYL_RS03255	BE24_RS08550	SSP_RS03030
purine nucleosidase	BEK99_RS02135	SCA_RS09035	SE1039_RS11890	SEQMU2_RS03735	BK815_RS10760	A6V26_RS08605	AA913_RS10010	SXYL_RS01125	BE24_RS10575	SSP_RS01030
biotin transporter BioY	BEK99_RS02140	SCA_RS09030	SE1039_RS09930	SEQMU2_RS01790	BK815_RS12565	A6V26_RS06780	AA913_RS00480	SXYL_RS03265	BE24_RS08540	SSP_RS03040
N-acetyltransferase	BEK99_RS02145	SCA_RS09025	SE1039_RS09925	SEQMU2_RS01785	BK815_RS12570	A6V26_RS06775	AA913_RS00475	SXYL_RS03270	BE24_RS08535	SSP_RS03045
sufurtransferase FdhD	BEK99_RS02150	SCA_RS09020	SE1039_RS09920	SEQMU2_RS01780	BK815_RS12575	A6V26_RS06770	AA913_RS00470	SXYL_RS03275	BE24_RS08530	SSP_RS03050
LacI family transcriptional regulator	BEK99_RS02155	SCA_RS09015	SE1039_RS09915	SEQMU2_RS01775	BK815_RS12580	A6V26_RS06765	AA913_RS00465	SXYL_RS03280	BE24_RS08525	SSP_RS03055
galactokinase	BEK99_RS02160	SCA_RS09010	SE1039_RS09910	SEQMU2_RS01770	BK815_RS12585	A6V26_RS06760	AA913_RS00460	SXYL_RS03285	BE24_RS08520	SSP_RS03060
UDP-glucose 4-epimerase GalE	BEK99_RS02165	SCA_RS09005	SE1039_RS09905	SEQMU2_RS01765	BK815_RS12590	A6V26_RS06755	AA913_RS00455	SXYL_RS03290	BE24_RS08515	SSP_RS03065
UDP-glucose--hexose-1-phosphate uridylyltransferase	BEK99_RS02170	SCA_RS09000	SE1039_RS09900	SEQMU2_RS01760	BK815_RS12595	A6V26_RS06750	AA913_RS00450	SXYL_RS03295	BE24_RS08510	SSP_RS03070
molybdate ABC transporter permease subunit	BEK99_RS02180	SCA_RS08990	SE1039_RS09890	SEQMU2_RS01750	BK815_RS12605	A6V26_RS06740	AA913_RS00440	SXYL_RS03305	BE24_RS08500	SSP_RS03080
molybdenum ABC transporter ATP-binding protein	BEK99_RS02185	SCA_RS08985	SE1039_RS09885	SEQMU2_RS01745	BK815_RS12610	A6V26_RS06735	AA913_RS00435	SXYL_RS03310	BE24_RS08495	SSP_RS03085
molybdopterin biosynthesis protein MoeB	BEK99_RS02190	SCA_RS08980	SE1039_RS09875	SEQMU2_RS01735	BK815_RS12620	A6V26_RS06725	AA913_RS00425	SXYL_RS03320	BE24_RS08485	SSP_RS03100
molybdenum cofactor biosynthesis protein	BEK99_RS02195	SCA_RS08975	SE1039_RS09870	SEQMU2_RS01730	BK815_RS12625	A6V26_RS06720	AA913_RS00420	SXYL_RS03325	BE24_RS08480	SSP_RS03105
cyclic pyranopterin monophosphate synthase MoaC	BEK99_RS02200	SCA_RS08970	SE1039_RS09865	SEQMU2_RS01725	BK815_RS12630	A6V26_RS06715	AA913_RS00415	SXYL_RS03330	BE24_RS08475	SSP_RS03110
molybdopterin molybdenumtransferase MoeA	BEK99_RS02205	SCA_RS08965	SE1039_RS09860	SEQMU2_RS01720	BK815_RS12635	A6V26_RS06710	AA913_RS00410	SXYL_RS03335	BE24_RS08470	SSP_RS03115
molybdopterin-guanine dinucleotide biosynthesis protein B	BEK99_RS02210	SCA_RS08960	SE1039_RS09855	SEQMU2_RS01715	BK815_RS12640	A6V26_RS06705	AA913_RS00405	SXYL_RS03340	BE24_RS08465	SSP_RS03120
molybdopterin synthase subunit 2	BEK99_RS02215	SCA_RS08955	SE1039_RS09850	SEQMU2_RS01710	BK815_RS12645	A6V26_RS06700	AA913_RS00400	SXYL_RS03345	BE24_RS08460	SSP_RS03125

Product	*S. carnosus*		*S. equorum*		*S. succinus*			*S. xylosus*		*S. saprophyticus*
	**JCM 6069**	**TM300**	**KS1039**	**Mu2**	**14BME20**	**CSM 77**	**DSM 14617**	**C2a**	**HKUOPL8**	**ATCC 15305**
molybdopterin converting factor subunit 1	BEK99_RS02220	SCA_RS08950	SE1039_RS09845	SEQMU2_RS01705	BK815_RS12650	A6V26_RS06695	AA913_RS00395	SXYL_RS03350	BE24_RS08455	SSP_RS03130
molybdenum cofactor guanylyltransferase	BEK99_RS02225	SCA_RS08945	SE1039_RS09840	SEQMU2_RS01700	BK815_RS12655	A6V26_RS06690	AA913_RS00390	SXYL_RS03355	BE24_RS08450	SSP_RS03135
cyclic pyranopterin monophosphate synthase	BEK99_RS02230	SCA_RS08940	SE1039_RS09835	SEQMU2_RS01695	BK815_RS12660	A6V26_RS06685	AA913_RS00385	SXYL_RS03360	BE24_RS08445	SSP_RS03140
MarR family transcriptional regulator	BEK99_RS02235	SCA_RS08935	SE1039_RS09830	SEQMU2_RS01690	BK815_RS12665	A6V26_RS06680	AA913_RS00380	SXYL_RS03365	BE24_RS08440	SSP_RS03145
MFS transporter	BEK99_RS02240	SCA_RS08930	SE1039_RS09825	SEQMU2_RS01685	BK815_RS12670	A6V26_RS06675	AA913_RS00375	SXYL_RS03370	BE24_RS08435	SSP_RS03150
MarR family transcriptional regulator	BEK99_RS02245	SCA_RS08925	SE1039_RS09820	SEQMU2_RS01680	BK815_RS12675	A6V26_RS06670	AA913_RS00370	SXYL_RS03375	BE24_RS08430	SSP_RS03155
sulfurtransferase	BEK99_RS02250	SCA_RS08920	SE1039_RS09815	SEQMU2_RS01675	BK815_RS12680	A6V26_RS06665	AA913_RS00365	SXYL_RS03380	BE24_RS08425	SSP_RS03160
hypothetical protein	BEK99_RS02255	SCA_RS08915	SE1039_RS09810	SEQMU2_RS01670	BK815_RS12685	A6V26_RS13495	AA913_RS14220	SXYL_RS03385	BE24_RS08420	SSP_RS03165
N-acetyltransferase	BEK99_RS02260	SCA_RS08910	SE1039_RS09805	SEQMU2_RS01665	BK815_RS12690	A6V26_RS06660	AA913_RS00360	SXYL_RS03390	BE24_RS08415	SSP_RS03170
hypothetical protein	BEK99_RS02280	SCA_RS08890	SE1039_RS09795	SEQMU2_RS01655	BK815_RS12700	A6V26_RS06650	AA913_RS00350	SXYL_RS03400	BE24_RS08405	SSP_RS03180
malonate transporter	BEK99_RS02285	SCA_RS08885	SE1039_RS09790	SEQMU2_RS01650	BK815_RS12705	A6V26_RS06645	AA913_RS00345	SXYL_RS03405	BE24_RS08400	SSP_RS03185
sugar dehydrogenase	BEK99_RS02290	SCA_RS08880	SE1039_RS09785	SEQMU2_RS01645	BK815_RS12710	A6V26_RS06640	AA913_RS00340	SXYL_RS03410	BE24_RS08395	SSP_RS03190
glucose transporter	BEK99_RS02295	SCA_RS08875	SE1039_RS09780	SEQMU2_RS01640	BK815_RS12715	A6V26_RS06635	AA913_RS00335	SXYL_RS03415	BE24_RS08390	SSP_RS03195
N-acetyltransferase	BEK99_RS02300	SCA_RS08870	SE1039_RS09775	SEQMU2_RS01635	BK815_RS12720	A6V26_RS06630	AA913_RS00330	SXYL_RS03420	BE24_RS08385	SSP_RS03200
DNA topoisomerase III	BEK99_RS02305	SCA_RS08865	SE1039_RS09770	SEQMU2_RS01630	BK815_RS12725	A6V26_RS06625	AA913_RS00325	SXYL_RS03425	BE24_RS08380	SSP_RS03205
NCS2 family permease	BEK99_RS02310	SCA_RS08860	SE1039_RS09765	SEQMU2_RS01625	BK815_RS12730	A6V26_RS06620	AA913_RS00320	SXYL_RS03430	BE24_RS08375	SSP_RS03210
hypothetical protein	BEK99_RS02320	SCA_RS08850	SE1039_RS09760	SEQMU2_RS01620	BK815_RS12735	A6V26_RS06615	AA913_RS00315	SXYL_RS03435	BE24_RS08370	SSP_RS03215
30S ribosomal protein S10	BEK99_RS02325	SCA_RS08845	SE1039_RS09755	SEQMU2_RS01615	BK815_RS12740	A6V26_RS06610	AA913_RS00310	SXYL_RS03440	BE24_RS08365	SSP_RS03220
50S ribosomal protein L3	BEK99_RS02330	SCA_RS08840	SE1039_RS09750	SEQMU2_RS01610	BK815_RS12745	A6V26_RS06605	AA913_RS00305	SXYL_RS03445	BE24_RS08360	SSP_RS03225
50S ribosomal protein L4	BEK99_RS02335	SCA_RS08835	SE1039_RS09745	SEQMU2_RS01605	BK815_RS12750	A6V26_RS06600	AA913_RS00300	SXYL_RS03450	BE24_RS08355	SSP_RS03230
50S ribosomal protein L23	BEK99_RS02340	SCA_RS08830	SE1039_RS09740	SEQMU2_RS01600	BK815_RS12755	A6V26_RS06595	AA913_RS00295	SXYL_RS03455	BE24_RS08350	SSP_RS03235
50S ribosomal protein L2	BEK99_RS02345	SCA_RS08825	SE1039_RS09735	SEQMU2_RS01595	BK815_RS12760	A6V26_RS06590	AA913_RS00290	SXYL_RS03460	BE24_RS08345	SSP_RS03240
30S ribosomal protein S19	BEK99_RS02350	SCA_RS08820	SE1039_RS09730	SEQMU2_RS01590	BK815_RS12765	A6V26_RS06585	AA913_RS00285	SXYL_RS03465	BE24_RS08340	SSP_RS03245
50S ribosomal protein L22	BEK99_RS02355	SCA_RS08815	SE1039_RS09725	SEQMU2_RS01585	BK815_RS12770	A6V26_RS06580	AA913_RS00280	SXYL_RS03470	BE24_RS08335	SSP_RS03250
30S ribosomal protein S3	BEK99_RS02360	SCA_RS08810	SE1039_RS09720	SEQMU2_RS01580	BK815_RS12775	A6V26_RS06575	AA913_RS00275	SXYL_RS03475	BE24_RS08330	SSP_RS03255
50S ribosomal protein L16	BEK99_RS02365	SCA_RS08805	SE1039_RS09715	SEQMU2_RS01575	BK815_RS12780	A6V26_RS06570	AA913_RS00270	SXYL_RS03480	BE24_RS08325	SSP_RS03260
50S ribosomal protein L29	BEK99_RS02370	SCA_RS08800	SE1039_RS09710	SEQMU2_RS01570	BK815_RS12785	A6V26_RS06565	AA913_RS00265	SXYL_RS03485	BE24_RS08320	SSP_RS03265
30S ribosomal protein S17	BEK99_RS02375	SCA_RS08795	SE1039_RS09705	SEQMU2_RS01565	BK815_RS12790	A6V26_RS06560	AA913_RS00260	SXYL_RS03490	BE24_RS08315	SSP_RS03270
50S ribosomal protein L14	BEK99_RS02380	SCA_RS08790	SE1039_RS09700	SEQMU2_RS01560	BK815_RS12795	A6V26_RS06555	AA913_RS00255	SXYL_RS03495	BE24_RS08310	SSP_RS03275
50S ribosomal protein L24	BEK99_RS02385	SCA_RS08785	SE1039_RS09695	SEQMU2_RS01555	BK815_RS12800	A6V26_RS06550	AA913_RS00250	SXYL_RS03500	BE24_RS08305	SSP_RS03280
50S ribosomal protein L5	BEK99_RS02390	SCA_RS08780	SE1039_RS09690	SEQMU2_RS01550	BK815_RS12805	A6V26_RS06545	AA913_RS00245	SXYL_RS03505	BE24_RS08300	SSP_RS03285

Product	*S. carnosus*		*S. equorum*		*S. succinus*			*S. xylosus*		*S. saprophyticus*
	**JCM 6069**	**TM300**	**KS1039**	**Mu2**	**14BME20**	**CSM 77**	**DSM 14617**	**C2a**	**HKUOPL8**	**ATCC 15305**
30S ribosomal protein S14 type Z	BEK99_RS02395	SCA_RS08775	SE1039_RS09685	SEQMU2_RS01545	BK815_RS12810	A6V26_RS06540	AA913_RS00240	SXYL_RS03510	BE24_RS08295	SSP_RS03290
30S ribosomal protein S8	BEK99_RS02400	SCA_RS08770	SE1039_RS09680	SEQMU2_RS01540	BK815_RS12815	A6V26_RS06535	AA913_RS00235	SXYL_RS03515	BE24_RS08290	SSP_RS03295
50S ribosomal protein L6	BEK99_RS02405	SCA_RS08765	SE1039_RS09675	SEQMU2_RS01535	BK815_RS12820	A6V26_RS06530	AA913_RS00230	SXYL_RS03520	BE24_RS08285	SSP_RS03300
50S ribosomal protein L18	BEK99_RS02410	SCA_RS08760	SE1039_RS09670	SEQMU2_RS01530	BK815_RS12825	A6V26_RS06525	AA913_RS00225	SXYL_RS03525	BE24_RS08280	SSP_RS03305
30S ribosomal protein S5	BEK99_RS02415	SCA_RS08755	SE1039_RS09665	SEQMU2_RS01525	BK815_RS12830	A6V26_RS06520	AA913_RS00220	SXYL_RS03530	BE24_RS08275	SSP_RS03310
preprotein translocase subunit SecY	BEK99_RS02430	SCA_RS08740	SE1039_RS09650	SEQMU2_RS01510	BK815_RS12845	A6V26_RS06505	AA913_RS00205	SXYL_RS03545	BE24_RS08260	SSP_RS03325
adenylate kinase	BEK99_RS02435	SCA_RS08735	SE1039_RS09645	SEQMU2_RS01505	BK815_RS12850	A6V26_RS06500	AA913_RS00200	SXYL_RS03550	BE24_RS08255	SSP_RS03330
translation initiation factor IF-1	BEK99_RS02440	SCA_RS08730	SE1039_RS09640	SEQMU2_RS01500	BK815_RS12855	A6V26_RS06495	AA913_RS00195	SXYL_RS03555	BE24_RS08250	SSP_RS03335
50S ribosomal protein L36	BEK99_RS02445	SCA_RS08725	SE1039_RS09635	SEQMU2_RS01495	BK815_RS12860	A6V26_RS06490	AA913_RS00190	SXYL_RS03560	BE24_RS08245	SSP_RS03340
30S ribosomal protein S13	BEK99_RS02450	SCA_RS08720	SE1039_RS09630	SEQMU2_RS01490	BK815_RS12865	A6V26_RS06485	AA913_RS00185	SXYL_RS03565	BE24_RS08240	SSP_RS03345
30S ribosomal protein S11	BEK99_RS02455	SCA_RS08715	SE1039_RS09625	SEQMU2_RS01485	BK815_RS12870	A6V26_RS06480	AA913_RS00180	SXYL_RS03570	BE24_RS08235	SSP_RS03350
DNA-directed RNA polymerase subunit alpha	BEK99_RS02460	SCA_RS08710	SE1039_RS09620	SEQMU2_RS01480	BK815_RS12875	A6V26_RS06475	AA913_RS00175	SXYL_RS03575	BE24_RS08230	SSP_RS03355
50S ribosomal protein L17	BEK99_RS02465	SCA_RS08705	SE1039_RS09615	SEQMU2_RS01475	BK815_RS12880	A6V26_RS06470	AA913_RS00170	SXYL_RS03580	BE24_RS08225	SSP_RS03360
energy-coupling factor transporter ATPase	BEK99_RS02470	SCA_RS08700	SE1039_RS09610	SEQMU2_RS01470	BK815_RS12885	A6V26_RS06465	AA913_RS00165	SXYL_RS03585	BE24_RS08220	SSP_RS03365
energy-coupling factor transporter ATPase	BEK99_RS02475	SCA_RS08695	SE1039_RS09605	SEQMU2_RS01465	BK815_RS12890	A6V26_RS06460	AA913_RS00160	SXYL_RS03590	BE24_RS08215	SSP_RS03370
energy-coupling factor transporter transmembrane protein EcfT	BEK99_RS02480	SCA_RS08690	SE1039_RS09600	SEQMU2_RS01460	BK815_RS12895	A6V26_RS06455	AA913_RS00155	SXYL_RS03595	BE24_RS08210	SSP_RS03375
tRNA pseudouridine synthase A	BEK99_RS02485	SCA_RS08685	SE1039_RS09595	SEQMU2_RS01455	BK815_RS12900	A6V26_RS06450	AA913_RS00150	SXYL_RS03600	BE24_RS08205	SSP_RS03380
50S ribosomal protein L13	BEK99_RS02490	SCA_RS08680	SE1039_RS09590	SEQMU2_RS01450	BK815_RS12905	A6V26_RS06445	AA913_RS00145	SXYL_RS03605	BE24_RS08200	SSP_RS03385
30S ribosomal protein S9	BEK99_RS02495	SCA_RS08675	SE1039_RS09585	SEQMU2_RS01445	BK815_RS12910	A6V26_RS06440	AA913_RS00140	SXYL_RS03610	BE24_RS08195	SSP_RS03390
kinase/pyrophosphorylase	BEK99_RS02540	SCA_RS08630	SE1039_RS12030	SEQMU2_RS03875	BK815_RS10625	A6V26_RS08740	AA913_RS09875	SXYL_RS01010	BE24_RS10700	SSP_RS00895
alpha/beta hydrolase	BEK99_RS02550	SCA_RS08620	SE1039_RS09580	SEQMU2_RS01440	BK815_RS12915	A6V26_RS06435	AA913_RS00135	SXYL_RS03615	BE24_RS11335	SSP_RS00470
zinc-binding alcohol dehydrogenase family protein	BEK99_RS02560	SCA_RS08610	SE1039_RS09565	SEQMU2_RS01425	BK815_RS12930	A6V26_RS06420	AA913_RS00120	SXYL_RS03630	BE24_RS08175	SSP_RS03410
hypothetical protein	BEK99_RS02570	SCA_RS08600	SE1039_RS09555	SEQMU2_RS01415	BK815_RS12940	A6V26_RS06410	AA913_RS00110	SXYL_RS03640	BE24_RS08165	SSP_RS03420
membrane protein	BEK99_RS02575	SCA_RS08595	SE1039_RS09550	SEQMU2_RS01410	BK815_RS12945	A6V26_RS06405	AA913_RS00105	SXYL_RS03645	BE24_RS08160	SSP_RS03425
Asp23/Gls24 family envelope stress response protein	BEK99_RS02580	SCA_RS08590	SE1039_RS09545	SEQMU2_RS01405	BK815_RS12950	A6V26_RS06400	AA913_RS00100	SXYL_RS03650	BE24_RS08155	SSP_RS03430
sialic acid synthase	BEK99_RS02585	SCA_RS08585	SE1039_RS09540	SEQMU2_RS01400	BK815_RS12955	A6V26_RS06395	AA913_RS00095	SXYL_RS03655	BE24_RS08150	SSP_RS03435
siderophore synthetase	BEK99_RS02595	SCA_RS08575	SE1039_RS09535	SEQMU2_RS01395	BK815_RS12965	A6V26_RS06385	AA913_RS00085	SXYL_RS03665	BE24_RS08140	SSP_RS03445
alanine racemase	BEK99_RS02600	SCA_RS08570	SE1039_RS09530	SEQMU2_RS01390	BK815_RS12970	A6V26_RS06380	AA913_RS00080	SXYL_RS03670	BE24_RS08135	SSP_RS03450
iron citrate ABC transporter substrate-binding protein	BEK99_RS02605	SCA_RS08565	SE1039_RS09525	SEQMU2_RS01385	BK815_RS12975	A6V26_RS06375	AA913_RS00075	SXYL_RS03675	BE24_RS08130	SSP_RS03455
iron ABC transporter permease	BEK99_RS02610	SCA_RS08560	SE1039_RS09520	SEQMU2_RS01380	BK815_RS12980	A6V26_RS06370	AA913_RS00070	SXYL_RS03680	BE24_RS08125	SSP_RS03460
iron-dicitrate transporter subunit FecD	BEK99_RS02615	SCA_RS08555	SE1039_RS09515	SEQMU2_RS01375	BK815_RS12985	A6V26_RS06365	AA913_RS00065	SXYL_RS03685	BE24_RS08120	SSP_RS03465

Product	*S. carnosus*		*S. equorum*		*S. succinus*			*S. xylosus*		*S. saprophyticus*
	**JCM 6069**	**TM300**	**KS1039**	**Mu2**	**14BME20**	**CSM 77**	**DSM 14617**	**C2a**	**HKUOPL8**	**ATCC 15305**
hypothetical protein	BEK99_RS02620	SCA_RS08550	SE1039_RS09510	SEQMU2_RS01370	BK815_RS12990	A6V26_RS06360	AA913_RS00060	SXYL_RS03690	BE24_RS08115	SSP_RS03470
heme-degrading monooxygenase IsdG	BEK99_RS02625	SCA_RS08545	SE1039_RS09505	SEQMU2_RS01365	BK815_RS12995	A6V26_RS06355	AA913_RS00055	SXYL_RS03695	BE24_RS08110	SSP_RS03475
hypothetical protein	BEK99_RS02635	SCA_RS08535	SE1039_RS09500	SEQMU2_RS01360	BK815_RS13010	A6V26_RS06340	AA913_RS00040	SXYL_RS03700	BE24_RS08105	SSP_RS03480
metal-dependent hydrolase	BEK99_RS02640	SCA_RS08530	SE1039_RS09495	SEQMU2_RS01355	BK815_RS13015	A6V26_RS06335	AA913_RS00035	SXYL_RS03705	BE24_RS08100	SSP_RS03485
uridylyltransferase	BEK99_RS02645	SCA_RS08525	SE1039_RS09490	SEQMU2_RS01350	BK815_RS13020	A6V26_RS06330	AA913_RS00030	SXYL_RS03710	BE24_RS08095	SSP_RS03490
MFS transporter	BEK99_RS02655	SCA_RS08515	SE1039_RS09480	SEQMU2_RS01340	BK815_RS13030	A6V26_RS06320	AA913_RS00020	SXYL_RS03720	BE24_RS08085	SSP_RS03500
multidrug resistance protein SepA	BEK99_RS02660	SCA_RS08510	SE1039_RS09475	SEQMU2_RS01335	BK815_RS13035	A6V26_RS06315	AA913_RS00015	SXYL_RS03725	BE24_RS08080	SSP_RS03505
ABC transporter permease	BEK99_RS02720	SCA_RS08445	SE1039_RS09415	SEQMU2_RS01290	BK815_RS13100	A6V26_RS12125	AA913_RS11705	SXYL_RS03785	BE24_RS08010	SSP_RS03565
hypothetical protein	BEK99_RS02725	SCA_RS08440	SE1039_RS09410	SEQMU2_RS01285	BK815_RS13105	A6V26_RS12120	AA913_RS11700	SXYL_RS03790	BE24_RS08005	SSP_RS03570
phosphoglucosamine mutase	BEK99_RS02730	SCA_RS08435	SE1039_RS09405	SEQMU2_RS01280	BK815_RS13110	A6V26_RS12115	AA913_RS11695	SXYL_RS03795	BE24_RS08000	SSP_RS03575
PTS mannitol transporter subunit IIA	BEK99_RS02745	SCA_RS08420	SE1039_RS09395	SEQMU2_RS01270	BK815_RS00010	A6V26_RS12105	AA913_RS11685	SXYL_RS03805	BE24_RS07990	SSP_RS03585
PTS lactose transporter subunit IIB	BEK99_RS02750	SCA_RS08415	SE1039_RS09390	SEQMU2_RS01265	BK815_RS00015	A6V26_RS12100	AA913_RS11680	SXYL_RS03810	BE24_RS07985	SSP_RS03590
glutamine--fructose-6-phosphate aminotransferase	BEK99_RS02760	SCA_RS08405	SE1039_RS09380	SEQMU2_RS01255	BK815_RS00025	A6V26_RS12090	AA913_RS11670	SXYL_RS03820	BE24_RS07975	SSP_RS03600
haloacid dehalogenase	BEK99_RS02770	SCA_RS08395	SE1039_RS09360	SEQMU2_RS01235	BK815_RS00040	A6V26_RS12075	AA913_RS11655	SXYL_RS03835	BE24_RS07935	SSP_RS03615
phosphodiesterase	BEK99_RS02780	SCA_RS08385	SE1039_RS09350	SEQMU2_RS01225	BK815_RS00050	A6V26_RS12065	AA913_RS11645	SXYL_RS03845	BE24_RS07915	SSP_RS03625
cation transporter	BEK99_RS02785	SCA_RS08380	SE1039_RS09345	SEQMU2_RS01220	BK815_RS00055	A6V26_RS12060	AA913_RS11640	SXYL_RS03850	BE24_RS07910	SSP_RS03630
transcriptional regulator	BEK99_RS02790	SCA_RS08375	SE1039_RS09340	SEQMU2_RS01215	BK815_RS00060	A6V26_RS12055	AA913_RS11635	SXYL_RS03855	BE24_RS07905	SSP_RS03635
FadR family transcriptional regulator	BEK99_RS02800	SCA_RS08365	SE1039_RS09330	SEQMU2_RS01205	BK815_RS00070	A6V26_RS12045	AA913_RS11625	SXYL_RS03865	BE24_RS07895	SSP_RS03645
mannose-6-phosphate isomerase	BEK99_RS02805	SCA_RS08360	SE1039_RS09325	SEQMU2_RS01200	BK815_RS00075	A6V26_RS12035	AA913_RS11615	SXYL_RS03870	BE24_RS07890	SSP_RS03650
hypothetical protein	BEK99_RS02810	SCA_RS08355	SE1039_RS09320	SEQMU2_RS01195	BK815_RS00080	A6V26_RS12030	AA913_RS11610	SXYL_RS03875	BE24_RS07885	SSP_RS03655
hypothetical protein	BEK99_RS02815	SCA_RS08350	SE1039_RS09310	SEQMU2_RS01185	BK815_RS00090	A6V26_RS12020	AA913_RS11600	SXYL_RS03885	BE24_RS07875	SSP_RS03665
hypothetical protein	BEK99_RS02820	SCA_RS08345	SE1039_RS09305	SEQMU2_RS01180	BK815_RS00095	A6V26_RS12015	AA913_RS11595	SXYL_RS03890	BE24_RS07870	SSP_RS03670
DNA starvation/stationary phase protection protein	BEK99_RS02825	SCA_RS08340	SE1039_RS09295	SEQMU2_RS01170	BK815_RS00105	A6V26_RS12005	AA913_RS11585	SXYL_RS03900	BE24_RS07860	SSP_RS03680
hypothetical protein	BEK99_RS02830	SCA_RS08335	SE1039_RS08610	SEQMU2_RS00485	BK815_RS00800	A6V26_RS12410	AA913_RS11950	SXYL_RS04570	BE24_RS07165	SSP_RS04385
purine-nucleoside phosphorylase	BEK99_RS02835	SCA_RS08330	SE1039_RS09290	SEQMU2_RS01165	BK815_RS00110	A6V26_RS12000	AA913_RS11580	SXYL_RS03905	BE24_RS07855	SSP_RS03685
2-deoxyribose-5-phosphate aldolase	BEK99_RS02840	SCA_RS08325	SE1039_RS09285	SEQMU2_RS01160	BK815_RS00115	A6V26_RS11995	AA913_RS11575	SXYL_RS03910	BE24_RS07850	SSP_RS03690
S-ribosylhomocysteine lyase	BEK99_RS02850	SCA_RS08315	SE1039_RS09265	SEQMU2_RS01140	BK815_RS00135	A6V26_RS11975	AA913_RS11555	SXYL_RS03930	BE24_RS07830	SSP_RS03710
amidohydrolase	BEK99_RS02855	SCA_RS08310	SE1039_RS09260	SEQMU2_RS01135	BK815_RS00140	A6V26_RS11970	AA913_RS11550	SXYL_RS03935	BE24_RS07825	SSP_RS03715
DUF2750 domain-containing protein	BEK99_RS02860	SCA_RS08305	SE1039_RS09250	SEQMU2_RS01125	BK815_RS00150	A6V26_RS11960	AA913_RS11540	SXYL_RS03945	BE24_RS07815	SSP_RS03725
type II pantothenate kinase	BEK99_RS02865	SCA_RS08300	SE1039_RS09245	SEQMU2_RS01120	BK815_RS00155	A6V26_RS11955	AA913_RS11535	SXYL_RS03950	BE24_RS07810	SSP_RS03730
N-acetyltransferase	BEK99_RS02875	SCA_RS08290	SE1039_RS09235	SEQMU2_RS01110	BK815_RS00160	A6V26_RS11950	AA913_RS11530	SXYL_RS03955	BE24_RS07805	SSP_RS03735

Product	*S. carnosus*		*S. equorum*		*S. succinus*			*S. xylosus*		*S. saprophyticus*
	**JCM 6069**	**TM300**	**KS1039**	**Mu2**	**14BME20**	**CSM 77**	**DSM 14617**	**C2a**	**HKUOPL8**	**ATCC 15305**
CTP synthase	BEK99_RS02885	SCA_RS08280	SE1039_RS09225	SEQMU2_RS01100	BK815_RS00170	A6V26_RS11940	AA913_RS11520	SXYL_RS03965	BE24_RS07795	SSP_RS03745
fructose-bisphosphate aldolase	BEK99_RS02895	SCA_RS08270	SE1039_RS09220	SEQMU2_RS01095	BK815_RS00175	A6V26_RS11935	AA913_RS11515	SXYL_RS03970	BE24_RS07790	SSP_RS03750
UDP-N-acetylglucosamine 1-carboxyvinyltransferase	BEK99_RS02900	SCA_RS08265	SE1039_RS09215	SEQMU2_RS01090	BK815_RS00180	A6V26_RS11930	AA913_RS11510	SXYL_RS03975	BE24_RS07785	SSP_RS03755
transcriptional regulator	BEK99_RS02905	SCA_RS08260	SE1039_RS09210	SEQMU2_RS01085	BK815_RS00185	A6V26_RS11925	AA913_RS11505	SXYL_RS03980	BE24_RS07780	SSP_RS03760
aldehyde dehydrogenase family protein	BEK99_RS02910	SCA_RS08255	SE1039_RS09205	SEQMU2_RS01080	BK815_RS00190	A6V26_RS11920	AA913_RS11500	SXYL_RS03985	BE24_RS07775	SSP_RS03765
transcription termination factor Rho	BEK99_RS02915	SCA_RS08250	SE1039_RS09200	SEQMU2_RS01075	BK815_RS00195	A6V26_RS11915	AA913_RS11495	SXYL_RS03990	BE24_RS07770	SSP_RS03770
50S ribosomal protein L31 type B	BEK99_RS02920	SCA_RS08245	SE1039_RS09195	SEQMU2_RS01070	BK815_RS00200	A6V26_RS11910	AA913_RS11490	SXYL_RS03995	BE24_RS07765	SSP_RS03775
thymidine kinase	BEK99_RS02925	SCA_RS08240	SE1039_RS09190	SEQMU2_RS01065	BK815_RS00205	A6V26_RS11905	AA913_RS11485	SXYL_RS04000	BE24_RS07760	SSP_RS03780
peptide chain release factor 1	BEK99_RS02930	SCA_RS08235	SE1039_RS09185	SEQMU2_RS01060	BK815_RS00210	A6V26_RS11900	AA913_RS11480	SXYL_RS04005	BE24_RS07755	SSP_RS03785
protein-(glutamine-N5) methyltransferase release factor-specific	BEK99_RS02935	SCA_RS08230	SE1039_RS09180	SEQMU2_RS01055	BK815_RS00215	A6V26_RS11895	AA913_RS11475	SXYL_RS04010	BE24_RS07750	SSP_RS03790
threonylcarbamoyl-AMP synthase	BEK99_RS02940	SCA_RS08225	SE1039_RS09175	SEQMU2_RS01050	BK815_RS00220	A6V26_RS11890	AA913_RS11470	SXYL_RS04015	BE24_RS07745	SSP_RS03795
low molecular weight protein arginine phosphatase	BEK99_RS02945	SCA_RS08220	SE1039_RS09170	SEQMU2_RS01045	BK815_RS00225	A6V26_RS11885	AA913_RS11465	SXYL_RS04020	BE24_RS07740	SSP_RS03800
TIGR01440 family protein	BEK99_RS02950	SCA_RS08215	SE1039_RS09165	SEQMU2_RS01040	BK815_RS00230	A6V26_RS11880	AA913_RS11460	SXYL_RS04025	BE24_RS07735	SSP_RS03805
serine hydroxymethyltransferase	BEK99_RS02955	SCA_RS08210	SE1039_RS09160	SEQMU2_RS01035	BK815_RS00235	A6V26_RS11875	AA913_RS11455	SXYL_RS04030	BE24_RS07730	SSP_RS03810
uracil phosphoribosyltransferase	BEK99_RS02960	SCA_RS08205	SE1039_RS09155	SEQMU2_RS01030	BK815_RS00240	A6V26_RS11870	AA913_RS11450	SXYL_RS04035	BE24_RS07725	SSP_RS03815
UDP-N-acetylglucosamine 2-epimerase (non-hydrolyzing)	BEK99_RS02965	SCA_RS08200	SE1039_RS09150	SEQMU2_RS01025	BK815_RS00245	A6V26_RS11865	AA913_RS11445	SXYL_RS04040	BE24_RS07720	SSP_RS03820
hypothetical protein	BEK99_RS02970	SCA_RS08195	SE1039_RS09145	SEQMU2_RS01020	BK815_RS00250	A6V26_RS11860	AA913_RS11440	SXYL_RS13265	BE24_RS07715	SSP_RS03825
ATP synthase subunit A	BEK99_RS02975	SCA_RS08190	SE1039_RS09140	SEQMU2_RS01015	BK815_RS00255	A6V26_RS11855	AA913_RS11435	SXYL_RS04045	BE24_RS07710	SSP_RS03830
ATP synthase subunit C	BEK99_RS02980	SCA_RS08185	SE1039_RS09135	SEQMU2_RS01010	BK815_RS00260	A6V26_RS11850	AA913_RS11430	SXYL_RS04050	BE24_RS07705	SSP_RS03835
ATP synthase F0 subunit B	BEK99_RS02985	SCA_RS08180	SE1039_RS09130	SEQMU2_RS01005	BK815_RS00265	A6V26_RS11845	AA913_RS11425	SXYL_RS04055	BE24_RS07700	SSP_RS03840
F0F1 ATP synthase subunit gamma	BEK99_RS03000	SCA_RS08165	SE1039_RS09115	SEQMU2_RS00990	BK815_RS00280	A6V26_RS11830	AA913_RS11410	SXYL_RS04070	BE24_RS07685	SSP_RS03855
F0F1 ATP synthase subunit beta	BEK99_RS03005	SCA_RS08160	SE1039_RS09110	SEQMU2_RS00985	BK815_RS00285	A6V26_RS11825	AA913_RS11405	SXYL_RS04075	BE24_RS07680	SSP_RS03860
ATP synthase epsilon chain	BEK99_RS03010	SCA_RS08155	SE1039_RS09105	SEQMU2_RS00980	BK815_RS00290	A6V26_RS11820	AA913_RS11400	SXYL_RS04080	BE24_RS07675	SSP_RS03865
membrane protein	BEK99_RS03015	SCA_RS08150	SE1039_RS09100	SEQMU2_RS00975	BK815_RS00295	A6V26_RS11815	AA913_RS11395	SXYL_RS04085	BE24_RS07670	SSP_RS03870
UDP-N-acetylglucosamine 1-carboxyvinyltransferase	BEK99_RS03020	SCA_RS08145	SE1039_RS09095	SEQMU2_RS00970	BK815_RS00300	A6V26_RS11810	AA913_RS11390	SXYL_RS04090	BE24_RS07665	SSP_RS03875
beta-hydroxyacyl-ACP dehydratase	BEK99_RS03030	SCA_RS08135	SE1039_RS09090	SEQMU2_RS00965	BK815_RS00305	A6V26_RS11805	AA913_RS11385	SXYL_RS04095	BE24_RS07660	SSP_RS03880
hypothetical protein	BEK99_RS03035	SCA_RS08130	SE1039_RS09085	SEQMU2_RS00960	BK815_RS00310	A6V26_RS11800	AA913_RS11380	SXYL_RS04100	BE24_RS07655	SSP_RS03885
single-stranded DNA-binding protein	BEK99_RS03040	SCA_RS08125	SE1039_RS09080	SEQMU2_RS00955	BK815_RS00315	A6V26_RS11795	AA913_RS11375	SXYL_RS04105	BE24_RS07650	SSP_RS03890
transglycosylase	BEK99_RS03045	SCA_RS08120	SE1039_RS09075	SEQMU2_RS04330	BK815_RS00320	A6V26_RS11790	AA913_RS11370	SXYL_RS04110	BE24_RS07645	SSP_RS03895
thiaminase II	BEK99_RS03060	SCA_RS08105	SE1039_RS09070	SEQMU2_RS00945	BK815_RS00325	A6V26_RS11785	AA913_RS11365	SXYL_RS04115	BE24_RS07640	SSP_RS03900
bifunctional hydroxymethylpyrimidine kinase/phosphomethylpyrimidine kinase	BEK99_RS03065	SCA_RS08100	SE1039_RS09065	SEQMU2_RS00940	BK815_RS00330	A6V26_RS11780	AA913_RS11360	SXYL_RS04120	BE24_RS07635	SSP_RS03905

Product	*S. carnosus*		*S. equorum*		*S. succinus*			*S. xylosus*		*S. saprophyticus*
	**JCM 6069**	**TM300**	**KS1039**	**Mu2**	**14BME20**	**CSM 77**	**DSM 14617**	**C2a**	**HKUOPL8**	**ATCC 15305**
hydroxyethylthiazole kinase	BEK99_RS03070	SCA_RS08095	SE1039_RS09060	SEQMU2_RS00935	BK815_RS00335	A6V26_RS11775	AA913_RS11355	SXYL_RS04125	BE24_RS07630	SSP_RS03910
thiamine phosphate synthase	BEK99_RS03075	SCA_RS08090	SE1039_RS09055	SEQMU2_RS00930	BK815_RS00340	A6V26_RS11770	AA913_RS11350	SXYL_RS04130	BE24_RS07625	SSP_RS03915
hypothetical protein	BEK99_RS03080	SCA_RS08085	SE1039_RS09050	SEQMU2_RS00925	BK815_RS00345	A6V26_RS11765	AA913_RS11345	SXYL_RS04135	BE24_RS07620	SSP_RS03920
phosphohydrolase	BEK99_RS03090	SCA_RS08075	SE1039_RS09045	SEQMU2_RS00920	BK815_RS00350	A6V26_RS11760	AA913_RS11340	SXYL_RS04140	BE24_RS07615	SSP_RS03925
CsoR family transcriptional regulator	BEK99_RS03100	SCA_RS08065	SE1039_RS09035	SEQMU2_RS00910	BK815_RS00360	A6V26_RS11750	AA913_RS11330	SXYL_RS04150	BE24_RS07605	SSP_RS03935
hypothetical protein	BEK99_RS03105	SCA_RS08060	SE1039_RS09030	SEQMU2_RS00905	BK815_RS00365	A6V26_RS11745	AA913_RS11325	SXYL_RS04155	BE24_RS07600	SSP_RS03940
copper chaperone	BEK99_RS03110	SCA_RS08055	SE1039_RS11425	SEQMU2_RS03280	BK815_RS11145	A6V26_RS08220	AA913_RS04000	SXYL_RS01600	BE24_RS10145	SSP_RS01440
copper-translocating P-type ATPase	BEK99_RS03115	SCA_RS08050	SE1039_RS11420	SEQMU2_RS03275	BK815_RS11150	A6V26_RS08215	AA913_RS03995	SXYL_RS01605	BE24_RS10140	SSP_RS01445
cell division protein FtsW	BEK99_RS03120	SCA_RS08045	SE1039_RS09025	SEQMU2_RS00900	BK815_RS00370	A6V26_RS11740	AA913_RS11320	SXYL_RS04160	BE24_RS07595	SSP_RS03945
D-alanine--D-alanine ligase A	BEK99_RS03125	SCA_RS08040	SE1039_RS09020	SEQMU2_RS00895	BK815_RS00375	A6V26_RS11735	AA913_RS11315	SXYL_RS04165	BE24_RS07590	SSP_RS03950
UDP-N-acetylmuramoyl-tripeptide--D-alanyl-D- alanine ligase	BEK99_RS03130	SCA_RS08035	SE1039_RS09015	SEQMU2_RS00890	BK815_RS00380	A6V26_RS11730	AA913_RS11310	SXYL_RS04170	BE24_RS07585	SSP_RS03955
ATP-dependent helicase	BEK99_RS03135	SCA_RS08030	SE1039_RS09010	SEQMU2_RS00885	BK815_RS00385	A6V26_RS11725	AA913_RS11305	SXYL_RS04175	BE24_RS07580	SSP_RS03960
hypothetical protein	BEK99_RS03140	SCA_RS08025	SE1039_RS09005	SEQMU2_RS00880	BK815_RS00390	A6V26_RS11720	AA913_RS11300	SXYL_RS04180	BE24_RS07575	SSP_RS03965
hypothetical protein	BEK99_RS03145	SCA_RS08020	SE1039_RS09000	SEQMU2_RS00875	BK815_RS00395	A6V26_RS11715	AA913_RS11295	SXYL_RS04185	BE24_RS07570	SSP_RS03970
hypothetical protein	BEK99_RS03150	SCA_RS08015	SE1039_RS08995	SEQMU2_RS00870	BK815_RS00400	A6V26_RS11710	AA913_RS11290	SXYL_RS04190	BE24_RS07565	SSP_RS03975
holo-ACP synthase	BEK99_RS03155	SCA_RS08010	SE1039_RS08990	SEQMU2_RS00865	BK815_RS00405	A6V26_RS11705	AA913_RS11285	SXYL_RS04195	BE24_RS07560	SSP_RS03980
alanine racemase	BEK99_RS03160	SCA_RS08005	SE1039_RS08985	SEQMU2_RS00860	BK815_RS00410	A6V26_RS11700	AA913_RS11280	SXYL_RS04200	BE24_RS07555	SSP_RS03985
hypothetical protein	BEK99_RS03165	SCA_RS08000	SE1039_RS08980	SEQMU2_RS00855	BK815_RS00415	A6V26_RS11695	AA913_RS11275	SXYL_RS04205	BE24_RS07550	SSP_RS03990
MazF/PemK family toxin	BEK99_RS03170	SCA_RS07995	SE1039_RS08975	SEQMU2_RS00850	BK815_RS00420	A6V26_RS11690	AA913_RS11270	SXYL_RS04210	BE24_RS07545	SSP_RS03995
serine phosphatase	BEK99_RS03175	SCA_RS07990	SE1039_RS08970	SEQMU2_RS00845	BK815_RS00425	A6V26_RS11685	AA913_RS11265	SXYL_RS04215	BE24_RS07540	SSP_RS04000
anti-sigma B factor antagonist	BEK99_RS03180	SCA_RS07985	SE1039_RS08965	SEQMU2_RS00840	BK815_RS00430	A6V26_RS11680	AA913_RS11260	SXYL_RS04220	BE24_RS07535	SSP_RS04005
anti-sigma B factor RsbW	BEK99_RS03185	SCA_RS07980	SE1039_RS08960	SEQMU2_RS00835	BK815_RS00435	A6V26_RS11675	AA913_RS11255	SXYL_RS04225	BE24_RS07530	SSP_RS04010
3-isopropylmalate dehydratase small subunit	BEK99_RS03230	SCA_RS07935	SE1039_RS08910	SEQMU2_RS00800	BK815_RS00485	A6V26_RS12730	AA913_RS13365	SXYL_RS04275	BE24_RS07465	SSP_RS04070
3-isopropylmalate dehydratase large subunit	BEK99_RS03235	SCA_RS07930	SE1039_RS08905	SEQMU2_RS00795	BK815_RS00490	A6V26_RS12725	AA913_RS13360	SXYL_RS04280	BE24_RS07460	SSP_RS04075
3-isopropylmalate dehydrogenase	BEK99_RS03240	SCA_RS07925	SE1039_RS08900	SEQMU2_RS00790	BK815_RS00495	A6V26_RS12720	AA913_RS13355	SXYL_RS04285	BE24_RS07455	SSP_RS04080
2-isopropylmalate synthase	BEK99_RS03245	SCA_RS07920	SE1039_RS08895	SEQMU2_RS00785	BK815_RS00500	A6V26_RS12715	AA913_RS13350	SXYL_RS04290	BE24_RS07450	SSP_RS04085
ketol-acid reductoisomerase	BEK99_RS03250	SCA_RS07915	SE1039_RS08890	SEQMU2_RS00780	BK815_RS00505	A6V26_RS12710	AA913_RS13345	SXYL_RS04295	BE24_RS07445	SSP_RS04090
acetolactate synthase small subunit	BEK99_RS03255	SCA_RS07910	SE1039_RS08885	SEQMU2_RS00775	BK815_RS00510	A6V26_RS12705	AA913_RS13340	SXYL_RS04300	BE24_RS07440	SSP_RS04095
acetolactate synthase large subunit biosynthetic type	BEK99_RS03260	SCA_RS07905	SE1039_RS08880	SEQMU2_RS00770	BK815_RS00515	A6V26_RS12700	AA913_RS13335	SXYL_RS04305	BE24_RS07435	SSP_RS04100
dihydroxy-acid dehydratase	BEK99_RS03265	SCA_RS07900	SE1039_RS08875	SEQMU2_RS00765	BK815_RS00520	A6V26_RS12695	AA913_RS13330	SXYL_RS04310	BE24_RS07430	SSP_RS04105
tRNA (adenosine(37)-N6)-threonylcarbamoyltransferase complex ATPase subunit type 1 TsaE	BEK99_RS03270	SCA_RS07895	SE1039_RS08870	SEQMU2_RS00760	BK815_RS00525	A6V26_RS12690	AA913_RS13155	SXYL_RS04315	BE24_RS07425	SSP_RS04110

Product	*S. carnosus*		*S. equorum*		*S. succinus*			*S. xylosus*		*S. saprophyticus*
	**JCM 6069**	**TM300**	**KS1039**	**Mu2**	**14BME20**	**CSM 77**	**DSM 14617**	**C2a**	**HKUOPL8**	**ATCC 15305**
tRNA (adenosine(37)-N6)-threonylcarbamoyltransferase complex dimerization subunit type 1 TsaB	BEK99_RS03275	SCA_RS07890	SE1039_RS08865	SEQMU2_RS00755	BK815_RS00530	A6V26_RS12685	AA913_RS13160	SXYL_RS04320	BE24_RS07420	SSP_RS04115
ribosomal-protein-alanine N-acetyltransferase RimI	BEK99_RS03280	SCA_RS07885	SE1039_RS08860	SEQMU2_RS00750	BK815_RS00535	A6V26_RS12680	AA913_RS13165	SXYL_RS04325	BE24_RS07415	SSP_RS04120
tRNA (adenosine(37)-N6)-threonylcarbamoyltransferase complex transferase subunit TsaD	BEK99_RS03285	SCA_RS07880	SE1039_RS08855	SEQMU2_RS00745	BK815_RS00540	A6V26_RS12675	AA913_RS13170	SXYL_RS04330	BE24_RS07410	SSP_RS04125
ABC transporter ATP-binding protein	BEK99_RS03290	SCA_RS07875	SE1039_RS08840	SEQMU2_RS00730	BK815_RS00555	A6V26_RS12660	AA913_RS13185	SXYL_RS04345	BE24_RS07390	SSP_RS04140
oxidoreductase	BEK99_RS03300	SCA_RS07865	SE1039_RS08825	SEQMU2_RS00715	BK815_RS00570	A6V26_RS12645	AA913_RS13200	SXYL_RS04360	BE24_RS07375	SSP_RS04155
YeeE/YedE family protein	BEK99_RS03305	SCA_RS07860	SE1039_RS08830	SEQMU2_RS00720	BK815_RS00565	A6V26_RS12650	AA913_RS13195	SXYL_RS04355	BE24_RS07380	SSP_RS04150
sucrose-6-phosphate hydrolase	BEK99_RS03310	SCA_RS07855	SE1039_RS08815	SEQMU2_RS00705	BK815_RS00580	A6V26_RS12635	AA913_RS13210	SXYL_RS04370	BE24_RS07365	SSP_RS04165
DNA-binding response regulator	BEK99_RS03315	SCA_RS07850	SE1039_RS08805	SEQMU2_RS00695	BK815_RS00590	A6V26_RS12625	AA913_RS13220	SXYL_RS04380	BE24_RS07355	SSP_RS04175
cyclic lactone autoinducer peptide	BEK99_RS03325	SCA_RS07840	SE1039_RS08795	SEQMU2_RS00685	BK815_RS00600	A6V26_RS12615	AA913_RS13230	SXYL_RS04390	BE24_RS07345	SSP_RS04185
accessory gene regulator AgrB	BEK99_RS03330	SCA_RS07835	SE1039_RS08790	SEQMU2_RS00680	BK815_RS00605	A6V26_RS12610	AA913_RS13235	SXYL_RS04395	BE24_RS07340	SSP_RS04190
carbon-nitrogen family hydrolase	BEK99_RS03340	SCA_RS07825	SE1039_RS08785	SEQMU2_RS00675	BK815_RS00610	A6V26_RS12605	AA913_RS13240	SXYL_RS04400	BE24_RS07335	SSP_RS04195
CPBP family intramembrane metalloprotease	BEK99_RS03350	SCA_RS07815	SE1039_RS08770	SEQMU2_RS00660	BK815_RS00625	A6V26_RS12590	AA913_RS10120	SXYL_RS04415	BE24_RS07320	SSP_RS04210
co-chaperone GroES	BEK99_RS03355	SCA_RS07810	SE1039_RS08765	SEQMU2_RS00655	BK815_RS00630	A6V26_RS12585	AA913_RS10125	SXYL_RS04420	BE24_RS07315	SSP_RS04215
molecular chaperone GroEL	BEK99_RS03360	SCA_RS07805	SE1039_RS08760	SEQMU2_RS00650	BK815_RS00635	A6V26_RS12580	AA913_RS10130	SXYL_RS04425	BE24_RS07310	SSP_RS04220
aspartate aminotransferase	BEK99_RS03385	SCA_RS07665	SE1039_RS08745	SEQMU2_RS00635	BK815_RS00645	A6V26_RS12570	AA913_RS10140	SXYL_RS04435	BE24_RS07300	SSP_RS04245
hypothetical protein	BEK99_RS03390	SCA_RS07660	SE1039_RS08740	SEQMU2_RS00630	BK815_RS00650	A6V26_RS12565	AA913_RS10145	SXYL_RS04440	BE24_RS07295	SSP_RS04250
GntR family transcriptional regulator	BEK99_RS03395	SCA_RS07655	SE1039_RS08735	SEQMU2_RS00625	BK815_RS00655	A6V26_RS12560	AA913_RS10150	SXYL_RS04445	BE24_RS07290	SSP_RS04255
ABC transporter ATP-binding protein	BEK99_RS03410	SCA_RS07640	SE1039_RS08720	SEQMU2_RS00610	BK815_RS00670	A6V26_RS12545	AA913_RS10165	SXYL_RS04460	BE24_RS07275	SSP_RS04275
ABC transporter permease	BEK99_RS03415	SCA_RS07635	SE1039_RS08715	SEQMU2_RS00605	BK815_RS00675	A6V26_RS12540	AA913_RS10170	SXYL_RS04465	BE24_RS07270	SSP_RS04280
thioredoxin family protein	BEK99_RS03420	SCA_RS07630	SE1039_RS08710	SEQMU2_RS00600	BK815_RS00680	A6V26_RS12535	AA913_RS10175	SXYL_RS04470	BE24_RS07265	SSP_RS04285
hypothetical protein	BEK99_RS03430	SCA_RS07620	SE1039_RS08700	SEQMU2_RS00590	BK815_RS00690	A6V26_RS12525	AA913_RS10185	SXYL_RS04480	BE24_RS07255	SSP_RS04295
choloylglycine hydrolase	BEK99_RS03440	SCA_RS07605	SE1039_RS08690	SEQMU2_RS00565	BK815_RS00700	A6V26_RS12515	AA913_RS10195	SXYL_RS04490	BE24_RS07245	SSP_RS04305
YolD-like family protein	BEK99_RS03450	SCA_RS07595	SE1039_RS08685	SEQMU2_RS00560	BK815_RS00710	A6V26_RS12510	AA913_RS10200	SXYL_RS04495	BE24_RS07240	SSP_RS04310
hypothetical protein	BEK99_RS03455	SCA_RS07590	SE1039_RS08680	SEQMU2_RS00555	BK815_RS00730	A6V26_RS12480	AA913_RS10235	SXYL_RS04500	BE24_RS07235	SSP_RS04315
pectate lyase	BEK99_RS03465	SCA_RS07580	SE1039_RS08660	SEQMU2_RS00535	BK815_RS00750	A6V26_RS12460	AA913_RS10255	SXYL_RS04520	BE24_RS07215	SSP_RS04335
cysteine hydrolase	BEK99_RS03470	SCA_RS07575	SE1039_RS08665	SEQMU2_RS00540	BK815_RS00745	A6V26_RS12465	AA913_RS10250	SXYL_RS04515	BE24_RS07220	SSP_RS04330
manganese-dependent inorganic pyrophosphatase	BEK99_RS03475	SCA_RS07570	SE1039_RS08670	SEQMU2_RS00545	BK815_RS00740	A6V26_RS12470	AA913_RS10245	SXYL_RS04510	BE24_RS07225	SSP_RS04325
aldehyde dehydrogenase	BEK99_RS03480	SCA_RS07565	SE1039_RS08675	SEQMU2_RS00550	BK815_RS00735	A6V26_RS12475	AA913_RS10240	SXYL_RS04505	BE24_RS07230	SSP_RS04320
prephenate dehydratase	BEK99_RS03490	SCA_RS07555	SE1039_RS08650	SEQMU2_RS00525	BK815_RS00760	A6V26_RS12450	AA913_RS11990	SXYL_RS04530	BE24_RS07205	SSP_RS04345
nitric oxide synthase	BEK99_RS03495	SCA_RS07550	SE1039_RS08645	SEQMU2_RS00520	BK815_RS00765	A6V26_RS12445	AA913_RS11985	SXYL_RS04535	BE24_RS07200	SSP_RS04350
nicotinate phosphoribosyltransferase	BEK99_RS03500	SCA_RS07545	SE1039_RS08640	SEQMU2_RS00515	BK815_RS00770	A6V26_RS12440	AA913_RS11980	SXYL_RS04540	BE24_RS07195	SSP_RS04355

Product	*S. carnosus*		*S. equorum*		*S. succinus*			*S. xylosus*		*S. saprophyticus*
	**JCM 6069**	**TM300**	**KS1039**	**Mu2**	**14BME20**	**CSM 77**	**DSM 14617**	**C2a**	**HKUOPL8**	**ATCC 15305**
NAD(+) synthase	BEK99_RS03505	SCA_RS07540	SE1039_RS08635	SEQMU2_RS00510	BK815_RS00775	A6V26_RS12435	AA913_RS11975	SXYL_RS04545	BE24_RS07190	SSP_RS04360
membrane protein	BEK99_RS03510	SCA_RS07535	SE1039_RS08630	SEQMU2_RS00505	BK815_RS00780	A6V26_RS12430	AA913_RS11970	SXYL_RS04550	BE24_RS07185	SSP_RS04365
NETI motif-containing protein	BEK99_RS03515	SCA_RS12680	SE1039_RS08625	SEQMU2_RS00500	BK815_RS00785	A6V26_RS12425	AA913_RS11965	SXYL_RS04555	BE24_RS07180	SSP_RS04370
adenylosuccinate lyase	BEK99_RS03520	SCA_RS07530	SE1039_RS08620	SEQMU2_RS00495	BK815_RS00790	A6V26_RS12420	AA913_RS11960	SXYL_RS04560	BE24_RS07175	SSP_RS04375
hypothetical protein	BEK99_RS03525	SCA_RS07525	SE1039_RS08615	SEQMU2_RS00490	BK815_RS00795	A6V26_RS12415	AA913_RS11955	SXYL_RS04565	BE24_RS07170	SSP_RS04380
geranylgeranylglyceryl/heptaprenylglyceryl phosphate synthase	BEK99_RS03530	SCA_RS07520	SE1039_RS08605	SEQMU2_RS00480	BK815_RS00805	A6V26_RS12405	AA913_RS11945	SXYL_RS04575	BE24_RS07160	SSP_RS04390
DNA ligase (NAD(+)) LigA	BEK99_RS03540	SCA_RS07510	SE1039_RS08595	SEQMU2_RS00470	BK815_RS00815	A6V26_RS12395	AA913_RS11935	SXYL_RS04585	BE24_RS07150	SSP_RS04400
Asp-tRNA(Asn)/Glu-tRNA(Gln) amidotransferase GatCAB subunit C	BEK99_RS03560	SCA_RS07490	SE1039_RS08585	SEQMU2_RS00460	BK815_RS00830	A6V26_RS12380	AA913_RS11920	SXYL_RS04600	BE24_RS07135	SSP_RS04415
Asp-tRNA(Asn)/Glu-tRNA(Gln) amidotransferase GatCAB subunit B	BEK99_RS03570	SCA_RS07480	SE1039_RS08575	SEQMU2_RS00450	BK815_RS00840	A6V26_RS12370	AA913_RS11910	SXYL_RS04610	BE24_RS07125	SSP_RS04425
diacylglycerol kinase	BEK99_RS03575	SCA_RS07475	SE1039_RS08570	SEQMU2_RS00445	BK815_RS00845	A6V26_RS12365	AA913_RS11905	SXYL_RS04615	BE24_RS07120	SSP_RS04430
23S rRNA (uracil-5-)-methyltransferase RumA	BEK99_RS03580	SCA_RS07470	SE1039_RS08565	SEQMU2_RS00440	BK815_RS00850	A6V26_RS12360	AA913_RS11900	SXYL_RS04620	BE24_RS07115	SSP_RS04435
DNA polymerase IV	BEK99_RS03590	SCA_RS07460	SE1039_RS08555	SEQMU2_RS00430	BK815_RS00860	A6V26_RS12350	AA913_RS11890	SXYL_RS04630	BE24_RS07105	SSP_RS04445
DNA polymerase III subunit epsilon	BEK99_RS03595	SCA_RS07455	SE1039_RS08550	SEQMU2_RS00425	BK815_RS00865	A6V26_RS12345	AA913_RS11885	SXYL_RS04635	BE24_RS07100	SSP_RS04450
UDP-N-acetylmuramate--alanine ligase	BEK99_RS03600	SCA_RS07450	SE1039_RS08540	SEQMU2_RS00415	BK815_RS00875	A6V26_RS12335	AA913_RS11875	SXYL_RS04645	BE24_RS07090	SSP_RS04460
glutamine amidotransferase	BEK99_RS03605	SCA_RS07445	SE1039_RS08535	SEQMU2_RS00410	BK815_RS00880	A6V26_RS12330	AA913_RS11870	SXYL_RS04650	BE24_RS07085	SSP_RS04465
type I methionyl aminopeptidase	BEK99_RS03615	SCA_RS07435	SE1039_RS08525	SEQMU2_RS00400	BK815_RS00890	A6V26_RS12320	AA913_RS11860	SXYL_RS04660	BE24_RS07075	SSP_RS04490
hypothetical protein	BEK99_RS03620	SCA_RS07430	SE1039_RS08520	SEQMU2_RS00395	BK815_RS00895	A6V26_RS12315	AA913_RS11855	SXYL_RS04665	BE24_RS07070	SSP_RS04495
sensor histidine kinase	BEK99_RS03630	SCA_RS07420	SE1039_RS08510	SEQMU2_RS00385	BK815_RS00905	A6V26_RS12305	AA913_RS11845	SXYL_RS04675	BE24_RS07060	SSP_RS04505
DNA-binding response regulator	BEK99_RS03635	SCA_RS07415	SE1039_RS08505	SEQMU2_RS00380	BK815_RS00910	A6V26_RS12300	AA913_RS11840	SXYL_RS04680	BE24_RS07055	SSP_RS04510
YihY/virulence factor BrkB family protein	BEK99_RS03645	SCA_RS07405	SE1039_RS08500	SEQMU2_RS00375	BK815_RS00915	A6V26_RS12295	AA913_RS11835	SXYL_RS04685	BE24_RS07050	SSP_RS04515
hypothetical protein	BEK99_RS03650	SCA_RS07400	SE1039_RS08495	SEQMU2_RS00370	BK815_RS00920	A6V26_RS12290	AA913_RS11830	SXYL_RS04690	BE24_RS07045	SSP_RS04520
low molecular weight phosphotyrosine protein phosphatase	BEK99_RS03655	SCA_RS07395	SE1039_RS08490	SEQMU2_RS00365	BK815_RS00925	A6V26_RS12285	AA913_RS11825	SXYL_RS04695	BE24_RS07040	SSP_RS04525
hypothetical protein	BEK99_RS03660	SCA_RS07390	SE1039_RS08485	SEQMU2_RS00360	BK815_RS00930	A6V26_RS12280	AA913_RS11820	SXYL_RS04700	BE24_RS07035	SSP_RS04530
aminopeptidase	BEK99_RS03665	SCA_RS07385	SE1039_RS08480	SEQMU2_RS00355	BK815_RS00935	A6V26_RS12275	AA913_RS11815	SXYL_RS04705	BE24_RS07030	SSP_RS04535
acyl-CoA thioesterase	BEK99_RS03670	SCA_RS07380	SE1039_RS08475	SEQMU2_RS00350	BK815_RS00940	A6V26_RS12270	AA913_RS11810	SXYL_RS04710	BE24_RS07025	SSP_RS04540
glutamine amidotransferase	BEK99_RS03680	SCA_RS07370	SE1039_RS08470	SEQMU2_RS00345	BK815_RS00950	A6V26_RS12260	AA913_RS11800	SXYL_RS04725	BE24_RS07010	SSP_RS04550
glycosyltransferase	BEK99_RS03685	SCA_RS07365	SE1039_RS08465	SEQMU2_RS00340	BK815_RS00955	A6V26_RS12255	AA913_RS11795	SXYL_RS04730	BE24_RS07005	SSP_RS04555
hypothetical protein	BEK99_RS03695	SCA_RS07355	SE1039_RS08455	SEQMU2_RS00330	BK815_RS00965	A6V26_RS12245	AA913_RS11785	SXYL_RS04740	BE24_RS06995	SSP_RS04565
hypothetical protein	BEK99_RS03705	SCA_RS07345	SE1039_RS08445	SEQMU2_RS00320	BK815_RS00975	A6V26_RS12235	AA913_RS11775	SXYL_RS04750	BE24_RS06985	SSP_RS04575
metal-dependent hydrolase	BEK99_RS03710	SCA_RS07340	SE1039_RS08440	SEQMU2_RS13550	BK815_RS00980	A6V26_RS12230	AA913_RS11770	SXYL_RS04755	BE24_RS06980	SSP_RS04580
A/G-specific adenine glycosylase	BEK99_RS03715	SCA_RS07335	SE1039_RS08435	SEQMU2_RS13545	BK815_RS00985	A6V26_RS12225	AA913_RS11765	SXYL_RS04760	BE24_RS06975	SSP_RS04585

Product	*S. carnosus*		*S. equorum*		*S. succinus*			*S. xylosus*		*S. saprophyticus*
	**JCM 6069**	**TM300**	**KS1039**	**Mu2**	**14BME20**	**CSM 77**	**DSM 14617**	**C2a**	**HKUOPL8**	**ATCC 15305**
hypothetical protein	BEK99_RS03720	SCA_RS07330	SE1039_RS08430	SEQMU2_RS13540	BK815_RS00990	A6V26_RS12220	AA913_RS11760	SXYL_RS04765	BE24_RS06970	SSP_RS04590
ABC transporter ATP-binding protein	BEK99_RS03725	SCA_RS07325	SE1039_RS08425	SEQMU2_RS13535	BK815_RS00995	A6V26_RS12215	AA913_RS11755	SXYL_RS04770	BE24_RS06965	SSP_RS04595
aromatic acid exporter family protein	BEK99_RS03730	SCA_RS07320	SE1039_RS08420	SEQMU2_RS13530	BK815_RS01000	A6V26_RS12210	AA913_RS11750	SXYL_RS04775	BE24_RS06960	SSP_RS04600
peroxiredoxin	BEK99_RS03740	SCA_RS07310	SE1039_RS08410	SEQMU2_RS13520	BK815_RS01010	A6V26_RS12200	AA913_RS11740	SXYL_RS04785	BE24_RS06950	SSP_RS04610
hydroxyacid dehydrogenase	BEK99_RS03745	SCA_RS07305	SE1039_RS08405	SEQMU2_RS13515	BK815_RS01015	A6V26_RS12195	AA913_RS11735	SXYL_RS04790	BE24_RS06945	SSP_RS04615
transcriptional repressor	BEK99_RS03750	SCA_RS07300	SE1039_RS08400	SEQMU2_RS13510	BK815_RS01020	A6V26_RS12190	AA913_RS11730	SXYL_RS04795	BE24_RS06940	SSP_RS04620
PTS transporter subunit IIC	BEK99_RS03895	SCA_RS07160	SE1039_RS08250	SEQMU2_RS13380	BK815_RS01170	A6V26_RS11505	AA913_RS12070	SXYL_RS04950	BE24_RS06605	SSP_RS04775
amino acid ABC transporter ATP-binding protein	BEK99_RS03905	SCA_RS07150	SE1039_RS08240	SEQMU2_RS13370	BK815_RS01180	A6V26_RS11495	AA913_RS12080	SXYL_RS04960	BE24_RS06595	SSP_RS04785
epoxyqueuosine reductase	BEK99_RS03910	SCA_RS07145	SE1039_RS08235	SEQMU2_RS13365	BK815_RS01185	A6V26_RS11490	AA913_RS12085	SXYL_RS04965	BE24_RS06590	SSP_RS04790
glucosamine-6-phosphate isomerase	BEK99_RS03920	SCA_RS07135	SE1039_RS08225	SEQMU2_RS13355	BK815_RS01195	A6V26_RS11480	AA913_RS12095	SXYL_RS04975	BE24_RS06580	SSP_RS04800
hypothetical protein	BEK99_RS03925	SCA_RS07130	SE1039_RS08215	SEQMU2_RS13345	BK815_RS01205	A6V26_RS11470	AA913_RS12105	SXYL_RS04985	BE24_RS06570	SSP_RS04810
class II fumarate hydratase	BEK99_RS03930	SCA_RS07125	SE1039_RS08210	SEQMU2_RS13340	BK815_RS01210	A6V26_RS11465	AA913_RS12110	SXYL_RS04990	BE24_RS06565	SSP_RS04815
RNA pseudouridine synthase	BEK99_RS03935	SCA_RS07120	SE1039_RS08205	SEQMU2_RS13335	BK815_RS01215	A6V26_RS11460	AA913_RS12115	SXYL_RS04995	BE24_RS06560	SSP_RS04820
DNA-binding response regulator	BEK99_RS03945	SCA_RS07110	SE1039_RS08195	SEQMU2_RS13325	BK815_RS01225	A6V26_RS11450	AA913_RS12125	SXYL_RS05005	BE24_RS06550	SSP_RS04830
dicarboxylate/amino acid:cation symporter	BEK99_RS03950	SCA_RS07105	SE1039_RS08190	SEQMU2_RS13320	BK815_RS01230	A6V26_RS11445	AA913_RS12130	SXYL_RS05010	BE24_RS06545	SSP_RS04835
transcriptional regulator	BEK99_RS03955	SCA_RS07100	SE1039_RS08170	SEQMU2_RS13295	BK815_RS01280	A6V26_RS11395	AA913_RS12380	SXYL_RS05055	BE24_RS06490	SSP_RS04855
DUF445 domain-containing protein	BEK99_RS03960	SCA_RS07095	SE1039_RS08165	SEQMU2_RS13290	BK815_RS01285	A6V26_RS11390	AA913_RS12385	SXYL_RS05060	BE24_RS06485	SSP_RS04860
hypothetical protein	BEK99_RS03965	SCA_RS07090	SE1039_RS08160	SEQMU2_RS13285	BK815_RS01290	A6V26_RS11385	AA913_RS12390	SXYL_RS05065	BE24_RS06480	SSP_RS04865
DNA repair exonuclease	BEK99_RS03970	SCA_RS07085	SE1039_RS08155	SEQMU2_RS13280	BK815_RS01295	A6V26_RS11380	AA913_RS12395	SXYL_RS05070	BE24_RS06475	SSP_RS04870
DNA repair protein Rad50	BEK99_RS03975	SCA_RS07080	SE1039_RS08150	SEQMU2_RS13275	BK815_RS01300	A6V26_RS11375	AA913_RS12400	SXYL_RS05075	BE24_RS06470	SSP_RS04875
3-5 exoribonuclease YhaM	BEK99_RS03980	SCA_RS07075	SE1039_RS08145	SEQMU2_RS13270	BK815_RS01305	A6V26_RS11370	AA913_RS12405	SXYL_RS05080	BE24_RS06465	SSP_RS04880
peptidylprolyl isomerase	BEK99_RS03985	SCA_RS07070	SE1039_RS08140	SEQMU2_RS13265	BK815_RS01310	A6V26_RS11365	AA913_RS12410	SXYL_RS05085	BE24_RS06460	SSP_RS04885
hypothetical protein	BEK99_RS03990	SCA_RS07065	SE1039_RS08135	SEQMU2_RS13260	BK815_RS01315	A6V26_RS11360	AA913_RS12415	SXYL_RS05090	BE24_RS06455	SSP_RS04890
HIT family protein	BEK99_RS04000	SCA_RS07055	SE1039_RS08125	SEQMU2_RS13250	BK815_RS01325	A6V26_RS11350	AA913_RS12425	SXYL_RS05100	BE24_RS06445	SSP_RS04900
ABC transporter ATP-binding protein	BEK99_RS04005	SCA_RS07050	SE1039_RS08120	SEQMU2_RS13245	BK815_RS01330	A6V26_RS11345	AA913_RS12430	SXYL_RS05105	BE24_RS06440	SSP_RS04905
multidrug ABC transporter ATP-binding protein	BEK99_RS04010	SCA_RS07045	SE1039_RS08115	SEQMU2_RS13240	BK815_RS01335	A6V26_RS11340	AA913_RS12435	SXYL_RS05110	BE24_RS06435	SSP_RS04910
hypothetical protein	BEK99_RS04015	SCA_RS07040	SE1039_RS08110	SEQMU2_RS13235	BK815_RS01340	A6V26_RS11335	AA913_RS12440	SXYL_RS05115	BE24_RS06430	SSP_RS04915
uroporphyrinogen decarboxylase	BEK99_RS04020	SCA_RS07035	SE1039_RS08105	SEQMU2_RS13230	BK815_RS01345	A6V26_RS11330	AA913_RS12445	SXYL_RS05120	BE24_RS06425	SSP_RS04920
ferrochelatase	BEK99_RS04025	SCA_RS07030	SE1039_RS08100	SEQMU2_RS13225	BK815_RS01350	A6V26_RS11325	AA913_RS12450	SXYL_RS05125	BE24_RS06420	SSP_RS04925
protoporphyrinogen oxidase	BEK99_RS04030	SCA_RS07025	SE1039_RS08095	SEQMU2_RS13220	BK815_RS01355	A6V26_RS11320	AA913_RS12455	SXYL_RS05130	BE24_RS06415	SSP_RS04930
serine hydrolase family protein	BEK99_RS04035	SCA_RS07020	SE1039_RS08090	SEQMU2_RS13215	BK815_RS01360	A6V26_RS11315	AA913_RS12460	SXYL_RS05135	BE24_RS06410	SSP_RS04935

Product	*S. carnosus*		*S. equorum*		*S. succinus*			*S. xylosus*		*S. saprophyticus*
	**JCM 6069**	**TM300**	**KS1039**	**Mu2**	**14BME20**	**CSM 77**	**DSM 14617**	**C2a**	**HKUOPL8**	**ATCC 15305**
o-succinylbenzoate--CoA ligase	BEK99_RS04425	SCA_RS06925	SE1039_RS08025	SEQMU2_RS13150	BK815_RS01420	A6V26_RS10970	AA913_RS06105	SXYL_RS05315	BE24_RS06300	SSP_RS04990
o-succinylbenzoate synthase	BEK99_RS04430	SCA_RS06920	SE1039_RS08020	SEQMU2_RS13145	BK815_RS01425	A6V26_RS10965	AA913_RS06100	SXYL_RS05320	BE24_RS06295	SSP_RS04995
membrane protein insertion efficiency factor YidD	BEK99_RS04435	SCA_RS06915	SE1039_RS08015	SEQMU2_RS13140	BK815_RS01430	A6V26_RS10960	AA913_RS06095	SXYL_RS05325	BE24_RS06290	SSP_RS05000
nucleoside triphosphatase YtkD	BEK99_RS04440	SCA_RS06910	SE1039_RS08010	SEQMU2_RS13135	BK815_RS01435	A6V26_RS10955	AA913_RS06090	SXYL_RS05330	BE24_RS06285	SSP_RS05005
S9 family peptidase	BEK99_RS04445	SCA_RS06905	SE1039_RS08005	SEQMU2_RS13130	BK815_RS01440	A6V26_RS10950	AA913_RS06085	SXYL_RS05335	BE24_RS06280	SSP_RS05015
phosphoenolpyruvate carboxykinase (ATP)	BEK99_RS04450	SCA_RS06900	SE1039_RS08000	SEQMU2_RS13125	BK815_RS01445	A6V26_RS10945	AA913_RS06080	SXYL_RS05340	BE24_RS06275	SSP_RS05020
methionine adenosyltransferase	BEK99_RS04455	SCA_RS06895	SE1039_RS07995	SEQMU2_RS13120	BK815_RS01450	A6V26_RS10940	AA913_RS06075	SXYL_RS05345	BE24_RS06265	SSP_RS05025
hypothetical protein	BEK99_RS04460	SCA_RS06890	SE1039_RS07990	SEQMU2_RS13115	BK815_RS01455	A6V26_RS10935	AA913_RS06070	SXYL_RS05350	BE24_RS06260	SSP_RS05030
aldo/keto reductase	BEK99_RS04470	SCA_RS06880	SE1039_RS07985	SEQMU2_RS13110	BK815_RS01460	A6V26_RS10930	AA913_RS06065	SXYL_RS05355	BE24_RS06255	SSP_RS05035
CrcB family protein	BEK99_RS04480	SCA_RS06870	SE1039_RS07980	SEQMU2_RS13105	BK815_RS01465	A6V26_RS10925	AA913_RS06060	SXYL_RS05360	BE24_RS06250	SSP_RS05040
hypothetical protein	BEK99_RS04515	SCA_RS06840	SE1039_RS07940	SEQMU2_RS13065	BK815_RS01505	A6V26_RS10885	AA913_RS06020	SXYL_RS05400	BE24_RS06245	SSP_RS05080
riboflavin biosynthesis protein RibD	BEK99_RS04530	SCA_RS06825	SE1039_RS07920	SEQMU2_RS13045	BK815_RS01525	A6V26_RS10865	AA913_RS06000	SXYL_RS05420	BE24_RS06225	SSP_RS05110
riboflavin synthase	BEK99_RS04535	SCA_RS06820	SE1039_RS07915	SEQMU2_RS13040	BK815_RS01530	A6V26_RS10860	AA913_RS05995	SXYL_RS05425	BE24_RS06220	SSP_RS05115
6,7-dimethyl-8-ribityllumazine synthase	BEK99_RS04545	SCA_RS06810	SE1039_RS07905	SEQMU2_RS13030	BK815_RS01540	A6V26_RS10850	AA913_RS05985	SXYL_RS05435	BE24_RS06210	SSP_RS05125
proline dehydrogenase	BEK99_RS04550	SCA_RS06805	SE1039_RS07900	SEQMU2_RS13025	BK815_RS01545	A6V26_RS10845	AA913_RS05980	SXYL_RS05440	BE24_RS06205	SSP_RS05130
alpha/beta hydrolase	BEK99_RS04555	SCA_RS06800	SE1039_RS07895	SEQMU2_RS13020	BK815_RS01550	A6V26_RS10840	AA913_RS05975	SXYL_RS05445	BE24_RS06200	SSP_RS05135
rRNA methyltransferase	BEK99_RS04565	SCA_RS06790	SE1039_RS07880	SEQMU2_RS13005	BK815_RS01565	A6V26_RS10825	AA913_RS05960	SXYL_RS05460	BE24_RS06185	SSP_RS05150
TIGR01212 family radical SAM protein	BEK99_RS04570	SCA_RS06785	SE1039_RS07875	SEQMU2_RS13000	BK815_RS01570	A6V26_RS10820	AA913_RS05955	SXYL_RS05465	BE24_RS06180	SSP_RS05155
MFS transporter	BEK99_RS04575	SCA_RS06780	SE1039_RS07870	SEQMU2_RS12995	BK815_RS01575	A6V26_RS10815	AA913_RS05950	SXYL_RS05470	BE24_RS06175	SSP_RS05160
rhodanese-like domain-containing protein	BEK99_RS04585	SCA_RS06770	SE1039_RS07860	SEQMU2_RS12985	BK815_RS01585	A6V26_RS10805	AA913_RS05940	SXYL_RS05480	BE24_RS06165	SSP_RS05170
polysaccharide biosynthesis protein	BEK99_RS04595	SCA_RS06760	SE1039_RS07850	SEQMU2_RS12975	BK815_RS01595	A6V26_RS10775	AA913_RS05915	SXYL_RS05490	BE24_RS06155	SSP_RS05180
16S rRNA pseudouridine(516) synthase	BEK99_RS04600	SCA_RS06755	SE1039_RS07845	SEQMU2_RS12970	BK815_RS01600	A6V26_RS10770	AA913_RS05910	SXYL_RS05495	BE24_RS06150	SSP_RS05185
YtxH domain-containing protein	BEK99_RS04605	SCA_RS06750	SE1039_RS07840	SEQMU2_RS12965	BK815_RS01605	A6V26_RS10765	AA913_RS05905	SXYL_RS05500	BE24_RS06145	SSP_RS05190
dipeptidase PepV	BEK99_RS04610	SCA_RS06745	SE1039_RS07835	SEQMU2_RS12960	BK815_RS01610	A6V26_RS10760	AA913_RS05900	SXYL_RS05505	BE24_RS06140	SSP_RS05195
D-amino-acid transaminase	BEK99_RS04615	SCA_RS06740	SE1039_RS07830	SEQMU2_RS12955	BK815_RS01615	A6V26_RS10755	AA913_RS05895	SXYL_RS05510	BE24_RS06135	SSP_RS05200
tRNA (guanosine(46)-N7)-methyltransferase TrmB	BEK99_RS04625	SCA_RS06730	SE1039_RS07820	SEQMU2_RS12945	BK815_RS01625	A6V26_RS10745	AA913_RS05885	SXYL_RS05520	BE24_RS06125	SSP_RS05210
MBL fold metallo-hydrolase	BEK99_RS04630	SCA_RS06725	SE1039_RS07815	SEQMU2_RS12940	BK815_RS01630	A6V26_RS10740	AA913_RS05880	SXYL_RS05525	BE24_RS06120	SSP_RS05215
hypothetical protein	BEK99_RS04635	SCA_RS06720	SE1039_RS07810	SEQMU2_RS12935	BK815_RS01635	A6V26_RS10735	AA913_RS05875	SXYL_RS05530	BE24_RS06115	SSP_RS05220
peptidase M28	BEK99_RS04640	SCA_RS06715	SE1039_RS07805	SEQMU2_RS12930	BK815_RS01640	A6V26_RS10730	AA913_RS05870	SXYL_RS05535	BE24_RS06110	SSP_RS05225
thiol reductase thioredoxin	BEK99_RS04645	SCA_RS06710	SE1039_RS07800	SEQMU2_RS12925	BK815_RS01645	A6V26_RS10725	AA913_RS05865	SXYL_RS05540	BE24_RS06105	SSP_RS05230
DUF1444 domain-containing protein	BEK99_RS04650	SCA_RS06705	SE1039_RS07795	SEQMU2_RS12920	BK815_RS01650	A6V26_RS10720	AA913_RS05860	SXYL_RS05545	BE24_RS06100	SSP_RS05235

Product	*S. carnosus*		*S. equorum*		*S. succinus*			*S. xylosus*		*S. saprophyticus*
	**JCM 6069**	**TM300**	**KS1039**	**Mu2**	**14BME20**	**CSM 77**	**DSM 14617**	**C2a**	**HKUOPL8**	**ATCC 15305**
tRNA-binding protein	BEK99_RS04655	SCA_RS06700	SE1039_RS07790	SEQMU2_RS12915	BK815_RS01655	A6V26_RS10715	AA913_RS05855	SXYL_RS05550	BE24_RS06095	SSP_RS05240
UDP-N-acetylmuramate--L-alanine ligase	BEK99_RS04665	SCA_RS06690	SE1039_RS07780	SEQMU2_RS12905	BK815_RS01665	A6V26_RS10705	AA913_RS05845	SXYL_RS05560	BE24_RS06085	SSP_RS05250
DUF948 domain containing protein	BEK99_RS04670	SCA_RS06685	SE1039_RS07775	SEQMU2_RS12900	BK815_RS01670	A6V26_RS10700	AA913_RS05840	SXYL_RS05565	BE24_RS06080	SSP_RS05255
chorismate mutase	BEK99_RS04680	SCA_RS06675	SE1039_RS07765	SEQMU2_RS12890	BK815_RS01680	A6V26_RS10690	AA913_RS05830	SXYL_RS05575	BE24_RS06070	SSP_RS05265
catabolite control protein A	BEK99_RS04685	SCA_RS06670	SE1039_RS07760	SEQMU2_RS12885	BK815_RS01685	A6V26_RS10685	AA913_RS05825	SXYL_RS05580	BE24_RS06065	SSP_RS05270
N-acetyltransferase	BEK99_RS04695	SCA_RS06660	SE1039_RS07750	SEQMU2_RS12875	BK815_RS01695	A6V26_RS10675	AA913_RS05815	SXYL_RS05590	BE24_RS06050	SSP_RS05280
transglycosylase	BEK99_RS04710	SCA_RS06645	SE1039_RS07735	SEQMU2_RS12860	BK815_RS01710	A6V26_RS10660	AA913_RS05800	SXYL_RS05605	BE24_RS06035	SSP_RS05295
tyrosine--tRNA ligase	BEK99_RS04715	SCA_RS06640	SE1039_RS07730	SEQMU2_RS12855	BK815_RS01715	A6V26_RS10655	AA913_RS05795	SXYL_RS05610	BE24_RS06030	SSP_RS05300
serine protease	BEK99_RS04720	SCA_RS06635	SE1039_RS07725	SEQMU2_RS12850	BK815_RS01720	A6V26_RS10650	AA913_RS05790	SXYL_RS05615	BE24_RS06025	SSP_RS05305
1-acyl-sn-glycerol-3-phosphate acyltransferase	BEK99_RS04725	SCA_RS06630	SE1039_RS07720	SEQMU2_RS12845	BK815_RS01725	A6V26_RS10645	AA913_RS05785	SXYL_RS05620	BE24_RS06020	SSP_RS05310
HAD family hydrolase	BEK99_RS04735	SCA_RS06620	SE1039_RS07710	SEQMU2_RS12825	BK815_RS01735	A6V26_RS10635	AA913_RS05775	SXYL_RS05630	BE24_RS06010	SSP_RS05320
phosphoglycerate dehydrogenase	BEK99_RS04740	SCA_RS06615	SE1039_RS07705	SEQMU2_RS12820	BK815_RS01740	A6V26_RS10630	AA913_RS05770	SXYL_RS05635	BE24_RS06005	SSP_RS05325
alanine--glyoxylate aminotransferase family protein	BEK99_RS04745	SCA_RS06610	SE1039_RS07700	SEQMU2_RS12815	BK815_RS01745	A6V26_RS10625	AA913_RS05765	SXYL_RS05640	BE24_RS06000	SSP_RS05330
peroxiredoxin	BEK99_RS04750	SCA_RS06605	SE1039_RS07695	SEQMU2_RS12810	BK815_RS01750	A6V26_RS10620	AA913_RS05760	SXYL_RS05645	BE24_RS05995	SSP_RS05335
glycerophosphoryl diester phosphodiesterase	BEK99_RS04755	SCA_RS06600	SE1039_RS07690	SEQMU2_RS12805	BK815_RS01755	A6V26_RS10615	AA913_RS05755	SXYL_RS05650	BE24_RS05990	SSP_RS05340
30S ribosomal protein S4	BEK99_RS04760	SCA_RS06595	SE1039_RS07680	SEQMU2_RS12795	BK815_RS01765	A6V26_RS10605	AA913_RS05745	SXYL_RS05660	BE24_RS05980	SSP_RS05350
septation ring formation regulator EzrA	BEK99_RS04770	SCA_RS06585	SE1039_RS07670	SEQMU2_RS12785	BK815_RS01775	A6V26_RS10595	AA913_RS05735	SXYL_RS05670	BE24_RS05970	SSP_RS05360
cysteine desulfurase	BEK99_RS04775	SCA_RS06580	SE1039_RS07665	SEQMU2_RS12780	BK815_RS01780	A6V26_RS10590	AA913_RS05730	SXYL_RS05675	BE24_RS05965	SSP_RS05365
membrane protein	BEK99_RS04785	SCA_RS06570	SE1039_RS07655	SEQMU2_RS12770	BK815_RS01790	A6V26_RS10580	AA913_RS05720	SXYL_RS05685	BE24_RS05955	SSP_RS05375
2-Cys peroxiredoxin	BEK99_RS04790	SCA_RS06565	SE1039_RS07640	SEQMU2_RS12755	BK815_RS01805	A6V26_RS10565	AA913_RS05705	SXYL_RS05700	BE24_RS05940	SSP_RS05390
class I SAM-dependent methyltransferase	BEK99_RS04795	SCA_RS06560	SE1039_RS07635	SEQMU2_RS12750	BK815_RS01810	A6V26_RS10560	AA913_RS05700	SXYL_RS05705	BE24_RS05935	SSP_RS05395
universal stress protein	BEK99_RS04805	SCA_RS06550	SE1039_RS07620	SEQMU2_RS12735	BK815_RS01825	A6V26_RS10545	AA913_RS05685	SXYL_RS05720	BE24_RS05920	SSP_RS05410
alanine dehydrogenase	BEK99_RS04810	SCA_RS06545	SE1039_RS07615	SEQMU2_RS12730	BK815_RS01830	A6V26_RS10540	AA913_RS05680	SXYL_RS05725	BE24_RS05915	SSP_RS05415
peptidase M24 family protein	BEK99_RS04815	SCA_RS06540	SE1039_RS07605	SEQMU2_RS12720	BK815_RS01840	A6V26_RS10530	AA913_RS05670	SXYL_RS05735	BE24_RS05905	SSP_RS05425
metal-dependent hydrolase	BEK99_RS04820	SCA_RS06535	SE1039_RS07600	SEQMU2_RS12715	BK815_RS01845	A6V26_RS10525	AA913_RS05665	SXYL_RS05740	BE24_RS05900	SSP_RS05430
universal stress protein	BEK99_RS04825	SCA_RS06530	SE1039_RS07595	SEQMU2_RS12710	BK815_RS01850	A6V26_RS10520	AA913_RS05660	SXYL_RS05745	BE24_RS05895	SSP_RS05435
DNA polymerase III subunit alpha	BEK99_RS04840	SCA_RS06515	SE1039_RS07580	SEQMU2_RS12695	BK815_RS01865	A6V26_RS10505	AA913_RS05645	SXYL_RS05760	BE24_RS05880	SSP_RS05450
malate dehydrogenase	BEK99_RS04845	SCA_RS06510	SE1039_RS07575	SEQMU2_RS12690	BK815_RS01870	A6V26_RS10500	AA913_RS05640	SXYL_RS05765	BE24_RS05875	SSP_RS05455
acetyl-CoA carboxylase carboxyl transferase subunit alpha	BEK99_RS04855	SCA_RS06500	SE1039_RS07565	SEQMU2_RS12680	BK815_RS01880	A6V26_RS10490	AA913_RS05630	SXYL_RS05775	BE24_RS05865	SSP_RS05465
ATP-dependent 6-phosphofructokinase	BEK99_RS04860	SCA_RS06495	SE1039_RS07560	SEQMU2_RS12675	BK815_RS01885	A6V26_RS10485	AA913_RS05625	SXYL_RS05780	BE24_RS05860	SSP_RS05470
pyruvate kinase	BEK99_RS04865	SCA_RS06490	SE1039_RS07555	SEQMU2_RS12670	BK815_RS01890	A6V26_RS10480	AA913_RS05620	SXYL_RS05785	BE24_RS05855	SSP_RS05475

Product	*S. carnosus*		*S. equorum*		*S. succinus*			*S. xylosus*		*S. saprophyticus*
	**JCM 6069**	**TM300**	**KS1039**	**Mu2**	**14BME20**	**CSM 77**	**DSM 14617**	**C2a**	**HKUOPL8**	**ATCC 15305**
gamma-aminobutyrate permease	BEK99_RS04870	SCA_RS06485	SE1039_RS07550	SEQMU2_RS12665	BK815_RS01895	A6V26_RS10475	AA913_RS05615	SXYL_RS05790	BE24_RS05850	SSP_RS05480
citrate synthase	BEK99_RS04875	SCA_RS06480	SE1039_RS07545	SEQMU2_RS12660	BK815_RS01900	A6V26_RS10470	AA913_RS05610	SXYL_RS05795	BE24_RS05845	SSP_RS05485
DNA-binding response regulator	BEK99_RS04885	SCA_RS06470	SE1039_RS07535	SEQMU2_RS12650	BK815_RS01910	A6V26_RS10460	AA913_RS05600	SXYL_RS05805	BE24_RS05835	SSP_RS05495
histidine kinase	BEK99_RS04890	SCA_RS06465	SE1039_RS07530	SEQMU2_RS12645	BK815_RS01915	A6V26_RS10455	AA913_RS05595	SXYL_RS05810	BE24_RS05830	SSP_RS05500
DNA-formamidopyrimidine glycosylase	BEK99_RS04900	SCA_RS06455	SE1039_RS07520	SEQMU2_RS12635	BK815_RS01925	A6V26_RS10445	AA913_RS05585	SXYL_RS05820	BE24_RS05820	SSP_RS05510
type I glyceraldehyde-3-phosphate dehydrogenase	BEK99_RS04910	SCA_RS06445	SE1039_RS07510	SEQMU2_RS12625	BK815_RS01935	A6V26_RS10435	AA913_RS05575	SXYL_RS05830	BE24_RS05810	SSP_RS05520
transcriptional regulator NrdR	BEK99_RS04915	SCA_RS06440	SE1039_RS07505	SEQMU2_RS12620	BK815_RS01940	A6V26_RS10430	AA913_RS05570	SXYL_RS05835	BE24_RS05805	SSP_RS05525
helicase DnaB	BEK99_RS04920	SCA_RS06435	SE1039_RS07500	SEQMU2_RS12615	BK815_RS01945	A6V26_RS10425	AA913_RS05565	SXYL_RS05840	BE24_RS05800	SSP_RS05530
primosomal protein DnaI	BEK99_RS04925	SCA_RS06430	SE1039_RS07495	SEQMU2_RS12610	BK815_RS01950	A6V26_RS10420	AA913_RS05560	SXYL_RS05845	BE24_RS05795	SSP_RS05535
threonine--tRNA ligase	BEK99_RS04930	SCA_RS06425	SE1039_RS07490	SEQMU2_RS12605	BK815_RS01955	A6V26_RS10415	AA913_RS05555	SXYL_RS05850	BE24_RS05790	SSP_RS05540
gamma-aminobutyrate permease	BEK99_RS04935	SCA_RS06420	SE1039_RS07485	SEQMU2_RS12600	BK815_RS01960	A6V26_RS10410	AA913_RS05550	SXYL_RS05855	BE24_RS05785	SSP_RS05545
50S ribosomal protein L35	BEK99_RS04945	SCA_RS06410	SE1039_RS07475	SEQMU2_RS12590	BK815_RS01970	A6V26_RS10400	AA913_RS05540	SXYL_RS05865	BE24_RS05775	SSP_RS05555
50S ribosomal protein L20	BEK99_RS04950	SCA_RS06405	SE1039_RS07470	SEQMU2_RS12585	BK815_RS01975	A6V26_RS10395	AA913_RS05535	SXYL_RS05870	BE24_RS05770	SSP_RS05560
DNA mismatch repair protein MutT	BEK99_RS04955	SCA_RS06400	SE1039_RS07465	SEQMU2_RS12580	BK815_RS01980	A6V26_RS10390	AA913_RS05530	SXYL_RS05875	BE24_RS05765	SSP_RS05565
hypothetical protein	BEK99_RS04960	SCA_RS06395	SE1039_RS07460	SEQMU2_RS12575	BK815_RS01985	A6V26_RS10385	AA913_RS05525	SXYL_RS05880	BE24_RS05760	SSP_RS05570
trigger factor	BEK99_RS04965	SCA_RS06390	SE1039_RS07455	SEQMU2_RS12570	BK815_RS01990	A6V26_RS10380	AA913_RS05520	SXYL_RS05885	BE24_RS05755	SSP_RS05575
ATP-dependent Clp protease ATP-binding subunit ClpX	BEK99_RS04970	SCA_RS06385	SE1039_RS07450	SEQMU2_RS12565	BK815_RS01995	A6V26_RS10375	AA913_RS05515	SXYL_RS05890	BE24_RS05750	SSP_RS05580
GTP-binding protein	BEK99_RS04975	SCA_RS06380	SE1039_RS07445	SEQMU2_RS12560	BK815_RS02000	A6V26_RS10370	AA913_RS05510	SXYL_RS05895	BE24_RS05745	SSP_RS05585
glutamyl-tRNA reductase	BEK99_RS04980	SCA_RS06375	SE1039_RS07440	SEQMU2_RS12555	BK815_RS02005	A6V26_RS10365	AA913_RS05505	SXYL_RS05900	BE24_RS05740	SSP_RS05590
cytochrome c assembly protein	BEK99_RS04985	SCA_RS06370	SE1039_RS07435	SEQMU2_RS12550	BK815_RS02010	A6V26_RS10360	AA913_RS05500	SXYL_RS05905	BE24_RS05735	SSP_RS05595
hydroxymethylbilane synthase	BEK99_RS04990	SCA_RS06365	SE1039_RS07430	SEQMU2_RS12545	BK815_RS02015	A6V26_RS10355	AA913_RS05495	SXYL_RS05910	BE24_RS05730	SSP_RS05600
uroporphyrinogen-III synthase	BEK99_RS04995	SCA_RS06360	SE1039_RS07425	SEQMU2_RS12540	BK815_RS02020	A6V26_RS10350	AA913_RS05490	SXYL_RS05915	BE24_RS05725	SSP_RS05605
porphobilinogen synthase	BEK99_RS05000	SCA_RS06355	SE1039_RS07420	SEQMU2_RS12535	BK815_RS02025	A6V26_RS10345	AA913_RS05485	SXYL_RS05920	BE24_RS05720	SSP_RS05610
glutamate-1-semialdehyde 2,1-aminomutase	BEK99_RS05005	SCA_RS06350	SE1039_RS07415	SEQMU2_RS12530	BK815_RS02030	A6V26_RS10340	AA913_RS05480	SXYL_RS05925	BE24_RS05715	SSP_RS05615
aminopeptidase	BEK99_RS05010	SCA_RS06345	SE1039_RS07410	SEQMU2_RS12525	BK815_RS02035	A6V26_RS10335	AA913_RS05475	SXYL_RS05930	BE24_RS05710	SSP_RS05620
pyroglutamyl-peptidase I	BEK99_RS05015	SCA_RS06340	SE1039_RS10685	SEQMU2_RS02565	BK815_RS11890	A6V26_RS07450	AA913_RS01155	SXYL_RS02460	BE24_RS09325	SSP_RS02300
DNA-3-methyladenine glycosylase	BEK99_RS05020	SCA_RS06335	SE1039_RS07400	SEQMU2_RS12515	BK815_RS02045	A6V26_RS10325	AA913_RS05465	SXYL_RS05935	BE24_RS05705	SSP_RS05630
valine--tRNA ligase	BEK99_RS05025	SCA_RS06330	SE1039_RS07395	SEQMU2_RS12510	BK815_RS02050	A6V26_RS10320	AA913_RS05460	SXYL_RS05940	BE24_RS05700	SSP_RS05635
hypothetical protein	BEK99_RS05040	SCA_RS06315	SE1039_RS07375	SEQMU2_RS12490	BK815_RS02065	A6V26_RS10305	AA913_RS05445	SXYL_RS05960	BE24_RS05680	SSP_RS05655
DUF4930 domain-containing protein	BEK99_RS05045	SCA_RS06305	SE1039_RS07360	SEQMU2_RS12465	BK815_RS02130	A6V26_RS10250	AA913_RS05390	SXYL_RS05970	BE24_RS05670	SSP_RS05665
rod shape-determining protein MreC	BEK99_RS05050	SCA_RS06300	SE1039_RS07355	SEQMU2_RS12460	BK815_RS02135	A6V26_RS10245	AA913_RS05385	SXYL_RS05975	BE24_RS05665	SSP_RS05670

Product	*S. carnosus*		*S. equorum*		*S. succinus*			*S. xylosus*		*S. saprophyticus*
	**JCM 6069**	**TM300**	**KS1039**	**Mu2**	**14BME20**	**CSM 77**	**DSM 14617**	**C2a**	**HKUOPL8**	**ATCC 15305**
rod shape-determining protein MreD	BEK99_RS05055	SCA_RS06295	SE1039_RS07350	SEQMU2_RS12455	BK815_RS02140	A6V26_RS10240	AA913_RS05380	SXYL_RS05980	BE24_RS05660	SSP_RS05675
hypothetical protein	BEK99_RS05065	SCA_RS06285	SE1039_RS07340	SEQMU2_RS12445	BK815_RS02150	A6V26_RS10230	AA913_RS05370	SXYL_RS05990	BE24_RS05650	SSP_RS05685
50S ribosomal protein L27	BEK99_RS05070	SCA_RS06280	SE1039_RS07335	SEQMU2_RS12440	BK815_RS02155	A6V26_RS10225	AA913_RS05365	SXYL_RS05995	BE24_RS05645	SSP_RS05690
GTPase CgtA	BEK99_RS05075	SCA_RS06275	SE1039_RS07330	SEQMU2_RS12435	BK815_RS02160	A6V26_RS10220	AA913_RS05360	SXYL_RS06000	BE24_RS05640	SSP_RS05695
hypothetical protein	BEK99_RS05080	SCA_RS06270	SE1039_RS07325	SEQMU2_RS12430	BK815_RS02165	A6V26_RS10215	AA913_RS05355	SXYL_RS06005	BE24_RS05635	SSP_RS05700
Holliday junction branch migration protein RuvA	BEK99_RS05085	SCA_RS06265	SE1039_RS07320	SEQMU2_RS12425	BK815_RS02170	A6V26_RS10210	AA913_RS05350	SXYL_RS06010	BE24_RS05630	SSP_RS05705
Holliday junction branch migration DNA helicase RuvB	BEK99_RS05090	SCA_RS06260	SE1039_RS07315	SEQMU2_RS12420	BK815_RS02175	A6V26_RS10205	AA913_RS05345	SXYL_RS06015	BE24_RS05625	SSP_RS05710
tRNA preQ1(34) S-adenosylmethionine ribosyltransferase-isomerase QueA	BEK99_RS05095	SCA_RS06255	SE1039_RS07310	SEQMU2_RS12415	BK815_RS02180	A6V26_RS10200	AA913_RS05340	SXYL_RS06020	BE24_RS05620	SSP_RS05715
tRNA guanosine(34) transglycosylase Tgt	BEK99_RS05100	SCA_RS06250	SE1039_RS07305	SEQMU2_RS12410	BK815_RS02185	A6V26_RS10195	AA913_RS05335	SXYL_RS06025	BE24_RS05615	SSP_RS05720
preprotein translocase subunit YajC	BEK99_RS05105	SCA_RS06245	SE1039_RS07300	SEQMU2_RS12405	BK815_RS02190	A6V26_RS10190	AA913_RS05330	SXYL_RS06030	BE24_RS05610	SSP_RS05725
protein translocase subunit SecDF	BEK99_RS05110	SCA_RS06240	SE1039_RS07295	SEQMU2_RS12400	BK815_RS02195	A6V26_RS10185	AA913_RS05325	SXYL_RS06035	BE24_RS05605	SSP_RS05730
single-stranded-DNA-specific exonuclease RecJ	BEK99_RS05115	SCA_RS06235	SE1039_RS07290	SEQMU2_RS12395	BK815_RS02200	A6V26_RS10180	AA913_RS05320	SXYL_RS06040	BE24_RS05600	SSP_RS05735
adenine phosphoribosyltransferase	BEK99_RS05120	SCA_RS06230	SE1039_RS07285	SEQMU2_RS12390	BK815_RS02205	A6V26_RS10175	AA913_RS05315	SXYL_RS06045	BE24_RS05595	SSP_RS05740
bifunctional (p)ppGpp synthetase/guanosine-3,5-bis(diphosphate) 3-pyrophosphohydrolase	BEK99_RS05125	SCA_RS06225	SE1039_RS07280	SEQMU2_RS12385	BK815_RS02210	A6V26_RS10170	AA913_RS05310	SXYL_RS06050	BE24_RS05590	SSP_RS05745
D-tyrosyl-tRNA(Tyr) deacylase	BEK99_RS05130	SCA_RS06220	SE1039_RS07275	SEQMU2_RS12380	BK815_RS02215	A6V26_RS10165	AA913_RS05305	SXYL_RS06055	BE24_RS05585	SSP_RS05750
cell wall amidase	BEK99_RS05135	SCA_RS06215	SE1039_RS07270	SEQMU2_RS12375	BK815_RS02220	A6V26_RS10160	AA913_RS05300	SXYL_RS06060	BE24_RS05580	SSP_RS05755
histidine--tRNA ligase	BEK99_RS05140	SCA_RS06210	SE1039_RS07265	SEQMU2_RS12370	BK815_RS02225	A6V26_RS10155	AA913_RS05295	SXYL_RS06065	BE24_RS05575	SSP_RS05760
aspartate--tRNA ligase	BEK99_RS05145	SCA_RS06205	SE1039_RS07260	SEQMU2_RS12365	BK815_RS02230	A6V26_RS10150	AA913_RS05290	SXYL_RS06070	BE24_RS05570	SSP_RS05765
tRNA threonylcarbamoyladenosine dehydratase	BEK99_RS05155	SCA_RS06200	SE1039_RS07255	SEQMU2_RS12360	BK815_RS02240	A6V26_RS10140	AA913_RS05285	SXYL_RS06075	BE24_RS05565	SSP_RS05770
Rrf2 family transcriptional regulator	BEK99_RS05165	SCA_RS06190	SE1039_RS07245	SEQMU2_RS12350	BK815_RS02250	A6V26_RS10130	AA913_RS05275	SXYL_RS06085	BE24_RS05555	SSP_RS05780
CsbD family protein	BEK99_RS05170	SCA_RS06185	SE1039_RS07240	SEQMU2_RS12345	BK815_RS02255	A6V26_RS10125	AA913_RS05270	SXYL_RS06090	BE24_RS05550	SSP_RS05785
LLM class flavin-dependent oxidoreductase	BEK99_RS05175	SCA_RS06180	SE1039_RS07230	SEQMU2_RS12340	BK815_RS02260	A6V26_RS10120	AA913_RS05265	SXYL_RS06095	BE24_RS05545	SSP_RS05790
cysteine desulfurase	BEK99_RS05180	SCA_RS06175	SE1039_RS07220	SEQMU2_RS12330	BK815_RS02270	A6V26_RS10110	AA913_RS05255	SXYL_RS06105	BE24_RS05535	SSP_RS05805
tRNA 2-thiouridine(34) synthase MnmA	BEK99_RS05185	SCA_RS06170	SE1039_RS07215	SEQMU2_RS12325	BK815_RS02275	A6V26_RS10105	AA913_RS05250	SXYL_RS06110	BE24_RS05530	SSP_RS05810
hypothetical protein	BEK99_RS05190	SCA_RS06165	SE1039_RS07210	SEQMU2_RS12320	BK815_RS02280	A6V26_RS10100	AA913_RS05245	SXYL_RS06115	BE24_RS05525	SSP_RS05815
ATP-dependent RecD-like DNA helicase	BEK99_RS05195	SCA_RS06160	SE1039_RS07205	SEQMU2_RS12315	BK815_RS02285	A6V26_RS10095	AA913_RS05240	SXYL_RS06120	BE24_RS05520	SSP_RS05820
hypothetical protein	BEK99_RS05205	SCA_RS06150	SE1039_RS07195	SEQMU2_RS12305	BK815_RS02295	A6V26_RS10085	AA913_RS05230	SXYL_RS06130	BE24_RS05510	SSP_RS05830
Holliday junction resolvase RuvX	BEK99_RS05210	SCA_RS06145	SE1039_RS07190	SEQMU2_RS12300	BK815_RS02300	A6V26_RS10080	AA913_RS05225	SXYL_RS06135	BE24_RS05505	SSP_RS05835
DUF1292 domain-containing protein	BEK99_RS05215	SCA_RS06140	SE1039_RS07185	SEQMU2_RS12295	BK815_RS02305	A6V26_RS10075	AA913_RS05220	SXYL_RS06140	BE24_RS05500	SSP_RS05840
O-methyltransferase	BEK99_RS05220	SCA_RS06135	SE1039_RS07180	SEQMU2_RS12290	BK815_RS02310	A6V26_RS10070	AA913_RS05215	SXYL_RS06145	BE24_RS05495	SSP_RS05845
uridine kinase	BEK99_RS05235	SCA_RS06120	SE1039_RS07165	SEQMU2_RS12275	BK815_RS02325	A6V26_RS10055	AA913_RS05200	SXYL_RS06160	BE24_RS05480	SSP_RS05860

Product	*S. carnosus*		*S. equorum*		*S. succinus*			*S. xylosus*		*S. saprophyticus*
	**JCM 6069**	**TM300**	**KS1039**	**Mu2**	**14BME20**	**CSM 77**	**DSM 14617**	**C2a**	**HKUOPL8**	**ATCC 15305**
transcription elongation factor GreA	BEK99_RS05240	SCA_RS06115	SE1039_RS07160	SEQMU2_RS12270	BK815_RS02330	A6V26_RS10050	AA913_RS05195	SXYL_RS06165	BE24_RS05475	SSP_RS05865
5-methylthioadenosine/S-adenosylhomocysteine nucleosidase	BEK99_RS05245	SCA_RS06110	SE1039_RS07125	SEQMU2_RS12235	BK815_RS02365	A6V26_RS10015	AA913_RS05160	SXYL_RS06205	BE24_RS05435	SSP_RS05900
hypothetical protein	BEK99_RS05250	SCA_RS06105	SE1039_RS07120	SEQMU2_RS12230	BK815_RS02370	A6V26_RS10010	AA913_RS05155	SXYL_RS06210	BE24_RS05430	SSP_RS05905
ribosome biogenesis GTPase YqeH	BEK99_RS05255	SCA_RS06100	SE1039_RS07115	SEQMU2_RS12225	BK815_RS02375	A6V26_RS10005	AA913_RS05150	SXYL_RS06215	BE24_RS05425	SSP_RS05910
shikimate dehydrogenase	BEK99_RS05260	SCA_RS06095	SE1039_RS07110	SEQMU2_RS12220	BK815_RS02380	A6V26_RS10000	AA913_RS05145	SXYL_RS06220	BE24_RS05420	SSP_RS05915
RNA-binding protein	BEK99_RS05265	SCA_RS06090	SE1039_RS07105	SEQMU2_RS12215	BK815_RS02385	A6V26_RS09995	AA913_RS05140	SXYL_RS06225	BE24_RS05415	SSP_RS05920
nicotinic acid mononucleotide adenylyltransferase	BEK99_RS05270	SCA_RS06085	SE1039_RS07100	SEQMU2_RS12210	BK815_RS02390	A6V26_RS09990	AA913_RS05135	SXYL_RS06230	BE24_RS05410	SSP_RS05925
haloacid dehalogenase	BEK99_RS05275	SCA_RS06080	SE1039_RS07095	SEQMU2_RS12205	BK815_RS02395	A6V26_RS09985	AA913_RS05130	SXYL_RS06235	BE24_RS05405	SSP_RS05930
ribosome silencing factor RsfS	BEK99_RS05280	SCA_RS06075	SE1039_RS07090	SEQMU2_RS12200	BK815_RS02400	A6V26_RS09980	AA913_RS05125	SXYL_RS06240	BE24_RS05400	SSP_RS05935
SAM-dependent methyltransferase	BEK99_RS05285	SCA_RS06070	SE1039_RS07085	SEQMU2_RS12195	BK815_RS02405	A6V26_RS09975	AA913_RS05120	SXYL_RS06245	BE24_RS05395	SSP_RS05940
ComE operon protein 2	BEK99_RS05295	SCA_RS06060	SE1039_RS07075	SEQMU2_RS12185	BK815_RS02415	A6V26_RS09965	AA913_RS05110	SXYL_RS06255	BE24_RS05385	SSP_RS05950
30S ribosomal protein S20	BEK99_RS05310	SCA_RS06045	SE1039_RS07060	SEQMU2_RS12170	BK815_RS02430	A6V26_RS09950	AA913_RS05095	SXYL_RS06270	BE24_RS05360	SSP_RS05965
coproporphyrinogen III oxidase	BEK99_RS05320	SCA_RS06035	SE1039_RS07050	SEQMU2_RS12160	BK815_RS02440	A6V26_RS09940	AA913_RS05085	SXYL_RS06280	BE24_RS05350	SSP_RS05975
heat-inducible transcription repressor HrcA	BEK99_RS05325	SCA_RS06030	SE1039_RS07045	SEQMU2_RS12155	BK815_RS02445	A6V26_RS09935	AA913_RS05080	SXYL_RS06285	BE24_RS05345	SSP_RS05980
nucleotide exchange factor GrpE	BEK99_RS05330	SCA_RS06025	SE1039_RS07040	SEQMU2_RS12150	BK815_RS02450	A6V26_RS09930	AA913_RS05075	SXYL_RS06290	BE24_RS05340	SSP_RS05985
molecular chaperone DnaK	BEK99_RS05335	SCA_RS06020	SE1039_RS07035	SEQMU2_RS12145	BK815_RS02455	A6V26_RS09925	AA913_RS05070	SXYL_RS06295	BE24_RS05335	SSP_RS05990
molecular chaperone DnaJ	BEK99_RS05340	SCA_RS06015	SE1039_RS07030	SEQMU2_RS12140	BK815_RS02460	A6V26_RS09920	AA913_RS05065	SXYL_RS06300	BE24_RS05330	SSP_RS05995
50S ribosomal protein L11 methyltransferase	BEK99_RS05345	SCA_RS06010	SE1039_RS07025	SEQMU2_RS12135	BK815_RS02465	A6V26_RS09915	AA913_RS05060	SXYL_RS06305	BE24_RS05325	SSP_RS06000
16S rRNA (uracil(1498)-N(3))-methyltransferase	BEK99_RS05350	SCA_RS06005	SE1039_RS07020	SEQMU2_RS12130	BK815_RS02470	A6V26_RS09910	AA913_RS05055	SXYL_RS06310	BE24_RS05320	SSP_RS06005
30S ribosomal protein S21	BEK99_RS05360	SCA_RS05995	SE1039_RS07015	SEQMU2_RS12125	BK815_RS02475	A6V26_RS09905	AA913_RS05050	SXYL_RS06315	BE24_RS05035	SSP_RS06010
serine protease	BEK99_RS05365	SCA_RS05990	SE1039_RS07010	SEQMU2_RS12120	BK815_RS02480	A6V26_RS09900	AA913_RS05045	SXYL_RS06320	BE24_RS05030	SSP_RS06015
UPF0365 family protein	BEK99_RS05370	SCA_RS05985	SE1039_RS07005	SEQMU2_RS12115	BK815_RS02485	A6V26_RS09895	AA913_RS05040	SXYL_RS06325	BE24_RS05025	SSP_RS06020
hypothetical protein	BEK99_RS05375	SCA_RS05980	SE1039_RS07000	SEQMU2_RS12110	BK815_RS02490	A6V26_RS09890	AA913_RS05035	SXYL_RS06330	BE24_RS05020	SSP_RS06025
PhoH family protein	BEK99_RS05380	SCA_RS05975	SE1039_RS06995	SEQMU2_RS12105	BK815_RS02495	A6V26_RS09885	AA913_RS05030	SXYL_RS06335	BE24_RS05015	SSP_RS06030
endoribonuclease YbeY	BEK99_RS05385	SCA_RS05970	SE1039_RS06990	SEQMU2_RS12100	BK815_RS02500	A6V26_RS09880	AA913_RS05025	SXYL_RS06340	BE24_RS05010	SSP_RS06035
diacylglycerol kinase	BEK99_RS05390	SCA_RS05965	SE1039_RS06985	SEQMU2_RS12095	BK815_RS02505	A6V26_RS09875	AA913_RS05020	SXYL_RS06345	BE24_RS05005	SSP_RS06040
cytidine deaminase	BEK99_RS05395	SCA_RS05960	SE1039_RS06980	SEQMU2_RS12090	BK815_RS02510	A6V26_RS09870	AA913_RS05015	SXYL_RS06350	BE24_RS05000	SSP_RS06045
GTPase Era	BEK99_RS05400	SCA_RS05955	SE1039_RS06975	SEQMU2_RS12085	BK815_RS02515	A6V26_RS09865	AA913_RS05010	SXYL_RS06355	BE24_RS04995	SSP_RS06050
glycine--tRNA ligase	BEK99_RS05410	SCA_RS05945	SE1039_RS06965	SEQMU2_RS12075	BK815_RS02525	A6V26_RS09855	AA913_RS05000	SXYL_RS06365	BE24_RS04985	SSP_RS06060
hypothetical protein	BEK99_RS05415	SCA_RS05940	SE1039_RS06960	SEQMU2_RS12070	BK815_RS02530	A6V26_RS09850	AA913_RS04995	SXYL_RS06370	BE24_RS04980	SSP_RS06065
phosphoenolpyruvate synthase regulatory protein	BEK99_RS05420	SCA_RS05935	SE1039_RS06955	SEQMU2_RS12065	BK815_RS02535	A6V26_RS09845	AA913_RS04990	SXYL_RS06375	BE24_RS04975	SSP_RS06070

Product	*S. carnosus*		*S. equorum*		*S. succinus*			*S. xylosus*		*S. saprophyticus*
	**JCM 6069**	**TM300**	**KS1039**	**Mu2**	**14BME20**	**CSM 77**	**DSM 14617**	**C2a**	**HKUOPL8**	**ATCC 15305**
DNA primase	BEK99_RS05425	SCA_RS05930	SE1039_RS06950	SEQMU2_RS12060	BK815_RS02540	A6V26_RS09840	AA913_RS04985	SXYL_RS06380	BE24_RS04970	SSP_RS06075
RNA polymerase sigma factor RpoD	BEK99_RS05430	SCA_RS05925	SE1039_RS06945	SEQMU2_RS12055	BK815_RS02545	A6V26_RS09835	AA913_RS04980	SXYL_RS06385	BE24_RS04965	SSP_RS06080
tRNA methyltransferase	BEK99_RS05435	SCA_RS05920	SE1039_RS06940	SEQMU2_RS12050	BK815_RS02550	A6V26_RS09830	AA913_RS04975	SXYL_RS06390	BE24_RS04960	SSP_RS06085
Nif3-like dinuclear metal center hexameric protein	BEK99_RS05440	SCA_RS05915	SE1039_RS06935	SEQMU2_RS12045	BK815_RS02555	A6V26_RS09825	AA913_RS04970	SXYL_RS06395	BE24_RS04955	SSP_RS06090
ATP-dependent helicase	BEK99_RS05445	SCA_RS05910	SE1039_RS06930	SEQMU2_RS12040	BK815_RS02560	A6V26_RS09820	AA913_RS04965	SXYL_RS06400	BE24_RS04950	SSP_RS06095
endonuclease	BEK99_RS05450	SCA_RS05905	SE1039_RS06925	SEQMU2_RS12035	BK815_RS02565	A6V26_RS09815	AA913_RS04960	SXYL_RS06405	BE24_RS04945	SSP_RS06100
zinc ABC transporter ATP-binding protein	BEK99_RS05455	SCA_RS05900	SE1039_RS06920	SEQMU2_RS12030	BK815_RS02570	A6V26_RS09810	AA913_RS04955	SXYL_RS06410	BE24_RS04940	SSP_RS06105
metal ABC transporter permease	BEK99_RS05460	SCA_RS05895	SE1039_RS06915	SEQMU2_RS12025	BK815_RS02575	A6V26_RS09805	AA913_RS04950	SXYL_RS06415	BE24_RS04935	SSP_RS06110
transcriptional repressor	BEK99_RS05465	SCA_RS05890	SE1039_RS06910	SEQMU2_RS12020	BK815_RS02580	A6V26_RS09800	AA913_RS04945	SXYL_RS06420	BE24_RS04930	SSP_RS06115
superoxide dismutase [Mn/Fe]	BEK99_RS05470	SCA_RS05885	SE1039_RS06905	SEQMU2_RS12015	BK815_RS02585	A6V26_RS09795	AA913_RS04940	SXYL_RS06425	BE24_RS04925	SSP_RS06120
penicillin-binding protein	BEK99_RS05475	SCA_RS05880	SE1039_RS06900	SEQMU2_RS12010	BK815_RS02590	A6V26_RS09790	AA913_RS04935	SXYL_RS06430	BE24_RS04920	SSP_RS06125
5-formyltetrahydrofolate cyclo-ligase	BEK99_RS05480	SCA_RS05875	SE1039_RS06890	SEQMU2_RS12000	BK815_RS02600	A6V26_RS09780	AA913_RS04925	SXYL_RS06440	BE24_RS04910	SSP_RS06135
hypothetical protein	BEK99_RS05490	SCA_RS05865	SE1039_RS06880	SEQMU2_RS11990	BK815_RS02610	A6V26_RS09770	AA913_RS04915	SXYL_RS06450	BE24_RS04900	SSP_RS06145
glucokinase	BEK99_RS05495	SCA_RS05860	SE1039_RS06875	SEQMU2_RS11985	BK815_RS02625	A6V26_RS09765	AA913_RS04910	SXYL_RS06455	BE24_RS04895	SSP_RS06150
hypothetical protein	BEK99_RS05500	SCA_RS05855	SE1039_RS06870	SEQMU2_RS11980	BK815_RS02630	A6V26_RS09760	AA913_RS04905	SXYL_RS06460	BE24_RS04890	SSP_RS06155
MBL fold metallo-hydrolase	BEK99_RS05505	SCA_RS05850	SE1039_RS06865	SEQMU2_RS11975	BK815_RS02635	A6V26_RS09755	AA913_RS04900	SXYL_RS06465	BE24_RS04885	SSP_RS06160
glycine cleavage system protein T	BEK99_RS05545	SCA_RS05810	SE1039_RS06820	SEQMU2_RS11930	BK815_RS02985	A6V26_RS09710	AA913_RS04855	SXYL_RS06510	BE24_RS04840	SSP_RS06205
rhodanese	BEK99_RS05560	SCA_RS05795	SE1039_RS06805	SEQMU2_RS11915	BK815_RS03000	A6V26_RS09695	AA913_RS04840	SXYL_RS06525	BE24_RS04825	SSP_RS06220
biotin/lipoate A/B protein ligase family protein	BEK99_RS05565	SCA_RS05790	SE1039_RS06800	SEQMU2_RS11910	BK815_RS03005	A6V26_RS09690	AA913_RS04835	SXYL_RS06530	BE24_RS04820	SSP_RS06225
hypothetical protein	BEK99_RS05570	SCA_RS05785	SE1039_RS06795	SEQMU2_RS11905	BK815_RS03010	A6V26_RS09685	AA913_RS04830	SXYL_RS06535	BE24_RS04815	SSP_RS06230
peptidase M24 family protein	BEK99_RS05580	SCA_RS05775	SE1039_RS06785	SEQMU2_RS11895	BK815_RS03020	A6V26_RS09675	AA913_RS04820	SXYL_RS06545	BE24_RS04805	SSP_RS06240
elongation factor P	BEK99_RS05585	SCA_RS05770	SE1039_RS06780	SEQMU2_RS11890	BK815_RS03025	A6V26_RS09670	AA913_RS04815	SXYL_RS06550	BE24_RS04800	SSP_RS06245
acetyl-CoA carboxylase biotin carboxylase subunit	BEK99_RS05595	SCA_RS05760	SE1039_RS06770	SEQMU2_RS11880	BK815_RS03035	A6V26_RS09660	AA913_RS04805	SXYL_RS06560	BE24_RS04790	SSP_RS06255
Asp23/Gls24 family envelope stress response protein	BEK99_RS05600	SCA_RS05755	SE1039_RS06765	SEQMU2_RS11875	BK815_RS03040	A6V26_RS09655	AA913_RS04800	SXYL_RS06565	BE24_RS04785	SSP_RS06260
N utilization substance protein B	BEK99_RS05605	SCA_RS05750	SE1039_RS06760	SEQMU2_RS11870	BK815_RS03045	A6V26_RS09650	AA913_RS04795	SXYL_RS06570	BE24_RS04780	SSP_RS06265
exodeoxyribonuclease VII large subunit	BEK99_RS05610	SCA_RS05745	SE1039_RS06755	SEQMU2_RS11865	BK815_RS03050	A6V26_RS09645	AA913_RS04790	SXYL_RS06575	BE24_RS04775	SSP_RS06270
geranyl transferase	BEK99_RS05620	SCA_RS05735	SE1039_RS06745	SEQMU2_RS11855	BK815_RS03060	A6V26_RS09635	AA913_RS04780	SXYL_RS06585	BE24_RS04765	SSP_RS06280
arginine repressor	BEK99_RS05625	SCA_RS05730	SE1039_RS06740	SEQMU2_RS11850	BK815_RS03065	A6V26_RS09630	AA913_RS04775	SXYL_RS06590	BE24_RS04760	SSP_RS06285
DNA repair protein RecN	BEK99_RS05630	SCA_RS05725	SE1039_RS06735	SEQMU2_RS11845	BK815_RS03070	A6V26_RS09625	AA913_RS04770	SXYL_RS06595	BE24_RS04755	SSP_RS06290
dihydrolipoyl dehydrogenase	BEK99_RS05645	SCA_RS05710	SE1039_RS06720	SEQMU2_RS11830	BK815_RS03080	A6V26_RS09615	AA913_RS04760	SXYL_RS06610	BE24_RS04740	SSP_RS06300
2-oxoisovalerate dehydrogenase subunit alpha	BEK99_RS05650	SCA_RS05705	SE1039_RS06715	SEQMU2_RS11825	BK815_RS03085	A6V26_RS09610	AA913_RS04755	SXYL_RS06615	BE24_RS04735	SSP_RS06305

Product	*S. carnosus*		*S. equorum*		*S. succinus*			*S. xylosus*		*S. saprophyticus*
	**JCM 6069**	**TM300**	**KS1039**	**Mu2**	**14BME20**	**CSM 77**	**DSM 14617**	**C2a**	**HKUOPL8**	**ATCC 15305**
alpha-ketoacid dehydrogenase subunit beta	BEK99_RS05655	SCA_RS05700	SE1039_RS06710	SEQMU2_RS11820	BK815_RS03090	A6V26_RS09605	AA913_RS04750	SXYL_RS06620	BE24_RS04730	SSP_RS06310
2-oxoglutarate dehydrogenase	BEK99_RS05660	SCA_RS05695	SE1039_RS06705	SEQMU2_RS11815	BK815_RS03095	A6V26_RS09600	AA913_RS04745	SXYL_RS06625	BE24_RS04725	SSP_RS06315
hypothetical protein	BEK99_RS05665	SCA_RS05690	SE1039_RS06700	SEQMU2_RS11810	BK815_RS03100	A6V26_RS09595	AA913_RS04740	SXYL_RS06640	BE24_RS04720	SSP_RS06320
membrane protein	BEK99_RS05670	SCA_RS05685	SE1039_RS06695	SEQMU2_RS11805	BK815_RS03105	A6V26_RS09590	AA913_RS04735	SXYL_RS06645	BE24_RS04715	SSP_RS06325
hypothetical protein	BEK99_RS05680	SCA_RS05675	SE1039_RS06690	SEQMU2_RS11800	BK815_RS03110	A6V26_RS09585	AA913_RS04730	SXYL_RS06650	BE24_RS04710	SSP_RS06330
NADP-dependent 6-phosphogluconate dehydrogenase	BEK99_RS05685	SCA_RS05670	SE1039_RS06685	SEQMU2_RS11795	BK815_RS03115	A6V26_RS09580	AA913_RS04725	SXYL_RS06655	BE24_RS04705	SSP_RS06335
AraC family transcriptional regulator	BEK99_RS05690	SCA_RS05665	SE1039_RS06670	SEQMU2_RS11780	BK815_RS03130	A6V26_RS09565	AA913_RS04710	SXYL_RS06670	BE24_RS04690	SSP_RS06350
glucose-6-phosphate dehydrogenase	BEK99_RS05695	SCA_RS05660	SE1039_RS06665	SEQMU2_RS11775	BK815_RS03135	A6V26_RS09560	AA913_RS04705	SXYL_RS06675	BE24_RS04685	SSP_RS06355
ribonuclease Z	BEK99_RS05700	SCA_RS05655	SE1039_RS06660	SEQMU2_RS11770	BK815_RS03140	A6V26_RS09555	AA913_RS04700	SXYL_RS06680	BE24_RS04680	SSP_RS06360
NUDIX hydrolase	BEK99_RS05720	SCA_RS05635	SE1039_RS06640	SEQMU2_RS11750	BK815_RS03160	A6V26_RS09535	AA913_RS04680	SXYL_RS06700	BE24_RS04660	SSP_RS06380
transcriptional repressor	BEK99_RS05725	SCA_RS05630	SE1039_RS06635	SEQMU2_RS11745	BK815_RS03165	A6V26_RS09530	AA913_RS04675	SXYL_RS06705	BE24_RS04655	SSP_RS06385
site-specific tyrosine recombinase XerD	BEK99_RS05730	SCA_RS05625	SE1039_RS06630	SEQMU2_RS11740	BK815_RS03170	A6V26_RS09525	AA913_RS04670	SXYL_RS06710	BE24_RS04650	SSP_RS06390
hypothetical protein	BEK99_RS05735	SCA_RS05620	SE1039_RS06625	SEQMU2_RS11735	BK815_RS03175	A6V26_RS09520	AA913_RS04665	SXYL_RS06715	BE24_RS04645	SSP_RS06395
segregation and condensation protein A	BEK99_RS05740	SCA_RS05615	SE1039_RS06620	SEQMU2_RS11730	BK815_RS03180	A6V26_RS09515	AA913_RS04660	SXYL_RS06720	BE24_RS04640	SSP_RS06400
SMC-Scp complex subunit ScpB	BEK99_RS05745	SCA_RS05610	SE1039_RS06615	SEQMU2_RS11725	BK815_RS03185	A6V26_RS09510	AA913_RS04655	SXYL_RS06725	BE24_RS04635	SSP_RS06405
rRNA pseudouridine synthase	BEK99_RS05750	SCA_RS05605	SE1039_RS06610	SEQMU2_RS11720	BK815_RS03190	A6V26_RS09505	AA913_RS04650	SXYL_RS06730	BE24_RS04630	SSP_RS06410
DNA-binding response regulator	BEK99_RS05755	SCA_RS05600	SE1039_RS06605	SEQMU2_RS11715	BK815_RS03195	A6V26_RS09500	AA913_RS04645	SXYL_RS06735	BE24_RS04625	SSP_RS06415
sensor histidine kinase	BEK99_RS05760	SCA_RS05595	SE1039_RS06600	SEQMU2_RS11710	BK815_RS03200	A6V26_RS09495	AA913_RS04640	SXYL_RS06740	BE24_RS04620	SSP_RS06420
hypothetical protein	BEK99_RS05765	SCA_RS05590	SE1039_RS06595	SEQMU2_RS11705	BK815_RS03205	A6V26_RS09490	AA913_RS04635	SXYL_RS06745	BE24_RS04615	SSP_RS06425
ferredoxin	BEK99_RS05770	SCA_RS05585	SE1039_RS06590	SEQMU2_RS11700	BK815_RS03210	A6V26_RS09485	AA913_RS04630	SXYL_RS06750	BE24_RS04610	SSP_RS06430
ATP-dependent DNA helicase RecQ	BEK99_RS05780	SCA_RS05575	SE1039_RS06580	SEQMU2_RS11690	BK815_RS03220	A6V26_RS09475	AA913_RS04620	SXYL_RS06760	BE24_RS04600	SSP_RS06440
thioredoxin reductase	BEK99_RS05790	SCA_RS05565	SE1039_RS06570	SEQMU2_RS11680	BK815_RS03230	A6V26_RS09465	AA913_RS04610	SXYL_RS06770	BE24_RS04590	SSP_RS06450
L-asparaginase	BEK99_RS05795	SCA_RS05560	SE1039_RS06565	SEQMU2_RS11675	BK815_RS03235	A6V26_RS09460	AA913_RS04605	SXYL_RS06775	BE24_RS04585	SSP_RS06455
cytidylate kinase	BEK99_RS05800	SCA_RS05555	SE1039_RS06560	SEQMU2_RS11670	BK815_RS03240	A6V26_RS09455	AA913_RS04600	SXYL_RS06780	BE24_RS04580	SSP_RS06460
30S ribosomal protein S1	BEK99_RS05805	SCA_RS05550	SE1039_RS06555	SEQMU2_RS11665	BK815_RS03245	A6V26_RS09450	AA913_RS04595	SXYL_RS06785	BE24_RS04575	SSP_RS06465
ribosome biogenesis GTPase Der	BEK99_RS05810	SCA_RS05545	SE1039_RS06550	SEQMU2_RS11660	BK815_RS03250	A6V26_RS09445	AA913_RS04590	SXYL_RS06790	BE24_RS04570	SSP_RS06470
glycerol-3-phosphate dehydrogenase (NAD(P)(+))	BEK99_RS05815	SCA_RS05540	SE1039_RS06545	SEQMU2_RS11655	BK815_RS03255	A6V26_RS09440	AA913_RS04585	SXYL_RS06795	BE24_RS04565	SSP_RS06475
HU family DNA-binding protein	BEK99_RS05820	SCA_RS05535	SE1039_RS06540	SEQMU2_RS11650	BK815_RS03260	A6V26_RS09435	AA913_RS04580	SXYL_RS06800	BE24_RS04560	SSP_RS06480
heptaprenyl pyrophosphate synthase subunit A	BEK99_RS05825	SCA_RS05530	SE1039_RS06535	SEQMU2_RS11645	BK815_RS03265	A6V26_RS09430	AA913_RS04575	SXYL_RS06805	BE24_RS04555	SSP_RS06485
bifunctional demethylmenaquinone methyltransferase/2-methoxy-6-polyprenyl-1,4-benzoquinol methylase	BEK99_RS05830	SCA_RS05525	SE1039_RS06530	SEQMU2_RS11640	BK815_RS03270	A6V26_RS09425	AA913_RS04570	SXYL_RS06810	BE24_RS04550	SSP_RS06490
heptaprenyl diphosphate synthase subunit II	BEK99_RS05835	SCA_RS05520	SE1039_RS06525	SEQMU2_RS11635	BK815_RS03275	A6V26_RS09420	AA913_RS04565	SXYL_RS06815	BE24_RS04545	SSP_RS06495

Product	*S. carnosus*		*S. equorum*		*S. succinus*			*S. xylosus*		*S. saprophyticus*
	**JCM 6069**	**TM300**	**KS1039**	**Mu2**	**14BME20**	**CSM 77**	**DSM 14617**	**C2a**	**HKUOPL8**	**ATCC 15305**
nucleoside-diphosphate kinase	BEK99_RS05840	SCA_RS05515	SE1039_RS06520	SEQMU2_RS11630	BK815_RS03280	A6V26_RS09415	AA913_RS04560	SXYL_RS06820	BE24_RS04540	SSP_RS06500
chorismate synthase	BEK99_RS05845	SCA_RS05510	SE1039_RS06515	SEQMU2_RS11625	BK815_RS03285	A6V26_RS09410	AA913_RS04555	SXYL_RS06825	BE24_RS04535	SSP_RS06505
3-dehydroquinate synthase	BEK99_RS05850	SCA_RS05505	SE1039_RS06510	SEQMU2_RS11620	BK815_RS03290	A6V26_RS09405	AA913_RS09245	SXYL_RS06830	BE24_RS04530	SSP_RS06510
3-phosphoshikimate 1-carboxyvinyltransferase	BEK99_RS05855	SCA_RS05500	SE1039_RS06505	SEQMU2_RS11615	BK815_RS03295	A6V26_RS09400	AA913_RS09250	SXYL_RS06835	BE24_RS04525	SSP_RS06515
hypothetical protein	BEK99_RS05860	SCA_RS05495	SE1039_RS06500	SEQMU2_RS11610	BK815_RS03300	A6V26_RS09395	AA913_RS09255	SXYL_RS06840	BE24_RS04520	SSP_RS06520
hypothetical protein	BEK99_RS05865	SCA_RS05490	SE1039_RS06495	SEQMU2_RS11605	BK815_RS03305	A6V26_RS09390	AA913_RS09260	SXYL_RS06845	BE24_RS04515	SSP_RS06525
hypothetical protein	BEK99_RS05880	SCA_RS05475	SE1039_RS06480	SEQMU2_RS11590	BK815_RS03320	A6V26_RS09375	AA913_RS09275	SXYL_RS06860	BE24_RS04500	SSP_RS06540
N-acetyl-alpha-D-glucosaminyl L-malate synthase BshA	BEK99_RS05885	SCA_RS05470	SE1039_RS06475	SEQMU2_RS11585	BK815_RS03325	A6V26_RS09370	AA913_RS09280	SXYL_RS06865	BE24_RS04495	SSP_RS06545
[cytidine(C)-cytidine(C)-adenosine (A)]-adding enzyme	BEK99_RS05890	SCA_RS05465	SE1039_RS06470	SEQMU2_RS11580	BK815_RS03330	A6V26_RS09365	AA913_RS09285	SXYL_RS06870	BE24_RS04490	SSP_RS06550
biotin--[acetyl-CoA-carboxylase] ligase	BEK99_RS05895	SCA_RS05460	SE1039_RS06465	SEQMU2_RS11575	BK815_RS03335	A6V26_RS09360	AA913_RS09290	SXYL_RS06875	BE24_RS04485	SSP_RS06555
ATP-dependent helicase	BEK99_RS05900	SCA_RS05455	SE1039_RS06460	SEQMU2_RS11570	BK815_RS03340	A6V26_RS09355	AA913_RS09295	SXYL_RS06880	BE24_RS04480	SSP_RS06560
asparagine--tRNA ligase	BEK99_RS05905	SCA_RS05450	SE1039_RS06455	SEQMU2_RS11565	BK815_RS03345	A6V26_RS09350	AA913_RS09300	SXYL_RS06885	BE24_RS04475	SSP_RS06565
DNA replication protein DnaD	BEK99_RS05910	SCA_RS05445	SE1039_RS06450	SEQMU2_RS11560	BK815_RS03350	A6V26_RS09345	AA913_RS09305	SXYL_RS06890	BE24_RS04470	SSP_RS06570
endonuclease III	BEK99_RS05915	SCA_RS05440	SE1039_RS06445	SEQMU2_RS11555	BK815_RS03355	A6V26_RS09340	AA913_RS09310	SXYL_RS06895	BE24_RS04465	SSP_RS06575
hypothetical protein	BEK99_RS05920	SCA_RS05435	SE1039_RS06440	SEQMU2_RS11550	BK815_RS03360	A6V26_RS09335	AA913_RS09315	SXYL_RS06900	BE24_RS04460	SSP_RS06580
transglycosylase	BEK99_RS05925	SCA_RS05430	SE1039_RS06435	SEQMU2_RS11545	BK815_RS03365	A6V26_RS09330	AA913_RS09320	SXYL_RS06905	BE24_RS04455	SSP_RS06585
Holliday junction resolvase RecU	BEK99_RS05930	SCA_RS05425	SE1039_RS06430	SEQMU2_RS11540	BK815_RS03370	A6V26_RS09325	AA913_RS09325	SXYL_RS06910	BE24_RS04450	SSP_RS06590
hypothetical protein	BEK99_RS05935	SCA_RS05420	SE1039_RS06425	SEQMU2_RS11535	BK815_RS03375	A6V26_RS09320	AA913_RS09330	SXYL_RS06915	BE24_RS04445	SSP_RS06595
hypothetical protein	BEK99_RS05940	SCA_RS05415	SE1039_RS06420	SEQMU2_RS11530	BK815_RS03380	A6V26_RS09315	AA913_RS09335	SXYL_RS06920	BE24_RS04440	SSP_RS06600
cell division protein GpsB	BEK99_RS05945	SCA_RS05410	SE1039_RS06415	SEQMU2_RS11525	BK815_RS03385	A6V26_RS09310	AA913_RS09340	SXYL_RS06925	BE24_RS04435	SSP_RS06605
RNA methyltransferase	BEK99_RS05955	SCA_RS05405	SE1039_RS06410	SEQMU2_RS11520	BK815_RS03395	A6V26_RS09300	AA913_RS09345	SXYL_RS06930	BE24_RS04430	SSP_RS06610
protein RarD	BEK99_RS05960	SCA_RS05400	SE1039_RS06405	SEQMU2_RS11515	BK815_RS03400	A6V26_RS09295	AA913_RS09350	SXYL_RS06935	BE24_RS04425	SSP_RS06615
5-3 exonuclease	BEK99_RS05970	SCA_RS05390	SE1039_RS06390	SEQMU2_RS11500	BK815_RS03415	A6V26_RS09280	AA913_RS09365	SXYL_RS06950	BE24_RS04410	SSP_RS06635
ribonuclease H	BEK99_RS05975	SCA_RS05385	SE1039_RS06385	SEQMU2_RS11495	BK815_RS03420	A6V26_RS09275	AA913_RS09370	SXYL_RS06955	BE24_RS04405	SSP_RS06640
membrane protein	BEK99_RS05980	SCA_RS05380	SE1039_RS06375	SEQMU2_RS11485	BK815_RS03430	A6V26_RS09265	AA913_RS09380	SXYL_RS06965	BE24_RS04395	SSP_RS06650
hypothetical protein	BEK99_RS13060	SCA_RS12660	SE1039_RS06370	SEQMU2_RS11480	BK815_RS03435	A6V26_RS09260	AA913_RS09385	SXYL_RS06970	BE24_RS04390	SSP_RS06655
virulence factor	BEK99_RS05985	SCA_RS05375	SE1039_RS06360	SEQMU2_RS11470	BK815_RS03445	A6V26_RS09250	AA913_RS09395	SXYL_RS06980	BE24_RS04380	SSP_RS06665
hypothetical protein	BEK99_RS05990	SCA_RS05370	SE1039_RS06355	SEQMU2_RS11465	BK815_RS03450	A6V26_RS09245	AA913_RS09400	SXYL_RS06985	BE24_RS04375	SSP_RS06670
thymidylate synthase	BEK99_RS05995	SCA_RS05365	SE1039_RS06350	SEQMU2_RS11460	BK815_RS03455	A6V26_RS09240	AA913_RS09405	SXYL_RS06990	BE24_RS04370	SSP_RS06675
dihydrofolate reductase	BEK99_RS06000	SCA_RS05360	SE1039_RS06345	SEQMU2_RS11455	BK815_RS03460	A6V26_RS09235	AA913_RS09410	SXYL_RS06995	BE24_RS04365	SSP_RS06680
peptide-methionine (S)-S-oxide reductase	BEK99_RS06010	SCA_RS05350	SE1039_RS06335	SEQMU2_RS11445	BK815_RS03470	A6V26_RS09225	AA913_RS09420	SXYL_RS07005	BE24_RS04355	SSP_RS06690

**Product**	***S. carnosus***		***S. equorum***		***S. succinus***			***S. xylosus***		***S. saprophyticus***
	**JCM 6069**	**TM300**	**KS1039**	**Mu2**	**14BME20**	**CSM 77**	**DSM 14617**	**C2a**	**HKUOPL8**	**ATCC 15305**
peptide-methionine (R)-S-oxide reductase	BEK99_RS06015	SCA_RS05345	SE1039_RS06330	SEQMU2_RS11440	BK815_RS03475	A6V26_RS09220	AA913_RS09425	SXYL_RS07010	BE24_RS04350	SSP_RS06695
PTS glucose transporter subunit IIA	BEK99_RS06020	SCA_RS05340	SE1039_RS06325	SEQMU2_RS11435	BK815_RS03480	A6V26_RS09215	AA913_RS09430	SXYL_RS07015	BE24_RS04345	SSP_RS06700
YozE family protein	BEK99_RS06025	SCA_RS05335	SE1039_RS06320	SEQMU2_RS11430	BK815_RS03485	A6V26_RS09210	AA913_RS09435	SXYL_RS07020	BE24_RS04340	SSP_RS06705
serine protease	BEK99_RS06030	SCA_RS05330	SE1039_RS06315	SEQMU2_RS11425	BK815_RS03490	A6V26_RS09205	AA913_RS09440	SXYL_RS07025	BE24_RS04335	SSP_RS06710
N-acetyltransferase	BEK99_RS06035	SCA_RS05325	SE1039_RS06310	SEQMU2_RS11420	BK815_RS03495	A6V26_RS09200	AA913_RS09445	SXYL_RS07030	BE24_RS04330	SSP_RS06715
undecaprenyldiphospho-muramoylpentapeptide beta-N-acetylglucosaminyltransferase	BEK99_RS06040	SCA_RS05320	SE1039_RS06305	SEQMU2_RS11415	BK815_RS03500	A6V26_RS09195	AA913_RS09450	SXYL_RS07035	BE24_RS04325	SSP_RS06720
phosphatase PAP2 family protein	BEK99_RS06045	SCA_RS05315	SE1039_RS06300	SEQMU2_RS11410	BK815_RS03505	A6V26_RS09190	AA913_RS09455	SXYL_RS07040	BE24_RS04320	SSP_RS06725
DNA-binding response regulator	BEK99_RS06050	SCA_RS05310	SE1039_RS06295	SEQMU2_RS11405	BK815_RS03510	A6V26_RS09185	AA913_RS09460	SXYL_RS07045	BE24_RS04315	SSP_RS06730
sensor histidine kinase	BEK99_RS06055	SCA_RS05305	SE1039_RS06290	SEQMU2_RS11400	BK815_RS03515	A6V26_RS09180	AA913_RS09465	SXYL_RS07050	BE24_RS04310	SSP_RS06735
2-oxoglutarate dehydrogenase E1 component	BEK99_RS06060	SCA_RS05300	SE1039_RS06285	SEQMU2_RS11395	BK815_RS03520	A6V26_RS09175	AA913_RS09470	SXYL_RS07055	BE24_RS04305	SSP_RS06740
dihydrolipoamide succinyltransferase	BEK99_RS06065	SCA_RS05295	SE1039_RS06280	SEQMU2_RS11390	BK815_RS03525	A6V26_RS09170	AA913_RS09475	SXYL_RS07060	BE24_RS04300	SSP_RS06745
hypothetical protein	BEK99_RS06070	SCA_RS05290	SE1039_RS06225	SEQMU2_RS11275	BK815_RS03570	A6V26_RS09125	AA913_RS09520	SXYL_RS07195	BE24_RS04240	SSP_RS06805
hypothetical protein	BEK99_RS06075	SCA_RS05285	SE1039_RS06220	SEQMU2_RS11270	BK815_RS03575	A6V26_RS09120	AA913_RS09525	SXYL_RS07200	BE24_RS04235	SSP_RS06810
hypothetical protein	BEK99_RS06080	SCA_RS05280	SE1039_RS06215	SEQMU2_RS11265	BK815_RS03580	A6V26_RS09115	AA913_RS09530	SXYL_RS07205	BE24_RS04230	SSP_RS06815
MoxR family ATPase	BEK99_RS06085	SCA_RS05275	SE1039_RS06210	SEQMU2_RS11260	BK815_RS03585	A6V26_RS09110	AA913_RS09535	SXYL_RS07210	BE24_RS04225	SSP_RS06820
hypothetical protein	BEK99_RS06090	SCA_RS05270	SE1039_RS06205	SEQMU2_RS11255	BK815_RS03590	A6V26_RS09105	AA913_RS09540	SXYL_RS07215	BE24_RS04220	SSP_RS06825
branched-chain amino acid transport system II carrier protein	BEK99_RS06095	SCA_RS05265	SE1039_RS06200	SEQMU2_RS11250	BK815_RS03595	A6V26_RS09100	AA913_RS09545	SXYL_RS07220	BE24_RS04215	SSP_RS06830
lipid kinase	BEK99_RS06100	SCA_RS05260	SE1039_RS02795	SEQMU2_RS07585	BK815_RS06700	A6V26_RS02825	AA913_RS08105	SXYL_RS10395	BE24_RS01340	SSP_RS09985
toxic anion resistance protein	BEK99_RS06105	SCA_RS05255	SE1039_RS06190	SEQMU2_RS11240	BK815_RS03600	A6V26_RS09095	AA913_RS09550	SXYL_RS07230	BE24_RS04205	SSP_RS06840
5-bromo-4-chloroindolyl phosphate hydrolase	BEK99_RS06110	SCA_RS05250	SE1039_RS06185	SEQMU2_RS11235	BK815_RS03605	A6V26_RS09090	AA913_RS09555	SXYL_RS07235	BE24_RS04200	SSP_RS06845
acylphosphatase	BEK99_RS06115	SCA_RS05245	SE1039_RS06180	SEQMU2_RS11230	BK815_RS03610	A6V26_RS09085	AA913_RS09560	SXYL_RS07240	BE24_RS04195	SSP_RS06850
hypothetical protein	BEK99_RS06120	SCA_RS05240	SE1039_RS06175	SEQMU2_RS11225	BK815_RS03615	A6V26_RS09080	AA913_RS09565	SXYL_RS07245	BE24_RS04190	SSP_RS06855
cold-shock protein CspA	BEK99_RS06125	SCA_RS05235	SE1039_RS06170	SEQMU2_RS11220	BK815_RS03620	A6V26_RS09075	AA913_RS09570	SXYL_RS07250	BE24_RS04185	SSP_RS06860
diaminopimelate decarboxylase	BEK99_RS06130	SCA_RS05230	SE1039_RS06165	SEQMU2_RS11215	BK815_RS03625	A6V26_RS09070	AA913_RS09575	SXYL_RS07255	BE24_RS04180	SSP_RS06865
alanine racemase	BEK99_RS06135	SCA_RS05225	SE1039_RS06160	SEQMU2_RS11210	BK815_RS03630	A6V26_RS09065	AA913_RS09580	SXYL_RS07260	BE24_RS04175	SSP_RS06870
amidohydrolase	BEK99_RS06140	SCA_RS05220	SE1039_RS06155	SEQMU2_RS11205	BK815_RS03635	A6V26_RS09060	AA913_RS09585	SXYL_RS07265	BE24_RS04170	SSP_RS06875
2,3,4,5-tetrahydropyridine-2,6-dicarboxylate N-acetyltransferase	BEK99_RS06145	SCA_RS05215	SE1039_RS06150	SEQMU2_RS11200	BK815_RS03640	A6V26_RS09055	AA913_RS09590	SXYL_RS07270	BE24_RS04165	SSP_RS06880
4-hydroxy-tetrahydrodipicolinate reductase	BEK99_RS06150	SCA_RS05210	SE1039_RS06145	SEQMU2_RS11195	BK815_RS03645	A6V26_RS09050	AA913_RS09595	SXYL_RS07275	BE24_RS04160	SSP_RS06885
4-hydroxy-tetrahydrodipicolinate synthase	BEK99_RS06155	SCA_RS05205	SE1039_RS06140	SEQMU2_RS11190	BK815_RS03650	A6V26_RS09045	AA913_RS09600	SXYL_RS07280	BE24_RS04155	SSP_RS06890
aspartate-semialdehyde dehydrogenase	BEK99_RS06160	SCA_RS05200	SE1039_RS06135	SEQMU2_RS11185	BK815_RS03655	A6V26_RS09040	AA913_RS09605	SXYL_RS07285	BE24_RS04150	SSP_RS06895
aspartate kinase	BEK99_RS06165	SCA_RS05195	SE1039_RS06130	SEQMU2_RS11180	BK815_RS03660	A6V26_RS09035	AA913_RS09610	SXYL_RS07290	BE24_RS04145	SSP_RS06900

Product	*S. carnosus*		*S. equorum*		*S. succinus*			*S. xylosus*		*S. saprophyticus*
	**JCM 6069**	**TM300**	**KS1039**	**Mu2**	**14BME20**	**CSM 77**	**DSM 14617**	**C2a**	**HKUOPL8**	**ATCC 15305**
ABC transporter ATP-binding protein	BEK99_RS06170	SCA_RS05190	SE1039_RS06120	SEQMU2_RS11170	BK815_RS03670	A6V26_RS09025	AA913_RS09620	SXYL_RS07300	BE24_RS04135	SSP_RS06910
RNA-binding protein	BEK99_RS06175	SCA_RS05185	SE1039_RS06115	SEQMU2_RS11165	BK815_RS03675	A6V26_RS09020	AA913_RS09625	SXYL_RS07305	BE24_RS04130	SSP_RS06915
thioredoxin reductase	BEK99_RS06180	SCA_RS05180	SE1039_RS06110	SEQMU2_RS11160	BK815_RS03680	A6V26_RS09015	AA913_RS09630	SXYL_RS07310	BE24_RS04125	SSP_RS06920
phosphate ABC transporter permease subunit PstC	BEK99_RS06185	SCA_RS05175	SE1039_RS06105	SEQMU2_RS11155	BK815_RS03685	A6V26_RS09010	AA913_RS09635	SXYL_RS07315	BE24_RS04120	SSP_RS06925
phosphate ABC transporter permease protein PstA	BEK99_RS06190	SCA_RS05170	SE1039_RS06100	SEQMU2_RS11150	BK815_RS03690	A6V26_RS09005	AA913_RS09640	SXYL_RS07320	BE24_RS04115	SSP_RS06930
phosphate ABC transporter ATP-binding protein	BEK99_RS06195	SCA_RS05165	SE1039_RS06095	SEQMU2_RS11145	BK815_RS03695	A6V26_RS09000	AA913_RS09645	SXYL_RS07325	BE24_RS04110	SSP_RS06935
phosphate transport system regulatory protein PhoU	BEK99_RS06200	SCA_RS05160	SE1039_RS06090	SEQMU2_RS11140	BK815_RS03700	A6V26_RS08995	AA913_RS09650	SXYL_RS07330	BE24_RS04105	SSP_RS06940
oligoendopeptidase F	BEK99_RS06205	SCA_RS05155	SE1039_RS06085	SEQMU2_RS11135	BK815_RS03705	A6V26_RS08990	AA913_RS09655	SXYL_RS07335	BE24_RS04100	SSP_RS06945
hypothetical protein	BEK99_RS06210	SCA_RS05150	SE1039_RS06080	SEQMU2_RS11130	BK815_RS03710	A6V26_RS08985	AA913_RS09660	SXYL_RS07340	BE24_RS04095	SSP_RS06950
ABC transporter permease	BEK99_RS06220	SCA_RS05140	SE1039_RS11545	SEQMU2_RS03400	BK815_RS10995	A6V26_RS08370	AA913_RS04150	SXYL_RS01465	BE24_RS10280	SSP_RS01305
ABC transporter permease	BEK99_RS06225	SCA_RS05135	SE1039_RS11540	SEQMU2_RS03395	BK815_RS11000	A6V26_RS08365	AA913_RS04145	SXYL_RS01470	BE24_RS10275	SSP_RS01310
ABC transporter ATP-binding protein	BEK99_RS06230	SCA_RS05130	SE1039_RS11535	SEQMU2_RS03390	BK815_RS11005	A6V26_RS08360	AA913_RS04140	SXYL_RS01475	BE24_RS10270	SSP_RS01315
peptide ABC transporter ATP-binding protein	BEK99_RS06235	SCA_RS05125	SE1039_RS11530	SEQMU2_RS03385	BK815_RS11010	A6V26_RS08355	AA913_RS04135	SXYL_RS01480	BE24_RS10265	SSP_RS01320
aminoacyltransferase	BEK99_RS06245	SCA_RS05115	SE1039_RS06070	SEQMU2_RS11120	BK815_RS03720	A6V26_RS08975	AA913_RS09670	SXYL_RS07355	BE24_RS04080	SSP_RS06965
aminoacyltransferase	BEK99_RS06250	SCA_RS05110	SE1039_RS06065	SEQMU2_RS11115	BK815_RS03725	A6V26_RS08970	AA913_RS09675	SXYL_RS07360	BE24_RS04075	SSP_RS06970
tryptophan synthase subunit beta	BEK99_RS06260	SCA_RS05100	SE1039_RS06050	SEQMU2_RS11100	BK815_RS03740	A6V26_RS08955	AA913_RS09690	SXYL_RS07375	BE24_RS04060	SSP_RS06985
N-(5-phosphoribosyl)anthranilate isomerase	BEK99_RS06265	SCA_RS05095	SE1039_RS06045	SEQMU2_RS11095	BK815_RS03745	A6V26_RS08950	AA913_RS09695	SXYL_RS07380	BE24_RS04055	SSP_RS06990
indole-3-glycerol-phosphate synthase	BEK99_RS06270	SCA_RS05090	SE1039_RS06040	SEQMU2_RS11090	BK815_RS03750	A6V26_RS08945	AA913_RS09700	SXYL_RS07385	BE24_RS04050	SSP_RS06995
anthranilate phosphoribosyltransferase	BEK99_RS06275	SCA_RS05085	SE1039_RS06035	SEQMU2_RS11085	BK815_RS03755	A6V26_RS08940	AA913_RS09705	SXYL_RS07390	BE24_RS04045	SSP_RS07000
glutamine amidotransferase	BEK99_RS06280	SCA_RS05080	SE1039_RS06030	SEQMU2_RS11080	BK815_RS03760	A6V26_RS08935	AA913_RS09710	SXYL_RS07395	BE24_RS04040	SSP_RS07005
anthranilate synthase component I	BEK99_RS06285	SCA_RS05075	SE1039_RS06025	SEQMU2_RS11075	BK815_RS03765	A6V26_RS08930	AA913_RS13390	SXYL_RS07400	BE24_RS04035	SSP_RS07010
aminopeptidase	BEK99_RS06290	SCA_RS05070	SE1039_RS06015	SEQMU2_RS11065	BK815_RS03970	A6V26_RS00130	AA913_RS03805	SXYL_RS07440	BE24_RS04005	SSP_RS07025
prephenate dehydrogenase	BEK99_RS06295	SCA_RS05065	SE1039_RS05990	SEQMU2_RS11055	BK815_RS03975	A6V26_RS00135	AA913_RS03800	SXYL_RS07450	BE24_RS03995	SSP_RS07040
4-oxalocrotonate tautomerase	BEK99_RS06305	SCA_RS05055	SE1039_RS05955	SEQMU2_RS11045	BK815_RS03985	A6V26_RS00145	AA913_RS03790	SXYL_RS07460	BE24_RS03985	SSP_RS07050
LytR family transcriptional regulator	BEK99_RS06310	SCA_RS05050	SE1039_RS05950	SEQMU2_RS11040	BK815_RS03990	A6V26_RS00150	AA913_RS03785	SXYL_RS07465	BE24_RS03980	SSP_RS07055
peptide-methionine (S)-S-oxide reductase	BEK99_RS06315	SCA_RS05045	SE1039_RS05945	SEQMU2_RS11035	BK815_RS03995	A6V26_RS00155	AA913_RS03780	SXYL_RS07470	BE24_RS03975	SSP_RS07060
O-acetylhomoserine	BEK99_RS06325	SCA_RS05035	SE1039_RS11620	SEQMU2_RS03475	BK815_RS10915	A6V26_RS08450	AA913_RS04230	SXYL_RS01385	BE24_RS10360	SSP_RS01225
phosphatidylglycerol lysyltransferase	BEK99_RS06330	SCA_RS05030	SE1039_RS05910	SEQMU2_RS11000	BK815_RS04025	A6V26_RS00185	AA913_RS03750	SXYL_RS07505	BE24_RS03940	SSP_RS07090
PTS glucose transporter subunit IICBA	BEK99_RS06340	SCA_RS05015	SE1039_RS11220	SEQMU2_RS03095	BK815_RS11320	A6V26_RS08045	AA913_RS01750	SXYL_RS01810	BE24_RS09955	SSP_RS01635
transcriptional regulator	BEK99_RS06345	SCA_RS05010	SE1039_RS05890	SEQMU2_RS10985	BK815_RS04035	A6V26_RS00195	AA913_RS03740	SXYL_RS07520	BE24_RS03925	SSP_RS07110
alanine:cation symporter family protein	BEK99_RS06350	SCA_RS05005	SE1039_RS05885	SEQMU2_RS10980	BK815_RS04040	A6V26_RS00200	AA913_RS03735	SXYL_RS07525	BE24_RS03920	SSP_RS07115

Product	*S. carnosus*		*S. equorum*		*S. succinus*			*S. xylosus*		*S. saprophyticus*
	**JCM 6069**	**TM300**	**KS1039**	**Mu2**	**14BME20**	**CSM 77**	**DSM 14617**	**C2a**	**HKUOPL8**	**ATCC 15305**
DNA topoisomerase IV subunit A	BEK99_RS06355	SCA_RS05000	SE1039_RS05880	SEQMU2_RS10975	BK815_RS04045	A6V26_RS00205	AA913_RS03730	SXYL_RS07530	BE24_RS03915	SSP_RS07120
DNA topoisomerase IV subunit B	BEK99_RS06360	SCA_RS04995	SE1039_RS05875	SEQMU2_RS10970	BK815_RS04050	A6V26_RS00210	AA913_RS03725	SXYL_RS07535	BE24_RS03910	SSP_RS07125
glycerol-3-phosphate acyltransferase	BEK99_RS06365	SCA_RS04990	SE1039_RS05870	SEQMU2_RS10965	BK815_RS04055	A6V26_RS00215	AA913_RS03720	SXYL_RS07540	BE24_RS03905	SSP_RS07130
hypothetical protein	BEK99_RS06370	SCA_RS04985	SE1039_RS05865	SEQMU2_RS10960	BK815_RS04060	A6V26_RS00220	AA913_RS03715	SXYL_RS07545	BE24_RS03900	SSP_RS07135
4-hydroxybenzoyl-CoA thioesterase	BEK99_RS06375	SCA_RS04980	SE1039_RS05860	SEQMU2_RS10955	BK815_RS04065	A6V26_RS00225	AA913_RS03710	SXYL_RS07550	BE24_RS03895	SSP_RS07140
aconitate hydratase	BEK99_RS06380	SCA_RS04975	SE1039_RS05855	SEQMU2_RS10950	BK815_RS04070	A6V26_RS00230	AA913_RS03705	SXYL_RS07555	BE24_RS03890	SSP_RS07145
choline transporter	BEK99_RS06385	SCA_RS04970	SE1039_RS05850	SEQMU2_RS10945	BK815_RS04075	A6V26_RS00235	AA913_RS03700	SXYL_RS07560	BE24_RS03885	SSP_RS07150
large-conductance mechanosensitive channel	BEK99_RS06390	SCA_RS04965	SE1039_RS05845	SEQMU2_RS10940	BK815_RS04080	A6V26_RS00240	AA913_RS03695	SXYL_RS07565	BE24_RS03880	SSP_RS07155
hypothetical protein	BEK99_RS06395	SCA_RS04960	SE1039_RS05840	SEQMU2_RS10935	BK815_RS04085	A6V26_RS00245	AA913_RS03690	SXYL_RS07570	BE24_RS03875	SSP_RS07160
exonuclease sbcCD subunit D	BEK99_RS06400	SCA_RS04955	SE1039_RS05835	SEQMU2_RS10930	BK815_RS04090	A6V26_RS00250	AA913_RS03685	SXYL_RS07575	BE24_RS03870	SSP_RS07165
cytochrome C biogenesis protein CcdC	BEK99_RS06405	SCA_RS04950	SE1039_RS05830	SEQMU2_RS10925	BK815_RS04095	A6V26_RS00255	AA913_RS03680	SXYL_RS07580	BE24_RS03865	SSP_RS07170
hypothetical protein	BEK99_RS06410	SCA_RS04945	SE1039_RS05795	SEQMU2_RS10645	BK815_RS04105	A6V26_RS00265	AA913_RS03670	SXYL_RS07590	BE24_RS03855	SSP_RS07180
transketolase	BEK99_RS06415	SCA_RS04940	SE1039_RS05790	SEQMU2_RS10640	BK815_RS04110	A6V26_RS00270	AA913_RS03665	SXYL_RS07595	BE24_RS03850	SSP_RS07185
DUF896 family protein	BEK99_RS06420	SCA_RS04935	SE1039_RS05785	SEQMU2_RS10635	BK815_RS04115	A6V26_RS00275	AA913_RS03660	SXYL_RS07600	BE24_RS03845	SSP_RS07190
hypothetical protein	BEK99_RS06425	SCA_RS04930	SE1039_RS05780	SEQMU2_RS10630	BK815_RS04120	A6V26_RS00280	AA913_RS03655	SXYL_RS07605	BE24_RS03840	SSP_RS07195
LexA repressor	BEK99_RS06430	SCA_RS04925	SE1039_RS05775	SEQMU2_RS10625	BK815_RS04125	A6V26_RS00285	AA913_RS03650	SXYL_RS07610	BE24_RS03835	SSP_RS07200
secretion protein	BEK99_RS06435	SCA_RS04920	SE1039_RS05770	SEQMU2_RS10620	BK815_RS04130	A6V26_RS00290	AA913_RS03645	SXYL_RS07615	BE24_RS03830	SSP_RS07205
GMP reductase	BEK99_RS06440	SCA_RS04915	SE1039_RS05765	SEQMU2_RS10615	BK815_RS04135	A6V26_RS00295	AA913_RS03640	SXYL_RS07620	BE24_RS03825	SSP_RS07210
30S ribosomal protein S14	BEK99_RS06445	SCA_RS04910	SE1039_RS05760	SEQMU2_RS10610	BK815_RS04140	A6V26_RS00300	AA913_RS03635	SXYL_RS07625	BE24_RS03820	SSP_RS07215
50S ribosomal protein L33	BEK99_RS06450	SCA_RS04905	SE1039_RS05755	SEQMU2_RS10605	BK815_RS04145	A6V26_RS00305	AA913_RS03630	SXYL_RS07630	BE24_RS03815	SSP_RS07220
gamma-aminobutyrate permease	BEK99_RS06455	SCA_RS04900	SE1039_RS05740	SEQMU2_RS10590	BK815_RS04155	A6V26_RS00315	AA913_RS03620	SXYL_RS07640	BE24_RS03805	SSP_RS07275
hypothetical protein	BEK99_RS06460	SCA_RS04895	SE1039_RS05735	SEQMU2_RS10585	BK815_RS04160	A6V26_RS00320	AA913_RS03615	SXYL_RS07645	BE24_RS03800	SSP_RS07280
HAD family phosphatase	BEK99_RS06465	SCA_RS04890	SE1039_RS05730	SEQMU2_RS10580	BK815_RS04165	A6V26_RS00325	AA913_RS03610	SXYL_RS07650	BE24_RS03795	SSP_RS07285
homoserine kinase	BEK99_RS06470	SCA_RS04885	SE1039_RS05725	SEQMU2_RS10575	BK815_RS04170	A6V26_RS00330	AA913_RS03605	SXYL_RS07655	BE24_RS03790	SSP_RS07290
threonine synthase	BEK99_RS06475	SCA_RS04880	SE1039_RS05720	SEQMU2_RS10570	BK815_RS04175	A6V26_RS00335	AA913_RS03600	SXYL_RS07660	BE24_RS03785	SSP_RS07295
homoserine dehydrogenase	BEK99_RS06480	SCA_RS04875	SE1039_RS05715	SEQMU2_RS10565	BK815_RS04180	A6V26_RS00340	AA913_RS03595	SXYL_RS07665	BE24_RS03780	SSP_RS07300
aspartate kinase	BEK99_RS06485	SCA_RS04870	SE1039_RS05710	SEQMU2_RS10560	BK815_RS04185	A6V26_RS00345	AA913_RS03590	SXYL_RS07670	BE24_RS03775	SSP_RS07305
hypothetical protein	BEK99_RS06490	SCA_RS04865	SE1039_RS05705	SEQMU2_RS10555	BK815_RS04190	A6V26_RS00350	AA913_RS03585	SXYL_RS07675	BE24_RS03770	SSP_RS07310
hypothetical protein	BEK99_RS06505	SCA_RS04850	SE1039_RS05695	SEQMU2_RS10545	BK815_RS04200	A6V26_RS00360	AA913_RS03575	SXYL_RS07685	BE24_RS03760	SSP_RS07320
thermonuclease	BEK99_RS06515	SCA_RS04840	SE1039_RS05690	SEQMU2_RS10540	BK815_RS04205	A6V26_RS00365	AA913_RS03570	SXYL_RS07690	BE24_RS03745	SSP_RS07325
hypothetical protein	BEK99_RS06520	SCA_RS04835	SE1039_RS05685	SEQMU2_RS10535	BK815_RS04210	A6V26_RS00370	AA913_RS03565	SXYL_RS07695	BE24_RS03740	SSP_RS07330

Product	*S. carnosus*		*S. equorum*		*S. succinus*			*S. xylosus*		*S. saprophyticus*
	**JCM 6069**	**TM300**	**KS1039**	**Mu2**	**14BME20**	**CSM 77**	**DSM 14617**	**C2a**	**HKUOPL8**	**ATCC 15305**
cardiolipin synthase	BEK99_RS06525	SCA_RS08070	SE1039_RS09040	SEQMU2_RS00915	BK815_RS00355	A6V26_RS11755	AA913_RS11335	SXYL_RS04145	BE24_RS07610	SSP_RS00915
MerR family transcriptional regulator	BEK99_RS06535	SCA_RS04820	SE1039_RS05660	SEQMU2_RS10510	BK815_RS04260	A6V26_RS00420	AA913_RS03510	SXYL_RS07725	BE24_RS03695	SSP_RS07365
hypothetical protein	BEK99_RS06545	SCA_RS04810	SE1039_RS05655	SEQMU2_RS10505	BK815_RS04265	A6V26_RS00425	AA913_RS03505	SXYL_RS07730	BE24_RS03690	SSP_RS07370
GTPase HflX	BEK99_RS06550	SCA_RS04805	SE1039_RS05650	SEQMU2_RS10500	BK815_RS04270	A6V26_RS00430	AA913_RS03500	SXYL_RS07735	BE24_RS03685	SSP_RS07375
glutathione peroxidase	BEK99_RS06555	SCA_RS04800	SE1039_RS05645	SEQMU2_RS10495	BK815_RS04275	A6V26_RS00435	AA913_RS03495	SXYL_RS07740	BE24_RS03680	SSP_RS07380
RNA-binding protein Hfq	BEK99_RS06560	SCA_RS04795	SE1039_RS05640	SEQMU2_RS10490	BK815_RS04280	A6V26_RS00440	AA913_RS03490	SXYL_RS07745	BE24_RS03675	SSP_RS07385
lysophospholipase	BEK99_RS06570	SCA_RS04785	SE1039_RS05630	SEQMU2_RS10480	BK815_RS04290	A6V26_RS00450	AA913_RS03480	SXYL_RS07755	BE24_RS03665	SSP_RS07395
glycerol-3-phosphate dehydrogenase/oxidase	BEK99_RS06575	SCA_RS04780	SE1039_RS05610	SEQMU2_RS10460	BK815_RS04295	A6V26_RS00455	AA913_RS03475	SXYL_RS07760	BE24_RS03660	SSP_RS07400
glycerol kinase	BEK99_RS06580	SCA_RS04775	SE1039_RS05605	SEQMU2_RS10455	BK815_RS04300	A6V26_RS00460	AA913_RS03470	SXYL_RS07765	BE24_RS03655	SSP_RS07405
aquaporin family protein	BEK99_RS06585	SCA_RS04770	SE1039_RS05600	SEQMU2_RS10450	BK815_RS04305	A6V26_RS00465	AA913_RS03465	SXYL_RS07770	BE24_RS03650	SSP_RS07415
glycerol-3-phosphate responsive antiterminator	BEK99_RS06590	SCA_RS04765	SE1039_RS05590	SEQMU2_RS10440	BK815_RS04315	A6V26_RS00475	AA913_RS03455	SXYL_RS07780	BE24_RS03640	SSP_RS07425
DNA mismatch repair protein MutL	BEK99_RS06595	SCA_RS04760	SE1039_RS05585	SEQMU2_RS10435	BK815_RS04320	A6V26_RS00480	AA913_RS03450	SXYL_RS07785	BE24_RS03635	SSP_RS07430
DNA mismatch repair protein MutS	BEK99_RS06600	SCA_RS04755	SE1039_RS05580	SEQMU2_RS10430	BK815_RS04325	A6V26_RS00485	AA913_RS03445	SXYL_RS07790	BE24_RS03630	SSP_RS07435
glycerophosphodiester phosphodiesterase	BEK99_RS06605	SCA_RS04750	SE1039_RS03620	SEQMU2_RS08845	BK815_RS05910	A6V26_RS02040	AA913_RS09010	SXYL_RS09635	BE24_RS02100	SSP_RS09125
energy coupling factor transporter S component ThiW	BEK99_RS06610	SCA_RS04745	SE1039_RS05555	SEQMU2_RS10405	BK815_RS04330	A6V26_RS00490	AA913_RS03440	SXYL_RS07795	BE24_RS03625	SSP_RS07440
hypothetical protein	BEK99_RS06615	SCA_RS04740	SE1039_RS05550	SEQMU2_RS10400	BK815_RS04335	A6V26_RS00495	AA913_RS03435	SXYL_RS07800	BE24_RS03620	SSP_RS07445
hypothetical protein	BEK99_RS06650	SCA_RS04705	SE1039_RS05540	SEQMU2_RS10390	BK815_RS04345	A6V26_RS00505	AA913_RS03425	SXYL_RS07810	BE24_RS03610	SSP_RS07455
2-oxoacid ferredoxin oxidoreductase subunit beta	BEK99_RS06660	SCA_RS04695	SE1039_RS05520	SEQMU2_RS10370	BK815_RS04360	A6V26_RS00520	AA913_RS03410	SXYL_RS07835	BE24_RS03585	SSP_RS07475
2-oxoacid:acceptor oxidoreductase subunit alpha	BEK99_RS06665	SCA_RS04690	SE1039_RS05515	SEQMU2_RS10365	BK815_RS04365	A6V26_RS00525	AA913_RS03405	SXYL_RS07840	BE24_RS03580	SSP_RS07480
metallophosphoesterase	BEK99_RS06670	SCA_RS04685	SE1039_RS05510	SEQMU2_RS10360	BK815_RS04370	A6V26_RS00530	AA913_RS03400	SXYL_RS07845	BE24_RS03575	SSP_RS07485
hypothetical protein	BEK99_RS06675	SCA_RS04680	SE1039_RS05505	SEQMU2_RS10355	BK815_RS04375	A6V26_RS00535	AA913_RS03395	SXYL_RS07850	BE24_RS03570	SSP_RS07490
ribonuclease Y	BEK99_RS06690	SCA_RS04665	SE1039_RS05500	SEQMU2_RS10350	BK815_RS04380	A6V26_RS00540	AA913_RS03390	SXYL_RS07855	BE24_RS03565	SSP_RS07495
DNA recombination/repair protein RecA	BEK99_RS06695	SCA_RS04660	SE1039_RS05495	SEQMU2_RS10345	BK815_RS04385	A6V26_RS00545	AA913_RS03385	SXYL_RS07860	BE24_RS03560	SSP_RS07500
competence protein CinA	BEK99_RS06700	SCA_RS04655	SE1039_RS05490	SEQMU2_RS10340	BK815_RS04390	A6V26_RS00550	AA913_RS03380	SXYL_RS07865	BE24_RS03555	SSP_RS07505
CDP-diacylglycerol--glycerol-3-phosphate 3-phosphatidyltransferase	BEK99_RS06705	SCA_RS04650	SE1039_RS05485	SEQMU2_RS10335	BK815_RS04395	A6V26_RS00555	AA913_RS03375	SXYL_RS07870	BE24_RS03550	SSP_RS07510
transcriptional regulator	BEK99_RS06710	SCA_RS04645	SE1039_RS05480	SEQMU2_RS10330	BK815_RS04400	A6V26_RS00560	AA913_RS03370	SXYL_RS07875	BE24_RS03545	SSP_RS07515
hypothetical protein	BEK99_RS06715	SCA_RS04640	SE1039_RS05475	SEQMU2_RS10325	BK815_RS04405	A6V26_RS00565	AA913_RS03365	SXYL_RS07880	BE24_RS03540	SSP_RS07520
3-oxoacyl-ACP reductase	BEK99_RS06720	SCA_RS04635	SE1039_RS05470	SEQMU2_RS10320	BK815_RS04410	A6V26_RS00570	AA913_RS03360	SXYL_RS07885	BE24_RS03535	SSP_RS07530
insulinase family protein	BEK99_RS06725	SCA_RS04630	SE1039_RS05465	SEQMU2_RS10315	BK815_RS04415	A6V26_RS00575	AA913_RS03355	SXYL_RS07890	BE24_RS03530	SSP_RS07535
insulinase family protein	BEK99_RS06730	SCA_RS04625	SE1039_RS05460	SEQMU2_RS10310	BK815_RS04420	A6V26_RS00580	AA913_RS03350	SXYL_RS07895	BE24_RS03525	SSP_RS07540
UTRA domain-containing protein	BEK99_RS06735	SCA_RS04620	SE1039_RS05455	SEQMU2_RS10305	BK815_RS04425	A6V26_RS00585	AA913_RS03345	SXYL_RS07900	BE24_RS03520	SSP_RS07545

Product	*S. carnosus*		*S. equorum*		*S. succinus*			*S. xylosus*		*S. saprophyticus*
	**JCM 6069**	**TM300**	**KS1039**	**Mu2**	**14BME20**	**CSM 77**	**DSM 14617**	**C2a**	**HKUOPL8**	**ATCC 15305**
DNA translocase FtsK	BEK99_RS06740	SCA_RS04615	SE1039_RS05450	SEQMU2_RS10300	BK815_RS04430	A6V26_RS00590	AA913_RS03340	SXYL_RS07905	BE24_RS03515	SSP_RS07550
ribonuclease J	BEK99_RS06745	SCA_RS04610	SE1039_RS05445	SEQMU2_RS10295	BK815_RS04435	A6V26_RS00595	AA913_RS03335	SXYL_RS07910	BE24_RS03510	SSP_RS07555
polyribonucleotide nucleotidyltransferase	BEK99_RS06750	SCA_RS04605	SE1039_RS05440	SEQMU2_RS10290	BK815_RS04440	A6V26_RS00600	AA913_RS03330	SXYL_RS07915	BE24_RS03505	SSP_RS07560
30S ribosomal protein S15	BEK99_RS06755	SCA_RS04600	SE1039_RS05435	SEQMU2_RS10285	BK815_RS04445	A6V26_RS00605	AA913_RS03325	SXYL_RS07920	BE24_RS03500	SSP_RS07565
riboflavin biosynthesis protein RibF	BEK99_RS06760	SCA_RS04595	SE1039_RS05430	SEQMU2_RS10280	BK815_RS04450	A6V26_RS00610	AA913_RS03320	SXYL_RS07925	BE24_RS03495	SSP_RS07570
tRNA pseudouridine(55) synthase TruB	BEK99_RS06765	SCA_RS04590	SE1039_RS05425	SEQMU2_RS10275	BK815_RS04455	A6V26_RS00615	AA913_RS03315	SXYL_RS07930	BE24_RS03490	SSP_RS07575
ribosome-binding factor A	BEK99_RS06770	SCA_RS04585	SE1039_RS05420	SEQMU2_RS10270	BK815_RS04460	A6V26_RS00620	AA913_RS03310	SXYL_RS07935	BE24_RS03485	SSP_RS07580
translation initiation factor IF-2	BEK99_RS06795	SCA_RS04560	SE1039_RS05405	SEQMU2_RS10255	BK815_RS04475	A6V26_RS00635	AA913_RS03295	SXYL_RS07950	BE24_RS03470	SSP_RS07595
hypothetical protein	BEK99_RS06800	SCA_RS04555	SE1039_RS05400	SEQMU2_RS10250	BK815_RS04480	A6V26_RS00640	AA913_RS03290	SXYL_RS07955	BE24_RS03465	SSP_RS07600
DNA-binding protein	BEK99_RS06805	SCA_RS04550	SE1039_RS05395	SEQMU2_RS10245	BK815_RS04485	A6V26_RS00645	AA913_RS03285	SXYL_RS07960	BE24_RS03460	SSP_RS07605
transcription termination/antitermination protein NusA	BEK99_RS06810	SCA_RS04545	SE1039_RS05390	SEQMU2_RS10240	BK815_RS04490	A6V26_RS00650	AA913_RS03280	SXYL_RS07965	BE24_RS03455	SSP_RS07610
ribosome maturation factor RimP	BEK99_RS06815	SCA_RS04540	SE1039_RS05385	SEQMU2_RS10235	BK815_RS04495	A6V26_RS00655	AA913_RS03275	SXYL_RS07970	BE24_RS03450	SSP_RS07615
PolC-type DNA polymerase III	BEK99_RS06820	SCA_RS04535	SE1039_RS05380	SEQMU2_RS10230	BK815_RS04500	A6V26_RS00660	AA913_RS03270	SXYL_RS07975	BE24_RS03445	SSP_RS07620
proline--tRNA ligase	BEK99_RS06825	SCA_RS04530	SE1039_RS05375	SEQMU2_RS10225	BK815_RS04505	A6V26_RS00665	AA913_RS03265	SXYL_RS07980	BE24_RS03440	SSP_RS07625
RIP metalloprotease RseP	BEK99_RS06830	SCA_RS04525	SE1039_RS05370	SEQMU2_RS10220	BK815_RS04510	A6V26_RS00670	AA913_RS03260	SXYL_RS07985	BE24_RS03435	SSP_RS07630
phosphatidate cytidylyltransferase	BEK99_RS06835	SCA_RS04520	SE1039_RS05365	SEQMU2_RS10215	BK815_RS04515	A6V26_RS00675	AA913_RS03255	SXYL_RS07990	BE24_RS03430	SSP_RS07635
isoprenyl transferase	BEK99_RS06840	SCA_RS04515	SE1039_RS05360	SEQMU2_RS10210	BK815_RS04520	A6V26_RS00680	AA913_RS03250	SXYL_RS07995	BE24_RS03425	SSP_RS07640
ribosome-recycling factor	BEK99_RS06845	SCA_RS04510	SE1039_RS05355	SEQMU2_RS10205	BK815_RS04525	A6V26_RS00685	AA913_RS03245	SXYL_RS08000	BE24_RS03420	SSP_RS07645
UMP kinase	BEK99_RS06850	SCA_RS04505	SE1039_RS05350	SEQMU2_RS10200	BK815_RS04530	A6V26_RS00690	AA913_RS03240	SXYL_RS08005	BE24_RS03415	SSP_RS07650
elongation factor Ts	BEK99_RS06855	SCA_RS04500	SE1039_RS05345	SEQMU2_RS10195	BK815_RS04535	A6V26_RS00695	AA913_RS03235	SXYL_RS08010	BE24_RS03410	SSP_RS07655
30S ribosomal protein S2	BEK99_RS06860	SCA_RS04495	SE1039_RS05340	SEQMU2_RS10190	BK815_RS04540	A6V26_RS00700	AA913_RS03230	SXYL_RS08015	BE24_RS03405	SSP_RS07660
GTP-sensing pleiotropic transcriptional regulator CodY	BEK99_RS06865	SCA_RS04490	SE1039_RS05335	SEQMU2_RS10185	BK815_RS04545	A6V26_RS00705	AA913_RS03225	SXYL_RS08020	BE24_RS03400	SSP_RS07665
HslU--HslV peptidase ATPase subunit	BEK99_RS06870	SCA_RS04485	SE1039_RS05330	SEQMU2_RS10180	BK815_RS04550	A6V26_RS00710	AA913_RS03220	SXYL_RS08025	BE24_RS03395	SSP_RS07670
ATP-dependent protease subunit HslV	BEK99_RS06875	SCA_RS04480	SE1039_RS05325	SEQMU2_RS10175	BK815_RS04555	A6V26_RS00715	AA913_RS03215	SXYL_RS08030	BE24_RS03390	SSP_RS07675
recombinase XerC	BEK99_RS06880	SCA_RS04475	SE1039_RS05320	SEQMU2_RS10170	BK815_RS04560	A6V26_RS00720	AA913_RS03210	SXYL_RS08035	BE24_RS03385	SSP_RS07680
FADH(2)-oxidizing methylenetetrahydrofolate--tRNA-(uracil(54)-C(5))- methyltransferase TrmFO	BEK99_RS06885	SCA_RS04470	SE1039_RS05315	SEQMU2_RS10165	BK815_RS04565	A6V26_RS00725	AA913_RS03205	SXYL_RS08040	BE24_RS03380	SSP_RS07685
DNA topoisomerase I	BEK99_RS06890	SCA_RS04465	SE1039_RS05310	SEQMU2_RS10160	BK815_RS04570	A6V26_RS00730	AA913_RS03200	SXYL_RS08045	BE24_RS03375	SSP_RS07690
succinate--CoA ligase subunit alpha	BEK99_RS06895	SCA_RS04460	SE1039_RS05300	SEQMU2_RS10150	BK815_RS04580	A6V26_RS00740	AA913_RS03190	SXYL_RS08055	BE24_RS03365	SSP_RS07700
succinyl-CoA ligase subunit beta	BEK99_RS06900	SCA_RS04455	SE1039_RS05295	SEQMU2_RS10145	BK815_RS04585	A6V26_RS00745	AA913_RS03185	SXYL_RS08060	BE24_RS03360	SSP_RS07705
ribonuclease HII	BEK99_RS06905	SCA_RS04450	SE1039_RS05290	SEQMU2_RS10140	BK815_RS04590	A6V26_RS00750	AA913_RS03180	SXYL_RS08065	BE24_RS03355	SSP_RS07710
ribosome biogenesis GTPase YlqF	BEK99_RS06910	SCA_RS04445	SE1039_RS05285	SEQMU2_RS10135	BK815_RS04595	A6V26_RS00755	AA913_RS03175	SXYL_RS08070	BE24_RS03350	SSP_RS07715

Product	*S. carnosus*		*S. equorum*		*S. succinus*			*S. xylosus*		*S. saprophyticus*
	**JCM 6069**	**TM300**	**KS1039**	**Mu2**	**14BME20**	**CSM 77**	**DSM 14617**	**C2a**	**HKUOPL8**	**ATCC 15305**
50S ribosomal protein L19	BEK99_RS06985	SCA_RS04375	SE1039_RS05270	SEQMU2_RS10120	BK815_RS04610	A6V26_RS00770	AA913_RS03160	SXYL_RS08085	BE24_RS03335	SSP_RS07730
tRNA (guanosine(37)-N1)-methyltransferase TrmD	BEK99_RS06990	SCA_RS04370	SE1039_RS05265	SEQMU2_RS10115	BK815_RS04615	A6V26_RS00775	AA913_RS03155	SXYL_RS08090	BE24_RS03330	SSP_RS07735
ribosome maturation factor RimM	BEK99_RS06995	SCA_RS04365	SE1039_RS05260	SEQMU2_RS10110	BK815_RS04620	A6V26_RS00780	AA913_RS03150	SXYL_RS08095	BE24_RS03325	SSP_RS07740
30S ribosomal protein S16	BEK99_RS07000	SCA_RS04360	SE1039_RS05255	SEQMU2_RS10105	BK815_RS04625	A6V26_RS00785	AA913_RS03145	SXYL_RS08100	BE24_RS03320	SSP_RS07745
DNA-binding protein	BEK99_RS07015	SCA_RS04345	SE1039_RS05235	SEQMU2_RS10085	BK815_RS04645	A6V26_RS00805	AA913_RS03125	SXYL_RS08120	BE24_RS03300	SSP_RS07765
signal recognition particle-docking protein FtsY	BEK99_RS07020	SCA_RS04340	SE1039_RS05230	SEQMU2_RS10080	BK815_RS04650	A6V26_RS00810	AA913_RS03120	SXYL_RS08125	BE24_RS03295	SSP_RS07770
chromosome segregation protein SMC	BEK99_RS07025	SCA_RS04335	SE1039_RS05225	SEQMU2_RS10075	BK815_RS04655	A6V26_RS00815	AA913_RS03115	SXYL_RS08130	BE24_RS03290	SSP_RS07775
ribonuclease 3	BEK99_RS07030	SCA_RS04330	SE1039_RS05220	SEQMU2_RS10070	BK815_RS04660	A6V26_RS00820	AA913_RS03110	SXYL_RS08135	BE24_RS03285	SSP_RS07780
acyl carrier protein	BEK99_RS07035	SCA_RS04325	SE1039_RS05215	SEQMU2_RS10065	BK815_RS04665	A6V26_RS00825	AA913_RS03105	SXYL_RS08140	BE24_RS03280	SSP_RS07785
3-oxoacyl-[acyl-carrier-protein] reductase	BEK99_RS07040	SCA_RS04320	SE1039_RS05210	SEQMU2_RS10060	BK815_RS04670	A6V26_RS00830	AA913_RS03100	SXYL_RS08145	BE24_RS03275	SSP_RS07790
malonyl CoA-acyl carrier protein transacylase	BEK99_RS07045	SCA_RS04315	SE1039_RS05205	SEQMU2_RS10055	BK815_RS04675	A6V26_RS00835	AA913_RS03095	SXYL_RS08150	BE24_RS03270	SSP_RS07795
phosphate acyltransferase	BEK99_RS07050	SCA_RS04310	SE1039_RS05200	SEQMU2_RS10050	BK815_RS04680	A6V26_RS00840	AA913_RS03090	SXYL_RS08155	BE24_RS03265	SSP_RS07800
fatty acid biosynthesis transcriptional regulator	BEK99_RS07055	SCA_RS04305	SE1039_RS05195	SEQMU2_RS10045	BK815_RS04685	A6V26_RS00845	AA913_RS03085	SXYL_RS08160	BE24_RS03260	SSP_RS07805
L-serine dehydratase iron-sulfur-dependent subunit beta	BEK99_RS07075	SCA_RS04290	SE1039_RS05175	SEQMU2_RS10025	BK815_RS04705	A6V26_RS00865	AA913_RS03065	SXYL_RS08180	BE24_RS03240	SSP_RS07825
hypothetical protein	BEK99_RS07080	SCA_RS04285	SE1039_RS05170	SEQMU2_RS10020	BK815_RS04710	A6V26_RS00870	AA913_RS03060	SXYL_RS08185	BE24_RS03235	SSP_RS07830
Asp23/Gls24 family envelope stress response protein	BEK99_RS07085	SCA_RS04280	SE1039_RS05165	SEQMU2_RS10015	BK815_RS04715	A6V26_RS00875	AA913_RS03055	SXYL_RS08190	BE24_RS03230	SSP_RS07835
50S ribosomal protein L28	BEK99_RS07090	SCA_RS04275	SE1039_RS05160	SEQMU2_RS10010	BK815_RS04720	A6V26_RS00880	AA913_RS03050	SXYL_RS08195	BE24_RS03225	SSP_RS07840
thiamine pyrophosphokinase	BEK99_RS07100	SCA_RS04265	SE1039_RS05155	SEQMU2_RS10005	BK815_RS04725	A6V26_RS00885	AA913_RS03045	SXYL_RS08200	BE24_RS03220	SSP_RS07845
ribulose-phosphate 3-epimerase	BEK99_RS07105	SCA_RS04260	SE1039_RS05150	SEQMU2_RS10000	BK815_RS04730	A6V26_RS00890	AA913_RS03040	SXYL_RS08205	BE24_RS03215	SSP_RS07850
GTPase	BEK99_RS07110	SCA_RS04255	SE1039_RS05145	SEQMU2_RS09995	BK815_RS04735	A6V26_RS00895	AA913_RS03035	SXYL_RS08210	BE24_RS03210	SSP_RS07855
serine/threonine protein kinase	BEK99_RS07115	SCA_RS04250	SE1039_RS05135	SEQMU2_RS09990	BK815_RS04740	A6V26_RS00900	AA913_RS03030	SXYL_RS08215	BE24_RS03205	SSP_RS07860
protein phosphatase	BEK99_RS07120	SCA_RS04245	SE1039_RS05130	SEQMU2_RS09985	BK815_RS04745	A6V26_RS00905	AA913_RS03025	SXYL_RS08220	BE24_RS03200	SSP_RS07865
23S rRNA (adenine(2503)-C(2))-methyltransferase RlmN	BEK99_RS07125	SCA_RS04240	SE1039_RS05125	SEQMU2_RS09980	BK815_RS04750	A6V26_RS00910	AA913_RS03020	SXYL_RS08225	BE24_RS03195	SSP_RS07870
16S rRNA (cytosine(967)-C(5))-methyltransferase	BEK99_RS07130	SCA_RS04235	SE1039_RS05120	SEQMU2_RS09975	BK815_RS04755	A6V26_RS00915	AA913_RS03015	SXYL_RS08230	BE24_RS03190	SSP_RS07875
methionyl-tRNA formyltransferase	BEK99_RS07135	SCA_RS04230	SE1039_RS05115	SEQMU2_RS09970	BK815_RS04760	A6V26_RS00920	AA913_RS03010	SXYL_RS08235	BE24_RS03185	SSP_RS07880
peptide deformylase	BEK99_RS07140	SCA_RS04225	SE1039_RS05110	SEQMU2_RS09965	BK815_RS04765	A6V26_RS00925	AA913_RS03005	SXYL_RS08240	BE24_RS03180	SSP_RS07885
primosomal protein N	BEK99_RS07165	SCA_RS04200	SE1039_RS04770	SEQMU2_RS09950	BK815_RS04800	A6V26_RS00960	AA913_RS02970	SXYL_RS08255	BE24_RS03165	SSP_RS07895
phosphopantothenoylcysteine decarboxylase	BEK99_RS07170	SCA_RS04195	SE1039_RS04765	SEQMU2_RS09945	BK815_RS04805	A6V26_RS00965	AA913_RS02965	SXYL_RS08260	BE24_RS03160	SSP_RS07900
thiol reductase thioredoxin	BEK99_RS07175	SCA_RS04190	SE1039_RS11200	SEQMU2_RS03075	BK815_RS11060	A6V26_RS08305	AA913_RS04085	SXYL_RS01835	BE24_RS09930	SSP_RS01655
DNA-directed RNA polymerase subunit omega	BEK99_RS07180	SCA_RS04185	SE1039_RS04760	SEQMU2_RS09940	BK815_RS04810	A6V26_RS00970	AA913_RS02960	SXYL_RS08265	BE24_RS03155	SSP_RS07905
hypothetical protein	BEK99_RS07190	SCA_RS04175	SE1039_RS04750	SEQMU2_RS09930	BK815_RS04820	A6V26_RS00980	AA913_RS02950	SXYL_RS08275	BE24_RS03145	SSP_RS07915

**Product**	***S. carnosus***		***S. equorum***		***S. succinus***			***S. xylosus***		***S. saprophyticus***
	**JCM 6069**	**TM300**	**KS1039**	**Mu2**	**14BME20**	**CSM 77**	**DSM 14617**	**C2a**	**HKUOPL8**	**ATCC 15305**
hypothetical protein	BEK99_RS07200	SCA_RS04165	SE1039_RS04745	SEQMU2_RS09925	BK815_RS04825	A6V26_RS00985	AA913_RS02945	SXYL_RS08280	BE24_RS03140	SSP_RS07920
hypothetical protein	BEK99_RS07205	SCA_RS04160	SE1039_RS04735	SEQMU2_RS09915	BK815_RS04835	A6V26_RS00995	AA913_RS02935	SXYL_RS08290	BE24_RS03130	SSP_RS07930
orotate phosphoribosyltransferase	BEK99_RS07210	SCA_RS04155	SE1039_RS04730	SEQMU2_RS09910	BK815_RS04840	A6V26_RS01000	AA913_RS02930	SXYL_RS08295	BE24_RS03125	SSP_RS07935
orotidine-5-phosphate decarboxylase	BEK99_RS07215	SCA_RS04150	SE1039_RS04725	SEQMU2_RS09905	BK815_RS04845	A6V26_RS01005	AA913_RS02925	SXYL_RS08300	BE24_RS03120	SSP_RS07940
carbamoyl phosphate synthase large subunit	BEK99_RS07230	SCA_RS04135	SE1039_RS04720	SEQMU2_RS09900	BK815_RS04850	A6V26_RS01010	AA913_RS02920	SXYL_RS08305	BE24_RS03115	SSP_RS07945
carbamoyl phosphate synthase small subunit	BEK99_RS07235	SCA_RS04130	SE1039_RS04715	SEQMU2_RS09895	BK815_RS04855	A6V26_RS01015	AA913_RS02915	SXYL_RS08310	BE24_RS03110	SSP_RS07950
dihydroorotase	BEK99_RS07240	SCA_RS04125	SE1039_RS04710	SEQMU2_RS09890	BK815_RS04860	A6V26_RS01020	AA913_RS02910	SXYL_RS08315	BE24_RS03105	SSP_RS07955
aspartate carbamoyltransferase	BEK99_RS07245	SCA_RS04120	SE1039_RS04705	SEQMU2_RS09885	BK815_RS04865	A6V26_RS01025	AA913_RS02905	SXYL_RS08320	BE24_RS03100	SSP_RS07960
uracil permease	BEK99_RS07250	SCA_RS04115	SE1039_RS04700	SEQMU2_RS09880	BK815_RS04870	A6V26_RS01030	AA913_RS02900	SXYL_RS08325	BE24_RS03095	SSP_RS07965
bifunctional pyrimidine operon transcriptional regulator/uracil phosphoribosyltransferase	BEK99_RS07255	SCA_RS04110	SE1039_RS04695	SEQMU2_RS09875	BK815_RS04875	A6V26_RS01035	AA913_RS02895	SXYL_RS08330	BE24_RS03090	SSP_RS07970
pseudouridine synthase	BEK99_RS07260	SCA_RS04105	SE1039_RS04690	SEQMU2_RS09870	BK815_RS04880	A6V26_RS01040	AA913_RS02890	SXYL_RS08335	BE24_RS03085	SSP_RS07975
signal peptidase II	BEK99_RS07265	SCA_RS04100	SE1039_RS04685	SEQMU2_RS09865	BK815_RS04885	A6V26_RS01045	AA913_RS02885	SXYL_RS08340	BE24_RS03080	SSP_RS07980
isoleucine--tRNA ligase	BEK99_RS07280	SCA_RS04085	SE1039_RS04670	SEQMU2_RS09850	BK815_RS04910	A6V26_RS01070	AA913_RS02860	SXYL_RS08350	BE24_RS03070	SSP_RS07990
cell division protein DivIVA	BEK99_RS07285	SCA_RS04080	SE1039_RS04665	SEQMU2_RS09845	BK815_RS04915	A6V26_RS01075	AA913_RS02855	SXYL_RS08355	BE24_RS03065	SSP_RS07995
RNA-binding protein	BEK99_RS07290	SCA_RS04075	SE1039_RS04660	SEQMU2_RS09840	BK815_RS04920	A6V26_RS01080	AA913_RS02850	SXYL_RS08360	BE24_RS03060	SSP_RS08000
cell division protein	BEK99_RS07295	SCA_RS04070	SE1039_RS04655	SEQMU2_RS09835	BK815_RS04925	A6V26_RS01085	AA913_RS02845	SXYL_RS08365	BE24_RS03055	SSP_RS08005
cell division protein SepF	BEK99_RS07300	SCA_RS04065	SE1039_RS04650	SEQMU2_RS09830	BK815_RS04930	A6V26_RS01090	AA913_RS02840	SXYL_RS08370	BE24_RS03050	SSP_RS08010
YggS family pyridoxal phosphate enzyme	BEK99_RS07305	SCA_RS04060	SE1039_RS04645	SEQMU2_RS09825	BK815_RS04935	A6V26_RS01095	AA913_RS02835	SXYL_RS08375	BE24_RS03045	SSP_RS08015
laccase domain-containing protein	BEK99_RS07310	SCA_RS04055	SE1039_RS04640	SEQMU2_RS09820	BK815_RS04940	A6V26_RS01100	AA913_RS02830	SXYL_RS08380	BE24_RS03040	SSP_RS08020
cell division protein FtsZ	BEK99_RS07315	SCA_RS04050	SE1039_RS04635	SEQMU2_RS09815	BK815_RS04945	A6V26_RS01105	AA913_RS02825	SXYL_RS08385	BE24_RS03035	SSP_RS08025
cell division protein FtsA	BEK99_RS07320	SCA_RS04045	SE1039_RS04630	SEQMU2_RS09810	BK815_RS04950	A6V26_RS01110	AA913_RS02820	SXYL_RS08390	BE24_RS03030	SSP_RS08030
hypothetical protein	BEK99_RS07325	SCA_RS04040	SE1039_RS04625	SEQMU2_RS09805	BK815_RS04955	A6V26_RS01115	AA913_RS02815	SXYL_RS08395	BE24_RS03025	SSP_RS08035
UDP-N-acetylmuramoyl-L-alanine--D-glutamate ligase	BEK99_RS07330	SCA_RS04035	SE1039_RS04620	SEQMU2_RS09800	BK815_RS04960	A6V26_RS01120	AA913_RS02810	SXYL_RS08400	BE24_RS03020	SSP_RS08040
phospho-N-acetylmuramoyl-pentapeptide- transferase	BEK99_RS07335	SCA_RS04030	SE1039_RS04615	SEQMU2_RS09795	BK815_RS04965	A6V26_RS01125	AA913_RS02805	SXYL_RS08405	BE24_RS03015	SSP_RS08045
penicillin-binding protein	BEK99_RS07340	SCA_RS04025	SE1039_RS04610	SEQMU2_RS09790	BK815_RS04970	A6V26_RS01130	AA913_RS02800	SXYL_RS08410	BE24_RS03010	SSP_RS08050
cell division protein FtsL	BEK99_RS07345	SCA_RS04020	SE1039_RS04605	SEQMU2_RS09785	BK815_RS04975	A6V26_RS01135	AA913_RS02795	SXYL_RS08415	BE24_RS03005	SSP_RS08055
16S rRNA (cytosine(1402)-N(4))-methyltransferase	BEK99_RS07350	SCA_RS04015	SE1039_RS04600	SEQMU2_RS09780	BK815_RS04980	A6V26_RS01140	AA913_RS02790	SXYL_RS08420	BE24_RS03000	SSP_RS08060
cell division/cell wall cluster transcriptional repressor MraZ	BEK99_RS07355	SCA_RS04010	SE1039_RS04595	SEQMU2_RS09775	BK815_RS04985	A6V26_RS01145	AA913_RS02785	SXYL_RS08425	BE24_RS02995	SSP_RS08065
hypothetical protein	BEK99_RS07365	SCA_RS04000	SE1039_RS04585	SEQMU2_RS09765	BK815_RS04995	A6V26_RS01155	AA913_RS02775	SXYL_RS08435	BE24_RS02985	SSP_RS08075
noncanonical pyrimidine nucleotidase YjjG family	BEK99_RS07370	SCA_RS03995	SE1039_RS04580	SEQMU2_RS09760	BK815_RS05000	A6V26_RS01160	AA913_RS02770	SXYL_RS08440	BE24_RS02980	SSP_RS08080
hemolytic protein	BEK99_RS07385	SCA_RS12655	SE1039_RS13570	SEQMU2_RS09755	BK815_RS05005	A6V26_RS01165	AA913_RS02760	SXYL_RS13290	BE24_RS13605	SSP_RS08085

Product	*S. carnosus*		*S. equorum*		*S. succinus*			*S. xylosus*		*S. saprophyticus*
	**JCM 6069**	**TM300**	**KS1039**	**Mu2**	**14BME20**	**CSM 77**	**DSM 14617**	**C2a**	**HKUOPL8**	**ATCC 15305**
GCN5 family N-acetyltransferase	BEK99_RS07435	SCA_RS03930	SE1039_RS03250	SEQMU2_RS08460	BK815_RS06270	A6V26_RS02415	AA913_RS08505	SXYL_RS09995	BE24_RS01730	SSP_RS09480
nucleoside-triphosphate diphosphatase	BEK99_RS07460	SCA_RS03905	SE1039_RS04505	SEQMU2_RS09715	BK815_RS05065	A6V26_RS01205	AA913_RS02725	SXYL_RS08805	BE24_RS02925	SSP_RS08285
glutamate racemase	BEK99_RS07465	SCA_RS03900	SE1039_RS04500	SEQMU2_RS09710	BK815_RS05070	A6V26_RS01210	AA913_RS02720	SXYL_RS08810	BE24_RS02920	SSP_RS08290
succinate dehydrogenase iron-sulfur subunit	BEK99_RS07480	SCA_RS03885	SE1039_RS04495	SEQMU2_RS09705	BK815_RS05075	A6V26_RS01215	AA913_RS02715	SXYL_RS08815	BE24_RS02915	SSP_RS08295
succinate dehydrogenase flavoprotein subunit	BEK99_RS07485	SCA_RS03880	SE1039_RS04490	SEQMU2_RS09700	BK815_RS05080	A6V26_RS01220	AA913_RS02710	SXYL_RS08820	BE24_RS02910	SSP_RS08300
succinate dehydrogenase cytochrome B558	BEK99_RS07490	SCA_RS03875	SE1039_RS04485	SEQMU2_RS09695	BK815_RS05085	A6V26_RS01225	AA913_RS02705	SXYL_RS08825	BE24_RS02905	SSP_RS08305
excinuclease ABC subunit C	BEK99_RS07495	SCA_RS03870	SE1039_RS04480	SEQMU2_RS09690	BK815_RS05090	A6V26_RS01230	AA913_RS02700	SXYL_RS08830	BE24_RS02900	SSP_RS08310
thiol reductase thioredoxin	BEK99_RS07500	SCA_RS03865	SE1039_RS04475	SEQMU2_RS09685	BK815_RS05095	A6V26_RS01235	AA913_RS02695	SXYL_RS08835	BE24_RS02895	SSP_RS08315
endonuclease MutS2	BEK99_RS07505	SCA_RS03860	SE1039_RS04470	SEQMU2_RS09680	BK815_RS05100	A6V26_RS01240	AA913_RS02690	SXYL_RS08840	BE24_RS02890	SSP_RS08320
DNA polymerase/3-5 exonuclease PolX	BEK99_RS07510	SCA_RS03855	SE1039_RS04465	SEQMU2_RS09675	BK815_RS05105	A6V26_RS01245	AA913_RS02685	SXYL_RS08845	BE24_RS02885	SSP_RS08325
hypothetical protein	BEK99_RS07515	SCA_RS03850	SE1039_RS04460	SEQMU2_RS09670	BK815_RS05110	A6V26_RS01250	AA913_RS02680	SXYL_RS08850	BE24_RS02880	SSP_RS08330
cell division protein ZapA	BEK99_RS07520	SCA_RS03845	SE1039_RS04455	SEQMU2_RS09665	BK815_RS05115	A6V26_RS01255	AA913_RS02675	SXYL_RS08855	BE24_RS02875	SSP_RS08335
ribonuclease HIII	BEK99_RS07525	SCA_RS03840	SE1039_RS04450	SEQMU2_RS09660	BK815_RS05120	A6V26_RS01260	AA913_RS02670	SXYL_RS08860	BE24_RS02870	SSP_RS08340
phenylalanine--tRNA ligase subunit alpha	BEK99_RS07535	SCA_RS03830	SE1039_RS04440	SEQMU2_RS09650	BK815_RS05130	A6V26_RS01270	AA913_RS02660	SXYL_RS08870	BE24_RS02860	SSP_RS08350
RNA methyltransferase	BEK99_RS07540	SCA_RS03825	SE1039_RS04435	SEQMU2_RS09645	BK815_RS05135	A6V26_RS01275	AA913_RS02655	SXYL_RS08875	BE24_RS02855	SSP_RS08355
50S ribosomal protein L32	BEK99_RS07545	SCA_RS03820	SE1039_RS04425	SEQMU2_RS09635	BK815_RS05145	A6V26_RS01285	AA913_RS02645	SXYL_RS08885	BE24_RS02845	SSP_RS08365
hypothetical protein	BEK99_RS07555	SCA_RS03810	SE1039_RS04415	SEQMU2_RS09625	BK815_RS05155	A6V26_RS01295	AA913_RS02635	SXYL_RS08895	BE24_RS02835	SSP_RS08375
pantetheine-phosphate adenylyltransferase	BEK99_RS07565	SCA_RS03800	SE1039_RS04410	SEQMU2_RS09620	BK815_RS05160	A6V26_RS01300	AA913_RS02630	SXYL_RS08900	BE24_RS02830	SSP_RS08380
16S rRNA (guanine(966)-N(2))-methyltransferase RsmD	BEK99_RS07570	SCA_RS03795	SE1039_RS04405	SEQMU2_RS09615	BK815_RS05165	A6V26_RS01305	AA913_RS02625	SXYL_RS08905	BE24_RS02825	SSP_RS08385
hypothetical protein	BEK99_RS07575	SCA_RS03790	SE1039_RS04400	SEQMU2_RS09610	BK815_RS05170	A6V26_RS01310	AA913_RS02620	SXYL_RS08910	BE24_RS02820	SSP_RS08390
hypothetical protein	BEK99_RS07580	SCA_RS03785	SE1039_RS04395	SEQMU2_RS09605	BK815_RS05175	A6V26_RS01315	AA913_RS02615	SXYL_RS08915	BE24_RS02815	SSP_RS08395
glycerophosphodiester phosphodiesterase	BEK99_RS07585	SCA_RS03780	SE1039_RS04390	SEQMU2_RS09600	BK815_RS05180	A6V26_RS01320	AA913_RS02610	SXYL_RS08920	BE24_RS02810	SSP_RS08400
hypothetical protein	BEK99_RS07590	SCA_RS03775	SE1039_RS04385	SEQMU2_RS09595	BK815_RS05185	A6V26_RS01325	AA913_RS02605	SXYL_RS08925	BE24_RS02805	SSP_RS08405
SCP-like extracellular protein	BEK99_RS07595	SCA_RS03770	SE1039_RS04380	SEQMU2_RS09590	BK815_RS05190	A6V26_RS01330	AA913_RS02600	SXYL_RS08930	BE24_RS02800	SSP_RS08410
membrane protein	BEK99_RS07600	SCA_RS03765	SE1039_RS04370	SEQMU2_RS09580	BK815_RS05200	A6V26_RS01340	AA913_RS02590	SXYL_RS08940	BE24_RS02790	SSP_RS08420
protoheme IX farnesyltransferase	BEK99_RS07605	SCA_RS03760	SE1039_RS04365	SEQMU2_RS09575	BK815_RS05205	A6V26_RS01345	AA913_RS02585	SXYL_RS08945	BE24_RS02785	SSP_RS08425
heme A synthase	BEK99_RS07610	SCA_RS03755	SE1039_RS04360	SEQMU2_RS09570	BK815_RS05210	A6V26_RS01350	AA913_RS02580	SXYL_RS08950	BE24_RS02780	SSP_RS08430
pyruvate carboxylase	BEK99_RS07615	SCA_RS03750	SE1039_RS04355	SEQMU2_RS09565	BK815_RS05215	A6V26_RS01355	AA913_RS02575	SXYL_RS08955	BE24_RS02775	SSP_RS08435
cell division protein FtsW	BEK99_RS07620	SCA_RS03745	SE1039_RS04350	SEQMU2_RS09560	BK815_RS05220	A6V26_RS01360	AA913_RS02570	SXYL_RS08960	BE24_RS02770	SSP_RS08440
hypothetical protein	BEK99_RS07625	SCA_RS03740	SE1039_RS04345	SEQMU2_RS09555	BK815_RS05225	A6V26_RS01365	AA913_RS02565	SXYL_RS08965	BE24_RS02765	SSP_RS08445
hypothetical protein	BEK99_RS07630	SCA_RS03735	SE1039_RS04340	SEQMU2_RS09550	BK815_RS05230	A6V26_RS01370	AA913_RS02560	SXYL_RS08970	BE24_RS02760	SSP_RS08450

Product	*S. carnosus*		*S. equorum*		*S. succinus*			*S. xylosus*		*S. saprophyticus*
	**JCM 6069**	**TM300**	**KS1039**	**Mu2**	**14BME20**	**CSM 77**	**DSM 14617**	**C2a**	**HKUOPL8**	**ATCC 15305**
translational GTPase TypA	BEK99_RS07640	SCA_RS03725	SE1039_RS04335	SEQMU2_RS09545	BK815_RS05240	A6V26_RS01375	AA913_RS02555	SXYL_RS08975	BE24_RS02755	SSP_RS08455
hypothetical protein	BEK99_RS07645	SCA_RS03720	SE1039_RS04330	SEQMU2_RS09540	BK815_RS05245	A6V26_RS01380	AA913_RS02550	SXYL_RS08980	BE24_RS02750	SSP_RS08460
inositol monophosphatase	BEK99_RS07650	SCA_RS03715	SE1039_RS04325	SEQMU2_RS09535	BK815_RS05250	A6V26_RS01385	AA913_RS02545	SXYL_RS08985	BE24_RS02745	SSP_RS08465
hypothetical protein	BEK99_RS07655	SCA_RS03710	SE1039_RS04320	SEQMU2_RS09530	BK815_RS05255	A6V26_RS01390	AA913_RS02540	SXYL_RS08990	BE24_RS02740	SSP_RS08470
hypothetical protein	BEK99_RS07670	SCA_RS03695	SE1039_RS04310	SEQMU2_RS09520	BK815_RS05270	A6V26_RS01405	AA913_RS02525	SXYL_RS09005	BE24_RS02725	SSP_RS08485
spermidine/putrescine ABC transporter substrate-binding protein	BEK99_RS07675	SCA_RS03690	SE1039_RS04305	SEQMU2_RS09515	BK815_RS05275	A6V26_RS01410	AA913_RS02520	SXYL_RS09010	BE24_RS02720	SSP_RS08490
spermidine/purescine ABC transporter permease	BEK99_RS07680	SCA_RS03685	SE1039_RS04300	SEQMU2_RS09510	BK815_RS05280	A6V26_RS01415	AA913_RS02515	SXYL_RS09015	BE24_RS02715	SSP_RS08495
spermidine/putrescine ABC transporter permease	BEK99_RS07685	SCA_RS03680	SE1039_RS04295	SEQMU2_RS09505	BK815_RS05285	A6V26_RS01420	AA913_RS02510	SXYL_RS09020	BE24_RS02710	SSP_RS08500
spermidine/putrescine ABC transporter ATP-binding protein	BEK99_RS07690	SCA_RS03675	SE1039_RS04290	SEQMU2_RS09500	BK815_RS05290	A6V26_RS01425	AA913_RS02505	SXYL_RS09025	BE24_RS02705	SSP_RS08505
hypothetical protein	BEK99_RS07700	SCA_RS03665	SE1039_RS04285	SEQMU2_RS09495	BK815_RS05300	A6V26_RS01435	AA913_RS02495	SXYL_RS09035	BE24_RS02695	SSP_RS08515
dihydrolipoyl dehydrogenase	BEK99_RS07705	SCA_RS03660	SE1039_RS04280	SEQMU2_RS09490	BK815_RS05305	A6V26_RS01440	AA913_RS02490	SXYL_RS09040	BE24_RS02690	SSP_RS08520
branched-chain alpha-keto acid dehydrogenase subunit E2	BEK99_RS07710	SCA_RS03655	SE1039_RS04275	SEQMU2_RS09485	BK815_RS05310	A6V26_RS01445	AA913_RS02485	SXYL_RS09045	BE24_RS02685	SSP_RS08525
alpha-ketoacid dehydrogenase subunit beta	BEK99_RS07715	SCA_RS03650	SE1039_RS04270	SEQMU2_RS09480	BK815_RS05315	A6V26_RS01450	AA913_RS02480	SXYL_RS09050	BE24_RS02680	SSP_RS08530
pyruvate dehydrogenase (acetyl-transferring) E1 component subunit alpha	BEK99_RS07720	SCA_RS03645	SE1039_RS04265	SEQMU2_RS09475	BK815_RS05320	A6V26_RS01455	AA913_RS02475	SXYL_RS09055	BE24_RS02675	SSP_RS08535
lipoprotein	BEK99_RS07725	SCA_RS03640	SE1039_RS04260	SEQMU2_RS09470	BK815_RS05325	A6V26_RS01460	AA913_RS02470	SXYL_RS09060	BE24_RS02670	SSP_RS08540
peptide deformylase	BEK99_RS07730	SCA_RS03635	SE1039_RS04255	SEQMU2_RS09465	BK815_RS05330	A6V26_RS01465	AA913_RS02465	SXYL_RS09065	BE24_RS02665	SSP_RS08545
hypothetical protein	BEK99_RS07740	SCA_RS03625	SE1039_RS04245	SEQMU2_RS09455	BK815_RS05340	A6V26_RS01475	AA913_RS02455	SXYL_RS09075	BE24_RS02655	SSP_RS08555
ribonuclease J	BEK99_RS07745	SCA_RS03620	SE1039_RS04240	SEQMU2_RS09450	BK815_RS05345	A6V26_RS01480	AA913_RS02450	SXYL_RS09080	BE24_RS02650	SSP_RS08560
potassium transporter Trk	BEK99_RS07765	SCA_RS03600	SE1039_RS04235	SEQMU2_RS09445	BK815_RS05350	A6V26_RS01485	AA913_RS02445	SXYL_RS09085	BE24_RS02645	SSP_RS08570
cytochrome D ubiquinol oxidase subunit II	BEK99_RS07770	SCA_RS03595	SE1039_RS04230	SEQMU2_RS09440	BK815_RS05355	A6V26_RS01490	AA913_RS02440	SXYL_RS09090	BE24_RS02640	SSP_RS08575
cytochrome ubiquinol oxidase subunit I	BEK99_RS07775	SCA_RS03590	SE1039_RS04225	SEQMU2_RS09435	BK815_RS05360	A6V26_RS01495	AA913_RS02435	SXYL_RS09095	BE24_RS02635	SSP_RS08580
NrdH-redoxin	BEK99_RS07780	SCA_RS03585	SE1039_RS04220	SEQMU2_RS09430	BK815_RS05365	A6V26_RS01500	AA913_RS02430	SXYL_RS09100	BE24_RS02630	SSP_RS08585
phosphoenolpyruvate--protein phosphotransferase	BEK99_RS07785	SCA_RS03580	SE1039_RS04215	SEQMU2_RS09425	BK815_RS05370	A6V26_RS01505	AA913_RS02425	SXYL_RS09105	BE24_RS02625	SSP_RS08590
phosphocarrier protein HPr	BEK99_RS07790	SCA_RS03575	SE1039_RS04210	SEQMU2_RS09420	BK815_RS05375	A6V26_RS01510	AA913_RS02420	SXYL_RS09110	BE24_RS02620	SSP_RS08595
hypothetical protein	BEK99_RS07795	SCA_RS03570	SE1039_RS04205	SEQMU2_RS09415	BK815_RS05380	A6V26_RS01515	AA913_RS02415	SXYL_RS09115	BE24_RS02615	SSP_RS08600
hypothetical protein	BEK99_RS07805	SCA_RS03560	SE1039_RS04195	SEQMU2_RS09405	BK815_RS05390	A6V26_RS01525	AA913_RS02405	SXYL_RS09125	BE24_RS02605	SSP_RS08610
thiamine ABC transporter permease	BEK99_RS07810	SCA_RS03555	SE1039_RS04190	SEQMU2_RS09400	BK815_RS05395	A6V26_RS01530	AA913_RS02400	SXYL_RS09130	BE24_RS02600	SSP_RS08615
energy-coupling factor transporter transmembrane protein EcfT	BEK99_RS07820	SCA_RS03545	SE1039_RS04180	SEQMU2_RS09390	BK815_RS05405	A6V26_RS01540	AA913_RS02390	SXYL_RS09140	BE24_RS02590	SSP_RS08625
phosphoribosylamine--glycine ligase	BEK99_RS07830	SCA_RS03535	SE1039_RS04175	SEQMU2_RS09385	BK815_RS05410	A6V26_RS01545	AA913_RS02385	SXYL_RS09145	BE24_RS02585	SSP_RS08630
bifunctional phosphoribosylaminoimidazolecarboxamide formyltransferase/IMP cyclohydrolase	BEK99_RS07835	SCA_RS03530	SE1039_RS04170	SEQMU2_RS09380	BK815_RS05415	A6V26_RS01550	AA913_RS02380	SXYL_RS09150	BE24_RS02580	SSP_RS08635

Product	*S. carnosus*		*S. equorum*		*S. succinus*			*S. xylosus*		*S. saprophyticus*
	**JCM 6069**	**TM300**	**KS1039**	**Mu2**	**14BME20**	**CSM 77**	**DSM 14617**	**C2a**	**HKUOPL8**	**ATCC 15305**
phosphoribosylglycinamide formyltransferase	BEK99_RS07840	SCA_RS03525	SE1039_RS04165	SEQMU2_RS09375	BK815_RS05420	A6V26_RS01555	AA913_RS02375	SXYL_RS09155	BE24_RS02575	SSP_RS08640
phosphoribosylformylglycinamidine cyclo-ligase	BEK99_RS07845	SCA_RS03520	SE1039_RS04160	SEQMU2_RS09370	BK815_RS05425	A6V26_RS01560	AA913_RS02370	SXYL_RS09160	BE24_RS02570	SSP_RS08645
amidophosphoribosyltransferase	BEK99_RS07850	SCA_RS03515	SE1039_RS04155	SEQMU2_RS09365	BK815_RS05430	A6V26_RS01565	AA913_RS02365	SXYL_RS09165	BE24_RS02565	SSP_RS08650
phosphoribosylformylglycinamidine synthase II	BEK99_RS07855	SCA_RS03510	SE1039_RS04150	SEQMU2_RS09360	BK815_RS05435	A6V26_RS01570	AA913_RS02360	SXYL_RS09170	BE24_RS02560	SSP_RS08655
phosphoribosylformylglycinamidine synthase I	BEK99_RS07860	SCA_RS03505	SE1039_RS04145	SEQMU2_RS09355	BK815_RS05440	A6V26_RS01575	AA913_RS02355	SXYL_RS09175	BE24_RS02555	SSP_RS08660
phosphoribosylformylglycinamidine synthase	BEK99_RS07865	SCA_RS03500	SE1039_RS04140	SEQMU2_RS09350	BK815_RS05445	A6V26_RS01580	AA913_RS02350	SXYL_RS09180	BE24_RS02550	SSP_RS08665
phosphoribosylaminoimidazolesuccinocarboxamide synthase	BEK99_RS07870	SCA_RS03495	SE1039_RS04135	SEQMU2_RS09345	BK815_RS05450	A6V26_RS01585	AA913_RS02345	SXYL_RS09185	BE24_RS02545	SSP_RS08670
5-(carboxyamino)imidazole ribonucleotide synthase	BEK99_RS07875	SCA_RS03490	SE1039_RS04130	SEQMU2_RS09340	BK815_RS05455	A6V26_RS01590	AA913_RS02340	SXYL_RS09190	BE24_RS02540	SSP_RS08675
5-(carboxyamino)imidazole ribonucleotide mutase	BEK99_RS07880	SCA_RS03485	SE1039_RS04125	SEQMU2_RS09335	BK815_RS05460	A6V26_RS01595	AA913_RS02335	SXYL_RS09195	BE24_RS02535	SSP_RS08680
bifunctional 5,10-methylene-tetrahydrofolate dehydrogenase/5,10-methylene-tetrahydrofolate cyclohydrolase	BEK99_RS07885	SCA_RS03480	SE1039_RS04120	SEQMU2_RS09330	BK815_RS05465	A6V26_RS01600	AA913_RS02330	SXYL_RS09200	BE24_RS02530	SSP_RS08685
peptidase M28	BEK99_RS07890	SCA_RS03475	SE1039_RS10820	SEQMU2_RS02700	BK815_RS11770	A6V26_RS07570	AA913_RS01275	SXYL_RS02320	BE24_RS09465	SSP_RS02130
nitric oxide dioxygenase	BEK99_RS07895	SCA_RS03470	SE1039_RS00395	SEQMU2_RS05900	BK815_RS08790	A6V26_RS04625	AA913_RS07045	SXYL_RS09205	BE24_RS02525	SSP_RS08690
hypothetical protein	BEK99_RS07900	SCA_RS03465	SE1039_RS04110	SEQMU2_RS09320	BK815_RS05470	A6V26_RS01605	AA913_RS02325	SXYL_RS09210	BE24_RS02520	SSP_RS08695
cytochrome aa3 quinol oxidase subunit I	BEK99_RS07910	SCA_RS03455	SE1039_RS04100	SEQMU2_RS09310	BK815_RS05480	A6V26_RS01615	AA913_RS02315	SXYL_RS09220	BE24_RS02510	SSP_RS08705
cytochrome aa3 quinol oxidase subunit III	BEK99_RS07915	SCA_RS03450	SE1039_RS04095	SEQMU2_RS09305	BK815_RS05485	A6V26_RS01620	AA913_RS02310	SXYL_RS09225	BE24_RS02505	SSP_RS08710
cytochrome aa3 quinol oxidase subunit IV	BEK99_RS07920	SCA_RS03445	SE1039_RS04090	SEQMU2_RS09300	BK815_RS05490	A6V26_RS01625	AA913_RS02305	SXYL_RS09230	BE24_RS02500	SSP_RS08715
ribonucleoside hydrolase RihC	BEK99_RS07925	SCA_RS03440	SE1039_RS04085	SEQMU2_RS09295	BK815_RS05495	A6V26_RS01630	AA913_RS02300	SXYL_RS09235	BE24_RS02495	SSP_RS08720
phosphoglucomutase	BEK99_RS07990	SCA_RS03375	SE1039_RS04075	SEQMU2_RS09285	BK815_RS05505	A6V26_RS01640	AA913_RS02290	SXYL_RS09245	BE24_RS02485	SSP_RS08730
hypothetical protein	BEK99_RS07995	SCA_RS03370	SE1039_RS04070	SEQMU2_RS09280	BK815_RS05510	A6V26_RS01645	AA913_RS02285	SXYL_RS09250	BE24_RS02480	SSP_RS08735
hypothetical protein	BEK99_RS08000	SCA_RS03365	SE1039_RS04065	SEQMU2_RS09275	BK815_RS05515	A6V26_RS01650	AA913_RS02280	SXYL_RS09255	BE24_RS02475	SSP_RS08740
GNAT family N-acetyltransferase	BEK99_RS08005	SCA_RS03360	SE1039_RS04060	SEQMU2_RS09270	BK815_RS05520	A6V26_RS01655	AA913_RS02275	SXYL_RS09260	BE24_RS02470	SSP_RS08745
MarR family transcriptional regulator	BEK99_RS08015	SCA_RS03350	SE1039_RS04050	SEQMU2_RS09260	BK815_RS05530	A6V26_RS01665	AA913_RS02265	SXYL_RS09270	BE24_RS02460	SSP_RS08755
N-acetyl-L,L-diaminopimelate aminotransferase	BEK99_RS08025	SCA_RS03340	SE1039_RS04040	SEQMU2_RS09250	BK815_RS05540	A6V26_RS01675	AA913_RS02255	SXYL_RS09280	BE24_RS02450	SSP_RS08765
1,4-dihydroxy-2-naphthoyl-CoA synthase	BEK99_RS08035	SCA_RS03330	SE1039_RS04030	SEQMU2_RS09240	BK815_RS05550	A6V26_RS01685	AA913_RS02245	SXYL_RS09290	BE24_RS02440	SSP_RS08775
2-succinyl-6-hydroxy-2, 4-cyclohexadiene-1-carboxylate synthase	BEK99_RS08040	SCA_RS03325	SE1039_RS04025	SEQMU2_RS09235	BK815_RS05555	A6V26_RS01690	AA913_RS02240	SXYL_RS09295	BE24_RS02435	SSP_RS08780
2-succinyl-5-enolpyruvyl-6-hydroxy-3- cyclohexene-1-carboxylic-acid synthase	BEK99_RS08045	SCA_RS03320	SE1039_RS04020	SEQMU2_RS09230	BK815_RS05560	A6V26_RS01695	AA913_RS13120	SXYL_RS09300	BE24_RS02430	SSP_RS08785
isochorismate synthase	BEK99_RS08050	SCA_RS03315	SE1039_RS04015	SEQMU2_RS09225	BK815_RS05565	A6V26_RS01700	AA913_RS13115	SXYL_RS09305	BE24_RS02425	SSP_RS08790
1,4-dihydroxy-2-naphthoate polyprenyltransferase	BEK99_RS08055	SCA_RS03310	SE1039_RS04010	SEQMU2_RS09220	BK815_RS05570	A6V26_RS01705	AA913_RS13110	SXYL_RS09310	BE24_RS02420	SSP_RS08795
N-acetyltransferase	BEK99_RS08060	SCA_RS03305	SE1039_RS04005	SEQMU2_RS09215	BK815_RS05575	A6V26_RS01710	AA913_RS13105	SXYL_RS09315	BE24_RS02415	SSP_RS08800
NINE protein	BEK99_RS08065	SCA_RS03300	SE1039_RS04000	SEQMU2_RS08495	BK815_RS05580	A6V26_RS01715	AA913_RS13100	SXYL_RS09320	BE24_RS02410	SSP_RS09445

Product	*S. carnosus*		*S. equorum*		*S. succinus*			*S. xylosus*		*S. saprophyticus*
	**JCM 6069**	**TM300**	**KS1039**	**Mu2**	**14BME20**	**CSM 77**	**DSM 14617**	**C2a**	**HKUOPL8**	**ATCC 15305**
CAAX protease	BEK99_RS08075	SCA_RS03290	SE1039_RS03900	SEQMU2_RS09130	BK815_RS05620	A6V26_RS01755	AA913_RS13060	SXYL_RS09360	BE24_RS02370	SSP_RS08845
hypothetical protein	BEK99_RS08080	SCA_RS03285	SE1039_RS03905	SEQMU2_RS09135	BK815_RS05615	A6V26_RS01750	AA913_RS13065	SXYL_RS09355	BE24_RS02375	SSP_RS08840
lipoate--protein ligase A	BEK99_RS08085	SCA_RS03280	SE1039_RS03910	SEQMU2_RS09140	BK815_RS05610	A6V26_RS01745	AA913_RS13070	SXYL_RS09350	BE24_RS02380	SSP_RS08835
hypothetical protein	BEK99_RS08090	SCA_RS03275	SE1039_RS03915	SEQMU2_RS09145	BK815_RS05605	A6V26_RS01740	AA913_RS13075	SXYL_RS09345	BE24_RS02385	SSP_RS08830
ATP phosphoribosyltransferase regulatory subunit	BEK99_RS08130	SCA_RS03235	SE1039_RS10850	SEQMU2_RS02730	BK815_RS11715	A6V26_RS07625	AA913_RS01330	SXYL_RS02290	BE24_RS09495	SSP_RS02100
ATP phosphoribosyltransferase	BEK99_RS08135	SCA_RS03230	SE1039_RS10855	SEQMU2_RS02735	BK815_RS11710	A6V26_RS07630	AA913_RS01335	SXYL_RS02285	BE24_RS09500	SSP_RS02095
histidinol dehydrogenase	BEK99_RS08140	SCA_RS03225	SE1039_RS10860	SEQMU2_RS02740	BK815_RS11705	A6V26_RS07635	AA913_RS01340	SXYL_RS02280	BE24_RS09505	SSP_RS02090
histidinol-phosphate aminotransferase	BEK99_RS08145	SCA_RS03220	SE1039_RS10865	SEQMU2_RS02745	BK815_RS11700	A6V26_RS07640	AA913_RS01345	SXYL_RS02275	BE24_RS09510	SSP_RS02085
imidazoleglycerol-phosphate dehydratase	BEK99_RS08150	SCA_RS03215	SE1039_RS10870	SEQMU2_RS02750	BK815_RS11695	A6V26_RS07645	AA913_RS01350	SXYL_RS02270	BE24_RS09515	SSP_RS02080
imidazole glycerol phosphate synthase subunit HisH	BEK99_RS08155	SCA_RS03210	SE1039_RS10875	SEQMU2_RS02755	BK815_RS11690	A6V26_RS07650	AA913_RS01355	SXYL_RS02265	BE24_RS09520	SSP_RS02075
1-(5-phosphoribosyl)-5-((5-phosphoribosylamino)methylideneamino)imidazole-4-carboxamide isomerase	BEK99_RS08160	SCA_RS03205	SE1039_RS10880	SEQMU2_RS02760	BK815_RS11685	A6V26_RS07655	AA913_RS01360	SXYL_RS02260	BE24_RS09525	SSP_RS02070
imidazole glycerol phosphate synthase subunit HisF	BEK99_RS08165	SCA_RS03200	SE1039_RS10885	SEQMU2_RS02765	BK815_RS11680	A6V26_RS07660	AA913_RS01365	SXYL_RS02255	BE24_RS09530	SSP_RS02065
bifunctional phosphoribosyl-AMP cyclohydrolase/phosphoribosyl-ATP diphosphatase	BEK99_RS08170	SCA_RS03195	SE1039_RS10890	SEQMU2_RS02770	BK815_RS11675	A6V26_RS07665	AA913_RS01370	SXYL_RS02250	BE24_RS09535	SSP_RS02060
Ktr system potassium uptake protein D	BEK99_RS08185	SCA_RS03180	SE1039_RS03890	SEQMU2_RS09120	BK815_RS05630	A6V26_RS01765	AA913_RS13050	SXYL_RS09370	BE24_RS02360	SSP_RS08855
hypothetical protein	BEK99_RS08195	SCA_RS03170	SE1039_RS03880	SEQMU2_RS09110	BK815_RS05640	A6V26_RS01775	AA913_RS13040	SXYL_RS09380	BE24_RS02350	SSP_RS08865
peptide chain release factor 3	BEK99_RS08200	SCA_RS03165	SE1039_RS03875	SEQMU2_RS09105	BK815_RS05645	A6V26_RS01780	AA913_RS13035	SXYL_RS09385	BE24_RS02345	SSP_RS08870
UDP-N-acetylmuramoylalanyl-D-glutamate--L- lysine ligase	BEK99_RS08210	SCA_RS03155	SE1039_RS03865	SEQMU2_RS09095	BK815_RS05655	A6V26_RS01790	AA913_RS13025	SXYL_RS09395	BE24_RS02335	SSP_RS08880
diglucosyl diacylglycerol synthase	BEK99_RS08215	SCA_RS03150	SE1039_RS03860	SEQMU2_RS09090	BK815_RS05660	A6V26_RS01795	AA913_RS13020	SXYL_RS09400	BE24_RS02330	SSP_RS08885
MFS transporter	BEK99_RS08220	SCA_RS03145	SE1039_RS03855	SEQMU2_RS09085	BK815_RS05665	A6V26_RS01800	AA913_RS13015	SXYL_RS09405	BE24_RS02325	SSP_RS08890
hypothetical protein	BEK99_RS08225	SCA_RS03140	SE1039_RS03850	SEQMU2_RS09080	BK815_RS05670	A6V26_RS01805	AA913_RS13010	SXYL_RS09410	BE24_RS02320	SSP_RS08895
esterase family protein	BEK99_RS08230	SCA_RS03135	SE1039_RS03845	SEQMU2_RS09075	BK815_RS05675	A6V26_RS01810	AA913_RS13005	SXYL_RS09415	BE24_RS02315	SSP_RS08900
AI-2E family transporter	BEK99_RS08240	SCA_RS03125	SE1039_RS03835	SEQMU2_RS09065	BK815_RS05685	A6V26_RS01820	AA913_RS09230	SXYL_RS09425	BE24_RS02305	SSP_RS08910
enoyl-[acyl-carrier-protein] reductase	BEK99_RS08250	SCA_RS03115	SE1039_RS03830	SEQMU2_RS09060	BK815_RS05690	A6V26_RS01825	AA913_RS09225	SXYL_RS09430	BE24_RS02300	SSP_RS08915
sodium:proton antiporter	BEK99_RS08255	SCA_RS03110	SE1039_RS03825	SEQMU2_RS09055	BK815_RS05695	A6V26_RS01830	AA913_RS09220	SXYL_RS09435	BE24_RS02295	SSP_RS08920
magnesium transporter	BEK99_RS08260	SCA_RS03105	SE1039_RS03820	SEQMU2_RS09050	BK815_RS05700	A6V26_RS01835	AA913_RS09215	SXYL_RS09440	BE24_RS02290	SSP_RS08925
RNA pseudouridine synthase	BEK99_RS08265	SCA_RS03100	SE1039_RS03815	SEQMU2_RS09045	BK815_RS05705	A6V26_RS01840	AA913_RS09210	SXYL_RS09445	BE24_RS02285	SSP_RS08930
NAD(+) kinase	BEK99_RS08270	SCA_RS03095	SE1039_RS03810	SEQMU2_RS09040	BK815_RS05710	A6V26_RS01845	AA913_RS09205	SXYL_RS09450	BE24_RS02280	SSP_RS08935
GTP pyrophosphokinase	BEK99_RS08275	SCA_RS03090	SE1039_RS03805	SEQMU2_RS09035	BK815_RS05715	A6V26_RS01850	AA913_RS09200	SXYL_RS09455	BE24_RS02275	SSP_RS08940
hypothetical protein	BEK99_RS08280	SCA_RS03085	SE1039_RS03800	SEQMU2_RS09030	BK815_RS05720	A6V26_RS01855	AA913_RS09195	SXYL_RS09460	BE24_RS02270	SSP_RS08945
adenylate cyclase	BEK99_RS08285	SCA_RS03080	SE1039_RS03795	SEQMU2_RS09025	BK815_RS05725	A6V26_RS01860	AA913_RS09190	SXYL_RS09465	BE24_RS02265	SSP_RS08950

Product	*S. carnosus*		*S. equorum*		*S. succinus*			*S. xylosus*		*S. saprophyticus*
	**JCM 6069**	**TM300**	**KS1039**	**Mu2**	**14BME20**	**CSM 77**	**DSM 14617**	**C2a**	**HKUOPL8**	**ATCC 15305**
globin	BEK99_RS08290	SCA_RS03075	SE1039_RS03790	SEQMU2_RS09020	BK815_RS05730	A6V26_RS01865	AA913_RS09185	SXYL_RS09470	BE24_RS02260	SSP_RS08955
hypothetical protein	BEK99_RS08295	SCA_RS03070	SE1039_RS03785	SEQMU2_RS09015	BK815_RS05735	A6V26_RS01870	AA913_RS09180	SXYL_RS09475	BE24_RS02255	SSP_RS08960
oligoendopeptidase F	BEK99_RS08310	SCA_RS03055	SE1039_RS03775	SEQMU2_RS09005	BK815_RS05745	A6V26_RS01880	AA913_RS09170	SXYL_RS09480	BE24_RS02250	SSP_RS08970
adaptor protein MecA	BEK99_RS08315	SCA_RS03050	SE1039_RS03765	SEQMU2_RS08995	BK815_RS05755	A6V26_RS01890	AA913_RS09160	SXYL_RS09490	BE24_RS02240	SSP_RS08980
transcriptional regulator Spx	BEK99_RS08320	SCA_RS03045	SE1039_RS03760	SEQMU2_RS08990	BK815_RS05760	A6V26_RS01895	AA913_RS09155	SXYL_RS09495	BE24_RS02235	SSP_RS08985
tryptophan--tRNA ligase	BEK99_RS08325	SCA_RS03040	SE1039_RS03755	SEQMU2_RS08985	BK815_RS05765	A6V26_RS01900	AA913_RS09150	SXYL_RS09500	BE24_RS02230	SSP_RS08990
LLM class flavin-dependent oxidoreductase	BEK99_RS08335	SCA_RS03030	SE1039_RS10440	SEQMU2_RS02300	BK815_RS12080	A6V26_RS07260	AA913_RS00960	SXYL_RS02735	BE24_RS09040	SSP_RS02520
ABC transporter ATP-binding protein	BEK99_RS08345	SCA_RS03020	SE1039_RS03745	SEQMU2_RS08975	BK815_RS05775	A6V26_RS01910	AA913_RS09140	SXYL_RS09510	BE24_RS02220	SSP_RS09000
ABC transporter ATP-binding protein	BEK99_RS08350	SCA_RS03015	SE1039_RS03740	SEQMU2_RS08970	BK815_RS05780	A6V26_RS01915	AA913_RS09135	SXYL_RS09515	BE24_RS02215	SSP_RS09005
ABC transporter permease	BEK99_RS08355	SCA_RS03010	SE1039_RS03735	SEQMU2_RS08965	BK815_RS05785	A6V26_RS01920	AA913_RS09130	SXYL_RS09520	BE24_RS02210	SSP_RS09010
ABC transporter permease	BEK99_RS08360	SCA_RS03005	SE1039_RS03730	SEQMU2_RS08960	BK815_RS05790	A6V26_RS01925	AA913_RS09125	SXYL_RS09525	BE24_RS02205	SSP_RS09015
beta-ketoacyl-[acyl-carrier-protein] synthase II	BEK99_RS08375	SCA_RS02990	SE1039_RS03715	SEQMU2_RS08945	BK815_RS05805	A6V26_RS01935	AA913_RS09115	SXYL_RS09540	BE24_RS02195	SSP_RS09030
ketoacyl-ACP synthase III	BEK99_RS08380	SCA_RS02985	SE1039_RS03710	SEQMU2_RS08940	BK815_RS05810	A6V26_RS01940	AA913_RS09110	SXYL_RS09545	BE24_RS02190	SSP_RS09035
DUF2929 domain-containing protein	BEK99_RS08385	SCA_RS02980	SE1039_RS03705	SEQMU2_RS08935	BK815_RS05815	A6V26_RS01945	AA913_RS09105	SXYL_RS09550	BE24_RS02185	SSP_RS09040
ATP-dependent chaperone ClpB	BEK99_RS08390	SCA_RS02975	SE1039_RS03700	SEQMU2_RS08930	BK815_RS05820	A6V26_RS01950	AA913_RS09100	SXYL_RS09555	BE24_RS02180	SSP_RS09045
DNA methyltransferase	BEK99_RS08400	SCA_RS02965	SE1039_RS03690	SEQMU2_RS08915	BK815_RS05830	A6V26_RS01960	AA913_RS09090	SXYL_RS09565	BE24_RS02170	SSP_RS09055
HAD family phosphatase	BEK99_RS08405	SCA_RS02960	SE1039_RS03685	SEQMU2_RS08910	BK815_RS05835	A6V26_RS01965	AA913_RS09085	SXYL_RS09570	BE24_RS02165	SSP_RS09060
CoA-disulfide reductase	BEK99_RS08410	SCA_RS02955	SE1039_RS03680	SEQMU2_RS08905	BK815_RS05840	A6V26_RS01970	AA913_RS09080	SXYL_RS09575	BE24_RS02160	SSP_RS09065
hypothetical protein	BEK99_RS08415	SCA_RS02950	SE1039_RS03675	SEQMU2_RS08900	BK815_RS05855	A6V26_RS01985	AA913_RS09065	SXYL_RS09580	BE24_RS02155	SSP_RS09070
hypothetical protein	BEK99_RS08420	SCA_RS02945	SE1039_RS03670	SEQMU2_RS08895	BK815_RS05860	A6V26_RS01990	AA913_RS09060	SXYL_RS09585	BE24_RS02150	SSP_RS09075
helicase-exonuclease AddAB subunit AddB	BEK99_RS08430	SCA_RS02935	SE1039_RS03655	SEQMU2_RS08880	BK815_RS05875	A6V26_RS02005	AA913_RS09045	SXYL_RS09600	BE24_RS02135	SSP_RS09090
signal peptidase I	BEK99_RS08435	SCA_RS02930	SE1039_RS03650	SEQMU2_RS08875	BK815_RS05880	A6V26_RS02010	AA913_RS09040	SXYL_RS09605	BE24_RS02130	SSP_RS09095
signal peptidase I	BEK99_RS08440	SCA_RS02925	SE1039_RS03645	SEQMU2_RS08870	BK815_RS05885	A6V26_RS02015	AA913_RS09035	SXYL_RS09610	BE24_RS02125	SSP_RS09100
TVP38/TMEM64 family protein	BEK99_RS08445	SCA_RS02920	SE1039_RS03640	SEQMU2_RS08865	BK815_RS05890	A6V26_RS02020	AA913_RS09030	SXYL_RS09615	BE24_RS02120	SSP_RS09105
glucose-6-phosphate isomerase	BEK99_RS08450	SCA_RS02915	SE1039_RS03635	SEQMU2_RS08860	BK815_RS05895	A6V26_RS02025	AA913_RS09025	SXYL_RS09620	BE24_RS02115	SSP_RS09110
argininosuccinate synthase	BEK99_RS08455	SCA_RS02910	SE1039_RS03630	SEQMU2_RS08855	BK815_RS05900	A6V26_RS02030	AA913_RS09020	SXYL_RS09625	BE24_RS02110	SSP_RS09115
argininosuccinate lyase	BEK99_RS08460	SCA_RS02905	SE1039_RS03625	SEQMU2_RS08850	BK815_RS05905	A6V26_RS02035	AA913_RS09015	SXYL_RS09630	BE24_RS02105	SSP_RS09120
glutamate dehydrogenase	BEK99_RS08470	SCA_RS02895	SE1039_RS03600	SEQMU2_RS08825	BK815_RS05915	A6V26_RS02045	AA913_RS09005	SXYL_RS09640	BE24_RS02095	SSP_RS09130
ornithine--oxo-acid transaminase	BEK99_RS08475	SCA_RS02890	SE1039_RS03595	SEQMU2_RS08820	BK815_RS05920	A6V26_RS02050	AA913_RS09000	SXYL_RS09645	BE24_RS02090	SSP_RS09135
L-glutamate gamma-semialdehyde dehydrogenase	BEK99_RS08480	SCA_RS02885	SE1039_RS11330	SEQMU2_RS03205	BK815_RS11215	A6V26_RS08150	AA913_RS01855	SXYL_RS01700	BE24_RS10075	SSP_RS01515
NADH-dependent flavin oxidoreductase	BEK99_RS08485	SCA_RS02880	SE1039_RS03590	SEQMU2_RS08815	BK815_RS05925	A6V26_RS02055	AA913_RS08995	SXYL_RS09650	BE24_RS02085	SSP_RS09140

Product	*S. carnosus*		*S. equorum*		*S. succinus*			*S. xylosus*		*S. saprophyticus*
	**JCM 6069**	**TM300**	**KS1039**	**Mu2**	**14BME20**	**CSM 77**	**DSM 14617**	**C2a**	**HKUOPL8**	**ATCC 15305**
general stress protein	BEK99_RS08490	SCA_RS02875	SE1039_RS03585	SEQMU2_RS08810	BK815_RS05930	A6V26_RS02060	AA913_RS08990	SXYL_RS09655	BE24_RS02080	SSP_RS09145
peptidyl-prolyl cis-trans isomerase	BEK99_RS08505	SCA_RS02860	SE1039_RS03580	SEQMU2_RS08805	BK815_RS05935	A6V26_RS02065	AA913_RS08985	SXYL_RS09660	BE24_RS02075	SSP_RS09150
kinase	BEK99_RS08510	SCA_RS02855	SE1039_RS03575	SEQMU2_RS08800	BK815_RS05940	A6V26_RS02070	AA913_RS08980	SXYL_RS09665	BE24_RS02070	SSP_RS09155
Na+/H+ antiporter subunit A	BEK99_RS08515	SCA_RS02850	SE1039_RS03570	SEQMU2_RS08795	BK815_RS05945	A6V26_RS02075	AA913_RS08975	SXYL_RS09670	BE24_RS02065	SSP_RS09160
monovalent cation/H+ antiporter subunit B	BEK99_RS08520	SCA_RS02845	SE1039_RS03565	SEQMU2_RS08790	BK815_RS05950	A6V26_RS02080	AA913_RS08970	SXYL_RS09675	BE24_RS02060	SSP_RS09165
Na(+)/H(+) antiporter subunit C	BEK99_RS08525	SCA_RS02840	SE1039_RS03560	SEQMU2_RS08785	BK815_RS05955	A6V26_RS02085	AA913_RS08965	SXYL_RS09680	BE24_RS02055	SSP_RS09170
Na+/H+ antiporter subunit D	BEK99_RS08530	SCA_RS02835	SE1039_RS03555	SEQMU2_RS08780	BK815_RS05960	A6V26_RS02090	AA913_RS08960	SXYL_RS09685	BE24_RS02050	SSP_RS09175
Na+/H+ antiporter subunit E	BEK99_RS08535	SCA_RS02830	SE1039_RS03550	SEQMU2_RS08775	BK815_RS05965	A6V26_RS02095	AA913_RS08955	SXYL_RS09690	BE24_RS02045	SSP_RS09180
Na(+)/H(+) antiporter subunit F	BEK99_RS08540	SCA_RS02825	SE1039_RS03545	SEQMU2_RS08770	BK815_RS05970	A6V26_RS02100	AA913_RS08950	SXYL_RS09695	BE24_RS02040	SSP_RS09185
Na+/H+ antiporter subunit G1	BEK99_RS08545	SCA_RS02820	SE1039_RS03540	SEQMU2_RS08765	BK815_RS05975	A6V26_RS02105	AA913_RS08945	SXYL_RS09700	BE24_RS02035	SSP_RS09190
pyridine nucleotide-disulfide oxidoreductase	BEK99_RS08550	SCA_RS02815	SE1039_RS03535	SEQMU2_RS08760	BK815_RS05980	A6V26_RS02110	AA913_RS08940	SXYL_RS09705	BE24_RS02030	SSP_RS09195
thioesterase	BEK99_RS08555	SCA_RS02810	SE1039_RS03530	SEQMU2_RS08755	BK815_RS05985	A6V26_RS02115	AA913_RS08935	SXYL_RS09710	BE24_RS02020	SSP_RS09200
sodium:proton antiporter	BEK99_RS08560	SCA_RS02805	SE1039_RS03525	SEQMU2_RS08750	BK815_RS05990	A6V26_RS02120	AA913_RS08930	SXYL_RS09715	BE24_RS02015	SSP_RS09205
aminopeptidase	BEK99_RS08565	SCA_RS02800	SE1039_RS03520	SEQMU2_RS08745	BK815_RS05995	A6V26_RS02125	AA913_RS08925	SXYL_RS09720	BE24_RS02010	SSP_RS09210
NADH dehydrogenase family protein	BEK99_RS08570	SCA_RS02795	SE1039_RS03515	SEQMU2_RS08740	BK815_RS06000	A6V26_RS02130	AA913_RS08920	SXYL_RS09725	BE24_RS02005	SSP_RS09215
iron-sulfur cluster assembly accessory protein	BEK99_RS08575	SCA_RS02790	SE1039_RS03510	SEQMU2_RS08735	BK815_RS06005	A6V26_RS02135	AA913_RS08915	SXYL_RS09730	BE24_RS02000	SSP_RS09220
hypothetical protein	BEK99_RS08580	SCA_RS02785	SE1039_RS03505	SEQMU2_RS08730	BK815_RS06010	A6V26_RS02140	AA913_RS08910	SXYL_RS09735	BE24_RS01995	SSP_RS09225
NADH dehydrogenase family protein	BEK99_RS08585	SCA_RS02780	SE1039_RS03500	SEQMU2_RS08725	BK815_RS06015	A6V26_RS02145	AA913_RS08905	SXYL_RS09740	BE24_RS01990	SSP_RS09230
disulfide oxidoreductase	BEK99_RS08595	SCA_RS02770	SE1039_RS03495	SEQMU2_RS08720	BK815_RS06020	A6V26_RS02150	AA913_RS08900	SXYL_RS09745	BE24_RS01985	SSP_RS09235
NifU family protein	BEK99_RS08600	SCA_RS02765	SE1039_RS03490	SEQMU2_RS08715	BK815_RS06025	A6V26_RS02155	AA913_RS08895	SXYL_RS09750	BE24_RS01980	SSP_RS09240
D-alanyl-lipoteichoic acid biosynthesis protein DltD	BEK99_RS08615	SCA_RS02750	SE1039_RS03485	SEQMU2_RS08710	BK815_RS06030	A6V26_RS02175	AA913_RS08875	SXYL_RS09755	BE24_RS01975	SSP_RS09245
D-alanine--poly(phosphoribitol) ligase subunit 2	BEK99_RS08620	SCA_RS02745	SE1039_RS03480	SEQMU2_RS08705	BK815_RS06035	A6V26_RS02180	AA913_RS08870	SXYL_RS09760	BE24_RS01970	SSP_RS09250
D-alanyl-lipoteichoic acid biosynthesis protein DltB	BEK99_RS08625	SCA_RS02740	SE1039_RS03475	SEQMU2_RS08700	BK815_RS06040	A6V26_RS02185	AA913_RS08865	SXYL_RS09765	BE24_RS01965	SSP_RS09255
teichoic acid D-Ala incorporation-associated protein DltX	BEK99_RS08635	SCA_RS02730	SE1039_RS03465	SEQMU2_RS08690	BK815_RS06050	A6V26_RS02195	AA913_RS08855	SXYL_RS09775	BE24_RS01955	SSP_RS09265
D-glycerate dehydrogenase	BEK99_RS08640	SCA_RS02725	SE1039_RS03455	SEQMU2_RS08680	BK815_RS06055	A6V26_RS02200	AA913_RS08850	SXYL_RS09780	BE24_RS01950	SSP_RS09270
TIGR01457 family HAD-type hydrolase	BEK99_RS08645	SCA_RS02720	SE1039_RS03450	SEQMU2_RS08675	BK815_RS06060	A6V26_RS02205	AA913_RS08845	SXYL_RS09785	BE24_RS01945	SSP_RS09275
DUF86 domain-containing protein	BEK99_RS08650	SCA_RS02715	SE1039_RS03445	SEQMU2_RS08670	BK815_RS06065	A6V26_RS02210	AA913_RS08840	SXYL_RS09790	BE24_RS01940	SSP_RS09280
cytosolic protein	BEK99_RS08655	SCA_RS02710	SE1039_RS03440	SEQMU2_RS08665	BK815_RS06070	A6V26_RS02215	AA913_RS08835	SXYL_RS09795	BE24_RS01935	SSP_RS09285
hypothetical protein	BEK99_RS08660	SCA_RS02705	SE1039_RS03435	SEQMU2_RS08660	BK815_RS06075	A6V26_RS02220	AA913_RS08830	SXYL_RS09800	BE24_RS01930	SSP_RS09290
lipoyl synthase	BEK99_RS08665	SCA_RS02700	SE1039_RS03430	SEQMU2_RS08655	BK815_RS06080	A6V26_RS02225	AA913_RS08825	SXYL_RS09805	BE24_RS01925	SSP_RS09295
anion permease	BEK99_RS08675	SCA_RS02690	SE1039_RS03420	SEQMU2_RS08645	BK815_RS06090	A6V26_RS02235	AA913_RS08815	SXYL_RS09815	BE24_RS01915	SSP_RS09305

Product	*S. carnosus*		*S. equorum*		*S. succinus*			*S. xylosus*		*S. saprophyticus*
	**JCM 6069**	**TM300**	**KS1039**	**Mu2**	**14BME20**	**CSM 77**	**DSM 14617**	**C2a**	**HKUOPL8**	**ATCC 15305**
DUF72 domain-containing protein	BEK99_RS08680	SCA_RS02685	SE1039_RS03415	SEQMU2_RS08640	BK815_RS06095	A6V26_RS02240	AA913_RS08680	SXYL_RS09820	BE24_RS01910	SSP_RS09310
nitronate monooxygenase	BEK99_RS08685	SCA_RS02680	SE1039_RS03410	SEQMU2_RS08635	BK815_RS06100	A6V26_RS02245	AA913_RS08675	SXYL_RS09825	BE24_RS01905	SSP_RS09315
Fe-S cluster assembly protein SufB	BEK99_RS09005	SCA_RS02350	SE1039_RS03400	SEQMU2_RS08625	BK815_RS06110	A6V26_RS02255	AA913_RS08665	SXYL_RS09845	BE24_RS01880	SSP_RS09325
iron-sulfur cluster assembly scaffold protein NifU	BEK99_RS09010	SCA_RS02345	SE1039_RS03395	SEQMU2_RS08620	BK815_RS06115	A6V26_RS02260	AA913_RS08660	SXYL_RS09850	BE24_RS01875	SSP_RS09330
cysteine desulfurase	BEK99_RS09015	SCA_RS02340	SE1039_RS03390	SEQMU2_RS08615	BK815_RS06120	A6V26_RS02265	AA913_RS08655	SXYL_RS09855	BE24_RS01870	SSP_RS09335
Fe-S cluster assembly protein SufD	BEK99_RS09020	SCA_RS02335	SE1039_RS03385	SEQMU2_RS08610	BK815_RS06125	A6V26_RS02270	AA913_RS08650	SXYL_RS09860	BE24_RS01865	SSP_RS09340
ABC transporter ATP-binding protein	BEK99_RS09025	SCA_RS02330	SE1039_RS03380	SEQMU2_RS08605	BK815_RS06130	A6V26_RS02275	AA913_RS08645	SXYL_RS09865	BE24_RS01860	SSP_RS09345
methionine ABC transporter substrate-binding protein	BEK99_RS09030	SCA_RS02325	SE1039_RS03365	SEQMU2_RS08575	BK815_RS06160	A6V26_RS02305	AA913_RS08615	SXYL_RS09885	BE24_RS01840	SSP_RS09365
methionine ABC transporter permease	BEK99_RS09035	SCA_RS02320	SE1039_RS03360	SEQMU2_RS08570	BK815_RS06165	A6V26_RS02310	AA913_RS08610	SXYL_RS09890	BE24_RS01835	SSP_RS09370
methionine ABC transporter ATP-binding protein	BEK99_RS09040	SCA_RS02315	SE1039_RS03355	SEQMU2_RS08565	BK815_RS06170	A6V26_RS02315	AA913_RS08605	SXYL_RS09895	BE24_RS01830	SSP_RS09375
thioredoxin	BEK99_RS09045	SCA_RS02310	SE1039_RS03350	SEQMU2_RS08560	BK815_RS06175	A6V26_RS02320	AA913_RS08600	SXYL_RS09900	BE24_RS01825	SSP_RS09380
topiosmerase	BEK99_RS09050	SCA_RS02305	SE1039_RS03345	SEQMU2_RS08555	BK815_RS06180	A6V26_RS02325	AA913_RS08595	SXYL_RS09905	BE24_RS01820	SSP_RS09385
glycine cleavage system protein H	BEK99_RS09055	SCA_RS02300	SE1039_RS03340	SEQMU2_RS08550	BK815_RS06185	A6V26_RS02330	AA913_RS08590	SXYL_RS09910	BE24_RS01815	SSP_RS09390
arsenate reductase	BEK99_RS09060	SCA_RS02295	SE1039_RS03335	SEQMU2_RS08545	BK815_RS06190	A6V26_RS02335	AA913_RS08585	SXYL_RS09915	BE24_RS01810	SSP_RS09395
thiol reductase thioredoxin	BEK99_RS09065	SCA_RS02290	SE1039_RS03330	SEQMU2_RS08540	BK815_RS06195	A6V26_RS02340	AA913_RS08580	SXYL_RS09920	BE24_RS01805	SSP_RS09400
nitroreductase	BEK99_RS09070	SCA_RS02285	SE1039_RS03325	SEQMU2_RS08535	BK815_RS06200	A6V26_RS02345	AA913_RS08575	SXYL_RS09925	BE24_RS01800	SSP_RS09405
3-dehydroquinase	BEK99_RS09075	SCA_RS02280	SE1039_RS03320	SEQMU2_RS08530	BK815_RS06205	A6V26_RS02350	AA913_RS08570	SXYL_RS09930	BE24_RS01795	SSP_RS09410
hypothetical protein	BEK99_RS09090	SCA_RS02265	SE1039_RS03290	SEQMU2_RS08500	BK815_RS06235	A6V26_RS02380	AA913_RS08540	SXYL_RS09960	BE24_RS01765	SSP_RS09440
SsrA-binding protein	BEK99_RS09155	SCA_RS02205	SE1039_RS03100	SEQMU2_RS08205	BK815_RS06385	A6V26_RS02510	AA913_RS08420	SXYL_RS10085	BE24_RS01645	SSP_RS09545
ribonuclease R	BEK99_RS09160	SCA_RS02200	SE1039_RS03095	SEQMU2_RS08200	BK815_RS06390	A6V26_RS02515	AA913_RS08415	SXYL_RS10090	BE24_RS01640	SSP_RS09550
carboxylesterase	BEK99_RS09165	SCA_RS02195	SE1039_RS03090	SEQMU2_RS08195	BK815_RS06395	A6V26_RS02520	AA913_RS08410	SXYL_RS10095	BE24_RS01635	SSP_RS09555
preprotein translocase subunit SecG	BEK99_RS09170	SCA_RS02190	SE1039_RS03085	SEQMU2_RS08190	BK815_RS06400	A6V26_RS02525	AA913_RS08405	SXYL_RS10100	BE24_RS01630	SSP_RS09560
hypothetical protein	BEK99_RS09175	SCA_RS02185	SE1039_RS03080	SEQMU2_RS08185	BK815_RS06405	A6V26_RS02530	AA913_RS08400	SXYL_RS10105	BE24_RS01625	SSP_RS09565
phosphopyruvate hydratase	BEK99_RS09180	SCA_RS02180	SE1039_RS03075	SEQMU2_RS08180	BK815_RS06410	A6V26_RS02535	AA913_RS08395	SXYL_RS10110	BE24_RS01620	SSP_RS09575
phosphoglycerate mutase (2,3-diphosphoglycerate-independent)	BEK99_RS09185	SCA_RS02175	SE1039_RS03070	SEQMU2_RS08175	BK815_RS06415	A6V26_RS02540	AA913_RS08390	SXYL_RS10115	BE24_RS01615	SSP_RS09580
triose-phosphate isomerase	BEK99_RS09190	SCA_RS02170	SE1039_RS03065	SEQMU2_RS08170	BK815_RS06420	A6V26_RS02545	AA913_RS08385	SXYL_RS10120	BE24_RS01610	SSP_RS09585
phosphoglycerate kinase	BEK99_RS09195	SCA_RS02165	SE1039_RS03060	SEQMU2_RS08165	BK815_RS06425	A6V26_RS02550	AA913_RS08380	SXYL_RS10125	BE24_RS01605	SSP_RS09590
type I glyceraldehyde-3-phosphate dehydrogenase	BEK99_RS09200	SCA_RS02160	SE1039_RS03055	SEQMU2_RS08160	BK815_RS06430	A6V26_RS02555	AA913_RS08375	SXYL_RS10130	BE24_RS01600	SSP_RS09595
transcriptional regulator	BEK99_RS09205	SCA_RS02155	SE1039_RS03050	SEQMU2_RS08155	BK815_RS06435	A6V26_RS02560	AA913_RS08370	SXYL_RS10135	BE24_RS01595	SSP_RS09600
ATP-dependent Clp protease proteolytic subunit	BEK99_RS09225	SCA_RS02135	SE1039_RS03025	SEQMU2_RS08135	BK815_RS06465	A6V26_RS02590	AA913_RS08340	SXYL_RS10170	BE24_RS01565	SSP_RS09630
branched-chain amino acid transport system II carrier protein	BEK99_RS09235	SCA_RS02125	SE1039_RS01040	SEQMU2_RS06625	BK815_RS08250	A6V26_RS04090	AA913_RS06510	SXYL_RS12125	BE24_RS13145	SSP_RS11585

Product	*S. carnosus*		*S. equorum*		*S. succinus*			*S. xylosus*		*S. saprophyticus*
	**JCM 6069**	**TM300**	**KS1039**	**Mu2**	**14BME20**	**CSM 77**	**DSM 14617**	**C2a**	**HKUOPL8**	**ATCC 15305**
DNA-binding protein WhiA	BEK99_RS09245	SCA_RS02115	SE1039_RS03010	SEQMU2_RS07800	BK815_RS06485	A6V26_RS02610	AA913_RS08320	SXYL_RS10180	BE24_RS01555	SSP_RS09770
YvcK family protein	BEK99_RS09250	SCA_RS02110	SE1039_RS03005	SEQMU2_RS07795	BK815_RS06490	A6V26_RS02615	AA913_RS08315	SXYL_RS10185	BE24_RS01550	SSP_RS09775
nucleotide-binding protein	BEK99_RS09255	SCA_RS02105	SE1039_RS03000	SEQMU2_RS07790	BK815_RS06495	A6V26_RS02620	AA913_RS08310	SXYL_RS10190	BE24_RS01545	SSP_RS09780
thioredoxin-disulfide reductase	BEK99_RS09260	SCA_RS02100	SE1039_RS02995	SEQMU2_RS07785	BK815_RS06500	A6V26_RS02625	AA913_RS08305	SXYL_RS10195	BE24_RS01540	SSP_RS09785
hypothetical protein	BEK99_RS09265	SCA_RS02095	SE1039_RS02990	SEQMU2_RS07780	BK815_RS06505	A6V26_RS02630	AA913_RS08300	SXYL_RS10200	BE24_RS01535	SSP_RS09790
acetyltransferase	BEK99_RS09270	SCA_RS02090	SE1039_RS02985	SEQMU2_RS07775	BK815_RS06510	A6V26_RS02635	AA913_RS08295	SXYL_RS10205	BE24_RS01530	SSP_RS09795
prolipoprotein diacylglyceryl transferase	BEK99_RS09275	SCA_RS02085	SE1039_RS02980	SEQMU2_RS07770	BK815_RS06515	A6V26_RS02640	AA913_RS08290	SXYL_RS10210	BE24_RS01525	SSP_RS09800
excinuclease ABC subunit A	BEK99_RS09285	SCA_RS02075	SE1039_RS02970	SEQMU2_RS07760	BK815_RS06525	A6V26_RS02650	AA913_RS08280	SXYL_RS10220	BE24_RS01515	SSP_RS09810
excinuclease ABC subunit B	BEK99_RS09290	SCA_RS02070	SE1039_RS02965	SEQMU2_RS07755	BK815_RS06530	A6V26_RS02655	AA913_RS08275	SXYL_RS10225	BE24_RS01510	SSP_RS09815
hypothetical protein	BEK99_RS09295	SCA_RS02065	SE1039_RS02960	SEQMU2_RS07750	BK815_RS06535	A6V26_RS02660	AA913_RS08270	SXYL_RS10230	BE24_RS01505	SSP_RS09820
hydrolase	BEK99_RS09300	SCA_RS02060	SE1039_RS02955	SEQMU2_RS07745	BK815_RS06540	A6V26_RS02665	AA913_RS08265	SXYL_RS10235	BE24_RS01500	SSP_RS09825
preprotein translocase subunit SecA	BEK99_RS09315	SCA_RS02045	SE1039_RS02940	SEQMU2_RS07730	BK815_RS06555	A6V26_RS02680	AA913_RS08250	SXYL_RS10250	BE24_RS01485	SSP_RS09840
ribosomal subunit interface protein	BEK99_RS09320	SCA_RS02040	SE1039_RS02935	SEQMU2_RS07725	BK815_RS06560	A6V26_RS02685	AA913_RS08245	SXYL_RS10255	BE24_RS01480	SSP_RS09845
DNA/RNA helicase	BEK99_RS09330	SCA_RS02030	SE1039_RS02925	SEQMU2_RS07715	BK815_RS06570	A6V26_RS02695	AA913_RS08235	SXYL_RS10265	BE24_RS01470	SSP_RS09855
DegV family protein	BEK99_RS09335	SCA_RS02025	SE1039_RS02920	SEQMU2_RS07710	BK815_RS06575	A6V26_RS02700	AA913_RS08230	SXYL_RS10270	BE24_RS01465	SSP_RS09860
YigZ family protein	BEK99_RS09340	SCA_RS02020	SE1039_RS02915	SEQMU2_RS07705	BK815_RS06580	A6V26_RS02705	AA913_RS08225	SXYL_RS10275	BE24_RS01460	SSP_RS09865
undecaprenyl-phosphate alpha-N-acetylglucosaminyl 1-phosphate transferase	BEK99_RS09345	SCA_RS02015	SE1039_RS02910	SEQMU2_RS07700	BK815_RS06585	A6V26_RS02710	AA913_RS08220	SXYL_RS10280	BE24_RS01455	SSP_RS09870
membrane protein	BEK99_RS09350	SCA_RS02010	SE1039_RS02905	SEQMU2_RS07695	BK815_RS06590	A6V26_RS02715	AA913_RS08215	SXYL_RS10285	BE24_RS01450	SSP_RS09875
threonine/serine exporter	BEK99_RS09355	SCA_RS02005	SE1039_RS02900	SEQMU2_RS07690	BK815_RS06595	A6V26_RS02720	AA913_RS08210	SXYL_RS10290	BE24_RS01445	SSP_RS09880
membrane protein	BEK99_RS09360	SCA_RS02000	SE1039_RS02895	SEQMU2_RS07685	BK815_RS06600	A6V26_RS02725	AA913_RS08205	SXYL_RS10295	BE24_RS01440	SSP_RS09885
peptidase T	BEK99_RS09365	SCA_RS01995	SE1039_RS02890	SEQMU2_RS07680	BK815_RS06605	A6V26_RS02730	AA913_RS08200	SXYL_RS10300	BE24_RS01435	SSP_RS09890
glycerate kinase	BEK99_RS09370	SCA_RS01990	SE1039_RS02885	SEQMU2_RS07675	BK815_RS06610	A6V26_RS02735	AA913_RS08195	SXYL_RS10305	BE24_RS01430	SSP_RS09895
hypothetical protein	BEK99_RS09375	SCA_RS01985	SE1039_RS02880	SEQMU2_RS07670	BK815_RS06615	A6V26_RS02740	AA913_RS08190	SXYL_RS10310	BE24_RS01425	SSP_RS09900
hypothetical protein	BEK99_RS09380	SCA_RS01980	SE1039_RS02875	SEQMU2_RS07665	BK815_RS06620	A6V26_RS02745	AA913_RS08185	SXYL_RS10315	BE24_RS01420	SSP_RS09905
hypothetical protein	BEK99_RS09395	SCA_RS01965	SE1039_RS02860	SEQMU2_RS07650	BK815_RS06635	A6V26_RS02760	AA913_RS08170	SXYL_RS10330	BE24_RS01405	SSP_RS09920
lipoprotein SstD	BEK99_RS09405	SCA_RS01955	SE1039_RS02850	SEQMU2_RS07640	BK815_RS06645	A6V26_RS02770	AA913_RS08160	SXYL_RS10340	BE24_RS01395	SSP_RS09930
iron ABC transporter ATP-binding protein	BEK99_RS09410	SCA_RS01950	SE1039_RS02845	SEQMU2_RS07635	BK815_RS06650	A6V26_RS02775	AA913_RS08155	SXYL_RS10345	BE24_RS01390	SSP_RS09935
iron ABC transporter permease	BEK99_RS09415	SCA_RS01945	SE1039_RS02840	SEQMU2_RS07630	BK815_RS06655	A6V26_RS02780	AA913_RS08150	SXYL_RS10350	BE24_RS01385	SSP_RS09940
iron ABC transporter permease	BEK99_RS09420	SCA_RS01940	SE1039_RS02835	SEQMU2_RS07625	BK815_RS06660	A6V26_RS02785	AA913_RS08145	SXYL_RS10355	BE24_RS01380	SSP_RS09945
ribonucleotide-diphosphate reductase subunit beta	BEK99_RS09425	SCA_RS01935	SE1039_RS02830	SEQMU2_RS07620	BK815_RS06665	A6V26_RS02790	AA913_RS08140	SXYL_RS10360	BE24_RS01375	SSP_RS09950
ribonucleotide-diphosphate reductase	BEK99_RS09430	SCA_RS01930	SE1039_RS02825	SEQMU2_RS07615	BK815_RS06670	A6V26_RS02795	AA913_RS08135	SXYL_RS10365	BE24_RS01370	SSP_RS09955

Product	*S. carnosus*		*S. equorum*		*S. succinus*			*S. xylosus*		*S. saprophyticus*
	**JCM 6069**	**TM300**	**KS1039**	**Mu2**	**14BME20**	**CSM 77**	**DSM 14617**	**C2a**	**HKUOPL8**	**ATCC 15305**
class Ib ribonucleoside-diphosphate reductase assembly flavoprotein NrdI	BEK99_RS09435	SCA_RS01925	SE1039_RS02820	SEQMU2_RS07610	BK815_RS06675	A6V26_RS02800	AA913_RS08130	SXYL_RS10370	BE24_RS01365	SSP_RS09960
EamA/RhaT family transporter	BEK99_RS09440	SCA_RS01920	SE1039_RS02815	SEQMU2_RS07605	BK815_RS06680	A6V26_RS02805	AA913_RS08125	SXYL_RS10375	BE24_RS01360	SSP_RS09965
NADPH-dependent 7-cyano-7-deazaguanine reductase QueF	BEK99_RS09445	SCA_RS01915	SE1039_RS02810	SEQMU2_RS07600	BK815_RS06685	A6V26_RS02810	AA913_RS08120	SXYL_RS10380	BE24_RS01355	SSP_RS09970
MFS transporter	BEK99_RS09450	SCA_RS01910	SE1039_RS02805	SEQMU2_RS07595	BK815_RS06690	A6V26_RS02815	AA913_RS08115	SXYL_RS10385	BE24_RS01350	SSP_RS09975
peptide MFS transporter	BEK99_RS09455	SCA_RS01905	SE1039_RS02800	SEQMU2_RS07590	BK815_RS06695	A6V26_RS02820	AA913_RS08110	SXYL_RS10390	BE24_RS01345	SSP_RS09980
pyridoxal phosphate-dependent aminotransferase	BEK99_RS09465	SCA_RS01895	SE1039_RS02785	SEQMU2_RS07575	BK815_RS06710	A6V26_RS02835	AA913_RS08095	SXYL_RS10405	BE24_RS01330	SSP_RS09995
glycine/betaine ABC transporter permease	BEK99_RS09470	SCA_RS01890	SE1039_RS02780	SEQMU2_RS07570	BK815_RS06715	A6V26_RS02840	AA913_RS08090	SXYL_RS10410	BE24_RS01325	SSP_RS10000
glycine/betaine ABC transporter ATP-binding protein	BEK99_RS09475	SCA_RS01885	SE1039_RS02775	SEQMU2_RS07565	BK815_RS06720	A6V26_RS02845	AA913_RS08085	SXYL_RS10415	BE24_RS01320	SSP_RS10005
DNA helicase RecQ	BEK99_RS09480	SCA_RS01880	SE1039_RS02770	SEQMU2_RS07560	BK815_RS06725	A6V26_RS02850	AA913_RS08080	SXYL_RS10420	BE24_RS01315	SSP_RS10010
ABC transporter ATP-binding protein	BEK99_RS09485	SCA_RS01875	SE1039_RS02765	SEQMU2_RS07555	BK815_RS06730	A6V26_RS02855	AA913_RS08075	SXYL_RS10425	BE24_RS01310	SSP_RS10015
glycerol phosphate lipoteichoic acid synthase	BEK99_RS09490	SCA_RS01870	SE1039_RS02755	SEQMU2_RS07545	BK815_RS06740	A6V26_RS02865	AA913_RS08065	SXYL_RS10435	BE24_RS01300	SSP_RS10025
allophanate hydrolase	BEK99_RS09495	SCA_RS01865	SE1039_RS02750	SEQMU2_RS07540	BK815_RS06745	A6V26_RS02870	AA913_RS08060	SXYL_RS10440	BE24_RS01295	SSP_RS10030
allophanate hydrolase	BEK99_RS09500	SCA_RS01860	SE1039_RS02745	SEQMU2_RS07535	BK815_RS06750	A6V26_RS02875	AA913_RS08055	SXYL_RS10445	BE24_RS01290	SSP_RS10035
aminodeoxychorismate lyase	BEK99_RS09505	SCA_RS01855	SE1039_RS02735	SEQMU2_RS07525	BK815_RS06760	A6V26_RS02885	AA913_RS08045	SXYL_RS10450	BE24_RS01285	SSP_RS10040
aminodeoxychorismate synthase component I	BEK99_RS09510	SCA_RS01850	SE1039_RS02730	SEQMU2_RS07520	BK815_RS06765	A6V26_RS02890	AA913_RS08040	SXYL_RS10455	BE24_RS01280	SSP_RS10045
type 1 glutamine amidotransferase	BEK99_RS09515	SCA_RS01845	SE1039_RS02725	SEQMU2_RS07515	BK815_RS06770	A6V26_RS02895	AA913_RS08035	SXYL_RS10460	BE24_RS01275	SSP_RS10050
7-cyano-7-deazaguanine synthase QueC	BEK99_RS09520	SCA_RS01840	SE1039_RS02720	SEQMU2_RS07510	BK815_RS06775	A6V26_RS02900	AA913_RS08030	SXYL_RS10465	BE24_RS01270	SSP_RS10055
6-carboxytetrahydropterin synthase QueD	BEK99_RS09525	SCA_RS01835	SE1039_RS02715	SEQMU2_RS07505	BK815_RS06780	A6V26_RS02905	AA913_RS08025	SXYL_RS10470	BE24_RS01265	SSP_RS10060
7-carboxy-7-deazaguanine synthase QueE	BEK99_RS09530	SCA_RS01830	SE1039_RS02710	SEQMU2_RS07500	BK815_RS06785	A6V26_RS02910	AA913_RS08020	SXYL_RS10475	BE24_RS01260	SSP_RS10065
hypothetical protein	BEK99_RS09535	SCA_RS01825	SE1039_RS02705	SEQMU2_RS07495	BK815_RS06790	A6V26_RS02915	AA913_RS08015	SXYL_RS10480	BE24_RS01255	SSP_RS10070
GlsB/YeaQ/YmgE family stress response membrane protein	BEK99_RS09540	SCA_RS01820	SE1039_RS03995	SEQMU2_RS07490	BK815_RS06795	A6V26_RS02920	AA913_RS08010	SXYL_RS10485	BE24_RS01250	SSP_RS10075
aldo/keto reductase	BEK99_RS09545	SCA_RS01815	SE1039_RS02690	SEQMU2_RS07480	BK815_RS06805	A6V26_RS02930	AA913_RS08000	SXYL_RS10495	BE24_RS01240	SSP_RS10085
HlyC/CorC family transporter	BEK99_RS09575	SCA_RS01785	SE1039_RS02685	SEQMU2_RS07475	BK815_RS06810	A6V26_RS02935	AA913_RS07995	SXYL_RS10500	BE24_RS01235	SSP_RS10090
PTS fructose transporter subunit IIC	BEK99_RS09580	SCA_RS01780	SE1039_RS02675	SEQMU2_RS07465	BK815_RS06820	A6V26_RS02945	AA913_RS07985	SXYL_RS10510	BE24_RS01225	SSP_RS10100
1-phosphofructokinase	BEK99_RS09585	SCA_RS01775	SE1039_RS02670	SEQMU2_RS07460	BK815_RS06825	A6V26_RS02950	AA913_RS07980	SXYL_RS10515	BE24_RS01220	SSP_RS10105
DeoR/GlpR transcriptional regulator	BEK99_RS09590	SCA_RS01770	SE1039_RS02665	SEQMU2_RS07455	BK815_RS06830	A6V26_RS02955	AA913_RS07975	SXYL_RS10520	BE24_RS01215	SSP_RS10110
Cys-tRNA(Pro) deacylase	BEK99_RS09595	SCA_RS01765	SE1039_RS02660	SEQMU2_RS07450	BK815_RS06835	A6V26_RS02960	AA913_RS07970	SXYL_RS10525	BE24_RS01210	SSP_RS10115
lactate dehydrogenase	BEK99_RS09605	SCA_RS01755	SE1039_RS11655	SEQMU2_RS03510	BK815_RS10890	A6V26_RS08475	AA913_RS04255	SXYL_RS01350	BE24_RS10405	SSP_RS01215
methionine ABC transporter substrate-binding protein	BEK99_RS09610	SCA_RS01750	SE1039_RS11660	SEQMU2_RS03515	BK815_RS10885	A6V26_RS08480	AA913_RS04260	SXYL_RS01345	BE24_RS10410	SSP_RS01210
multidrug efflux MFS transporter NorA	BEK99_RS09615	SCA_RS01745	SE1039_RS02645	SEQMU2_RS07435	BK815_RS06850	A6V26_RS02975	AA913_RS07955	SXYL_RS10540	BE24_RS01195	SSP_RS10135
hypothetical protein	BEK99_RS09620	SCA_RS01740	SE1039_RS02635	SEQMU2_RS07425	BK815_RS06860	A6V26_RS02985	AA913_RS07945	SXYL_RS10550	BE24_RS01185	SSP_RS10145

Product	*S. carnosus*		*S. equorum*		*S. succinus*			*S. xylosus*		*S. saprophyticus*
	**JCM 6069**	**TM300**	**KS1039**	**Mu2**	**14BME20**	**CSM 77**	**DSM 14617**	**C2a**	**HKUOPL8**	**ATCC 15305**
malate dehydrogenase	BEK99_RS09635	SCA_RS01725	SE1039_RS02630	SEQMU2_RS07420	BK815_RS06870	A6V26_RS02995	AA913_RS07940	SXYL_RS10555	BE24_RS01175	SSP_RS10150
DedA family protein	BEK99_RS09640	SCA_RS01720	SE1039_RS02625	SEQMU2_RS07415	BK815_RS06875	A6V26_RS03000	AA913_RS07935	SXYL_RS10560	BE24_RS01170	SSP_RS10155
hypothetical protein	BEK99_RS09645	SCA_RS01715	SE1039_RS02620	SEQMU2_RS07410	BK815_RS06880	A6V26_RS03005	AA913_RS07930	SXYL_RS10565	BE24_RS01165	SSP_RS10160
oxidoreductase	BEK99_RS09650	SCA_RS01710	SE1039_RS02615	SEQMU2_RS07405	BK815_RS06885	A6V26_RS03010	AA913_RS07925	SXYL_RS10570	BE24_RS01160	SSP_RS10165
GTP-binding protein	BEK99_RS09655	SCA_RS01705	SE1039_RS02610	SEQMU2_RS07400	BK815_RS06890	A6V26_RS03015	AA913_RS07920	SXYL_RS10575	BE24_RS01155	SSP_RS10170
MarR family transcriptional regulator	BEK99_RS09660	SCA_RS01700	SE1039_RS02605	SEQMU2_RS07395	BK815_RS06895	A6V26_RS03020	AA913_RS07915	SXYL_RS10580	BE24_RS01150	SSP_RS10175
thiol reductant ABC exporter subunit CydC	BEK99_RS09665	SCA_RS01695	SE1039_RS02600	SEQMU2_RS07390	BK815_RS06900	A6V26_RS03025	AA913_RS07910	SXYL_RS10585	BE24_RS01145	SSP_RS10180
cysteine ABC transporter ATP-binding protein	BEK99_RS09670	SCA_RS01690	SE1039_RS02595	SEQMU2_RS07385	BK815_RS06905	A6V26_RS03030	AA913_RS07905	SXYL_RS10590	BE24_RS01140	SSP_RS10185
undecaprenyl-diphosphatase	BEK99_RS09675	SCA_RS01685	SE1039_RS02590	SEQMU2_RS07380	BK815_RS06910	A6V26_RS03035	AA913_RS07900	SXYL_RS10595	BE24_RS01135	SSP_RS10190
DUF188 domain-containing protein	BEK99_RS09685	SCA_RS01675	SE1039_RS02580	SEQMU2_RS07370	BK815_RS06920	A6V26_RS03045	AA913_RS07890	SXYL_RS10605	BE24_RS01125	SSP_RS10200
Rossman fold protein TIGR00730 family	BEK99_RS09690	SCA_RS01670	SE1039_RS02575	SEQMU2_RS07365	BK815_RS06925	A6V26_RS03050	AA913_RS07885	SXYL_RS10610	BE24_RS01120	SSP_RS10205
N-acetyltransferase	BEK99_RS09695	SCA_RS01665	SE1039_RS02565	SEQMU2_RS07350	BK815_RS06940	A6V26_RS03065	AA913_RS07870	SXYL_RS10625	BE24_RS01105	SSP_RS10220
hypothetical protein	BEK99_RS09700	SCA_RS01660	SE1039_RS02560	SEQMU2_RS07345	BK815_RS06945	A6V26_RS03070	AA913_RS07865	SXYL_RS10630	BE24_RS01100	SSP_RS10225
hypothetical protein	BEK99_RS09710	SCA_RS01650	SE1039_RS02545	SEQMU2_RS07330	BK815_RS06960	A6V26_RS03085	AA913_RS07850	SXYL_RS10645	BE24_RS01085	SSP_RS10240
membrane protein	BEK99_RS09715	SCA_RS01645	SE1039_RS02535	SEQMU2_RS07320	BK815_RS06970	A6V26_RS03095	AA913_RS07840	SXYL_RS10655	BE24_RS01075	SSP_RS10250
MFS transporter	BEK99_RS09720	SCA_RS01640	SE1039_RS02530	SEQMU2_RS07315	BK815_RS06975	A6V26_RS03100	AA913_RS07835	SXYL_RS10660	BE24_RS01070	SSP_RS10255
LysR family transcriptional regulator	BEK99_RS09725	SCA_RS01635	SE1039_RS02525	SEQMU2_RS07310	BK815_RS06980	A6V26_RS03105	AA913_RS07830	SXYL_RS10665	BE24_RS01065	SSP_RS10260
hypothetical protein	BEK99_RS09730	SCA_RS01630	SE1039_RS02520	SEQMU2_RS07305	BK815_RS06985	A6V26_RS03110	AA913_RS07825	SXYL_RS10670	BE24_RS01060	SSP_RS10265
cupin	BEK99_RS09735	SCA_RS01625	SE1039_RS02510	SEQMU2_RS07295	BK815_RS06995	A6V26_RS03120	AA913_RS07815	SXYL_RS10680	BE24_RS01050	SSP_RS10275
transcriptional regulator	BEK99_RS09740	SCA_RS01620	SE1039_RS02505	SEQMU2_RS07290	BK815_RS07000	A6V26_RS03125	AA913_RS07810	SXYL_RS10685	BE24_RS01045	SSP_RS10280
MarR family transcriptional regulator	BEK99_RS09745	SCA_RS01615	SE1039_RS02500	SEQMU2_RS07285	BK815_RS07005	A6V26_RS03130	AA913_RS07805	SXYL_RS10690	BE24_RS01040	SSP_RS10285
membrane protein	BEK99_RS09760	SCA_RS01590	SE1039_RS02495	SEQMU2_RS07280	BK815_RS07010	A6V26_RS03135	AA913_RS07800	SXYL_RS10700	BE24_RS01030	SSP_RS10295
peptidase M23	BEK99_RS09765	SCA_RS01585	SE1039_RS02490	SEQMU2_RS07275	BK815_RS07015	A6V26_RS03140	AA913_RS07795	SXYL_RS10705	BE24_RS01025	SSP_RS10300
aldo/keto reductase	BEK99_RS09780	SCA_RS01570	SE1039_RS00075	SEQMU2_RS05670	BK815_RS08895	A6V26_RS04730	AA913_RS07150	SXYL_RS13110	BE24_RS12135	SSP_RS12210
anion permease	BEK99_RS09785	SCA_RS01565	SE1039_RS02485	SEQMU2_RS07270	BK815_RS07020	A6V26_RS03145	AA913_RS07790	SXYL_RS10710	BE24_RS01020	SSP_RS10305
hypothetical protein	BEK99_RS09790	SCA_RS01560	SE1039_RS02480	SEQMU2_RS07265	BK815_RS07025	A6V26_RS03150	AA913_RS07785	SXYL_RS10715	BE24_RS01015	SSP_RS10310
sensor histidine kinase	BEK99_RS09805	SCA_RS01545	SE1039_RS02475	SEQMU2_RS07260	BK815_RS07030	A6V26_RS03155	AA913_RS07780	SXYL_RS10720	BE24_RS01010	SSP_RS10315
DNA-binding response regulator	BEK99_RS09810	SCA_RS01540	SE1039_RS02470	SEQMU2_RS07255	BK815_RS07035	A6V26_RS03160	AA913_RS07775	SXYL_RS10725	BE24_RS01005	SSP_RS10320
N-acetyltransferase	BEK99_RS09815	SCA_RS01535	SE1039_RS02460	SEQMU2_RS07245	BK815_RS07045	A6V26_RS03170	AA913_RS07765	SXYL_RS10735	BE24_RS00995	SSP_RS10330
hypothetical protein	BEK99_RS09825	SCA_RS01525	SE1039_RS02450	SEQMU2_RS07235	BK815_RS07055	A6V26_RS03180	AA913_RS07755	SXYL_RS10745	BE24_RS00985	SSP_RS10340
hypothetical protein	BEK99_RS09830	SCA_RS01520	SE1039_RS02445	SEQMU2_RS07230	BK815_RS07060	A6V26_RS03185	AA913_RS07750	SXYL_RS10750	BE24_RS00980	SSP_RS10345

Product	*S. carnosus*		*S. equorum*		*S. succinus*			*S. xylosus*		*S. saprophyticus*
	**JCM 6069**	**TM300**	**KS1039**	**Mu2**	**14BME20**	**CSM 77**	**DSM 14617**	**C2a**	**HKUOPL8**	**ATCC 15305**
iron ABC transporter permease	BEK99_RS09835	SCA_RS01515	SE1039_RS02425	SEQMU2_RS07210	BK815_RS07080	A6V26_RS03205	AA913_RS07730	SXYL_RS10770	BE24_RS00960	SSP_RS10365
iron ABC transporter permease	BEK99_RS09840	SCA_RS01510	SE1039_RS02420	SEQMU2_RS07205	BK815_RS07085	A6V26_RS03210	AA913_RS07725	SXYL_RS10775	BE24_RS00955	SSP_RS10370
ABC transporter ATP-binding protein	BEK99_RS09845	SCA_RS01505	SE1039_RS02415	SEQMU2_RS07200	BK815_RS07090	A6V26_RS03215	AA913_RS07720	SXYL_RS10780	BE24_RS00950	SSP_RS10375
YitT family protein	BEK99_RS09850	SCA_RS01500	SE1039_RS02410	SEQMU2_RS07195	BK815_RS07095	A6V26_RS03220	AA913_RS07715	SXYL_RS10785	BE24_RS00945	SSP_RS10380
ABC transporter ATP-binding protein	BEK99_RS09860	SCA_RS01490	SE1039_RS02400	SEQMU2_RS07185	BK815_RS07105	A6V26_RS03230	AA913_RS07705	SXYL_RS10795	BE24_RS00935	SSP_RS10390
D-alanyl-D-alanine carboxypeptidase	BEK99_RS09865	SCA_RS01485	SE1039_RS02395	SEQMU2_RS07180	BK815_RS07110	A6V26_RS03235	AA913_RS07700	SXYL_RS10800	BE24_RS00930	SSP_RS10395
CDP-glycerol glycerophosphotransferase family protein	BEK99_RS09880	SCA_RS01470	SE1039_RS02380	SEQMU2_RS07165	BK815_RS07125	A6V26_RS03250	AA913_RS07685	SXYL_RS10815	BE24_RS00915	SSP_RS10410
teichoic acid ABC transporter permease	BEK99_RS09885	SCA_RS01465	SE1039_RS02375	SEQMU2_RS07160	BK815_RS07130	A6V26_RS03255	AA913_RS07680	SXYL_RS10820	BE24_RS00910	SSP_RS10415
teichoic acid ABC transporter ATP-binding protein	BEK99_RS09890	SCA_RS01460	SE1039_RS02370	SEQMU2_RS07155	BK815_RS07135	A6V26_RS03260	AA913_RS07675	SXYL_RS10825	BE24_RS00905	SSP_RS10420
glycosyltransferase	BEK99_RS09915	SCA_RS01435	SE1039_RS02365	SEQMU2_RS07150	BK815_RS07140	A6V26_RS03265	AA913_RS07670	SXYL_RS10830	BE24_RS00900	SSP_RS10425
maltose acetyltransferase	BEK99_RS09920	SCA_RS01430	SE1039_RS11360	SEQMU2_RS03235	BK815_RS11190	A6V26_RS08175	AA913_RS03955	SXYL_RS01675	BE24_RS10100	SSP_RS01490
membrane protein	BEK99_RS09925	SCA_RS01425	SE1039_RS02360	SEQMU2_RS07145	BK815_RS07145	A6V26_RS03270	AA913_RS07665	SXYL_RS10835	BE24_RS00895	SSP_RS10430
DtxR family transcriptional regulator	BEK99_RS09940	SCA_RS01410	SE1039_RS02355	SEQMU2_RS07140	BK815_RS07150	A6V26_RS03275	AA913_RS07660	SXYL_RS10840	BE24_RS00890	SSP_RS10435
metal ABC transporter permease	BEK99_RS09945	SCA_RS01400	SE1039_RS02345	SEQMU2_RS07130	BK815_RS07160	A6V26_RS03285	AA913_RS07650	SXYL_RS10850	BE24_RS00880	SSP_RS10445
metal ABC transporter substrate-binding protein	BEK99_RS09950	SCA_RS01395	SE1039_RS02340	SEQMU2_RS07125	BK815_RS07165	A6V26_RS03290	AA913_RS07645	SXYL_RS10855	BE24_RS00875	SSP_RS10450
sodium:proton antiporter	BEK99_RS09955	SCA_RS01390	SE1039_RS02335	SEQMU2_RS07120	BK815_RS07170	A6V26_RS03295	AA913_RS07640	SXYL_RS10860	BE24_RS00870	SSP_RS10455
hypothetical protein	BEK99_RS09960	SCA_RS01385	SE1039_RS02290	SEQMU2_RS07075	BK815_RS07215	A6V26_RS03340	AA913_RS07595	SXYL_RS10905	BE24_RS00825	SSP_RS10500
DUF2922 domain-containing protein	BEK99_RS09965	SCA_RS01380	SE1039_RS02285	SEQMU2_RS07070	BK815_RS07220	A6V26_RS03345	AA913_RS07590	SXYL_RS10910	BE24_RS00820	SSP_RS10505
hypothetical protein	BEK99_RS09970	SCA_RS01375	SE1039_RS02280	SEQMU2_RS07065	BK815_RS07225	A6V26_RS03350	AA913_RS07585	SXYL_RS10915	BE24_RS00815	SSP_RS10510
membrane protein	BEK99_RS09975	SCA_RS01370	SE1039_RS02275	SEQMU2_RS07060	BK815_RS07230	A6V26_RS03355	AA913_RS07580	SXYL_RS10920	BE24_RS00810	SSP_RS10515
transcriptional regulator	BEK99_RS09985	SCA_RS01360	SE1039_RS02265	SEQMU2_RS07050	BK815_RS07240	A6V26_RS03370	AA913_RS07565	SXYL_RS10930	BE24_RS00800	SSP_RS10525
hypothetical protein	BEK99_RS09990	SCA_RS01355	SE1039_RS02255	SEQMU2_RS07040	BK815_RS07250	A6V26_RS03380	AA913_RS07555	SXYL_RS10940	BE24_RS00790	SSP_RS10535
hypothetical protein	BEK99_RS09995	SCA_RS01350	SE1039_RS02250	SEQMU2_RS07035	BK815_RS07255	A6V26_RS03385	AA913_RS07550	SXYL_RS10945	BE24_RS00785	SSP_RS10540
alpha/beta hydrolase	BEK99_RS10000	SCA_RS01345	SE1039_RS02245	SEQMU2_RS07030	BK815_RS07260	A6V26_RS03390	AA913_RS07545	SXYL_RS10950	BE24_RS00780	SSP_RS10545
HAD family hydrolase	BEK99_RS10005	SCA_RS01340	SE1039_RS02240	SEQMU2_RS07025	BK815_RS07265	A6V26_RS03395	AA913_RS07540	SXYL_RS10955	BE24_RS00775	SSP_RS10550
iron ABC transporter permease	BEK99_RS10010	SCA_RS01335	SE1039_RS02235	SEQMU2_RS07020	BK815_RS07270	A6V26_RS03400	AA913_RS07535	SXYL_RS10960	BE24_RS00770	SSP_RS10555
ABC transporter substrate-binding protein	BEK99_RS10015	SCA_RS01330	SE1039_RS02230	SEQMU2_RS07015	BK815_RS07285	A6V26_RS03410	AA913_RS07525	SXYL_RS10965	BE24_RS00765	SSP_RS10560
arginine--tRNA ligase	BEK99_RS10020	SCA_RS01325	SE1039_RS02215	SEQMU2_RS07000	BK815_RS07295	A6V26_RS03420	AA913_RS07515	SXYL_RS10975	BE24_RS00755	SSP_RS10570
hypothetical protein	BEK99_RS10025	SCA_RS01320	SE1039_RS02210	SEQMU2_RS06995	BK815_RS07300	A6V26_RS03425	AA913_RS07510	SXYL_RS10980	BE24_RS00750	SSP_RS10575
amidohydrolase	BEK99_RS10030	SCA_RS01315	SE1039_RS11305	SEQMU2_RS03180	BK815_RS11240	A6V26_RS08125	AA913_RS01830	SXYL_RS01725	BE24_RS10050	SSP_RS01540
hypothetical protein	BEK99_RS10060	SCA_RS01285	SE1039_RS02205	SEQMU2_RS06990	BK815_RS07305	A6V26_RS03430	AA913_RS07505	SXYL_RS10985	BE24_RS00745	SSP_RS10580

Product	*S. carnosus*		*S. equorum*		*S. succinus*			*S. xylosus*		*S. saprophyticus*
	**JCM 6069**	**TM300**	**KS1039**	**Mu2**	**14BME20**	**CSM 77**	**DSM 14617**	**C2a**	**HKUOPL8**	**ATCC 15305**
HD domain-containing protein	BEK99_RS10065	SCA_RS01280	SE1039_RS02200	SEQMU2_RS06985	BK815_RS07310	A6V26_RS03435	AA913_RS07500	SXYL_RS10990	BE24_RS00740	SSP_RS10585
acetyltransferase	BEK99_RS10070	SCA_RS01275	SE1039_RS02195	SEQMU2_RS06980	BK815_RS07315	A6V26_RS03440	AA913_RS07495	SXYL_RS10995	BE24_RS00735	SSP_RS10590
flavodoxin family protein	BEK99_RS10075	SCA_RS01270	SE1039_RS02190	SEQMU2_RS06975	BK815_RS07320	A6V26_RS03445	AA913_RS07490	SXYL_RS11000	BE24_RS00730	SSP_RS10595
hypothetical protein	BEK99_RS10085	SCA_RS01260	SE1039_RS02180	SEQMU2_RS06965	BK815_RS07330	A6V26_RS03455	AA913_RS07480	SXYL_RS11010	BE24_RS00720	SSP_RS10605
phosphomevalonate kinase	BEK99_RS10090	SCA_RS01255	SE1039_RS02145	SEQMU2_RS06930	BK815_RS07365	A6V26_RS03490	AA913_RS07445	SXYL_RS11045	BE24_RS00715	SSP_RS10610
diphosphomevalonate decarboxylase	BEK99_RS10095	SCA_RS01250	SE1039_RS02140	SEQMU2_RS06925	BK815_RS07370	A6V26_RS03495	AA913_RS07440	SXYL_RS11050	BE24_RS00710	SSP_RS10615
mevalonate kinase	BEK99_RS10100	SCA_RS01245	SE1039_RS02135	SEQMU2_RS06920	BK815_RS07375	A6V26_RS03500	AA913_RS07435	SXYL_RS11055	BE24_RS00705	SSP_RS10620
biotin/lipoate A/B protein ligase family protein	BEK99_RS10105	SCA_RS01240	SE1039_RS02130	SEQMU2_RS06915	BK815_RS07380	A6V26_RS03505	AA913_RS07430	SXYL_RS11060	BE24_RS00700	SSP_RS10625
phosphate acetyltransferase	BEK99_RS10110	SCA_RS01235	SE1039_RS02125	SEQMU2_RS06910	BK815_RS07385	A6V26_RS03510	AA913_RS07425	SXYL_RS11065	BE24_RS00695	SSP_RS10630
heme-binding protein	BEK99_RS10115	SCA_RS01230	SE1039_RS02120	SEQMU2_RS06905	BK815_RS07390	A6V26_RS03515	AA913_RS07420	SXYL_RS11070	BE24_RS00690	SSP_RS10635
threonine/serine exporter	BEK99_RS10120	SCA_RS01225	SE1039_RS02115	SEQMU2_RS06900	BK815_RS07395	A6V26_RS03520	AA913_RS07415	SXYL_RS11075	BE24_RS00685	SSP_RS10640
hypothetical protein	BEK99_RS10125	SCA_RS01220	SE1039_RS02110	SEQMU2_RS06895	BK815_RS07400	A6V26_RS03525	AA913_RS07410	SXYL_RS11080	BE24_RS00680	SSP_RS10645
amino acid permease	BEK99_RS10140	SCA_RS01205	SE1039_RS02105	SEQMU2_RS06890	BK815_RS07405	A6V26_RS03530	AA913_RS07405	SXYL_RS11085	BE24_RS00675	SSP_RS10660
DUF423 domain-containing protein	BEK99_RS10145	SCA_RS01200	SE1039_RS02100	SEQMU2_RS06885	BK815_RS07410	A6V26_RS03535	AA913_RS07400	SXYL_RS11090	BE24_RS00670	SSP_RS10665
uracil-DNA glycosylase	BEK99_RS10155	SCA_RS01190	SE1039_RS02090	SEQMU2_RS06875	BK815_RS07420	A6V26_RS03545	AA913_RS07390	SXYL_RS11100	BE24_RS00660	SSP_RS10675
hydroxymethylpyrimidine/phosphomethylpyrimidine kinase	BEK99_RS10160	SCA_RS01185	SE1039_RS02085	SEQMU2_RS06870	BK815_RS07425	A6V26_RS03550	AA913_RS11225	SXYL_RS11105	BE24_RS00655	SSP_RS10680
MFS transporter	BEK99_RS10165	SCA_RS01180	SE1039_RS11885	SEQMU2_RS03730	BK815_RS07435	A6V26_RS03560	AA913_RS11210	SXYL_RS11120	BE24_RS00640	SSP_RS10710
GTP cyclohydrolase I FolE2	BEK99_RS10170	SCA_RS01175	SE1039_RS02020	SEQMU2_RS06805	BK815_RS07485	A6V26_RS03610	AA913_RS11160	SXYL_RS11225	BE24_RS00545	SSP_RS10760
bacillithiol biosynthesis deacetylase BshB2	BEK99_RS10175	SCA_RS01170	SE1039_RS02025	SEQMU2_RS06810	BK815_RS07480	A6V26_RS03605	AA913_RS11165	SXYL_RS11220	BE24_RS00550	SSP_RS10755
hypothetical protein	BEK99_RS10180	SCA_RS01165	SE1039_RS02030	SEQMU2_RS06815	BK815_RS07475	A6V26_RS03600	AA913_RS11170	SXYL_RS11215	BE24_RS00555	SSP_RS10750
6-phospho-3-hexuloisomerase	BEK99_RS10195	SCA_RS01150	SE1039_RS02040	SEQMU2_RS14200	BK815_RS07460	A6V26_RS13350	AA913_RS12915	SXYL_RS11200	BE24_RS00570	SSP_RS08160
NAD(P)H-dependent oxidoreductase	BEK99_RS10200	SCA_RS01145	SE1039_RS02005	SEQMU2_RS06790	BK815_RS07500	A6V26_RS03625	AA913_RS11145	SXYL_RS11240	BE24_RS00530	SSP_RS10770
HAD family phosphatase	BEK99_RS10205	SCA_RS01140	SE1039_RS02000	SEQMU2_RS06785	BK815_RS07505	A6V26_RS03630	AA913_RS11140	SXYL_RS11245	BE24_RS00525	SSP_RS10775
tRNA-specific adenosine deaminase	BEK99_RS10210	SCA_RS01135	SE1039_RS01995	SEQMU2_RS06780	BK815_RS07510	A6V26_RS03635	AA913_RS11135	SXYL_RS11250	BE24_RS00520	SSP_RS10780
deoxyguanosine kinase	BEK99_RS10215	SCA_RS01130	SE1039_RS01990	SEQMU2_RS06775	BK815_RS07515	A6V26_RS03640	AA913_RS11130	SXYL_RS11255	BE24_RS00515	SSP_RS10785
deoxynucleoside kinase	BEK99_RS10220	SCA_RS01125	SE1039_RS01985	SEQMU2_RS06770	BK815_RS07520	A6V26_RS03645	AA913_RS11125	SXYL_RS11260	BE24_RS00510	SSP_RS10790
hypothetical protein	BEK99_RS10230	SCA_RS01115	SE1039_RS01980	SEQMU2_RS06765	BK815_RS07525	A6V26_RS03650	AA913_RS11120	SXYL_RS11265	BE24_RS00505	SSP_RS10795
HAD family hydrolase	BEK99_RS10235	SCA_RS01110	SE1039_RS01975	SEQMU2_RS06760	BK815_RS07530	A6V26_RS03655	AA913_RS11115	SXYL_RS11270	BE24_RS00500	SSP_RS10800
branched-chain amino acid aminotransferase	BEK99_RS10245	SCA_RS01100	SE1039_RS01825	SEQMU2_RS05145	BK815_RS07535	A6V26_RS03660	AA913_RS11110	SXYL_RS11285	BE24_RS00485	SSP_RS10815
L-threonine 3-dehydrogenase	BEK99_RS10255	SCA_RS01090	SE1039_RS01820	SEQMU2_RS05140	BK815_RS07540	A6V26_RS03665	AA913_RS11105	SXYL_RS11290	BE24_RS00480	SSP_RS10820
GNAT family acetyltransferase	BEK99_RS10265	SCA_RS01080	SE1039_RS01640	SEQMU2_RS00185	BK815_RS07670	A6V26_RS03790	AA913_RS10980	SXYL_RS11550	BE24_RS00185	SSP_RS11000

Product	*S. carnosus*		*S. equorum*		*S. succinus*			*S. xylosus*		*S. saprophyticus*
	**JCM 6069**	**TM300**	**KS1039**	**Mu2**	**14BME20**	**CSM 77**	**DSM 14617**	**C2a**	**HKUOPL8**	**ATCC 15305**
2-amino-3-ketobutyrate CoA ligase	BEK99_RS10275	SCA_RS01070	SE1039_RS01620	SEQMU2_RS00165	BK815_RS07690	A6V26_RS03810	AA913_RS10960	SXYL_RS11570	BE24_RS00165	SSP_RS11020
elongation factor Tu	BEK99_RS10285	SCA_RS01060	SE1039_RS01595	SEQMU2_RS00155	BK815_RS07700	A6V26_RS03820	AA913_RS10950	SXYL_RS11580	BE24_RS00155	SSP_RS11030
elongation factor G	BEK99_RS10290	SCA_RS01055	SE1039_RS01590	SEQMU2_RS00150	BK815_RS07705	A6V26_RS03825	AA913_RS10945	SXYL_RS11585	BE24_RS00150	SSP_RS11035
30S ribosomal protein S7	BEK99_RS10295	SCA_RS01050	SE1039_RS01585	SEQMU2_RS00145	BK815_RS07710	A6V26_RS03830	AA913_RS10940	SXYL_RS11590	BE24_RS00145	SSP_RS11040
30S ribosomal protein S12	BEK99_RS10300	SCA_RS01045	SE1039_RS01580	SEQMU2_RS00140	BK815_RS07715	A6V26_RS03835	AA913_RS10935	SXYL_RS11595	BE24_RS00140	SSP_RS11045
50S ribosomal protein L7ae-like protein	BEK99_RS10305	SCA_RS01040	SE1039_RS01575	SEQMU2_RS00135	BK815_RS07720	A6V26_RS03840	AA913_RS10930	SXYL_RS11600	BE24_RS00135	SSP_RS11050
DNA-directed RNA polymerase subunit beta	BEK99_RS10315	SCA_RS01030	SE1039_RS01570	SEQMU2_RS00130	BK815_RS07725	A6V26_RS03845	AA913_RS10925	SXYL_RS11605	BE24_RS00130	SSP_RS11055
DNA-directed RNA polymerase subunit beta	BEK99_RS10320	SCA_RS01025	SE1039_RS01565	SEQMU2_RS00125	BK815_RS07730	A6V26_RS03850	AA913_RS10920	SXYL_RS11610	BE24_RS00125	SSP_RS11060
methyltransferase	BEK99_RS10325	SCA_RS01020	SE1039_RS01560	SEQMU2_RS00120	BK815_RS07735	A6V26_RS03855	AA913_RS10915	SXYL_RS11615	BE24_RS00120	SSP_RS11065
50S ribosomal protein L7/L12	BEK99_RS10330	SCA_RS01015	SE1039_RS01555	SEQMU2_RS00115	BK815_RS07740	A6V26_RS03860	AA913_RS10910	SXYL_RS11620	BE24_RS00115	SSP_RS11070
50S ribosomal protein L10	BEK99_RS10335	SCA_RS01010	SE1039_RS01550	SEQMU2_RS00110	BK815_RS07745	A6V26_RS03865	AA913_RS10905	SXYL_RS11625	BE24_RS00110	SSP_RS11075
50S ribosomal protein L1	BEK99_RS10340	SCA_RS01005	SE1039_RS01545	SEQMU2_RS00105	BK815_RS07750	A6V26_RS03870	AA913_RS10900	SXYL_RS11630	BE24_RS00105	SSP_RS11080
50S ribosomal protein L11	BEK99_RS10345	SCA_RS01000	SE1039_RS01540	SEQMU2_RS00100	BK815_RS07755	A6V26_RS03875	AA913_RS10895	SXYL_RS11635	BE24_RS00100	SSP_RS11085
transcription termination/antitermination protein NusG	BEK99_RS10350	SCA_RS00995	SE1039_RS01535	SEQMU2_RS00095	BK815_RS07760	A6V26_RS03880	AA913_RS10890	SXYL_RS11640	BE24_RS00095	SSP_RS11090
preprotein translocase subunit SecE	BEK99_RS10355	SCA_RS00990	SE1039_RS01530	SEQMU2_RS00090	BK815_RS07765	A6V26_RS03885	AA913_RS10885	SXYL_RS11645	BE24_RS00090	SSP_RS11095
hypothetical protein	BEK99_RS10365	SCA_RS00980	SE1039_RS01520	SEQMU2_RS00080	BK815_RS07780	A6V26_RS03895	AA913_RS10875	SXYL_RS11660	BE24_RS00080	SSP_RS11110
23S rRNA (guanosine(2251)-2-O)-methyltransferase RlmB	BEK99_RS10370	SCA_RS00975	SE1039_RS01515	SEQMU2_RS00075	BK815_RS07785	A6V26_RS03900	AA913_RS10870	SXYL_RS11665	BE24_RS00075	SSP_RS11115
ribonuclease III	BEK99_RS10375	SCA_RS00970	SE1039_RS01510	SEQMU2_RS00070	BK815_RS07790	A6V26_RS03905	AA913_RS10865	SXYL_RS11670	BE24_RS00070	SSP_RS11120
cysteine--tRNA ligase	BEK99_RS10380	SCA_RS00965	SE1039_RS01505	SEQMU2_RS00065	BK815_RS07795	A6V26_RS03910	AA913_RS10860	SXYL_RS11675	BE24_RS00065	SSP_RS11125
serine O-acetyltransferase	BEK99_RS10385	SCA_RS00960	SE1039_RS01500	SEQMU2_RS00060	BK815_RS07800	A6V26_RS03915	AA913_RS10855	SXYL_RS11680	BE24_RS00060	SSP_RS11130
glutamate--tRNA ligase	BEK99_RS10390	SCA_RS00955	SE1039_RS01495	SEQMU2_RS00055	BK815_RS07805	A6V26_RS03920	AA913_RS10850	SXYL_RS11685	BE24_RS00055	SSP_RS11135
PIN/TRAM domain-containing protein	BEK99_RS10395	SCA_RS00950	SE1039_RS01490	SEQMU2_RS00050	BK815_RS07810	A6V26_RS03925	AA913_RS10845	SXYL_RS11690	BE24_RS00050	SSP_RS11140
DNA repair protein RadA	BEK99_RS10400	SCA_RS00945	SE1039_RS01485	SEQMU2_RS00045	BK815_RS07815	A6V26_RS03930	AA913_RS10840	SXYL_RS11695	BE24_RS00045	SSP_RS11145
ATP-dependent Clp protease ATP-binding subunit	BEK99_RS10405	SCA_RS00940	SE1039_RS01480	SEQMU2_RS00040	BK815_RS07820	A6V26_RS03935	AA913_RS10835	SXYL_RS11700	BE24_RS00040	SSP_RS11150
ATP--guanido phosphotransferase	BEK99_RS10410	SCA_RS00935	SE1039_RS01475	SEQMU2_RS00035	BK815_RS07825	A6V26_RS03940	AA913_RS10830	SXYL_RS11705	BE24_RS00035	SSP_RS11155
excinuclease ABC subunit B	BEK99_RS10415	SCA_RS00930	SE1039_RS01470	SEQMU2_RS00030	BK815_RS07830	A6V26_RS03945	AA913_RS10825	SXYL_RS11710	BE24_RS00030	SSP_RS11160
hypothetical protein	BEK99_RS10420	SCA_RS00925	SE1039_RS01465	SEQMU2_RS00025	BK815_RS07835	A6V26_RS03950	AA913_RS10820	SXYL_RS11715	BE24_RS00025	SSP_RS11165
nucleoside permease	BEK99_RS10425	SCA_RS00920	SE1039_RS01460	SEQMU2_RS00020	BK815_RS07840	A6V26_RS03955	AA913_RS10815	SXYL_RS11720	BE24_RS00020	SSP_RS11170
RNA-binding protein	BEK99_RS10430	SCA_RS00915	SE1039_RS02070	SEQMU2_RS06855	BK815_RS09375	A6V26_RS05235	AA913_RS02090	SXYL_RS11115	BE24_RS00645	SSP_RS10695
pyridoxal 5-phosphate synthase glutaminase subunit PdxT	BEK99_RS10440	SCA_RS00905	SE1039_RS01455	SEQMU2_RS00015	BK815_RS07845	A6V26_RS03960	AA913_RS10810	SXYL_RS11725	BE24_RS00015	SSP_RS11175
pyridoxal 5-phosphate synthase lyase subunit PdxS	BEK99_RS10445	SCA_RS00900	SE1039_RS01450	SEQMU2_RS00010	BK815_RS07850	A6V26_RS03965	AA913_RS10805	SXYL_RS11730	BE24_RS00010	SSP_RS11180

Product	*S. carnosus*		*S. equorum*		*S. succinus*			*S. xylosus*		*S. saprophyticus*
	**JCM 6069**	**TM300**	**KS1039**	**Mu2**	**14BME20**	**CSM 77**	**DSM 14617**	**C2a**	**HKUOPL8**	**ATCC 15305**
MFS transporter	BEK99_RS10460	SCA_RS00885	SE1039_RS02540	SEQMU2_RS07325	BK815_RS06965	A6V26_RS03090	AA913_RS07845	SXYL_RS10650	BE24_RS01080	SSP_RS10245
lysine--tRNA ligase	BEK99_RS10545	SCA_RS00820	SE1039_RS01360	SEQMU2_RS13600	BK815_RS07940	A6V26_RS13195	AA913_RS12340	SXYL_RS11805	BE24_RS06730	SSP_RS11270
2-amino-4-hydroxy-6- hydroxymethyldihydropteridine diphosphokinase	BEK99_RS10555	SCA_RS00810	SE1039_RS01355	SEQMU2_RS13605	BK815_RS07950	A6V26_RS13190	AA913_RS12335	SXYL_RS11810	BE24_RS06735	SSP_RS11275
dihydroneopterin aldolase	BEK99_RS10560	SCA_RS00805	SE1039_RS01350	SEQMU2_RS13610	BK815_RS07955	A6V26_RS13185	AA913_RS12330	SXYL_RS11815	BE24_RS06740	SSP_RS11280
cysteine synthase A	BEK99_RS10570	SCA_RS00795	SE1039_RS01340	SEQMU2_RS13620	BK815_RS07965	A6V26_RS13175	AA913_RS12320	SXYL_RS11825	BE24_RS06750	SSP_RS11290
redox-regulated molecular chaperone Hsp33	BEK99_RS10575	SCA_RS00790	SE1039_RS01335	SEQMU2_RS13625	BK815_RS07970	A6V26_RS13170	AA913_RS12315	SXYL_RS11830	BE24_RS06755	SSP_RS11295
zinc metalloprotease	BEK99_RS10580	SCA_RS00785	SE1039_RS01330	SEQMU2_RS13630	BK815_RS07975	A6V26_RS13165	AA913_RS12310	SXYL_RS11835	BE24_RS06760	SSP_RS11300
hypoxanthine phosphoribosyltransferase	BEK99_RS10585	SCA_RS00780	SE1039_RS01325	SEQMU2_RS13635	BK815_RS07980	A6V26_RS13160	AA913_RS12305	SXYL_RS11840	BE24_RS06765	SSP_RS11305
tRNA(Ile)-lysidine synthetase	BEK99_RS10590	SCA_RS00775	SE1039_RS01320	SEQMU2_RS13640	BK815_RS07985	A6V26_RS13155	AA913_RS12300	SXYL_RS11845	BE24_RS06770	SSP_RS11310
RNA-binding protein S1	BEK99_RS10595	SCA_RS00770	SE1039_RS01315	SEQMU2_RS13645	BK815_RS07990	A6V26_RS13150	AA913_RS12295	SXYL_RS11850	BE24_RS06775	SSP_RS11315
cell division protein DIVIC	BEK99_RS10600	SCA_RS00765	SE1039_RS01310	SEQMU2_RS13650	BK815_RS07995	A6V26_RS13145	AA913_RS12290	SXYL_RS11855	BE24_RS06780	SSP_RS11320
hypothetical protein	BEK99_RS10605	SCA_RS00760	SE1039_RS01305	SEQMU2_RS13655	BK815_RS08000	A6V26_RS13140	AA913_RS12285	SXYL_RS11860	BE24_RS06785	SSP_RS11325
nucleotide pyrophosphohydrolase	BEK99_RS10610	SCA_RS00750	SE1039_RS01300	SEQMU2_RS13660	BK815_RS08005	A6V26_RS13135	AA913_RS12280	SXYL_RS11865	BE24_RS06790	SSP_RS11330
stage V sporulation protein B	BEK99_RS10615	SCA_RS00745	SE1039_RS01295	SEQMU2_RS13665	BK815_RS08010	A6V26_RS13130	AA913_RS12275	SXYL_RS11870	BE24_RS06795	SSP_RS11335
transcription-repair coupling factor	BEK99_RS10620	SCA_RS00740	SE1039_RS01290	SEQMU2_RS13670	BK815_RS08015	A6V26_RS13125	AA913_RS12270	SXYL_RS11875	BE24_RS06800	SSP_RS11340
peptidyl-tRNA hydrolase	BEK99_RS10625	SCA_RS00735	SE1039_RS01285	SEQMU2_RS13675	BK815_RS08020	A6V26_RS13120	AA913_RS12265	SXYL_RS11880	BE24_RS06805	SSP_RS11345
50S ribosomal protein L25	BEK99_RS10630	SCA_RS00730	SE1039_RS01280	SEQMU2_RS13680	BK815_RS08025	A6V26_RS13115	AA913_RS12260	SXYL_RS11885	BE24_RS06810	SSP_RS11350
ribose-phosphate pyrophosphokinase	BEK99_RS10635	SCA_RS00725	SE1039_RS01275	SEQMU2_RS13685	BK815_RS08030	A6V26_RS13110	AA913_RS12255	SXYL_RS11890	BE24_RS06815	SSP_RS11355
UDP-N-acetylglucosamine diphosphorylase/glucosamine-1-phosphate N-acetyltransferase	BEK99_RS10640	SCA_RS00720	SE1039_RS01270	SEQMU2_RS13690	BK815_RS08035	A6V26_RS13105	AA913_RS12250	SXYL_RS11895	BE24_RS06820	SSP_RS11360
stage V sporulation protein G	BEK99_RS10645	SCA_RS00715	SE1039_RS01265	SEQMU2_RS13695	BK815_RS08040	A6V26_RS13100	AA913_RS12245	SXYL_RS11900	BE24_RS06825	SSP_RS11365
RidA family protein	BEK99_RS10650	SCA_RS00710	SE1039_RS01260	SEQMU2_RS13700	BK815_RS08045	A6V26_RS13095	AA913_RS12240	SXYL_RS11905	BE24_RS06830	SSP_RS11370
pur operon repressor	BEK99_RS10655	SCA_RS00705	SE1039_RS01255	SEQMU2_RS13705	BK815_RS08050	A6V26_RS13090	AA913_RS12235	SXYL_RS11910	BE24_RS06835	SSP_RS11375
4-(cytidine 5-diphospho)-2-C-methyl-D-erythritol kinase	BEK99_RS10660	SCA_RS00700	SE1039_RS01250	SEQMU2_RS13710	BK815_RS08055	A6V26_RS13085	AA913_RS12230	SXYL_RS11915	BE24_RS06840	SSP_RS11380
hypothetical protein	BEK99_RS10665	SCA_RS00695	SE1039_RS01245	SEQMU2_RS13715	BK815_RS08060	A6V26_RS13080	AA913_RS12225	SXYL_RS11920	BE24_RS06845	SSP_RS11385
ribosomal RNA small subunit methyltransferase A	BEK99_RS10670	SCA_RS00690	SE1039_RS01240	SEQMU2_RS13720	BK815_RS08065	A6V26_RS13075	AA913_RS12220	SXYL_RS11925	BE24_RS06850	SSP_RS11390
ribonuclease M5	BEK99_RS10675	SCA_RS00685	SE1039_RS01235	SEQMU2_RS13725	BK815_RS08070	A6V26_RS13070	AA913_RS12215	SXYL_RS11930	BE24_RS06855	SSP_RS11395
TatD family deoxyribonuclease	BEK99_RS10680	SCA_RS00680	SE1039_RS01230	SEQMU2_RS13730	BK815_RS08075	A6V26_RS13065	AA913_RS12210	SXYL_RS11935	BE24_RS06860	SSP_RS11400
methionine--tRNA ligase	BEK99_RS10685	SCA_RS00675	SE1039_RS01225	SEQMU2_RS13735	BK815_RS08080	A6V26_RS13060	AA913_RS12205	SXYL_RS11940	BE24_RS06865	SSP_RS11405
rRNA (cytidine-2-O-)-methyltransferase	BEK99_RS10730	SCA_RS00635	SE1039_RS01220	SEQMU2_RS13740	BK815_RS08085	A6V26_RS13055	AA913_RS12200	SXYL_RS11945	BE24_RS06870	SSP_RS11410
GIY-YIG nuclease family protein	BEK99_RS10735	SCA_RS00630	SE1039_RS01215	SEQMU2_RS13745	BK815_RS08090	A6V26_RS13050	AA913_RS12195	SXYL_RS11950	BE24_RS06875	SSP_RS11415
DNA replication protein YabA	BEK99_RS10745	SCA_RS00620	SE1039_RS01205	SEQMU2_RS13755	BK815_RS08100	A6V26_RS13040	AA913_RS12185	SXYL_RS11960	BE24_RS06885	SSP_RS11425

Product	*S. carnosus*		*S. equorum*		*S. succinus*			*S. xylosus*		*S. saprophyticus*
	**JCM 6069**	**TM300**	**KS1039**	**Mu2**	**14BME20**	**CSM 77**	**DSM 14617**	**C2a**	**HKUOPL8**	**ATCC 15305**
signal peptidase II	BEK99_RS10750	SCA_RS00615	SE1039_RS01200	SEQMU2_RS13760	BK815_RS08105	A6V26_RS13035	AA913_RS12180	SXYL_RS11965	BE24_RS06890	SSP_RS11430
hypothetical protein	BEK99_RS10760	SCA_RS00605	SE1039_RS01190	SEQMU2_RS13770	BK815_RS08115	A6V26_RS13025	AA913_RS12170	SXYL_RS11975	BE24_RS06900	SSP_RS11440
dTMP kinase	BEK99_RS10765	SCA_RS00600	SE1039_RS01185	SEQMU2_RS13775	BK815_RS08120	A6V26_RS13020	AA913_RS12165	SXYL_RS11980	BE24_RS06905	SSP_RS11445
lysine decarboxylase	BEK99_RS10770	SCA_RS00595	SE1039_RS01180	SEQMU2_RS13780	BK815_RS08125	A6V26_RS13015	AA913_RS12160	SXYL_RS11985	BE24_RS06910	SSP_RS11450
recombination protein RecR	BEK99_RS10790	SCA_RS00575	SE1039_RS01140	SEQMU2_RS06725	BK815_RS08145	A6V26_RS03990	AA913_RS06415	SXYL_RS12020	BE24_RS13255	SSP_RS11470
YbaB/EbfC family nucleoid-associated protein	BEK99_RS10795	SCA_RS00570	SE1039_RS01135	SEQMU2_RS06720	BK815_RS08150	A6V26_RS03995	AA913_RS06420	SXYL_RS12025	BE24_RS13250	SSP_RS11475
DNA polymerase III subunit gamma/tau	BEK99_RS10800	SCA_RS00565	SE1039_RS01130	SEQMU2_RS06715	BK815_RS08155	A6V26_RS04000	AA913_RS06425	SXYL_RS12030	BE24_RS13245	SSP_RS11480
trehalose operon repressor	BEK99_RS10840	SCA_RS00530	SE1039_RS01120	SEQMU2_RS06705	BK815_RS08170	A6V26_RS04015	AA913_RS06435	SXYL_RS12040	BE24_RS13235	SSP_RS11490
glucohydrolase	BEK99_RS10845	SCA_RS00525	SE1039_RS01115	SEQMU2_RS06700	BK815_RS08175	A6V26_RS04020	AA913_RS06440	SXYL_RS12045	BE24_RS13230	SSP_RS11495
glyoxalase/bleomycin resistance/extradiol dioxygenase family protein	BEK99_RS10855	SCA_RS00515	SE1039_RS03245	SEQMU2_RS08455	BK815_RS06275	A6V26_RS02420	AA913_RS08500	SXYL_RS10000	BE24_RS01725	SSP_RS09485
glutamate synthase subunit beta	BEK99_RS10865	SCA_RS00505	SE1039_RS01100	SEQMU2_RS06685	BK815_RS08190	A6V26_RS04035	AA913_RS06455	SXYL_RS12060	BE24_RS13215	SSP_RS11510
glutamate synthase subunit alpha	BEK99_RS10870	SCA_RS00500	SE1039_RS01095	SEQMU2_RS06680	BK815_RS08195	A6V26_RS04040	AA913_RS06460	SXYL_RS12065	BE24_RS13210	SSP_RS11515
LysR family transcriptional regulator	BEK99_RS10875	SCA_RS00495	SE1039_RS01090	SEQMU2_RS06675	BK815_RS08200	A6V26_RS04045	AA913_RS06465	SXYL_RS12070	BE24_RS13205	SSP_RS11520
YibE/F-like protein	BEK99_RS10880	SCA_RS00490	SE1039_RS01085	SEQMU2_RS06670	BK815_RS08205	A6V26_RS04050	AA913_RS06470	SXYL_RS12075	BE24_RS13200	SSP_RS11525
YibE/F-like protein	BEK99_RS10885	SCA_RS00485	SE1039_RS01080	SEQMU2_RS06665	BK815_RS08210	A6V26_RS04055	AA913_RS06475	SXYL_RS12080	BE24_RS13195	SSP_RS11530
hypothetical protein	BEK99_RS10890	SCA_RS00480	SE1039_RS01065	SEQMU2_RS06650	BK815_RS08225	A6V26_RS04070	AA913_RS06490	SXYL_RS12095	BE24_RS13180	SSP_RS11545
N-acetylmuramoyl-L-alanine amidase	BEK99_RS10920	SCA_RS00450	SE1039_RS01060	SEQMU2_RS06645	BK815_RS08230	A6V26_RS04075	AA913_RS06495	SXYL_RS12100	BE24_RS13175	SSP_RS11550
TetR family transcriptional regulator	BEK99_RS10935	SCA_RS00435	SE1039_RS10775	SEQMU2_RS02655	BK815_RS11815	A6V26_RS07525	AA913_RS01230	SXYL_RS02370	BE24_RS09415	SSP_RS02180
3-keto-5-aminohexanoate cleavage protein	BEK99_RS10940	SCA_RS00430	SE1039_RS10770	SEQMU2_RS02650	BK815_RS11820	A6V26_RS07520	AA913_RS01225	SXYL_RS02375	BE24_RS09410	SSP_RS02185
3-hydroxybutyryl-CoA dehydrogenase	BEK99_RS10945	SCA_RS00425	SE1039_RS10765	SEQMU2_RS02645	BK815_RS11825	A6V26_RS07515	AA913_RS01220	SXYL_RS02380	BE24_RS09405	SSP_RS02190
thioesterase	BEK99_RS10950	SCA_RS00420	SE1039_RS10760	SEQMU2_RS02640	BK815_RS11830	A6V26_RS07510	AA913_RS01215	SXYL_RS02385	BE24_RS09400	SSP_RS02195
lysine transporter LysE	BEK99_RS10955	SCA_RS00415	SE1039_RS02570	SEQMU2_RS07360	BK815_RS06930	A6V26_RS03055	AA913_RS07880	SXYL_RS10615	BE24_RS01115	SSP_RS10210
cysteine synthase	BEK99_RS10965	SCA_RS00405	SE1039_RS11615	SEQMU2_RS03470	BK815_RS10920	A6V26_RS08445	AA913_RS04225	SXYL_RS01390	BE24_RS10355	SSP_RS01230
sodium-dependent transporter	BEK99_RS10970	SCA_RS00400	SE1039_RS01045	SEQMU2_RS06630	BK815_RS08245	A6V26_RS04085	AA913_RS06505	SXYL_RS12120	BE24_RS13150	SSP_RS11580
putative esterase/lipase	BEK99_RS10980	SCA_RS00390	SE1039_RS01030	SEQMU2_RS06615	BK815_RS08260	A6V26_RS04100	AA913_RS06520	SXYL_RS12165	BE24_RS13105	SSP_RS11600
carboxylesterase	BEK99_RS11020	SCA_RS00350	SE1039_RS11025	SEQMU2_RS02905	BK815_RS11525	A6V26_RS07815	AA913_RS01520	SXYL_RS02060	BE24_RS09725	SSP_RS01920
ring-cleaving dioxygenase	BEK99_RS11025	SCA_RS00345	SE1039_RS11035	SEQMU2_RS02915	BK815_RS11515	A6V26_RS07825	AA913_RS01530	SXYL_RS02050	BE24_RS09735	SSP_RS01910
5-methyltetrahydropteroyltriglutamate-- homocysteine methyltransferase	BEK99_RS11045	SCA_RS00325	SE1039_RS02515	SEQMU2_RS07300	BK815_RS06990	A6V26_RS03115	AA913_RS07820	SXYL_RS10675	BE24_RS01055	SSP_RS10270
thioesterase	BEK99_RS11060	SCA_RS00315	SE1039_RS11605	SEQMU2_RS03460	BK815_RS10940	A6V26_RS08425	AA913_RS04205	SXYL_RS01410	BE24_RS10335	SSP_RS01250
sulfate adenylyltransferase	BEK99_RS11075	SCA_RS00300	SE1039_RS00435	SEQMU2_RS05940	BK815_RS08750	A6V26_RS04585	AA913_RS07005	SXYL_RS12870	BE24_RS12355	SSP_RS12060
anion permease	BEK99_RS11080	SCA_RS00295	SE1039_RS00430	SEQMU2_RS05935	BK815_RS08755	A6V26_RS04590	AA913_RS07010	SXYL_RS12875	BE24_RS12350	SSP_RS12065

Product	*S. carnosus*		*S. equorum*		*S. succinus*			*S. xylosus*		*S. saprophyticus*
	**JCM 6069**	**TM300**	**KS1039**	**Mu2**	**14BME20**	**CSM 77**	**DSM 14617**	**C2a**	**HKUOPL8**	**ATCC 15305**
uroporphyrinogen-III C-methyltransferase	BEK99_RS11085	SCA_RS00290	SE1039_RS00415	SEQMU2_RS05920	BK815_RS08770	A6V26_RS04605	AA913_RS07025	SXYL_RS12890	BE24_RS12335	SSP_RS12080
assimilatory sulfite reductase (NADPH) flavoprotein subunit	BEK99_RS11095	SCA_RS00280	SE1039_RS00405	SEQMU2_RS05910	BK815_RS08780	A6V26_RS04615	AA913_RS07035	SXYL_RS12900	BE24_RS12325	SSP_RS12090
phosphoadenosine phosphosulfate reductase	BEK99_RS11100	SCA_RS00275	SE1039_RS00400	SEQMU2_RS05905	BK815_RS08785	A6V26_RS04620	AA913_RS07040	SXYL_RS12905	BE24_RS12320	SSP_RS12095
glycosyl transferase	BEK99_RS11110	SCA_RS00265	SE1039_RS04560	SEQMU2_RS09735	BK815_RS05015	A6V26_RS01175	AA913_RS02755	SXYL_RS08460	BE24_RS02950	SSP_RS08095
glyoxalase	BEK99_RS11130	SCA_RS00245	SE1039_RS11075	SEQMU2_RS02955	BK815_RS11485	A6V26_RS07855	AA913_RS01560	SXYL_RS02010	BE24_RS09775	SSP_RS01870
GMP synthase (glutamine-hydrolyzing)	BEK99_RS11135	SCA_RS00240	SE1039_RS00895	SEQMU2_RS06420	BK815_RS08385	A6V26_RS04220	AA913_RS06640	SXYL_RS12345	BE24_RS12840	SSP_RS11690
IMP dehydrogenase	BEK99_RS11140	SCA_RS00235	SE1039_RS00890	SEQMU2_RS06415	BK815_RS08390	A6V26_RS04225	AA913_RS06645	SXYL_RS12350	BE24_RS12835	SSP_RS11695
xanthine permease	BEK99_RS11145	SCA_RS00230	SE1039_RS00885	SEQMU2_RS06410	BK815_RS08395	A6V26_RS04230	AA913_RS06650	SXYL_RS12355	BE24_RS12830	SSP_RS11700
xanthine phosphoribosyltransferase	BEK99_RS11150	SCA_RS00225	SE1039_RS00880	SEQMU2_RS06405	BK815_RS08400	A6V26_RS04235	AA913_RS06655	SXYL_RS12360	BE24_RS12825	SSP_RS11705
hypothetical protein	BEK99_RS11155	SCA_RS00220	SE1039_RS00875	SEQMU2_RS06400	BK815_RS08405	A6V26_RS04240	AA913_RS06660	SXYL_RS12365	BE24_RS12820	SSP_RS11710
hypothetical protein	BEK99_RS11160	SCA_RS12605	SE1039_RS00870	SEQMU2_RS06395	BK815_RS08410	A6V26_RS04245	AA913_RS06665	SXYL_RS12370	BE24_RS12815	SSP_RS11715
hypothetical protein	BEK99_RS11165	SCA_RS00210	SE1039_RS00865	SEQMU2_RS06390	BK815_RS08415	A6V26_RS04250	AA913_RS06670	SXYL_RS12375	BE24_RS12810	SSP_RS11720
L-cystine transporter	BEK99_RS11170	SCA_RS00205	SE1039_RS00855	SEQMU2_RS06380	BK815_RS08420	A6V26_RS04255	AA913_RS06675	SXYL_RS12380	BE24_RS12805	SSP_RS11725
NADPH-dependent oxidoreductase	BEK99_RS11175	SCA_RS00200	SE1039_RS00845	SEQMU2_RS06370	BK815_RS08430	A6V26_RS04265	AA913_RS06685	SXYL_RS12410	BE24_RS12780	SSP_RS11735
peroxiredoxin	BEK99_RS11185	SCA_RS00190	SE1039_RS00810	SEQMU2_RS06335	BK815_RS08440	A6V26_RS04275	AA913_RS06695	SXYL_RS12420	BE24_RS12770	SSP_RS11745
alkyl hydroperoxide reductase subunit F	BEK99_RS11190	SCA_RS00185	SE1039_RS00805	SEQMU2_RS06330	BK815_RS08445	A6V26_RS04280	AA913_RS06700	SXYL_RS12425	BE24_RS12765	SSP_RS11750
hypothetical protein	BEK99_RS11205	SCA_RS00170	SE1039_RS00800	SEQMU2_RS06325	BK815_RS08450	A6V26_RS04285	AA913_RS06705	SXYL_RS12430	BE24_RS12760	SSP_RS11755
succinyl-diaminopimelate desuccinylase	BEK99_RS11215	SCA_RS00160	SE1039_RS11180	SEQMU2_RS03055	BK815_RS10795	A6V26_RS08570	AA913_RS10045	SXYL_RS01850	BE24_RS09915	SSP_RS01680
histidine phosphatase family protein	BEK99_RS11220	SCA_RS00155	SE1039_RS00790	SEQMU2_RS06315	BK815_RS08460	A6V26_RS04295	AA913_RS06715	SXYL_RS12440	BE24_RS12750	SSP_RS11765
30S ribosomal protein S18	BEK99_RS11240	SCA_RS00135	SE1039_RS00695	SEQMU2_RS06205	BK815_RS08520	A6V26_RS04355	AA913_RS06775	SXYL_RS12560	BE24_RS12610	SSP_RS11910
single-stranded DNA-binding protein	BEK99_RS11245	SCA_RS00130	SE1039_RS00690	SEQMU2_RS06200	BK815_RS08540	A6V26_RS04375	AA913_RS06795	SXYL_RS12580	BE24_RS12585	SSP_RS11915
30S ribosomal protein S6	BEK99_RS11250	SCA_RS00125	SE1039_RS00685	SEQMU2_RS06195	BK815_RS08545	A6V26_RS04380	AA913_RS06800	SXYL_RS12585	BE24_RS12580	SSP_RS11920
ornithine carbamoyltransferase IDENTICAL PARALOGS:	BEK99_RS11270	SCA_RS00105	SE1039_RS11805	SEQMU2_RS03650	BK815_RS10815	A6V26_RS08550	AA913_RS10065	SXYL_RS01230	BE24_RS10495	SSP_RS01130
ornithine carbamoyltransferase	BEK99_RS12875	SCA_RS11340	-	-	-	-	-	-	-	-
GTP-binding protein YchF	BEK99_RS11295	SCA_RS00085	SE1039_RS00675	SEQMU2_RS06185	BK815_RS08555	A6V26_RS04390	AA913_RS06810	SXYL_RS12595	BE24_RS12570	SSP_RS11935
mechanosensitive ion channel protein MscS	BEK99_RS11305	SCA_RS00075	SE1039_RS00665	SEQMU2_RS06175	BK815_RS08565	A6V26_RS04400	AA913_RS06820	SXYL_RS12605	BE24_RS12565	SSP_RS11945
chromosome partitioning protein ParB	BEK99_RS11310	SCA_RS00070	SE1039_RS00385	SEQMU2_RS05890	BK815_RS08795	A6V26_RS04630	AA913_RS07050	SXYL_RS12915	BE24_RS12315	SSP_RS12110
cystathionine gamma-synthase	BEK99_RS11315	SCA_RS00065	SE1039_RS00375	SEQMU2_RS05880	BK815_RS08805	A6V26_RS04640	AA913_RS07060	SXYL_RS12925	BE24_RS12305	SSP_RS12120
bifunctional homocysteine S-methyltransferase/methylenetetrahydrofolate reductase	BEK99_RS11320	SCA_RS00060	SE1039_RS00370	SEQMU2_RS05875	BK815_RS08810	A6V26_RS04645	AA913_RS07065	SXYL_RS12930	BE24_RS12300	SSP_RS12125
metal-dependent hydrolase	BEK99_RS11330	SCA_RS00050	SE1039_RS00360	SEQMU2_RS05865	BK815_RS08820	A6V26_RS04655	AA913_RS07075	SXYL_RS12940	BE24_RS12290	SSP_RS12135
N-succinyldiaminopimelate aminotransferase	BEK99_RS11335	SCA_RS00045	SE1039_RS11435	SEQMU2_RS03290	BK815_RS11135	A6V26_RS08230	AA913_RS04010	SXYL_RS01590	BE24_RS10155	SSP_RS01430

Product	*S. carnosus*		*S. equorum*		*S. succinus*			*S. xylosus*		*S. saprophyticus*
	**JCM 6069**	**TM300**	**KS1039**	**Mu2**	**14BME20**	**CSM 77**	**DSM 14617**	**C2a**	**HKUOPL8**	**ATCC 15305**
nucleoid occlusion protein	BEK99_RS11350	SCA_RS00030	SE1039_RS00030	SEQMU2_RS05625	BK815_RS09495	A6V26_RS05380	AA913_RS12000	SXYL_RS13150	BE24_RS12075	SSP_RS12245
16S rRNA methyltransferase G	BEK99_RS11355	SCA_RS00025	SE1039_RS00025	SEQMU2_RS05620	BK815_RS09500	A6V26_RS05385	AA913_RS12005	SXYL_RS13155	BE24_RS12070	SSP_RS12250
tRNA uridine-5-carboxymethylaminomethyl(34) synthesis enzyme MnmG	BEK99_RS11360	SCA_RS00020	SE1039_RS00020	SEQMU2_RS05615	BK815_RS09505	A6V26_RS05390	AA913_RS12010	SXYL_RS13160	BE24_RS12065	SSP_RS12255
tRNA uridine-5-carboxymethylaminomethyl(34) synthesis GTPase MnmE	BEK99_RS11365	SCA_RS00015	SE1039_RS00015	SEQMU2_RS05610	BK815_RS09510	A6V26_RS05395	AA913_RS12015	SXYL_RS13165	BE24_RS12060	SSP_RS12260
ribonuclease P protein component	BEK99_RS11370	SCA_RS00010	SE1039_RS00010	SEQMU2_RS05605	BK815_RS09515	A6V26_RS05400	AA913_RS12020	SXYL_RS13170	BE24_RS12055	SSP_RS12265
50S ribosomal protein L34	BEK99_RS11375	SCA_RS00005	SE1039_RS00005	SEQMU2_RS05600	BK815_RS09520	A6V26_RS05405	AA913_RS12025	SXYL_RS13175	BE24_RS12050	SSP_RS12270
chromosomal replication initiation protein DnaA	BEK99_RS11380	SCA_RS12430	SE1039_RS13510	SEQMU2_RS05595	BK815_RS09525	A6V26_RS05410	AA913_RS12030	SXYL_RS00005	BE24_RS12045	SSP_RS00005
DNA polymerase III subunit beta	BEK99_RS11385	SCA_RS12425	SE1039_RS13505	SEQMU2_RS05590	BK815_RS09530	A6V26_RS05415	AA913_RS12035	SXYL_RS00010	BE24_RS12040	SSP_RS00010
RNA-binding protein	BEK99_RS11390	SCA_RS12420	SE1039_RS13500	SEQMU2_RS05585	BK815_RS09535	A6V26_RS05420	AA913_RS12040	SXYL_RS00015	BE24_RS12035	SSP_RS00015
DNA replication and repair protein RecF	BEK99_RS11395	SCA_RS12415	SE1039_RS13495	SEQMU2_RS05580	BK815_RS09540	A6V26_RS05425	AA913_RS12045	SXYL_RS00020	BE24_RS12030	SSP_RS00020
DNA gyrase subunit B	BEK99_RS11400	SCA_RS12410	SE1039_RS13490	SEQMU2_RS05575	BK815_RS09545	A6V26_RS05430	AA913_RS12050	SXYL_RS00025	BE24_RS12025	SSP_RS00025
DNA gyrase subunit A	BEK99_RS11405	SCA_RS12405	SE1039_RS13485	SEQMU2_RS05570	BK815_RS09550	A6V26_RS05435	AA913_RS12055	SXYL_RS00030	BE24_RS12020	SSP_RS00030
NAD(P)H-hydrate dehydratase	BEK99_RS11410	SCA_RS12400	SE1039_RS13480	SEQMU2_RS05565	BK815_RS09555	A6V26_RS05440	AA913_RS10790	SXYL_RS00035	BE24_RS12015	SSP_RS00035
histidine ammonia-lyase	BEK99_RS11420	SCA_RS12390	SE1039_RS13475	SEQMU2_RS05560	BK815_RS09560	A6V26_RS05445	AA913_RS10785	SXYL_RS00040	BE24_RS12010	SSP_RS00040
serine--tRNA ligase	BEK99_RS11425	SCA_RS12385	SE1039_RS13470	SEQMU2_RS05555	BK815_RS09565	A6V26_RS05450	AA913_RS10780	SXYL_RS00045	BE24_RS12005	SSP_RS00045
hypothetical protein	BEK99_RS11435	SCA_RS12375	SE1039_RS13450	SEQMU2_RS05535	BK815_RS09585	A6V26_RS05470	AA913_RS10760	SXYL_RS00065	BE24_RS11985	SSP_RS00065
hypothetical protein	BEK99_RS11440	SCA_RS12370	SE1039_RS13445	SEQMU2_RS05530	BK815_RS09590	A6V26_RS05475	AA913_RS10755	SXYL_RS00070	BE24_RS11980	SSP_RS00070
50S ribosomal protein L9	BEK99_RS11445	SCA_RS12365	SE1039_RS13440	SEQMU2_RS05525	BK815_RS09595	A6V26_RS05480	AA913_RS10750	SXYL_RS00075	BE24_RS11975	SSP_RS00075
replicative DNA helicase	BEK99_RS11450	SCA_RS12360	SE1039_RS13435	SEQMU2_RS05520	BK815_RS09600	A6V26_RS05485	AA913_RS10745	SXYL_RS00080	BE24_RS11970	SSP_RS00080
adenylosuccinate synthase	BEK99_RS11455	SCA_RS12355	SE1039_RS13430	SEQMU2_RS05515	BK815_RS09605	A6V26_RS05490	AA913_RS10740	SXYL_RS00085	BE24_RS11965	SSP_RS00085
DNA-binding response regulator	BEK99_RS11475	SCA_RS12335	SE1039_RS13400	SEQMU2_RS05485	BK815_RS09635	A6V26_RS05520	AA913_RS10710	SXYL_RS00105	BE24_RS11945	SSP_RS00115
cell wall metabolism sensor histidine kinase WalK	BEK99_RS11480	SCA_RS12330	SE1039_RS13395	SEQMU2_RS05480	BK815_RS09640	A6V26_RS05525	AA913_RS10705	SXYL_RS00110	BE24_RS11940	SSP_RS00120
hypothetical protein	BEK99_RS11485	SCA_RS12325	SE1039_RS13390	SEQMU2_RS05475	BK815_RS09645	A6V26_RS05530	AA913_RS10700	SXYL_RS00115	BE24_RS11935	SSP_RS00125
hypothetical protein	BEK99_RS11490	SCA_RS12320	SE1039_RS13385	SEQMU2_RS05470	BK815_RS09650	A6V26_RS05535	AA913_RS10695	SXYL_RS00120	BE24_RS11930	SSP_RS00130
MBL fold metallo-hydrolase	BEK99_RS11495	SCA_RS12315	SE1039_RS13380	SEQMU2_RS05465	BK815_RS09665	A6V26_RS05550	AA913_RS10680	SXYL_RS00135	BE24_RS11925	SSP_RS00135
23S rRNA (pseudouridine(1915)-N(3))-methyltransferase RlmH	BEK99_RS11500	SCA_RS12310	SE1039_RS13375	SEQMU2_RS05460	BK815_RS09670	A6V26_RS05555	AA913_RS10675	SXYL_RS00140	BE24_RS11920	SSP_RS00140
MFS transporter	BEK99_RS11895	SCA_RS12270	SE1039_RS00585	SEQMU2_RS06090	BK815_RS08625	A6V26_RS04460	AA913_RS06880	SXYL_RS12800	BE24_RS12425	SSP_RS12010
NAD-dependent protein deacylase	BEK99_RS11940	SCA_RS12225	SE1039_RS12940	SEQMU2_RS04815	BK815_RS09865	A6V26_RS05730	AA913_RS10495	SXYL_RS00325	BE24_RS11385	SSP_RS00465
glyoxalase/bleomycin resistance/dioxygenase family protein	BEK99_RS12040	SCA_RS12175	SE1039_RS11210	SEQMU2_RS03085	BK815_RS11330	A6V26_RS08035	AA913_RS01740	SXYL_RS01820	BE24_RS09945	SSP_RS11655
D-ribose pyranase	BEK99_RS12055	SCA_RS12160	SE1039_RS12490	SEQMU2_RS04285	BK815_RS04015	A6V26_RS00175	AA913_RS03760	SXYL_RS07490	BE24_RS03955	SSP_RS07080

Product	*S. carnosus*		*S. equorum*		*S. succinus*			*S. xylosus*		*S. saprophyticus*
	**JCM 6069**	**TM300**	**KS1039**	**Mu2**	**14BME20**	**CSM 77**	**DSM 14617**	**C2a**	**HKUOPL8**	**ATCC 15305**
acetyl-CoA acetyltransferase	BEK99_RS12070	SCA_RS12110	SE1039_RS00335	SEQMU2_RS05840	BK815_RS08830	A6V26_RS04665	AA913_RS07085	SXYL_RS12970	BE24_RS12260	SSP_RS12165
3-hydroxyacyl-CoA dehydrogenase	BEK99_RS12075	SCA_RS12105	SE1039_RS00330	SEQMU2_RS05835	BK815_RS08835	A6V26_RS04670	AA913_RS07090	SXYL_RS12975	BE24_RS12255	SSP_RS12170
glutaryl-CoA dehydrogenase	BEK99_RS12080	SCA_RS12100	SE1039_RS00325	SEQMU2_RS05830	BK815_RS08840	A6V26_RS04675	AA913_RS07095	SXYL_RS12980	BE24_RS12250	SSP_RS12175
long-chain fatty acid--CoA ligase	BEK99_RS12085	SCA_RS12095	SE1039_RS00320	SEQMU2_RS05825	BK815_RS08845	A6V26_RS04680	AA913_RS07100	SXYL_RS12985	BE24_RS12245	SSP_RS12180
excinuclease ABC subunit UvrA	BEK99_RS12105	SCA_RS12075	SE1039_RS04375	SEQMU2_RS14060	BK815_RS05195	A6V26_RS01335	AA913_RS13140	SXYL_RS08935	BE24_RS02795	SSP_RS08195
DUF896 family protein	BEK99_RS12120	SCA_RS12060	SE1039_RS11490	SEQMU2_RS03345	BK815_RS11075	A6V26_RS08290	AA913_RS04070	SXYL_RS01530	BE24_RS10215	SSP_RS01365
LLM class oxidoreductase	BEK99_RS12125	SCA_RS12055	SE1039_RS11485	SEQMU2_RS03340	BK815_RS11080	A6V26_RS08285	AA913_RS04065	SXYL_RS01535	BE24_RS10210	SSP_RS01370
N-acetylmannosamine-6-phosphate 2-epimerase	BEK99_RS12155	SCA_RS12025	SE1039_RS11110	SEQMU2_RS02990	BK815_RS11445	A6V26_RS07915	AA913_RS01620	SXYL_RS01970	BE24_RS09815	SSP_RS01815
MurR/RpiR family transcriptional regulator	BEK99_RS12160	SCA_RS12020	SE1039_RS11105	SEQMU2_RS02985	BK815_RS11450	A6V26_RS07910	AA913_RS01615	SXYL_RS01975	BE24_RS09810	SSP_RS01820
ROK family protein	BEK99_RS12165	SCA_RS12015	SE1039_RS11100	SEQMU2_RS02980	BK815_RS11455	A6V26_RS07905	AA913_RS01610	SXYL_RS01980	BE24_RS09805	SSP_RS01825
N-acetylneuraminate lyase	BEK99_RS12170	SCA_RS12010	SE1039_RS11095	SEQMU2_RS02975	BK815_RS11460	A6V26_RS07900	AA913_RS01605	SXYL_RS01985	BE24_RS09800	SSP_RS01830
solute:Na+ symporter protein SSS family	BEK99_RS12175	SCA_RS12005	SE1039_RS11090	SEQMU2_RS02970	BK815_RS11465	A6V26_RS07895	AA913_RS01600	SXYL_RS01990	BE24_RS09795	SSP_RS01835
nitroreductase family protein	BEK99_RS12210	SCA_RS11970	SE1039_RS11905	SEQMU2_RS03750	BK815_RS10755	A6V26_RS08610	AA913_RS10005	SXYL_RS01115	BE24_RS10585	SSP_RS01020
phosphohydrolase	BEK99_RS12220	SCA_RS11960	SE1039_RS10675	SEQMU2_RS02555	BK815_RS11905	A6V26_RS07435	AA913_RS01140	SXYL_RS02480	BE24_RS09305	SSP_RS02320
NAD-dependent succinate-semialdehyde dehydrogenase	BEK99_RS12245	SCA_RS11940	SE1039_RS11980	SEQMU2_RS03825	BK815_RS10685	A6V26_RS08680	AA913_RS09935	SXYL_RS01060	BE24_RS10650	SSP_RS00930
oxidoreductase	BEK99_RS12285	SCA_RS11895	SE1039_RS00530	SEQMU2_RS06035	BK815_RS10160	A6V26_RS06000	AA913_RS12565	SXYL_RS00515	BE24_RS11170	SSP_RS02125
citrate transporter	BEK99_RS12305	SCA_RS11875	SE1039_RS03300	SEQMU2_RS08510	BK815_RS06225	A6V26_RS02370	AA913_RS08550	SXYL_RS09950	BE24_RS01775	SSP_RS09430
TetR family transcriptional regulator	BEK99_RS12310	SCA_RS11865	SE1039_RS11325	SEQMU2_RS03200	BK815_RS11220	A6V26_RS08145	AA913_RS01850	SXYL_RS01705	BE24_RS10070	SSP_RS01520
hypothetical protein	BEK99_RS12345	SCA_RS11765	SE1039_RS00505	SEQMU2_RS06010	BK815_RS06640	A6V26_RS02765	AA913_RS08165	SXYL_RS12280	BE24_RS12985	SSP_RS09925
catalase	BEK99_RS12350	SCA_RS11760	SE1039_RS05745	SEQMU2_RS10595	BK815_RS04150	A6V26_RS00310	AA913_RS03625	SXYL_RS07635	BE24_RS03810	SSP_RS07270
carbon starvation protein A	BEK99_RS12495	SCA_RS11625	SE1039_RS00035	SEQMU2_RS05630	BK815_RS09490	A6V26_RS05375	AA913_RS02230	SXYL_RS13145	BE24_RS12080	SSP_RS12240
N-acetyltransferase	BEK99_RS12545	SCA_RS11575	SE1039_RS11055	SEQMU2_RS02935	BK815_RS11495	A6V26_RS07845	AA913_RS01550	SXYL_RS02030	BE24_RS09755	SSP_RS01890
glyoxalase	BEK99_RS12550	SCA_RS11570	SE1039_RS12250	SEQMU2_RS04110	BK815_RS10245	A6V26_RS06085	AA913_RS12765	SXYL_RS00760	BE24_RS10925	SSP_RS00720
hypothetical protein	BEK99_RS12585	SCA_RS11530	SE1039_RS12315	SEQMU2_RS04175	BK815_RS10195	A6V26_RS06035	AA913_RS12600	SXYL_RS00675	BE24_RS11015	SSP_RS00690
methionine sulfoxide reductase A	BEK99_RS12595	SCA_RS11520	SE1039_RS12500	SEQMU2_RS04295	BK815_RS10180	A6V26_RS06020	AA913_RS12585	SXYL_RS00590	BE24_RS11100	SSP_RS00635
LLM class flavin-dependent oxidoreductase	BEK99_RS12625	SCA_RS11490	SE1039_RS05245	SEQMU2_RS10095	BK815_RS04635	A6V26_RS00795	AA913_RS03135	SXYL_RS08110	BE24_RS03310	SSP_RS07755
3-oxoacyl-ACP reductase	BEK99_RS12630	SCA_RS11485	SE1039_RS05250	SEQMU2_RS10100	BK815_RS04630	A6V26_RS00790	AA913_RS03140	SXYL_RS08105	BE24_RS03315	SSP_RS07750
YfcC family protein	BEK99_RS12655	SCA_RS11460	SE1039_RS11225	SEQMU2_RS03100	BK815_RS11315	A6V26_RS08050	AA913_RS01755	SXYL_RS01805	BE24_RS09960	SSP_RS01625
glycerate kinase	BEK99_RS12670	SCA_RS11445	SE1039_RS12045	SEQMU2_RS03890	BK815_RS10605	A6V26_RS08760	AA913_RS09855	SXYL_RS00995	BE24_RS10715	SSP_RS00875
MarR family transcriptional regulator	BEK99_RS12680	SCA_RS11435	SE1039_RS12320	SEQMU2_RS04180	BK815_RS10190	A6V26_RS06030	AA913_RS12595	SXYL_RS00670	BE24_RS11020	SSP_RS00685
malate dehydrogenase (acceptor)	BEK99_RS12700	SCA_RS11415	SE1039_RS12195	SEQMU2_RS04055	BK815_RS10435	A6V26_RS06275	AA913_RS12650	SXYL_RS00830	BE24_RS10870	SSP_RS00765

Product	*S. carnosus*		*S. equorum*		*S. succinus*			*S. xylosus*		*S. saprophyticus*
	**JCM 6069**	**TM300**	**KS1039**	**Mu2**	**14BME20**	**CSM 77**	**DSM 14617**	**C2a**	**HKUOPL8**	**ATCC 15305**
aminobenzoyl-glutamate transporter	BEK99_RS12705	SCA_RS11410	SE1039_RS10825	SEQMU2_RS02705	BK815_RS11735	A6V26_RS07605	AA913_RS01310	SXYL_RS02310	BE24_RS09475	SSP_RS02120
sodium/proline symporter	BEK99_RS12725	SCA_RS11390	SE1039_RS11005	SEQMU2_RS02885	BK815_RS11565	A6V26_RS07775	AA913_RS01480	SXYL_RS02090	BE24_RS09695	SSP_RS01950
acyl--CoA ligase	BEK99_RS12730	SCA_RS11385	SE1039_RS00590	SEQMU2_RS06095	BK815_RS08620	A6V26_RS04455	AA913_RS06875	SXYL_RS12795	BE24_RS12430	SSP_RS12000
Na+/H+ antiporter NhaC	BEK99_RS12740	SCA_RS11375	SE1039_RS11250	SEQMU2_RS03125	BK815_RS11290	A6V26_RS08075	AA913_RS01780	SXYL_RS01780	BE24_RS09985	SSP_RS01600
hypothetical protein	BEK99_RS00010	SCA_RS11200	-	-	-	-	-	-	-	-
hypothetical protein	BEK99_RS00015	SCA_RS11195	-	-	-	-	-	-	-	-
deferrochelatase/peroxidase EfeB	BEK99_RS00040	SCA_RS11170	-	-	-	-	-	-	-	-
hypothetical protein	BEK99_RS00045	SCA_RS11165	-	-	-	-	-	-	-	-
twin-arginine translocase subunit TatC	BEK99_RS00050	SCA_RS11160	-	-	-	-	-	-	-	-
twin-arginine translocase TatA/TatE family subunit	BEK99_RS00055	SCA_RS11155	-	-	-	-	-	-	-	-
catalase HPII	BEK99_RS00065	SCA_RS11145	SE1039_RS00835	SEQMU2_RS06360	BK815_RS08435	A6V26_RS04270	AA913_RS06690	SXYL_RS12415	BE24_RS12775	-
hypothetical protein	BEK99_RS00070	SCA_RS11140	-	-	-	-	-	-	-	-
C4-dicarboxylate ABC transporter	BEK99_RS00075	SCA_RS11135	-	-	-	-	-	-	-	-
hypothetical protein	BEK99_RS00080	SCA_RS12575	-	-	-	-	-	-	-	-
hypothetical protein	BEK99_RS00085	SCA_RS12570	-	-	-	-	-	-	-	-
hypothetical protein	BEK99_RS00090	SCA_RS12565	-	-	-	-	-	-	-	-
hypothetical protein	BEK99_RS00095	SCA_RS12560	-	-	-	-	-	-	-	-
accessory Sec system protein translocase subunit SecY2	BEK99_RS00120	SCA_RS11115	-	-	-	-	AA913_RS13295	-	-	-
accessory Sec system protein Asp1	BEK99_RS00125	SCA_RS11110	-	-	-	-	AA913_RS13290	-	-	-
accessory Sec system protein Asp2	BEK99_RS00130	SCA_RS11105	-	-	-	-	AA913_RS13285	-	-	-
accessory Sec system protein Asp3	BEK99_RS00135	SCA_RS11100	-	-	-	-	-	-	-	-
accessory Sec system translocase SecA2	BEK99_RS00140	SCA_RS11095	-	-	-	-	AA913_RS13275	-	-	-
accessory Sec system glycosyltransferase GtfA	BEK99_RS00145	-	SE1039_RS12795	-	BK815_RS10370	A6V26_RS06210	AA913_RS13270	-	-	SSP_RS00550
accessory Sec system glycosylation chaperone GtfB	BEK99_RS00150	SCA_RS11085	-	-	BK815_RS10375	A6V26_RS06215	AA913_RS12710	-	-	-
hypothetical protein	BEK99_RS00155	SCA_RS11080	-	-	-	-	-	-	-	-
hypothetical protein	BEK99_RS00185	SCA_RS11060	-	-	-	-	-	-	-	-
methionine ABC transporter ATP-binding protein	BEK99_RS00190	SCA_RS11055	-	-	BK815_RS10315	A6V26_RS06155	AA913_RS12835	SXYL_RS12725	-	-
methionine ABC transporter permease	BEK99_RS00195	SCA_RS11050	-	-	BK815_RS10320	A6V26_RS06160	AA913_RS12840	SXYL_RS12730	-	-
methionine ABC transporter substrate-binding protein	BEK99_RS00200	SCA_RS11045	-	-	BK815_RS10325	A6V26_RS06165	AA913_RS12845	SXYL_RS12735	-	-
alpha/beta hydrolase	BEK99_RS00210	SCA_RS11035	-	-	-	-	-	-	-	-
AraC family transcriptional regulator	BEK99_RS00215	SCA_RS11030	-	-	-	-	-	-	-	-

Product	*S. carnosus*		*S. equorum*		*S. succinus*			*S. xylosus*		*S. saprophyticus*
	**JCM 6069**	**TM300**	**KS1039**	**Mu2**	**14BME20**	**CSM 77**	**DSM 14617**	**C2a**	**HKUOPL8**	**ATCC 15305**
hypothetical protein	BEK99_RS00220	SCA_RS11025	-	-	-	-	-	-	-	-
hypothetical protein	BEK99_RS00225	SCA_RS11020	SE1039_RS13370	-	-	-	-	-	-	-
CDP-glycerol glycerophosphotransferase family protein	BEK99_RS00230	SCA_RS12540	-	-	-	-	-	-	-	-
hypothetical protein	BEK99_RS00235	SCA_RS11010	-	-	-	-	-	-	-	-
hypothetical protein	BEK99_RS00240	SCA_RS11005	-	-	-	-	-	-	-	-
teichoic acid biosynthesis protein	BEK99_RS00245	SCA_RS11000	-	-	-	-	-	-	-	-
glycosyl transferase family 1	BEK99_RS00255	SCA_RS10990	-	-	-	-	-	-	-	-
TetR family transcriptional regulator	BEK99_RS00285	SCA_RS10960	-	-	-	-	-	-	-	-
alpha/beta hydrolase	BEK99_RS00290	SCA_RS10955	-	-	-	-	-	-	-	-
hypothetical protein	BEK99_RS00300	SCA_RS10945	-	-	-	-	-	-	-	-
hypothetical protein	BEK99_RS00305	SCA_RS10940	-	-	-	-	-	-	-	-
hypothetical protein	BEK99_RS00310	SCA_RS10935	-	-	-	-	-	-	-	-
hypothetical protein	BEK99_RS00350	SCA_RS10895	-	-	-	-	-	-	-	-
NAD(P)-dependent oxidoreductase	BEK99_RS00355	SCA_RS10890	-	-	-	-	-	-	-	-
sugar transferase	BEK99_RS00360	SCA_RS10885	-	SEQMU2_RS05350	-	-	-	-	BE24_RS11765	SSP_RS00360
hypothetical protein	BEK99_RS00365	SCA_RS10880	-	-	-	-	-	-	-	-
oligosaccharide repeat unit polymerase	BEK99_RS00370	SCA_RS10875	-	-	-	-	-	-	-	-
hypothetical protein	BEK99_RS00375	SCA_RS10870	-	-	-	-	-	-	-	-
sulfatase	BEK99_RS00380	SCA_RS10865	-	-	-	-	-	-	-	-
TetR/AcrR family transcriptional regulator	BEK99_RS00385	SCA_RS10860	-	-	-	-	-	-	-	-
hypothetical protein	BEK99_RS00405	SCA_RS10845	SE1039_RS11965	SEQMU2_RS03810	-	-	-	-	-	-
(deoxy)nucleoside triphosphate pyrophosphohydrolase	BEK99_RS00410	SCA_RS10840	SE1039_RS12640	SEQMU2_RS04520	-	-	-	-	-	-
NgoFVII family restriction endonuclease	BEK99_RS00415	SCA_RS10835	SE1039_RS12645	SEQMU2_RS04525	-	-	-	-	-	-
topology modulation protein	BEK99_RS00420	SCA_RS10830	SE1039_RS03160	SEQMU2_RS08370	-	-	-	-	-	-
MOSC domain-containing protein	BEK99_RS00435	SCA_RS10815	-	-	-	-	-	-	-	-
FmdB family regulatory protein	BEK99_RS00440	SCA_RS10810	SE1039_RS00640	SEQMU2_RS06150	-	-	-	-	-	-
DUF871 domain-containing protein	BEK99_RS00455	SCA_RS10795	-	-	-	-	-	-	-	-
MurR/RpiR family transcriptional regulator	BEK99_RS00470	SCA_RS10780	-	-	-	-	-	-	-	-
alpha/beta hydrolase	BEK99_RS00475	SCA_RS10775	-	-	-	-	-	-	-	-
formate dehydrogenase	BEK99_RS00480	SCA_RS10770	-	-	-	-	-	-	-	-
hypothetical protein	BEK99_RS00485	SCA_RS10765	-	-	-	-	-	-	-	-

Product	*S. carnosus*		*S. equorum*		*S. succinus*			*S. xylosus*		*S. saprophyticus*
	**JCM 6069**	**TM300**	**KS1039**	**Mu2**	**14BME20**	**CSM 77**	**DSM 14617**	**C2a**	**HKUOPL8**	**ATCC 15305**
hypothetical protein	BEK99_RS00490	SCA_RS10760	-	-	-	-	-	-	-	-
5,10-methylene-tetrahydrofolate dehydrogenase	BEK99_RS00495	SCA_RS10755	SE1039_RS10985	SEQMU2_RS02865	-	-	-	SXYL_RS02120	BE24_RS09665	SSP_RS01975
hypothetical protein	BEK99_RS00505	SCA_RS10745	-	-	-	-	-	-	-	-
cation:proton antiporter	BEK99_RS00515	SCA_RS10735	SE1039_RS02300	SEQMU2_RS07085	-	A6V26_RS03330	AA913_RS07605	SXYL_RS10895	BE24_RS00835	SSP_RS10490
hypothetical protein	BEK99_RS00550	SCA_RS10700	-	-	-	-	-	-	-	-
alanine glycine permease	BEK99_RS00560	SCA_RS10690	-	-	-	-	-	-	-	-
hypothetical protein	BEK99_RS00565	SCA_RS12535	-	-	-	-	-	-	-	-
hypothetical protein	BEK99_RS00570	SCA_RS10685	-	-	-	-	-	-	-	-
ABC transporter permease	BEK99_RS00580	SCA_RS10675	SE1039_RS00490	SEQMU2_RS05995	-	-	-	-	-	-
alkylphosphonate utilization protein	BEK99_RS00585	SCA_RS10670	-	-	BK815_RS09085	A6V26_RS04920	AA913_RS07340	SXYL_RS11340	BE24_RS00430	SSP_RS10840
hypothetical protein	BEK99_RS00590	SCA_RS10665	-	SEQMU2_RS14035	BK815_RS09320	A6V26_RS05140	AA913_RS01995	-	-	-
glycerol-3-phosphate cytidylyltransferase	BEK99_RS00620	SCA_RS10635	-	SEQMU2_RS07175	-	-	-	-	-	-
amino acid permease	BEK99_RS00630	SCA_RS10625	-	-	-	-	-	-	-	-
2-dehydropantoate 2-reductase	BEK99_RS00635	SCA_RS10620	SE1039_RS12130	SEQMU2_RS03975	BK815_RS10520	A6V26_RS08845	AA913_RS09770	-	-	-
aldehyde dehydrogenase	BEK99_RS00640	SCA_RS10615	SE1039_RS12190	SEQMU2_RS13960	BK815_RS10445	A6V26_RS06285	AA913_RS12640	SXYL_RS00845	BE24_RS10855	-
aldehyde dehydrogenase	BEK99_RS00645	SCA_RS10610	-	-	-	-	-	-	-	-
CoA-disulfide reductase	BEK99_RS00655	SCA_RS10600	-	-	-	-	-	-	-	-
transcriptional regulator	BEK99_RS00665	SCA_RS10590	-	-	-	-	-	-	-	-
ATPase	BEK99_RS00680	SCA_RS10575	SE1039_RS00110	SEQMU2_RS05700	BK815_RS10535	A6V26_RS08830	AA913_RS09785	SXYL_RS00600	BE24_RS11090	-
hypothetical protein	BEK99_RS00695	SCA_RS10560	-	-	-	-	-	-	-	-
hypothetical protein	BEK99_RS00705	SCA_RS10550	-	-	-	-	-	-	-	-
CoA ligase	BEK99_RS00710	SCA_RS10545	-	-	-	-	-	-	-	-
hypothetical protein	BEK99_RS00720	SCA_RS10535	-	-	-	-	-	-	-	-
arginine deiminase	BEK99_RS00725	SCA_RS10530	-	-	-	-	-	-	-	-
arginine deiminase	BEK99_RS00730	SCA_RS10525	-	-	-	-	-	-	-	-
arginine-ornithine antiporter	BEK99_RS00735	SCA_RS10520	-	-	-	-	-	-	-	-
Crp/Fnr family transcriptional regulator	BEK99_RS00740	SCA_RS10515	-	-	-	-	-	-	-	-
transcriptional regulator protein	BEK99_RS00745	SCA_RS10510	-	-	-	-	-	-	-	-
hypothetical protein	BEK99_RS00750	SCA_RS10505	-	-	-	-	-	-	-	-
thiazole synthase	BEK99_RS00760	SCA_RS10495	SE1039_RS12285	SEQMU2_RS04145	BK815_RS10230	A6V26_RS06070	AA913_RS12750	SXYL_RS00710	BE24_RS10970	-
thiamine biosynthesis protein ThiS	BEK99_RS00765	SCA_RS10490	SE1039_RS12290	SEQMU2_RS04150	BK815_RS10225	A6V26_RS06065	AA913_RS12745	SXYL_RS00705	BE24_RS10975	-

Product	S. carnosus		S. equorum		S. succinus			S. xylosus		S. saprophyticus
	JCM 6069	TM300	KS1039	Mu2	14BME20	CSM 77	DSM 14617	C2a	HKUOPL8	ATCC 15305
glycine oxidase ThiO	BEK99_RS00770	SCA_RS10485	SE1039_RS12295	SEQMU2_RS04155	BK815_RS10220	A6V26_RS06060	-	SXYL_RS00700	BE24_RS10980	-
thiamine phosphate synthase	BEK99_RS00775	SCA_RS10480	SE1039_RS12300	SEQMU2_RS04160	BK815_RS10215	A6V26_RS06055	AA913_RS12620	SXYL_RS00695	BE24_RS10985	-
serine protease	BEK99_RS00780	SCA_RS10475	-	-	-	-	-	-	-	-
hypothetical protein	BEK99_RS00805	SCA_RS10450	-	-	-	-	-	-	-	-
hypothetical protein	BEK99_RS00810	SCA_RS10445	-	-	-	-	-	-	-	-
serine/threonine protein phosphatase	BEK99_RS00820	SCA_RS10435	SE1039_RS12635	SEQMU2_RS04515	BK815_RS10010	A6V26_RS05850	AA913_RS10375	SXYL_RS00300	BE24_RS11400	-
hypothetical protein	BEK99_RS00825	SCA_RS10430	SE1039_RS12630	SEQMU2_RS04510	BK815_RS10015	A6V26_RS05855	AA913_RS10370	SXYL_RS00305	BE24_RS11395	-
NAD-dependent dehydratase	BEK99_RS00830	SCA_RS10425	-	-	-	-	-	-	-	-
hypothetical protein	BEK99_RS13025	SCA_RS12700	-	-	-	-	-	-	-	-
hypothetical protein	BEK99_RS00835	SCA_RS10420	-	-	-	-	-	-	-	-
Zn-dependent hydrolase	BEK99_RS00840	SCA_RS10415	-	-	BK815_RS08335	A6V26_RS04170	AA913_RS06590	SXYL_RS12235	BE24_RS13035	-
PucR family transcriptional regulator	BEK99_RS00845	SCA_RS10410	-	-	BK815_RS08355	A6V26_RS04190	AA913_RS06610	SXYL_RS12295	BE24_RS12970	-
allantoin permease	BEK99_RS00850	SCA_RS10405	-	-	BK815_RS08350	A6V26_RS04185	AA913_RS06605	SXYL_RS12290	BE24_RS12975	-
hypothetical protein	BEK99_RS00890	SCA_RS10290	-	-	BK815_RS09820	A6V26_RS13490	AA913_RS14175	-	-	-
N-acetyltransferase	BEK99_RS00895	SCA_RS10285	SE1039_RS01635	-	BK815_RS07675	A6V26_RS03795	AA913_RS10975	SXYL_RS11555	BE24_RS00180	SSP_RS11005
serine/threonine protein phosphatase	BEK99_RS00900	SCA_RS10280	-	-	-	-	-	-	-	-
hypothetical protein	BEK99_RS00905	SCA_RS10275	-	-	-	-	-	-	-	-
PadR family transcriptional regulator	BEK99_RS00910	SCA_RS10270	SE1039_RS10590	SEQMU2_RS02470	-	-	-	SXYL_RS02555	BE24_RS09220	SSP_RS02405
FeoB-associated Cys-rich membrane protein	BEK99_RS13035	SCA_RS12695	SE1039_RS13515	-	-	-	-	-	BE24_RS13665	-
ferrous iron transport protein B	BEK99_RS00915	SCA_RS10265	SE1039_RS00060	SEQMU2_RS05655	-	-	-	-	BE24_RS12115	-
ferrous iron transport protein A	BEK99_RS00920	SCA_RS10260	SE1039_RS00065	SEQMU2_RS05660	-	-	-	-	BE24_RS12120	SSP_RS12220
hypothetical protein	BEK99_RS00925	SCA_RS10255	-	-	-	-	-	-	-	-
polysaccharide biosynthesis protein	BEK99_RS00930	SCA_RS10250	-	SEQMU2_RS05390	-	-	-	-	BE24_RS11815	SSP_RS00320
UTP--glucose-1-phosphate uridylyltransferase	BEK99_RS00935	SCA_RS10245	SE1039_RS13250	SEQMU2_RS07445	BK815_RS06840	-	-	SXYL_RS10530	BE24_RS01205	SSP_RS10120
hypothetical protein	BEK99_RS00945	SCA_RS10235	-	-	-	-	-	-	-	-
DNA-binding protein	BEK99_RS00955	SCA_RS10225	-	-	-	-	-	-	-	-
formate acetyltransferase	BEK99_RS00970	SCA_RS10210	-	-	-	-	-	SXYL_RS05045	BE24_RS06500	-
hypothetical protein	BEK99_RS00985	SCA_RS10195	-	-	-	-	-	-	-	-
NADPH:quinone reductase	BEK99_RS00990	SCA_RS10190	-	-	-	-	-	-	-	-
PTS sugar transporter subunit IIC	BEK99_RS01005	SCA_RS10175	SE1039_RS11970	SEQMU2_RS03815	-	-	-	SXYL_RS01070	BE24_RS10640	SSP_RS00940
polyphosphate--AMP phosphotransferase	BEK99_RS01015	SCA_RS10165	-	-	-	-	-	-	-	-

Product	S. carnosus		S. equorum		S. succinus			S. xylosus		S. saprophyticus
	JCM 6069	TM300	KS1039	Mu2	14BME20	CSM 77	DSM 14617	C2a	HKUOPL8	ATCC 15305
cation transporter	BEK99_RS01020	SCA_RS10160	-	-	-	-	-	-	-	-
FMN-dependent NADH-azoreductase	BEK99_RS01025	SCA_RS10155	-	-	-	-	-	-	-	-
hypothetical protein	BEK99_RS01030	SCA_RS10150	-	-	-	-	-	-	-	-
hypothetical protein	BEK99_RS01035	SCA_RS10145	-	-	-	-	-	-	-	-
biotin synthase BioB	BEK99_RS01045	SCA_RS10135	SE1039_RS12860	SEQMU2_RS04700	BK815_RS10830	A6V26_RS08535	AA913_RS10080	SXYL_RS12760	BE24_RS12470	-
hypothetical protein	BEK99_RS01050	SCA_RS10130	-	-	-	-	-	-	-	-
alpha/beta hydrolase	BEK99_RS01060	SCA_RS10120	-	-	BK815_RS08330	A6V26_RS04165	AA913_RS06585	SXYL_RS12230	BE24_RS13040	-
hypothetical protein	BEK99_RS01065	SCA_RS10115	-	-	-	-	-	SXYL_RS12790	BE24_RS12440	SSP_RS11995
RND transporter	BEK99_RS01070	SCA_RS10110	-	-	BK815_RS09990	A6V26_RS05830	AA913_RS10395	SXYL_RS12750	-	-
ABC transporter permease	BEK99_RS01080	SCA_RS10100	-	-	-	-	-	SXYL_RS12740	BE24_RS12490	-
acyl-CoA thioester hydrolase	BEK99_RS01115	SCA_RS10065	-	-	-	-	-	SXYL_RS01830	-	SSP_RS01650
branched-chain amino acid ABC transporter permease	BEK99_RS01120	SCA_RS10060	-	-	-	-	-	-	-	-
branched-chain amino acid transporter AzlD	BEK99_RS01125	SCA_RS10055	-	-	-	-	-	-	-	-
nickel transporter NixA	BEK99_RS01130	SCA_RS10050	-	-	-	-	-	-	-	-
LacI family transcriptional regulator	BEK99_RS01140	SCA_RS10040	-	-	-	-	-	SXYL_RS01925	-	SSP_RS01760
hypothetical protein	BEK99_RS01145	SCA_RS10035	-	-	-	-	-	-	-	-
transcriptional regulator	BEK99_RS01150	-	SE1039_RS11355	SEQMU2_RS03230	BK815_RS11195	A6V26_RS08170	AA913_RS03950	SXYL_RS12400	BE24_RS12790	SSP_RS01495
L-serine dehydratase iron-sulfur-dependent subunit beta	BEK99_RS01155	SCA_RS10025	-	-	-	-	-	SXYL_RS12395	BE24_RS12795	-
flavodoxin family protein	BEK99_RS01165	SCA_RS10015	-	-	BK815_RS11470	A6V26_RS07890	AA913_RS01595	SXYL_RS01995	BE24_RS09790	SSP_RS01840
lactate dehydrogenase	BEK99_RS01175	SCA_RS10005	-	-	-	-	-	-	-	-
ABC transporter ATP-binding protein	BEK99_RS01185	SCA_RS09995	SE1039_RS11060	SEQMU2_RS02940	-	-	-	SXYL_RS02025	BE24_RS09760	SSP_RS01885
acetyltransferase	BEK99_RS01190	SCA_RS09990	-	-	-	-	-	-	-	-
ABC transporter ATP-binding protein	BEK99_RS01230	SCA_RS09950	SE1039_RS12780	-	BK815_RS09965	-	-	SXYL_RS00420	-	-
ABC-2 transporter family protein	BEK99_RS01235	SCA_RS09945	-	-	-	-	-	-	-	-
DedA family protein	BEK99_RS01240	SCA_RS09940	-	-	BK815_RS11570	A6V26_RS07770	AA913_RS01475	SXYL_RS02095	BE24_RS09690	SSP_RS01955
hypothetical protein	BEK99_RS01265	SCA_RS09915	SE1039_RS10975	SEQMU2_RS02855	BK815_RS11595	A6V26_RS07745	AA913_RS01450	SXYL_RS02130	-	SSP_RS01985
GTP-binding protein	BEK99_RS01270	SCA_RS09910	SE1039_RS12915	SEQMU2_RS04790	BK815_RS10905	A6V26_RS08460	AA913_RS04240	-	-	-
ATP-dependent Clp protease ATP-binding subunit	BEK99_RS01280	SCA_RS09900	-	-	-	-	-	-	-	-
protease	BEK99_RS01290	SCA_RS09890	-	-	BK815_RS08880	A6V26_RS04715	AA913_RS07135	SXYL_RS07705	BE24_RS03710	SSP_RS11870
hypothetical protein	BEK99_RS01310	SCA_RS09870	-	-	-	-	-	-	-	-
acetylornithine deacetylase	BEK99_RS01315	SCA_RS09865	-	-	-	-	-	-	-	-

Product	*S. carnosus*		*S. equorum*		*S. succinus*			*S. xylosus*		*S. saprophyticus*
	**JCM 6069**	**TM300**	**KS1039**	**Mu2**	**14BME20**	**CSM 77**	**DSM 14617**	**C2a**	**HKUOPL8**	**ATCC 15305**
exopolyphosphatase	BEK99_RS01325	SCA_RS09855	SE1039_RS10830	SEQMU2_RS02710	BK815_RS11730	A6V26_RS07610	AA913_RS01315	SXYL_RS02305	-	SSP_RS02115
lipoprotein	BEK99_RS01330	SCA_RS09850	-	-	-	-	-	-	-	-
CPBP family intramembrane metalloprotease	BEK99_RS01335	SCA_RS09845	SE1039_RS10805	SEQMU2_RS02685	BK815_RS11785	A6V26_RS07555	AA913_RS01260	SXYL_RS02335	-	SSP_RS02145
CPBP family intramembrane metalloprotease	BEK99_RS01340	SCA_RS09840	-	-	-	-	-	-	-	-
carboxylesterase/lipase family protein	BEK99_RS01360	SCA_RS09820	-	-	-	-	-	-	-	-
hypothetical protein	BEK99_RS01370	SCA_RS09810	SE1039_RS10780	SEQMU2_RS02660	BK815_RS11810	A6V26_RS07530	AA913_RS01235	SXYL_RS02365	BE24_RS09420	-
glycine/betaine ABC transporter ATP-binding protein	BEK99_RS01375	SCA_RS09805	SE1039_RS10755	SEQMU2_RS02635	BK815_RS11835	A6V26_RS07505	AA913_RS01210	SXYL_RS02390	-	SSP_RS02200
transcriptional regulator	BEK99_RS01400	SCA_RS09780	-	-	-	-	-	-	-	-
hypothetical protein	BEK99_RS01405	SCA_RS09775	-	-	-	-	-	-	-	-
hypothetical protein	BEK99_RS01410	SCA_RS09770	-	-	-	-	-	-	-	-
antiholin	BEK99_RS01415	SCA_RS09765	-	-	BK815_RS11860	A6V26_RS07480	AA913_RS01185	SXYL_RS02415	BE24_RS09370	SSP_RS02250
murein hydrolase transporter LrgA	BEK99_RS01420	SCA_RS09760	-	-	BK815_RS11865	A6V26_RS07475	AA913_RS01180	SXYL_RS02420	BE24_RS09365	SSP_RS02255
DNA-binding response regulator	BEK99_RS01425	SCA_RS09755	-	-	BK815_RS11870	A6V26_RS07470	AA913_RS01175	SXYL_RS02425	BE24_RS09360	SSP_RS02260
histidine kinase	BEK99_RS01430	SCA_RS09750	-	-	BK815_RS11875	A6V26_RS07465	AA913_RS01170	SXYL_RS02430	BE24_RS09355	SSP_RS02265
hypothetical protein	BEK99_RS01445	SCA_RS09740	-	-	-	-	-	-	-	-
amino acid permease	BEK99_RS01450	SCA_RS09735	-	-	-	-	-	-	-	-
5-nucleotidase lipoprotein e(P4) family	BEK99_RS01455	SCA_RS09730	-	-	BK815_RS07645	A6V26_RS03765	AA913_RS11005	SXYL_RS00310	BE24_RS11390	-
gamma-aminobutyrate permease	BEK99_RS01460	SCA_RS09725	-	-	BK815_RS08695	A6V26_RS04530	AA913_RS06950	-	-	-
amino acid ABC transporter substrate-binding protein	BEK99_RS01495	SCA_RS09690	-	-	BK815_RS10840	A6V26_RS08525	AA913_RS10090	SXYL_RS01290	BE24_RS10465	SSP_RS01180
amino acid ABC transporter permease	BEK99_RS01500	SCA_RS09685	-	-	BK815_RS10845	A6V26_RS08520	AA913_RS10095	SXYL_RS01295	BE24_RS10460	SSP_RS01185
amino acid ABC transporter ATP-binding protein	BEK99_RS01505	SCA_RS09680	-	-	BK815_RS10850	A6V26_RS08515	AA913_RS10100	SXYL_RS01300	BE24_RS10455	SSP_RS01190
pyridine nucleotide-disulfide oxidoreductase	BEK99_RS01515	SCA_RS09670	-	-	-	-	-	-	-	-
ferrous iron transporter B	BEK99_RS01520	SCA_RS09665	-	-	-	-	-	-	-	-
GTP-binding protein	BEK99_RS01525	SCA_RS09660	-	-	BK815_RS10910	A6V26_RS08455	AA913_RS04235	-	-	-
hypothetical protein	BEK99_RS01540	SCA_RS09645	-	SEQMU2_RS02455	-	-	-	-	-	-
NirR protein	BEK99_RS01550	SCA_RS09635	-	-	-	-	-	-	-	-
preprotein translocase subunit TatB	BEK99_RS01555	SCA_RS09630	SE1039_RS10555	SEQMU2_RS02415	-	-	-	SXYL_RS02605	BE24_RS09170	-
nitrite reductase large subunit	BEK99_RS01560	SCA_RS09625	SE1039_RS10550	SEQMU2_RS02410	-	-	-	SXYL_RS02610	BE24_RS09165	-
nitrite reductase (NAD(P)H) small subunit	BEK99_RS01565	SCA_RS09620	SE1039_RS10545	SEQMU2_RS02405	-	-	-	SXYL_RS02615	BE24_RS09160	-
uroporphyrinogen-III C-methyltransferase	BEK99_RS01570	SCA_RS09615	SE1039_RS10540	SEQMU2_RS02400	-	-	-	SXYL_RS02620	BE24_RS09155	-
nitrate reductase subunit alpha	BEK99_RS01575	SCA_RS09610	SE1039_RS10535	SEQMU2_RS02395	-	-	-	SXYL_RS02630	BE24_RS09145	-

Product	*S. carnosus*		*S. equorum*		*S. succinus*			*S. xylosus*		*S. saprophyticus*
	JCM 6069	TM300	KS1039	Mu2	14BME20	CSM 77	DSM 14617	C2a	HKUOPL8	ATCC 15305
nitrate reductase subunit beta	BEK99_RS01580	SCA_RS09605	SE1039_RS10530	SEQMU2_RS02390	-	-	-	SXYL_RS02635	BE24_RS09140	-
nitrate reductase molybdenum cofactor assembly chaperone	BEK99_RS01585	SCA_RS09600	SE1039_RS10525	SEQMU2_RS02385	-	-	-	SXYL_RS02640	BE24_RS09135	-
nitrate reductase	BEK99_RS01590	SCA_RS09595	SE1039_RS10520	SEQMU2_RS02380	-	-	-	SXYL_RS02645	-	-
GAF domain-containing protein	BEK99_RS01595	SCA_RS09590	SE1039_RS10515	SEQMU2_RS02375	-	-	-	SXYL_RS02650	BE24_RS09125	-
sensor histidine kinase	BEK99_RS01600	SCA_RS09585	SE1039_RS10510	SEQMU2_RS02370	-	-	-	SXYL_RS02655	BE24_RS09120	-
DNA-binding response regulator	BEK99_RS01605	SCA_RS09580	SE1039_RS10505	-	-	-	-	SXYL_RS02660	BE24_RS09115	-
NarK/NasA family nitrate transporter	BEK99_RS01610	SCA_RS09575	SE1039_RS10500	SEQMU2_RS02360	-	-	-	SXYL_RS02665	BE24_RS09110	-
cell division protein	BEK99_RS01615	SCA_RS09570	SE1039_RS00460	SEQMU2_RS05965	BK815_RS08615	A6V26_RS04450	AA913_RS06870	SXYL_RS05015	BE24_RS06530	-
EamA family transporter	BEK99_RS01650	-	-	SEQMU2_RS14050	-	-	-	-	-	SSP_RS08180
proton/sodium-glutamate symport protein GltT	BEK99_RS01655	SCA_RS09530	-	-	BK815_RS12035	A6V26_RS07305	AA913_RS01005	SXYL_RS12340	BE24_RS12945	SSP_RS11660
Rossman fold protein TIGR00730 family	BEK99_RS01665	SCA_RS09520	SE1039_RS05905	-	BK815_RS06375	-	-	-	-	-
MarR family transcriptional regulator	BEK99_RS01675	SCA_RS12530	-	-	-	-	-	-	-	-
TetR family transcriptional regulator	BEK99_RS01770	SCA_RS09415	SE1039_RS10295	SEQMU2_RS02155	BK815_RS12230	A6V26_RS07115	AA913_RS00815	SXYL_RS02885	-	SSP_RS02660
hypothetical protein	BEK99_RS01785	SCA_RS09400	SE1039_RS10280	SEQMU2_RS02140	BK815_RS12245	A6V26_RS07100	AA913_RS00800	SXYL_RS02900	BE24_RS08890	-
hypothetical protein	BEK99_RS01790	SCA_RS09395	-	-	-	-	-	-	-	-
hypothetical protein	BEK99_RS01810	SCA_RS09375	-	-	-	-	-	-	-	-
anaerobic ribonucleoside-triphosphate reductase	BEK99_RS01815	SCA_RS09370	SE1039_RS12275	SEQMU2_RS04135	-	-	-	-	BE24_RS10960	SSP_RS00705
anaerobic ribonucleoside-triphosphate reductase activating protein	BEK99_RS01820	-	SE1039_RS12270	SEQMU2_RS04130	-	-	-	SXYL_RS00735	BE24_RS10955	SSP_RS00710
DUF805 domain-containing protein	BEK99_RS01830	SCA_RS09355	SE1039_RS10235	SEQMU2_RS02095	-	A6V26_RS07060	AA913_RS00760	-	-	SSP_RS02730
DUF805 domain-containing protein	BEK99_RS01835	SCA_RS09345	-	-	-	-	-	-	-	-
DUF805 domain-containing protein	BEK99_RS01840	SCA_RS09340	-	-	-	-	-	-	-	-
transcriptional regulator	BEK99_RS01845	SCA_RS09335	SE1039_RS12770	SEQMU2_RS03855	BK815_RS09955	-	-	SXYL_RS00430	-	-
hypothetical protein	BEK99_RS01850	SCA_RS09330	-	-	-	-	-	-	-	-
hypothetical protein	BEK99_RS01865	SCA_RS09315	-	-	-	-	-	-	-	-
formimidoylglutamase	BEK99_RS01885	SCA_RS09295	SE1039_RS10200	SEQMU2_RS02060	BK815_RS12325	A6V26_RS07020	AA913_RS00720	SXYL_RS03000	BE24_RS08805	-
histidine transporter	BEK99_RS01895	SCA_RS09285	SE1039_RS10190	SEQMU2_RS02050	BK815_RS09905	A6V26_RS05770	AA913_RS10455	SXYL_RS03010	BE24_RS08795	-
glycerol-3-phosphate transporter	BEK99_RS01940	SCA_RS09240	-	-	BK815_RS09110	A6V26_RS04945	AA913_RS07365	-	BE24_RS12085	-
MurR/RpiR family transcriptional regulator	BEK99_RS01980	-	SE1039_RS10090	SEQMU2_RS01950	BK815_RS12425	A6V26_RS06920	AA913_RS00620	SXYL_RS03110	BE24_RS08695	SSP_RS02885
hypothetical protein	BEK99_RS01985	SCA_RS09195	-	-	-	-	-	-	-	-
CPBP family intramembrane metalloprotease	BEK99_RS01990	SCA_RS09190	-	-	BK815_RS12450	A6V26_RS06895	AA913_RS00595	-	-	-
hypothetical protein	BEK99_RS02010	SCA_RS09170	-	-	-	-	-	-	-	-

Product	*S. carnosus*		*S. equorum*		*S. succinus*			*S. xylosus*		*S. saprophyticus*
	**JCM 6069**	**TM300**	**KS1039**	**Mu2**	**14BME20**	**CSM 77**	**DSM 14617**	**C2a**	**HKUOPL8**	**ATCC 15305**
hypothetical protein	BEK99_RS02070	SCA_RS09110	SE1039_RS09990	SEQMU2_RS01850	-	-	-	-	-	-
AcrB/AcrD/AcrF family protein	BEK99_RS02265	SCA_RS08905	SE1039_RS09800	SEQMU2_RS01660	BK815_RS12695	A6V26_RS06655	AA913_RS00355	SXYL_RS03395	-	SSP_RS03175
hypothetical protein	BEK99_RS02270	SCA_RS08900	-	-	-	-	-	-	-	-
amidohydrolase family protein	BEK99_RS02275	SCA_RS08895	-	-	-	-	-	-	-	-
N-acetyltransferase	BEK99_RS02315	SCA_RS08855	-	-	-	-	-	-	-	-
50S ribosomal protein L30	BEK99_RS02420	SCA_RS08750	SE1039_RS09660	SEQMU2_RS01520	BK815_RS12835	-	-	SXYL_RS03535	BE24_RS08270	SSP_RS03315
50S ribosomal protein L15	BEK99_RS02425	SCA_RS08745	SE1039_RS09655	SEQMU2_RS01515	BK815_RS12840	-	-	SXYL_RS03540	BE24_RS08265	SSP_RS03320
MarR family transcriptional regulator	BEK99_RS02500	SCA_RS08670	-	-	-	-	-	-	-	SSP_RS10655
MFS transporter	BEK99_RS02505	SCA_RS08665	-	-	-	-	-	-	-	SSP_RS10650
cysteine hydrolase	BEK99_RS02510	SCA_RS08660	-	-	BK815_RS09425	A6V26_RS05310	AA913_RS02165	-	-	-
histidine phosphatase family protein	BEK99_RS02515	SCA_RS08655	-	-	-	-	-	-	-	-
PAS domain S-box protein	BEK99_RS02520	SCA_RS08650	-	-	-	-	-	-	-	-
PTS mannitol transporter subunit IIB	BEK99_RS02525	SCA_RS08645	-	-	-	-	-	-	-	-
hydrolase	BEK99_RS02530	SCA_RS08640	SE1039_RS12540	SEQMU2_RS04335	BK815_RS10070	A6V26_RS05910	AA913_RS12475	SXYL_RS00540	BE24_RS11145	-
NADP-dependent oxidoreductase	BEK99_RS02545	SCA_RS08625	-	-	-	-	-	-	-	-
NADP-dependent oxidoreductase	BEK99_RS02555	SCA_RS08615	-	-	-	-	-	-	-	-
MFS transporter	BEK99_RS02590	SCA_RS08580	-	-	BK815_RS12960	A6V26_RS06390	AA913_RS00090	SXYL_RS03660	BE24_RS08145	SSP_RS03440
low temperature requirement protein A	BEK99_RS02630	SCA_RS08540	-	-	BK815_RS08585	A6V26_RS04420	AA913_RS06840	-	-	-
MFS transporter	BEK99_RS02665	SCA_RS08505	-	-	BK815_RS13040	A6V26_RS06310	AA913_RS00010	SXYL_RS12305	BE24_RS12960	SSP_RS11625
chromosome partitioning protein ParA	BEK99_RS02670	SCA_RS08500	SE1039_RS09470	SEQMU2_RS01330	BK815_RS13045	A6V26_RS06305	AA913_RS00005	-	BE24_RS08075	SSP_RS03510
methyltransferase	BEK99_RS02735	SCA_RS08430	-	-	-	-	-	-	-	-
hypothetical protein	BEK99_RS02765	SCA_RS08400	-	-	-	-	-	-	-	-
hypothetical protein	BEK99_RS02775	SCA_RS08390	SE1039_RS09355	SEQMU2_RS01230	BK815_RS00045	A6V26_RS12070	AA913_RS11650	SXYL_RS03840	-	SSP_RS03620
oxidoreductase	BEK99_RS02795	SCA_RS08370	SE1039_RS09335	-	-	-	-	-	-	-
VOC family protein	BEK99_RS02870	SCA_RS08295	SE1039_RS09240	SEQMU2_RS01115	-	-	-	-	-	-
DNA-directed RNA polymerase subunit delta	BEK99_RS02880	SCA_RS08285	SE1039_RS09230	SEQMU2_RS01105	BK815_RS00165	A6V26_RS11945	AA913_RS11525	-	BE24_RS07800	SSP_RS03740
hypothetical protein	BEK99_RS02890	SCA_RS08275	-	-	-	-	-	-	-	-
ATP synthase subunit delta	BEK99_RS02990	SCA_RS08175	SE1039_RS09125	-	BK815_RS00270	A6V26_RS11840	AA913_RS11420	SXYL_RS04060	BE24_RS07695	SSP_RS03845
F0F1 ATP synthase subunit alpha	BEK99_RS02995	-	SE1039_RS09120	SEQMU2_RS00995	BK815_RS00275	A6V26_RS11835	AA913_RS11415	SXYL_RS04065	BE24_RS07690	SSP_RS03850
hypothetical protein	BEK99_RS03025	SCA_RS08140	-	-	-	-	-	-	-	-
transglycosylase	BEK99_RS03050	SCA_RS08115	SE1039_RS11440	SEQMU2_RS03295	BK815_RS11130	A6V26_RS08235	AA913_RS04015	SXYL_RS01585	BE24_RS10160	-

Product	*S. carnosus*		*S. equorum*		*S. succinus*			*S. xylosus*		*S. saprophyticus*
	**JCM 6069**	**TM300**	**KS1039**	**Mu2**	**14BME20**	**CSM 77**	**DSM 14617**	**C2a**	**HKUOPL8**	**ATCC 15305**
RNA polymerase sigma factor SigB	BEK99_RS03190	SCA_RS07975	SE1039_RS08955	SEQMU2_RS00830	BK815_RS00440	A6V26_RS11670	-	SXYL_RS04230	BE24_RS07525	-
RNA-binding transcriptional accessory protein	BEK99_RS03195	SCA_RS07970	SE1039_RS08950	SEQMU2_RS00825	-	A6V26_RS11665	AA913_RS11245	SXYL_RS04235	BE24_RS07520	SSP_RS04025
redox-sensing transcriptional repressor Rex	BEK99_RS03295	SCA_RS07870	SE1039_RS08835	-	BK815_RS00560	A6V26_RS12655	AA913_RS13190	SXYL_RS04350	BE24_RS07385	SSP_RS04145
histidine kinase	BEK99_RS03320	-	SE1039_RS08800	SEQMU2_RS00690	BK815_RS00595	A6V26_RS12620	AA913_RS13225	SXYL_RS04385	BE24_RS07350	SSP_RS04180
delta-hemolysin	BEK99_RS03335	SCA_RS07830	-	-	-	-	-	-	-	-
hypothetical protein	BEK99_RS03345	SCA_RS07820	-	-	-	-	-	-	-	-
threonine aldolase	BEK99_RS03365	SCA_RS07680	-	-	-	-	-	-	-	-
hypothetical protein	BEK99_RS03375	SCA_RS07670	-	-	-	-	-	-	-	-
ABC transporter ATP-binding protein	BEK99_RS03400	SCA_RS07650	SE1039_RS08730	SEQMU2_RS00620	BK815_RS00660	A6V26_RS12555	AA913_RS10155	SXYL_RS04450	BE24_RS07285	-
hypothetical protein	BEK99_RS03405	SCA_RS07645	-	-	-	-	-	-	-	SSP_RS04270
hypothetical protein	BEK99_RS03425	SCA_RS07625	-	SEQMU2_RS00595	-	-	-	SXYL_RS04475	BE24_RS07260	SSP_RS04290
hypothetical protein	BEK99_RS03435	SCA_RS07610	-	-	BK815_RS00695	A6V26_RS12520	AA913_RS10190	-	-	-
hypothetical protein	BEK99_RS03445	SCA_RS07600	-	-	-	-	-	-	-	-
DNA helicase PcrA	BEK99_RS03535	SCA_RS07515	SE1039_RS08600	SEQMU2_RS00475	BK815_RS00810	A6V26_RS12400	AA913_RS11940	SXYL_RS04580	-	SSP_RS04395
hypothetical protein	BEK99_RS03545	SCA_RS07505	SE1039_RS08590	-	BK815_RS00820	A6V26_RS12390	AA913_RS11930	SXYL_RS04590	BE24_RS07145	SSP_RS04405
formate dehydrogenase	BEK99_RS03550	SCA_RS07500	-	-	BK815_RS09930	-	-	-	-	SSP_RS12020
sodium:proline symporter	BEK99_RS03555	SCA_RS07495	-	-	BK815_RS00825	A6V26_RS12385	AA913_RS11925	SXYL_RS04595	BE24_RS07140	SSP_RS04410
general stress protein	BEK99_RS03585	SCA_RS07465	-	-	-	-	-	-	-	-
carbonic anhydrase	BEK99_RS03640	SCA_RS07410	-	-	-	-	-	-	-	-
radical SAM/CxCxxxxC motif protein YfkAB	BEK99_RS03675	SCA_RS07375	-	-	BK815_RS00945	A6V26_RS12265	AA913_RS11805	SXYL_RS04715	BE24_RS07020	SSP_RS04545
recombination regulator RecX	BEK99_RS03690	SCA_RS07360	SE1039_RS08460	SEQMU2_RS00335	BK815_RS00960	A6V26_RS12250	AA913_RS11790	SXYL_RS04735	-	SSP_RS04560
glutamate ABC transporter permease	BEK99_RS03900	SCA_RS07155	SE1039_RS08245	SEQMU2_RS13375	BK815_RS01175	A6V26_RS11500	AA913_RS12075	SXYL_RS04955	BE24_RS06600	-
hypothetical protein	BEK99_RS03995	SCA_RS12520	-	-	-	-	-	-	-	-
quinolinate synthetase	BEK99_RS04040	SCA_RS07015	-	-	BK815_RS10050	A6V26_RS05890	AA913_RS10335	-	-	-
nicotinate-nucleotide diphosphorylase (carboxylating)	BEK99_RS04045	SCA_RS07010	-	-	BK815_RS10045	A6V26_RS05885	AA913_RS10340	-	-	-
cysteine desulfurase	BEK99_RS04055	SCA_RS07000	-	-	BK815_RS10035	A6V26_RS05875	AA913_RS10350	-	-	-
hypothetical protein	BEK99_RS04060	SCA_RS06995	-	-	BK815_RS10030	A6V26_RS05870	AA913_RS10355	-	-	-
SAM-dependent methyltransferase	BEK99_RS04065	SCA_RS06990	-	-	-	-	-	-	-	-
hypothetical protein	BEK99_RS04070	SCA_RS06985	-	-	-	-	-	-	-	-
integrase	BEK99_RS04115	-	SE1039_RS04520	SEQMU2_RS07810	-	A6V26_RS11270	AA913_RS06405	SXYL_RS05180	-	SSP_RS09755
hypothetical protein	BEK99_RS04125	-	-	-	BK815_RS02665	-	-	SXYL_RS08780	-	-

**Product**	***S. carnosus***		***S. equorum***		***S. succinus***			***S. xylosus***		***S. saprophyticus***
	**JCM 6069**	**TM300**	**KS1039**	**Mu2**	**14BME20**	**CSM 77**	**DSM 14617**	**C2a**	**HKUOPL8**	**ATCC 15305**
transcriptional regulator	BEK99_RS04130	-	-	-	-	-	-	SXYL_RS08775	-	-
hypothetical protein	BEK99_RS04140	-	-	-	-	-	AA913_RS06375	-	-	-
hypothetical protein	BEK99_RS04150	SCA_RS02410	-	-	-	-	-	-	-	-
hypothetical protein	BEK99_RS04170	-	-	SEQMU2_RS07885	BK815_RS02705	A6V26_RS11215	AA913_RS06365	-	-	-
single-stranded DNA-binding protein	BEK99_RS04175	-	-	SEQMU2_RS07890	BK815_RS02710	A6V26_RS11210	AA913_RS06360	SXYL_RS08735	-	-
DNA primase	BEK99_RS04195	-	-	SEQMU2_RS11360	-	-	-	SXYL_RS07090	-	-
hypothetical protein	BEK99_RS04205	SCA_RS12455	-	-	-	-	-	-	-	-
hypothetical protein	BEK99_RS04210	SCA_RS02470	-	-	-	-	-	-	-	-
hypothetical protein	BEK99_RS04225	-	SE1039_RS04925	SEQMU2_RS07975	BK815_RS02780	A6V26_RS11130	AA913_RS06280	SXYL_RS08660	-	-
hypothetical protein	BEK99_RS04230	SCA_RS02490	-	-	-	-	-	-	-	-
DUF1381 domain-containing protein	BEK99_RS04235	SCA_RS02495	-	-	-	A6V26_RS11120	AA913_RS06270	-	-	-
hypothetical protein	BEK99_RS04255	-	-	-	-	-	-	SXYL_RS08645	BE24_RS05165	-
hypothetical protein	BEK99_RS04260	-	-	-	BK815_RS02785	-	-	-	BE24_RS05160	-
hypothetical protein	BEK99_RS04265	SCA_RS02525	-	-	-	-	-	-	-	-
hypothetical protein	BEK99_RS04270	SCA_RS02530	-	-	-	-	-	-	-	-
terminase	BEK99_RS04275	SCA_RS02535	-	-	-	-	-	-	-	-
phage portal protein	BEK99_RS04280	SCA_RS02540	-	-	-	-	-	-	-	-
phage major capsid protein	BEK99_RS04290	SCA_RS02550	-	-	-	-	-	-	-	-
hypothetical protein	BEK99_RS04295	SCA_RS02555	-	-	-	-	-	-	-	-
hypothetical protein	BEK99_RS04305	-	-	-	-	-	-	SXYL_RS08590	-	-
hypothetical protein	BEK99_RS04310	-	-	-	-	-	-	SXYL_RS08585	-	-
hypothetical protein	BEK99_RS04320	-	-	-	-	-	-	SXYL_RS08575	-	-
hypothetical protein	BEK99_RS04325	-	-	-	-	-	-	SXYL_RS08570	-	-
phage tail family protein	BEK99_RS04335	SCA_RS02600	-	-	-	-	-	SXYL_RS08550	-	-
peptidase	BEK99_RS04340	SCA_RS02605	-	-	-	-	-	-	-	-
hypothetical protein	BEK99_RS04360	SCA_RS12460	-	-	-	-	-	-	-	-
hypothetical protein	BEK99_RS04365	SCA_RS12465	-	-	-	-	AA913_RS06155	-	-	-
hypothetical protein	BEK99_RS13050	SCA_RS12645	-	-	-	A6V26_RS13505	AA913_RS14300	SXYL_RS13300	-	-
hypothetical protein	BEK99_RS04375	-	-	SEQMU2_RS08105	-	-	-	-	BE24_RS05060	-
N-acetylmuramoyl-L-alanine amidase	BEK99_RS04380	-	-	SEQMU2_RS10905	-	-	-	-	-	-
hypothetical protein	BEK99_RS04410	SCA_RS06940	-	-	-	-	-	-	-	-

Product	*S. carnosus*		*S. equorum*		*S. succinus*			*S. xylosus*		*S. saprophyticus*
	**JCM 6069**	**TM300**	**KS1039**	**Mu2**	**14BME20**	**CSM 77**	**DSM 14617**	**C2a**	**HKUOPL8**	**ATCC 15305**
hypothetical protein	BEK99_RS04415	SCA_RS06935	-	-	-	-	-	-	-	-
hypothetical protein	BEK99_RS04420	SCA_RS06930	-	-	-	-	-	-	-	-
ADP-ribosylglycohydrolase family protein	BEK99_RS04475	SCA_RS06875	-	-	-	-	-	-	-	-
camphor resistance protein CrcB	BEK99_RS04485	SCA_RS06865	SE1039_RS07975	SEQMU2_RS13100	BK815_RS01470	A6V26_RS10920	AA913_RS06055	SXYL_RS05365	-	SSP_RS05045
hypothetical protein	BEK99_RS04490	SCA_RS06860	SE1039_RS07970	SEQMU2_RS13095	BK815_RS01475	A6V26_RS10915	AA913_RS06050	SXYL_RS05370	-	SSP_RS05050
transaldolase	BEK99_RS04495	SCA_RS06855	SE1039_RS07965	SEQMU2_RS13090	BK815_RS01480	A6V26_RS10910	AA913_RS06045	SXYL_RS05375	-	SSP_RS05055
hypothetical protein	BEK99_RS04500	SCA_RS06850	SE1039_RS07955	-	BK815_RS01490	A6V26_RS10900	AA913_RS06035	SXYL_RS05385	-	SSP_RS05065
hypothetical protein	BEK99_RS04510	SCA_RS12515	-	-	-	-	-	-	-	-
pyridine nucleotide-disulfide oxidoreductase	BEK99_RS04525	SCA_RS06830	SE1039_RS07925	SEQMU2_RS13050	-	A6V26_RS10870	AA913_RS06005	SXYL_RS05415	BE24_RS06230	SSP_RS05105
leucine--tRNA ligase	BEK99_RS04580	-	SE1039_RS07865	SEQMU2_RS12990	BK815_RS01580	A6V26_RS10810	AA913_RS05945	SXYL_RS05475	BE24_RS06170	SSP_RS05165
aminoacetone oxidase family FAD-binding enzyme	BEK99_RS04590	SCA_RS06765	SE1039_RS07855	SEQMU2_RS12980	BK815_RS01590	A6V26_RS10800	AA913_RS05935	SXYL_RS05485	-	SSP_RS05175
phosphotransferase	BEK99_RS04620	-	SE1039_RS07825	SEQMU2_RS12950	BK815_RS01620	A6V26_RS10750	AA913_RS05890	SXYL_RS05515	BE24_RS06130	SSP_RS05205
hypothetical protein	BEK99_RS04675	SCA_RS06680	-	-	-	-	-	-	-	-
formate--tetrahydrofolate ligase	BEK99_RS04705	SCA_RS06650	SE1039_RS07740	SEQMU2_RS12865	BK815_RS01705	A6V26_RS10665	AA913_RS05805	SXYL_RS05600	BE24_RS06040	-
free methionine-(R)-sulfoxide reductase	BEK99_RS04765	-	SE1039_RS07675	SEQMU2_RS12790	BK815_RS01770	A6V26_RS10600	AA913_RS05740	SXYL_RS05665	BE24_RS05975	SSP_RS05355
tRNA 4-thiouridine(8) synthase ThiI	BEK99_RS04780	-	SE1039_RS07660	SEQMU2_RS12775	BK815_RS01785	A6V26_RS10585	AA913_RS05725	SXYL_RS05680	BE24_RS05960	SSP_RS05370
translation initiation factor IF-3	BEK99_RS04940	SCA_RS06415	SE1039_RS07480	SEQMU2_RS12595	BK815_RS01965	A6V26_RS10405	AA913_RS05545	SXYL_RS05860	-	SSP_RS05550
folylpolyglutamate synthase	BEK99_RS05030	-	SE1039_RS07390	SEQMU2_RS12505	BK815_RS02055	A6V26_RS10315	AA913_RS05455	SXYL_RS05945	BE24_RS05695	SSP_RS05640
hypothetical protein	BEK99_RS05035	SCA_RS06320	SE1039_RS07380	SEQMU2_RS12495	BK815_RS02060	A6V26_RS10310	AA913_RS05450	-	BE24_RS05685	SSP_RS05650
alanine--tRNA ligase	BEK99_RS05200	SCA_RS06155	SE1039_RS07200	SEQMU2_RS12310	BK815_RS02290	A6V26_RS10090	AA913_RS05235	SXYL_RS06125	-	SSP_RS05825
protease	BEK99_RS05230	-	SE1039_RS07170	SEQMU2_RS12280	BK815_RS02320	A6V26_RS10060	AA913_RS05205	SXYL_RS06155	BE24_RS05485	SSP_RS05855
hypothetical protein	BEK99_RS05290	SCA_RS06065	SE1039_RS07080	SEQMU2_RS12190	BK815_RS02410	A6V26_RS09970	AA913_RS05115	-	BE24_RS05390	SSP_RS05945
tRNA (N(6)-L-threonylcarbamoyladenosine(37)-C(2))-methylthiotransferase MtaB	BEK99_RS05355	SCA_RS06000	-	-	-	-	-	-	-	-
rhomboid family intramembrane serine protease	BEK99_RS05485	SCA_RS05870	SE1039_RS06885	-	BK815_RS02605	A6V26_RS09775	AA913_RS04920	SXYL_RS06445	BE24_RS04905	SSP_RS06140
competence protein ComGA	BEK99_RS05510	SCA_RS05845	SE1039_RS06860	SEQMU2_RS11970	-	A6V26_RS09750	AA913_RS04895	SXYL_RS06470	BE24_RS04880	SSP_RS06165
competence protein ComGC	BEK99_RS05520	SCA_RS05835	SE1039_RS06850	SEQMU2_RS11960	BK815_RS02955	A6V26_RS09740	AA913_RS04885	-	BE24_RS04870	SSP_RS06175
hypothetical protein	BEK99_RS05525	SCA_RS05830	-	SEQMU2_RS11955	BK815_RS02960	A6V26_RS09735	AA913_RS04880	SXYL_RS06485	BE24_RS04865	SSP_RS06180
hypothetical protein	BEK99_RS05530	SCA_RS05825	SE1039_RS06840	SEQMU2_RS11950	BK815_RS02965	A6V26_RS09730	AA913_RS04875	-	-	SSP_RS06185
hypothetical protein	BEK99_RS05535	SCA_RS05820	-	-	-	-	-	-	-	-
glycine dehydrogenase (aminomethyl-transferring)	BEK99_RS05555	-	SE1039_RS06810	SEQMU2_RS11920	BK815_RS02995	A6V26_RS09700	AA913_RS04845	SXYL_RS06520	BE24_RS04830	SSP_RS06215
acetyl-CoA carboxylase biotin carboxyl carrier protein subunit	BEK99_RS05590	SCA_RS05765	SE1039_RS06775	SEQMU2_RS11885	BK815_RS03030	A6V26_RS09665	AA913_RS04810	SXYL_RS06555	-	SSP_RS06250

Product	*S. carnosus*		*S. equorum*		*S. succinus*			*S. xylosus*		*S. saprophyticus*
	**JCM 6069**	**TM300**	**KS1039**	**Mu2**	**14BME20**	**CSM 77**	**DSM 14617**	**C2a**	**HKUOPL8**	**ATCC 15305**
phosphate butyryltransferase	BEK99_RS05635	SCA_RS05720	SE1039_RS06730	SEQMU2_RS11840	BK815_RS03075	A6V26_RS09620	AA913_RS04765	SXYL_RS06600	BE24_RS04750	-
butyrate kinase	BEK99_RS05640	SCA_RS05715	SE1039_RS06725	SEQMU2_RS11835	-	-	-	SXYL_RS06605	BE24_RS04745	-
hypothetical protein	BEK99_RS05675	SCA_RS05680	-	-	-	-	-	-	-	-
hypothetical protein	BEK99_RS05775	SCA_RS05580	SE1039_RS06585	SEQMU2_RS11695	BK815_RS03215	A6V26_RS09480	AA913_RS04625	SXYL_RS06755	-	SSP_RS06435
hypothetical protein	BEK99_RS05785	SCA_RS05570	-	-	-	-	-	-	-	-
hypothetical protein	BEK99_RS05870	SCA_RS05485	SE1039_RS06490	-	BK815_RS03310	A6V26_RS09385	AA913_RS09265	SXYL_RS06850	BE24_RS04510	SSP_RS06530
zinc metallopeptidase	BEK99_RS05875	SCA_RS05480	SE1039_RS06485	SEQMU2_RS11595	BK815_RS03315	A6V26_RS09380	AA913_RS09270	SXYL_RS06855	-	SSP_RS06535
dynamin family protein	BEK99_RS05965	SCA_RS05395	SE1039_RS06395	SEQMU2_RS11505	BK815_RS03410	A6V26_RS09285	AA913_RS09360	SXYL_RS06945	-	SSP_RS06630
DegV family protein	BEK99_RS06005	SCA_RS05355	SE1039_RS06340	SEQMU2_RS11450	BK815_RS03465	A6V26_RS09230	AA913_RS09415	SXYL_RS07000	-	SSP_RS06685
membrane protein	BEK99_RS06240	SCA_RS05120	SE1039_RS06075	SEQMU2_RS11125	-	-	-	SXYL_RS07350	BE24_RS04085	SSP_RS06960
tryptophan synthase subunit alpha	BEK99_RS06255	SCA_RS05105	SE1039_RS06055	SEQMU2_RS11105	BK815_RS03735	A6V26_RS08960	AA913_RS09685	-	-	SSP_RS06980
DNA repair protein	BEK99_RS06300	-	SE1039_RS05960	SEQMU2_RS11050	BK815_RS03980	A6V26_RS00140	AA913_RS03795	SXYL_RS07455	BE24_RS03990	SSP_RS07045
homoserine O-succinyltransferase	BEK99_RS06320	SCA_RS05040	-	-	-	-	-	-	-	-
hypothetical protein	BEK99_RS06495	SCA_RS04860	SE1039_RS05700	-	BK815_RS04195	A6V26_RS00355	AA913_RS03580	SXYL_RS07680	BE24_RS03765	-
hypothetical protein	BEK99_RS06500	SCA_RS04855	-	-	-	-	-	-	-	-
iron ABC transporter substrate-binding protein	BEK99_RS06510	SCA_RS04845	-	-	-	-	-	-	-	-
hypothetical protein	BEK99_RS06530	SCA_RS04825	-	-	-	-	-	-	-	-
tRNA (adenosine(37)-N6)-dimethylallyltransferase MiaA	BEK99_RS06565	SCA_RS04790	SE1039_RS05635	-	BK815_RS04285	A6V26_RS00445	AA913_RS03485	SXYL_RS07750	BE24_RS03670	SSP_RS07390
ABC transporter permease	BEK99_RS06625	SCA_RS04730	-	-	-	-	-	-	-	-
transcriptional regulator	BEK99_RS06655	SCA_RS04700	-	-	-	-	-	-	-	-
LysR family transcriptional regulator	BEK99_RS06680	SCA_RS04675	-	-	BK815_RS10085	A6V26_RS05925	AA913_RS12490	SXYL_RS00395	BE24_RS11315	-
hypothetical protein	BEK99_RS06685	SCA_RS04670	-	-	BK815_RS10080	A6V26_RS05920	AA913_RS12485	SXYL_RS00390	BE24_RS11320	-
MFS transporter	BEK99_RS06775	SCA_RS04580	-	SEQMU2_RS13925	-	-	-	-	-	-
member of glyoxalase/bleomycin resistance protein family	BEK99_RS06785	SCA_RS04570	-	-	-	A6V26_RS08340	AA913_RS04120	SXYL_RS01505	BE24_RS10240	-
exotoxin-like protein	BEK99_RS06790	SCA_RS04565	-	-	-	-	-	-	-	-
phosphonoacetaldehyde hydrolase	BEK99_RS06915	SCA_RS04440	-	-	-	-	-	-	-	-
2-aminoethylphosphonate--pyruvate transaminase	BEK99_RS06920	SCA_RS04435	-	-	-	-	-	-	-	-
iron ABC transporter permease	BEK99_RS06930	SCA_RS04420	-	-	-	-	-	-	-	-
DeoR/GlpR transcriptional regulator	BEK99_RS06940	SCA_RS04410	-	-	-	-	-	-	-	-
alpha/beta hydrolase	BEK99_RS06945	SCA_RS04405	-	-	-	-	-	-	-	-
hypothetical protein	BEK99_RS06950	SCA_RS04400	-	-	-	-	-	-	-	-

Product	*S. carnosus*		*S. equorum*		*S. succinus*			*S. xylosus*		*S. saprophyticus*
	**JCM 6069**	**TM300**	**KS1039**	**Mu2**	**14BME20**	**CSM 77**	**DSM 14617**	**C2a**	**HKUOPL8**	**ATCC 15305**
hypothetical protein	BEK99_RS06960	SCA_RS04390	-	SEQMU2_RS10125	BK815_RS04605	A6V26_RS00765	AA913_RS03165	SXYL_RS08080	-	SSP_RS07725
hypothetical protein	BEK99_RS06965	SCA_RS04385	-	-	-	-	-	-	-	-
hypothetical protein	BEK99_RS06975	SCA_RS04380	-	-	-	-	-	-	-	-
hypothetical protein	BEK99_RS07005	SCA_RS04355	SE1039_RS11390	-	-	-	-	SXYL_RS01645	-	SSP_RS01470
signal recognition particle protein	BEK99_RS07010	-	SE1039_RS05240	SEQMU2_RS10090	BK815_RS04640	A6V26_RS00800	AA913_RS03130	SXYL_RS08115	BE24_RS03305	SSP_RS07760
hypothetical protein	BEK99_RS07095	SCA_RS04270	-	-	-	-	-	-	-	-
hypothetical protein	BEK99_RS07145	SCA_RS04220	-	-	-	-	-	-	-	-
hypothetical protein	BEK99_RS07150	SCA_RS04215	-	-	-	-	-	-	-	-
putative phosphotyrosine-protein phosphatase	BEK99_RS07155	SCA_RS04210	-	-	-	-	-	-	-	-
NADH-flavin reductase	BEK99_RS07160	SCA_RS04205	-	-	BK815_RS05740	A6V26_RS01875	AA913_RS09175	-	-	-
capsular polysaccharide biosynthesis protein Cap8C	BEK99_RS07195	SCA_RS04170	-	SEQMU2_RS05395	-	-	-	-	BE24_RS11820	SSP_RS00315
dihydroorotate dehydrogenase B catalytic subunit	BEK99_RS07220	SCA_RS04145	-	-	-	-	-	-	-	-
dihydroorotate dehydrogenase electron transfer subunit	BEK99_RS07225	SCA_RS04140	-	-	-	-	-	-	-	-
CHAP domain-containing protein	BEK99_RS07270	SCA_RS04095	SE1039_RS04680	SEQMU2_RS09860	BK815_RS04900	A6V26_RS01060	AA913_RS02870	-	-	-
bacillithiol biosynthesis cysteine-adding enzyme BshC	BEK99_RS07360	SCA_RS04005	SE1039_RS04590	SEQMU2_RS09770	-	A6V26_RS01150	AA913_RS02780	SXYL_RS08430	BE24_RS02990	SSP_RS08070
hypothetical protein	BEK99_RS07375	SCA_RS03990	-	-	-	-	-	-	-	-
hypothetical protein	BEK99_RS07380	SCA_RS03985	-	-	-	-	-	-	-	-
phenol soluble modulin	BEK99_RS07390	SCA_RS03975	SE1039_RS04570	SEQMU2_RS09745	-	-	-	SXYL_RS08455	BE24_RS02955	-
hypothetical protein	BEK99_RS07405	SCA_RS12505	-	-	-	-	-	-	-	-
hypothetical protein	BEK99_RS07410	SCA_RS12500	-	-	-	-	-	-	-	-
hypothetical protein	BEK99_RS07415	SCA_RS03955	-	SEQMU2_RS10665	-	A6V26_RS10780	AA913_RS05920	-	-	-
hypothetical protein	BEK99_RS07420	SCA_RS12495	-	SEQMU2_RS08290	-	-	-	SXYL_RS10075	-	-
hypothetical protein	BEK99_RS07425	SCA_RS03940	-	-	-	-	-	-	-	-
hypothetical protein	BEK99_RS07430	SCA_RS03935	-	-	-	-	-	-	-	-
VOC family protein	BEK99_RS07440	SCA_RS03925	-	-	-	-	-	-	-	SSP_RS11645
glyoxalase/bleomycin resistance/dioxygenase family protein	BEK99_RS07445	SCA_RS03920	SE1039_RS00750	SEQMU2_RS06275	BK815_RS05030	-	-	-	BE24_RS01665	SSP_RS11835
hypothetical protein	BEK99_RS07450	SCA_RS03915	-	-	-	-	-	-	-	-
metal-dependent phosphodiesterase	BEK99_RS07455	-	SE1039_RS04510	SEQMU2_RS09720	BK815_RS05060	A6V26_RS01200	AA913_RS02730	SXYL_RS08800	BE24_RS02930	SSP_RS08280
MarR family transcriptional regulator	BEK99_RS07470	SCA_RS03895	-	-	-	-	-	-	-	-
oxidoreductase	BEK99_RS07475	SCA_RS03890	-	-	-	-	-	-	-	-
DNA-binding protein	BEK99_RS07550	-	SE1039_RS04420	SEQMU2_RS09630	BK815_RS05150	A6V26_RS01290	AA913_RS02640	SXYL_RS08890	BE24_RS02840	SSP_RS08370

Product	*S. carnosus*		*S. equorum*		*S. succinus*			*S. xylosus*		*S. saprophyticus*
	**JCM 6069**	**TM300**	**KS1039**	**Mu2**	**14BME20**	**CSM 77**	**DSM 14617**	**C2a**	**HKUOPL8**	**ATCC 15305**
ornithine cyclodeaminase	BEK99_RS07560	SCA_RS03805	-	-	-	-	-	-	-	-
DUF2197 domain-containing protein	BEK99_RS07635	SCA_RS03730	SE1039_RS13560	-	BK815_RS05235	A6V26_RS13430	AA913_RS14240	SXYL_RS13310	BE24_RS13595	SSP_RS12825
divalent metal cation transporter	BEK99_RS07660	SCA_RS03705	-	SEQMU2_RS14135	BK815_RS05265	A6V26_RS01400	AA913_RS02530	SXYL_RS09000	BE24_RS02730	SSP_RS08480
hypothetical protein	BEK99_RS07665	SCA_RS03700	-	-	-	-	-	-	-	-
XRE family transcriptional regulator	BEK99_RS07695	SCA_RS03670	-	-	BK815_RS05295	A6V26_RS01430	AA913_RS02500	SXYL_RS09030	BE24_RS02700	SSP_RS08510
hypothetical protein	BEK99_RS07735	SCA_RS03630	-	-	-	-	-	-	-	-
transcriptional regulator	BEK99_RS07750	SCA_RS03615	-	SEQMU2_RS14210	BK815_RS09455	A6V26_RS13370	AA913_RS13130	-	-	SSP_RS08170
3-hexulose-6-phosphate synthase	BEK99_RS07755	SCA_RS03610	SE1039_RS02035	SEQMU2_RS14205	BK815_RS07465	-	AA913_RS12920	-	BE24_RS00565	-
6-phospho-3-hexuloisomerase	BEK99_RS07760	SCA_RS03605	-	-	-	-	-	-	-	-
hypothetical protein	BEK99_RS07825	SCA_RS03540	-	-	-	-	-	-	-	-
DeoR/GlpR transcriptional regulator	BEK99_RS07930	SCA_RS03435	-	-	-	-	-	-	-	-
galactose-6-phosphate isomerase LacA subunit	BEK99_RS07935	SCA_RS03430	-	-	-	-	-	-	-	-
galactose-6-phosphate isomerase subunit LacB	BEK99_RS07940	SCA_RS03425	-	-	-	-	-	-	-	-
arsenical efflux pump membrane protein ArsB	BEK99_RS07955	SCA_RS03410	-	-	-	-	-	-	-	-
arsenate reductase	BEK99_RS07960	SCA_RS03405	-	-	-	-	-	-	-	-
6-phospho-beta-galactosidase	BEK99_RS07965	SCA_RS03400	-	-	-	-	-	-	-	-
PTS lactose transporter subunit IIB	BEK99_RS07970	SCA_RS03395	-	-	-	-	-	-	-	-
PTS lactose/cellobiose transporter subunit IIA	BEK99_RS07975	SCA_RS03390	SE1039_RS11745	SEQMU2_RS03600	-	-	-	SXYL_RS01270	-	SSP_RS01175
peptidase	BEK99_RS07980	SCA_RS03385	SE1039_RS08040	SEQMU2_RS13165	BK815_RS04225	A6V26_RS00385	AA913_RS03550	SXYL_RS05310	BE24_RS06305	-
peptidoglycan hydrolase	BEK99_RS08010	SCA_RS03355	-	SEQMU2_RS09265	BK815_RS05525	A6V26_RS01660	AA913_RS02270	SXYL_RS09265	BE24_RS02465	SSP_RS08750
MarR family transcriptional regulator	BEK99_RS08030	-	SE1039_RS04035	SEQMU2_RS09245	BK815_RS05545	A6V26_RS01680	AA913_RS02250	SXYL_RS09285	BE24_RS02445	SSP_RS08770
hypothetical protein	BEK99_RS08070	SCA_RS03295	-	-	-	-	-	-	-	-
competence protein ComK	BEK99_RS08095	SCA_RS03270	SE1039_RS03920	SEQMU2_RS09150	BK815_RS05600	A6V26_RS01735	AA913_RS13080	-	BE24_RS02390	SSP_RS08825
hypothetical protein	BEK99_RS08110	SCA_RS12490	-	-	-	-	-	-	-	-
hypothetical protein	BEK99_RS08115	SCA_RS12485	-	-	-	-	-	-	-	-
hypothetical protein	BEK99_RS08120	SCA_RS03250	-	-	-	-	-	-	-	-
alpha/beta hydrolase	BEK99_RS08125	SCA_RS03240	-	-	-	-	-	-	-	-
transcriptional regulator	BEK99_RS08180	SCA_RS03185	SE1039_RS00755	SEQMU2_RS06280	-	-	-	SXYL_RS12495	BE24_RS12700	SSP_RS11825
hypothetical protein	BEK99_RS08205	SCA_RS03160	-	-	-	-	-	-	-	SSP_RS08875
alanine:cation symporter family protein	BEK99_RS08235	-	SE1039_RS03840	SEQMU2_RS09070	BK815_RS05680	A6V26_RS01815	AA913_RS12675	SXYL_RS09420	BE24_RS02310	SSP_RS08905
MFS transporter	BEK99_RS08245	SCA_RS03120	-	-	-	-	-	-	-	-

Product	*S. carnosus*		*S. equorum*		*S. succinus*			*S. xylosus*		*S. saprophyticus*
	**JCM 6069**	**TM300**	**KS1039**	**Mu2**	**14BME20**	**CSM 77**	**DSM 14617**	**C2a**	**HKUOPL8**	**ATCC 15305**
hypothetical protein	BEK99_RS13070	SCA_RS12480	-	-	-	-	-	-	-	-
hypothetical protein	BEK99_RS13075	SCA_RS12475	-	-	-	-	-	-	-	-
hypothetical protein	BEK99_RS08305	SCA_RS03060	-	-	-	-	-	-	-	-
phosphomethylpyrimidine synthase ThiC	BEK99_RS08330	-	-	-	BK815_RS11430	A6V26_RS07930	AA913_RS01635	SXYL_RS12675	BE24_RS12510	-
peptide ABC transporter substrate-binding protein	BEK99_RS08340	SCA_RS03025	SE1039_RS03750	SEQMU2_RS08980	BK815_RS05770	A6V26_RS01905	AA913_RS09145	SXYL_RS09505	-	SSP_RS08995
membrane protein	BEK99_RS08365	SCA_RS03000	SE1039_RS03725	SEQMU2_RS08955	BK815_RS05795	A6V26_RS01930	AA913_RS09120	SXYL_RS09530	-	SSP_RS09020
MFS transporter	BEK99_RS08370	SCA_RS02995	SE1039_RS03720	SEQMU2_RS08950	BK815_RS05800	-	-	SXYL_RS09535	BE24_RS02200	SSP_RS09025
acetyltransferase	BEK99_RS08395	SCA_RS02970	-	SEQMU2_RS08920	BK815_RS05825	A6V26_RS01955	AA913_RS09095	SXYL_RS09560	BE24_RS02175	SSP_RS09050
helicase-exonuclease AddAB subunit AddA	BEK99_RS08425	SCA_RS02940	SE1039_RS03660	SEQMU2_RS08885	BK815_RS05870	A6V26_RS02000	AA913_RS09050	SXYL_RS09595	-	SSP_RS09085
amidohydrolase	BEK99_RS08465	SCA_RS02900	-	-	-	-	-	-	-	-
hypothetical protein	BEK99_RS08495	SCA_RS02870	-	-	-	-	-	-	-	-
hypothetical protein	BEK99_RS08500	SCA_RS02865	-	-	-	-	-	-	-	-
acetyltransferase	BEK99_RS08590	SCA_RS02775	-	-	-	-	-	-	BE24_RS06325	-
MerR family transcriptional regulator	BEK99_RS08605	SCA_RS02760	-	-	-	-	-	-	-	SSP_RS12230
copper-translocating P-type ATPase	BEK99_RS08610	SCA_RS02755	SE1039_RS00600	SEQMU2_RS06105	-	A6V26_RS12890	AA913_RS04440	-	-	-
hemolysin	BEK99_RS08690	-	SE1039_RS03405	SEQMU2_RS08630	BK815_RS06105	A6V26_RS02250	AA913_RS08670	SXYL_RS09830	BE24_RS01900	SSP_RS09320
hypothetical protein	BEK99_RS08695	SCA_RS02670	-	-	-	-	-	-	-	-
hypothetical protein	BEK99_RS08710	-	SE1039_RS05050	SEQMU2_RS08110	BK815_RS02915	-	-	-	-	-
hypothetical protein	BEK99_RS08715	-	SE1039_RS05045	-	BK815_RS02910	-	-	SXYL_RS05240	-	-
hypothetical protein	BEK99_RS08720	-	SE1039_RS05040	SEQMU2_RS08100	BK815_RS02905	-	-	-	-	-
hypothetical protein	BEK99_RS08740	-	-	-	-	-	-	SXYL_RS08540	-	-
peptidase	BEK99_RS08745	-	-	-	-	A6V26_RS11030	AA913_RS06175	SXYL_RS08545	-	-
phage tail family protein	BEK99_RS08750	-	-	-	-	A6V26_RS11035	AA913_RS06180	-	-	-
hypothetical protein	BEK99_RS08760	SCA_RS02585	-	-	-	-	-	-	-	-
phage tail protein	BEK99_RS08765	SCA_RS02580	-	-	-	-	-	-	-	-
phage tail protein	BEK99_RS08770	SCA_RS02575	-	-	-	-	-	-	-	-
hypothetical protein	BEK99_RS08775	SCA_RS02570	-	-	-	-	-	-	-	-
hypothetical protein	BEK99_RS08780	SCA_RS02565	-	-	-	-	-	-	-	-
hypothetical protein	BEK99_RS08785	SCA_RS02560	-	-	-	-	-	-	-	-
hypothetical protein	BEK99_RS08825	-	-	-	-	A6V26_RS11110	AA913_RS06260	-	-	-
hypothetical protein	BEK99_RS08880	SCA_RS02460	-	-	-	-	-	-	-	-

Product	*S. carnosus*		*S. equorum*		*S. succinus*			*S. xylosus*		*S. saprophyticus*
	**JCM 6069**	**TM300**	**KS1039**	**Mu2**	**14BME20**	**CSM 77**	**DSM 14617**	**C2a**	**HKUOPL8**	**ATCC 15305**
RusA family crossover junction endodeoxyribonuclease	BEK99_RS08885	SCA_RS02455	SE1039_RS04880	-	BK815_RS02740	-	-	SXYL_RS08705	BE24_RS05220	-
hypothetical protein	BEK99_RS08890	SCA_RS02450	-	-	-	-	-	-	-	-
DNA replication protein DnaC	BEK99_RS08895	SCA_RS02445	SE1039_RS04870	SEQMU2_RS10740	BK815_RS02730	A6V26_RS11190	AA913_RS06340	SXYL_RS08720	BE24_RS05235	-
replication protein	BEK99_RS08900	-	SE1039_RS04865	-	-	-	-	SXYL_RS08725	BE24_RS05240	-
hypothetical protein	BEK99_RS08910	SCA_RS02435	SE1039_RS04855	-	BK815_RS02715	A6V26_RS11205	AA913_RS06355	SXYL_RS08730	BE24_RS05255	-
single-stranded DNA-binding protein	BEK99_RS08915	-	SE1039_RS04850	-	-	-	-	-	-	-
chromosomal replication initiator DnaA	BEK99_RS08925	-	SE1039_RS04840	-	-	-	-	-	BE24_RS05265	-
hypothetical protein	BEK99_RS08930	SCA_RS02415	-	-	BK815_RS02700	-	AA913_RS06370	SXYL_RS08745	BE24_RS05270	-
hypothetical protein	BEK99_RS08940	SCA_RS02405	-	-	-	-	-	-	-	-
hypothetical protein	BEK99_RS08945	SCA_RS02400	-	-	-	-	-	-	-	-
antirepressor	BEK99_RS08960	SCA_RS02380	-	-	-	-	-	-	BE24_RS05295	-
transcriptional regulator	BEK99_RS08965	SCA_RS02375	-	-	-	-	-	-	-	-
transcriptional regulator	BEK99_RS08970	SCA_RS02370	-	-	-	-	-	-	-	-
toxin	BEK99_RS08975	SCA_RS02365	-	SEQMU2_RS07820	-	-	AA913_RS06395	-	-	-
hypothetical protein	BEK99_RS08990	-	-	-	BK815_RS03940	A6V26_RS00075	AA913_RS03860	-	-	-
site-specific integrase	BEK99_RS09000	SCA_RS02355	-	-	-	-	-	-	BE24_RS05315	-
acetyltransferase	BEK99_RS09080	SCA_RS02275	-	-	BK815_RS06215	A6V26_RS02360	AA913_RS08560	-	-	-
hypothetical protein	BEK99_RS09085	SCA_RS02270	-	-	-	-	-	-	-	-
cupin	BEK99_RS09095	SCA_RS02260	-	-	-	-	-	-	-	-
hypothetical protein	BEK99_RS09100	SCA_RS02255	-	-	-	-	-	-	-	-
LemA family protein	BEK99_RS09105	SCA_RS02250	-	-	-	-	-	-	-	-
potassium transporter KtrB	BEK99_RS09110	SCA_RS02245	-	-	BK815_RS06145	A6V26_RS02290	AA913_RS08630	-	BE24_RS12685	SSP_RS11845
hypothetical protein	BEK99_RS09120	SCA_RS02235	-	-	-	-	-	-	-	-
hypothetical protein	BEK99_RS09125	SCA_RS02230	SE1039_RS03215	SEQMU2_RS08425	-	-	-	-	-	-
cold-shock protein	BEK99_RS09130	SCA_RS02225	-	-	-	-	-	-	-	-
hypothetical protein	BEK99_RS09135	SCA_RS02220	-	-	-	-	-	-	-	-
hypothetical protein	BEK99_RS09140	SCA_RS02215	-	-	-	-	-	-	-	-
hypothetical protein	BEK99_RS09145	SCA_RS02210	-	-	-	-	-	-	-	-
hypothetical protein	BEK99_RS13085	SCA_RS12635	-	-	-	-	-	-	-	-
hypothetical protein	BEK99_RS09210	SCA_RS02150	SE1039_RS03045	-	BK815_RS06440	A6V26_RS02565	AA913_RS08365	SXYL_RS10140	BE24_RS01590	SSP_RS09605
hypothetical protein	BEK99_RS09215	SCA_RS02145	-	-	-	-	-	-	-	-

Product	*S. carnosus*		*S. equorum*		*S. succinus*			*S. xylosus*		*S. saprophyticus*
	**JCM 6069**	**TM300**	**KS1039**	**Mu2**	**14BME20**	**CSM 77**	**DSM 14617**	**C2a**	**HKUOPL8**	**ATCC 15305**
TIGR01777 family protein	BEK99_RS09220	SCA_RS02140	SE1039_RS03030	SEQMU2_RS08140	BK815_RS06455	-	-	SXYL_RS10160	BE24_RS01575	SSP_RS09620
MFS transporter	BEK99_RS09240	SCA_RS02120	-	-	-	-	-	-	BE24_RS11480	-
LysM family protein	BEK99_RS09305	SCA_RS02055	-	SEQMU2_RS07740	BK815_RS06545	A6V26_RS02670	AA913_RS08260	SXYL_RS10240	BE24_RS01495	SSP_RS09830
peptide chain release factor 2	BEK99_RS09310	SCA_RS02050	SE1039_RS02945	SEQMU2_RS07735	BK815_RS06550	A6V26_RS02675	AA913_RS08255	SXYL_RS10245	BE24_RS01490	-
hypothetical protein	BEK99_RS09325	SCA_RS02035	SE1039_RS02930	-	BK815_RS06565	A6V26_RS02690	AA913_RS08240	SXYL_RS10260	BE24_RS01475	SSP_RS09850
hypothetical protein	BEK99_RS09385	SCA_RS01975	-	-	BK815_RS06625	A6V26_RS02750	AA913_RS08180	SXYL_RS10320	BE24_RS01415	SSP_RS09910
hypothetical protein	BEK99_RS09400	SCA_RS01960	-	-	-	-	-	-	-	-
5(3)-deoxyribonucleotidase	BEK99_RS09460	-	SE1039_RS02790	SEQMU2_RS07580	BK815_RS06705	A6V26_RS02830	AA913_RS08100	SXYL_RS10400	BE24_RS01335	SSP_RS09990
iron-sulfur cluster repair di-iron protein	BEK99_RS09550	SCA_RS01810	-	-	-	-	-	-	-	-
two-component sensor histidine kinase	BEK99_RS09555	SCA_RS01805	-	-	-	-	-	SXYL_RS00560	BE24_RS11125	-
DNA-binding response regulator	BEK99_RS09560	-	-	-	-	-	-	SXYL_RS00555	BE24_RS11130	-
DoxX family protein	BEK99_RS09565	SCA_RS01795	-	-	-	-	-	SXYL_RS00550	BE24_RS11135	-
hypothetical protein	BEK99_RS09570	SCA_RS01790	-	-	-	-	-	SXYL_RS00545	BE24_RS11140	-
hypothetical protein	BEK99_RS09600	SCA_RS01760	-	-	-	-	-	-	-	-
hypothetical protein	BEK99_RS09625	SCA_RS01735	SE1039_RS13545	-	BK815_RS06865	A6V26_RS02990	AA913_RS14320	SXYL_RS13325	BE24_RS01180	SSP_RS12845
hypothetical protein	BEK99_RS09630	SCA_RS01730	SE1039_RS12545	SEQMU2_RS04340	-	-	-	-	-	-
hypothetical protein	BEK99_RS09705	SCA_RS12625	-	-	-	-	-	-	-	-
hypothetical protein	BEK99_RS09750	SCA_RS01610	-	-	-	-	-	-	-	-
AraC family transcriptional regulator	BEK99_RS09755	SCA_RS01600	-	-	-	-	-	-	-	-
HxlR family transcriptional regulator	BEK99_RS09770	SCA_RS01580	SE1039_RS00570	SEQMU2_RS06075	BK815_RS08640	A6V26_RS04475	AA913_RS06895	SXYL_RS12815	BE24_RS12410	-
nucleoside permease	BEK99_RS09855	SCA_RS01495	SE1039_RS02405	SEQMU2_RS07190	-	A6V26_RS03225	AA913_RS07710	SXYL_RS10790	BE24_RS00940	SSP_RS10385
glycerol-3-phosphate cytidylyltransferase	BEK99_RS09870	SCA_RS01480	SE1039_RS02390	-	BK815_RS07115	A6V26_RS03240	AA913_RS07695	SXYL_RS10805	BE24_RS00925	SSP_RS10400
hypothetical protein	BEK99_RS09895	SCA_RS01455	-	-	BK815_RS09925	-	-	-	-	-
hypothetical protein	BEK99_RS09900	SCA_RS01450	-	-	-	-	-	-	-	-
threonine--tRNA ligase	BEK99_RS09910	SCA_RS01440	-	SEQMU2_RS14125	BK815_RS01265	A6V26_RS11410	AA913_RS12365	-	-	-
N-acetyltransferase	BEK99_RS09930	SCA_RS01420	-	-	-	-	-	-	-	-
phosphatase PAP2 family protein	BEK99_RS09935	SCA_RS01415	-	-	-	-	-	-	-	-
hypothetical protein	BEK99_RS09980	SCA_RS01365	-	-	-	-	-	-	-	-
Txe/YoeB family addiction module toxin	BEK99_RS10035	SCA_RS01310	-	-	-	-	-	-	-	-
prevent-host-death protein	BEK99_RS10040	SCA_RS01305	-	-	-	-	-	-	-	-
LysE type translocator protein	BEK99_RS10055	SCA_RS01290	-	-	-	-	-	-	-	-

Product	*S. carnosus*		*S. equorum*		*S. succinus*			*S. xylosus*		*S. saprophyticus*
	**JCM 6069**	**TM300**	**KS1039**	**Mu2**	**14BME20**	**CSM 77**	**DSM 14617**	**C2a**	**HKUOPL8**	**ATCC 15305**
hypothetical protein	BEK99_RS10080	SCA_RS01265	-	-	-	-	-	-	-	-
hypothetical protein	BEK99_RS10130	SCA_RS01215	-	-	-	-	-	-	-	-
DedA family protein	BEK99_RS10135	SCA_RS01210	-	-	-	-	-	-	-	-
hypothetical protein	BEK99_RS10150	SCA_RS01195	SE1039_RS02095	SEQMU2_RS06880	-	A6V26_RS03540	AA913_RS07395	SXYL_RS11095	BE24_RS00665	SSP_RS10670
glucosamine-6-phosphate deaminase	BEK99_RS10185	SCA_RS01160	-	-	BK815_RS07470	A6V26_RS03595	AA913_RS11175	SXYL_RS11210	BE24_RS00560	SSP_RS10745
3-hexulose-6-phosphate synthase	BEK99_RS10190	SCA_RS01155	-	-	-	A6V26_RS13355	-	SXYL_RS11205	-	SSP_RS10740
hydrolase	BEK99_RS10225	SCA_RS01120	-	-	-	-	-	-	-	-
DUF454 domain-containing protein	BEK99_RS10240	SCA_RS01105	SE1039_RS03615	SEQMU2_RS08840	-	-	-	SXYL_RS11185	BE24_RS00585	-
LD-carboxypeptidase	BEK99_RS10250	SCA_RS01095	SE1039_RS05595	SEQMU2_RS10445	BK815_RS04310	A6V26_RS00470	AA913_RS03460	-	BE24_RS03645	SSP_RS07420
N-acetyltransferase	BEK99_RS10260	SCA_RS01085	-	-	-	-	-	-	-	-
choloylglycine hydrolase	BEK99_RS10270	SCA_RS01075	SE1039_RS12265	SEQMU2_RS04125	BK815_RS08590	A6V26_RS04425	AA913_RS06845	-	BE24_RS10950	SSP_RS00715
amidohydrolase	BEK99_RS10280	-	SE1039_RS01615	SEQMU2_RS00160	BK815_RS07695	A6V26_RS03815	AA913_RS10955	SXYL_RS11575	BE24_RS00160	SSP_RS11025
hypothetical protein	BEK99_RS10310	SCA_RS01035	-	-	-	-	-	-	-	-
50S ribosomal protein L33	BEK99_RS13100	SCA_RS12620	SE1039_RS13540	-	BK815_RS07770	A6V26_RS13440	AA913_RS14370	SXYL_RS11650	BE24_RS13560	SSP_RS11100
RNA polymerase sigma factor	BEK99_RS10360	SCA_RS00985	-	-	-	-	-	-	-	-
N-acetyltransferase	BEK99_RS10435	SCA_RS00910	-	-	-	-	-	SXYL_RS00150	-	-
N-acetyltransferase	BEK99_RS10455	SCA_RS00890	-	-	-	-	-	-	-	-
tRNA dihydrouridine synthase DusB	BEK99_RS10550	SCA_RS00815	-	-	-	-	-	-	-	-
dihydropteroate synthase	BEK99_RS10565	SCA_RS00800	SE1039_RS01345	SEQMU2_RS13615	BK815_RS07960	A6V26_RS13180	AA913_RS12325	SXYL_RS11820	-	SSP_RS11285
hypothetical protein	BEK99_RS10690	SCA_RS00670	-	-	-	-	-	-	-	-
putative ABC transporter ATP-binding protein	BEK99_RS10695	SCA_RS00665	-	-	-	-	-	-	-	-
hypothetical protein	BEK99_RS10700	SCA_RS00660	-	-	-	-	-	-	-	-
hypothetical protein	BEK99_RS10705	SCA_RS00655	-	-	-	-	-	-	-	-
DUF5085 domain-containing protein	BEK99_RS10710	SCA_RS00650	-	-	-	-	-	-	-	-
hypothetical protein	BEK99_RS10720	SCA_RS00645	-	-	-	-	-	-	-	-
hypothetical protein	BEK99_RS10725	SCA_RS12440	-	-	BK815_RS08240	-	-	-	BE24_RS13155	SSP_RS02445
N-acetyltransferase	BEK99_RS10805	SCA_RS00560	SE1039_RS01125	SEQMU2_RS06710	-	-	-	SXYL_RS12035	BE24_RS13240	SSP_RS11485
hypothetical protein	BEK99_RS10810	SCA_RS00555	-	-	-	-	-	-	-	-
MFS transporter	BEK99_RS10815	SCA_RS00550	-	-	BK815_RS02085	A6V26_RS10285	AA913_RS05425	-	-	SSP_RS10835
LysR family transcriptional regulator	BEK99_RS10820	SCA_RS00545	-	-	-	-	-	-	-	SSP_RS10830
hypothetical protein	BEK99_RS10830	SCA_RS00540	-	-	-	-	-	-	-	-

Product	*S. carnosus*		*S. equorum*		*S. succinus*			*S. xylosus*		*S. saprophyticus*
	**JCM 6069**	**TM300**	**KS1039**	**Mu2**	**14BME20**	**CSM 77**	**DSM 14617**	**C2a**	**HKUOPL8**	**ATCC 15305**
transcriptional regulator	BEK99_RS10835	SCA_RS00535	-	-	-	-	-	-	-	-
hypothetical protein	BEK99_RS10895	SCA_RS00475	-	-	-	-	-	-	-	-
patatin	BEK99_RS10900	SCA_RS00470	-	-	-	-	-	-	-	-
HesB/YadR/YfhF family protein	BEK99_RS10905	SCA_RS00465	-	-	-	-	-	-	-	-
DNA mismatch repair protein MutT	BEK99_RS10910	SCA_RS00460	-	-	-	-	-	-	-	-
hypothetical protein	BEK99_RS10915	SCA_RS00455	-	-	-	-	-	-	-	-
bifunctional metallophosphatase/5-nucleotidase	BEK99_RS10925	SCA_RS00445	-	-	-	-	-	-	-	-
N-acetyltransferase	BEK99_RS10930	SCA_RS00440	SE1039_RS01055	SEQMU2_RS06640	-	-	-	SXYL_RS12105	BE24_RS13170	SSP_RS11555
cystathionine gamma-synthase	BEK99_RS10960	SCA_RS00410	SE1039_RS11610	SEQMU2_RS03465	BK815_RS10925	-	-	SXYL_RS01395	BE24_RS10350	SSP_RS01235
TetR/AcrR family transcriptional regulator	BEK99_RS10975	SCA_RS00395	-	-	-	-	-	-	BE24_RS11205	SSP_RS00575
type 1 glutamine amidotransferase domain-containing protein	BEK99_RS10990	SCA_RS00380	-	-	-	-	-	-	-	-
hypothetical protein	BEK99_RS10995	SCA_RS00375	-	-	-	-	-	-	-	-
hypothetical protein	BEK99_RS11000	SCA_RS00370	-	-	-	-	-	-	-	-
hypothetical protein	BEK99_RS11005	SCA_RS12435	-	-	-	-	-	-	-	-
hypothetical protein	BEK99_RS11010	SCA_RS00360	-	-	-	-	-	-	-	-
hypothetical protein	BEK99_RS11030	SCA_RS00340	-	-	-	-	-	-	-	-
hypothetical protein	BEK99_RS11035	SCA_RS00335	-	-	-	-	-	SXYL_RS02940	-	SSP_RS02710
PadR family transcriptional regulator	BEK99_RS11040	SCA_RS00330	-	-	-	-	-	SXYL_RS02935	-	-
hypothetical protein	BEK99_RS11050	SCA_RS00320	SE1039_RS04525	-	-	-	-	-	-	-
acetoin reductase	BEK99_RS11065	SCA_RS00310	-	-	-	-	-	SXYL_RS05280	-	-
hypothetical protein	BEK99_RS11105	SCA_RS00270	-	-	-	-	-	-	-	-
hypothetical protein	BEK99_RS11115	SCA_RS00260	-	-	-	-	-	-	-	-
NUDIX hydrolase	BEK99_RS11120	SCA_RS00255	-	-	-	-	-	-	-	-
hypothetical protein	BEK99_RS11125	SCA_RS00250	-	-	-	-	-	-	-	-
2-keto-4-pentenoate hydratase	BEK99_RS11180	SCA_RS00195	SE1039_RS00840	SEQMU2_RS06365	-	-	-	-	-	-
hypothetical protein	BEK99_RS11195	SCA_RS00180	-	-	-	-	-	-	-	-
phosphatase	BEK99_RS11200	SCA_RS00175	-	-	-	-	-	-	-	-
carboxymuconolactone decarboxylase family protein	BEK99_RS11210	SCA_RS00165	SE1039_RS08045	SEQMU2_RS13170	-	-	-	-	-	-
hypothetical protein	BEK99_RS11225	SCA_RS00150	-	-	-	-	-	-	-	-
monooxygenase IsdI	BEK99_RS11230	SCA_RS00145	-	-	-	-	-	-	-	-
hypothetical protein	BEK99_RS11235	SCA_RS00140	-	-	-	-	-	-	-	-

Product	*S. carnosus*		*S. equorum*		*S. succinus*			*S. xylosus*		*S. saprophyticus*
	**JCM 6069**	**TM300**	**KS1039**	**Mu2**	**14BME20**	**CSM 77**	**DSM 14617**	**C2a**	**HKUOPL8**	**ATCC 15305**
thermonuclease	BEK99_RS11255	SCA_RS00120	-	-	-	-	-	-	-	-
hypothetical protein	BEK99_RS11260	SCA_RS00115	-	-	-	-	-	-	-	-
MazF/PemK family toxin	BEK99_RS11265	SCA_RS00110	-	-	-	-	-	-	-	-
oxidoreductase	BEK99_RS11275	SCA_RS00100	-	-	-	-	-	-	-	-
hypothetical protein	BEK99_RS11280	SCA_RS00095	-	-	-	-	-	-	-	-
5-amino-6-(5-phosphoribosylamino)uracil reductase	BEK99_RS11290	SCA_RS00090	-	-	-	-	-	-	-	-
hypothetical protein	BEK99_RS11300	SCA_RS00080	SE1039_RS00670	-	BK815_RS08560	A6V26_RS04395	AA913_RS06815	SXYL_RS12600	BE24_RS13675	SSP_RS11940
hypothetical protein	BEK99_RS11340	SCA_RS00035	-	-	-	-	-	-	-	-
hypothetical protein	BEK99_RS11415	SCA_RS12395	-	-	-	-	-	-	-	-
cadmium transporter	BEK99_RS11505	-	-	SEQMU2_RS14025	-	A6V26_RS12995	AA913_RS04340	-	BE24_RS01680	-
transcriptional regulator	BEK99_RS11510	-	-	SEQMU2_RS14030	-	A6V26_RS12990	AA913_RS04345	-	BE24_RS01685	-
arsenate reductase	BEK99_RS11515	-	SE1039_RS03990	SEQMU2_RS05175	BK815_RS00715	A6V26_RS12495	AA913_RS10220	-	-	SSP_RS08230
transcriptional regulator	BEK99_RS11525	-	SE1039_RS03980	SEQMU2_RS05185	BK815_RS00725	A6V26_RS12790	AA913_RS04540	-	-	SSP_RS11980
hypothetical protein	BEK99_RS11530	-	-	-	-	A6V26_RS12770	AA913_RS04300	-	-	-
transcriptional regulator	BEK99_RS11535	-	SE1039_RS03960	SEQMU2_RS09190	-	A6V26_RS12765	AA913_RS04305	-	-	-
permease	BEK99_RS11540	-	SE1039_RS03955	SEQMU2_RS09185	-	A6V26_RS12760	AA913_RS04310	-	-	-
hypothetical protein	BEK99_RS11560	-	SE1039_RS13345	SEQMU2_RS04925	BK815_RS09695	-	-	-	BE24_RS11740	SSP_RS00170
hypothetical protein	BEK99_RS11565	-	SE1039_RS13340	SEQMU2_RS04930	BK815_RS09700	-	-	-	BE24_RS11735	SSP_RS00175
hypothetical protein	BEK99_RS11570	-	SE1039_RS13335	-	-	-	-	-	-	SSP_RS00180
recombinase RecB	BEK99_RS11575	-	SE1039_RS13330	SEQMU2_RS05295	BK815_RS09710	-	-	-	BE24_RS11725	SSP_RS00185
recombinase family protein	BEK99_RS11580	-	SE1039_RS13325	SEQMU2_RS05290	BK815_RS09715	-	-	-	BE24_RS11720	SSP_RS00190
hypothetical protein	BEK99_RS11585	-	SE1039_RS13320	SEQMU2_RS05285	BK815_RS09720	-	-	-	-	SSP_RS00195
hypothetical protein	BEK99_RS11590	-	SE1039_RS13315	SEQMU2_RS05280	BK815_RS09725	-	-	-	BE24_RS11710	SSP_RS00200
hypothetical protein	BEK99_RS11595	-	-	SEQMU2_RS05235	-	-	-	-	-	-
hypothetical protein	BEK99_RS11600	-	-	SEQMU2_RS05230	-	-	-	-	-	-
IS6 family transposase	BEK99_RS11640	-	-	SEQMU2_RS13865	-	A6V26_RS13340	AA913_RS12905	-	-	SSP_RS08265
hypothetical protein	BEK99_RS11655	-	SE1039_RS13220	SEQMU2_RS05160	-	-	-	-	BE24_RS11895	SSP_RS00225
hypothetical protein	BEK99_RS11660	-	SE1039_RS13215	SEQMU2_RS05155	-	-	-	-	BE24_RS11890	SSP_RS00230
DNA primase	BEK99_RS11665	-	SE1039_RS13210	SEQMU2_RS05150	-	-	-	-	BE24_RS11885	SSP_RS00235
recombinase RecB	BEK99_RS11670	-	SE1039_RS13205	SEQMU2_RS05020	-	-	-	-	BE24_RS11880	SSP_RS00240
hypothetical protein	BEK99_RS11675	-	-	SEQMU2_RS05300	BK815_RS09705	-	-	-	BE24_RS11730	SSP_RS00245

Product	*S. carnosus*		*S. equorum*		*S. succinus*			*S. xylosus*		*S. saprophyticus*
	**JCM 6069**	**TM300**	**KS1039**	**Mu2**	**14BME20**	**CSM 77**	**DSM 14617**	**C2a**	**HKUOPL8**	**ATCC 15305**
hypothetical protein	BEK99_RS11680	-	SE1039_RS13195	SEQMU2_RS05015	-	-	-	-	-	SSP_RS00250
hypothetical protein	BEK99_RS11685	-	SE1039_RS13190	SEQMU2_RS05010	-	-	-	-	-	SSP_RS00255
hypothetical protein	BEK99_RS11690	-	-	SEQMU2_RS04920	-	-	-	-	BE24_RS11745	-
transcriptional regulator	BEK99_RS11695	-	-	SEQMU2_RS04915	-	-	-	-	-	-
UTP--glucose-1-phosphate uridylyltransferase	BEK99_RS11715	-	SE1039_RS13285	SEQMU2_RS04965	BK815_RS03795	A6V26_RS02965	AA913_RS07965	-	-	SSP_RS00300
copper-translocating P-type ATPase	BEK99_RS11730	-	-	-	-	-	-	-	BE24_RS11680	-
hypothetical protein	BEK99_RS11735	-	SE1039_RS00595	SEQMU2_RS06100	BK815_RS09080	A6V26_RS12900	AA913_RS07335	-	BE24_RS11675	-
DUF156 domain-containing protein	BEK99_RS11745	-	SE1039_RS12435	SEQMU2_RS04230	BK815_RS04240	A6V26_RS00400	AA913_RS03530	-	BE24_RS12625	-
dihydroneopterin aldolase	BEK99_RS11750	SCA_RS10950	SE1039_RS12430	SEQMU2_RS04225	BK815_RS04245	A6V26_RS12865	AA913_RS04465	-	BE24_RS12630	-
MBL fold metallo-hydrolase	BEK99_RS11755	-	SE1039_RS12425	SEQMU2_RS04220	BK815_RS04250	A6V26_RS12860	AA913_RS04470	-	BE24_RS12635	-
arsenical efflux pump membrane protein ArsB	BEK99_RS11770	-	SE1039_RS03985	SEQMU2_RS09200	BK815_RS00720	A6V26_RS12795	AA913_RS04535	SXYL_RS07145	BE24_RS04290	SSP_RS11975
MerR family transcriptional regulator	BEK99_RS11780	-	SE1039_RS13075	SEQMU2_RS04845	-	-	-	-	BE24_RS11695	-
type 1 glutamine amidotransferase domain-containing protein	BEK99_RS11785	-	SE1039_RS13070	SEQMU2_RS04840	-	-	-	-	BE24_RS11690	-
oxidoreductase	BEK99_RS11790	-	SE1039_RS13065	SEQMU2_RS04835	BK815_RS00065	A6V26_RS12050	AA913_RS11630	SXYL_RS03860	BE24_RS11685	SSP_RS03640
NADP-dependent oxidoreductase	BEK99_RS11795	-	SE1039_RS13060	SEQMU2_RS04690	BK815_RS12925	A6V26_RS06425	AA913_RS00125	SXYL_RS03625	BE24_RS08180	SSP_RS03405
hypothetical protein	BEK99_RS11800	-	SE1039_RS13055	-	-	-	-	-	-	-
NmrA family protein	BEK99_RS11810	-	SE1039_RS13045	-	BK815_RS11765	A6V26_RS07575	AA913_RS01280	SXYL_RS00230	BE24_RS11470	-
tryptophan-rich sensory protein	BEK99_RS11815	-	SE1039_RS13040	SEQMU2_RS08435	BK815_RS11760	A6V26_RS07580	AA913_RS01285	SXYL_RS00235	BE24_RS11465	-
hypothetical protein	BEK99_RS11820	-	SE1039_RS13035	-	-	-	-	SXYL_RS00240	BE24_RS11460	-
NAD(P)/FAD-dependent oxidoreductase	BEK99_RS11825	-	SE1039_RS13030	-	-	-	-	SXYL_RS00245	BE24_RS11455	-
glycosyl transferase family 2	BEK99_RS11830	-	SE1039_RS13025	-	-	-	-	SXYL_RS00250	BE24_RS11450	-
squalene synthase	BEK99_RS11835	-	SE1039_RS13020	-	-	-	-	SXYL_RS00255	BE24_RS11445	-
phytoene desaturase	BEK99_RS11840	-	SE1039_RS13015	-	-	-	-	SXYL_RS00260	BE24_RS11440	-
pyridoxamine 5-phosphate oxidase	BEK99_RS11845	-	SE1039_RS13010	SEQMU2_RS04095	BK815_RS11755	A6V26_RS07585	AA913_RS01290	SXYL_RS00265	BE24_RS11435	-
deoxyribodipyrimidine photo-lyase	BEK99_RS11850	-	SE1039_RS13005	-	BK815_RS11745	A6V26_RS07595	AA913_RS01300	SXYL_RS00270	BE24_RS11430	-
antibiotic biosynthesis monooxygenase	BEK99_RS11855	-	SE1039_RS13000	-	-	-	-	SXYL_RS00275	BE24_RS11425	-
HNH endonuclease	BEK99_RS11860	SCA_RS12305	-	-	-	-	-	-	-	-
MFS transporter	BEK99_RS11865	SCA_RS12300	-	-	-	-	-	-	-	-
MFS transporter	BEK99_RS11870	SCA_RS12295	-	-	-	-	-	-	-	-
cysteine hydrolase	BEK99_RS11875	SCA_RS12290	-	-	BK815_RS11530	A6V26_RS07810	AA913_RS01515	SXYL_RS02065	BE24_RS09720	SSP_RS01925
pyridoxal-dependent decarboxylase	BEK99_RS11880	SCA_RS12285	-	-	-	-	-	-	-	-

Product	*S. carnosus*		*S. equorum*		*S. succinus*			*S. xylosus*		*S. saprophyticus*
	**JCM 6069**	**TM300**	**KS1039**	**Mu2**	**14BME20**	**CSM 77**	**DSM 14617**	**C2a**	**HKUOPL8**	**ATCC 15305**
penicillin-binding protein	BEK99_RS11885	SCA_RS12280	-	-	-	-	-	-	-	-
hypothetical protein	BEK99_RS11890	SCA_RS12275	-	-	-	-	-	-	-	-
hypothetical protein	BEK99_RS11900	SCA_RS12265	-	-	-	-	-	-	-	-
PTS mannose transporter subunit IIA	BEK99_RS11905	SCA_RS12260	SE1039_RS11650	SEQMU2_RS03505	-	-	-	SXYL_RS01355	BE24_RS10390	-
PTS mannose transporter subunit IIABC	BEK99_RS11910	SCA_RS12255	SE1039_RS11645	SEQMU2_RS03500	-	-	-	SXYL_RS01360	-	-
mannose-6-phosphate isomerase class I	BEK99_RS11915	SCA_RS12250	SE1039_RS11640	SEQMU2_RS03495	BK815_RS10895	A6V26_RS08470	AA913_RS04250	SXYL_RS01365	BE24_RS10380	-
NAD(P)-dependent oxidoreductase	BEK99_RS11920	SCA_RS12245	-	-	-	-	-	-	-	SSP_RS10965
transcriptional regulator	BEK99_RS11925	SCA_RS12240	-	-	-	-	-	-	-	-
NAD-dependent dehydratase	BEK99_RS11930	-	-	-	-	A6V26_RS05075	AA913_RS01930	-	-	-
type 1 glutamine amidotransferase domain-containing protein	BEK99_RS11935	SCA_RS12230	-	-	BK815_RS03730	A6V26_RS05080	AA913_RS01935	-	-	-
multidrug efflux MATE transporter MepA	BEK99_RS11945	SCA_RS12220	-	-	-	-	-	-	-	SSP_RS10700
MarR family transcriptional regulator	BEK99_RS11950	SCA_RS12215	-	-	-	-	-	-	-	SSP_RS10705
hypothetical protein	BEK99_RS11955	SCA_RS12210	-	SEQMU2_RS04865	-	-	-	-	-	-
N-acetylmuramoyl-L-alanine amidase	BEK99_RS11960	SCA_RS12205	-	SEQMU2_RS04860	-	-	-	-	-	-
phage infection protein	BEK99_RS11965	SCA_RS12200	-	SEQMU2_RS04855	-	-	-	-	-	-
hypothetical protein	BEK99_RS11970	SCA_RS12195	-	SEQMU2_RS04850	-	-	-	-	-	-
hypothetical protein	BEK99_RS11975	-	-	SEQMU2_RS04735	BK815_RS09370	A6V26_RS05230	AA913_RS02085	SXYL_RS12690	BE24_RS12500	-
poly-beta-1,6-N-acetyl-D-glucosamine export protein	BEK99_RS11990	-	-	-	-	-	-	-	BE24_RS11200	-
intracellular adhesion protein D	BEK99_RS12000	-	-	-	-	-	-	-	BE24_RS13645	-
hypothetical protein	BEK99_RS12035	SCA_RS12185	-	-	-	-	-	-	-	-
LacI family transcriptional regulator	BEK99_RS12045	-	SE1039_RS12480	SEQMU2_RS04275	-	-	-	-	-	-
ribokinase	BEK99_RS12060	SCA_RS12155	SE1039_RS12495	SEQMU2_RS04290	-	-	-	-	-	-
N-acetyltransferase	BEK99_RS12065	SCA_RS12115	-	-	-	-	-	-	-	SSP_RS04235
acyl CoA:acetate/3-ketoacid CoA transferase	BEK99_RS12090	SCA_RS12090	SE1039_RS00315	SEQMU2_RS05820	-	A6V26_RS04685	AA913_RS07105	SXYL_RS12990	BE24_RS12240	SSP_RS12185
CDP-glycerol glycerophosphotransferase	BEK99_RS12095	SCA_RS12085	-	-	-	-	-	-	-	-
type 1 glutamine amidotransferase domain-containing protein	BEK99_RS12130	SCA_RS12050	-	-	-	-	-	-	-	-
magnesium transporter CorA	BEK99_RS12140	SCA_RS12040	-	-	-	-	-	-	-	-
hypothetical protein	BEK99_RS12145	SCA_RS12035	-	-	-	-	-	-	-	-
glucosamine-6-phosphate deaminase	BEK99_RS12150	SCA_RS12030	SE1039_RS11795	SEQMU2_RS03640	BK815_RS10825	A6V26_RS08540	AA913_RS10075	SXYL_RS01240	BE24_RS10485	-
amino acid permease	BEK99_RS12190	SCA_RS11990	-	-	-	-	-	-	-	-
50S rRNA methyltransferase	BEK99_RS12195	SCA_RS11985	-	-	-	-	-	-	-	SSP_RS00400

Product	*S. carnosus*		*S. equorum*		*S. succinus*			*S. xylosus*		*S. saprophyticus*
	**JCM 6069**	**TM300**	**KS1039**	**Mu2**	**14BME20**	**CSM 77**	**DSM 14617**	**C2a**	**HKUOPL8**	**ATCC 15305**
hypothetical protein	BEK99_RS12200	SCA_RS11980	-	-	-	-	-	-	-	-
MerR family transcriptional regulator	BEK99_RS12205	SCA_RS11975	-	-	-	-	-	-	-	-
hypothetical protein	BEK99_RS12215	SCA_RS11965	-	-	-	-	-	-	-	-
peptidase M23	BEK99_RS12225	SCA_RS11955	SE1039_RS12165	SEQMU2_RS04010	-	-	-	SXYL_RS00880	BE24_RS10820	-
hypothetical protein	BEK99_RS12230	SCA_RS11950	-	-	-	-	-	-	-	-
hypothetical protein	BEK99_RS12240	SCA_RS11945	SE1039_RS12445	SEQMU2_RS04240	BK815_RS04230	A6V26_RS00390	AA913_RS03540	-	-	-
hypothetical protein	BEK99_RS12255	-	SE1039_RS11985	SEQMU2_RS03830	BK815_RS10680	A6V26_RS08685	AA913_RS09930	SXYL_RS01055	BE24_RS10655	SSP_RS00925
acetoacetate decarboxylase	BEK99_RS12265	SCA_RS11915	-	SEQMU2_RS04770	BK815_RS09940	A6V26_RS05790	AA913_RS10435	-	-	-
hypothetical protein	BEK99_RS12270	-	-	-	BK815_RS10145	A6V26_RS05985	AA913_RS12550	SXYL_RS00500	BE24_RS11185	-
Fis family transcriptional regulator	BEK99_RS12275	SCA_RS11905	-	-	BK815_RS10150	A6V26_RS05990	AA913_RS12555	SXYL_RS00505	BE24_RS11180	-
ethanolamine utilization protein EutH	BEK99_RS12280	SCA_RS11900	-	-	BK815_RS10155	A6V26_RS05995	AA913_RS12560	SXYL_RS00510	BE24_RS11175	-
glutamine synthetase	BEK99_RS12290	SCA_RS11890	-	-	BK815_RS10165	A6V26_RS06005	AA913_RS12570	SXYL_RS00520	BE24_RS11165	-
NAD-dependent succinate-semialdehyde dehydrogenase	BEK99_RS12295	SCA_RS11885	-	-	BK815_RS10170	A6V26_RS06010	AA913_RS12575	SXYL_RS00525	BE24_RS11160	-
succinyl-diaminopimelate desuccinylase	BEK99_RS12300	SCA_RS11880	-	-	BK815_RS10175	A6V26_RS06015	AA913_RS12580	SXYL_RS00530	BE24_RS11155	-
hypothetical protein	BEK99_RS12315	SCA_RS11860	-	-	-	-	-	-	-	-
TetR family transcriptional regulator	BEK99_RS12320	SCA_RS12600	-	-	-	-	-	-	-	-
glycosyl hydrolase	BEK99_RS12325	-	SE1039_RS05575	SEQMU2_RS10425	-	-	-	-	-	-
hypothetical protein	BEK99_RS12330	SCA_RS11780	-	-	-	-	-	-	-	-
anion permease	BEK99_RS12335	SCA_RS11775	SE1039_RS12440	-	BK815_RS04235	A6V26_RS12875	AA913_RS04455	-	BE24_RS12620	-
CPBP family intramembrane metalloprotease domain-containing protein	BEK99_RS12340	SCA_RS11770	-	-	-	-	-	-	-	-
butanediol dehydrogenase	BEK99_RS12355	SCA_RS11755	-	SEQMU2_RS13890	-	A6V26_RS12960	AA913_RS04375	-	-	-
succinyl-diaminopimelate desuccinylase	BEK99_RS12360	SCA_RS11750	-	-	-	-	-	-	-	-
alpha/beta hydrolase	BEK99_RS12365	SCA_RS11745	-	-	-	-	-	-	-	-
NADH oxidase	BEK99_RS12370	SCA_RS11740	-	-	BK815_RS09400	A6V26_RS05285	AA913_RS02140	-	-	-
transcriptional regulator	BEK99_RS12375	SCA_RS11735	-	-	-	-	-	-	-	-
hypothetical protein	BEK99_RS12380	SCA_RS11730	-	-	-	-	-	-	-	-
zinc ribbon domain-containing protein	BEK99_RS12385	SCA_RS11725	-	SEQMU2_RS03630	-	-	-	-	-	-
hypothetical protein	BEK99_RS12390	SCA_RS11720	-	-	-	-	-	-	-	-
zinc ribbon domain-containing protein	BEK99_RS12395	SCA_RS11715	-	-	-	-	-	-	-	-
hypothetical protein	BEK99_RS12405	SCA_RS11705	-	-	-	-	-	-	-	-
hypothetical protein	BEK99_RS12420	SCA_RS11700	-	-	-	-	-	-	-	-

Product	*S. carnosus*		*S. equorum*		*S. succinus*			*S. xylosus*		*S. saprophyticus*
	**JCM 6069**	**TM300**	**KS1039**	**Mu2**	**14BME20**	**CSM 77**	**DSM 14617**	**C2a**	**HKUOPL8**	**ATCC 15305**
PTS sorbitol transporter subunit IIA	BEK99_RS12425	SCA_RS11695	-	SEQMU2_RS05740	BK815_RS08990	A6V26_RS04825	AA913_RS07245	-	BE24_RS11580	-
PTS sorbitol transporter subunit IIB	BEK99_RS12430	SCA_RS11690	-	SEQMU2_RS05735	BK815_RS08995	A6V26_RS04830	AA913_RS07250	-	BE24_RS11585	-
PTS sorbitol transporter subunit IIC	BEK99_RS12435	SCA_RS11685	-	SEQMU2_RS05730	BK815_RS09000	A6V26_RS04835	AA913_RS07255	-	BE24_RS11590	-
hypothetical protein	BEK99_RS12440	SCA_RS11680	-	SEQMU2_RS05725	BK815_RS09005	A6V26_RS04840	AA913_RS07260	-	BE24_RS11595	-
hypothetical protein	BEK99_RS12445	SCA_RS11675	-	SEQMU2_RS05720	BK815_RS09010	A6V26_RS04845	AA913_RS07265	-	BE24_RS11600	-
NAD(P)-dependent oxidoreductase	BEK99_RS12450	SCA_RS11670	-	SEQMU2_RS05715	BK815_RS09015	A6V26_RS04850	AA913_RS07270	-	BE24_RS11605	-
hypothetical protein	BEK99_RS12455	SCA_RS11665	-	-	-	-	-	-	-	-
hypothetical protein	BEK99_RS12460	SCA_RS11660	-	-	-	-	-	-	-	-
DUF4870 domain-containing protein	BEK99_RS12465	SCA_RS11655	-	-	-	-	-	-	-	-
WYL domain-containing protein	BEK99_RS12470	SCA_RS11650	-	-	-	-	-	-	-	-
hypothetical protein	BEK99_RS12475	SCA_RS11645	-	-	-	-	-	-	-	-
hypothetical protein	BEK99_RS12480	SCA_RS11640	-	-	-	-	-	-	-	-
hypothetical protein	BEK99_RS12485	SCA_RS11635	-	-	-	-	-	-	-	-
glycine/betaine ABC transporter permease	BEK99_RS12490	SCA_RS11630	SE1039_RS11345	SEQMU2_RS03220	-	-	-	-	-	-
hypothetical protein	BEK99_RS12500	SCA_RS11620	-	-	-	-	-	-	-	-
hypothetical protein	BEK99_RS12505	SCA_RS11615	-	-	-	-	-	-	-	-
hypothetical protein	BEK99_RS12510	SCA_RS11610	-	-	-	-	-	-	-	-
hypothetical protein	BEK99_RS12515	SCA_RS11605	-	-	-	-	-	-	-	-
hypothetical protein	BEK99_RS12520	SCA_RS11600	-	-	-	-	-	-	BE24_RS11640	-
hypothetical protein	BEK99_RS12530	SCA_RS11590	SE1039_RS12150	SEQMU2_RS03995	-	-	-	-	-	-
hypothetical protein	BEK99_RS12535	SCA_RS11585	-	-	-	-	-	-	-	-
hypothetical protein	BEK99_RS12540	SCA_RS11580	-	-	-	-	-	-	-	-
hypothetical protein	BEK99_RS12555	SCA_RS11560	-	-	-	-	-	-	-	-
hypothetical protein	BEK99_RS12560	SCA_RS11555	-	-	-	-	-	-	-	-
hypothetical protein	BEK99_RS12565	SCA_RS11550	SE1039_RS12505	SEQMU2_RS04300	-	-	-	-	-	SSP_RS00630
MFS transporter permease	BEK99_RS12570	SCA_RS11545	SE1039_RS12180	SEQMU2_RS04025	BK815_RS10465	A6V26_RS08880	AA913_RS09735	-	-	SSP_RS00805
aldo/keto reductase	BEK99_RS12580	SCA_RS11535	-	-	-	-	-	SXYL_RS11490	BE24_RS00255	SSP_RS10945
short-chain dehydrogenase	BEK99_RS12590	SCA_RS11525	-	-	-	-	-	-	-	-
hypothetical protein	BEK99_RS12600	SCA_RS11510	-	-	-	-	-	-	-	-
hypothetical protein	BEK99_RS12610	SCA_RS11505	-	-	-	-	-	-	-	-
hypothetical protein	BEK99_RS13155	SCA_RS12595	-	-	-	-	-	-	-	-

Product	S. carnosus		S. equorum		S. succinus			S. xylosus		S. saprophyticus
	JCM 6069	TM300	KS1039	Mu2	14BME20	CSM 77	DSM 14617	C2a	HKUOPL8	ATCC 15305
hypothetical protein	BEK99_RS12635	SCA_RS11480	-	-	-	-	-	-	-	-
hypothetical protein	BEK99_RS12640	SCA_RS11475	-	-	-	-	-	-	-	-
MFS transporter	BEK99_RS12645	SCA_RS11470	-	-	-	-	-	-	-	-
hypothetical protein	BEK99_RS12650	SCA_RS11465	-	-	BK815_RS02115	-	-	-	-	SSP_RS12785
squalene synthase	BEK99_RS12660	SCA_RS11455	-	-	-	-	-	-	-	-
pyridoxamine 5-phosphate oxidase	BEK99_RS12665	SCA_RS11450	-	-	-	-	-	-	-	-
YhgE/Pip domain-containing protein	BEK99_RS12675	SCA_RS11440	SE1039_RS12325	SEQMU2_RS04185	BK815_RS10185	A6V26_RS06025	AA913_RS12590	SXYL_RS00665	-	SSP_RS00680
NADPH:quinone reductase	BEK99_RS12685	SCA_RS11430	-	-	BK815_RS09410	A6V26_RS05295	AA913_RS02150	-	-	-
short-chain dehydrogenase/reductase	BEK99_RS12690	SCA_RS11425	SE1039_RS00635	-	BK815_RS09465	A6V26_RS05350	AA913_RS02205	-	-	-
hypothetical protein	BEK99_RS12695	SCA_RS11420	-	-	-	-	-	-	-	-
amidohydrolase	BEK99_RS12710	SCA_RS11405	-	-	-	-	-	-	-	-
hypothetical protein	BEK99_RS12715	SCA_RS11400	-	-	-	-	-	-	-	-
HAD family phosphatase	BEK99_RS12720	SCA_RS11395	-	SEQMU2_RS00580	-	-	-	-	-	SSP_RS12105
hypothetical protein	BEK99_RS12735	SCA_RS11380	-	-	-	-	-	-	-	-
alpha/beta hydrolase	BEK99_RS12770	-	SE1039_RS00180	-	-	-	-	-	-	-
hypothetical protein	BEK99_RS12780	-	SE1039_RS00745	SEQMU2_RS06270	BK815_RS08270	A6V26_RS04110	AA913_RS06770	SXYL_RS12505	BE24_RS12690	SSP_RS11840
hypothetical protein	BEK99_RS12785	-	SE1039_RS05275	-	-	-	-	-	-	-
gamma-glutamyltranspeptidase	BEK99_RS12790	-	SE1039_RS11155	SEQMU2_RS03030	BK815_RS11370	A6V26_RS07990	AA913_RS01695	SXYL_RS01875	BE24_RS09890	SSP_RS01705
capsule biosynthesis protein CapA	BEK99_RS12800	-	SE1039_RS11160	SEQMU2_RS03035	BK815_RS11365	A6V26_RS07995	AA913_RS01700	SXYL_RS01870	-	SSP_RS01700
poly-gamma-glutamate biosynthesis protein PgsC	BEK99_RS12805	-	SE1039_RS11165	SEQMU2_RS03040	-	A6V26_RS08000	-	SXYL_RS01865	BE24_RS09900	SSP_RS01695
poly-gamma-glutamate synthase PgsB	BEK99_RS12810	-	SE1039_RS11170	SEQMU2_RS03045	BK815_RS11360	A6V26_RS08005	AA913_RS01710	SXYL_RS01860	BE24_RS09905	SSP_RS01690
hypothetical protein	BEK99_RS12820	-	-	-	BK815_RS10455	A6V26_RS08890	AA913_RS09725	-	-	SSP_RS00795
DUF3100 domain-containing protein	BEK99_RS12825	-	-	-	-	A6V26_RS08885	AA913_RS09730	-	-	SSP_RS00800
ornithine carbamoyltransferase	BEK99_RS12845	SCA_RS11370	-	-	-	-	-	-	-	-
carbamate kinase	BEK99_RS12850	SCA_RS11365	-	-	BK815_RS08290	A6V26_RS04125	AA913_RS06545	SXYL_RS12190	-	-
YfcC family protein	BEK99_RS12855	SCA_RS11360	-	-	-	-	-	-	-	-
hypothetical protein	BEK99_RS12860	SCA_RS11355	-	-	-	-	-	-	-	-
YfcC family protein	BEK99_RS12865	SCA_RS11350	-	-	-	-	-	-	-	-
carbamate kinase	BEK99_RS12870	SCA_RS11345	-	-	-	-	-	-	BE24_RS13080	-
RNA-binding protein	BEK99_RS12880	SCA_RS11335	-	-	-	-	-	-	-	-
membrane protein	BEK99_RS12885	SCA_RS11330	-	-	-	-	-	-	-	SSP_RS00830

Product	*S. carnosus*		*S. equorum*		*S. succinus*			*S. xylosus*		*S. saprophyticus*
	**JCM 6069**	**TM300**	**KS1039**	**Mu2**	**14BME20**	**CSM 77**	**DSM 14617**	**C2a**	**HKUOPL8**	**ATCC 15305**
hypothetical protein	BEK99_RS12890	SCA_RS11325	-	-	-	-	-	-	-	-
hypothetical protein	BEK99_RS12895	SCA_RS11320	-	-	-	-	-	-	-	-
hypothetical protein	BEK99_RS12900	SCA_RS11315	-	-	-	-	-	-	-	-
LysR family transcriptional regulator	BEK99_RS12905	SCA_RS11310	-	-	-	-	-	-	-	-
ABC transporter ATP-binding protein	BEK99_RS12910	SCA_RS12585	-	-	-	-	-	-	-	-
hypothetical protein	BEK99_RS12925	SCA_RS11290	-	-	-	-	-	-	-	-
osmoprotectant ABC transporter substrate-binding protein	BEK99_RS12930	-	-	-	-	-	-	-	-	SSP_RS05100
CPBP family intramembrane metalloprotease	BEK99_RS12935	SCA_RS11280	-	-	-	-	-	-	-	-
NmrA/HSCARG family protein	BEK99_RS12940	SCA_RS11275	-	-	-	-	-	-	-	-
TetR family transcriptional regulator	BEK99_RS12945	SCA_RS11270	-	-	-	-	-	-	-	-
short-chain dehydrogenase	BEK99_RS12950	SCA_RS11265	-	-	-	-	-	-	-	-
TetR/AcrR family transcriptional regulator	BEK99_RS12955	SCA_RS11260	SE1039_RS00195	-	-	-	-	-	-	-
acyl esterase	BEK99_RS12960	SCA_RS11255	SE1039_RS00190	-	-	-	-	-	-	-
tryptophan-rich sensory protein	BEK99_RS12970	SCA_RS11245	-	-	-	-	-	-	-	-
hypothetical protein	BEK99_RS12975	SCA_RS11240	-	-	-	-	-	-	-	-
thiaminase II	BEK99_RS12980	SCA_RS11235	-	-	-	-	-	-	-	-
amidohydrolase	BEK99_RS12985	SCA_RS11230	-	-	-	-	-	-	-	-
biotin transporter BioY	BEK99_RS12990	SCA_RS11225	-	-	-	-	-	-	-	-
dethiobiotin synthase	BEK99_RS12995	SCA_RS11220	-	-	BK815_RS08665	A6V26_RS04500	AA913_RS06920	-	-	-
hypothetical protein	BEK99_RS13005	SCA_RS11210	-	-	-	-	-	-	-	-
hypothetical protein	BEK99_RS13010	SCA_RS12580	-	-	-	-	-	-	-	-
5-methyltetrahydropteroyltriglutamate-- homocysteine S-methyltransferase	-	SCA_RS00055	SE1039_RS00365	SEQMU2_RS05870	BK815_RS08815	A6V26_RS04650	AA913_RS07070	SXYL_RS12935	BE24_RS12295	SSP_RS12130
assimilatory sulfite reductase (NADPH) hemoprotein subunit	-	SCA_RS00285	SE1039_RS00410	SEQMU2_RS05915	BK815_RS08775	A6V26_RS04610	AA913_RS07030	SXYL_RS12895	BE24_RS12330	SSP_RS12085
adenylyl-sulfate kinase	-	SCA_RS00305	SE1039_RS00440	SEQMU2_RS05945	BK815_RS08745	A6V26_RS04580	AA913_RS07000	SXYL_RS12865	BE24_RS12360	SSP_RS12055
FAD-dependent monooxygenase	-	SCA_RS00355	SE1039_RS00185	-	-	-	-	-	-	-
DNA polymerase III subunit delta	-	SCA_RS00610	SE1039_RS01195	SEQMU2_RS13765	BK815_RS08110	A6V26_RS13030	AA913_RS12175	SXYL_RS11970	BE24_RS06895	SSP_RS11435
tRNA1(Val) (adenine(37)-N6)-methyltransferase	-	SCA_RS00625	SE1039_RS01210	SEQMU2_RS13750	BK815_RS08095	A6V26_RS13045	AA913_RS12190	SXYL_RS11955	BE24_RS06880	SSP_RS11420
PLP-dependent aminotransferase family protein	-	SCA_RS00895	SE1039_RS01445	SEQMU2_RS00005	BK815_RS07855	A6V26_RS03970	AA913_RS10800	SXYL_RS11735	BE24_RS00005	SSP_RS11185
PLP-dependent aminotransferase family protein	-	SCA_RS01300	-	SEQMU2_RS07355	BK815_RS06935	A6V26_RS03060	AA913_RS07875	SXYL_RS10620	BE24_RS01110	SSP_RS10215
phosphonate ABC transporter ATP-binding protein	-	SCA_RS01405	SE1039_RS02350	SEQMU2_RS07135	BK815_RS07155	A6V26_RS03280	AA913_RS07655	SXYL_RS10845	BE24_RS00885	SSP_RS10440
aspartate aminotransferase family protein	-	SCA_RS01445	-	-	-	-	-	SXYL_RS01880	BE24_RS09885	SSP_RS01710

Product	*S. carnosus*		*S. equorum*		*S. succinus*			*S. xylosus*		*S. saprophyticus*
	**JCM 6069**	**TM300**	**KS1039**	**Mu2**	**14BME20**	**CSM 77**	**DSM 14617**	**C2a**	**HKUOPL8**	**ATCC 15305**
teichoic acid biosynthesis protein	-	SCA_RS01475	SE1039_RS02385	SEQMU2_RS07170	BK815_RS07120	A6V26_RS03245	AA913_RS07690	SXYL_RS10810	BE24_RS00920	SSP_RS10405
alpha/beta hydrolase	-	SCA_RS01530	SE1039_RS02455	SEQMU2_RS07240	BK815_RS07050	A6V26_RS03175	AA913_RS07760	SXYL_RS10740	BE24_RS00990	SSP_RS10335
ABC transporter ATP-binding protein	-	SCA_RS01550	SE1039_RS11410	SEQMU2_RS03265	BK815_RS11160	A6V26_RS08205	AA913_RS03985	SXYL_RS01615	BE24_RS10130	SSP_RS01455
bacitracin ABC transporter permease	-	SCA_RS01555	SE1039_RS11405	SEQMU2_RS03260	BK815_RS11165	A6V26_RS08200	AA913_RS03980	SXYL_RS01620	BE24_RS10125	SSP_RS01460
MFS transporter	-	SCA_RS01575	SE1039_RS00575	SEQMU2_RS06080	BK815_RS08635	A6V26_RS04470	AA913_RS06890	SXYL_RS12810	BE24_RS12415	-
hypothetical protein	-	SCA_RS01680	SE1039_RS02585	SEQMU2_RS07375	BK815_RS06915	A6V26_RS03040	AA913_RS07895	SXYL_RS10600	-	-
UDP-N-acetylenolpyruvoylglucosamine reductase	-	SCA_RS01970	SE1039_RS02865	SEQMU2_RS07655	BK815_RS06630	A6V26_RS02755	AA913_RS08175	SXYL_RS10325	BE24_RS01410	SSP_RS09915
HPr kinase/phosphorylase	-	SCA_RS02080	SE1039_RS02975	SEQMU2_RS07765	BK815_RS06520	A6V26_RS02645	AA913_RS08285	SXYL_RS10215	BE24_RS01520	SSP_RS09805
hypothetical protein	-	SCA_RS02240	SE1039_RS03205	SEQMU2_RS08290	BK815_RS02655	-	-	SXYL_RS10075	-	-
single-stranded DNA-binding protein	-	SCA_RS02420	-	-	-	-	-	SXYL_RS08740	-	-
dUTP pyrophosphatase	-	SCA_RS02485	-	-	-	-	-	-	BE24_RS05175	-
hypothetical protein	-	SCA_RS02520	-	-	BK815_RS02785	A6V26_RS11110	AA913_RS06260	-	-	-
hypothetical protein	-	SCA_RS02630	-	-	-	-	-	SXYL_RS08520	BE24_RS05070	-
hypothetical protein	-	SCA_RS02635	-	-	-	-	-	SXYL_RS08515	BE24_RS05065	-
phage holin	-	SCA_RS02645	-	-	-	A6V26_RS11000	-	-	-	-
amidase	-	SCA_RS02650	-	-	-	A6V26_RS10995	-	SXYL_RS08500	-	-
glycerophosphodiester phosphodiesterase	-	SCA_RS02665	-	-	-	-	-	-	BE24_RS04015	-
bifunctional metallophosphatase/5-nucleotidase	-	SCA_RS02695	SE1039_RS03425	SEQMU2_RS08650	BK815_RS06085	A6V26_RS02230	AA913_RS08820	SXYL_RS09810	BE24_RS01920	SSP_RS09300
D-alanine--poly(phosphoribitol) ligase	-	SCA_RS02735	SE1039_RS03470	SEQMU2_RS08695	BK815_RS06045	A6V26_RS02190	AA913_RS08860	SXYL_RS09770	BE24_RS01960	SSP_RS09260
serine protease	-	SCA_RS03175	SE1039_RS03885	SEQMU2_RS09115	BK815_RS05635	A6V26_RS01770	AA913_RS13045	SXYL_RS09375	BE24_RS02355	SSP_RS08860
dihydrolipoamide dehydrogenase	-	SCA_RS03190	SE1039_RS00760	SEQMU2_RS06285	BK815_RS08505	A6V26_RS04340	AA913_RS06760	SXYL_RS12490	BE24_RS12705	SSP_RS11820
acyltransferase	-	SCA_RS03345	SE1039_RS04045	SEQMU2_RS09255	BK815_RS05535	A6V26_RS01670	AA913_RS02260	SXYL_RS09275	BE24_RS02455	SSP_RS08760
methicillin resistance protein FmtA	-	SCA_RS03380	SE1039_RS04080	SEQMU2_RS09290	BK815_RS05500	A6V26_RS01635	AA913_RS02295	SXYL_RS09240	BE24_RS02490	SSP_RS08725
cytochrome aa3 quinol oxidase subunit II	-	SCA_RS03460	SE1039_RS04105	SEQMU2_RS09315	BK815_RS05475	A6V26_RS01610	AA913_RS02320	SXYL_RS09215	BE24_RS02515	SSP_RS08700
ABC transporter ATP-binding protein	-	SCA_RS03550	SE1039_RS04185	SEQMU2_RS09395	BK815_RS05400	A6V26_RS01535	AA913_RS02395	SXYL_RS09135	BE24_RS02595	SSP_RS08620
class I SAM-dependent rRNA methyltransferase	-	SCA_RS03565	SE1039_RS04200	SEQMU2_RS09410	BK815_RS05385	A6V26_RS01520	AA913_RS02410	SXYL_RS09120	BE24_RS02610	SSP_RS08605
phenylalanine--tRNA ligase subunit beta	-	SCA_RS03835	SE1039_RS04445	SEQMU2_RS09655	BK815_RS05125	A6V26_RS01265	AA913_RS02665	SXYL_RS08865	BE24_RS02865	SSP_RS08345
guanylate kinase	-	SCA_RS04180	SE1039_RS04755	SEQMU2_RS09935	BK815_RS04815	A6V26_RS00975	AA913_RS02955	SXYL_RS08270	-	SSP_RS07910
L-serine dehydratase iron-sulfur-dependent subunit alpha	-	SCA_RS04295	SE1039_RS05180	SEQMU2_RS10030	BK815_RS04700	A6V26_RS00860	AA913_RS03070	SXYL_RS08175	BE24_RS03245	SSP_RS07820
DNA helicase RecG	-	SCA_RS04300	SE1039_RS05185	SEQMU2_RS10035	BK815_RS04695	A6V26_RS00855	AA913_RS03075	SXYL_RS08170	BE24_RS03250	SSP_RS07815
iron citrate ABC transporter substrate-binding protein	-	SCA_RS04395	-	-	-	-	-	-	-	SSP_RS00900

Product	*S. carnosus*		*S. equorum*		*S. succinus*			*S. xylosus*		*S. saprophyticus*
	**JCM 6069**	**TM300**	**KS1039**	**Mu2**	**14BME20**	**CSM 77**	**DSM 14617**	**C2a**	**HKUOPL8**	**ATCC 15305**
tRNA (N6-isopentenyl adenosine(37)-C2)-methylthiotransferase MiaB	-	SCA_RS04735	SE1039_RS05545	SEQMU2_RS10395	BK815_RS04340	A6V26_RS00500	AA913_RS03430	SXYL_RS07805	BE24_RS03615	SSP_RS07450
type I glutamate--ammonia ligase	-	SCA_RS04815	SE1039_RS05665	SEQMU2_RS10515	BK815_RS04255	A6V26_RS00415	AA913_RS03515	SXYL_RS07720	BE24_RS03700	SSP_RS07360
AI-2E family transporter	-	SCA_RS05025	SE1039_RS05900	SEQMU2_RS10995	BK815_RS04030	A6V26_RS00190	AA913_RS03745	SXYL_RS07510	BE24_RS03935	SSP_RS07095
aldo/keto reductase	-	SCA_RS05640	SE1039_RS06645	SEQMU2_RS11755	BK815_RS03155	A6V26_RS09540	AA913_RS04685	SXYL_RS06695	BE24_RS04665	SSP_RS06375
hypothetical protein	-	SCA_RS05645	SE1039_RS06650	SEQMU2_RS11760	BK815_RS03150	A6V26_RS09545	AA913_RS04690	SXYL_RS06690	BE24_RS12145	SSP_RS06370
pyrroline-5-carboxylate reductase	-	SCA_RS05650	SE1039_RS06655	SEQMU2_RS11765	BK815_RS03145	A6V26_RS09550	AA913_RS04695	SXYL_RS06685	BE24_RS04675	SSP_RS06365
exodeoxyribonuclease VII small subunit	-	SCA_RS05740	SE1039_RS06750	SEQMU2_RS11860	BK815_RS03055	A6V26_RS09640	AA913_RS04785	SXYL_RS06580	BE24_RS04770	SSP_RS06275
hypothetical protein	-	SCA_RS05780	SE1039_RS06790	SEQMU2_RS11900	BK815_RS03015	A6V26_RS09680	AA913_RS04825	SXYL_RS06540	BE24_RS04810	SSP_RS06235
glycine dehydrogenase (aminomethyl-transferring)	-	SCA_RS05805	SE1039_RS06815	SEQMU2_RS11925	BK815_RS02990	A6V26_RS09705	AA913_RS04850	SXYL_RS06515	BE24_RS04835	SSP_RS06210
competence protein ComG	-	SCA_RS05840	SE1039_RS06855	SEQMU2_RS11965	BK815_RS02950	A6V26_RS09745	AA913_RS04890	SXYL_RS06475	BE24_RS04875	SSP_RS06170
DNA repair protein RecO	-	SCA_RS05950	SE1039_RS06970	SEQMU2_RS12080	BK815_RS02520	A6V26_RS09860	AA913_RS05005	SXYL_RS06360	BE24_RS04990	SSP_RS06055
elongation factor 4	-	SCA_RS06040	SE1039_RS07055	SEQMU2_RS12165	BK815_RS02435	A6V26_RS09945	AA913_RS05090	SXYL_RS06275	BE24_RS05355	SSP_RS05970
DNA polymerase III subunit delta	-	SCA_RS06050	SE1039_RS07065	SEQMU2_RS12175	BK815_RS02425	A6V26_RS09955	AA913_RS05100	SXYL_RS06265	BE24_RS05375	SSP_RS05960
peptidase U32	-	SCA_RS06130	SE1039_RS07175	SEQMU2_RS12285	BK815_RS02315	A6V26_RS10065	AA913_RS05210	SXYL_RS06150	BE24_RS05490	SSP_RS05850
recombinase RarA	-	SCA_RS06195	SE1039_RS07250	SEQMU2_RS12355	BK815_RS02245	A6V26_RS10135	AA913_RS05280	SXYL_RS06080	BE24_RS05560	SSP_RS05775
50S ribosomal protein L21	-	SCA_RS06290	SE1039_RS07345	SEQMU2_RS12450	BK815_RS02145	A6V26_RS10235	AA913_RS05375	SXYL_RS05985	BE24_RS05655	SSP_RS05680
dephospho-CoA kinase	-	SCA_RS06450	SE1039_RS07515	SEQMU2_RS12630	BK815_RS01930	A6V26_RS10440	AA913_RS05580	SXYL_RS05825	BE24_RS05815	SSP_RS05515
DNA polymerase I	-	SCA_RS06460	SE1039_RS07525	SEQMU2_RS12640	BK815_RS01920	A6V26_RS10450	AA913_RS05590	SXYL_RS05815	BE24_RS05825	SSP_RS05505
isocitrate dehydrogenase (NADP(+))	-	SCA_RS06475	SE1039_RS07540	SEQMU2_RS12655	BK815_RS01905	A6V26_RS10465	AA913_RS05605	SXYL_RS05800	BE24_RS05840	SSP_RS05490
acetyl-CoA carboxylase carboxyl transferase subunit beta	-	SCA_RS06505	SE1039_RS07570	SEQMU2_RS12685	BK815_RS01875	A6V26_RS10495	AA913_RS05635	SXYL_RS05770	BE24_RS05870	SSP_RS05460
bifunctional oligoribonuclease/PAP phosphatase NrnA	-	SCA_RS06520	SE1039_RS07585	SEQMU2_RS12700	BK815_RS01860	A6V26_RS10510	AA913_RS05650	SXYL_RS05755	BE24_RS05885	SSP_RS05445
hypothetical protein	-	SCA_RS06525	SE1039_RS07590	SEQMU2_RS12705	BK815_RS01855	A6V26_RS10515	AA913_RS05655	SXYL_RS05750	BE24_RS05890	SSP_RS05440
acetate kinase	-	SCA_RS06555	SE1039_RS07630	SEQMU2_RS12745	BK815_RS01815	A6V26_RS10555	AA913_RS05695	SXYL_RS05710	BE24_RS05930	SSP_RS05400
PTS glucose transporter subunit IIBC	-	SCA_RS06625	SE1039_RS07715	SEQMU2_RS12830	BK815_RS01730	A6V26_RS10640	AA913_RS05780	SXYL_RS05625	BE24_RS06015	SSP_RS05315
acetate--CoA ligase	-	SCA_RS06655	SE1039_RS07745	SEQMU2_RS12870	BK815_RS01700	A6V26_RS10670	AA913_RS05810	SXYL_RS05595	BE24_RS06045	SSP_RS05285
acetoin utilization protein AcuC	-	SCA_RS06665	SE1039_RS07755	SEQMU2_RS12880	BK815_RS01690	A6V26_RS10680	AA913_RS05820	SXYL_RS05585	BE24_RS06055	SSP_RS05275
DNA translocase FtsK	-	SCA_RS06695	SE1039_RS07785	SEQMU2_RS12910	BK815_RS01660	A6V26_RS10710	AA913_RS05850	SXYL_RS05555	BE24_RS06090	SSP_RS05245
L-lactate dehydrogenase	-	SCA_RS06795	SE1039_RS07890	SEQMU2_RS13015	BK815_RS01555	A6V26_RS10835	AA913_RS05970	SXYL_RS05450	BE24_RS06195	SSP_RS05140
bifunctional 3,4-dihydroxy-2-butanone-4-phosphate synthase/GTP cyclohydrolase II	-	SCA_RS06815	SE1039_RS07910	SEQMU2_RS13035	BK815_RS01535	A6V26_RS10855	AA913_RS05990	SXYL_RS05430	BE24_RS06215	SSP_RS05120
autolysin	-	SCA_RS06835	SE1039_RS07935	SEQMU2_RS13060	BK815_RS01510	A6V26_RS10880	AA913_RS06015	SXYL_RS05405	BE24_RS06240	SSP_RS05085
putative L-aspartate oxidase	-	SCA_RS07005	-	-	BK815_RS10040	A6V26_RS05880	AA913_RS10345	-	-	-

Product	*S. carnosus*		*S. equorum*		*S. succinus*			*S. xylosus*		*S. saprophyticus*
	**JCM 6069**	**TM300**	**KS1039**	**Mu2**	**14BME20**	**CSM 77**	**DSM 14617**	**C2a**	**HKUOPL8**	**ATCC 15305**
histidine kinase	-	SCA_RS07115	SE1039_RS08200	SEQMU2_RS13330	BK815_RS01220	A6V26_RS11455	AA913_RS12120	SXYL_RS05000	BE24_RS06555	SSP_RS04825
tRNA (cytidine(34)-2-O)-methyltransferase	-	SCA_RS07140	SE1039_RS08230	SEQMU2_RS13360	BK815_RS01190	A6V26_RS11485	AA913_RS12090	SXYL_RS04970	BE24_RS06585	SSP_RS04795
glutamate-1-semialdehyde 2,1-aminomutase	-	SCA_RS07315	SE1039_RS08415	SEQMU2_RS13525	BK815_RS01005	A6V26_RS12205	AA913_RS11745	SXYL_RS04780	BE24_RS06955	SSP_RS04605
teichoic acid ABC transporter ATP-binding protein	-	SCA_RS07350	SE1039_RS08450	SEQMU2_RS00325	BK815_RS00970	A6V26_RS12240	AA913_RS11780	SXYL_RS04745	BE24_RS06990	SSP_RS04570
transporter	-	SCA_RS07425	SE1039_RS08515	SEQMU2_RS00390	BK815_RS00900	A6V26_RS12310	AA913_RS11850	SXYL_RS04670	BE24_RS07065	SSP_RS04500
aromatic acid exporter family protein	-	SCA_RS07440	SE1039_RS08530	SEQMU2_RS00405	BK815_RS00885	A6V26_RS12325	AA913_RS11865	SXYL_RS04655	BE24_RS07080	SSP_RS04485
Asp-tRNA(Asn)/Glu-tRNA(Gln) amidotransferase GatCAB subunit A	-	SCA_RS07485	SE1039_RS08580	SEQMU2_RS00455	BK815_RS00835	A6V26_RS12375	AA913_RS11915	SXYL_RS04605	BE24_RS07130	SSP_RS04420
excinuclease ABC subunit UvrA	-	SCA_RS07560	SE1039_RS04375	SEQMU2_RS09585	BK815_RS05195	A6V26_RS01335	AA913_RS02595	SXYL_RS08935	BE24_RS02795	-
hypothetical protein	-	SCA_RS07700	SE1039_RS03220	SEQMU2_RS08430	BK815_RS11750	A6V26_RS07590	AA913_RS01295	SXYL_RS10025	BE24_RS01700	SSP_RS00455
hypothetical protein	-	SCA_RS07705	SE1039_RS03180	SEQMU2_RS08390	BK815_RS05585	A6V26_RS01720	AA913_RS13095	SXYL_RS05295	BE24_RS11475	SSP_RS08140
hypothetical protein	-	SCA_RS07725	-	SEQMU2_RS06475	-	-	-	-	-	SSP_RS09670
hypothetical protein	-	SCA_RS07730	-	-	-	-	-	-	-	SSP_RS09675
hypothetical protein	-	SCA_RS07735	-	SEQMU2_RS05025	-	-	-	-	-	SSP_RS09680
terminase	-	SCA_RS07740	-	-	-	-	-	-	-	SSP_RS09690
hypothetical protein	-	SCA_RS07750	-	-	-	-	-	-	-	SSP_RS09705
hypothetical protein	-	SCA_RS07755	-	-	-	-	-	-	-	SSP_RS09710
hypothetical protein	-	SCA_RS07760	-	-	-	-	-	-	-	SSP_RS09715
primase	-	SCA_RS07765	-	SEQMU2_RS08245	-	-	-	-	-	SSP_RS09720
mobile element-associated protein	-	SCA_RS07770	-	SEQMU2_RS06455	-	-	-	-	BE24_RS12875	-
hypothetical protein	-	SCA_RS07775	-	SEQMU2_RS08240	-	-	-	-	BE24_RS12870	SSP_RS09725
excisionase	-	SCA_RS07780	-	SEQMU2_RS00295	-	-	-	-	-	-
phage repressor protein	-	SCA_RS07785	-	SEQMU2_RS08230	-	-	-	SXYL_RS07085	-	SSP_RS09740
transcriptional regulator	-	SCA_RS07790	-	SEQMU2_RS06435	-	-	-	-	BE24_RS12855	-
site-specific integrase	-	SCA_RS07800	-	SEQMU2_RS00280	-	-	-	-	-	-
threonine dehydratase	-	SCA_RS07940	SE1039_RS08915	SEQMU2_RS00805	BK815_RS00480	A6V26_RS12735	AA913_RS13370	SXYL_RS04270	BE24_RS07470	SSP_RS04065
SprT family protein	-	SCA_RS07965	SE1039_RS08945	SEQMU2_RS00820	BK815_RS00450	A6V26_RS11660	AA913_RS11240	SXYL_RS04240	BE24_RS07515	SSP_RS04030
N-acetyltransferase	-	SCA_RS08080	SE1039_RS10365	SEQMU2_RS02225	BK815_RS06340	A6V26_RS02485	AA913_RS08440	SXYL_RS02800	BE24_RS08975	SSP_RS02590
PTS mannitol transferase subunit IIB	-	SCA_RS08410	SE1039_RS09385	SEQMU2_RS01260	BK815_RS00020	A6V26_RS12095	AA913_RS11675	SXYL_RS03815	BE24_RS07980	SSP_RS03595
mannitol-1-phosphate 5-dehydrogenase	-	SCA_RS08425	SE1039_RS09400	SEQMU2_RS01275	-	A6V26_RS12110	AA913_RS11690	SXYL_RS03800	BE24_RS07995	SSP_RS03580
hemolysin III	-	SCA_RS08520	SE1039_RS09485	SEQMU2_RS01345	BK815_RS13025	A6V26_RS06325	AA913_RS00025	SXYL_RS03715	BE24_RS08090	SSP_RS03495
choline transporter	-	SCA_RS08605	SE1039_RS09560	SEQMU2_RS01420	BK815_RS12935	A6V26_RS06415	AA913_RS00115	SXYL_RS03635	BE24_RS08170	SSP_RS03415

Product	*S. carnosus*		*S. equorum*		*S. succinus*			*S. xylosus*		*S. saprophyticus*
	**JCM 6069**	**TM300**	**KS1039**	**Mu2**	**14BME20**	**CSM 77**	**DSM 14617**	**C2a**	**HKUOPL8**	**ATCC 15305**
pyruvate phosphate dikinase	-	SCA_RS08635	SE1039_RS12035	SEQMU2_RS03880	BK815_RS10620	A6V26_RS08745	AA913_RS09870	SXYL_RS01005	BE24_RS10705	SSP_RS00890
molybdate ABC transporter substrate-binding protein	-	SCA_RS08995	SE1039_RS09895	SEQMU2_RS01755	BK815_RS12600	A6V26_RS06745	AA913_RS00445	SXYL_RS03300	BE24_RS08505	SSP_RS03075
urease subunit gamma	-	SCA_RS09055	SE1039_RS11560	SEQMU2_RS03415	BK815_RS10980	A6V26_RS08385	AA913_RS04165	SXYL_RS01450	BE24_RS10295	SSP_RS01290
urease subunit beta	-	SCA_RS09060	SE1039_RS11565	SEQMU2_RS03420	BK815_RS10975	A6V26_RS08390	AA913_RS04170	SXYL_RS01445	BE24_RS10300	SSP_RS01285
urease accessory protein	-	SCA_RS09085	SE1039_RS11590	SEQMU2_RS03445	BK815_RS10950	A6V26_RS08415	AA913_RS04195	SXYL_RS01420	BE24_RS10325	SSP_RS01260
LLM class flavin-dependent oxidoreductase	-	SCA_RS09100	SE1039_RS09980	SEQMU2_RS01840	BK815_RS12535	A6V26_RS06810	AA913_RS00510	SXYL_RS03215	BE24_RS08590	SSP_RS02990
imidazolonepropionase	-	SCA_RS09275	SE1039_RS10180	SEQMU2_RS02040	BK815_RS12340	A6V26_RS07005	AA913_RS00705	SXYL_RS03020	BE24_RS08785	SSP_RS02785
two-component sensor histidine kinase	-	SCA_RS09460	SE1039_RS10335	SEQMU2_RS02195	BK815_RS12190	A6V26_RS07155	AA913_RS00855	SXYL_RS02845	BE24_RS08945	SSP_RS02620
malate:quinone oxidoreductase	-	SCA_RS09465	SE1039_RS10340	SEQMU2_RS02200	BK815_RS12185	A6V26_RS07160	AA913_RS00860	SXYL_RS02825	BE24_RS08950	SSP_RS02615
amino acid:proton symporter	-	SCA_RS09815	-	-	BK815_RS10770	A6V26_RS08595	AA913_RS10020	SXYL_RS01155	BE24_RS10565	SSP_RS01065
MFS transporter	-	SCA_RS09935	-	-	BK815_RS11575	A6V26_RS07765	AA913_RS01470	SXYL_RS02100	BE24_RS09685	SSP_RS01960
beta-galactosidase	-	SCA_RS09960	SE1039_RS12745	SEQMU2_RS04620	BK815_RS11550	A6V26_RS07790	AA913_RS01495	SXYL_RS00410	BE24_RS11300	SSP_RS00525
multidrug ABC transporter ATP-binding protein	-	SCA_RS10000	SE1039_RS11065	SEQMU2_RS02945	-	-	-	SXYL_RS02020	BE24_RS09765	SSP_RS01880
lysophospholipase	-	SCA_RS10045	SE1039_RS03230	SEQMU2_RS08440	-	-	-	SXYL_RS10015	BE24_RS01710	SSP_RS09495
pyruvate formate-lyase 1-activating enzyme	-	SCA_RS10205	-	-	-	-	-	SXYL_RS05040	-	-
allophanate hydrolase	-	SCA_RS10320	SE1039_RS07150	SEQMU2_RS12260	BK815_RS02340	A6V26_RS10040	AA913_RS05185	SXYL_RS06175	BE24_RS05465	SSP_RS05875
PepSY domain-containing protein	-	SCA_RS10335	-	-	BK815_RS08475	A6V26_RS04310	AA913_RS06730	SXYL_RS12455	BE24_RS12735	SSP_RS11780
frataxin; iron chaperone	-	SCA_RS10340	SE1039_RS01035	SEQMU2_RS06620	BK815_RS08255	A6V26_RS04095	AA913_RS06515	SXYL_RS12130	BE24_RS13140	SSP_RS11590
MFS transporter	-	SCA_RS10345	-	-	-	-	-	SXYL_RS00435	-	-
carbamate kinase	-	SCA_RS10355	-	-	BK815_RS08290	A6V26_RS04125	AA913_RS06545	SXYL_RS12190	BE24_RS13080	-
hypothetical protein	-	SCA_RS10360	-	-	BK815_RS08295	A6V26_RS04130	AA913_RS06550	SXYL_RS12195	BE24_RS13075	-
hypothetical protein	-	SCA_RS10365	-	-	BK815_RS08300	A6V26_RS04135	AA913_RS06555	SXYL_RS12200	BE24_RS13070	-
acyl-CoA synthetase FdrA	-	SCA_RS10370	-	-	BK815_RS08305	A6V26_RS04140	AA913_RS06560	SXYL_RS12205	BE24_RS13065	-
ureidoglycolate dehydrogenase	-	SCA_RS10375	-	-	BK815_RS08310	A6V26_RS04145	AA913_RS06565	SXYL_RS12210	BE24_RS13060	-
ureidoglycolate dehydrogenase	-	SCA_RS10380	-	-	BK815_RS08315	A6V26_RS04150	AA913_RS06570	SXYL_RS12215	BE24_RS13055	-
(S)-ureidoglycine aminohydrolase	-	SCA_RS10385	-	-	BK815_RS08320	A6V26_RS04155	AA913_RS06575	SXYL_RS12220	BE24_RS13050	-
Zn-dependent hydrolase	-	SCA_RS10390	-	-	BK815_RS08325	A6V26_RS04160	AA913_RS06580	SXYL_RS12225	BE24_RS13045	-
N-acetylglucosamine-6-phosphate deacetylase	-	SCA_RS10440	SE1039_RS02680	SEQMU2_RS07470	BK815_RS06815	A6V26_RS02940	AA913_RS07990	SXYL_RS10505	BE24_RS01230	SSP_RS10095
3-methyl-2-oxobutanoate hydroxymethyltransferase	-	SCA_RS10460	SE1039_RS12205	SEQMU2_RS04065	BK815_RS10425	A6V26_RS06265	AA913_RS12660	SXYL_RS00820	BE24_RS10880	SSP_RS00755
aspartate 1-decarboxylase	-	SCA_RS10470	-	-	BK815_RS01160	A6V26_RS11515	AA913_RS12060	SXYL_RS04940	BE24_RS06615	-
thiamine/molybdopterin biosynthesis protein	-	SCA_RS10500	SE1039_RS12280	SEQMU2_RS04140	BK815_RS10235	A6V26_RS06075	AA913_RS12755	SXYL_RS00715	BE24_RS10965	-

Product	*S. carnosus*		*S. equorum*		*S. succinus*			*S. xylosus*		*S. saprophyticus*
	**JCM 6069**	**TM300**	**KS1039**	**Mu2**	**14BME20**	**CSM 77**	**DSM 14617**	**C2a**	**HKUOPL8**	**ATCC 15305**
teichoic acid biosynthesis protein	-	SCA_RS10630	SE1039_RS13270	SEQMU2_RS04945	BK815_RS03785	-	-	-	-	-
glycosyl transferase family 1	-	SCA_RS10645	SE1039_RS13245	-	BK815_RS03810	-	-	-	-	-
ABC transporter ATP-binding protein	-	SCA_RS10680	SE1039_RS00485	SEQMU2_RS05990	-	A6V26_RS02165	AA913_RS08885	-	-	-
hypothetical protein	-	SCA_RS10750	SE1039_RS10980	SEQMU2_RS02860	-	-	-	SXYL_RS02125	BE24_RS09660	SSP_RS01980
acetamidase/formamidase family protein	-	SCA_RS10805	SE1039_RS00645	SEQMU2_RS06155	-	-	-	-	-	-
CDP-glycerol:glycerophosphate glycerophosphotransferase	-	SCA_RS12715	SE1039_RS13610	SEQMU2_RS03760	BK815_RS10745	A6V26_RS08620	AA913_RS14340	SXYL_RS13255	BE24_RS13640	SSP_RS12655
phage infection protein	-	SCA_RS10985	SE1039_RS10450	SEQMU2_RS02310	BK815_RS12065	A6V26_RS07275	AA913_RS00975	SXYL_RS02720	BE24_RS09055	-
adenosylmethionine--8-amino-7-oxononanoate aminotransferase BioA	-	SCA_RS11215	-	-	BK815_RS08670	A6V26_RS04505	AA913_RS06925	-	-	-
rhodanese domain-containing protein	-	SCA_RS11595	SE1039_RS12510	SEQMU2_RS04305	BK815_RS10110	A6V26_RS05950	AA913_RS12515	SXYL_RS00585	BE24_RS11105	SSP_RS00625
sugar transferase	-	SCA_RS11795	-	SEQMU2_RS05350	-	-	-	-	BE24_RS11765	SSP_RS00360
nucleotide sugar dehydrogenase	-	SCA_RS11815	-	SEQMU2_RS05360	-	-	-	-	BE24_RS11775	-
NAD-dependent epimerase	-	SCA_RS11820	-	SEQMU2_RS05375	-	-	-	-	BE24_RS11780	-
capsular biosynthesis protein	-	SCA_RS11845	-	SEQMU2_RS05395	-	-	-	-	BE24_RS11820	SSP_RS00315
capsular biosynthesis protein	-	SCA_RS11850	-	SEQMU2_RS05400	-	-	-	-	BE24_RS11825	SSP_RS00310
capsule biosynthesis protein CapA	-	SCA_RS11855	-	SEQMU2_RS05405	-	-	-	-	BE24_RS11830	SSP_RS00305
2-dehydropantoate 2-reductase	-	SCA_RS11920	-	SEQMU2_RS04765	-	A6V26_RS05795	AA913_RS10430	-	-	-
LysR family transcriptional regulator	-	SCA_RS11925	-	SEQMU2_RS04760	-	A6V26_RS05800	AA913_RS10425	-	-	-
alcohol dehydrogenase	-	SCA_RS11930	-	-	-	-	-	SXYL_RS01250	-	SSP_RS01155
MBL fold metallo-hydrolase	-	SCA_RS11935	-	-	-	-	-	SXYL_RS01245	-	SSP_RS01150
lipase	-	SCA_RS12045	-	-	BK815_RS08240	-	-	-	BE24_RS13155	-
LysE family translocator	-	SCA_RS12070	SE1039_RS00780	SEQMU2_RS06305	BK815_RS08480	A6V26_RS04315	AA913_RS06735	SXYL_RS12460	BE24_RS12730	SSP_RS11785
RpiB/LacA/LacB family sugar-phosphate isomerase	-	SCA_RS12125	SE1039_RS10405	SEQMU2_RS02265	BK815_RS12115	A6V26_RS07230	AA913_RS00930	SXYL_RS02770	BE24_RS09005	SSP_RS02555
dihydroxyacetone kinase subunit L	-	SCA_RS12130	SE1039_RS10410	SEQMU2_RS02270	BK815_RS12110	A6V26_RS07235	AA913_RS00935	SXYL_RS02765	BE24_RS09010	SSP_RS02550
dihydroxyacetone kinase	-	SCA_RS12135	SE1039_RS10415	SEQMU2_RS02275	BK815_RS12105	A6V26_RS07240	AA913_RS00940	SXYL_RS02760	BE24_RS09015	SSP_RS02545
iditol 2-dehydrogenase	-	SCA_RS12145	SE1039_RS10420	SEQMU2_RS02280	BK815_RS12100	A6V26_RS07245	AA913_RS00945	SXYL_RS02755	BE24_RS09020	SSP_RS02540
DeoR/GlpR transcriptional regulator	-	SCA_RS12150	SE1039_RS10425	SEQMU2_RS02285	BK815_RS12095	A6V26_RS07250	AA913_RS00950	SXYL_RS02750	BE24_RS09025	SSP_RS02535
ribose transporter RbsU	-	SCA_RS12165	SE1039_RS12485	SEQMU2_RS04280	-	-	-	-	-	-
transcriptional regulator	-	-	SE1039_RS00040	SEQMU2_RS05635	-	-	-	SXYL_RS13135	BE24_RS12090	SSP_RS12235
galactose:cation symporter	-	-	SE1039_RS00045	SEQMU2_RS05640	BK815_RS09655	A6V26_RS05540	AA913_RS10690	SXYL_RS00125	-	-
hypothetical protein	-	-	SE1039_RS00050	SEQMU2_RS05645	BK815_RS09660	A6V26_RS05545	AA913_RS10685	SXYL_RS00130	-	-
glycosyl transferase family 1	-	-	SE1039_RS00055	SEQMU2_RS05650	BK815_RS01245	A6V26_RS11430	AA913_RS12145	-	BE24_RS00190	-

**Product**	***S. carnosus***		***S. equorum***		***S. succinus***			***S. xylosus***		***S. saprophyticus***
	**JCM 6069**	**TM300**	**KS1039**	**Mu2**	**14BME20**	**CSM 77**	**DSM 14617**	**C2a**	**HKUOPL8**	**ATCC 15305**
hypothetical protein	-	-	SE1039_RS00070	SEQMU2_RS05665	BK815_RS08905	A6V26_RS04740	AA913_RS07160	SXYL_RS13120	BE24_RS12125	SSP_RS12215
amino acid permease	-	-	SE1039_RS00080	SEQMU2_RS05675	BK815_RS08890	A6V26_RS04725	AA913_RS07145	SXYL_RS13105	BE24_RS12140	SSP_RS12205
hypothetical protein	-	-	SE1039_RS00085	SEQMU2_RS05680	-	-	-	-	-	-
hypothetical protein	-	-	SE1039_RS00090	SEQMU2_RS05685	-	-	-	-	-	-
hypothetical protein	-	-	SE1039_RS00095	SEQMU2_RS05690	BK815_RS03840	-	-	-	-	-
hypothetical protein	-	-	SE1039_RS00100	SEQMU2_RS05695	-	-	-	-	-	-
hypothetical protein	-	-	SE1039_RS00105	SEQMU2_RS13800	-	-	-	-	-	-
hydrolase	-	-	SE1039_RS00125	SEQMU2_RS05745	-	-	-	SXYL_RS13050	BE24_RS12205	SSP_RS12200
MFS transporter	-	-	SE1039_RS00130	SEQMU2_RS05750	BK815_RS08865	A6V26_RS04700	AA913_RS07120	SXYL_RS13045	BE24_RS12210	-
phenolic acid decarboxylase	-	-	SE1039_RS00140	-	BK815_RS10305	A6V26_RS06145	AA913_RS12825	SXYL_RS00640	BE24_RS11050	-
PadR family transcriptional regulator	-	-	SE1039_RS00145	-	-	-	-	SXYL_RS00635	BE24_RS11055	-
flavodoxin family protein	-	-	SE1039_RS00150	SEQMU2_RS05755	-	-	-	-	-	-
peroxiredoxin	-	-	SE1039_RS00155	SEQMU2_RS05760	-	-	-	-	BE24_RS12100	-
hypothetical protein	-	-	SE1039_RS00160	SEQMU2_RS05765	-	-	-	SXYL_RS13130	BE24_RS12105	-
alcohol dehydrogenase	-	-	SE1039_RS00165	SEQMU2_RS14055	-	A6V26_RS13365	AA913_RS13135	-	-	SSP_RS08190
hypothetical protein	-	-	SE1039_RS00200	-	-	-	-	-	-	SSP_RS11890
NAD(P)H-dependent oxidoreductase	-	-	SE1039_RS00210	-	BK815_RS10295	A6V26_RS06135	AA913_RS12815	-	-	-
citrate transporter	-	-	SE1039_RS00215	-	-	-	-	SXYL_RS02355	BE24_RS09430	-
hypothetical protein	-	-	SE1039_RS00245	SEQMU2_RS14040	-	-	-	-	-	-
teichoic acid ABC transporter permease	-	-	SE1039_RS00250	-	BK815_RS01240	A6V26_RS11435	AA913_RS12140	SXYL_RS05035	-	SSP_RS10970
EamA-like transporter family protein	-	-	SE1039_RS00255	-	BK815_RS08715	A6V26_RS04550	AA913_RS06970	SXYL_RS07410	-	-
EamA-like transporter family protein	-	-	SE1039_RS00260	-	BK815_RS08710	A6V26_RS04545	AA913_RS06965	SXYL_RS07415	-	-
ArsR family transcriptional regulator	-	-	SE1039_RS00265	-	BK815_RS08705	A6V26_RS04540	AA913_RS06960	SXYL_RS07420	-	-
hypothetical protein	-	-	SE1039_RS00270	SEQMU2_RS05775	-	-	-	-	-	-
hypothetical protein	-	-	SE1039_RS00275	SEQMU2_RS05780	BK815_RS09340	A6V26_RS05160	AA913_RS02015	SXYL_RS11180	BE24_RS00590	-
hypothetical protein	-	-	SE1039_RS00280	SEQMU2_RS05785	-	-	-	-	-	-
LysR family transcriptional regulator	-	-	SE1039_RS00285	SEQMU2_RS05790	BK815_RS09235	-	-	SXYL_RS12535	BE24_RS11560	-
tricarballylate dehydrogenase	-	-	SE1039_RS00290	SEQMU2_RS05795	BK815_RS09230	-	-	-	BE24_RS11555	-
tripartite tricarboxylate transporter substrate binding protein	-	-	SE1039_RS00295	SEQMU2_RS05800	-	-	-	-	BE24_RS11550	-
universal stress protein	-	-	SE1039_RS00310	SEQMU2_RS05815	BK815_RS09315	A6V26_RS05120	AA913_RS01975	-	-	-
hypothetical protein	-	-	SE1039_RS00340	SEQMU2_RS05845	-	-	-	SXYL_RS12950	BE24_RS12280	SSP_RS12145

Product	S. carnosus		S. equorum		S. succinus			S. xylosus		S. saprophyticus
	JCM 6069	TM300	KS1039	Mu2	14BME20	CSM 77	DSM 14617	C2a	HKUOPL8	ATCC 15305
sulfite reductase subunit alpha	-	-	SE1039_RS00345	SEQMU2_RS05850	BK815_RS08825	A6V26_RS04660	AA913_RS07080	SXYL_RS12945	BE24_RS12285	SSP_RS12140
DNA-binding protein	-	-	SE1039_RS00355	-	BK815_RS10020	A6V26_RS05860	AA913_RS10365	SXYL_RS12955	BE24_RS12275	-
cystathionine gamma-synthase	-	-	SE1039_RS00380	SEQMU2_RS05885	BK815_RS08800	A6V26_RS04635	AA913_RS07055	SXYL_RS12920	BE24_RS12310	SSP_RS12115
hypothetical protein	-	-	SE1039_RS00390	-	-	A6V26_RS05085	AA913_RS01940	-	-	-
sirohydrochlorin ferrochelatase	-	-	SE1039_RS00420	SEQMU2_RS05925	BK815_RS08765	A6V26_RS04600	AA913_RS07020	SXYL_RS12885	BE24_RS12340	SSP_RS12075
precorrin-2 dehydrogenase	-	-	SE1039_RS00425	SEQMU2_RS05930	BK815_RS08760	A6V26_RS04595	AA913_RS07015	SXYL_RS12880	BE24_RS12345	SSP_RS12070
hypothetical protein	-	-	SE1039_RS00445	SEQMU2_RS05950	BK815_RS08740	A6V26_RS04575	AA913_RS06995	SXYL_RS12860	BE24_RS12365	SSP_RS12045
adenosine deaminase	-	-	SE1039_RS00450	SEQMU2_RS05955	BK815_RS08735	A6V26_RS04570	AA913_RS06990	SXYL_RS12855	BE24_RS12370	SSP_RS12040
phospholipid phosphatase	-	-	SE1039_RS00465	SEQMU2_RS05970	BK815_RS08725	A6V26_RS04560	AA913_RS06980	SXYL_RS12840	BE24_RS12385	SSP_RS12030
hypothetical protein	-	-	SE1039_RS00470	SEQMU2_RS05975	-	-	-	SXYL_RS12835	BE24_RS12390	-
hypothetical protein	-	-	SE1039_RS00475	SEQMU2_RS05980	-	-	-	-	-	-
general stress protein	-	-	SE1039_RS00480	SEQMU2_RS05985	-	-	-	-	-	-
ferrochelatase	-	-	SE1039_RS00495	SEQMU2_RS06000	-	-	-	-	-	-
catalase	-	-	SE1039_RS00500	SEQMU2_RS06005	-	-	-	SXYL_RS12275	BE24_RS12990	-
3-alpha-hydroxysteroid dehydrogenase	-	-	SE1039_RS00510	SEQMU2_RS06015	-	-	-	-	-	-
MarR family transcriptional regulator	-	-	SE1039_RS00515	SEQMU2_RS06020	-	-	-	-	-	-
oxidoreductase	-	-	SE1039_RS00520	SEQMU2_RS06025	-	-	-	-	-	-
hypothetical protein	-	-	SE1039_RS00525	SEQMU2_RS06030	BK815_RS10095	A6V26_RS05935	AA913_RS12500	SXYL_RS12820	BE24_RS12405	-
hypothetical protein	-	-	SE1039_RS00535	SEQMU2_RS06040	-	-	-	-	-	-
hypothetical protein	-	-	SE1039_RS00540	SEQMU2_RS06045	-	-	-	-	-	-
hypothetical protein	-	-	SE1039_RS00545	SEQMU2_RS06050	-	-	-	-	-	-
hypothetical protein	-	-	SE1039_RS00550	SEQMU2_RS06055	-	-	-	-	-	-
ABC transporter substrate-binding protein	-	-	SE1039_RS00555	SEQMU2_RS06060	-	-	-	-	-	-
iron ABC transporter permease	-	-	SE1039_RS00560	SEQMU2_RS06065	-	-	-	-	-	-
ABC transporter ATP-binding protein	-	-	SE1039_RS00565	SEQMU2_RS06070	-	-	-	-	-	-
amidohydrolase	-	-	SE1039_RS00580	SEQMU2_RS06085	BK815_RS08630	A6V26_RS04465	AA913_RS06885	SXYL_RS12805	BE24_RS12420	-
MerR family transcriptional regulator	-	-	SE1039_RS00605	SEQMU2_RS06110	BK815_RS09190	A6V26_RS05015	AA913_RS01870	SXYL_RS02140	BE24_RS09645	-
hypothetical protein	-	-	SE1039_RS00610	SEQMU2_RS06115	-	-	-	-	-	-
NINE protein	-	-	SE1039_RS00615	SEQMU2_RS06120	-	-	-	-	-	-
4-phosphopantetheinyl transferase	-	-	SE1039_RS00620	SEQMU2_RS06125	-	-	-	-	-	-
non-ribosomal peptide synthetase	-	-	SE1039_RS00625	SEQMU2_RS06130	-	-	-	-	BE24_RS12595	-

Product	*S. carnosus*		*S. equorum*		*S. succinus*			*S. xylosus*		*S. saprophyticus*
	**JCM 6069**	**TM300**	**KS1039**	**Mu2**	**14BME20**	**CSM 77**	**DSM 14617**	**C2a**	**HKUOPL8**	**ATCC 15305**
peptide synthetase	-	-	SE1039_RS00630	SEQMU2_RS06140	BK815_RS10055	A6V26_RS05895	AA913_RS10330	-	-	-
NADH dehydrogenase	-	-	SE1039_RS00650	SEQMU2_RS06160	BK815_RS08580	A6V26_RS04415	AA913_RS06835	SXYL_RS12620	BE24_RS12550	SSP_RS11960
hypothetical protein	-	-	SE1039_RS00655	SEQMU2_RS06165	BK815_RS08575	A6V26_RS04410	AA913_RS06830	SXYL_RS12615	BE24_RS12555	SSP_RS11955
hypothetical protein	-	-	SE1039_RS00660	SEQMU2_RS06170	BK815_RS08570	A6V26_RS04405	AA913_RS06825	SXYL_RS12610	BE24_RS12560	SSP_RS11950
lysozyme	-	-	SE1039_RS00680	SEQMU2_RS06190	BK815_RS08550	A6V26_RS04385	AA913_RS06805	SXYL_RS12590	BE24_RS12575	SSP_RS11925
hypothetical protein	-	-	SE1039_RS00700	SEQMU2_RS06225	-	-	-	SXYL_RS12550	BE24_RS12645	SSP_RS11865
amidase	-	-	SE1039_RS00705	SEQMU2_RS06230	-	-	-	SXYL_RS12545	BE24_RS12650	SSP_RS11860
hypothetical protein	-	-	SE1039_RS00715	SEQMU2_RS06240	BK815_RS03900	A6V26_RS00045	AA913_RS03890	-	BE24_RS13565	-
PTS sugar transporter subunit IIB	-	-	SE1039_RS00720	SEQMU2_RS06245	-	-	-	-	-	-
PTS system, cellobiose-specific IIC component	-	-	SE1039_RS00725	SEQMU2_RS06250	-	-	-	-	-	-
PTS lactose/cellobiose transporter subunit IIA	-	-	SE1039_RS00730	SEQMU2_RS06255	-	-	-	-	-	-
6-phospho-beta-glucosidase	-	-	SE1039_RS00735	SEQMU2_RS06260	-	-	-	-	-	-
hypothetical protein	-	-	SE1039_RS00765	SEQMU2_RS06290	BK815_RS08495	A6V26_RS04330	AA913_RS06750	SXYL_RS12475	BE24_RS12715	SSP_RS11805
multidrug resistance protein SMR	-	-	SE1039_RS00770	SEQMU2_RS06295	BK815_RS08490	A6V26_RS04325	AA913_RS06745	SXYL_RS12470	BE24_RS12720	-
QacE family quaternary ammonium compound efflux SMR transporter	-	-	SE1039_RS00775	SEQMU2_RS06300	BK815_RS08485	A6V26_RS04320	AA913_RS06740	SXYL_RS12465	BE24_RS12725	-
hypothetical protein	-	-	SE1039_RS00785	SEQMU2_RS06310	BK815_RS08465	A6V26_RS04300	AA913_RS06720	SXYL_RS12445	BE24_RS12745	SSP_RS11770
hypothetical protein	-	-	SE1039_RS00795	SEQMU2_RS06320	BK815_RS08455	A6V26_RS04290	AA913_RS06710	SXYL_RS12435	BE24_RS12755	SSP_RS11760
hypothetical protein	-	-	SE1039_RS00815	SEQMU2_RS06340	-	-	-	-	-	-
ABC transporter ATP-binding protein	-	-	SE1039_RS00820	SEQMU2_RS06345	-	-	-	-	-	-
ABC transporter ATP-binding protein	-	-	SE1039_RS00825	SEQMU2_RS06350	-	-	-	-	-	-
transcriptional regulator	-	-	SE1039_RS00830	SEQMU2_RS06355	-	-	-	-	-	SSP_RS11740
hypothetical protein	-	-	SE1039_RS00850	SEQMU2_RS06375	BK815_RS08425	A6V26_RS04260	AA913_RS06680	SXYL_RS12405	BE24_RS12785	SSP_RS11730
hypothetical protein	-	-	SE1039_RS00860	SEQMU2_RS06385	-	-	-	-	-	-
hypothetical protein	-	-	SE1039_RS00910	SEQMU2_RS14225	-	A6V26_RS00090	AA913_RS03845	-	-	SSP_RS11680
hypothetical protein	-	-	SE1039_RS00940	SEQMU2_RS06525	-	-	-	-	-	SSP_RS11905
hypothetical protein	-	-	SE1039_RS00945	SEQMU2_RS06530	-	-	-	-	-	SSP_RS11900
hypothetical protein	-	-	SE1039_RS00950	SEQMU2_RS06535	-	-	-	-	-	SSP_RS11670
hypothetical protein	-	-	SE1039_RS00955	SEQMU2_RS06540	-	-	-	SXYL_RS12330	-	-
hypothetical protein	-	-	SE1039_RS00960	SEQMU2_RS06545	BK815_RS03835	-	-	SXYL_RS12325	-	-
phosphate-starvation-inducible protein PsiE	-	-	SE1039_RS00965	SEQMU2_RS06550	-	-	-	-	BE24_RS01885	-
cold-shock protein	-	-	SE1039_RS00970	SEQMU2_RS06555	BK815_RS08380	A6V26_RS04215	AA913_RS06635	SXYL_RS12320	BE24_RS12955	SSP_RS11640

Product	*S. carnosus*		*S. equorum*		*S. succinus*			*S. xylosus*		*S. saprophyticus*
	**JCM 6069**	**TM300**	**KS1039**	**Mu2**	**14BME20**	**CSM 77**	**DSM 14617**	**C2a**	**HKUOPL8**	**ATCC 15305**
MFS transporter	-	-	SE1039_RS00975	SEQMU2_RS06560	BK815_RS08375	A6V26_RS04210	AA913_RS06630	SXYL_RS12315	-	SSP_RS11635
LysR family transcriptional regulator	-	-	SE1039_RS00980	SEQMU2_RS06565	BK815_RS08370	A6V26_RS04205	AA913_RS06625	SXYL_RS12310	-	SSP_RS00975
hypothetical protein	-	-	SE1039_RS00985	SEQMU2_RS06570	-	-	-	-	-	-
RNA polymerase sigma factor	-	-	SE1039_RS00990	SEQMU2_RS06575	BK815_RS09170	A6V26_RS04995	AA913_RS10290	SXYL_RS12250	BE24_RS13020	-
hypothetical protein	-	-	SE1039_RS00995	SEQMU2_RS06580	BK815_RS09165	A6V26_RS04990	AA913_RS10285	SXYL_RS12255	BE24_RS13015	-
UDP-N-acetylmuramoylalanyl-D-glutamate--2, 6-diaminopimelate ligase	-	-	SE1039_RS01000	SEQMU2_RS06585	BK815_RS09160	A6V26_RS04985	AA913_RS10280	SXYL_RS12260	BE24_RS13010	-
hypothetical protein	-	-	SE1039_RS01005	SEQMU2_RS06590	BK815_RS09155	A6V26_RS04980	AA913_RS10275	SXYL_RS12265	-	-
histidine phosphatase family protein	-	-	SE1039_RS01010	SEQMU2_RS06595	BK815_RS08340	A6V26_RS04175	AA913_RS06595	SXYL_RS12240	BE24_RS13030	-
MFS transporter	-	-	SE1039_RS01015	SEQMU2_RS06600	BK815_RS08285	A6V26_RS04120	AA913_RS06540	SXYL_RS12185	BE24_RS13085	-
hypothetical protein	-	-	SE1039_RS01020	SEQMU2_RS06605	BK815_RS08270	A6V26_RS04110	AA913_RS06530	SXYL_RS12175	BE24_RS13095	SSP_RS11610
hypothetical protein	-	-	SE1039_RS01025	SEQMU2_RS06610	BK815_RS08265	A6V26_RS04105	AA913_RS06525	SXYL_RS12170	BE24_RS13100	-
dihydroxy-acid dehydratase	-	-	SE1039_RS01050	SEQMU2_RS06635	BK815_RS08235	A6V26_RS04080	AA913_RS06500	SXYL_RS12110	BE24_RS13165	SSP_RS11560
hypothetical protein	-	-	SE1039_RS01070	SEQMU2_RS06655	-	A6V26_RS04065	AA913_RS06485	SXYL_RS12090	BE24_RS13185	SSP_RS11540
ammonia permease	-	-	SE1039_RS01075	SEQMU2_RS06660	BK815_RS08215	A6V26_RS04060	AA913_RS06480	SXYL_RS12085	BE24_RS13190	SSP_RS11535
trehalose permease IIC protein	-	-	SE1039_RS01110	SEQMU2_RS06695	BK815_RS08180	A6V26_RS04025	AA913_RS06445	SXYL_RS12050	BE24_RS13225	SSP_RS11500
sigma-70 family RNA polymerase sigma factor	-	-	SE1039_RS01525	SEQMU2_RS00085	BK815_RS07775	A6V26_RS03890	AA913_RS10880	SXYL_RS11655	BE24_RS00085	SSP_RS11105
hypothetical protein	-	-	SE1039_RS01625	SEQMU2_RS00170	BK815_RS07680	A6V26_RS03800	AA913_RS10970	SXYL_RS11560	BE24_RS00175	SSP_RS11010
succinyl-CoA--3-ketoacid-CoA transferase	-	-	SE1039_RS01645	SEQMU2_RS00190	BK815_RS07665	A6V26_RS03785	AA913_RS10985	SXYL_RS11535	BE24_RS00200	SSP_RS10995
succinyl-CoA--3-ketoacid-CoA transferase	-	-	SE1039_RS01650	SEQMU2_RS00195	BK815_RS07660	A6V26_RS03780	AA913_RS10990	SXYL_RS11530	BE24_RS00205	SSP_RS10990
3-hydroxybutyrate dehydrogenase	-	-	SE1039_RS01655	SEQMU2_RS00200	BK815_RS07655	A6V26_RS03775	AA913_RS10995	SXYL_RS11525	BE24_RS00210	SSP_RS10985
hypothetical protein	-	-	SE1039_RS01660	-	-	-	-	SXYL_RS11520	BE24_RS00215	SSP_RS10980
ornithine cyclodeaminase	-	-	SE1039_RS01665	-	BK815_RS07650	A6V26_RS03770	AA913_RS11000	SXYL_RS11515	BE24_RS00220	SSP_RS10975
hypothetical protein	-	-	SE1039_RS01680	-	-	-	-	-	-	SSP_RS00545
accessory Sec system glycosyltransferase GtfA	-	-	SE1039_RS01685	-	BK815_RS10370	A6V26_RS06210	AA913_RS12715	-	-	SSP_RS00550
accessory Sec system glycosylation chaperone GtfB	-	-	SE1039_RS01690	-	-	-	-	-	-	SSP_RS00555
sialate O-acetylesterase	-	-	SE1039_RS01695	SEQMU2_RS00205	BK815_RS07640	A6V26_RS03760	AA913_RS11010	SXYL_RS11500	BE24_RS00245	SSP_RS10960
monooxygenase	-	-	SE1039_RS01700	SEQMU2_RS00210	BK815_RS07635	A6V26_RS03755	AA913_RS11015	SXYL_RS11495	BE24_RS00250	SSP_RS10950
hypothetical protein	-	-	SE1039_RS01705	SEQMU2_RS00215	BK815_RS09365	A6V26_RS05225	AA913_RS02080	SXYL_RS11480	BE24_RS00290	SSP_RS10935
class A beta-lactamase	-	-	SE1039_RS01710	SEQMU2_RS00220	BK815_RS07620	A6V26_RS03745	AA913_RS11025	SXYL_RS11475	BE24_RS00295	SSP_RS10925
LysR family transcriptional regulator	-	-	SE1039_RS01715	SEQMU2_RS00225	BK815_RS07615	A6V26_RS03740	AA913_RS11030	SXYL_RS11470	BE24_RS00300	SSP_RS10920
transcriptional regulator	-	-	SE1039_RS01720	SEQMU2_RS00230	BK815_RS07610	A6V26_RS03735	AA913_RS11035	SXYL_RS11465	BE24_RS00305	SSP_RS10915

Product	*S. carnosus*		*S. equorum*		*S. succinus*			*S. xylosus*		*S. saprophyticus*
	**JCM 6069**	**TM300**	**KS1039**	**Mu2**	**14BME20**	**CSM 77**	**DSM 14617**	**C2a**	**HKUOPL8**	**ATCC 15305**
2-dehydro-3-deoxy-phosphogluconate aldolase	-	-	SE1039_RS01725	SEQMU2_RS00235	BK815_RS07605	A6V26_RS03730	AA913_RS11040	SXYL_RS11460	BE24_RS00310	-
sugar kinase	-	-	SE1039_RS01730	SEQMU2_RS00240	BK815_RS07600	A6V26_RS03725	AA913_RS11045	SXYL_RS11455	BE24_RS00315	-
TRAP transporter substrate-binding protein DctP	-	-	SE1039_RS01735	SEQMU2_RS00245	-	-	-	SXYL_RS11450	BE24_RS00320	-
TRAP transporter small permease	-	-	SE1039_RS01740	SEQMU2_RS00250	-	-	-	SXYL_RS11445	BE24_RS00325	-
membrane protein	-	-	SE1039_RS01745	SEQMU2_RS00255	-	-	-	SXYL_RS11440	BE24_RS00330	-
uronate isomerase	-	-	SE1039_RS01750	SEQMU2_RS00260	BK815_RS07595	A6V26_RS03720	AA913_RS11050	SXYL_RS11435	BE24_RS09580	-
mannonate dehydratase	-	-	SE1039_RS01755	SEQMU2_RS00265	BK815_RS07590	A6V26_RS03715	AA913_RS11055	SXYL_RS11430	BE24_RS00340	-
D-mannonate oxidoreductase	-	-	SE1039_RS01760	SEQMU2_RS00270	BK815_RS07585	A6V26_RS03710	AA913_RS11060	SXYL_RS11425	BE24_RS00345	-
ABC transporter ATP-binding protein	-	-	SE1039_RS01765	SEQMU2_RS05085	-	-	-	SXYL_RS11420	BE24_RS00350	-
ribulokinase	-	-	SE1039_RS01770	SEQMU2_RS05090	BK815_RS07580	A6V26_RS03705	AA913_RS11065	SXYL_RS11400	BE24_RS00370	SSP_RS10885
hypothetical protein	-	-	SE1039_RS01775	SEQMU2_RS05095	-	-	-	-	-	-
divalent metal cation transporter	-	-	SE1039_RS01780	SEQMU2_RS05100	BK815_RS07575	A6V26_RS03700	AA913_RS11070	SXYL_RS11385	BE24_RS00385	SSP_RS10875
agmatinase	-	-	SE1039_RS01785	SEQMU2_RS05105	BK815_RS07570	A6V26_RS03695	AA913_RS11075	SXYL_RS11380	BE24_RS00390	SSP_RS10870
glutamate dehydrogenase	-	-	SE1039_RS01790	SEQMU2_RS05110	BK815_RS07565	A6V26_RS03690	AA913_RS11080	SXYL_RS11375	BE24_RS00395	SSP_RS10865
amino acid permease	-	-	SE1039_RS01795	SEQMU2_RS05115	BK815_RS07560	A6V26_RS03685	AA913_RS11085	SXYL_RS11370	BE24_RS00400	SSP_RS10860
homocysteine S-methyltransferase	-	-	SE1039_RS01800	SEQMU2_RS05120	BK815_RS07555	A6V26_RS03680	AA913_RS11090	SXYL_RS11365	BE24_RS00405	SSP_RS10855
fatty acid desaturase	-	-	SE1039_RS01805	SEQMU2_RS05125	BK815_RS07550	A6V26_RS03675	AA913_RS11095	SXYL_RS11355	BE24_RS00415	-
hypothetical protein	-	-	SE1039_RS01810	SEQMU2_RS05130	BK815_RS07545	A6V26_RS03670	AA913_RS11100	SXYL_RS11345	BE24_RS00425	SSP_RS10845
hypothetical protein	-	-	SE1039_RS01815	SEQMU2_RS05135	-	-	-	-	-	-
TetR family transcriptional regulator	-	-	SE1039_RS01830	-	-	A6V26_RS02160	AA913_RS08890	-	-	-
ABC transporter ATP-binding protein	-	-	SE1039_RS01835	-	-	A6V26_RS02165	AA913_RS08885	-	-	-
hypothetical protein	-	-	SE1039_RS01840	-	-	A6V26_RS02170	AA913_RS08880	-	-	-
Lrp/AsnC family transcriptional regulator	-	-	SE1039_RS01865	-	BK815_RS02075	A6V26_RS10295	AA913_RS05435	-	BE24_RS00275	-
MBL fold metallo-hydrolase	-	-	SE1039_RS01870	SEQMU2_RS06740	BK815_RS02080	A6V26_RS10290	AA913_RS05430	-	BE24_RS00280	-
MFS transporter	-	-	SE1039_RS01880	-	BK815_RS02085	A6V26_RS10285	AA913_RS05425	-	BE24_RS11270	-
transferrin-binding protein	-	-	SE1039_RS01885	-	BK815_RS03715	A6V26_RS08980	AA913_RS09665	SXYL_RS07345	BE24_RS04090	SSP_RS06955
hypothetical protein	-	-	SE1039_RS01890	SEQMU2_RS06745	BK815_RS10205	A6V26_RS06045	AA913_RS12610	SXYL_RS00195	BE24_RS11510	SSP_RS00700
hypothetical protein	-	-	SE1039_RS01900	-	-	A6V26_RS05045	AA913_RS01900	-	-	-
GTP pyrophosphokinase	-	-	SE1039_RS01910	-	-	A6V26_RS05035	AA913_RS01890	-	-	-
hypothetical protein	-	-	SE1039_RS01915	-	-	A6V26_RS05030	AA913_RS01885	-	-	-
transcriptional regulator	-	-	SE1039_RS01920	-	BK815_RS03965	A6V26_RS00125	AA913_RS03810	SXYL_RS12705	-	-

Product	S. carnosus		S. equorum		S. succinus			S. xylosus		S. saprophyticus
	JCM 6069	TM300	KS1039	Mu2	14BME20	CSM 77	DSM 14617	C2a	HKUOPL8	ATCC 15305
nitronate monooxygenase	-	-	SE1039_RS01925	-	BK815_RS09350	-	-	SXYL_RS12700	-	-
nitroreductase A	-	-	SE1039_RS01930	-	BK815_RS03960	A6V26_RS00120	AA913_RS03815	-	-	-
3-hydroxyisobutyrate dehydrogenase	-	-	SE1039_RS01935	-	BK815_RS09195	A6V26_RS05165	AA913_RS02020	SXYL_RS12710	-	-
ketoacyl-ACP synthase III	-	-	SE1039_RS01940	-	BK815_RS09385	A6V26_RS05270	AA913_RS02125	SXYL_RS12145	BE24_RS13125	-
hypothetical protein	-	-	SE1039_RS01945	SEQMU2_RS14245	-	-	-	-	BE24_RS12435	-
transposon DNA-invertase	-	-	SE1039_RS01955	SEQMU2_RS14130	-	A6V26_RS12880	AA913_RS04450	-	-	-
transcriptional regulator	-	-	SE1039_RS01960	-	-	A6V26_RS12505	AA913_RS10210	-	-	SSP_RS08115
heavy metal translocating P-type ATPase	-	-	SE1039_RS01965	-	-	A6V26_RS12500	AA913_RS10215	-	-	SSP_RS08110
hypothetical protein	-	-	SE1039_RS01970	SEQMU2_RS06755	-	-	-	SXYL_RS11280	BE24_RS00490	SSP_RS10805
transcriptional regulator	-	-	SE1039_RS02010	SEQMU2_RS06795	BK815_RS07495	A6V26_RS03620	AA913_RS11150	SXYL_RS11235	BE24_RS00535	SSP_RS10765
hypothetical protein	-	-	SE1039_RS02015	SEQMU2_RS06800	BK815_RS07490	A6V26_RS03615	AA913_RS11155	SXYL_RS11230	BE24_RS00540	-
sodium:alanine symporter family protein	-	-	SE1039_RS02045	SEQMU2_RS06830	-	-	-	-	-	-
acyl-CoA synthetase	-	-	SE1039_RS02050	SEQMU2_RS06835	BK815_RS07455	A6V26_RS03580	AA913_RS11190	SXYL_RS11140	BE24_RS00620	SSP_RS10730
hypothetical protein	-	-	SE1039_RS02060	SEQMU2_RS06845	BK815_RS07445	A6V26_RS03570	AA913_RS11200	SXYL_RS11130	BE24_RS00630	SSP_RS10720
hypothetical protein	-	-	SE1039_RS02065	SEQMU2_RS06850	BK815_RS07440	A6V26_RS03565	AA913_RS11205	SXYL_RS11125	BE24_RS00635	SSP_RS10715
hypothetical protein	-	-	SE1039_RS02080	SEQMU2_RS06865	BK815_RS07430	A6V26_RS03555	AA913_RS11220	-	-	SSP_RS10685
PTS alpha-glucoside transporter subunit IIBC	-	-	SE1039_RS02150	SEQMU2_RS06935	BK815_RS07360	A6V26_RS03485	AA913_RS07450	SXYL_RS11040	-	-
MurR/RpiR family transcriptional regulator	-	-	SE1039_RS02155	SEQMU2_RS06940	BK815_RS07355	A6V26_RS03480	AA913_RS07455	SXYL_RS11035	-	-
gfo/Idh/MocA family oxidoreductase	-	-	SE1039_RS02160	SEQMU2_RS06945	BK815_RS07350	A6V26_RS03475	AA913_RS07460	SXYL_RS11030	-	-
glutamine amidotransferase	-	-	SE1039_RS02165	SEQMU2_RS06950	BK815_RS07345	A6V26_RS03470	AA913_RS07465	SXYL_RS11025	-	-
gfo/Idh/MocA family oxidoreductase	-	-	SE1039_RS02170	SEQMU2_RS06955	BK815_RS07340	A6V26_RS03465	AA913_RS07470	SXYL_RS11020	-	-
xylose isomerase	-	-	SE1039_RS02175	SEQMU2_RS06960	BK815_RS07335	A6V26_RS03460	AA913_RS07475	SXYL_RS11015	-	-
aldo/keto reductase	-	-	SE1039_RS02185	SEQMU2_RS06970	BK815_RS07325	A6V26_RS03450	AA913_RS07485	SXYL_RS11005	BE24_RS00725	SSP_RS10600
endonuclease III	-	-	SE1039_RS02220	SEQMU2_RS07005	BK815_RS07290	A6V26_RS03415	AA913_RS07520	SXYL_RS10970	BE24_RS00760	SSP_RS10565
MerR family transcriptional regulator	-	-	SE1039_RS02225	SEQMU2_RS07010	-	-	-	-	-	-
alpha/beta hydrolase	-	-	SE1039_RS02260	SEQMU2_RS07045	BK815_RS07245	A6V26_RS03375	AA913_RS07560	SXYL_RS10935	BE24_RS00795	SSP_RS10530
hypothetical protein	-	-	SE1039_RS02270	-	BK815_RS07235	-	-	SXYL_RS10925	BE24_RS00805	SSP_RS10520
3-beta hydroxysteroid dehydrogenase	-	-	SE1039_RS02465	SEQMU2_RS07250	BK815_RS07040	A6V26_RS03165	AA913_RS07770	SXYL_RS10730	BE24_RS01000	SSP_RS10325
N-acetyltransferase	-	-	SE1039_RS02550	SEQMU2_RS07335	BK815_RS06955	A6V26_RS03080	AA913_RS07855	SXYL_RS10640	BE24_RS01090	SSP_RS10235
hypothetical protein	-	-	SE1039_RS02555	SEQMU2_RS07340	BK815_RS06950	A6V26_RS03075	AA913_RS07860	SXYL_RS10635	BE24_RS01095	SSP_RS10230
hypothetical protein	-	-	SE1039_RS02640	SEQMU2_RS07430	BK815_RS06855	A6V26_RS02980	AA913_RS07950	SXYL_RS10545	BE24_RS01190	SSP_RS10140

Product	*S. carnosus*		*S. equorum*		*S. succinus*			*S. xylosus*		*S. saprophyticus*
	**JCM 6069**	**TM300**	**KS1039**	**Mu2**	**14BME20**	**CSM 77**	**DSM 14617**	**C2a**	**HKUOPL8**	**ATCC 15305**
hypothetical protein	-	-	SE1039_RS02650	SEQMU2_RS07440	BK815_RS06845	A6V26_RS02970	AA913_RS07960	SXYL_RS10535	BE24_RS01200	SSP_RS10130
UTP--glucose-1-phosphate uridylyltransferase	-	-	SE1039_RS02655	SEQMU2_RS07445	BK815_RS06840	A6V26_RS02965	AA913_RS07965	SXYL_RS10530	BE24_RS01205	SSP_RS10120
GlsB/YeaQ/YmgE family stress response membrane protein	-	-	SE1039_RS02700	SEQMU2_RS07490	BK815_RS06795	A6V26_RS02920	AA913_RS08010	SXYL_RS10485	BE24_RS01250	SSP_RS10075
hypothetical protein	-	-	SE1039_RS02740	SEQMU2_RS07530	BK815_RS06755	A6V26_RS02880	AA913_RS08050	SXYL_RS13320	BE24_RS13575	SSP_RS12840
ZIP family metal transporter	-	-	SE1039_RS02760	SEQMU2_RS07550	BK815_RS06735	A6V26_RS02860	AA913_RS08070	SXYL_RS10430	BE24_RS01305	SSP_RS10020
hypothetical protein	-	-	SE1039_RS02855	SEQMU2_RS07645	BK815_RS06640	A6V26_RS02765	AA913_RS08165	SXYL_RS10335	BE24_RS01400	SSP_RS09925
YitT family protein	-	-	SE1039_RS03015	SEQMU2_RS07805	-	-	-	-	-	SSP_RS09765
hypothetical protein	-	-	SE1039_RS03035	SEQMU2_RS08145	BK815_RS06450	A6V26_RS02575	AA913_RS08355	SXYL_RS10155	BE24_RS01580	SSP_RS09615
hypothetical protein	-	-	SE1039_RS03040	SEQMU2_RS08150	BK815_RS06445	A6V26_RS02570	AA913_RS08360	SXYL_RS10150	-	SSP_RS09610
N-acetyltransferase	-	-	SE1039_RS03105	SEQMU2_RS08305	-	-	-	-	-	-
HAD family hydrolase	-	-	SE1039_RS03110	SEQMU2_RS08310	-	-	-	-	-	SSP_RS09535
N-acetyltransferase	-	-	SE1039_RS03115	SEQMU2_RS08315	-	-	-	-	-	-
metallothiol transferase FosB	-	-	SE1039_RS03120	SEQMU2_RS06505	BK815_RS06365	-	-	-	-	SSP_RS09650
N-acetyltransferase	-	-	SE1039_RS03125	SEQMU2_RS08330	-	-	-	-	-	-
cytidine deaminase	-	-	SE1039_RS03130	SEQMU2_RS08335	-	-	-	-	-	-
hypothetical protein	-	-	SE1039_RS03135	SEQMU2_RS08345	BK815_RS02660	A6V26_RS11265	-	-	-	-
hypothetical protein	-	-	SE1039_RS03140	SEQMU2_RS08350	-	-	-	-	-	-
hypothetical protein	-	-	SE1039_RS03145	SEQMU2_RS08355	BK815_RS04795	A6V26_RS00955	AA913_RS02975	SXYL_RS00795	-	SSP_RS00735
CPBP family intramembrane metalloprotease	-	-	SE1039_RS03150	SEQMU2_RS08360	-	-	-	-	-	-
hypothetical protein	-	-	SE1039_RS03155	SEQMU2_RS08365	BK815_RS06325	A6V26_RS02475	AA913_RS08450	-	-	-
hypothetical protein	-	-	SE1039_RS03165	SEQMU2_RS08375	-	-	-	-	-	-
hypothetical protein	-	-	SE1039_RS03170	SEQMU2_RS08380	-	-	-	-	-	SSP_RS04225
hypothetical protein	-	-	SE1039_RS03175	SEQMU2_RS08385	-	-	-	-	-	-
isomerase	-	-	SE1039_RS03185	SEQMU2_RS08395	BK815_RS01270	A6V26_RS11405	AA913_RS12370	-	BE24_RS00605	-
hypothetical protein	-	-	SE1039_RS03190	SEQMU2_RS08400	-	-	-	-	-	-
hypothetical protein	-	-	SE1039_RS03195	SEQMU2_RS08405	-	-	-	SXYL_RS11275	BE24_RS00495	-
cold-shock protein	-	-	SE1039_RS03200	SEQMU2_RS08410	-	-	-	-	-	-
hypothetical protein	-	-	SE1039_RS03210	SEQMU2_RS08420	BK815_RS06290	A6V26_RS02435	AA913_RS08485	SXYL_RS10030	BE24_RS01695	SSP_RS09505
tryptophan-rich sensory protein	-	-	SE1039_RS03225	SEQMU2_RS08435	-	-	-	-	-	-
hypothetical protein	-	-	SE1039_RS03235	SEQMU2_RS08445	BK815_RS06280	A6V26_RS02425	AA913_RS08495	SXYL_RS10010	BE24_RS01715	-
transcriptional regulator	-	-	SE1039_RS03240	SEQMU2_RS08450	BK815_RS09380	A6V26_RS05240	AA913_RS02095	SXYL_RS10005	BE24_RS01720	SSP_RS09490

Product	*S. carnosus*		*S. equorum*		*S. succinus*			*S. xylosus*		*S. saprophyticus*
	**JCM 6069**	**TM300**	**KS1039**	**Mu2**	**14BME20**	**CSM 77**	**DSM 14617**	**C2a**	**HKUOPL8**	**ATCC 15305**
hypothetical protein	-	-	SE1039_RS03255	SEQMU2_RS08465	-	-	-	-	-	SSP_RS09475
arsenic resistance protein	-	-	SE1039_RS03260	SEQMU2_RS08470	BK815_RS06265	A6V26_RS02410	AA913_RS08510	SXYL_RS09990	BE24_RS01735	SSP_RS09470
hypothetical protein	-	-	SE1039_RS03265	SEQMU2_RS08475	BK815_RS06260	A6V26_RS02405	AA913_RS08515	SXYL_RS09985	BE24_RS01740	SSP_RS09465
hypothetical protein	-	-	SE1039_RS03270	SEQMU2_RS08480	BK815_RS06255	A6V26_RS02400	AA913_RS08520	SXYL_RS09980	BE24_RS01745	SSP_RS09460
hypothetical protein	-	-	SE1039_RS03275	SEQMU2_RS08485	BK815_RS06250	A6V26_RS02395	AA913_RS08525	SXYL_RS09975	BE24_RS01750	SSP_RS09455
hypothetical protein	-	-	SE1039_RS03280	SEQMU2_RS08490	BK815_RS06245	A6V26_RS02390	AA913_RS08530	SXYL_RS09970	BE24_RS01755	SSP_RS09450
NINE protein	-	-	SE1039_RS03285	SEQMU2_RS08495	BK815_RS06240	A6V26_RS02385	AA913_RS08535	SXYL_RS09965	BE24_RS01760	SSP_RS09445
hypothetical protein	-	-	SE1039_RS03295	SEQMU2_RS08505	BK815_RS06230	A6V26_RS02375	AA913_RS08545	SXYL_RS09955	BE24_RS01770	SSP_RS09435
hypothetical protein	-	-	SE1039_RS03305	SEQMU2_RS08515	BK815_RS06220	A6V26_RS02365	AA913_RS08555	SXYL_RS09945	BE24_RS01780	SSP_RS09425
hypothetical protein	-	-	SE1039_RS03310	SEQMU2_RS08520	BK815_RS06215	A6V26_RS02360	AA913_RS08560	SXYL_RS09940	BE24_RS01785	SSP_RS09420
Organic hydroperoxide resistance protein-like 1	-	-	SE1039_RS03315	SEQMU2_RS08525	BK815_RS06210	A6V26_RS02355	AA913_RS08565	SXYL_RS09935	BE24_RS01790	SSP_RS09415
HlyC/CorC family transporter	-	-	SE1039_RS03370	SEQMU2_RS08595	BK815_RS06140	A6V26_RS02285	AA913_RS08635	SXYL_RS09875	BE24_RS01850	SSP_RS09355
DUF368 domain-containing protein	-	-	SE1039_RS03375	SEQMU2_RS08600	BK815_RS06135	A6V26_RS02280	AA913_RS08640	SXYL_RS09870	BE24_RS01855	SSP_RS09350
hypothetical protein	-	-	SE1039_RS03460	SEQMU2_RS08685	-	-	-	-	-	-
MFS transporter	-	-	SE1039_RS03605	SEQMU2_RS08830	-	-	-	-	-	-
RimJ/RimL family protein N-acetyltransferase	-	-	SE1039_RS03610	SEQMU2_RS08835	-	-	-	-	-	-
hypothetical protein	-	-	SE1039_RS03665	SEQMU2_RS08890	BK815_RS05865	A6V26_RS01995	AA913_RS09055	SXYL_RS09590	BE24_RS02145	SSP_RS09080
histidine kinase	-	-	SE1039_RS03695	SEQMU2_RS08925	BK815_RS08280	-	-	-	-	-
competence protein	-	-	SE1039_RS03770	-	BK815_RS05750	A6V26_RS01885	AA913_RS09165	SXYL_RS09485	BE24_RS02245	SSP_RS08975
dihydrodipicolinate reductase	-	-	SE1039_RS03780	SEQMU2_RS09010	BK815_RS05740	A6V26_RS01875	AA913_RS09175	-	-	SSP_RS08965
hypothetical protein	-	-	SE1039_RS03870	SEQMU2_RS09100	BK815_RS05650	A6V26_RS01785	AA913_RS13030	SXYL_RS09390	BE24_RS02340	SSP_RS08875
bifunctional metallophosphatase/5-nucleotidase	-	-	SE1039_RS03895	SEQMU2_RS09125	BK815_RS05625	A6V26_RS01760	AA913_RS13055	SXYL_RS09365	BE24_RS02365	SSP_RS08850
resolvase	-	-	SE1039_RS03935	SEQMU2_RS09165	BK815_RS10485	A6V26_RS12805	AA913_RS04525	-	-	-
SulP family inorganic anion transporter	-	-	SE1039_RS03940	SEQMU2_RS09170	BK815_RS10490	-	-	-	-	-
universal stress protein	-	-	SE1039_RS03945	SEQMU2_RS09175	BK815_RS10495	-	-	-	-	-
hypothetical protein	-	-	SE1039_RS03950	SEQMU2_RS09180	-	A6V26_RS12755	AA913_RS04315	-	-	-
arsenical resistance operon transcriptional repressor ArsD	-	-	SE1039_RS03965	-	-	A6V26_RS12775	AA913_RS04295	-	-	-
arsenical pump-driving ATPase	-	-	SE1039_RS03970	-	-	A6V26_RS12780	AA913_RS04550	-	-	-
dehydrogenase	-	-	SE1039_RS03975	-	-	A6V26_RS12785	AA913_RS04545	-	-	-
autolysin	-	-	SE1039_RS04055	SEQMU2_RS09265	BK815_RS05525	A6V26_RS01660	AA913_RS02270	SXYL_RS09265	BE24_RS02465	SSP_RS08750
hypothetical protein	-	-	SE1039_RS04115	SEQMU2_RS09325	-	-	-	-	-	-

Product	*S. carnosus*		*S. equorum*		*S. succinus*			*S. xylosus*		*S. saprophyticus*
	**JCM 6069**	**TM300**	**KS1039**	**Mu2**	**14BME20**	**CSM 77**	**DSM 14617**	**C2a**	**HKUOPL8**	**ATCC 15305**
hypothetical protein	-	-	SE1039_RS04250	SEQMU2_RS09460	BK815_RS05335	A6V26_RS01470	AA913_RS02460	SXYL_RS09070	BE24_RS02660	SSP_RS08550
hypothetical protein	-	-	SE1039_RS04315	SEQMU2_RS09525	BK815_RS05260	A6V26_RS01395	AA913_RS02535	SXYL_RS08995	BE24_RS02735	SSP_RS08475
peptidase M23	-	-	SE1039_RS04430	SEQMU2_RS09640	BK815_RS05140	A6V26_RS01280	AA913_RS02650	SXYL_RS08880	BE24_RS02850	SSP_RS08360
XRE family transcriptional regulator	-	-	SE1039_RS04535	-	BK815_RS02940	-	AA913_RS06125	-	BE24_RS06340	-
hypothetical protein	-	-	SE1039_RS04555	SEQMU2_RS09730	-	-	-	-	-	-
N-acetyltransferase	-	-	SE1039_RS04565	SEQMU2_RS09740	BK815_RS03880	-	-	-	-	SSP_RS09530
phenol soluble modulin	-	-	SE1039_RS04575	SEQMU2_RS09750	BK815_RS05005	A6V26_RS01165	AA913_RS02765	SXYL_RS08445	BE24_RS02965	SSP_RS08085
glyoxalase	-	-	SE1039_RS04675	SEQMU2_RS09855	BK815_RS04905	A6V26_RS01065	AA913_RS02865	SXYL_RS08345	BE24_RS03075	SSP_RS07985
YitT family protein	-	-	SE1039_RS04740	SEQMU2_RS09920	BK815_RS04830	A6V26_RS00990	AA913_RS02940	SXYL_RS08285	BE24_RS03135	SSP_RS07925
site-specific integrase	-	-	SE1039_RS04775	-	BK815_RS02645	-	-	-	-	-
XRE family transcriptional regulator	-	-	SE1039_RS04790	SEQMU2_RS10670	-	-	-	-	-	-
helix-turn-helix domain-containing protein	-	-	SE1039_RS04795	SEQMU2_RS10675	-	-	-	-	-	-
transcriptional regulator	-	-	SE1039_RS04800	SEQMU2_RS07840	-	-	-	-	-	-
hypothetical protein	-	-	SE1039_RS04820	-	BK815_RS02685	-	-	-	-	-
hypothetical protein	-	-	SE1039_RS04825	SEQMU2_RS07875	-	-	-	SXYL_RS08755	BE24_RS05280	-
hypothetical protein	-	-	SE1039_RS04830	-	BK815_RS02695	-	-	-	BE24_RS05275	-
transcriptional regulator	-	-	SE1039_RS04860	-	BK815_RS02720	A6V26_RS11200	AA913_RS06350	-	BE24_RS05250	-
hypothetical protein	-	-	SE1039_RS04875	-	BK815_RS02735	A6V26_RS11185	AA913_RS06335	SXYL_RS08710	BE24_RS05225	-
hypothetical protein	-	-	SE1039_RS04885	SEQMU2_RS07925	-	-	-	-	BE24_RS05215	-
hypothetical protein	-	-	SE1039_RS04890	-	-	-	-	SXYL_RS08695	-	-
hypothetical protein	-	-	SE1039_RS04900	SEQMU2_RS07945	BK815_RS02760	-	-	-	-	-
hypothetical protein	-	-	SE1039_RS04905	SEQMU2_RS07950	BK815_RS02765	A6V26_RS11155	AA913_RS06305	SXYL_RS08675	BE24_RS05195	-
hypothetical protein	-	-	SE1039_RS04910	SEQMU2_RS07960	-	-	-	-	-	-
hypothetical protein	-	-	SE1039_RS04915	SEQMU2_RS07965	-	-	-	-	-	-
hypothetical protein	-	-	SE1039_RS04920	SEQMU2_RS07970	-	-	-	-	-	-
hypothetical protein	-	-	SE1039_RS04930	SEQMU2_RS07980	-	-	-	-	-	-
transcriptional regulator	-	-	SE1039_RS04935	SEQMU2_RS07985	-	-	-	-	-	-
hypothetical protein	-	-	SE1039_RS04940	SEQMU2_RS07990	-	-	-	-	-	-
terminase	-	-	SE1039_RS04950	SEQMU2_RS08000	-	-	-	-	-	-
phage portal protein	-	-	SE1039_RS04955	SEQMU2_RS08005	BK815_RS02815	-	-	-	-	-
phage head morphogenesis protein	-	-	SE1039_RS04960	SEQMU2_RS08010	BK815_RS02820	-	-	-	-	-

Product	*S. carnosus*		*S. equorum*		*S. succinus*			*S. xylosus*		*S. saprophyticus*
	**JCM 6069**	**TM300**	**KS1039**	**Mu2**	**14BME20**	**CSM 77**	**DSM 14617**	**C2a**	**HKUOPL8**	**ATCC 15305**
scaffolding protein	-	-	SE1039_RS04965	SEQMU2_RS08015	BK815_RS02825	-	-	-	-	-
major capsid protein	-	-	SE1039_RS04970	SEQMU2_RS08020	BK815_RS02830	-	-	-	-	-
hypothetical protein	-	-	SE1039_RS04975	SEQMU2_RS08030	-	-	-	-	-	-
phage head-tail adapter protein	-	-	SE1039_RS04980	SEQMU2_RS08035	BK815_RS02840	-	-	-	-	-
hypothetical protein	-	-	SE1039_RS04985	SEQMU2_RS08040	BK815_RS02845	-	-	-	-	-
hypothetical protein	-	-	SE1039_RS04990	SEQMU2_RS08045	BK815_RS02850	-	-	-	-	-
hypothetical protein	-	-	SE1039_RS04995	SEQMU2_RS08055	BK815_RS02855	-	-	-	-	-
phage major tail protein, TP901-1 family	-	-	SE1039_RS05000	SEQMU2_RS08060	BK815_RS02860	-	-	-	-	-
phage tail protein	-	-	SE1039_RS05005	SEQMU2_RS08065	BK815_RS02865	-	-	-	-	-
hypothetical protein	-	-	SE1039_RS05010	SEQMU2_RS08070	BK815_RS02870	-	-	-	-	-
hypothetical protein	-	-	SE1039_RS05015	SEQMU2_RS08075	BK815_RS02875	-	-	-	-	-
phage tail family protein	-	-	SE1039_RS05020	SEQMU2_RS08080	BK815_RS02880	-	-	-	-	-
hypothetical protein	-	-	SE1039_RS05030	-	-	A6V26_RS11010	-	SXYL_RS08525	-	-
hypothetical protein	-	-	SE1039_RS05035	SEQMU2_RS08095	BK815_RS13120	-	-	-	-	-
transcriptional regulator	-	-	SE1039_RS05055	SEQMU2_RS08115	BK815_RS08610	A6V26_RS04445	AA913_RS06865	SXYL_RS05020	-	-
hypothetical protein	-	-	SE1039_RS05060	SEQMU2_RS08120	-	-	-	-	-	-
hypothetical protein	-	-	SE1039_RS05065	SEQMU2_RS08125	-	-	-	-	-	-
hypothetical protein	-	-	SE1039_RS05085	SEQMU2_RS14190	BK815_RS03925	-	-	SXYL_RS05285	BE24_RS06355	-
hypothetical protein	-	-	SE1039_RS05090	-	-	-	-	SXYL_RS05290	BE24_RS05045	-
hypothetical protein	-	-	SE1039_RS05100	SEQMU2_RS09955	BK815_RS04775	A6V26_RS00935	AA913_RS02995	SXYL_RS08250	BE24_RS03170	-
N-acetyltransferase	-	-	SE1039_RS05105	SEQMU2_RS09960	BK815_RS04770	A6V26_RS00930	AA913_RS03000	SXYL_RS08245	BE24_RS03175	SSP_RS07890
peptidase M4 family protein	-	-	SE1039_RS05190	SEQMU2_RS10040	BK815_RS04690	A6V26_RS00850	AA913_RS03080	SXYL_RS08165	BE24_RS03255	SSP_RS07810
hypothetical protein	-	-	SE1039_RS05280	SEQMU2_RS10130	BK815_RS04600	A6V26_RS00760	AA913_RS03170	SXYL_RS08075	BE24_RS03345	SSP_RS07720
DNA-processing protein DprA	-	-	SE1039_RS05305	SEQMU2_RS10155	BK815_RS04575	A6V26_RS00735	AA913_RS03195	SXYL_RS08050	BE24_RS03370	SSP_RS07695
hypothetical protein	-	-	SE1039_RS05410	SEQMU2_RS10260	BK815_RS04470	A6V26_RS00630	AA913_RS03300	SXYL_RS07945	BE24_RS03475	SSP_RS07590
hypothetical protein	-	-	SE1039_RS05415	SEQMU2_RS10265	BK815_RS04465	A6V26_RS00625	AA913_RS03305	SXYL_RS07940	BE24_RS03480	-
hypothetical protein	-	-	SE1039_RS05525	SEQMU2_RS10375	BK815_RS04355	A6V26_RS00515	AA913_RS03415	SXYL_RS07830	BE24_RS03590	SSP_RS07470
Rrf2 family transcriptional regulator	-	-	SE1039_RS05530	SEQMU2_RS10380	BK815_RS04350	A6V26_RS00510	AA913_RS03420	SXYL_RS07820	BE24_RS03600	SSP_RS07460
NAD(P)/FAD-dependent oxidoreductase	-	-	SE1039_RS05535	SEQMU2_RS10385	-	-	-	-	BE24_RS03605	-
AraC family transcriptional regulator	-	-	SE1039_RS05560	SEQMU2_RS10415	-	-	-	-	-	-
lipase	-	-	SE1039_RS05570	SEQMU2_RS10420	-	-	-	-	-	-

Product	*S. carnosus*		*S. equorum*		*S. succinus*			*S. xylosus*		*S. saprophyticus*
	**JCM 6069**	**TM300**	**KS1039**	**Mu2**	**14BME20**	**CSM 77**	**DSM 14617**	**C2a**	**HKUOPL8**	**ATCC 15305**
MepB protein	-	-	SE1039_RS05620	SEQMU2_RS10470	BK815_RS02095	A6V26_RS10275	AA913_RS05415	SXYL_RS10020	BE24_RS01705	SSP_RS09500
anion permease	-	-	SE1039_RS05625	SEQMU2_RS10475	-	-	-	-	-	-
hypothetical protein	-	-	SE1039_RS05670	SEQMU2_RS10520	-	-	-	-	-	-
VOC family protein	-	-	SE1039_RS05675	SEQMU2_RS10525	-	-	-	-	-	-
hypothetical protein	-	-	SE1039_RS05680	SEQMU2_RS10530	BK815_RS04215	A6V26_RS00375	AA913_RS03560	SXYL_RS07700	BE24_RS03715	SSP_RS07355
hypothetical protein	-	-	SE1039_RS05750	SEQMU2_RS10600	-	-	-	SXYL_RS12555	BE24_RS12615	-
hypothetical protein	-	-	SE1039_RS05810	-	BK815_RS03770	A6V26_RS08925	AA913_RS13395	-	-	-
hypothetical protein	-	-	SE1039_RS05815	-	-	-	-	-	-	SSP_RS09360
hypothetical protein	-	-	SE1039_RS05820	SEQMU2_RS10915	-	-	-	-	-	-
hypothetical protein	-	-	SE1039_RS05825	SEQMU2_RS10920	BK815_RS04100	A6V26_RS00260	AA913_RS03675	SXYL_RS07585	BE24_RS03860	SSP_RS07175
acyltransferase	-	-	SE1039_RS05895	SEQMU2_RS10990	-	-	-	SXYL_RS07515	BE24_RS03930	SSP_RS07100
hypothetical protein	-	-	SE1039_RS05915	SEQMU2_RS11005	-	-	-	-	-	-
LacI family transcriptional regulator	-	-	SE1039_RS05920	SEQMU2_RS11010	BK815_RS04020	A6V26_RS00180	AA913_RS03755	SXYL_RS07495	BE24_RS03950	SSP_RS07085
D-ribose pyranase	-	-	SE1039_RS05925	SEQMU2_RS11015	BK815_RS04015	A6V26_RS00175	AA913_RS03760	SXYL_RS07490	BE24_RS03955	SSP_RS07080
sugar ABC transporter ATP-binding protein	-	-	SE1039_RS05930	-	BK815_RS04010	-	-	SXYL_RS07485	BE24_RS03960	SSP_RS07075
ribose ABC transporter permease	-	-	SE1039_RS05935	SEQMU2_RS11025	BK815_RS04005	A6V26_RS00165	AA913_RS03770	SXYL_RS07480	BE24_RS03965	SSP_RS07070
D-ribose ABC transporter substrate-binding protein	-	-	SE1039_RS05940	SEQMU2_RS11030	BK815_RS04000	A6V26_RS00160	AA913_RS03775	SXYL_RS07475	BE24_RS03970	SSP_RS07065
LysR family transcriptional regulator	-	-	SE1039_RS05965	-	BK815_RS10635	A6V26_RS08730	AA913_RS09885	-	-	-
phenolic acid decarboxylase	-	-	SE1039_RS05970	-	BK815_RS10640	A6V26_RS08725	AA913_RS09890	-	-	-
UbiD family decarboxylase	-	-	SE1039_RS05975	-	BK815_RS10645	A6V26_RS08720	AA913_RS09895	-	-	-
hypothetical protein	-	-	SE1039_RS05980	-	BK815_RS10650	A6V26_RS08715	AA913_RS09900	-	-	-
hypothetical protein	-	-	SE1039_RS05985	-	BK815_RS10655	A6V26_RS08710	AA913_RS09905	-	-	-
D-2-hydroxyacid dehydrogenase	-	-	SE1039_RS05995	SEQMU2_RS11060	-	-	-	SXYL_RS07445	BE24_RS04000	SSP_RS07035
hypothetical protein	-	-	SE1039_RS06020	SEQMU2_RS11070	-	-	-	-	-	SSP_RS08565
type 1 glutamine amidotransferase domain-containing protein	-	-	SE1039_RS06060	SEQMU2_RS11110	BK815_RS03730	A6V26_RS08965	AA913_RS09680	SXYL_RS07365	BE24_RS04070	SSP_RS06975
aldo/keto reductase	-	-	SE1039_RS06125	SEQMU2_RS11175	BK815_RS03665	A6V26_RS09030	AA913_RS09615	SXYL_RS07295	BE24_RS04140	SSP_RS06905
hypothetical protein	-	-	SE1039_RS13580	-	BK815_RS03565	A6V26_RS09130	AA913_RS09515	SXYL_RS07190	BE24_RS04245	SSP_RS06800
ABC transporter ATP-binding protein	-	-	SE1039_RS06235	SEQMU2_RS11285	BK815_RS03560	A6V26_RS09135	AA913_RS09510	SXYL_RS07185	BE24_RS04250	SSP_RS06795
dihydrofolate reductase	-	-	SE1039_RS06240	-	BK815_RS03555	A6V26_RS09140	AA913_RS09505	SXYL_RS07180	BE24_RS04255	SSP_RS06790
gfo/Idh/MocA family oxidoreductase	-	-	SE1039_RS06245	SEQMU2_RS11295	BK815_RS03550	A6V26_RS09145	AA913_RS09500	SXYL_RS07175	BE24_RS04260	SSP_RS06785
gfo/Idh/MocA family oxidoreductase	-	-	SE1039_RS06250	SEQMU2_RS11300	BK815_RS03545	A6V26_RS09150	AA913_RS09495	SXYL_RS07170	BE24_RS04265	-

Product	*S. carnosus*		*S. equorum*		*S. succinus*			*S. xylosus*		*S. saprophyticus*
	**JCM 6069**	**TM300**	**KS1039**	**Mu2**	**14BME20**	**CSM 77**	**DSM 14617**	**C2a**	**HKUOPL8**	**ATCC 15305**
hypothetical protein	-	-	SE1039_RS06255	SEQMU2_RS11305	BK815_RS03540	A6V26_RS09155	AA913_RS09490	SXYL_RS07165	-	-
sugar phosphate isomerase	-	-	SE1039_RS06260	SEQMU2_RS11310	BK815_RS03535	A6V26_RS09160	AA913_RS09485	SXYL_RS07160	BE24_RS04275	SSP_RS06770
AraC family transcriptional regulator	-	-	SE1039_RS06265	SEQMU2_RS11315	BK815_RS03530	A6V26_RS09165	AA913_RS09480	SXYL_RS07155	BE24_RS04280	-
XRE family transcriptional regulator	-	-	SE1039_RS06270	SEQMU2_RS11320	-	-	-	SXYL_RS07150	BE24_RS04285	SSP_RS06755
scaffolding protein	-	-	SE1039_RS06365	SEQMU2_RS11475	-	-	-	SXYL_RS06975	BE24_RS04385	SSP_RS06660
serine hydrolase family protein	-	-	SE1039_RS06380	SEQMU2_RS11490	BK815_RS03425	A6V26_RS09270	AA913_RS09375	SXYL_RS06960	BE24_RS04400	SSP_RS06645
NAD-dependent dehydratase	-	-	SE1039_RS06400	SEQMU2_RS11510	BK815_RS03405	A6V26_RS09290	AA913_RS09355	SXYL_RS06940	BE24_RS04420	SSP_RS06620
elastin-binding protein	-	-	SE1039_RS06575	SEQMU2_RS11685	BK815_RS03225	A6V26_RS09470	AA913_RS04615	SXYL_RS06765	BE24_RS04595	SSP_RS06445
alpha-glucosidase	-	-	SE1039_RS06675	SEQMU2_RS11785	BK815_RS03125	A6V26_RS09570	AA913_RS04715	SXYL_RS06665	BE24_RS04695	SSP_RS06345
LacI family transcriptional regulator	-	-	SE1039_RS06680	SEQMU2_RS11790	BK815_RS03120	A6V26_RS09575	AA913_RS04720	SXYL_RS06660	BE24_RS04700	SSP_RS06340
shikimate kinase	-	-	SE1039_RS06825	SEQMU2_RS11935	BK815_RS02980	A6V26_RS09715	AA913_RS04860	SXYL_RS06505	BE24_RS04845	SSP_RS06200
hypothetical protein	-	-	SE1039_RS06830	SEQMU2_RS11940	BK815_RS02975	A6V26_RS09720	AA913_RS04865	SXYL_RS06500	BE24_RS04850	SSP_RS06195
competence protein	-	-	SE1039_RS06835	SEQMU2_RS11945	BK815_RS02970	A6V26_RS09725	AA913_RS04870	SXYL_RS06495	BE24_RS04855	SSP_RS06190
competence protein	-	-	SE1039_RS06845	SEQMU2_RS11955	BK815_RS02960	A6V26_RS09735	AA913_RS04880	SXYL_RS06485	BE24_RS04865	SSP_RS06180
50S ribosomal protein L33	-	-	SE1039_RS06895	SEQMU2_RS12005	BK815_RS02595	A6V26_RS09785	AA913_RS04930	SXYL_RS06435	BE24_RS04915	SSP_RS06130
DNA internalization-related competence protein ComEC/Rec2	-	-	SE1039_RS07070	SEQMU2_RS12180	BK815_RS02420	A6V26_RS09960	AA913_RS05105	SXYL_RS06260	-	SSP_RS05955
multidrug transporter	-	-	SE1039_RS07225	SEQMU2_RS12335	BK815_RS02265	A6V26_RS10115	AA913_RS05260	SXYL_RS06100	BE24_RS05540	SSP_RS05800
hypothetical protein	-	-	SE1039_RS13595	-	BK815_RS02125	A6V26_RS13500	AA913_RS14290	-	-	SSP_RS12775
hypothetical protein	-	-	SE1039_RS07365	SEQMU2_RS12470	BK815_RS02120	A6V26_RS10255	AA913_RS05395	SXYL_RS05965	BE24_RS05675	SSP_RS05660
aldo/keto reductase	-	-	SE1039_RS07370	SEQMU2_RS12475	-	-	-	-	-	-
phytoene synthase	-	-	SE1039_RS07385	SEQMU2_RS12500	BK815_RS02090	A6V26_RS10280	AA913_RS05420	SXYL_RS05950	BE24_RS05690	SSP_RS05645
hypothetical protein	-	-	SE1039_RS07405	SEQMU2_RS12520	-	-	-	-	-	-
copper homeostasis protein CutC	-	-	SE1039_RS07610	SEQMU2_RS12725	BK815_RS01835	A6V26_RS10535	AA913_RS05675	SXYL_RS05730	BE24_RS05910	SSP_RS05420
hypothetical protein	-	-	SE1039_RS07625	SEQMU2_RS12740	BK815_RS01820	A6V26_RS10550	AA913_RS05690	SXYL_RS05715	BE24_RS05925	SSP_RS05405
RDD family protein	-	-	SE1039_RS07645	SEQMU2_RS12760	BK815_RS01800	A6V26_RS10570	AA913_RS05710	SXYL_RS05695	BE24_RS05945	SSP_RS05385
S49 family peptidase	-	-	SE1039_RS07650	SEQMU2_RS12765	BK815_RS01795	A6V26_RS10575	AA913_RS05715	SXYL_RS05690	BE24_RS05950	SSP_RS05380
spermidine acetyltransferase	-	-	SE1039_RS07685	SEQMU2_RS12800	BK815_RS01760	A6V26_RS10610	AA913_RS05750	SXYL_RS05655	BE24_RS05985	SSP_RS05345
hypothetical protein	-	-	SE1039_RS07770	SEQMU2_RS12895	BK815_RS01675	A6V26_RS10695	AA913_RS05835	SXYL_RS05570	BE24_RS06075	SSP_RS05260
transcriptional regulator	-	-	SE1039_RS07885	SEQMU2_RS13010	BK815_RS01560	A6V26_RS10830	AA913_RS05965	SXYL_RS05455	BE24_RS06190	SSP_RS05145
osmoprotectant ABC transporter substrate-binding protein	-	-	SE1039_RS07930	SEQMU2_RS13055	BK815_RS01515	A6V26_RS10875	AA913_RS06010	SXYL_RS05410	BE24_RS06235	SSP_RS05100
sigma-70 family RNA polymerase sigma factor	-	-	SE1039_RS07945	-	BK815_RS01500	A6V26_RS10890	AA913_RS06025	SXYL_RS05395	-	SSP_RS05075

Product	*S. carnosus*		*S. equorum*		*S. succinus*			*S. xylosus*		*S. saprophyticus*
	**JCM 6069**	**TM300**	**KS1039**	**Mu2**	**14BME20**	**CSM 77**	**DSM 14617**	**C2a**	**HKUOPL8**	**ATCC 15305**
hypothetical protein	-	-	SE1039_RS07950	SEQMU2_RS13075	BK815_RS01495	A6V26_RS10895	AA913_RS06030	SXYL_RS05390	-	SSP_RS05070
pentapeptide repeat protein	-	-	SE1039_RS07960	SEQMU2_RS13085	BK815_RS01485	A6V26_RS10905	AA913_RS06040	SXYL_RS05380	-	-
glycosyl transferase family A	-	-	SE1039_RS08030	SEQMU2_RS13155	-	-	-	-	BE24_RS06310	-
hypothetical protein	-	-	SE1039_RS08035	SEQMU2_RS13160	-	-	-	-	BE24_RS06315	-
hypothetical protein	-	-	SE1039_RS08130	SEQMU2_RS13255	BK815_RS01320	A6V26_RS11355	AA913_RS12420	SXYL_RS05095	BE24_RS06450	SSP_RS04895
formyltetrahydrofolate deformylase	-	-	SE1039_RS08175	SEQMU2_RS13300	BK815_RS01275	A6V26_RS11400	AA913_RS12375	SXYL_RS05050	BE24_RS06495	SSP_RS04850
hypothetical protein	-	-	SE1039_RS08180	SEQMU2_RS13310	-	-	-	-	-	-
hypothetical protein	-	-	SE1039_RS08185	SEQMU2_RS13315	-	-	-	-	BE24_RS06515	SSP_RS04840
hypothetical protein	-	-	SE1039_RS08220	SEQMU2_RS13350	BK815_RS01200	A6V26_RS11475	AA913_RS12100	SXYL_RS04980	BE24_RS06575	SSP_RS04805
ferritin	-	-	SE1039_RS08545	SEQMU2_RS00420	BK815_RS00870	A6V26_RS12340	AA913_RS11880	SXYL_RS04640	BE24_RS07095	SSP_RS04455
hypothetical protein	-	-	SE1039_RS08560	SEQMU2_RS00435	BK815_RS00855	A6V26_RS12355	AA913_RS11895	SXYL_RS04625	BE24_RS07110	SSP_RS04440
C4-dicarboxylate ABC transporter	-	-	SE1039_RS08655	SEQMU2_RS00530	BK815_RS00755	A6V26_RS12455	AA913_RS10260	SXYL_RS04525	BE24_RS07210	SSP_RS04340
putative holin-like toxin	-	-	SE1039_RS13600	-	-	-	-	SXYL_RS13295	-	-
hypothetical protein	-	-	SE1039_RS08695	SEQMU2_RS00585	BK815_RS00695	A6V26_RS12520	AA913_RS10190	SXYL_RS04485	BE24_RS07250	SSP_RS04300
hypothetical protein	-	-	SE1039_RS08725	SEQMU2_RS00615	BK815_RS00665	A6V26_RS12550	AA913_RS10160	-	BE24_RS07280	SSP_RS04270
damage-inducible protein DinB	-	-	SE1039_RS08750	SEQMU2_RS00640	BK815_RS00640	A6V26_RS12575	AA913_RS10135	SXYL_RS04430	BE24_RS07305	SSP_RS04240
hypothetical protein	-	-	SE1039_RS08755	SEQMU2_RS00645	BK815_RS06330	A6V26_RS02480	AA913_RS08445	-	-	SSP_RS09525
hypothetical protein	-	-	SE1039_RS08775	SEQMU2_RS00665	BK815_RS00620	-	-	SXYL_RS04410	BE24_RS07325	-
nitroreductase	-	-	SE1039_RS08780	SEQMU2_RS00670	BK815_RS00615	A6V26_RS12600	AA913_RS13245	SXYL_RS04405	BE24_RS07330	SSP_RS04200
LacI family transcriptional regulator	-	-	SE1039_RS08820	SEQMU2_RS00710	BK815_RS00575	A6V26_RS12640	AA913_RS13205	SXYL_RS04365	BE24_RS07370	SSP_RS04160
DNA mismatch repair protein MutS	-	-	SE1039_RS08845	SEQMU2_RS00735	BK815_RS00550	A6V26_RS12665	AA913_RS13180	SXYL_RS04340	BE24_RS07400	SSP_RS04135
VOC family protein	-	-	SE1039_RS08850	SEQMU2_RS00740	BK815_RS00545	A6V26_RS12670	AA913_RS13175	SXYL_RS04335	BE24_RS07405	SSP_RS04130
ATP-grasp domain-containing protein	-	-	SE1039_RS09255	SEQMU2_RS01130	BK815_RS00145	A6V26_RS11965	AA913_RS11545	SXYL_RS03940	BE24_RS07820	SSP_RS03720
ribokinase	-	-	SE1039_RS09300	SEQMU2_RS01175	BK815_RS00100	A6V26_RS12010	AA913_RS11590	SXYL_RS03895	BE24_RS07865	SSP_RS03675
membrane protein	-	-	SE1039_RS09315	SEQMU2_RS01190	BK815_RS00085	A6V26_RS12025	AA913_RS11605	SXYL_RS03880	BE24_RS07880	SSP_RS03660
iron ABC transporter ATP-binding protein	-	-	SE1039_RS09365	SEQMU2_RS01240	BK815_RS00035	A6V26_RS12080	AA913_RS11660	SXYL_RS03830	BE24_RS07940	SSP_RS03610
hypothetical protein	-	-	SE1039_RS09370	SEQMU2_RS01245	BK815_RS00030	A6V26_RS12085	AA913_RS11665	SXYL_RS03825	BE24_RS07945	SSP_RS03605
hypothetical protein	-	-	SE1039_RS09375	SEQMU2_RS01250	-	-	-	-	-	-
arginase	-	-	SE1039_RS09420	SEQMU2_RS01295	BK815_RS13095	A6V26_RS12130	AA913_RS11710	SXYL_RS03780	BE24_RS08015	SSP_RS03560
NADP-dependent oxidoreductase	-	SCA_RS08615	SE1039_RS09570	SEQMU2_RS01430	BK815_RS12925	A6V26_RS06425	AA913_RS00125	SXYL_RS03625	BE24_RS08180	SSP_RS03405
luciferase family oxidoreductase	-	-	SE1039_RS09575	SEQMU2_RS01435	BK815_RS12920	A6V26_RS06430	AA913_RS00130	SXYL_RS03620	BE24_RS08185	SSP_RS03400

Product	*S. carnosus*		*S. equorum*		*S. succinus*			*S. xylosus*		*S. saprophyticus*
	**JCM 6069**	**TM300**	**KS1039**	**Mu2**	**14BME20**	**CSM 77**	**DSM 14617**	**C2a**	**HKUOPL8**	**ATCC 15305**
GbsR/MarR family transcriptional regulator	-	SCA_RS10915	SE1039_RS09880	SEQMU2_RS01740	BK815_RS12615	A6V26_RS06730	AA913_RS00430	SXYL_RS03315	BE24_RS08490	SSP_RS03095
amino acid ABC transporter ATP-binding protein	-	-	SE1039_RS09945	SEQMU2_RS01805	-	-	-	SXYL_RS03250	BE24_RS08555	SSP_RS03025
amino acid ABC transporter substrate-binding protein	-	-	SE1039_RS09950	SEQMU2_RS01810	-	-	-	-	BE24_RS08560	SSP_RS03020
amino acid ABC transporter permease	-	-	SE1039_RS09955	SEQMU2_RS01815	-	-	-	SXYL_RS03240	BE24_RS08565	SSP_RS03015
amino acid ABC transporter permease	-	-	SE1039_RS09960	SEQMU2_RS01820	-	-	-	SXYL_RS03235	BE24_RS08570	SSP_RS03010
transcriptional regulator	-	-	SE1039_RS09965	SEQMU2_RS01825	-	-	-	SXYL_RS03230	BE24_RS08575	SSP_RS03005
CHAP domain-containing protein	-	-	SE1039_RS10010	SEQMU2_RS01870	BK815_RS12510	A6V26_RS06835	AA913_RS00535	SXYL_RS03190	BE24_RS08615	SSP_RS02965
hypothetical protein	-	-	SE1039_RS10070	SEQMU2_RS01930	BK815_RS12445	A6V26_RS06900	AA913_RS00600	SXYL_RS03130	BE24_RS08675	SSP_RS02905
hypothetical protein	-	-	SE1039_RS10075	SEQMU2_RS01935	BK815_RS12440	A6V26_RS06905	AA913_RS00605	SXYL_RS03125	BE24_RS08680	SSP_RS02900
alkaline phosphatase	-	-	SE1039_RS10095	SEQMU2_RS01955	BK815_RS12420	A6V26_RS06925	AA913_RS00625	SXYL_RS03105	BE24_RS08700	SSP_RS02880
symporter	-	-	SE1039_RS10130	SEQMU2_RS01990	BK815_RS12385	A6V26_RS06960	AA913_RS00660	SXYL_RS03070	BE24_RS08735	SSP_RS02845
PTS alpha-glucoside transporter subunit IIBC	-	-	SE1039_RS10140	-	BK815_RS12375	A6V26_RS06970	AA913_RS00670	SXYL_RS03060	BE24_RS08745	SSP_RS02835
MurR/RpiR family transcriptional regulator	-	-	SE1039_RS10145	SEQMU2_RS02005	BK815_RS12370	A6V26_RS06975	AA913_RS00675	SXYL_RS03055	BE24_RS08750	SSP_RS02830
MOSC domain-containing protein	-	-	SE1039_RS10215	SEQMU2_RS02075	BK815_RS12310	A6V26_RS07035	AA913_RS00735	SXYL_RS02985	BE24_RS08820	SSP_RS02755
hypothetical protein	-	-	SE1039_RS10245	SEQMU2_RS02105	-	-	-	SXYL_RS02950	BE24_RS08855	SSP_RS02720
sodium/glutamate symporter	-	-	SE1039_RS10250	SEQMU2_RS02110	BK815_RS12275	A6V26_RS07070	AA913_RS00770	SXYL_RS02930	BE24_RS08860	SSP_RS02705
hypothetical protein	-	-	SE1039_RS10270	SEQMU2_RS02130	BK815_RS12255	A6V26_RS07090	AA913_RS00790	SXYL_RS02910	BE24_RS08880	SSP_RS02685
alpha/beta hydrolase	-	-	SE1039_RS10275	SEQMU2_RS02135	BK815_RS12250	A6V26_RS07095	AA913_RS00795	SXYL_RS02905	BE24_RS08885	SSP_RS02680
N-acetyltransferase	-	-	SE1039_RS10380	SEQMU2_RS02240	BK815_RS12140	A6V26_RS07205	AA913_RS00905	SXYL_RS02785	-	SSP_RS02575
nuclear export factor GLE1	-	-	SE1039_RS10395	SEQMU2_RS02255	BK815_RS12125	A6V26_RS07220	AA913_RS00920	-	-	-
hypothetical protein	-	-	SE1039_RS10400	SEQMU2_RS02260	BK815_RS12120	A6V26_RS07225	AA913_RS00925	-	-	-
YfcC family protein	-	-	SE1039_RS10430	SEQMU2_RS02290	BK815_RS12090	-	-	SXYL_RS02745	BE24_RS09030	SSP_RS02530
LLM class flavin-dependent oxidoreductase	-	-	SE1039_RS10435	SEQMU2_RS02295	BK815_RS12085	A6V26_RS07255	AA913_RS00955	SXYL_RS02740	BE24_RS09035	SSP_RS02525
Na/Pi cotransporter	-	-	SE1039_RS10445	SEQMU2_RS02305	BK815_RS12070	A6V26_RS07270	AA913_RS00970	SXYL_RS02725	BE24_RS09050	SSP_RS02510
PTS trehalose transporter subunits IIBC	-	-	SE1039_RS10465	SEQMU2_RS02325	BK815_RS12050	A6V26_RS07290	AA913_RS00990	SXYL_RS02705	BE24_RS09070	SSP_RS02490
hypothetical protein	-	-	SE1039_RS10470	SEQMU2_RS02330	-	-	-	SXYL_RS02700	BE24_RS09075	SSP_RS02485
AraC family transcriptional regulator	-	-	SE1039_RS10475	SEQMU2_RS02335	BK815_RS12045	A6V26_RS07295	AA913_RS00995	SXYL_RS02695	BE24_RS09080	SSP_RS02480
hypothetical protein	-	-	SE1039_RS10560	SEQMU2_RS02420	-	-	-	SXYL_RS02600	BE24_RS09175	-
N-acetyltransferase	-	-	SE1039_RS10565	SEQMU2_RS02425	BK815_RS12010	A6V26_RS07330	AA913_RS01030	SXYL_RS02595	BE24_RS09180	SSP_RS02440
transcriptional regulator	-	-	SE1039_RS10575	SEQMU2_RS02435	BK815_RS12000	A6V26_RS07340	AA913_RS01040	-	-	SSP_RS02430
DNA-binding protein	-	-	SE1039_RS10585	SEQMU2_RS02465	BK815_RS11975	A6V26_RS07365	AA913_RS01070	SXYL_RS02560	BE24_RS09215	SSP_RS02410

Product	*S. carnosus*		*S. equorum*		*S. succinus*			*S. xylosus*		*S. saprophyticus*
	**JCM 6069**	**TM300**	**KS1039**	**Mu2**	**14BME20**	**CSM 77**	**DSM 14617**	**C2a**	**HKUOPL8**	**ATCC 15305**
hypothetical protein	-	-	SE1039_RS10595	SEQMU2_RS02475	-	-	-	SXYL_RS02550	BE24_RS09225	SSP_RS02400
zinc ABC transporter substrate-binding protein	-	-	SE1039_RS10605	SEQMU2_RS02485	BK815_RS11965	A6V26_RS07375	AA913_RS01080	SXYL_RS02540	BE24_RS09235	SSP_RS02390
cystatin-like fold lipoprotein	-	-	SE1039_RS10615	SEQMU2_RS02495	BK815_RS11955	A6V26_RS07385	AA913_RS01090	SXYL_RS02530	BE24_RS09245	SSP_RS02380
hypothetical protein	-	-	SE1039_RS10620	SEQMU2_RS02500	BK815_RS11945	A6V26_RS07395	AA913_RS01100	SXYL_RS02525	BE24_RS09250	SSP_RS02370
lantibiotic ABC transporter ATP-binding protein	-	-	SE1039_RS10630	SEQMU2_RS02510	BK815_RS09335	A6V26_RS05155	AA913_RS02010	-	-	-
ABC transporter permease	-	-	SE1039_RS10635	SEQMU2_RS02515	BK815_RS09330	A6V26_RS05150	AA913_RS02005	-	-	-
AraC family transcriptional regulator	-	-	SE1039_RS10665	SEQMU2_RS02545	-	-	-	SXYL_RS02490	BE24_RS09295	SSP_RS02330
N-acetyltransferase	-	-	SE1039_RS10680	SEQMU2_RS02560	BK815_RS11895	A6V26_RS07445	AA913_RS01150	SXYL_RS02465	BE24_RS09320	SSP_RS02305
membrane protein	-	-	SE1039_RS10690	SEQMU2_RS02570	-	-	-	SXYL_RS02455	BE24_RS09330	SSP_RS02295
membrane protein	-	-	SE1039_RS10695	SEQMU2_RS02575	-	-	-	SXYL_RS02450	BE24_RS09335	-
hypothetical protein	-	-	SE1039_RS10700	SEQMU2_RS02580	BK815_RS11885	A6V26_RS07455	AA913_RS01160	SXYL_RS02445	BE24_RS09340	SSP_RS02285
arginine utilization protein RocB	-	-	SE1039_RS10705	SEQMU2_RS02585	BK815_RS11880	A6V26_RS07460	AA913_RS01165	SXYL_RS02440	BE24_RS09345	SSP_RS02275
hypothetical protein	-	-	SE1039_RS10710	SEQMU2_RS02590	-	-	-	-	-	SSP_RS02245
sulfonate ABC transporter ATP-binding protein	-	-	SE1039_RS10715	SEQMU2_RS02595	-	-	-	-	-	SSP_RS02240
nitrate ABC transporter substrate-binding protein	-	-	SE1039_RS10720	SEQMU2_RS02600	-	-	-	-	-	SSP_RS02235
ABC transporter permease	-	-	SE1039_RS10725	SEQMU2_RS02605	-	-	-	-	-	SSP_RS02230
acyl-CoA dehydrogenase	-	-	SE1039_RS10730	SEQMU2_RS02610	-	-	-	-	-	SSP_RS02225
amino acid transporter	-	-	SE1039_RS10785	SEQMU2_RS02665	BK815_RS11805	A6V26_RS07535	AA913_RS01240	SXYL_RS02360	BE24_RS09425	SSP_RS02170
hypothetical protein	-	-	SE1039_RS10815	SEQMU2_RS02695	BK815_RS11775	A6V26_RS07565	AA913_RS01270	SXYL_RS02325	BE24_RS09460	SSP_RS02135
hypothetical protein	-	-	SE1039_RS10840	SEQMU2_RS02720	-	-	-	-	-	-
D-serine ammonia-lyase	-	-	SE1039_RS10845	SEQMU2_RS02725	BK815_RS11720	A6V26_RS07620	AA913_RS01325	SXYL_RS02295	BE24_RS09490	SSP_RS02105
cobalamin ABC transporter substrate-binding protein	-	-	SE1039_RS10895	SEQMU2_RS02775	BK815_RS11670	A6V26_RS07670	AA913_RS01375	SXYL_RS02245	-	SSP_RS07020
glyoxalase	-	-	SE1039_RS10900	SEQMU2_RS02780	BK815_RS11665	A6V26_RS07675	AA913_RS01380	SXYL_RS02240	BE24_RS09545	SSP_RS02050
N-acetyltransferase	-	-	SE1039_RS10905	SEQMU2_RS02785	BK815_RS11655	A6V26_RS07685	AA913_RS01390	SXYL_RS02230	BE24_RS09555	SSP_RS02045
hypothetical protein	-	-	SE1039_RS10915	SEQMU2_RS02795	BK815_RS11645	A6V26_RS07695	AA913_RS01400	SXYL_RS02220	BE24_RS09565	SSP_RS02035
hypothetical protein	-	-	SE1039_RS10920	SEQMU2_RS02800	BK815_RS11640	A6V26_RS07700	AA913_RS01405	SXYL_RS02215	BE24_RS09570	SSP_RS02030
glucuronate isomerase	-	-	SE1039_RS10925	SEQMU2_RS02805	-	-	-	SXYL_RS02190	BE24_RS09595	-
glycosyl hydrolase	-	-	SE1039_RS10930	SEQMU2_RS02810	-	-	-	SXYL_RS02185	BE24_RS09600	-
beta-glucuronidase	-	-	SE1039_RS10935	SEQMU2_RS02815	BK815_RS11635	A6V26_RS07705	AA913_RS01410	SXYL_RS02180	-	-
MFS transporter	-	-	SE1039_RS10940	SEQMU2_RS02820	BK815_RS11630	A6V26_RS07710	AA913_RS01415	SXYL_RS02175	BE24_RS09610	-
AraC family transcriptional regulator	-	-	SE1039_RS10945	SEQMU2_RS02825	BK815_RS11625	A6V26_RS07715	AA913_RS01420	SXYL_RS02170	BE24_RS09615	-

Product	*S. carnosus*		*S. equorum*		*S. succinus*			*S. xylosus*		*S. saprophyticus*
	**JCM 6069**	**TM300**	**KS1039**	**Mu2**	**14BME20**	**CSM 77**	**DSM 14617**	**C2a**	**HKUOPL8**	**ATCC 15305**
membrane protein	-	-	SE1039_RS10950	SEQMU2_RS02830	BK815_RS11620	A6V26_RS07720	AA913_RS01425	SXYL_RS02165	BE24_RS09620	SSP_RS02020
sodium:proton antiporter	-	-	SE1039_RS11015	SEQMU2_RS02895	BK815_RS11555	A6V26_RS07785	AA913_RS01490	SXYL_RS02080	BE24_RS09705	SSP_RS01940
membrane protein	-	-	SE1039_RS11020	SEQMU2_RS02900	-	-	-	SXYL_RS02075	BE24_RS09710	SSP_RS01935
aldehyde dehydrogenase	-	-	SE1039_RS11045	SEQMU2_RS02925	BK815_RS11505	A6V26_RS07835	AA913_RS01540	SXYL_RS02040	BE24_RS09745	SSP_RS01900
hypothetical protein	-	-	SE1039_RS11070	SEQMU2_RS02950	BK815_RS11490	A6V26_RS07850	AA913_RS01555	SXYL_RS02015	BE24_RS09770	SSP_RS01875
haloacid dehalogenase	-	-	SE1039_RS11085	SEQMU2_RS02965	BK815_RS11475	A6V26_RS07885	AA913_RS01590	SXYL_RS02000	BE24_RS09785	SSP_RS01845
N-acetyltransferase	-	-	SE1039_RS11120	SEQMU2_RS03000	BK815_RS11435	A6V26_RS07925	AA913_RS01630	SXYL_RS01960	BE24_RS09825	SSP_RS01805
hypothetical protein	-	-	SE1039_RS13605	-	BK815_RS11415	A6V26_RS07945	AA913_RS01650	SXYL_RS01945	BE24_RS09840	-
transcriptional regulator	-	-	SE1039_RS11130	SEQMU2_RS03010	BK815_RS11410	A6V26_RS07950	AA913_RS01655	-	BE24_RS09845	SSP_RS01755
glycosyl transferase family 2	-	-	SE1039_RS11135	SEQMU2_RS03015	BK815_RS11390	A6V26_RS07970	AA913_RS01675	SXYL_RS01900	BE24_RS09865	SSP_RS01730
CDP-glycerol glycerophosphotransferase family protein	-	-	SE1039_RS11140	SEQMU2_RS03020	BK815_RS11385	A6V26_RS07975	AA913_RS01680	SXYL_RS01895	BE24_RS09870	SSP_RS01725
ribitol-5-phosphate dehydrogenase	-	-	SE1039_RS11145	SEQMU2_RS04895	BK815_RS11380	A6V26_RS07980	AA913_RS01685	SXYL_RS01890	BE24_RS09875	SSP_RS01720
2-C-methyl-D-erythritol 4-phosphate cytidylyltransferase	-	-	SE1039_RS11150	-	BK815_RS11375	A6V26_RS07985	AA913_RS01690	SXYL_RS01885	BE24_RS09880	SSP_RS01715
hypothetical protein	-	-	SE1039_RS11175	SEQMU2_RS03050	-	A6V26_RS08010	AA913_RS01715	SXYL_RS01855	BE24_RS09910	SSP_RS01685
alpha/beta hydrolase	-	-	SE1039_RS11185	SEQMU2_RS03060	BK815_RS11350	A6V26_RS08015	AA913_RS01720	SXYL_RS01845	BE24_RS09920	SSP_RS01665
N-acetyltransferase	-	-	SE1039_RS11190	SEQMU2_RS03065	BK815_RS11345	A6V26_RS08020	AA913_RS01725	SXYL_RS01840	BE24_RS09925	SSP_RS01660
D-cysteine desulfhydrase family protein	-	-	SE1039_RS11205	SEQMU2_RS03080	BK815_RS11335	A6V26_RS08030	AA913_RS01735	SXYL_RS01825	BE24_RS09940	SSP_RS01645
lactonase	-	-	SE1039_RS11255	-	BK815_RS11280	A6V26_RS08085	AA913_RS01790	SXYL_RS01770	BE24_RS09995	SSP_RS01590
glycine/betaine ABC transporter permease	-	-	SE1039_RS11275	SEQMU2_RS03150	-	-	-	-	-	SSP_RS01570
hypothetical protein	-	-	SE1039_RS11285	SEQMU2_RS03160	-	-	-	SXYL_RS01745	BE24_RS10030	SSP_RS01560
MFS transporter	-	-	SE1039_RS11290	SEQMU2_RS03165	BK815_RS11255	A6V26_RS08110	AA913_RS01815	SXYL_RS01740	BE24_RS10035	-
esterase	-	-	SE1039_RS11295	SEQMU2_RS03170	BK815_RS11250	A6V26_RS08115	AA913_RS01820	SXYL_RS01735	BE24_RS10040	SSP_RS01550
hypothetical protein	-	-	SE1039_RS11300	-	BK815_RS11245	A6V26_RS08120	AA913_RS01825	SXYL_RS01730	BE24_RS10045	SSP_RS01545
hypothetical protein	-	-	SE1039_RS11315	SEQMU2_RS03190	BK815_RS11230	A6V26_RS08135	AA913_RS01840	SXYL_RS01715	BE24_RS10060	SSP_RS01530
intercellular adhesion protein C	-	-	SE1039_RS11335	-	BK815_RS11210	A6V26_RS08155	AA913_RS01860	SXYL_RS01695	BE24_RS10080	SSP_RS01510
hypothetical protein	-	-	SE1039_RS11340	SEQMU2_RS03215	BK815_RS11205	A6V26_RS08160	AA913_RS03940	SXYL_RS01690	BE24_RS10085	SSP_RS01505
membrane protein	-	-	SE1039_RS11365	SEQMU2_RS03240	BK815_RS11185	A6V26_RS08180	AA913_RS03960	SXYL_RS01670	BE24_RS10105	SSP_RS01485
anion transporter	-	-	SE1039_RS11370	SEQMU2_RS03245	BK815_RS11180	A6V26_RS08185	AA913_RS03965	SXYL_RS01665	BE24_RS10110	SSP_RS01480
hypothetical protein	-	-	SE1039_RS11375	SEQMU2_RS03250	BK815_RS11175	A6V26_RS08190	AA913_RS03970	SXYL_RS01660	BE24_RS10115	SSP_RS01475
lactococcin 972 family bacteriocin	-	-	SE1039_RS11380	-	-	-	-	SXYL_RS01655	-	-
hypothetical protein	-	-	SE1039_RS11385	-	-	-	-	SXYL_RS01650	-	-

Product	*S. carnosus*		*S. equorum*		*S. succinus*			*S. xylosus*		*S. saprophyticus*
	**JCM 6069**	**TM300**	**KS1039**	**Mu2**	**14BME20**	**CSM 77**	**DSM 14617**	**C2a**	**HKUOPL8**	**ATCC 15305**
bacteriocin ABC transporter ATP-binding protein	-	-	SE1039_RS11395	-	-	-	-	SXYL_RS01640	-	-
short-chain dehydrogenase	-	-	SE1039_RS11400	SEQMU2_RS03255	BK815_RS11170	A6V26_RS08195	AA913_RS03975	SXYL_RS01625	BE24_RS10120	SSP_RS01465
SulP family inorganic anion transporter	-	-	SE1039_RS11415	SEQMU2_RS03270	BK815_RS11155	A6V26_RS08210	AA913_RS03990	SXYL_RS01610	BE24_RS10135	SSP_RS01450
6-pyruvoyl tetrahydrobiopterin synthase	-	-	SE1039_RS11450	SEQMU2_RS03305	BK815_RS11120	A6V26_RS08245	AA913_RS04025	SXYL_RS01575	BE24_RS10170	SSP_RS01415
DNA-3-methyladenine glycosylase 2 family protein	-	-	SE1039_RS11460	SEQMU2_RS03315	-	-	-	-	-	SSP_RS01405
peptidase M23	-	-	SE1039_RS11465	SEQMU2_RS03320	BK815_RS11110	A6V26_RS08255	AA913_RS04035	SXYL_RS01565	-	SSP_RS01400
nucleoside hydrolase	-	-	SE1039_RS11470	SEQMU2_RS03325	BK815_RS11105	A6V26_RS08260	AA913_RS04040	SXYL_RS01560	BE24_RS10185	SSP_RS01395
TetR family transcriptional regulator	-	-	SE1039_RS11475	SEQMU2_RS03330	BK815_RS11090	A6V26_RS08275	AA913_RS04055	SXYL_RS01545	BE24_RS10200	SSP_RS01380
serine protease	-	-	SE1039_RS11480	SEQMU2_RS03335	BK815_RS11085	A6V26_RS08280	AA913_RS04060	SXYL_RS01540	BE24_RS10205	SSP_RS01375
dihydroorotate dehydrogenase (quinone)	-	-	SE1039_RS11500	SEQMU2_RS03355	BK815_RS11040	A6V26_RS08325	AA913_RS04105	SXYL_RS01520	BE24_RS10225	SSP_RS01355
hydrolase	-	-	SE1039_RS11510	SEQMU2_RS03365	BK815_RS11030	A6V26_RS08335	AA913_RS04115	SXYL_RS01510	BE24_RS10235	SSP_RS01345
glyoxalase	-	-	SE1039_RS11515	SEQMU2_RS03370	BK815_RS11025	A6V26_RS08340	AA913_RS04120	SXYL_RS01505	BE24_RS10240	SSP_RS01340
MarR family transcriptional regulator	-	-	SE1039_RS11520	SEQMU2_RS03375	BK815_RS11015	A6V26_RS08350	AA913_RS04130	SXYL_RS01490	BE24_RS10255	SSP_RS01330
ferrichrome ABC transporter substrate-binding protein	-	-	SE1039_RS11525	SEQMU2_RS03380	-	-	-	SXYL_RS01485	BE24_RS10260	SSP_RS01325
nickel ABC transporter substrate-binding protein	-	-	SE1039_RS11550	SEQMU2_RS03405	BK815_RS10990	A6V26_RS08375	AA913_RS04155	SXYL_RS01460	BE24_RS10285	SSP_RS01300
hypothetical protein	-	-	SE1039_RS11595	SEQMU2_RS03450	BK815_RS10945	A6V26_RS08420	AA913_RS04200	SXYL_RS01415	BE24_RS10330	SSP_RS01255
membrane protein	-	-	SE1039_RS11600	SEQMU2_RS03455	-	-	-	-	-	-
threonine/serine exporter	-	-	SE1039_RS11625	SEQMU2_RS03480	-	-	-	SXYL_RS01380	BE24_RS10365	SSP_RS11965
threonine/serine exporter	-	-	SE1039_RS11630	SEQMU2_RS03485	-	-	-	SXYL_RS01375	BE24_RS10370	SSP_RS11970
ROK family protein	-	-	SE1039_RS11635	SEQMU2_RS03490	BK815_RS10900	A6V26_RS08465	AA913_RS04245	SXYL_RS01370	BE24_RS10375	SSP_RS12735
2-dehydropantoate 2-reductase	-	-	SE1039_RS11665	SEQMU2_RS03520	BK815_RS10880	A6V26_RS08485	AA913_RS04265	SXYL_RS01335	BE24_RS10420	SSP_RS01200
transporter	-	-	SE1039_RS11670	SEQMU2_RS03525	-	A6V26_RS05125	AA913_RS01980	SXYL_RS12515	BE24_RS11540	-
tripartite tricarboxylate transporter TctB family protein	-	-	SE1039_RS11675	SEQMU2_RS03530	-	A6V26_RS05130	AA913_RS01985	-	BE24_RS11545	-
tripartite tricarboxylate transporter substrate binding protein	-	-	SE1039_RS11680	SEQMU2_RS03535	-	A6V26_RS05135	AA913_RS01990	-	-	-
thioesterase family protein	-	-	SE1039_RS11685	SEQMU2_RS03540	BK815_RS08655	A6V26_RS04490	AA913_RS06910	SXYL_RS01330	BE24_RS10425	-
3-hydroxybenzoate 6-hydroxylase	-	-	SE1039_RS11690	SEQMU2_RS03545	-	-	-	-	-	-
cupin	-	-	SE1039_RS11695	SEQMU2_RS03550	-	-	-	-	-	-
DinB family protein	-	-	SE1039_RS11700	SEQMU2_RS03555	-	-	-	-	-	-
2-hydroxyhepta-2,4-diene-1,7-dioate isomerase	-	-	SE1039_RS11705	SEQMU2_RS03560	-	-	-	-	-	-
IclR family transcriptional regulator	-	-	SE1039_RS11710	SEQMU2_RS03565	-	-	-	-	-	-
MFS transporter	-	-	SE1039_RS11715	SEQMU2_RS03570	-	-	-	-	-	-

Product	*S. carnosus*		*S. equorum*		*S. succinus*			*S. xylosus*		*S. saprophyticus*
	**JCM 6069**	**TM300**	**KS1039**	**Mu2**	**14BME20**	**CSM 77**	**DSM 14617**	**C2a**	**HKUOPL8**	**ATCC 15305**
hypothetical protein	-	-	SE1039_RS11720	-	BK815_RS08700	A6V26_RS04535	AA913_RS06955	-	-	-
PTS ascorbate transporter subunit IIC	-	-	SE1039_RS11725	SEQMU2_RS03580	BK815_RS10875	A6V26_RS08490	AA913_RS04270	SXYL_RS01320	BE24_RS10435	-
PTS lactose transporter subunit IIB	-	-	SE1039_RS11730	SEQMU2_RS03585	BK815_RS10870	A6V26_RS08495	AA913_RS04275	SXYL_RS01315	BE24_RS10440	-
PTS ascorbate transporter subunit IIA	-	-	SE1039_RS11735	SEQMU2_RS03590	BK815_RS10865	A6V26_RS08500	AA913_RS04280	SXYL_RS01310	BE24_RS10445	-
PTS sugar transporter subunit IIA	-	-	SE1039_RS11740	SEQMU2_RS03595	BK815_RS10860	A6V26_RS08505	AA913_RS04285	SXYL_RS01305	BE24_RS10450	-
PTS sugar transporter subunit IIB	-	-	SE1039_RS11750	SEQMU2_RS03605	-	-	-	SXYL_RS01265	-	SSP_RS01170
hypothetical protein	-	-	SE1039_RS11755	SEQMU2_RS03610	-	-	-	SXYL_RS01260	-	SSP_RS01165
PTS cellobiose transporter subunit IIC	-	-	SE1039_RS11760	SEQMU2_RS03615	-	-	-	SXYL_RS01255	-	SSP_RS01160
ROK family protein	-	-	SE1039_RS11765	SEQMU2_RS03620	-	-	-	-	-	-
carbohydrate kinase	-	-	SE1039_RS11770	-	-	-	-	SXYL_RS01285	BE24_RS10470	-
pseudouridine-5-phosphate glycosidase	-	-	SE1039_RS11775	-	-	-	-	SXYL_RS01280	BE24_RS10475	-
pyrimidine nucleoside transporter NupC	-	-	SE1039_RS11780	-	-	-	-	SXYL_RS01275	BE24_RS10480	-
CHAP domain-containing protein	-	-	SE1039_RS11785	-	BK815_RS11285	A6V26_RS08080	AA913_RS01785	SXYL_RS01775	BE24_RS09990	SSP_RS01595
aminoacyltransferase	-	-	SE1039_RS11790	SEQMU2_RS03635	BK815_RS11950	A6V26_RS07390	AA913_RS01095	-	-	SSP_RS02375
PTS glucose transporter subunit IIB	-	-	SE1039_RS11800	SEQMU2_RS03645	BK815_RS10820	A6V26_RS08545	AA913_RS10070	SXYL_RS01235	BE24_RS10490	-
MFS transporter	-	-	SE1039_RS11810	SEQMU2_RS03655	BK815_RS10810	A6V26_RS08555	AA913_RS10060	SXYL_RS01225	BE24_RS10500	SSP_RS01125
lactate permease	-	-	SE1039_RS11815	SEQMU2_RS03660	-	-	-	SXYL_RS01220	BE24_RS10505	SSP_RS01120
hypothetical protein	-	-	SE1039_RS11820	SEQMU2_RS03665	BK815_RS10805	A6V26_RS08560	AA913_RS10055	SXYL_RS01200	BE24_RS10525	SSP_RS01115
FMN reductase (NADPH)	-	-	SE1039_RS11825	SEQMU2_RS03670	BK815_RS10800	A6V26_RS08565	AA913_RS10050	SXYL_RS01185	BE24_RS10530	SSP_RS01100
5,10-methylenetetrahydromethanopterin reductase	-	-	SE1039_RS11830	SEQMU2_RS03675	-	-	-	-	-	-
acetate--CoA ligase	-	-	SE1039_RS11835	SEQMU2_RS03680	-	-	-	-	-	-
acetylglutamate kinase	-	-	SE1039_RS11840	SEQMU2_RS03685	BK815_RS10790	A6V26_RS08575	AA913_RS10040	SXYL_RS01175	BE24_RS10545	SSP_RS01085
bifunctional ornithine acetyltransferase/N-acetylglutamate synthase	-	-	SE1039_RS11845	SEQMU2_RS03690	BK815_RS10785	A6V26_RS08580	AA913_RS10035	SXYL_RS01170	BE24_RS10550	SSP_RS01080
N-acetyl-gamma-glutamyl-phosphate reductase	-	-	SE1039_RS11850	SEQMU2_RS03695	BK815_RS10780	A6V26_RS08585	AA913_RS10030	SXYL_RS01165	BE24_RS10555	SSP_RS01075
ornithine--oxo-acid transaminase	-	-	SE1039_RS11855	SEQMU2_RS03700	BK815_RS10775	A6V26_RS08590	AA913_RS10025	SXYL_RS01160	BE24_RS10560	SSP_RS01070
idonate transporter	-	-	SE1039_RS11860	SEQMU2_RS03705	-	-	-	-	-	-
multidrug ABC transporter ATP-binding protein	-	-	SE1039_RS11865	SEQMU2_RS03710	-	-	-	SXYL_RS01150	-	SSP_RS01055
ABC transporter permease	-	-	SE1039_RS11870	SEQMU2_RS03715	-	-	-	SXYL_RS01145	-	SSP_RS01050
ABC transporter permease	-	-	SE1039_RS11875	SEQMU2_RS03720	-	-	-	SXYL_RS01140	-	SSP_RS01045
hypothetical protein	-	-	SE1039_RS11880	SEQMU2_RS03725	-	-	-	SXYL_RS01135	-	SSP_RS01040
capsular polysaccharide biosynthesis protein CapA	-	-	SE1039_RS11895	SEQMU2_RS03740	BK815_RS12555	A6V26_RS06790	AA913_RS00490	SXYL_RS01120	BE24_RS10580	SSP_RS01025

Product	*S. carnosus*		*S. equorum*		*S. succinus*			*S. xylosus*		*S. saprophyticus*
	**JCM 6069**	**TM300**	**KS1039**	**Mu2**	**14BME20**	**CSM 77**	**DSM 14617**	**C2a**	**HKUOPL8**	**ATCC 15305**
hypothetical protein	-	-	SE1039_RS11900	SEQMU2_RS03745	-	-	-	-	-	-
N-acetyltransferase	-	-	SE1039_RS11910	SEQMU2_RS03755	BK815_RS10750	A6V26_RS08615	AA913_RS10000	SXYL_RS01110	BE24_RS10590	SSP_RS01010
N-acetyltransferase	-	-	SE1039_RS11920	SEQMU2_RS03765	-	-	-	-	-	-
QacE family quaternary ammonium compound efflux SMR transporter	-	-	SE1039_RS11925	SEQMU2_RS03770	-	A6V26_RS13380	-	-	-	-
ABC transporter substrate-binding protein	-	-	SE1039_RS11950	SEQMU2_RS03795	-	-	-	-	-	-
SAM-dependent methyltransferase	-	-	SE1039_RS11955	SEQMU2_RS03800	BK815_RS10705	A6V26_RS08660	AA913_RS09955	SXYL_RS01080	BE24_RS10630	SSP_RS00950
amino acid permease	-	-	SE1039_RS11975	SEQMU2_RS03820	BK815_RS10690	-	-	SXYL_RS01065	BE24_RS10645	SSP_RS00935
PLP-dependent aminotransferase family protein	-	-	SE1039_RS11990	SEQMU2_RS03835	BK815_RS10675	A6V26_RS08690	AA913_RS09925	SXYL_RS01050	BE24_RS10660	SSP_RS00920
cardiolipin synthase	-	-	SE1039_RS11995	SEQMU2_RS03840	BK815_RS10670	A6V26_RS08695	AA913_RS09920	SXYL_RS01045	BE24_RS10665	SSP_RS00915
MFS transporter	-	-	SE1039_RS12000	SEQMU2_RS03845	BK815_RS10665	A6V26_RS08700	AA913_RS09915	SXYL_RS01040	BE24_RS10670	SSP_RS00910
XRE family transcriptional regulator	-	-	SE1039_RS12005	SEQMU2_RS03850	BK815_RS10660	A6V26_RS08705	AA913_RS09910	SXYL_RS01035	BE24_RS10675	SSP_RS00905
transcriptional regulator	-	-	SE1039_RS12010	SEQMU2_RS03855	-	-	-	SXYL_RS01030	BE24_RS10680	SSP_RS12750
hypothetical protein	-	-	SE1039_RS12015	SEQMU2_RS03860	-	-	-	SXYL_RS01025	BE24_RS10685	-
hypothetical protein	-	-	SE1039_RS12020	SEQMU2_RS03865	-	-	-	SXYL_RS01020	BE24_RS10690	-
VOC family protein	-	-	SE1039_RS12025	SEQMU2_RS03870	BK815_RS10630	A6V26_RS08735	AA913_RS09880	SXYL_RS01015	BE24_RS10695	-
transcriptional regulator	-	-	SE1039_RS12040	SEQMU2_RS03885	BK815_RS10610	A6V26_RS08755	AA913_RS09860	SXYL_RS01000	BE24_RS10710	SSP_RS00880
2-keto-3-deoxygluconate permease	-	-	SE1039_RS12050	SEQMU2_RS03895	BK815_RS10600	A6V26_RS08765	AA913_RS09850	SXYL_RS00990	BE24_RS10720	-
hypothetical protein	-	-	SE1039_RS12055	SEQMU2_RS03900	BK815_RS10595	A6V26_RS08770	AA913_RS09845	SXYL_RS00985	BE24_RS10725	SSP_RS00870
lactate racemization operon protein LarE	-	-	SE1039_RS12060	SEQMU2_RS03905	BK815_RS10590	A6V26_RS08775	AA913_RS09840	SXYL_RS00980	BE24_RS10730	SSP_RS00865
1-(5-phosphoribosyl)-5-amino-4-imidazole- carboxylate carboxylase	-	-	SE1039_RS12065	SEQMU2_RS03910	BK815_RS10585	A6V26_RS08780	AA913_RS09835	SXYL_RS00975	BE24_RS10735	SSP_RS00860
TIGR00299 family protein	-	-	SE1039_RS12070	SEQMU2_RS03915	BK815_RS10580	A6V26_RS08785	AA913_RS09830	SXYL_RS00970	BE24_RS10740	SSP_RS00855
gfo/Idh/MocA family oxidoreductase	-	-	SE1039_RS12075	SEQMU2_RS03920	BK815_RS10575	A6V26_RS08790	AA913_RS09825	SXYL_RS00965	BE24_RS10745	-
hypothetical protein	-	-	SE1039_RS12080	SEQMU2_RS03925	BK815_RS09285	-	-	SXYL_RS00960	BE24_RS10750	-
general stress protein	-	-	SE1039_RS12085	SEQMU2_RS03930	BK815_RS09290	A6V26_RS05095	AA913_RS01950	SXYL_RS00955	BE24_RS10755	-
hypothetical protein	-	-	SE1039_RS12090	SEQMU2_RS03935	-	-	-	SXYL_RS00950	BE24_RS10760	-
lipoate--protein ligase	-	-	SE1039_RS12095	SEQMU2_RS03940	-	-	-	SXYL_RS00945	BE24_RS10765	-
deacetylase SIR2	-	-	SE1039_RS12100	SEQMU2_RS03945	-	-	-	SXYL_RS00940	BE24_RS10770	-
hypothetical protein	-	-	SE1039_RS12105	SEQMU2_RS03950	-	-	-	SXYL_RS00935	BE24_RS10775	-
glycine cleavage system protein H	-	-	SE1039_RS12110	SEQMU2_RS03955	-	-	-	SXYL_RS00930	BE24_RS10780	-
LLM class flavin-dependent oxidoreductase	-	-	SE1039_RS12115	SEQMU2_RS03960	-	-	-	SXYL_RS00925	BE24_RS10785	-
NADH-dependent flavin oxidoreductase	-	-	SE1039_RS12120	SEQMU2_RS03965	-	-	-	-	BE24_RS10790	-

Product	*S. carnosus*		*S. equorum*		*S. succinus*			*S. xylosus*		*S. saprophyticus*
	**JCM 6069**	**TM300**	**KS1039**	**Mu2**	**14BME20**	**CSM 77**	**DSM 14617**	**C2a**	**HKUOPL8**	**ATCC 15305**
TetR family transcriptional regulator	-	-	SE1039_RS12140	SEQMU2_RS03985	BK815_RS05850	A6V26_RS01980	AA913_RS09070	-	-	-
ACP synthase	-	-	SE1039_RS12145	SEQMU2_RS03990	BK815_RS05845	A6V26_RS01975	AA913_RS09075	-	-	-
NAD(+)--rifampin ADP-ribosyltransferase	-	-	SE1039_RS12155	SEQMU2_RS04000	BK815_RS10505	A6V26_RS08860	AA913_RS09755	SXYL_RS00890	BE24_RS10810	-
glycerophosphodiester phosphodiesterase	-	-	SE1039_RS12160	SEQMU2_RS04005	BK815_RS10480	A6V26_RS08865	AA913_RS09750	SXYL_RS00885	BE24_RS10815	SSP_RS00835
MFS transporter	-	-	SE1039_RS12170	SEQMU2_RS04015	BK815_RS10470	A6V26_RS08875	AA913_RS09740	SXYL_RS00875	BE24_RS10825	SSP_RS00815
tRNA-dihydrouridine synthase	-	-	SE1039_RS12175	SEQMU2_RS04020	BK815_RS09780	A6V26_RS05640	AA913_RS10585	SXYL_RS00870	BE24_RS10830	SSP_RS00810
hypothetical protein	-	-	SE1039_RS13615	SEQMU2_RS04040	-	-	AA913_RS12635	-	BE24_RS10865	-
hypothetical protein	-	-	SE1039_RS12215	SEQMU2_RS04075	BK815_RS10415	A6V26_RS06255	AA913_RS12670	SXYL_RS00810	BE24_RS10890	SSP_RS00745
hypothetical protein	-	-	SE1039_RS13620	SEQMU2_RS04080	-	-	-	-	-	-
XRE family transcriptional regulator	-	-	SE1039_RS12225	SEQMU2_RS04085	BK815_RS10405	A6V26_RS06245	AA913_RS12680	SXYL_RS00790	BE24_RS10895	SSP_RS00725
transporter	-	-	SE1039_RS12230	SEQMU2_RS04090	BK815_RS10400	A6V26_RS06240	AA913_RS12685	SXYL_RS00785	BE24_RS10900	-
pyridoxamine 5-phosphate oxidase	-	SCA_RS11450	SE1039_RS12235	SEQMU2_RS04095	-	-	-	-	-	-
peptide ABC transporter permease	-	-	SE1039_RS12240	SEQMU2_RS04100	BK815_RS09995	A6V26_RS05835	AA913_RS10390	SXYL_RS00210	BE24_RS11495	SSP_RS00445
peptide ABC transporter ATP-binding protein	-	-	SE1039_RS12245	SEQMU2_RS04105	BK815_RS10000	A6V26_RS05840	AA913_RS10385	SXYL_RS00215	BE24_RS11490	SSP_RS00450
LLM class flavin-dependent oxidoreductase	-	-	SE1039_RS12255	SEQMU2_RS04115	BK815_RS10240	A6V26_RS06080	AA913_RS12760	SXYL_RS00755	BE24_RS10930	-
NAD(P)H-dependent oxidoreductase	-	-	SE1039_RS12260	SEQMU2_RS04120	-	-	-	SXYL_RS00750	-	-
permease	-	-	SE1039_RS12310	SEQMU2_RS04170	BK815_RS10200	A6V26_RS06040	AA913_RS12605	SXYL_RS00685	BE24_RS10995	SSP_RS00695
ABC transporter ATP-binding protein	-	-	SE1039_RS12330	-	BK815_RS09810	A6V26_RS05680	AA913_RS10545	-	-	SSP_RS00675
hypothetical protein	-	-	SE1039_RS12335	-	BK815_RS09815	A6V26_RS05685	AA913_RS10540	-	-	SSP_RS00670
XylR family transcriptional regulator	-	-	SE1039_RS12340	SEQMU2_RS04190	BK815_RS10555	A6V26_RS08810	AA913_RS09805	SXYL_RS00660	BE24_RS11030	-
xylose isomerase	-	-	SE1039_RS12345	SEQMU2_RS04195	BK815_RS10560	A6V26_RS08805	AA913_RS09810	SXYL_RS00655	BE24_RS11035	-
xylulokinase	-	-	SE1039_RS12350	SEQMU2_RS04200	BK815_RS10565	A6V26_RS08800	AA913_RS09815	SXYL_RS00650	BE24_RS11040	-
D-xylose transporter XylE	-	-	SE1039_RS12355	SEQMU2_RS04205	BK815_RS10570	-	-	SXYL_RS00645	BE24_RS11045	-
2-keto-3-deoxygluconate permease	-	-	SE1039_RS12360	-	BK815_RS10140	A6V26_RS05980	AA913_RS12545	-	-	-
GntR family transcriptional regulator	-	-	SE1039_RS12365	-	BK815_RS10135	A6V26_RS05975	AA913_RS12540	-	-	-
sugar kinase	-	-	SE1039_RS12370	-	BK815_RS10130	A6V26_RS05970	AA913_RS12535	-	-	-
2-dehydro-3-deoxyphosphooctonate aldolase	-	-	SE1039_RS12375	-	BK815_RS10125	A6V26_RS05965	AA913_RS12530	-	-	-
SelA-like pyridoxal phosphate-dependent enzyme	-	-	SE1039_RS12380	-	BK815_RS10120	A6V26_RS05960	AA913_RS12525	-	-	-
amidohydrolase/deacetylase family metallohydrolase	-	-	SE1039_RS12385	-	BK815_RS10115	A6V26_RS05955	AA913_RS12520	-	-	-
2-deoxy-D-gluconate 3-dehydrogenase	-	-	SE1039_RS12390	-	-	-	-	SXYL_RS02160	BE24_RS09625	-
hemolysin activation protein	-	-	SE1039_RS12450	SEQMU2_RS04245	-	-	-	SXYL_RS00620	BE24_RS11070	SSP_RS00640

Product	*S. carnosus*		*S. equorum*		*S. succinus*			*S. xylosus*		*S. saprophyticus*
	**JCM 6069**	**TM300**	**KS1039**	**Mu2**	**14BME20**	**CSM 77**	**DSM 14617**	**C2a**	**HKUOPL8**	**ATCC 15305**
MFS transporter	-	-	SE1039_RS12455	-	BK815_RS10550	A6V26_RS08815	AA913_RS09800	SXYL_RS00615	BE24_RS11075	-
L-arabinose isomerase	-	-	SE1039_RS12460	SEQMU2_RS04255	BK815_RS10545	A6V26_RS08820	AA913_RS09795	SXYL_RS00610	BE24_RS11080	-
ribulose 5-phosphate epimerase	-	-	SE1039_RS12465	SEQMU2_RS04260	BK815_RS10540	A6V26_RS08825	AA913_RS09790	SXYL_RS00605	BE24_RS11085	-
ATPase	-	-	SE1039_RS12470	SEQMU2_RS04265	BK815_RS10535	A6V26_RS08830	AA913_RS09785	SXYL_RS00600	BE24_RS11090	-
GntR family transcriptional regulator	-	-	SE1039_RS12475	SEQMU2_RS04270	BK815_RS10530	A6V26_RS08835	AA913_RS09780	SXYL_RS00595	BE24_RS11095	-
haloacid dehalogenase	-	-	SE1039_RS12515	SEQMU2_RS04310	-	-	-	-	-	-
hypothetical protein	-	-	SE1039_RS12520	SEQMU2_RS04315	-	-	-	-	-	-
transglycosylase	-	SCA_RS08120	SE1039_RS12525	SEQMU2_RS04320	BK815_RS10065	A6V26_RS05905	AA913_RS10320	SXYL_RS00570	BE24_RS11120	-
transglycosylase	-	-	SE1039_RS12530	SEQMU2_RS04325	-	-	-	-	-	-
transglycosylase SceD 3	-	-	SE1039_RS12535	-	-	-	-	-	-	SSP_RS03895
hypothetical protein	-	-	SE1039_RS12550	SEQMU2_RS04365	-	-	-	-	-	-
PTS sugar transporter subunit IIA	-	-	SE1039_RS12555	SEQMU2_RS04370	BK815_RS09900	A6V26_RS05765	AA913_RS10460	-	BE24_RS11210	-
PTS sugar transporter subunit IIA	-	-	SE1039_RS12560	SEQMU2_RS04375	BK815_RS09895	A6V26_RS05760	AA913_RS10465	-	BE24_RS11215	-
PTS galactitol transporter subunit IIB	-	-	SE1039_RS12565	SEQMU2_RS04380	BK815_RS09890	A6V26_RS05755	AA913_RS10470	-	BE24_RS11220	-
PTS galactitol transporter subunit IIC	-	-	SE1039_RS12570	SEQMU2_RS04385	BK815_RS09885	A6V26_RS05750	AA913_RS10475	-	BE24_RS11225	-
sorbitol dehydrogenase	-	-	SE1039_RS12575	SEQMU2_RS04390	BK815_RS09880	A6V26_RS05745	AA913_RS10480	-	BE24_RS11230	-
galactitol-1-phosphate 5-dehydrogenase	-	-	SE1039_RS12580	SEQMU2_RS04395	BK815_RS09875	A6V26_RS05740	AA913_RS10485	-	BE24_RS11235	-
YitT family protein	-	-	SE1039_RS12585	SEQMU2_RS04400	BK815_RS10105	A6V26_RS05945	AA913_RS12510	SXYL_RS00495	BE24_RS11240	-
2,4-dienoyl-CoA reductase	-	-	SE1039_RS12590	SEQMU2_RS04405	BK815_RS10060	A6V26_RS05900	AA913_RS10325	SXYL_RS00490	BE24_RS11245	SSP_RS00570
PadR family transcriptional regulator	-	-	SE1039_RS12595	SEQMU2_RS04450	-	-	-	SXYL_RS00485	BE24_RS11250	-
hypothetical protein	-	-	SE1039_RS12600	SEQMU2_RS04455	BK815_RS09440	A6V26_RS05325	AA913_RS02180	SXYL_RS00480	BE24_RS11255	-
hypothetical protein	-	-	SE1039_RS12605	SEQMU2_RS04485	-	-	-	SXYL_RS00475	BE24_RS11260	SSP_RS00540
glycosyltransferase	-	-	SE1039_RS12610	SEQMU2_RS04490	-	-	-	SXYL_RS00470	BE24_RS11265	SSP_RS00535
GTP-binding protein	-	-	SE1039_RS12615	SEQMU2_RS04495	-	-	-	SXYL_RS00460	BE24_RS11275	-
N-acetyltransferase	-	-	SE1039_RS12620	SEQMU2_RS04500	-	-	-	SXYL_RS00455	-	-
hypothetical protein	-	-	SE1039_RS12625	SEQMU2_RS04505	-	-	-	-	-	-
endonuclease	-	-	SE1039_RS12650	SEQMU2_RS04530	BK815_RS09950	A6V26_RS05810	AA913_RS10415	-	-	-
siderophore biosynthesis protein SbnI	-	-	SE1039_RS12655	SEQMU2_RS04535	-	-	-	-	-	-
diaminopimelate decarboxylase	-	-	SE1039_RS12660	SEQMU2_RS04540	-	-	-	-	-	-
siderophore biosynthesis protein SbnG	-	-	SE1039_RS12665	SEQMU2_RS04545	-	-	-	-	-	-
IucA/IucC family siderophore biosynthesis protein	-	-	SE1039_RS12670	SEQMU2_RS04550	-	-	-	-	-	-

Product	*S. carnosus*		*S. equorum*		*S. succinus*			*S. xylosus*		*S. saprophyticus*
	**JCM 6069**	**TM300**	**KS1039**	**Mu2**	**14BME20**	**CSM 77**	**DSM 14617**	**C2a**	**HKUOPL8**	**ATCC 15305**
siderophore biosynthesis protein SbnE	-	-	SE1039_RS12675	SEQMU2_RS04555	-	-	-	-	-	-
MFS transporter	-	-	SE1039_RS12680	SEQMU2_RS04560	-	-	-	-	-	-
siderophore biosynthesis protein SbnC	-	-	SE1039_RS12685	SEQMU2_RS04565	-	-	-	-	-	-
2,3-diaminopropionate biosynthesis protein SbnB	-	-	SE1039_RS12690	SEQMU2_RS04570	-	-	-	-	-	-
2,3-diaminopropionate biosynthesis protein SbnA	-	-	SE1039_RS12695	SEQMU2_RS04575	-	-	-	-	-	-
iron ABC transporter substrate-binding protein	-	-	SE1039_RS12700	SEQMU2_RS04580	-	-	-	-	-	-
iron ABC transporter permease	-	-	SE1039_RS12705	SEQMU2_RS04585	-	-	-	-	-	-
hypothetical protein	-	-	SE1039_RS12715	SEQMU2_RS04595	-	-	-	-	-	-
hypothetical protein	-	-	SE1039_RS12720	SEQMU2_RS04600	-	-	-	SXYL_RS13070	BE24_RS12185	-
hypothetical protein	-	-	SE1039_RS12725	SEQMU2_RS04605	-	A6V26_RS12955	AA913_RS04380	SXYL_RS13065	BE24_RS12190	-
acyl-CoA thioesterase	-	-	SE1039_RS12730	SEQMU2_RS04610	-	A6V26_RS12950	AA913_RS04385	SXYL_RS13060	BE24_RS12195	-
LysR family transcriptional regulator	-	-	SE1039_RS12735	SEQMU2_RS04615	-	A6V26_RS12945	AA913_RS04390	SXYL_RS13055	BE24_RS12200	-
hypothetical protein	-	-	SE1039_RS12775	-	BK815_RS09960	-	-	SXYL_RS00425	-	-
ABC-2 transporter family protein	-	-	SE1039_RS12785	-	BK815_RS09970	-	-	SXYL_RS00415	-	-
accessory Sec system glycosylation chaperone GtfB	-	SCA_RS11085	SE1039_RS12790	-	BK815_RS10375	A6V26_RS06215	AA913_RS12710	-	-	-
peptide ABC transporter ATP-binding protein	-	-	SE1039_RS12810	SEQMU2_RS04650	-	-	-	-	-	-
peptide ABC transporter permease	-	-	SE1039_RS12815	SEQMU2_RS04655	-	-	-	-	-	-
tautomerase family protein	-	-	SE1039_RS12820	SEQMU2_RS04660	BK815_RS10380	A6V26_RS06220	AA913_RS12705	SXYL_RS00190	BE24_RS11515	-
sodium:pantothenate symporter	-	-	SE1039_RS12825	SEQMU2_RS04665	-	-	-	-	-	-
N5,N10-methylene tetrahydromethanopterin reductase	-	-	SE1039_RS12830	SEQMU2_RS04670	BK815_RS08685	A6V26_RS04520	AA913_RS06940	-	-	-
hypothetical protein	-	-	SE1039_RS12835	SEQMU2_RS04675	-	-	-	-	-	-
membrane protein	-	-	SE1039_RS12840	SEQMU2_RS04680	BK815_RS08875	A6V26_RS04710	AA913_RS07130	SXYL_RS12825	BE24_RS12400	-
MerR family transcriptional regulator	-	SCA_RS11975	SE1039_RS12845	SEQMU2_RS04685	-	-	-	-	-	-
NADP-dependent oxidoreductase	-	-	SE1039_RS12850	SEQMU2_RS04690	-	-	-	-	-	-
sodium:proline symporter	-	-	SE1039_RS12855	SEQMU2_RS04695	-	-	-	-	-	-
hypothetical protein	-	-	SE1039_RS12865	SEQMU2_RS04705	BK815_RS09775	A6V26_RS05630	AA913_RS10595	-	BE24_RS11570	SSP_RS00410
hypothetical protein	-	-	SE1039_RS12870	SEQMU2_RS04710	BK815_RS09770	A6V26_RS05625	AA913_RS10600	-	BE24_RS11575	SSP_RS00405
hypothetical protein	-	-	SE1039_RS12875	SEQMU2_RS04715	-	-	-	-	-	-
luciferase family oxidoreductase	-	-	SE1039_RS12880	SEQMU2_RS04740	-	-	-	-	-	-
DsbA family protein	-	-	SE1039_RS12885	-	-	-	-	SXYL_RS00855	BE24_RS10845	-
TetR family transcriptional regulator	-	-	SE1039_RS12890	SEQMU2_RS04750	-	-	-	SXYL_RS00860	BE24_RS10840	-

Product	*S. carnosus*		*S. equorum*		*S. succinus*			*S. xylosus*		*S. saprophyticus*
	**JCM 6069**	**TM300**	**KS1039**	**Mu2**	**14BME20**	**CSM 77**	**DSM 14617**	**C2a**	**HKUOPL8**	**ATCC 15305**
hypothetical protein	-	-	SE1039_RS12895	SEQMU2_RS04755	-	-	-	SXYL_RS00865	BE24_RS10835	SSP_RS00790
hypothetical protein	-	-	SE1039_RS12900	-	-	-	-	SXYL_RS00280	BE24_RS11420	-
ABC transporter ATPase	-	-	SE1039_RS12905	SEQMU2_RS04780	-	-	-	SXYL_RS00450	BE24_RS11285	-
LPXTG cell wall anchor domain-containing protein	-	-	SE1039_RS13635	SEQMU2_RS04785	-	-	-	-	-	SSP_RS00530
malate dehydrogenase	-	-	SE1039_RS12920	SEQMU2_RS04795	BK815_RS09075	A6V26_RS04910	AA913_RS07330	SXYL_RS11145	BE24_RS00615	-
malate permease	-	-	SE1039_RS12925	SEQMU2_RS04800	BK815_RS09070	A6V26_RS04905	AA913_RS07325	SXYL_RS11150	BE24_RS00610	-
response regulator	-	-	SE1039_RS12930	SEQMU2_RS04805	BK815_RS09065	A6V26_RS04900	AA913_RS07320	SXYL_RS11190	BE24_RS00580	-
two-component system sensor histidine kinase DcuS	-	-	SE1039_RS12935	SEQMU2_RS04810	BK815_RS09060	A6V26_RS04895	AA913_RS07315	SXYL_RS11195	BE24_RS00575	-
6-phospho-beta-glucosidase	-	-	SE1039_RS12945	SEQMU2_RS02450	BK815_RS09860	A6V26_RS05725	AA913_RS10500	SXYL_RS00295	BE24_RS11405	SSP_RS00615
PTS beta-glucoside transporter subunit EIIBCA	-	-	SE1039_RS12950	-	BK815_RS09855	A6V26_RS05720	AA913_RS10505	SXYL_RS00290	BE24_RS11410	-
transcription antiterminator LicT	-	-	SE1039_RS12955	-	BK815_RS09850	A6V26_RS05715	AA913_RS10510	SXYL_RS00285	BE24_RS11415	-
alcohol dehydrogenase	-	-	SE1039_RS12960	SEQMU2_RS04820	BK815_RS09845	A6V26_RS05710	AA913_RS10515	SXYL_RS12655	BE24_RS12515	-
tRNA-specific adenosine deaminase	-	-	SE1039_RS12965	SEQMU2_RS04825	-	-	-	-	-	-
hypothetical protein	-	-	SE1039_RS13095	SEQMU2_RS04970	-	-	-	-	-	-
hypothetical protein	-	-	SE1039_RS13165	SEQMU2_RS04980	-	-	-	-	-	SSP_RS00280
type-1 restriction enzyme R protein	-	-	SE1039_RS13170	SEQMU2_RS04985	-	A6V26_RS05570	AA913_RS10655	-	-	SSP_RS00275
type I restriction-modification system subunit M	-	-	SE1039_RS13175	SEQMU2_RS04990	-	A6V26_RS05580	AA913_RS10645	-	-	SSP_RS00270
hypothetical protein	-	-	SE1039_RS13185	SEQMU2_RS05005	-	-	-	-	BE24_RS11860	SSP_RS00260
hypothetical protein	-	-	SE1039_RS13200	-	-	-	-	-	BE24_RS11875	-
hypothetical protein	-	-	SE1039_RS13275	SEQMU2_RS04950	BK815_RS01250	A6V26_RS11425	-	SXYL_RS11540	BE24_RS00195	-
hypothetical protein	-	-	SE1039_RS13280	SEQMU2_RS04960	-	-	-	-	-	-
hypothetical protein	-	-	SE1039_RS13295	SEQMU2_RS04975	-	-	-	-	-	SSP_RS00285
hypothetical protein	-	-	SE1039_RS13310	-	BK815_RS09735	-	-	-	-	SSP_RS00205
hypothetical protein	-	-	SE1039_RS13350	-	-	-	-	-	BE24_RS11565	-
hypothetical protein	-	-	SE1039_RS13360	SEQMU2_RS04880	-	-	-	-	-	-
MFS transporter	-	-	SE1039_RS13405	SEQMU2_RS05490	BK815_RS09630	A6V26_RS05515	AA913_RS10715	-	-	SSP_RS00110
MarR family transcriptional regulator	-	-	SE1039_RS13410	SEQMU2_RS05495	BK815_RS09625	A6V26_RS05510	AA913_RS10720	-	-	SSP_RS00105
glycosyl transferase	-	-	SE1039_RS13425	SEQMU2_RS05510	BK815_RS09610	A6V26_RS05495	AA913_RS10735	SXYL_RS00090	BE24_RS11960	SSP_RS00090
homoserine acetyltransferase	-	-	SE1039_RS13455	SEQMU2_RS05540	BK815_RS09580	A6V26_RS05465	AA913_RS10765	SXYL_RS00060	BE24_RS11990	SSP_RS00060
membrane protein	-	-	SE1039_RS13460	SEQMU2_RS05545	BK815_RS09575	A6V26_RS05460	AA913_RS10770	SXYL_RS00055	BE24_RS11995	SSP_RS00055
branched-chain amino acid ABC transporter permease	-	-	SE1039_RS13465	SEQMU2_RS05550	BK815_RS09570	A6V26_RS05455	AA913_RS10775	SXYL_RS00050	BE24_RS12000	SSP_RS00050

Product	*S. carnosus*		*S. equorum*		*S. succinus*			*S. xylosus*		*S. saprophyticus*
	**JCM 6069**	**TM300**	**KS1039**	**Mu2**	**14BME20**	**CSM 77**	**DSM 14617**	**C2a**	**HKUOPL8**	**ATCC 15305**
aminoacyltransferase	-	-	-	SEQMU2_RS14220	BK815_RS11950	A6V26_RS07390	AA913_RS01095	-	-	SSP_RS02375
mobilization protein	-	-	-	SEQMU2_RS14230	-	A6V26_RS13275	AA913_RS12950	-	-	-
protein rlx|Protein rlx	-	-	-	SEQMU2_RS14235	-	A6V26_RS13280	AA913_RS12955	-	-	-
plasmid replication initiation protein|Probable replication protein rep	-	-	-	SEQMU2_RS14255	-	-	AA913_RS08690	-	-	-
glutamine amidotransferase	-	-	-	SEQMU2_RS14065	-	A6V26_RS13400	AA913_RS13145	-	-	SSP_RS08200
3-hexulose-6-phosphate synthase 2	-	-	-	SEQMU2_RS14070	-	A6V26_RS13320	-	-	-	SSP_RS08205
Zn-dependent alcohol dehydrogenase|Lovastatin di ketide synthase LovF	-	-	-	SEQMU2_RS14075	-	A6V26_RS13315	AA913_RS12990	-	-	-
plasmid replication-associated protein	-	-	-	SEQMU2_RS14080	-	A6V26_RS13310	AA913_RS12985	-	-	-
replication initiator protein A	-	-	-	SEQMU2_RS14085	-	A6V26_RS13305	AA913_RS12980	-	-	SSP_RS08260
N-acetyltransferase	-	-	-	SEQMU2_RS14090	BK815_RS09435	A6V26_RS05320	AA913_RS02175	SXYL_RS12245	BE24_RS13025	-
short chain dehydrogenase	-	-	-	SEQMU2_RS14140	BK815_RS10740	A6V26_RS08625	AA913_RS09990	SXYL_RS12150	BE24_RS13120	-
hypothetical protein	-	-	-	SEQMU2_RS14145	BK815_RS10735	A6V26_RS08630	AA913_RS09985	SXYL_RS12155	BE24_RS13115	-
MerR family transcriptional regulator	-	-	-	SEQMU2_RS14150	BK815_RS10730	A6V26_RS08635	AA913_RS09980	SXYL_RS12160	BE24_RS13110	-
hypothetical protein	-	-	-	SEQMU2_RS13785	BK815_RS03820	A6V26_RS00010	AA913_RS03925	SXYL_RS11175	-	-
hypothetical protein	-	-	-	SEQMU2_RS13790	BK815_RS03835	-	-	-	-	-
membrane protein|Uncharacterized protein YhcI	-	-	-	SEQMU2_RS13805	BK815_RS09330	A6V26_RS05150	AA913_RS02005	-	-	-
lantibiotic ABC transporter ATP-binding protein|Uncharacterized ABC transporter ATP-binding protein YdbJ	-	-	-	SEQMU2_RS13810	BK815_RS09335	A6V26_RS05155	AA913_RS02010	-	-	-
hypothetical protein	-	-	-	SEQMU2_RS13815	-	-	-	SXYL_RS11165	BE24_RS00595	-
membrane protein	-	-	-	SEQMU2_RS13825	BK815_RS03830	A6V26_RS00020	AA913_RS03915	-	-	-
hypothetical protein	-	-	-	SEQMU2_RS13830	-	A6V26_RS13270	AA913_RS12945	-	-	-
transposase|Putative transposon Tn552 DNA-invert ase bin3	-	-	-	SEQMU2_RS13880	-	A6V26_RS13330	AA913_RS12895	-	-	-
hypothetical protein	-	-	-	SEQMU2_RS13895	-	A6V26_RS12955	AA913_RS04380	-	-	-
acyl-CoA thioester hydrolase|Uncharacterized acy l-CoA thioester hydrolase YkhA	-	-	-	SEQMU2_RS13900	-	A6V26_RS12950	AA913_RS04385	-	-	-
transposase|Transposon Tn917 resolvase	-	-	-	SEQMU2_RS13910	-	A6V26_RS12855	AA913_RS04475	-	-	-
transposase IDENTICAL PARALOGS:	-	-	-	SEQMU2_RS13915	-	-	-	-	BE24_RS03755	-
transposase IDENTICAL PARALOGS:	-	-	-	-	-	-	-	-	BE24_RS06535	-
transposase	-	-	-	-	-	-	-	-	BE24_RS09275	-
Rossman fold protein, TIGR00730 family	-	-	SE1039_RS05905	SEQMU2_RS13930	-	-	-	-	-	-
restriction endonuclease subunit M|Type I restri ction enzyme EcoR124II M protein	-	-	-	SEQMU2_RS13945	-	A6V26_RS05580	AA913_RS10645	-	-	-

Product	*S. carnosus*		*S. equorum*		*S. succinus*			*S. xylosus*		*S. saprophyticus*
	**JCM 6069**	**TM300**	**KS1039**	**Mu2**	**14BME20**	**CSM 77**	**DSM 14617**	**C2a**	**HKUOPL8**	**ATCC 15305**
hypothetical protein	-	-	-	SEQMU2_RS13950	-	A6V26_RS05575	AA913_RS10650	-	-	-
type I restriction endonuclease subunit R|Type-1 restriction enzyme R protein	-	-	-	SEQMU2_RS13955	-	A6V26_RS05570	AA913_RS10655	-	-	-
partitioning protein	-	-	-	SEQMU2_RS13985	-	A6V26_RS12750	AA913_RS04320	-	-	-
plasmid replication initiator protein	-	-	-	SEQMU2_RS13990	-	A6V26_RS12745	AA913_RS04325	-	-	-
protein rep|Replication protein	-	-	-	SEQMU2_RS13995	-	A6V26_RS13515	AA913_RS14395	-	-	-
hypothetical protein	-	-	-	SEQMU2_RS14000	BK815_RS06335	A6V26_RS13000	AA913_RS04335	-	-	-
hypothetical protein	-	-	-	SEQMU2_RS14010	-	A6V26_RS12985	AA913_RS04350	-	-	-
hypothetical protein	-	-	SE1039_RS05700	SEQMU2_RS10550	BK815_RS04195	A6V26_RS00355	AA913_RS03580	SXYL_RS07680	BE24_RS03765	SSP_RS07315
Rha family transcriptional regulator	-	-	-	SEQMU2_RS10680	BK815_RS02680	-	-	-	-	-
phage protein	-	-	-	SEQMU2_RS10735	-	-	-	SXYL_RS08725	-	-
hypothetical protein	-	-	-	SEQMU2_RS10795	BK815_RS02805	-	-	SXYL_RS07115	-	-
BCCT transporter	-	-	-	SEQMU2_RS11245	BK815_RS09025	A6V26_RS04860	AA913_RS07280	SXYL_RS07225	BE24_RS04210	SSP_RS06835
phage integrase|Integrase	-	-	-	SEQMU2_RS11385	-	-	-	SXYL_RS07065	-	-
protein ArsC 1	-	SCA_RS03405	SE1039_RS03990	SEQMU2_RS09205	BK815_RS00715	A6V26_RS12800	AA913_RS04530	-	-	SSP_RS08230
NINE protein	-	SCA_RS03300	SE1039_RS04000	SEQMU2_RS09210	BK815_RS05580	A6V26_RS01715	AA913_RS13100	SXYL_RS09320	BE24_RS02410	SSP_RS08805
pathogenicity island protein	-	-	-	SEQMU2_RS08265	-	-	-	-	-	SSP_RS09695
membrane protein	-	-	-	SEQMU2_RS08340	BK815_RS03905	A6V26_RS13295	AA913_RS12970	-	-	-
transcriptional regulator	-	-	-	SEQMU2_RS08220	BK815_RS09955	-	-	-	-	-
aldo/keto reductase|Pyridoxal 4-dehydrogenase	-	-	-	SEQMU2_RS06730	BK815_RS09360	A6V26_RS05220	AA913_RS02075	-	-	-
3-hexulose-6-phosphate synthase	-	-	SE1039_RS02035	SEQMU2_RS06820	-	-	AA913_RS12920	SXYL_RS11205	BE24_RS00565	SSP_RS10740
6-phospho-3-hexuloisomerase	-	-	SE1039_RS02040	SEQMU2_RS06825	BK815_RS07460	A6V26_RS03585	-	SXYL_RS11200	BE24_RS00570	-
hypothetical protein	-	-	-	SEQMU2_RS07815	BK815_RS03815	-	-	-	-	-
hypothetical protein	-	-	-	SEQMU2_RS07825	-	-	AA913_RS06390	-	-	-
hypothetical protein	-	-	-	SEQMU2_RS07880	-	A6V26_RS11220	-	-	-	-
phage protein	-	-	-	SEQMU2_RS07895	BK815_RS02725	A6V26_RS11195	AA913_RS06345	-	-	-
hypothetical protein	-	-	-	SEQMU2_RS07920	-	A6V26_RS11180	AA913_RS06330	-	-	-
hypothetical protein	-	-	-	SEQMU2_RS07935	BK815_RS02750	A6V26_RS11170	AA913_RS06320	-	BE24_RS05210	-
hypothetical protein	-	-	-	SEQMU2_RS07940	-	A6V26_RS13510	AA913_RS14305	-	BE24_RS13615	-
hypothetical protein	-	-	-	SEQMU2_RS08085	BK815_RS02885	-	-	-	-	-
hypothetical protein	-	-	-	SEQMU2_RS08090	BK815_RS02890	-	-	-	-	-
YolD-like family protein	-	-	-	SEQMU2_RS06480	-	-	AA913_RS06135	SXYL_RS08480	BE24_RS05040	SSP_RS09665

Product	*S. carnosus*		*S. equorum*		*S. succinus*			*S. xylosus*		*S. saprophyticus*
	**JCM 6069**	**TM300**	**KS1039**	**Mu2**	**14BME20**	**CSM 77**	**DSM 14617**	**C2a**	**HKUOPL8**	**ATCC 15305**
hypothetical protein|DNA topoisomerase 1	-	-	-	SEQMU2_RS06500	-	A6V26_RS11265	-	-	-	-
transporter	-	-	-	SEQMU2_RS05810	-	-	-	SXYL_RS12515	BE24_RS11540	-
site-specific integrase	-	-	-	SEQMU2_RS06425	-	-	-	-	BE24_RS12845	-
hypothetical protein	-	-	-	SEQMU2_RS06440	-	-	AA913_RS13640	-	BE24_RS12860	-
hypothetical protein	-	-	-	SEQMU2_RS06445	-	-	-	-	BE24_RS12865	SSP_RS09730
hypothetical protein	-	-	-	SEQMU2_RS06450	-	-	-	-	-	SSP_RS09725
hypothetical protein	-	-	-	SEQMU2_RS06460	-	-	-	-	BE24_RS12880	-
recombinase family protein	-	-	-	SEQMU2_RS05420	-	-	-	-	BE24_RS11880	-
hypothetical protein	-	-	-	SEQMU2_RS05165	-	-	-	-	BE24_RS11900	SSP_RS00220
hypothetical protein	-	-	-	SEQMU2_RS05170	-	-	-	-	-	SSP_RS00215
alpha-acetolactate decarboxylase	-	-	-	SEQMU2_RS05245	-	-	-	-	-	SSP_RS00425
acetolactate synthase AlsS	-	-	-	SEQMU2_RS05250	-	-	-	-	-	SSP_RS00420
malate:quinone oxidoreductase	-	-	-	SEQMU2_RS05255	-	-	-	-	-	SSP_RS00415
hypothetical protein	-	-	-	SEQMU2_RS05260	-	-	-	-	-	SSP_RS00410
hypothetical protein	-	-	-	SEQMU2_RS05270	-	-	-	-	BE24_RS11705	-
hypothetical protein	-	-	-	SEQMU2_RS05325	-	A6V26_RS13485	AA913_RS14180	-	-	-
restriction endonuclease subunit R|Type-1 restri ction enzyme R protein	-	-	-	SEQMU2_RS05330	-	-	-	SXYL_RS00155	-	-
glycosyltransferase family 1 protein	-	-	-	SEQMU2_RS05355	-	-	-	-	BE24_RS11770	-
hypothetical protein	-	-	-	SEQMU2_RS05410	BK815_RS09695	-	-	-	BE24_RS11865	SSP_RS00170
hypothetical protein	-	-	-	SEQMU2_RS05415	-	-	-	-	BE24_RS11870	SSP_RS00175
hypothetical protein	-	-	-	SEQMU2_RS05030	-	-	-	-	-	SSP_RS09670
XRE family transcriptional regulator	-	-	-	SEQMU2_RS05055	BK815_RS03920	A6V26_RS13415	AA913_RS14255	-	BE24_RS06340	SSP_RS09660
phage head-tail adapter protein	-	-	-	SEQMU2_RS05075	-	-	-	-	BE24_RS12925	-
hypothetical protein	-	-	-	SEQMU2_RS04935	BK815_RS09705	-	-	-	-	-
restriction endonuclease S subunit	-	-	-	SEQMU2_RS04995	-	-	-	-	-	SSP_RS12635
hypothetical protein	-	-	-	SEQMU2_RS03625	-	-	AA913_RS08705	SXYL_RS12720	-	-
hypothetical protein	-	-	-	SEQMU2_RS04030	-	-	-	-	-	SSP_RS00785
aldehyde dehydrogenase	-	-	SE1039_RS12190	SEQMU2_RS04045	BK815_RS10445	A6V26_RS06285	AA913_RS12640	SXYL_RS00845	BE24_RS10855	-
hypothetical protein	-	-	-	SEQMU2_RS04050	-	-	AA913_RS12635	-	BE24_RS10865	-
hypothetical protein	-	-	-	SEQMU2_RS04410	-	-	-	-	-	SSP_RS00565
transcriptional regulator	-	-	-	SEQMU2_RS04415	-	-	-	-	-	SSP_RS00560

Product	*S. carnosus*		*S. equorum*		*S. succinus*			*S. xylosus*		*S. saprophyticus*
	**JCM 6069**	**TM300**	**KS1039**	**Mu2**	**14BME20**	**CSM 77**	**DSM 14617**	**C2a**	**HKUOPL8**	**ATCC 15305**
GntR family transcriptional regulator	-	-	-	SEQMU2_RS02440	BK815_RS11995	A6V26_RS07345	AA913_RS01045	SXYL_RS02585	BE24_RS09190	-
PTS beta-glucoside transporter subunit EIIBCA	-	-	-	SEQMU2_RS02445	BK815_RS11990	A6V26_RS07350	AA913_RS01050	SXYL_RS02580	BE24_RS09195	-
transglycosylase SceD 1	-	-	SE1039_RS09075	SEQMU2_RS00950	-	-	-	-	-	SSP_RS00580
oxidoreductase	-	SCA_RS08370	SE1039_RS09335	SEQMU2_RS01210	BK815_RS00065	A6V26_RS12050	AA913_RS11630	SXYL_RS03860	BE24_RS07900	SSP_RS03640
hypothetical protein	-	-	-	SEQMU2_RS00305	-	-	-	-	BE24_RS12870	-
hypothetical protein	-	-	-	-	BK815_RS09690	-	-	-	-	SSP_RS00160
LLM class flavin-dependent oxidoreductase	-	-	-	-	BK815_RS09910	A6V26_RS05775	AA913_RS10450	SXYL_RS00315	-	SSP_RS00460
transglycosylase	-	-	-	-	-	-	AA913_RS10320	SXYL_RS00565	BE24_RS07645	SSP_RS00585
dihydrofolate reductase	-	-	-	-	-	-	-	SXYL_RS00575	BE24_RS11110	SSP_RS00590
VOC family protein	-	-	-	-	-	-	-	SXYL_RS00580	-	SSP_RS00595
hypothetical protein	-	-	-	-	-	-	-	SXYL_RS09325	-	SSP_RS00645
hypothetical protein	-	-	-	SEQMU2_RS04040	-	-	-	-	-	SSP_RS00775
LysR family transcriptional regulator	-	-	-	-	BK815_RS10475	A6V26_RS08870	AA913_RS09745	-	-	SSP_RS00820
MFS transporter	-	-	SE1039_RS11885	SEQMU2_RS03730	BK815_RS10765	A6V26_RS08600	AA913_RS10015	SXYL_RS01130	BE24_RS10570	SSP_RS01035
succinyl-diaminopimelate desuccinylase	-	-	-	-	BK815_RS10795	A6V26_RS08570	AA913_RS10045	SXYL_RS01180	BE24_RS10540	SSP_RS01090
TetR/AcrR family transcriptional regulator	-	-	-	-	-	-	-	SXYL_RS01190	-	SSP_RS01105
CPBP family intramembrane metalloprotease	-	-	-	-	-	-	-	SXYL_RS01340	BE24_RS10415	SSP_RS01205
TetR family transcriptional regulator	-	-	-	-	-	-	-	SXYL_RS01405	BE24_RS10340	SSP_RS01245
amino acid permease	-	-	-	-	-	-	-	SXYL_RS01495	BE24_RS10250	SSP_RS01335
hypothetical protein	-	-	SE1039_RS11440	SEQMU2_RS03295	BK815_RS11130	A6V26_RS08235	AA913_RS04015	SXYL_RS01585	BE24_RS10160	SSP_RS01425
MFS transporter	-	-	SE1039_RS11310	-	BK815_RS11235	A6V26_RS08130	AA913_RS01835	SXYL_RS01720	BE24_RS10055	SSP_RS01535
transporter	-	-	-	-	BK815_RS11395	A6V26_RS07965	AA913_RS01670	SXYL_RS01905	BE24_RS09860	SSP_RS01735
energy-coupling factor transporter transmembrane protein EcfT	-	-	-	-	BK815_RS11400	A6V26_RS07960	AA913_RS01665	SXYL_RS01910	BE24_RS09855	SSP_RS01740
ABC transporter	-	-	-	-	BK815_RS11405	A6V26_RS07955	AA913_RS01660	SXYL_RS01915	BE24_RS09850	SSP_RS01745
NAD(P)-dependent alcohol dehydrogenase	-	-	-	-	-	-	-	SXYL_RS01940	-	SSP_RS01785
anion permease	-	-	-	-	BK815_RS11535	A6V26_RS07805	AA913_RS01510	SXYL_RS02070	BE24_RS09715	SSP_RS01930
2-deoxy-D-gluconate 3-dehydrogenase	-	-	-	-	-	-	-	SXYL_RS02160	BE24_RS09625	SSP_RS12660
sodium:proton antiporter	-	-	-	-	BK815_RS11900	A6V26_RS07440	AA913_RS01145	SXYL_RS02475	BE24_RS09310	SSP_RS02315
heat-shock protein	-	-	-	-	BK815_RS12020	A6V26_RS07320	AA913_RS01020	SXYL_RS02675	BE24_RS09100	SSP_RS02455
hypothetical protein	-	-	-	-	-	-	-	SXYL_RS02945	-	SSP_RS02715
ribulokinase	-	-	-	-	BK815_RS12290	A6V26_RS07055	AA913_RS00755	SXYL_RS02965	BE24_RS08840	SSP_RS02735

Product	*S. carnosus*		*S. equorum*		*S. succinus*			*S. xylosus*		*S. saprophyticus*
	**JCM 6069**	**TM300**	**KS1039**	**Mu2**	**14BME20**	**CSM 77**	**DSM 14617**	**C2a**	**HKUOPL8**	**ATCC 15305**
alpha/beta hydrolase	-	-	SE1039_RS09580	SEQMU2_RS01440	BK815_RS12915	A6V26_RS06435	AA913_RS00135	SXYL_RS03615	BE24_RS08190	SSP_RS03395
cardiolipin synthase	-	SCA_RS08070	SE1039_RS09040	SEQMU2_RS00915	BK815_RS00355	A6V26_RS11755	AA913_RS11335	SXYL_RS04145	BE24_RS07610	SSP_RS03930
hypothetical proteinIDENTICAL PARALOGS:	-	-	-	-	-	-	-	-	BE24_RS03750	SSP_RS04480
hypothetical proteinIDENTICAL PARALOGS:	-	-	-	-	-	-	-	-	BE24_RS06540	-
hypothetical protein	-	-	-	-	-	-	-	-	BE24_RS09270	-
teichoic acid ABC transporter permease	-	-	-	-	BK815_RS01240	A6V26_RS11435	AA913_RS12140	SXYL_RS05035	BE24_RS06510	SSP_RS04845
hypothetical protein	-	-	-	-	-	-	-	SXYL_RS07405	BE24_RS04030	SSP_RS07015
DNA-binding response regulator	-	-	-	-	-	-	-	-	BE24_RS03735	SSP_RS07335
sensor histidine kinase	-	-	-	-	-	-	-	-	BE24_RS03730	SSP_RS07340
ABC transporter permease	-	-	-	-	-	-	-	-	BE24_RS03725	SSP_RS07345
ABC transporter ATP-binding protein	-	-	-	-	-	-	-	-	BE24_RS03720	SSP_RS07350
hemolytic protein	-	-	SE1039_RS04570	SEQMU2_RS09745	-	-	-	-	-	SSP_RS08090
YolD-like family protein	-	-	-	-	-	-	-	SXYL_RS08480	BE24_RS05040	SSP_RS08135
3-hexulose-6-phosphate synthase 1	-	-	-	SEQMU2_RS14205	-	A6V26_RS13320	-	-	-	SSP_RS08165
oxidoreductase	-	-	-	-	BK815_RS09175	A6V26_RS05000	AA913_RS10295	-	-	SSP_RS08210
NAD(P)-dependent alcohol dehydrogenase	-	-	-	-	BK815_RS11065	A6V26_RS08300	AA913_RS04080	-	-	SSP_RS08235
MarR family transcriptional regulator	-	-	-	-	BK815_RS10395	A6V26_RS06235	AA913_RS12690	-	-	SSP_RS08240
pyridine nucleotide-disulfide oxidoreductase	-	-	-	-	BK815_RS10390	A6V26_RS06230	AA913_RS12695	-	-	SSP_RS08245
lipase	-	-	-	-	-	A6V26_RS05070	AA913_RS01925	-	-	SSP_RS08255
site-specific integrase	-	-	SE1039_RS04520	SEQMU2_RS07810	-	-	-	-	-	SSP_RS08270
osmotically inducible protein C	-	-	-	-	-	-	-	SXYL_RS07710	BE24_RS03705	SSP_RS08810
CsbD family protein	-	-	-	-	-	-	-	SXYL_RS10035	BE24_RS01690	SSP_RS09510
DinB family protein	-	-	-	-	BK815_RS06355	A6V26_RS02500	AA913_RS08425	-	-	SSP_RS09540
hypothetical protein	-	-	-	-	BK815_RS06460	A6V26_RS02585	AA913_RS08345	SXYL_RS10165	BE24_RS01570	SSP_RS09625
AraC family transcriptional regulator	-	-	-	-	-	-	-	SXYL_RS10695	BE24_RS01035	SSP_RS10290
6-phospho-3-hexuloisomerase	-	-	-	-	BK815_RS07460	A6V26_RS03585	AA913_RS11185	SXYL_RS11200	BE24_RS00570	SSP_RS10735
elongation factor G-binding protein	-	-	-	-	-	-	-	SXYL_RS11295	BE24_RS00475	SSP_RS10825
gamma-aminobutyrate permease	-	-	-	-	-	A6V26_RS08265	-	SXYL_RS11350	BE24_RS00420	SSP_RS10850
hypothetical protein	-	-	-	-	-	-	-	SXYL_RS11395	BE24_RS00375	SSP_RS10880
dehydrogenase	-	-	-	-	-	-	-	SXYL_RS11405	BE24_RS00365	SSP_RS10890
DeoR/GlpR transcriptional regulator	-	-	-	-	-	-	-	SXYL_RS11415	BE24_RS00355	SSP_RS10900

Product	*S. carnosus*		*S. equorum*		*S. succinus*			*S. xylosus*		*S. saprophyticus*
	**JCM 6069**	**TM300**	**KS1039**	**Mu2**	**14BME20**	**CSM 77**	**DSM 14617**	**C2a**	**HKUOPL8**	**ATCC 15305**
gluconate transporter	-	-	-	-	BK815_RS07630	-	-	SXYL_RS11485	BE24_RS00260	SSP_RS10940
membrane protein	-	-	-	-	BK815_RS05025	A6V26_RS01185	AA913_RS02745	SXYL_RS05305	BE24_RS06320	SSP_RS10955
hypothetical protein	-	-	-	-	BK815_RS07685	A6V26_RS03805	AA913_RS10965	SXYL_RS11565	BE24_RS00170	SSP_RS11015
GrpB family protein	-	-	-	-	BK815_RS08360	A6V26_RS04195	AA913_RS06615	-	BE24_RS12965	SSP_RS11620
DUF4889 domain-containing protein	-	-	-	-	BK815_RS08470	A6V26_RS04305	AA913_RS06725	SXYL_RS12450	BE24_RS12740	SSP_RS11775
N-acetyltransferase	-	-	-	-	BK815_RS08500	A6V26_RS04335	AA913_RS06755	SXYL_RS12480	BE24_RS12710	SSP_RS11810
hypothetical protein	-	-	-	-	-	-	-	SXYL_RS12485	-	SSP_RS11815
hypothetical protein	-	-	-	-	BK815_RS08510	A6V26_RS04345	AA913_RS06765	SXYL_RS12500	BE24_RS12695	SSP_RS11830
hypothetical protein	-	-	-	-	BK815_RS04890	A6V26_RS01050	AA913_RS02880	-	-	SSP_RS11850
Lrp/AsnC family transcriptional regulator	-	-	-	-	BK815_RS04895	A6V26_RS01055	AA913_RS02875	-	-	SSP_RS11855
hypothetical protein	-	-	-	-	-	-	-	SXYL_RS10055	BE24_RS01660	SSP_RS11885
transcriptional regulator	-	-	-	-	-	A6V26_RS05595	AA913_RS10630	SXYL_RS12670	-	SSP_RS12625
DUF805 domain-containing protein	-	-	-	-	-	-	-	SXYL_RS12660	-	SSP_RS11990
nitric oxide dioxygenase	-	-	SE1039_RS00395	SEQMU2_RS05900	BK815_RS08790	A6V26_RS04625	AA913_RS07045	SXYL_RS12910	-	SSP_RS12100
transcriptional regulator	-	-	-	-	-	-	-	-	BE24_RS12265	SSP_RS12160
transcriptional regulator	-	-	SE1039_RS00195	-	-	-	-	-	-	SSP_RS12195
hypothetical protein	-	-	-	SEQMU2_RS00595	BK815_RS00685	A6V26_RS12530	AA913_RS10180	SXYL_RS04475	BE24_RS07260	SSP_RS04290
CDP-glycerol:glycerophosphate glycerophosphotransferase	-	-	-	-	BK815_RS01235	A6V26_RS11440	AA913_RS12135	-	-	-
proline dehydrogenase	-	-	-	-	BK815_RS01255	A6V26_RS11420	AA913_RS12355	-	-	-
hypothetical protein	-	-	-	-	BK815_RS01260	A6V26_RS11415	AA913_RS12360	-	-	-
hypothetical protein	-	-	-	-	BK815_RS01405	A6V26_RS10980	AA913_RS06115	-	-	-
hypothetical protein	-	-	-	-	BK815_RS01410	A6V26_RS10975	AA913_RS06110	-	-	-
MFS transporter	-	-	-	-	BK815_RS02040	A6V26_RS10330	AA913_RS05470	-	-	-
hypothetical protein	-	-	-	-	BK815_RS02070	A6V26_RS10300	AA913_RS05440	-	-	-
N-acetyltransferase	-	-	-	-	BK815_RS02100	A6V26_RS10270	AA913_RS05410	-	-	-
N-acetyltransferase	-	-	-	-	BK815_RS02105	A6V26_RS10265	AA913_RS05405	-	-	-
hypothetical protein	-	-	-	-	BK815_RS02110	A6V26_RS10260	AA913_RS05400	-	-	-
hypothetical protein	-	-	-	-	BK815_RS02745	A6V26_RS11175	AA913_RS06325	-	-	-
hypothetical protein	-	-	-	-	BK815_RS02755	-	-	SXYL_RS08690	-	-
competence protein ComGA	-	-	SE1039_RS06860	SEQMU2_RS11970	BK815_RS02945	A6V26_RS09750	AA913_RS04895	SXYL_RS06470	BE24_RS04880	SSP_RS06165
hypothetical protein	-	-	-	-	BK815_RS03440	A6V26_RS09255	AA913_RS09390	-	-	-

Product	*S. carnosus*		*S. equorum*		*S. succinus*			*S. xylosus*		*S. saprophyticus*
	**JCM 6069**	**TM300**	**KS1039**	**Mu2**	**14BME20**	**CSM 77**	**DSM 14617**	**C2a**	**HKUOPL8**	**ATCC 15305**
FMN-dependent NADH-azoreductase	-	-	-	-	BK815_RS03775	A6V26_RS08920	AA913_RS13400	SXYL_RS11305	BE24_RS00465	-
transcriptional regulator	-	-	-	-	BK815_RS03780	A6V26_RS08915	AA913_RS13405	SXYL_RS11300	BE24_RS00470	-
hypothetical protein	-	-	-	-	BK815_RS03800	A6V26_RS08910	AA913_RS13410	-	-	-
hypothetical protein	-	-	-	-	BK815_RS03805	A6V26_RS08905	AA913_RS13415	-	-	-
hypothetical protein	-	-	-	-	BK815_RS03825	A6V26_RS00015	AA913_RS03920	-	-	-
hypothetical protein	-	-	-	-	BK815_RS03845	A6V26_RS00025	AA913_RS03910	-	-	-
MarR family transcriptional regulator	-	-	-	-	BK815_RS03850	A6V26_RS00030	AA913_RS03905	SXYL_RS13015	-	-
MFS transporter	-	-	-	-	BK815_RS03855	A6V26_RS00035	AA913_RS03900	-	-	-
N-acetyltransferase	-	-	-	-	BK815_RS03860	-	-	SXYL_RS07425	BE24_RS04025	-
metallothiol transferase FosB	-	-	-	-	BK815_RS03885	-	-	-	-	SSP_RS09650
hypothetical protein	-	-	-	SEQMU2_RS06540	BK815_RS03910	A6V26_RS00050	AA913_RS03885	-	-	-
hypothetical protein	-	-	-	-	BK815_RS03930	A6V26_RS00065	AA913_RS03870	-	-	-
hypothetical protein	-	-	-	-	BK815_RS03935	A6V26_RS00070	AA913_RS03865	-	-	-
MFS transporter	-	-	-	-	BK815_RS03950	A6V26_RS00095	AA913_RS03840	-	-	-
alpha-N-arabinofuranosidase	-	-	-	-	BK815_RS03955	A6V26_RS00100	AA913_RS03835	-	-	-
hypothetical protein	-	-	-	-	BK815_RS04220	A6V26_RS00380	AA913_RS03555	-	-	-
NINE protein	-	-	-	-	BK815_RS04780	A6V26_RS00940	AA913_RS02990	-	-	-
esterase	-	-	-	-	BK815_RS04785	A6V26_RS00945	AA913_RS02985	-	-	-
hypothetical protein	-	-	-	-	BK815_RS04790	A6V26_RS00950	AA913_RS02980	-	-	-
phenol soluble modulin	-	-	SE1039_RS04570	SEQMU2_RS09745	BK815_RS05010	A6V26_RS01170	AA913_RS02760	SXYL_RS08455	BE24_RS02955	-
hypothetical protein	-	-	-	-	BK815_RS05020	A6V26_RS01180	AA913_RS02750	-	BE24_RS01655	-
hypothetical protein	-	-	-	-	BK815_RS05045	A6V26_RS01190	AA913_RS02740	-	-	-
hypothetical protein	-	-	-	-	BK815_RS06150	A6V26_RS02295	AA913_RS08625	-	-	-
N-acetyltransferase	-	-	-	-	BK815_RS06155	A6V26_RS02300	AA913_RS08620	-	-	-
lysophospholipase	-	-	SE1039_RS03230	SEQMU2_RS08440	BK815_RS06285	A6V26_RS02430	AA913_RS08490	SXYL_RS10015	BE24_RS01710	SSP_RS09495
YfcC family protein	-	-	-	-	BK815_RS06295	A6V26_RS02440	AA913_RS08480	SXYL_RS12755	BE24_RS12475	-
DoxX family protein	-	-	-	-	BK815_RS06300	A6V26_RS02445	AA913_RS08475	-	-	-
CHAP domain-containing protein	-	-	-	-	BK815_RS06305	A6V26_RS02450	AA913_RS08470	SXYL_RS10040	BE24_RS01675	-
GlsB/YeaQ/YmgE family stress response membrane protein	-	-	SE1039_RS03995	-	BK815_RS06310	A6V26_RS02455	AA913_RS08465	SXYL_RS05255	BE24_RS02940	-
PLP-dependent aminotransferase family protein	-	-	-	-	BK815_RS06315	A6V26_RS02465	AA913_RS08460	-	-	-
EamA family transporter	-	-	-	-	BK815_RS06320	A6V26_RS02470	AA913_RS08455	-	-	-

Product	*S. carnosus*		*S. equorum*		*S. succinus*			*S. xylosus*		*S. saprophyticus*
	**JCM 6069**	**TM300**	**KS1039**	**Mu2**	**14BME20**	**CSM 77**	**DSM 14617**	**C2a**	**HKUOPL8**	**ATCC 15305**
hypothetical protein	-	-	-	-	BK815_RS06345	A6V26_RS02490	AA913_RS08435	-	-	-
ATP-binding protein	-	-	-	-	BK815_RS06350	A6V26_RS02495	AA913_RS08430	SXYL_RS10045	BE24_RS01670	-
damage-inducible protein DinB	-	-	-	-	BK815_RS06370	-	-	SXYL_RS10070	-	-
SAM-dependent methyltransferase	-	-	-	-	BK815_RS06475	A6V26_RS02605	AA913_RS08325	-	BE24_RS13130	-
hypothetical protein	-	-	-	-	BK815_RS07275	A6V26_RS03405	AA913_RS07530	-	-	-
hypothetical protein	-	-	-	-	BK815_RS07625	A6V26_RS03750	AA913_RS11020	-	-	-
N-acetyltransferase	-	-	SE1039_RS01125	SEQMU2_RS06710	BK815_RS08160	A6V26_RS04005	AA913_RS06430	SXYL_RS12035	BE24_RS13240	SSP_RS11485
alanine--glyoxylate aminotransferase family protein	-	-	-	-	BK815_RS08275	A6V26_RS04115	AA913_RS06535	SXYL_RS12180	BE24_RS13090	-
allantoinase	-	-	-	-	BK815_RS08345	A6V26_RS04180	AA913_RS06600	SXYL_RS12285	BE24_RS12980	-
MFS transporter	-	-	-	-	BK815_RS08365	A6V26_RS04200	AA913_RS06620	SXYL_RS12305	BE24_RS12960	SSP_RS11625
hypothetical protein	-	-	SE1039_RS00745	SEQMU2_RS06270	BK815_RS08515	A6V26_RS04350	AA913_RS06770	SXYL_RS12505	BE24_RS12690	SSP_RS11840
thioesterase	-	-	-	-	BK815_RS08525	A6V26_RS04360	AA913_RS06780	SXYL_RS12565	BE24_RS12605	-
hypothetical protein	-	-	-	-	BK815_RS08530	A6V26_RS04365	AA913_RS14155	SXYL_RS13225	BE24_RS13535	-
non-ribosomal peptide synthetase	-	-	-	-	BK815_RS08535	A6V26_RS04370	AA913_RS06790	SXYL_RS12575	BE24_RS12595	-
AraC family transcriptional regulator	-	-	-	-	BK815_RS08595	A6V26_RS04430	AA913_RS06850	SXYL_RS12625	BE24_RS12545	-
alpha-glucosidase/alpha-galactosidase	-	-	-	-	BK815_RS08600	A6V26_RS04435	AA913_RS06855	SXYL_RS12630	BE24_RS12540	-
melibiose:sodium transporter MelB	-	-	-	-	BK815_RS08605	A6V26_RS04440	AA913_RS06860	SXYL_RS12635	BE24_RS12535	-
LysR family transcriptional regulator	-	-	-	-	BK815_RS08650	A6V26_RS04485	AA913_RS06905	-	-	-
glyoxalase/bleomycin resistance/dioxygenase family protein	-	-	-	-	BK815_RS08660	A6V26_RS04495	AA913_RS06915	-	-	-
8-amino-7-oxononanoate synthase	-	-	-	-	BK815_RS08675	A6V26_RS04510	AA913_RS06930	-	-	-
6-carboxyhexanoate--CoA ligase	-	-	-	-	BK815_RS08680	A6V26_RS04515	AA913_RS06935	-	-	-
choline transporter	-	-	-	-	BK815_RS08690	A6V26_RS04525	AA913_RS06945	-	-	-
MFS transporter	-	-	-	-	BK815_RS08720	A6V26_RS04555	AA913_RS06975	SXYL_RS12830	BE24_RS12395	-
hypothetical protein	-	-	-	-	BK815_RS08855	A6V26_RS04690	AA913_RS07110	-	-	-
hypothetical protein	-	-	-	-	BK815_RS08860	A6V26_RS04695	AA913_RS07115	-	-	-
nuclear transport factor 2 family protein	-	-	-	-	BK815_RS08870	A6V26_RS04705	AA913_RS07125	SXYL_RS00385	BE24_RS11325	-
MarR family transcriptional regulator	-	-	-	-	BK815_RS08885	A6V26_RS04720	AA913_RS07140	-	-	-
ferrichrome ABC transporter substrate-binding protein	-	-	-	-	BK815_RS08900	A6V26_RS04735	AA913_RS07155	SXYL_RS13115	BE24_RS12130	-
hypothetical protein	-	-	-	-	BK815_RS08910	A6V26_RS04745	AA913_RS07165	SXYL_RS13100	BE24_RS12155	-
hypothetical protein	-	-	-	-	BK815_RS08915	A6V26_RS04750	AA913_RS07170	SXYL_RS13095	BE24_RS12160	-
hypothetical protein	-	-	-	-	BK815_RS08920	A6V26_RS04755	AA913_RS07175	-	-	-

Product	*S. carnosus*		*S. equorum*		*S. succinus*			*S. xylosus*		*S. saprophyticus*
	**JCM 6069**	**TM300**	**KS1039**	**Mu2**	**14BME20**	**CSM 77**	**DSM 14617**	**C2a**	**HKUOPL8**	**ATCC 15305**
gfo/Idh/MocA family oxidoreductase	-	-	-	-	BK815_RS08925	A6V26_RS04760	AA913_RS07180	-	-	-
6-phospho-5-dehydro-2-deoxy-D-gluconate aldolase	-	-	-	-	BK815_RS08930	A6V26_RS04765	AA913_RS07185	SXYL_RS00185	-	-
myo-inosose-2 dehydratase	-	-	-	-	BK815_RS08935	A6V26_RS04770	AA913_RS07190	-	-	-
5-dehydro-2-deoxygluconokinase	-	-	-	-	BK815_RS08940	A6V26_RS04775	AA913_RS07195	-	-	-
5-deoxy-glucuronate isomerase	-	-	-	-	BK815_RS08945	A6V26_RS04780	AA913_RS07200	-	-	-
methylmalonate-semialdehyde dehydrogenase (CoA acylating)	-	-	-	-	BK815_RS08950	A6V26_RS04785	AA913_RS07205	-	-	-
DeoR/GlpR transcriptional regulator	-	-	-	-	BK815_RS08955	A6V26_RS04790	AA913_RS07210	-	-	-
isomerase	-	-	-	-	BK815_RS08960	A6V26_RS04795	AA913_RS07215	-	-	-
protein iolH	-	-	-	-	BK815_RS08965	A6V26_RS04800	AA913_RS07220	-	-	-
3D-(3,5/4)-trihydroxycyclohexane-1,2-dione acylhydrolase (decyclizing)	-	-	-	-	BK815_RS08970	A6V26_RS04805	AA913_RS07225	-	-	-
sugar porter family MFS transporter	-	-	-	-	BK815_RS08975	A6V26_RS04810	AA913_RS07230	-	-	-
CoA transferase	-	-	-	-	BK815_RS08980	A6V26_RS04815	AA913_RS07235	SXYL_RS12775	BE24_RS12455	-
TetR family transcriptional regulator	-	-	-	-	BK815_RS08985	A6V26_RS04820	AA913_RS07240	SXYL_RS12780	BE24_RS13525	-
ATP-grasp domain-containing protein	-	-	-	-	BK815_RS09020	A6V26_RS04855	AA913_RS07275	-	-	-
LLM class flavin-dependent oxidoreductase	-	-	-	-	BK815_RS09030	A6V26_RS04865	AA913_RS07285	SXYL_RS11310	BE24_RS00460	-
signal peptidase I	-	-	-	-	BK815_RS09035	A6V26_RS04870	AA913_RS07290	SXYL_RS11170	-	-
MFS transporter	-	-	-	-	BK815_RS09040	A6V26_RS04875	AA913_RS07295	SXYL_RS12770	BE24_RS12460	-
hypothetical protein	-	-	-	-	BK815_RS09045	A6V26_RS04880	AA913_RS07300	SXYL_RS12765	BE24_RS12465	-
carbonyl reductase	-	-	-	-	BK815_RS09050	A6V26_RS04885	AA913_RS07305	SXYL_RS05300	BE24_RS06330	-
hypothetical protein	-	-	-	-	BK815_RS09055	A6V26_RS04890	AA913_RS07310	-	-	-
MFS transporter	-	-	-	-	BK815_RS09090	A6V26_RS04925	AA913_RS07345	-	-	-
sucrose-6-phosphate hydrolase	-	-	-	-	BK815_RS09095	A6V26_RS04930	AA913_RS07350	-	-	-
LacI family transcriptional regulator	-	-	-	-	BK815_RS09100	A6V26_RS04935	AA913_RS07355	-	-	-
MFS transporter	-	-	-	-	BK815_RS09105	A6V26_RS04940	AA913_RS07360	-	-	-
hypothetical protein	-	-	-	-	BK815_RS09115	A6V26_RS04950	AA913_RS07370	-	-	-
agmatinase	-	-	-	-	BK815_RS09120	A6V26_RS04955	AA913_RS07375	-	-	-
sodium:solute symporter	-	-	-	-	BK815_RS09125	A6V26_RS04960	AA913_RS07380	-	-	-
N-acetyl-L,L-diaminopimelate aminotransferase	-	-	-	-	BK815_RS09130	A6V26_RS04965	AA913_RS07385	-	-	-
hypothetical protein	-	-	-	-	BK815_RS09135	A6V26_RS04970	-	-	-	-
hypothetical protein	-	-	-	-	BK815_RS09145	A6V26_RS13455	AA913_RS14350	-	-	-
D-Ala-D-Ala carboxypeptidase	-	-	-	-	BK815_RS09150	A6V26_RS04975	AA913_RS10270	SXYL_RS12270	BE24_RS12995	-

Product	*S. carnosus*		*S. equorum*		*S. succinus*			*S. xylosus*		*S. saprophyticus*
	**JCM 6069**	**TM300**	**KS1039**	**Mu2**	**14BME20**	**CSM 77**	**DSM 14617**	**C2a**	**HKUOPL8**	**ATCC 15305**
hypothetical protein	-	-	-	-	BK815_RS09180	A6V26_RS05005	AA913_RS10300	-	-	-
glycerol dehydrogenase	-	-	-	-	BK815_RS09255	A6V26_RS05055	AA913_RS01910	-	-	-
iron citrate ABC transporter substrate-binding protein	-	-	-	-	BK815_RS09260	A6V26_RS05060	AA913_RS01915	-	-	SSP_RS00900
hypothetical protein	-	-	-	-	BK815_RS09265	A6V26_RS05065	AA913_RS01920	-	-	-
ATP-grasp domain-containing protein	-	-	-	-	BK815_RS09295	A6V26_RS05100	AA913_RS01955	-	-	-
hypothetical protein	-	-	-	-	BK815_RS09300	A6V26_RS05105	AA913_RS01960	-	-	-
hypothetical protein	-	-	-	-	BK815_RS09305	A6V26_RS05110	AA913_RS01965	-	-	-
beta-ketoacyl-[acyl-carrier-protein] synthase II	-	-	-	-	BK815_RS09310	A6V26_RS05115	AA913_RS01970	-	-	-
hypothetical protein	-	-	-	-	BK815_RS09325	A6V26_RS05145	AA913_RS02000	-	-	-
cold-shock protein	-	-	-	-	BK815_RS09355	A6V26_RS05215	AA913_RS02070	-	-	-
SDR family oxidoreductase	-	-	-	-	BK815_RS09390	A6V26_RS05275	AA913_RS02130	-	-	-
Rrf2 family transcriptional regulator	-	-	-	-	BK815_RS09395	A6V26_RS05280	AA913_RS02135	-	-	-
alpha/beta hydrolase	-	-	-	-	BK815_RS09405	A6V26_RS05290	AA913_RS02145	-	-	-
alpha/beta hydrolase	-	-	-	-	BK815_RS09415	A6V26_RS05300	AA913_RS02155	-	-	-
Lrp/AsnC family transcriptional regulator	-	-	-	-	BK815_RS09420	A6V26_RS05305	AA913_RS02160	-	-	-
hypothetical protein	-	-	-	-	BK815_RS09430	A6V26_RS05315	AA913_RS02170	-	-	-
6-phospho-3-hexuloisomerase	-	-	-	-	BK815_RS09445	A6V26_RS05330	AA913_RS02185	-	-	-
3-hexulose-6-phosphate synthase	-	-	-	-	BK815_RS09450	A6V26_RS05335	AA913_RS02190	-	-	-
MFS transporter	-	-	-	-	BK815_RS09460	A6V26_RS05345	AA913_RS02200	-	-	-
SDR family oxidoreductase	-	-	-	-	BK815_RS09470	A6V26_RS05355	AA913_RS02210	SXYL_RS00200	BE24_RS11505	-
hypothetical protein	-	-	-	-	BK815_RS09475	A6V26_RS05360	AA913_RS02215	-	-	-
hypothetical protein	-	-	-	-	BK815_RS09480	A6V26_RS05365	AA913_RS02220	SXYL_RS00445	BE24_RS11290	-
aldo/keto reductase	-	-	-	-	BK815_RS09485	A6V26_RS05370	AA913_RS02225	-	-	-
endonuclease	-	-	-	-	BK815_RS09755	-	-	SXYL_RS00165	-	-
hypothetical protein	-	-	-	-	BK815_RS09760	-	-	SXYL_RS00170	-	-
hypothetical protein	-	-	-	-	BK815_RS09785	A6V26_RS05645	AA913_RS10580	-	-	-
MFS transporter	-	-	-	-	BK815_RS09790	A6V26_RS05650	AA913_RS10575	SXYL_RS00465	BE24_RS11270	-
PadR family transcriptional regulator	-	-	-	-	BK815_RS09795	A6V26_RS05655	AA913_RS10570	SXYL_RS00910	-	-
tetracycline resistance protein	-	-	-	-	BK815_RS09800	A6V26_RS05660	AA913_RS10565	SXYL_RS00915	-	-
epidermal surface antigen	-	-	-	-	BK815_RS09805	A6V26_RS05665	AA913_RS10560	SXYL_RS12135	BE24_RS13135	-
type 1 glutamine amidotransferase	-	-	-	-	BK815_RS09825	A6V26_RS05690	AA913_RS10535	-	-	-

Product	*S. carnosus*		*S. equorum*		*S. succinus*			*S. xylosus*		*S. saprophyticus*
	**JCM 6069**	**TM300**	**KS1039**	**Mu2**	**14BME20**	**CSM 77**	**DSM 14617**	**C2a**	**HKUOPL8**	**ATCC 15305**
transcriptional regulator	-	-	-	-	BK815_RS09830	A6V26_RS05695	AA913_RS10530	-	-	-
transcriptional regulator	-	-	-	-	BK815_RS09835	A6V26_RS05700	AA913_RS10525	-	-	-
aldehyde dehydrogenase	-	-	-	-	BK815_RS09840	A6V26_RS05705	AA913_RS10520	-	-	-
non-ribosomal peptide synthetase	-	-	-	-	BK815_RS09870	A6V26_RS05735	AA913_RS10490	-	BE24_RS12150	-
solute:sodium symporter family transporter	-	-	-	-	BK815_RS09915	A6V26_RS05780	AA913_RS10445	SXYL_RS00320	BE24_RS12095	-
TetR/AcrR family transcriptional regulator	-	-	-	-	BK815_RS09920	A6V26_RS05785	AA913_RS10440	-	-	-
gfo/Idh/MocA family oxidoreductase	-	-	-	-	BK815_RS09975	A6V26_RS05815	AA913_RS10410	-	-	-
hypothetical protein	-	-	-	-	BK815_RS09980	A6V26_RS05820	AA913_RS10405	-	-	-
MFS transporter	-	-	-	-	BK815_RS09985	A6V26_RS05825	AA913_RS10400	-	-	-
alcohol dehydrogenase	-	-	-	-	BK815_RS10005	A6V26_RS05845	AA913_RS10380	-	-	-
transglycosylase	-	-	-	-	BK815_RS10075	A6V26_RS05915	AA913_RS12480	-	-	-
MerR family transcriptional regulator	-	-	-	-	BK815_RS10090	A6V26_RS05930	AA913_RS12495	-	-	-
quercetin 2,3-dioxygenase	-	-	-	-	BK815_RS10100	A6V26_RS05940	AA913_RS12505	-	-	-
GntR family transcriptional regulator	-	-	-	-	BK815_RS10250	A6V26_RS06090	AA913_RS12770	SXYL_RS00765	BE24_RS10920	-
tartronate semialdehyde reductase	-	-	-	-	BK815_RS10255	A6V26_RS06095	AA913_RS12775	SXYL_RS00770	BE24_RS10915	-
hypothetical protein	-	-	-	-	BK815_RS10260	A6V26_RS06100	AA913_RS12780	SXYL_RS00775	BE24_RS10910	-
gluconate permease	-	-	-	-	BK815_RS10265	A6V26_RS06105	AA913_RS12785	SXYL_RS00780	BE24_RS10905	-
nitrilotriacetate monooxygenase	-	-	-	-	BK815_RS10270	A6V26_RS06110	AA913_RS12790	-	-	-
AP endonuclease	-	-	-	-	BK815_RS10280	A6V26_RS06120	AA913_RS12800	-	-	-
hypothetical protein	-	-	-	-	BK815_RS10285	A6V26_RS06125	AA913_RS12805	-	-	-
ribokinase	-	-	-	-	BK815_RS10290	A6V26_RS06130	AA913_RS12810	-	-	-
PadR family transcriptional regulator	-	-	-	-	BK815_RS10300	A6V26_RS06140	AA913_RS12820	-	-	-
DUF3237 domain-containing protein	-	-	-	-	BK815_RS10310	A6V26_RS06150	AA913_RS12830	-	-	-
antibiotic biosynthesis monooxygenase	-	-	-	-	BK815_RS10330	A6V26_RS06170	AA913_RS12850	SXYL_RS11160	BE24_RS00600	-
ABC transporter ATP-binding protein	-	-	-	-	BK815_RS10335	A6V26_RS06175	AA913_RS12855	-	-	-
hypothetical protein	-	-	-	-	BK815_RS10340	A6V26_RS06180	AA913_RS12860	-	-	-
ABC transporter permease	-	-	-	-	BK815_RS10345	A6V26_RS06185	AA913_RS12865	-	-	-
peptide ABC transporter substrate-binding protein	-	-	-	-	BK815_RS10350	A6V26_RS06190	AA913_RS12870	-	-	-
gamma-glutamyltransferase	-	-	-	-	BK815_RS10355	A6V26_RS06195	AA913_RS12730	-	-	-
hypothetical protein	-	-	-	-	BK815_RS10360	A6V26_RS06200	AA913_RS12725	-	-	-
hypothetical protein	-	-	-	-	BK815_RS10365	A6V26_RS06205	AA913_RS12720	-	-	-

Product	*S. carnosus*		*S. equorum*		*S. succinus*			*S. xylosus*		*S. saprophyticus*
	**JCM 6069**	**TM300**	**KS1039**	**Mu2**	**14BME20**	**CSM 77**	**DSM 14617**	**C2a**	**HKUOPL8**	**ATCC 15305**
aldehyde dehydrogenase family protein	-	-	-	-	BK815_RS10385	A6V26_RS06225	AA913_RS12700	SXYL_RS12785	BE24_RS12445	-
alanine:cation symporter family protein	-	-	-	-	BK815_RS10410	A6V26_RS06250	AA913_RS12675	SXYL_RS00800	-	-
hypothetical protein	-	-	-	-	BK815_RS10440	A6V26_RS06280	AA913_RS12645	-	-	-
hypothetical protein	-	-	-	-	BK815_RS10450	A6V26_RS06290	AA913_RS12635	-	-	-
membrane protein	-	-	-	-	BK815_RS10510	A6V26_RS08855	AA913_RS09760	SXYL_RS00895	BE24_RS10805	-
gamma-aminobutyrate permease	-	-	-	-	BK815_RS10615	A6V26_RS08750	AA913_RS09865	-	-	-
GbsR/MarR family transcriptional regulator	-	-	SE1039_RS11940	SEQMU2_RS03785	BK815_RS10715	A6V26_RS08650	AA913_RS09965	SXYL_RS01090	BE24_RS10620	SSP_RS00960
endonuclease	-	-	-	-	BK815_RS10835	A6V26_RS08530	AA913_RS10085	-	-	-
type I pantothenate kinase	-	-	-	-	BK815_RS10855	A6V26_RS08510	AA913_RS10105	-	-	-
MBL fold metallo-hydrolase	-	-	-	-	BK815_RS11020	A6V26_RS08345	AA913_RS04125	SXYL_RS01500	BE24_RS10245	-
MFS transporter	-	-	-	-	BK815_RS11050	A6V26_RS08315	AA913_RS04095	SXYL_RS00440	BE24_RS11295	-
HXXEE domain-containing protein	-	-	-	-	BK815_RS11055	A6V26_RS08310	AA913_RS04090	-	-	-
transcriptional regulator	-	-	-	-	BK815_RS11070	A6V26_RS08295	AA913_RS04075	-	-	-
multidrug resistance efflux transporter family protein	-	-	-	-	BK815_RS11095	A6V26_RS08270	AA913_RS04050	SXYL_RS01550	BE24_RS10195	-
gamma-aminobutyrate permease	-	-	-	-	BK815_RS11100	A6V26_RS08265	AA913_RS04045	SXYL_RS01555	BE24_RS10190	-
thiol reductase thioredoxin	-	-	SE1039_RS11200	SEQMU2_RS03075	BK815_RS11340	A6V26_RS08025	AA913_RS01730	SXYL_RS01835	BE24_RS09930	SSP_RS01655
phosphoesterase	-	-	-	-	BK815_RS11425	A6V26_RS07935	AA913_RS01640	-	-	-
N-acetyltransferase	-	-	-	-	BK815_RS11660	A6V26_RS07680	AA913_RS01385	SXYL_RS02235	BE24_RS09550	-
short-chain dehydrogenase	-	-	-	-	BK815_RS11740	A6V26_RS07600	AA913_RS01305	SXYL_RS02315	BE24_RS09470	SSP_RS02125
aryl-phospho-beta-D-glucosidase|6-phospho-beta- g lucosidase BglA	-	-	-	SEQMU2_RS02450	BK815_RS11985	A6V26_RS07355	AA913_RS01055	SXYL_RS02575	-	-
N-acetyltransferase	-	-	SE1039_RS10365	SEQMU2_RS02225	BK815_RS12155	A6V26_RS07190	AA913_RS00890	SXYL_RS02800	BE24_RS08975	SSP_RS02590
hypothetical protein	-	-	-	-	BK815_RS12160	A6V26_RS07185	AA913_RS00885	-	-	-
carbon-nitrogen hydrolase family protein	-	-	-	-	BK815_RS13000	A6V26_RS06350	AA913_RS00050	-	-	-
glucose-6-phosphate isomerase	-	-	-	-	-	A6V26_RS13250	AA913_RS12925	-	-	-
phosphogluconate dehydrogenase (NADP(+)-dependent, decarboxylating)	-	-	-	-	-	A6V26_RS13255	AA913_RS12930	-	-	-
glucose-6-phosphate dehydrogenase	-	-	-	-	-	A6V26_RS13260	AA913_RS12935	-	-	-
flavodoxin family protein	-	-	-	-	-	A6V26_RS13265	AA913_RS12940	-	-	-
hypothetical protein	-	-	-	-	-	A6V26_RS13285	AA913_RS12960	-	-	-
N-acetyltransferase	-	-	-	-	-	A6V26_RS13290	AA913_RS12965	-	-	-
hypothetical protein	-	-	-	-	-	A6V26_RS13540	AA913_RS14390	-	-	-
replication protein	-	-	-	-	-	A6V26_RS13545	AA913_RS14395	-	-	-

Product	*S. carnosus*		*S. equorum*		*S. succinus*			*S. xylosus*		*S. saprophyticus*
	**JCM 6069**	**TM300**	**KS1039**	**Mu2**	**14BME20**	**CSM 77**	**DSM 14617**	**C2a**	**HKUOPL8**	**ATCC 15305**
6-phospho-3-hexuloisomerase	-	-	SE1039_RS02040	SEQMU2_RS14200	-	A6V26_RS13325	AA913_RS12890	-	-	SSP_RS08160
DUF536 domain-containing protein	-	-	-	-	-	A6V26_RS13550	AA913_RS14385	-	-	-
transposase	-	-	-	-	-	A6V26_RS13335	AA913_RS12900	-	-	-
transcriptional regulator	-	-	-	-	-	A6V26_RS13345	AA913_RS12910	-	-	-
transcriptional regulator	-	-	-	-	-	A6V26_RS12810	AA913_RS04520	-	-	-
copper-translocating P-type ATPase	-	-	-	-	-	A6V26_RS12815	AA913_RS04515	-	-	-
cadmium resistance transporter	-	-	-	-	-	A6V26_RS12820	AA913_RS04510	-	-	-
hypothetical protein	-	-	-	-	-	A6V26_RS13520	AA913_RS14280	-	-	-
hypothetical protein	-	-	-	-	-	A6V26_RS12830	AA913_RS04500	-	-	-
Hg(II)-responsive transcriptional regulator	-	-	-	-	-	A6V26_RS12835	AA913_RS04495	-	-	-
mercury(II) reductase	-	-	-	-	-	A6V26_RS12845	AA913_RS04485	-	-	-
hypothetical protein	-	-	-	-	-	A6V26_RS12850	AA913_RS04480	-	-	-
cytoplasmic protein	-	-	SE1039_RS12435	SEQMU2_RS04230	-	A6V26_RS12870	AA913_RS04460	-	-	-
four-helix bundle copper-binding protein	-	-	-	-	-	A6V26_RS12885	AA913_RS04445	-	-	-
multicopper oxidase	-	-	-	-	-	A6V26_RS12895	AA913_RS04435	-	-	-
XRE family transcriptional regulator	-	-	-	-	-	A6V26_RS12905	AA913_RS04425	-	-	-
hypothetical protein	-	-	-	-	-	A6V26_RS12910	AA913_RS14275	-	-	-
hypothetical protein	-	-	-	-	-	A6V26_RS13525	AA913_RS14130	-	-	-
Crp/Fnr family transcriptional regulator	-	-	-	-	-	A6V26_RS12920	AA913_RS04415	-	-	-
DNA starvation/stationary phase protection protein	-	-	-	-	-	A6V26_RS12925	AA913_RS04410	-	-	-
copper chaperone	-	-	-	-	-	A6V26_RS12930	AA913_RS04405	-	-	-
heavy metal translocating P-type ATPase	-	-	-	-	-	A6V26_RS12935	AA913_RS04400	-	-	-
hypothetical protein	-	-	-	-	-	A6V26_RS12965	AA913_RS04370	-	-	-
hypothetical protein	-	-	-	-	-	A6V26_RS12970	AA913_RS04365	-	-	-
hypothetical protein	-	-	-	-	-	A6V26_RS12975	AA913_RS04360	-	-	-
ArsR family transcriptional regulator	-	-	-	-	BK815_RS00725	A6V26_RS12485	AA913_RS10230	-	-	-
arsenical efflux pump membrane protein ArsB	-	SCA_RS03410	-	-	BK815_RS00720	A6V26_RS12490	AA913_RS10225	-	-	-
hypothetical protein	-	-	-	-	BK815_RS00620	A6V26_RS12595	-	-	-	-
hypothetical protein	-	-	-	-	-	A6V26_RS10785	AA913_RS14295	-	-	-
hypothetical protein	-	-	-	-	-	A6V26_RS10790	AA913_RS14135	-	-	-
hypothetical protein	-	-	-	-	-	A6V26_RS10795	AA913_RS14140	-	-	-

Product	*S. carnosus*		*S. equorum*		*S. succinus*			*S. xylosus*		*S. saprophyticus*
	**JCM 6069**	**TM300**	**KS1039**	**Mu2**	**14BME20**	**CSM 77**	**DSM 14617**	**C2a**	**HKUOPL8**	**ATCC 15305**
hypothetical protein	-	-	-	-	-	A6V26_RS11005	AA913_RS06150	SXYL_RS05235	-	-
DUF2479 domain-containing protein	-	-	-	-	-	A6V26_RS11015	-	SXYL_RS13205	-	-
peptidase G2	-	-	-	-	-	A6V26_RS11020	-	SXYL_RS08535	-	-
hypothetical protein	-	-	-	-	-	A6V26_RS11040	AA913_RS14150	-	BE24_RS13455	-
hypothetical protein	-	-	-	-	-	A6V26_RS11045	AA913_RS06195	-	BE24_RS05100	-
hypothetical protein	-	-	-	-	-	A6V26_RS11050	AA913_RS06200	-	BE24_RS05105	-
hypothetical protein	-	-	-	-	-	A6V26_RS11055	AA913_RS06205	-	BE24_RS05110	-
hypothetical protein	-	-	-	-	-	A6V26_RS11060	AA913_RS06210	-	BE24_RS05115	-
head-tail adaptor protein	-	-	-	-	-	A6V26_RS11065	AA913_RS06215	-	BE24_RS05120	-
hypothetical protein	-	-	-	-	-	A6V26_RS11070	AA913_RS06220	SXYL_RS08600	BE24_RS05125	-
major capsid protein	-	-	-	-	-	A6V26_RS11075	AA913_RS06225	SXYL_RS08605	BE24_RS05130	-
peptidase U35	-	-	-	-	-	A6V26_RS11080	AA913_RS06230	SXYL_RS08610	BE24_RS05135	-
phage portal protein	-	-	-	-	-	A6V26_RS11085	AA913_RS06235	SXYL_RS08615	BE24_RS05140	-
hypothetical protein	-	-	-	-	-	A6V26_RS11090	AA913_RS06240	SXYL_RS08620	-	-
terminase	-	-	-	-	-	A6V26_RS11095	AA913_RS06245	SXYL_RS08625	BE24_RS05145	-
terminase	-	-	-	-	-	A6V26_RS11100	AA913_RS06250	SXYL_RS08630	BE24_RS05150	-
HNH endonuclease	-	-	-	-	-	A6V26_RS11105	AA913_RS06255	SXYL_RS08635	BE24_RS05155	-
hypothetical protein	-	-	-	-	-	A6V26_RS11115	AA913_RS06265	-	-	-
DUF4870 domain-containing protein	-	-	-	-	-	A6V26_RS11125	AA913_RS06275	-	-	-
hypothetical protein	-	-	-	-	-	A6V26_RS11135	AA913_RS06285	-	-	-
hypothetical protein	-	-	-	-	-	A6V26_RS11140	AA913_RS06290	-	-	-
hypothetical protein	-	-	-	-	-	A6V26_RS11145	AA913_RS06295	-	-	-
hypothetical protein	-	-	-	-	-	A6V26_RS11150	AA913_RS06300	-	-	-
hypothetical protein	-	-	-	-	-	A6V26_RS11160	AA913_RS06310	-	-	-
hypothetical protein	-	-	-	SEQMU2_RS07875	-	A6V26_RS11225	-	SXYL_RS08755	BE24_RS05280	-
transcriptional regulator	-	-	-	-	-	A6V26_RS11260	-	-	BE24_RS05305	-
hypothetical protein	-	-	-	-	-	A6V26_RS12040	AA913_RS11620	-	-	-
hypothetical protein	-	-	-	-	-	A6V26_RS07860	AA913_RS01565	-	-	-
PTS beta-glucoside transporter subunit EIIBCA	-	-	-	-	-	A6V26_RS07865	AA913_RS01570	-	-	-
6-phospho-beta-glucosidase	-	-	-	-	-	A6V26_RS07870	AA913_RS01575	-	-	-
6-phospho-beta-glucosidase	-	-	-	-	-	A6V26_RS07875	AA913_RS01580	-	-	SSP_RS00615

Product	*S. carnosus*		*S. equorum*		*S. succinus*			*S. xylosus*		*S. saprophyticus*
	**JCM 6069**	**TM300**	**KS1039**	**Mu2**	**14BME20**	**CSM 77**	**DSM 14617**	**C2a**	**HKUOPL8**	**ATCC 15305**
hypothetical protein	-	-	-	-	BK815_RS09080	A6V26_RS04915	AA913_RS07335	-	BE24_RS11675	-
copper-translocating P-type ATPase	-	-	-	-	-	A6V26_RS05025	AA913_RS01880	-	-	-
peptidase M48	-	-	-	-	-	A6V26_RS05040	AA913_RS01895	-	-	-
hypothetical protein	-	-	-	-	BK815_RS09285	A6V26_RS05090	AA913_RS01945	-	-	-
transcriptional regulator	-	-	-	-	-	A6V26_RS13460	AA913_RS14230	-	-	-
PhzF family phenazine biosynthesis protein	-	-	-	-	-	A6V26_RS05170	AA913_RS02025	-	-	-
2-dehydro-3-deoxy-phosphogluconate aldolase	-	-	-	-	-	A6V26_RS05175	AA913_RS02030	-	-	-
6-phospho-3-hexuloisomerase	-	-	-	-	-	A6V26_RS05180	AA913_RS02035	-	-	-
3-hexulose-6-phosphate synthase	-	-	-	-	-	A6V26_RS05185	AA913_RS02040	-	-	-
sugar kinase	-	-	-	-	-	A6V26_RS05190	AA913_RS02045	-	-	-
gluconate:proton symporter	-	-	-	-	-	A6V26_RS05195	AA913_RS02050	-	-	-
hypothetical protein	-	-	-	-	-	A6V26_RS05200	AA913_RS02055	-	-	-
sugar phosphate isomerase/epimerase	-	-	-	-	-	A6V26_RS05205	AA913_RS02060	-	-	-
LacI family transcriptional regulator	-	-	-	-	-	A6V26_RS05210	AA913_RS02065	-	-	-
hypothetical protein	-	-	-	-	-	A6V26_RS05245	AA913_RS02100	-	-	-
Rrf2 family transcriptional regulator	-	-	-	-	-	A6V26_RS05250	AA913_RS02105	-	-	-
NADH-flavin reductase	-	-	-	-	-	A6V26_RS05255	AA913_RS02110	-	-	-
hypothetical protein	-	-	-	-	-	A6V26_RS05260	AA913_RS02115	-	-	-
hypothetical protein	-	-	-	-	-	A6V26_RS05265	AA913_RS02120	-	-	-
hypothetical protein	-	-	-	-	-	A6V26_RS13465	AA913_RS14235	-	-	-
transcriptional regulator	-	-	-	-	BK815_RS09455	A6V26_RS05340	AA913_RS02195	-	-	-
hypothetical protein	-	-	-	-	-	A6V26_RS13475	AA913_RS14360	-	-	-
hypothetical protein	-	-	-	-	-	A6V26_RS13480	AA913_RS14355	-	-	-
hypothetical protein	-	-	-	-	-	A6V26_RS05565	AA913_RS10660	-	-	-
restriction endonuclease subunit S	-	-	-	-	-	A6V26_RS05585	AA913_RS10640	-	-	-
hypothetical protein	-	-	-	-	-	A6V26_RS05590	AA913_RS10635	-	-	-
membrane protein	-	-	-	-	-	A6V26_RS05600	AA913_RS10625	SXYL_RS12665	-	-
hypothetical protein	-	-	-	-	-	A6V26_RS05605	AA913_RS10620	-	-	-
hypothetical protein	-	-	-	-	-	A6V26_RS05610	AA913_RS10615	SXYL_RS07435	BE24_RS04010	-
hypothetical protein	-	-	-	-	-	A6V26_RS05615	AA913_RS10610	SXYL_RS12695	-	-
class I SAM-dependent methyltransferase	-	-	-	-	-	A6V26_RS05620	AA913_RS10605	-	-	-

Product	*S. carnosus*		*S. equorum*		*S. succinus*			*S. xylosus*		*S. saprophyticus*
	**JCM 6069**	**TM300**	**KS1039**	**Mu2**	**14BME20**	**CSM 77**	**DSM 14617**	**C2a**	**HKUOPL8**	**ATCC 15305**
CPBP family intramembrane metalloprotease	-	-	-	-	-	A6V26_RS05670	AA913_RS10555	-	-	-
hypothetical protein	-	-	-	-	-	A6V26_RS05675	AA913_RS10550	-	-	-
CPBP family intramembrane metalloprotease	-	-	-	-	-	A6V26_RS05805	AA913_RS10420	-	-	-
glycosyl transferase family 1	-	-	-	-	-	A6V26_RS00005	AA913_RS03930	-	-	-
hypothetical protein	-	-	-	-	-	A6V26_RS00040	AA913_RS03895	-	-	-
hypothetical protein	-	-	-	-	-	A6V26_RS00055	AA913_RS03880	-	-	-
rhodanese-like domain-containing protein	-	-	-	-	-	A6V26_RS00080	AA913_RS03855	-	-	-
MarR family transcriptional regulator	-	-	-	-	-	A6V26_RS00085	AA913_RS03850	-	-	-
hypothetical protein	-	-	-	-	-	A6V26_RS00105	AA913_RS03830	-	-	-
hypothetical protein	-	-	-	-	-	A6V26_RS00110	AA913_RS03825	-	-	-
hypothetical protein	-	-	-	-	-	A6V26_RS00115	AA913_RS03820	-	-	-
sulfite exporter TauE/SafE family protein	-	-	-	-	BK815_RS04235	A6V26_RS00395	AA913_RS03535	-	-	-
dihydroneopterin aldolase	-	-	-	-	BK815_RS04245	A6V26_RS00405	AA913_RS03525	-	-	-
MBL fold metallo-hydrolase	-	-	-	-	BK815_RS04250	A6V26_RS00410	AA913_RS03520	-	-	-
CDP-glycerol:glycerophosphate glycerophosphotransferase	-	-	-	-	-	A6V26_RS02600	AA913_RS08330	-	-	-
hypothetical protein	-	-	-	-	-	A6V26_RS03360	AA913_RS07575	-	-	-
hypothetical protein	-	-	-	-	-	A6V26_RS03365	AA913_RS07570	-	-	-
3-hexulose-6-phosphate synthase	-	-	-	-	BK815_RS07465	A6V26_RS03590	AA913_RS11180	SXYL_RS11205	BE24_RS00565	SSP_RS10740
glycine oxidase	-	-	SE1039_RS12295	SEQMU2_RS04155	BK815_RS10220	A6V26_RS06060	AA913_RS12740	SXYL_RS00700	BE24_RS10980	-
zinc ribbon domain-containing protein	-	-	-	-	-	-	AA913_RS08710	SXYL_RS12715	-	-
phage holin	-	-	-	-	-	A6V26_RS11000	AA913_RS06145	SXYL_RS08510	-	-
hypothetical protein	-	-	-	-	BK815_RS02660	-	AA913_RS06400	-	-	-
hypothetical protein	-	-	SE1039_RS00595	SEQMU2_RS06100	-	A6V26_RS12900	AA913_RS04430	-	-	-
GTP pyrophosphokinase	-	SCA_RS09905	SE1039_RS10970	SEQMU2_RS02850	BK815_RS11600	A6V26_RS07740	AA913_RS01445	SXYL_RS02135	BE24_RS09650	SSP_RS01990
hypothetical protein	-	-	-	-	-	-	-	SXYL_RS00205	BE24_RS11500	-
flavin reductase	-	-	-	-	-	-	-	SXYL_RS00220	BE24_RS11485	-
hypothetical protein	-	-	-	-	-	-	-	SXYL_RS00225	BE24_RS11475	-
diaminopimelate epimerase	-	-	-	-	-	-	-	SXYL_RS00330	BE24_RS11380	-
ribosomal RNA methyltransferase FmrO domain protein	-	-	-	-	-	-	-	SXYL_RS00335	BE24_RS11375	-
hypothetical protein	-	-	-	-	-	-	-	SXYL_RS00340	BE24_RS11370	-
nickel ABC transporter nickel/metallophore periplasmic binding protein	-	-	-	-	-	-	-	SXYL_RS00345	BE24_RS11365	-

Product	*S. carnosus*		*S. equorum*		*S. succinus*			*S. xylosus*		*S. saprophyticus*
	**JCM 6069**	**TM300**	**KS1039**	**Mu2**	**14BME20**	**CSM 77**	**DSM 14617**	**C2a**	**HKUOPL8**	**ATCC 15305**
ABC transporter permease	-	-	-	-	-	-	-	SXYL_RS00350	BE24_RS11360	-
ABC transporter permease	-	-	-	-	-	-	-	SXYL_RS00355	BE24_RS11355	-
ABC transporter ATP-binding protein	-	-	-	-	-	-	-	SXYL_RS00360	BE24_RS11350	-
ABC transporter ATP-binding protein	-	-	-	-	-	-	-	SXYL_RS00365	BE24_RS11345	-
MFS transporter	-	-	-	-	-	-	-	SXYL_RS00370	BE24_RS11340	-
alpha/beta hydrolase	-	SCA_RS08620	-	-	-	-	-	SXYL_RS00375	BE24_RS11335	SSP_RS00470
TM2 domain-containing protein	-	-	-	-	-	-	-	SXYL_RS00380	BE24_RS11330	-
glyoxalase	-	-	-	-	-	-	-	SXYL_RS00535	BE24_RS11150	-
AraC family transcriptional regulator	-	-	-	-	-	-	-	SXYL_RS00680	BE24_RS11010	-
xylulokinase	-	SCA_RS10295	SE1039_RS12305	SEQMU2_RS04165	BK815_RS10210	A6V26_RS06050	AA913_RS12615	SXYL_RS00690	BE24_RS10990	-
hypothetical protein	-	-	-	-	-	-	-	SXYL_RS00745	BE24_RS10945	-
hypothetical protein	-	-	-	-	-	-	-	SXYL_RS00850	BE24_RS10850	-
IclR family transcriptional regulator	-	-	-	-	-	-	-	SXYL_RS01205	BE24_RS10520	-
gluconate permease	-	-	-	-	-	-	-	SXYL_RS01210	BE24_RS10515	-
alcohol dehydrogenase	-	-	-	-	-	-	-	SXYL_RS01215	BE24_RS10510	-
hypothetical protein	-	-	-	-	-	-	-	SXYL_RS01325	BE24_RS10430	-
MFS transporter	-	-	-	-	-	-	-	SXYL_RS01400	BE24_RS10345	-
transcriptional regulator	-	-	SE1039_RS11355	SEQMU2_RS03230	BK815_RS11195	A6V26_RS08170	AA913_RS03950	SXYL_RS01680	BE24_RS10095	SSP_RS01495
bifunctional 2-keto-4-hydroxyglutarate aldolase/2-keto-3-deoxy-6-phosphogluconate aldolase	-	-	-	-	-	-	-	SXYL_RS02195	BE24_RS09590	-
MFS transporter	-	-	-	-	-	-	-	SXYL_RS02200	BE24_RS09585	-
uronate isomerase	-	-	-	-	-	-	-	SXYL_RS02205	BE24_RS09580	-
lipase precursor	-	-	-	-	-	-	-	SXYL_RS13195	-	SSP_RS02445
DUF805 domain-containing protein	-	-	SE1039_RS10235	SEQMU2_RS02095	-	A6V26_RS07060	AA913_RS00760	SXYL_RS02960	BE24_RS08845	SSP_RS02730
hypothetical protein	-	-	-	-	-	-	-	SXYL_RS04720	BE24_RS07015	-
anion permease	-	-	-	-	-	-	-	SXYL_RS05025	BE24_RS06520	-
peptidase	-	SCA_RS02605	-	-	-	-	-	SXYL_RS05205	-	-
hypothetical protein	-	-	-	-	-	-	-	SXYL_RS05250	BE24_RS05050	-
signal peptidase	-	-	SE1039_RS07380	SEQMU2_RS12495	BK815_RS02060	A6V26_RS10310	AA913_RS05450	SXYL_RS05955	BE24_RS05685	SSP_RS05650
anion permease	-	-	-	-	-	-	-	SXYL_RS06200	BE24_RS05440	-
hypothetical protein	-	-	SE1039_RS06840	SEQMU2_RS11950	BK815_RS02965	A6V26_RS09730	AA913_RS04875	SXYL_RS06490	BE24_RS04860	SSP_RS06185
hypothetical protein	-	-	-	-	-	-	-	SXYL_RS07140	BE24_RS04295	-

Product	*S. carnosus*		*S. equorum*		*S. succinus*			*S. xylosus*		*S. saprophyticus*
	**JCM 6069**	**TM300**	**KS1039**	**Mu2**	**14BME20**	**CSM 77**	**DSM 14617**	**C2a**	**HKUOPL8**	**ATCC 15305**
hypothetical protein	-	-	-	-	-	-	-	SXYL_RS07500	BE24_RS03945	-
hypothetical protein	-	-	-	-	-	-	-	SXYL_RS07825	BE24_RS03595	-
GlsB/YeaQ/YmgE family stress response membrane protein	-	-	-	-	-	-	-	SXYL_RS08465	BE24_RS02940	-
hypothetical protein	-	-	-	-	-	A6V26_RS11120	AA913_RS06270	SXYL_RS08655	BE24_RS05170	-
hypothetical protein	-	-	-	SEQMU2_RS07965	-	-	-	SXYL_RS08670	-	-
hypothetical protein	-	-	-	-	-	-	-	SXYL_RS09835	BE24_RS01895	-
N-acetyltransferase	-	-	-	-	-	-	-	SXYL_RS09840	BE24_RS01890	-
hypothetical protein	-	-	-	-	-	-	-	SXYL_RS13330	BE24_RS13570	-
hypothetical protein	-	-	-	-	-	-	-	SXYL_RS11315	BE24_RS00455	-
C4-dicarboxylate ABC transporter permease	-	-	-	-	-	-	-	SXYL_RS11320	BE24_RS00450	-
hypothetical protein	-	-	-	-	-	-	-	SXYL_RS11325	BE24_RS00445	-
S-adenosylmethionine--2-demethylmenaquinone methyltransferase	-	-	-	-	-	-	-	SXYL_RS11330	BE24_RS00440	-
MurR/RpiR family transcriptional regulator	-	-	-	-	-	-	-	SXYL_RS11335	BE24_RS00435	-
YSIRK signal domain/LPXTG anchor domain surface protein	-	-	-	-	-	-	-	SXYL_RS11360	BE24_RS13450	-
hypothetical protein	-	-	-	-	-	-	-	SXYL_RS11390	BE24_RS00380	-
MFS transporter	-	-	-	-	-	-	-	SXYL_RS11410	BE24_RS00360	SSP_RS10895
hypothetical protein	-	-	-	-	-	-	-	SXYL_RS11510	BE24_RS00225	-
hypothetical protein	-	-	-	-	-	-	-	SXYL_RS12335	BE24_RS12950	-
hypothetical protein	-	-	-	-	-	-	-	SXYL_RS12385	BE24_RS13685	-
hypothetical protein	-	-	-	-	-	-	-	SXYL_RS12520	BE24_RS12675	-
hypothetical protein	-	-	-	-	-	-	-	SXYL_RS12525	BE24_RS12670	-
hypothetical protein	-	-	-	-	-	-	-	SXYL_RS12530	BE24_RS12665	-
hypothetical protein	-	-	-	-	-	-	-	SXYL_RS13220	BE24_RS13540	-
gluconate permease	-	-	-	-	-	-	-	SXYL_RS12640	BE24_RS12530	-
hypothetical protein	-	-	-	-	-	-	-	SXYL_RS12645	BE24_RS12525	-
2-hydroxy-3-oxopropionate reductase	-	-	-	-	-	-	-	SXYL_RS12650	BE24_RS12520	-
iron-containing alcohol dehydrogenase	-	-	-	-	-	-	-	SXYL_RS12680	BE24_RS12505	-
cell division protein FtsW	-	-	SE1039_RS00460	SEQMU2_RS05965	-	-	-	SXYL_RS12845	BE24_RS12380	-
magnesium transporter	-	-	-	-	-	-	-	SXYL_RS12995	BE24_RS12235	-
zinc ABC transporter substrate-binding protein	-	-	-	-	-	-	-	SXYL_RS13000	BE24_RS12230	-
metal ABC transporter permease	-	-	-	-	-	-	-	SXYL_RS13005	BE24_RS12225	-

Product	S. carnosus		S. equorum		S. succinus			S. xylosus		S. saprophyticus
	JCM 6069	TM300	KS1039	Mu2	14BME20	CSM 77	DSM 14617	C2a	HKUOPL8	ATCC 15305
manganese ABC transporter ATP-binding protein	-	-	-	-	-	-	-	SXYL_RS13010	BE24_RS12220	-
acyltransferase	-	-	-	-	-	-	-	SXYL_RS13040	BE24_RS12215	-
acyltransferase	-	-	-	-	-	-	-	SXYL_RS13075	BE24_RS12180	-
hypothetical protein	-	-	-	-	-	-	-	SXYL_RS13080	BE24_RS13515	-
CDP-glycerol--glycerophosphate glycerophosphotransferase	-	-	-	-	-	-	-	SXYL_RS13085	BE24_RS12170	-
CDP-glycerol:glycerophosphate glycerophosphotransferase	-	-	-	-	-	-	-	SXYL_RS13355	BE24_RS13670	-
MerR family transcriptional regulator	-	-	-	-	-	-	-	SXYL_RS13125	BE24_RS12110	SSP_RS12230
hypothetical protein	-	-	-	-	-	-	-	SXYL_RS09325	BE24_RS02405	-
phenol soluble modulin	-	-	-	-	-	-	-	SXYL_RS08445	BE24_RS02975	SSP_RS08085
hypothetical protein	-	-	-	-	-	-	-	SXYL_RS06260	BE24_RS13475	-
hypothetical protein	-	-	-	-	-	-	-	SXYL_RS05290	BE24_RS06350	-
transglycosylase	-	SCA_RS08120	-	-	-	-	-	SXYL_RS00570	BE24_RS11115	-
LysR family transcriptional regulator	-	-	-	-	-	-	-	SXYL_RS12535	BE24_RS12660	-
hypothetical protein	-	-	-	-	-	-	-	SXYL_RS12515	BE24_RS12680	-
hypothetical protein	-	-	-	-	-	-	-	-	BE24_RS12940	SSP_RS11890
hypothetical protein	BEK99_RS00005	-	-	-	-	-	-	-	-	-
hypothetical protein	BEK99_RS00100	-	-	-	-	-	-	-	-	-
hypothetical protein	BEK99_RS00105	-	-	-	-	-	-	-	-	-
hypothetical protein	BEK99_RS00115	-	-	-	-	-	-	-	-	-
hypothetical protein	BEK99_RS13015	-	-	-	-	-	-	-	-	-
hypothetical protein	BEK99_RS00160	-	-	-	-	-	-	-	-	-
hypothetical protein	BEK99_RS00170	-	-	-	-	-	-	-	-	-
hypothetical protein	BEK99_RS00175	-	-	-	-	-	-	-	-	-
hypothetical protein	BEK99_RS00180	-	-	-	-	-	-	-	-	-
DegT/DnrJ/EryC1/StrS family aminotransferase	BEK99_RS00345	-	-	-	-	-	-	-	-	-
low temperature requirement protein A	BEK99_RS00425	-	-	-	-	-	-	-	-	-
DNA integration/recombination/inversion protein	BEK99_RS00950	-	-	-	-	-	-	-	-	-
hypothetical protein	BEK99_RS01285	-	-	-	-	-	-	-	-	-
hypothetical protein	BEK99_RS01440	-	-	-	-	-	-	-	-	-
MMPL family protein	BEK99_RS01765	-	-	-	-	-	-	-	-	-
methionine ABC transporter substrate-binding protein	BEK99_RS03370	-	-	-	-	-	-	-	-	-

Product	*S. carnosus*		*S. equorum*		*S. succinus*			*S. xylosus*		*S. saprophyticus*
	**JCM 6069**	**TM300**	**KS1039**	**Mu2**	**14BME20**	**CSM 77**	**DSM 14617**	**C2a**	**HKUOPL8**	**ATCC 15305**
hypothetical protein	BEK99_RS03380	-	-	-	-	-	-	-	-	-
hypothetical protein	BEK99_RS13040	-	-	-	-	-	-	-	-	-
hypothetical protein	BEK99_RS04120	-	-	-	-	-	-	-	-	-
hypothetical protein	BEK99_RS04135	-	-	-	-	-	-	-	-	-
hypothetical protein	BEK99_RS04145	-	-	-	-	-	-	-	-	-
VRR-NUC domain-containing protein	BEK99_RS04155	-	-	-	-	-	-	-	-	-
hypothetical protein	BEK99_RS04160	-	-	-	-	-	-	-	-	-
hypothetical protein	BEK99_RS04165	-	-	-	-	-	-	-	-	-
hypothetical protein	BEK99_RS04180	-	-	-	-	-	-	-	-	-
hypothetical protein	BEK99_RS04185	-	-	-	-	-	-	-	-	-
hypothetical protein	BEK99_RS04190	-	-	-	-	-	-	-	-	-
hypothetical protein	BEK99_RS13045	-	-	-	-	-	-	-	-	-
hypothetical protein	BEK99_RS04215	-	-	-	-	-	-	-	-	-
hypothetical protein	BEK99_RS04220	-	-	-	-	-	-	-	-	-
hypothetical protein	BEK99_RS04240	-	-	-	-	-	-	-	-	-
hypothetical protein	BEK99_RS04245	-	-	-	-	-	-	-	-	-
hypothetical protein	BEK99_RS04250	-	-	-	-	-	-	-	-	-
peptidase	BEK99_RS04285	-	-	-	-	-	-	-	-	-
hypothetical protein	BEK99_RS04300	-	-	-	-	-	-	-	-	-
phage tail tape measure protein	BEK99_RS04330	-	-	-	-	-	-	-	-	-
hypothetical protein	BEK99_RS04345	-	-	-	-	-	-	-	-	-
hypothetical protein	BEK99_RS04350	-	-	-	-	-	-	-	-	-
hypothetical protein	BEK99_RS04355	-	-	-	-	-	-	-	-	-
hypothetical protein	BEK99_RS04370	-	-	-	-	-	-	-	-	-
hypothetical protein	BEK99_RS04385	-	-	-	-	-	-	-	-	-
hypothetical protein	BEK99_RS04390	-	-	-	-	-	-	-	-	-
hypothetical protein	BEK99_RS04400	-	-	-	-	-	-	-	-	-
hypothetical protein	BEK99_RS04405	-	-	-	-	-	-	-	-	-
hypothetical protein	BEK99_RS04505	-	-	-	-	-	-	-	-	-
ABC transporter ATP-binding protein	BEK99_RS06640	-	-	-	-	-	-	-	-	-
ABC transporter substrate-binding protein	BEK99_RS06925	-	-	-	-	-	-	-	-	-

Product	S. carnosus		S. equorum		S. succinus			S. xylosus		S. saprophyticus
	JCM 6069	TM300	KS1039	Mu2	14BME20	CSM 77	DSM 14617	C2a	HKUOPL8	ATCC 15305
ABC transporter ATP-binding protein	BEK99_RS06935	-	-	-	-	-	-	-	-	-
hypothetical protein	BEK99_RS06970	-	-	-	-	-	-	-	-	-
hypothetical protein	BEK99_RS06980	-	-	-	-	-	-	-	-	-
hypothetical protein	BEK99_RS07400	-	-	-	-	-	-	-	-	-
hypothetical protein	BEK99_RS13065	-	-	-	-	-	-	-	-	-
hypothetical protein	BEK99_RS08700	-	-	-	-	-	-	-	-	-
hypothetical protein	BEK99_RS08730	-	-	-	-	-	-	-	-	-
hypothetical protein	BEK99_RS08735	-	-	-	-	-	-	-	-	-
phage tail tape measure protein	BEK99_RS08755	-	-	-	-	-	-	-	-	-
phage gp6-like head-tail connector protein	BEK99_RS08790	-	-	-	-	-	-	-	-	-
phage major capsid protein	BEK99_RS08795	-	-	-	-	-	-	-	-	-
Clp protease ClpP	BEK99_RS08800	-	-	-	-	-	-	-	-	-
phage portal protein	BEK99_RS08805	-	-	-	-	-	-	-	-	-
terminase	BEK99_RS08810	-	-	-	-	-	-	-	-	-
terminase	BEK99_RS08815	-	-	-	-	-	-	-	-	-
hypothetical protein	BEK99_RS08820	-	-	-	-	-	-	-	-	-
hypothetical protein	BEK99_RS08830	-	-	-	-	-	-	-	-	-
hypothetical protein	BEK99_RS08835	-	-	-	-	-	-	-	-	-
hypothetical protein	BEK99_RS08840	-	-	-	-	-	-	-	-	-
dUTP pyrophosphatase	BEK99_RS08845	-	-	-	-	-	-	-	-	-
hypothetical protein	BEK99_RS08850	-	-	-	-	-	-	-	-	-
hypothetical protein	BEK99_RS08855	-	-	-	-	-	-	-	-	-
hypothetical protein	BEK99_RS08860	-	-	-	-	-	-	-	-	-
hypothetical protein	BEK99_RS08865	-	-	-	-	-	-	-	-	-
hypothetical protein	BEK99_RS08870	-	-	-	-	-	-	-	-	-
hypothetical protein	BEK99_RS08875	-	-	-	-	-	-	-	-	-
hypothetical protein	BEK99_RS08905	-	-	-	-	-	-	-	-	-
hypothetical protein	BEK99_RS08920	-	-	-	-	-	-	-	-	-
hypothetical protein	BEK99_RS08935	-	-	-	-	-	-	-	-	-
hypothetical protein	BEK99_RS08950	-	-	-	-	-	-	-	-	-
hypothetical protein	BEK99_RS08955	-	-	-	-	-	-	-	-	-

Product	*S. carnosus*		*S. equorum*		*S. succinus*			*S. xylosus*		*S. saprophyticus*
	**JCM 6069**	**TM300**	**KS1039**	**Mu2**	**14BME20**	**CSM 77**	**DSM 14617**	**C2a**	**HKUOPL8**	**ATCC 15305**
hypothetical protein	BEK99_RS08985	-	-	-	-	-	-	-	-	-
hypothetical protein	BEK99_RS10715	-	-	-	-	-	-	-	-	-
hypothetical protein	BEK99_RS13105	-	-	-	-	-	-	-	-	-
hypothetical protein	BEK99_RS11055	-	-	-	-	-	-	-	-	-
hypothetical protein	BEK99_RS13110	-	-	-	-	-	-	-	-	-
hypothetical protein	BEK99_RS11285	-	-	-	-	-	-	-	-	-
hypothetical protein	BEK99_RS11430	-	-	-	-	-	-	-	-	-
hypothetical protein	BEK99_RS11545	-	-	-	-	-	-	-	-	-
hypothetical protein	BEK99_RS11555	-	-	-	-	-	-	-	-	-
FRG domain-containing protein	BEK99_RS11605	-	-	-	-	-	-	-	-	-
dihydrolipoyl dehydrogenase	BEK99_RS11615	-	-	-	-	-	-	-	-	-
acetoin:2,6-dichlorophenolindophenol oxidoreductase subunit alpha	BEK99_RS11620	-	-	-	-	-	-	-	-	-
alpha-ketoacid dehydrogenase subunit beta	BEK99_RS11625	-	-	-	-	-	-	-	-	-
branched-chain alpha-keto acid dehydrogenase subunit E2	BEK99_RS11630	-	-	-	-	-	-	-	-	-
hypothetical protein	BEK99_RS11645	-	-	-	-	-	-	-	-	-
hypothetical protein	BEK99_RS11700	-	-	-	-	-	-	-	-	-
hypothetical protein	BEK99_RS11705	-	-	-	-	-	-	-	-	-
hypothetical protein	BEK99_RS11725	-	-	-	-	-	-	-	-	-
arsenate reductase	BEK99_RS11765	-	-	-	-	-	-	-	-	-
transcriptional regulator	BEK99_RS11775	-	-	-	-	-	-	-	-	-
hypothetical protein	BEK99_RS13125	-	-	-	-	-	-	-	-	-
hypothetical protein	BEK99_RS11980	-	-	-	-	-	-	-	-	-
hypothetical protein	BEK99_RS11985	-	-	-	-	-	-	-	-	-
hypothetical protein	BEK99_RS12015	-	-	-	-	-	-	-	-	-
macrolide ABC transporter ATP-binding protein	BEK99_RS12020	-	-	-	-	-	-	-	-	-
hypothetical protein	BEK99_RS12030	-	-	-	-	-	-	-	-	-
hypothetical protein	BEK99_RS13130	-	-	-	-	-	-	-	-	-
hypothetical protein	BEK99_RS13135	-	-	-	-	-	-	-	-	-
amino acid permease	BEK99_RS12180	-	-	-	-	-	-	-	-	-
hypothetical protein	BEK99_RS12235	-	-	-	-	-	-	-	-	-
hypothetical protein	BEK99_RS12410	-	-	-	-	-	-	-	-	-

Product	S. carnosus		S. equorum		S. succinus			S. xylosus		S. saprophyticus
	JCM 6069	TM300	KS1039	Mu2	14BME20	CSM 77	DSM 14617	C2a	HKUOPL8	ATCC 15305
hypothetical protein	BEK99_RS12415	-	-	-	-	-	-	-	-	-
hypothetical protein	BEK99_RS12605	-	-	-	-	-	-	-	-	-
poly(glycerol-phosphate) alpha-glucosyltransferase	BEK99_RS12615	-	-	-	-	-	-	-	-	-
polyphosphate--nucleotide phosphotransferase	BEK99_RS12745	-	-	-	-	-	-	-	-	-
hypothetical protein	BEK99_RS12750	-	-	-	-	-	-	-	-	-
3-beta hydroxysteroid dehydrogenase	BEK99_RS12755	-	-	-	-	-	-	-	-	-
hypothetical protein	BEK99_RS12760	-	-	-	-	-	-	-	-	-
amidohydrolase	BEK99_RS12765	-	-	-	-	-	-	-	-	-
hypothetical protein	BEK99_RS12775	-	-	-	-	-	-	-	-	-
hypothetical protein	BEK99_RS12795	-	-	-	-	-	-	-	-	-
hypothetical protein	BEK99_RS12815	-	-	-	-	-	-	-	-	-
DUF262 domain-containing protein	BEK99_RS12830	-	-	-	-	-	-	-	-	-
ATP-dependent exonuclease	BEK99_RS12835	-	-	-	-	-	-	-	-	-
nucleotide pyrophosphohydrolase	BEK99_RS12840	-	-	-	-	-	-	-	-	-
very short patch repair endonuclease	BEK99_RS12965	-	-	-	-	-	-	-	-	-
hypothetical protein	-	SCA_RS12610	-	-	-	-	-	-	-	-
membrane protein	-	SCA_RS00385	-	-	-	-	-	-	-	-
lipase (fragment 2)	-	SCA_RS12445	-	-	-	-	-	-	-	-
hypothetical protein	-	SCA_RS00755	-	-	-	-	-	-	-	-
hypothetical protein	-	SCA_RS01595	-	-	-	-	-	-	-	-
hypothetical protein	-	SCA_RS01605	-	-	-	-	-	-	-	-
hypothetical protein	-	SCA_RS02360	-	-	-	-	-	-	-	-
hypothetical protein	-	SCA_RS02385	-	-	-	-	-	-	-	-
hypothetical protein	-	SCA_RS02390	-	-	-	-	-	-	-	-
hypothetical protein	-	SCA_RS02395	-	-	-	-	-	-	-	-
single-stranded DNA-binding protein	-	SCA_RS02425	-	-	-	-	-	-	-	-
HNH endonuclease	-	SCA_RS02430	-	-	-	-	-	-	-	-
hypothetical protein	-	SCA_RS02440	-	-	-	-	-	-	-	-
hypothetical protein	-	SCA_RS02475	-	-	-	-	-	-	-	-
hypothetical protein	-	SCA_RS02480	-	-	-	-	-	-	-	-
hypothetical protein	-	SCA_RS02500	-	-	-	-	-	-	-	-

Product	*S. carnosus*		*S. equorum*		*S. succinus*			*S. xylosus*		*S. saprophyticus*
	**JCM 6069**	**TM300**	**KS1039**	**Mu2**	**14BME20**	**CSM 77**	**DSM 14617**	**C2a**	**HKUOPL8**	**ATCC 15305**
hypothetical protein	-	SCA_RS02505	-	-	-	-	-	-	-	-
hypothetical protein	-	SCA_RS02510	-	-	-	-	-	-	-	-
hypothetical protein	-	SCA_RS02515	-	-	-	-	-	-	-	-
peptidase	-	SCA_RS02545	-	-	-	-	-	-	-	-
phage tail tape measure protein	-	SCA_RS12640	-	-	-	-	-	-	-	-
hypothetical protein	-	SCA_RS02595	-	-	-	-	-	-	-	-
hypothetical protein	-	SCA_RS02610	-	-	-	-	-	-	-	-
hypothetical protein	-	SCA_RS02615	-	-	-	-	-	-	-	-
hypothetical protein	-	SCA_RS02655	-	-	-	-	-	-	-	-
hypothetical protein	-	SCA_RS02660	-	-	-	-	-	-	-	-
transcriptional regulator	-	SCA_RS03245	-	-	-	-	-	-	-	-
tagatose-bisphosphate aldolase	-	SCA_RS03415	-	-	-	-	-	-	-	-
tagatose-6-phosphate kinase	-	SCA_RS03420	-	-	-	-	-	-	-	-
ABC transporter ATP-binding protein	-	SCA_RS04720	-	-	-	-	-	-	-	-
ABC transporter permease	-	SCA_RS04725	-	-	-	-	-	-	-	-
PTS glucoside EIICBA component	-	SCA_RS05020	-	-	-	-	-	-	-	-
hypothetical protein	-	SCA_RS12675	-	-	-	-	-	-	-	-
hypothetical protein	-	SCA_RS07615	-	-	-	-	-	-	-	-
hypothetical protein	-	SCA_RS07685	-	-	-	-	-	-	-	-
hypothetical protein	-	SCA_RS07690	-	-	-	-	-	-	-	-
hypothetical protein	-	SCA_RS07695	-	-	-	-	-	-	-	-
hypothetical protein	-	SCA_RS07710	-	-	-	-	-	-	-	-
hypothetical protein	-	SCA_RS07715	-	-	-	-	-	-	-	-
phage-associated protein	-	SCA_RS07720	-	-	-	-	-	-	-	-
hypothetical protein	-	SCA_RS07745	-	-	-	-	-	-	-	-
hypothetical protein	-	SCA_RS09350	-	-	-	-	-	-	-	-
2-pyrone-4,6-dicarboxylate hydrolase	-	SCA_RS10075	-	-	-	-	-	-	-	-
hypothetical protein	-	SCA_RS10595	-	-	-	-	-	-	-	-
hypothetical protein	-	SCA_RS12705	-	-	-	-	-	-	-	-
hypothetical protein	-	SCA_RS12545	-	-	-	-	-	-	-	-
hypothetical protein	-	SCA_RS11120	-	-	-	-	-	-	-	-

Product	S. carnosus		S. equorum		S. succinus			S. xylosus		S. saprophyticus
	**JCM 6069**	**TM300**	**KS1039**	**Mu2**	**14BME20**	**CSM 77**	**DSM 14617**	**C2a**	**HKUOPL8**	**ATCC 15305**
EfeM/EfeO family lipoprotein	-	SCA_RS11175	-	-	-	-	-	-	-	-
iron ABC transporter permease	-	SCA_RS11295	-	-	-	-	-	-	-	-
ABC transporter substrate-binding protein	-	SCA_RS11300	-	-	-	-	-	-	-	-
hypothetical protein	-	SCA_RS12590	-	-	-	-	-	-	-	-
hypothetical protein	-	SCA_RS11515	-	-	-	-	-	-	-	-
hypothetical protein	-	SCA_RS11565	-	-	-	-	-	-	-	-
hypothetical protein	-	SCA_RS11710	-	-	-	-	-	-	-	-
NAD(P)-dependent oxidoreductase	-	SCA_RS11800	-	-	-	-	-	-	-	-
glycosyltransferase family 1 protein	-	SCA_RS11805	-	-	-	-	-	-	-	-
hypothetical protein	-	SCA_RS11810	-	-	-	-	-	-	-	-
hypothetical protein	-	SCA_RS11825	-	-	-	-	-	-	-	-
hypothetical protein	-	SCA_RS11830	-	-	-	-	-	-	-	-
hypothetical protein	-	SCA_RS11835	-	-	-	-	-	-	-	-
EamA family transporter	-	SCA_RS11870	-	-	-	-	-	-	-	-
gamma-glutamyl-gamma-aminobutyrate hydrolase	-	SCA_RS11995	-	-	-	-	-	-	-	-
histidine phosphatase family protein	-	SCA_RS12065	-	-	-	-	-	-	-	-
CDP-glycerol glycerophosphotransferase	-	SCA_RS12080	-	-	-	-	-	-	-	-
fructose-bisphosphatase class II	-	SCA_RS12120	-	-	-	-	-	-	-	-
hypothetical protein	-	SCA_RS12180	-	-	-	-	-	-	-	-
ABC transporter	-	SCA_RS12190	-	-	-	-	-	-	-	-
YitT family protein	-	SCA_RS12350	-	-	-	-	-	-	-	-
hypothetical protein	-	SCA_RS12380	-	-	-	-	-	-	-	-
aldo/keto reductase	-	-	SE1039_RS00170	-	-	-	-	-	-	-
MerR family transcriptional regulator	-	-	SE1039_RS00175	-	-	-	-	-	-	-
Mph(C) family macrolide 2-phosphotransferase	-	-	SE1039_RS00205	-	-	-	-	-	-	-
GntR family transcriptional regulator	-	-	SE1039_RS00220	-	-	-	-	-	-	-
tartrate dehydrogenase	-	-	SE1039_RS00225	-	-	-	-	-	-	-
cation transporter	-	-	SE1039_RS00230	-	-	-	-	-	-	-
glycerate kinase	-	-	SE1039_RS00235	-	-	-	-	-	-	-
helix-turn-helix domain-containing protein	-	-	SE1039_RS00240	-	-	-	-	-	-	-
tripartite tricarboxylate transporter TctB family protein	-	-	SE1039_RS00300	-	-	-	-	-	-	-

Product	*S. carnosus*		*S. equorum*		*S. succinus*			*S. xylosus*		*S. saprophyticus*
	**JCM 6069**	**TM300**	**KS1039**	**Mu2**	**14BME20**	**CSM 77**	**DSM 14617**	**C2a**	**HKUOPL8**	**ATCC 15305**
transporter	-	-	SE1039_RS00305	-	-	-	-	-	-	-
hypothetical protein	-	-	SE1039_RS00710	-	-	-	-	-	-	-
transcriptional antiterminator	-	-	SE1039_RS00740	-	-	-	-	-	-	-
hypothetical protein	-	-	SE1039_RS00905	-	-	-	-	-	-	-
hypothetical protein	-	-	SE1039_RS00915	-	-	-	-	-	-	-
hypothetical protein	-	-	SE1039_RS00920	-	-	-	-	-	-	-
hypothetical protein	-	-	SE1039_RS00925	-	-	-	-	-	-	-
hypothetical protein	-	-	SE1039_RS00930	-	-	-	-	-	-	-
ABC transporter	-	-	SE1039_RS00935	-	-	-	-	-	-	-
hypothetical protein	-	-	SE1039_RS13530	-	-	-	-	-	-	-
ISL3 family transposase	-	-	SE1039_RS01160	-	-	-	-	-	-	-
hypothetical protein	-	-	SE1039_RS01600	-	-	-	-	-	-	-
hypothetical protein	-	-	SE1039_RS01605	-	-	-	-	-	-	-
hypothetical protein	-	-	SE1039_RS01670	-	-	-	-	-	-	-
hypothetical protein	-	-	SE1039_RS01675	-	-	-	-	-	-	-
hypothetical protein	-	-	SE1039_RS01845	-	-	-	-	-	-	-
hypothetical protein	-	-	SE1039_RS01850	-	-	-	-	-	-	-
TetR/AcrR family transcriptional regulator	-	-	SE1039_RS01855	-	-	-	-	-	-	-
MFS transporter	-	-	SE1039_RS01860	-	-	-	-	-	-	-
hypothetical protein	-	-	SE1039_RS01905	-	-	-	-	-	-	-
hypothetical protein	-	-	SE1039_RS01950	-	-	-	-	-	-	-
hypothetical protein	-	-	SE1039_RS04530	-	-	-	-	-	-	-
hypothetical protein	-	-	SE1039_RS04540	-	-	-	-	-	-	-
DUF4352 domain-containing protein	-	-	SE1039_RS04545	-	-	-	-	-	-	-
putative holin-like toxin	-	-	SE1039_RS13565	-	-	-	-	-	-	-
hypothetical protein	-	-	SE1039_RS04550	-	-	-	-	-	-	-
hypothetical protein	-	-	SE1039_RS04780	-	-	-	-	-	-	-
hypothetical protein	-	-	SE1039_RS04785	-	-	-	-	-	-	-
hypothetical protein	-	-	SE1039_RS04805	-	-	-	-	-	-	-
hypothetical protein	-	-	SE1039_RS04810	-	-	-	-	-	-	-
hypothetical protein	-	-	SE1039_RS04835	-	-	-	-	-	-	-

Product	*S. carnosus*		*S. equorum*		*S. succinus*			*S. xylosus*		*S. saprophyticus*
	**JCM 6069**	**TM300**	**KS1039**	**Mu2**	**14BME20**	**CSM 77**	**DSM 14617**	**C2a**	**HKUOPL8**	**ATCC 15305**
XRE family transcriptional regulator	-	-	SE1039_RS04845	-	-	-	-	-	-	-
hypothetical protein	-	-	SE1039_RS04895	-	-	-	-	-	-	-
terminase small subunit	-	-	SE1039_RS04945	-	-	-	-	-	-	-
hypothetical protein	-	-	SE1039_RS05025	-	-	-	-	-	-	-
ParA family protein	-	-	SE1039_RS05070	-	-	-	-	-	-	-
hypothetical protein	-	-	SE1039_RS05075	-	-	-	-	-	-	-
hypothetical protein	-	-	SE1039_RS05080	-	-	-	-	-	-	-
hypothetical protein	-	-	SE1039_RS05095	-	-	-	-	-	-	-
hypothetical protein	-	-	SE1039_RS05140	-	-	-	-	-	-	-
hypothetical protein	-	-	SE1039_RS05565	-	-	-	-	-	-	-
hypothetical protein	-	-	SE1039_RS05805	-	-	-	-	-	-	-
hypothetical protein	-	-	SE1039_RS13575	-	-	-	-	-	-	-
hypothetical protein	-	-	SE1039_RS06005	-	-	-	-	-	-	-
hypothetical protein	-	-	SE1039_RS06275	-	-	-	-	-	-	-
hypothetical protein	-	-	SE1039_RS07235	-	-	-	-	-	-	-
3-hydroxybutyryl-CoA dehydrogenase	-	-	SE1039_RS12395	-	-	-	-	-	-	-
citrate transporter	-	-	SE1039_RS12400	-	-	-	-	-	-	-
histidinol dehydrogenase	-	-	SE1039_RS12405	-	-	-	-	-	-	-
LacI family transcriptional regulator	-	-	SE1039_RS12410	-	-	-	-	-	-	-
iron ABC transporter permease	-	-	SE1039_RS12710	-	-	-	-	-	-	-
preprotein translocase subunit YajC	-	-	SE1039_RS13630	-	-	-	-	-	-	-
hypothetical protein	-	-	SE1039_RS12755	-	-	-	-	-	-	-
hypothetical protein	-	-	SE1039_RS12765	-	-	-	-	-	-	-
hypothetical protein	-	-	SE1039_RS12800	-	-	-	-	-	-	-
hypothetical protein	-	-	SE1039_RS12805	-	-	-	-	-	-	-
hypothetical protein	-	-	SE1039_RS12970	-	-	-	-	-	-	-
hypothetical protein	-	-	SE1039_RS12975	-	-	-	-	-	-	-
DNA polymerase	-	-	SE1039_RS12985	-	-	-	-	-	-	-
hypothetical protein	-	-	SE1039_RS12990	-	-	-	-	-	-	-
hypothetical protein	-	-	SE1039_RS12995	-	-	-	-	-	-	-
hypothetical protein	-	-	SE1039_RS13080	-	-	-	-	-	-	-

Product	*S. carnosus*		*S. equorum*		*S. succinus*			*S. xylosus*		*S. saprophyticus*
	**JCM 6069**	**TM300**	**KS1039**	**Mu2**	**14BME20**	**CSM 77**	**DSM 14617**	**C2a**	**HKUOPL8**	**ATCC 15305**
5-methylcytosine-specific restriction system specificity protein McrC	-	-	SE1039_RS13085	-	-	-	-	-	-	-
GTPase	-	-	SE1039_RS13090	-	-	-	-	-	-	-
hypothetical protein	-	-	SE1039_RS13100	-	-	-	-	-	-	-
hypothetical protein	-	-	SE1039_RS13105	-	-	-	-	-	-	-
CRISPR-associated endoribonuclease Cas6	-	-	SE1039_RS13110	-	-	-	-	-	-	-
type III-A CRISPR-associated protein Csm6	-	-	SE1039_RS13115	-	-	-	-	-	-	-
type III-A CRISPR-associated RAMP protein Csm5	-	-	SE1039_RS13120	-	-	-	-	-	-	-
type III-A CRISPR-associated RAMP protein Csm4	-	-	SE1039_RS13125	-	-	-	-	-	-	-
type III-A CRISPR-associated RAMP protein Csm3	-	-	SE1039_RS13130	-	-	-	-	-	-	-
type III-A CRISPR-associated protein Csm2	-	-	SE1039_RS13135	-	-	-	-	-	-	-
CRISPR-associated endonuclease Cas2	-	-	SE1039_RS13145	-	-	-	-	-	-	-
type II CRISPR-associated endonuclease Cas1	-	-	SE1039_RS13150	-	-	-	-	-	-	-
hypothetical protein	-	-	SE1039_RS13160	-	-	-	-	-	-	-
type I restriction endonuclease subunit S	-	-	SE1039_RS13180	-	-	-	-	-	-	-
hypothetical protein	-	-	SE1039_RS13225	-	-	-	-	-	-	-
hypothetical protein	-	-	SE1039_RS13230	-	-	-	-	-	-	-
hypothetical protein	-	-	SE1039_RS13235	-	-	-	-	-	-	-
phosphoglucomutase	-	-	SE1039_RS13255	-	-	-	-	-	-	-
CDP-glycerol glycerophosphotransferase	-	-	SE1039_RS13265	-	-	-	-	-	-	-
hypothetical protein	-	-	SE1039_RS13300	-	-	-	-	-	-	-
hypothetical protein	-	-	SE1039_RS13305	-	-	-	-	-	-	-
hypothetical protein	-	-	SE1039_RS13645	-	-	-	-	-	-	-
hypothetical protein	-	-	SE1039_RS13365	-	-	-	-	-	-	-
hypothetical protein	-	-	-	SEQMU2_RS14270	-	-	-	-	-	-
hypothetical protein	-	-	-	SEQMU2_RS14275	-	-	-	-	-	-
hypothetical protein	-	-	-	SEQMU2_RS14280	-	-	-	-	-	-
MerR family transcriptional regulator	-	-	-	SEQMU2_RS14285	-	-	-	-	-	-
integrase	-	-	-	SEQMU2_RS14290	-	-	-	-	-	-
hypothetical protein	-	-	-	SEQMU2_RS14295	-	-	-	-	-	-
hypothetical protein	-	-	-	SEQMU2_RS14300	-	-	-	-	-	-
hypothetical protein	-	-	-	SEQMU2_RS14305	-	-	-	-	-	-

Product	*S. carnosus*		*S. equorum*		*S. succinus*			*S. xylosus*		*S. saprophyticus*
	**JCM 6069**	**TM300**	**KS1039**	**Mu2**	**14BME20**	**CSM 77**	**DSM 14617**	**C2a**	**HKUOPL8**	**ATCC 15305**
hypothetical protein|Uncharacterized protein C	-	-	-	SEQMU2_RS14240	-	-	-	-	-	-
hypothetical protein	-	-	-	SEQMU2_RS14260	-	-	-	-	-	-
hypothetical protein	-	-	-	SEQMU2_RS14265	-	-	-	-	-	-
hypothetical protein	-	-	-	SEQMU2_RS14095	-	-	-	-	-	-
hypothetical protein	-	-	-	SEQMU2_RS14100	-	-	-	-	-	-
hypothetical protein	-	-	-	SEQMU2_RS14105	-	-	-	-	-	-
transposase|Transposon Tn917 resolvase	-	-	-	SEQMU2_RS14110	-	-	-	-	-	-
hypothetical protein	-	-	-	SEQMU2_RS14120	-	-	-	-	-	-
membrane associated protein	-	-	-	SEQMU2_RS14155	-	-	-	-	-	-
cation-transporting ATPase|Zinc-transporting ATP ase	-	-	-	SEQMU2_RS14160	-	-	-	-	-	-
transposase|Transposase for insertion sequence e lement IS257 in transposon Tn4003	-	-	-	SEQMU2_RS14165	-	-	-	-	-	-
transposase|Transposase for insertion sequence e lement IS257 in transposon Tn4003	-	-	-	SEQMU2_RS14170	-	-	-	-	-	-
hypothetical protein	-	-	-	SEQMU2_RS14180	-	-	-	-	-	-
hypothetical protein	-	-	-	SEQMU2_RS14195	-	-	-	-	-	-
GntR family transcriptional regulator|Uncharacte rized HTH-type transcriptional regulator YhdI	-	-	-	SEQMU2_RS14215	-	-	-	-	-	-
hypothetical protein	-	-	-	SEQMU2_RS13795	-	-	-	-	-	-
hypothetical protein	-	-	-	SEQMU2_RS13820	-	-	-	-	-	-
hypothetical protein	-	-	-	SEQMU2_RS13835	-	-	-	-	-	-
addiction module protein	-	-	-	SEQMU2_RS13840	-	-	-	-	-	-
prevent-host-death protein	-	-	-	SEQMU2_RS13845	-	-	-	-	-	-
hypothetical protein	-	-	-	SEQMU2_RS13850	-	-	-	-	-	-
hypothetical protein	-	-	-	SEQMU2_RS13855	-	-	-	-	-	-
hypothetical protein	-	-	-	SEQMU2_RS13860	-	-	-	-	-	-
membrane protein|Uncharacterized protein YhcI	-	-	-	SEQMU2_RS13870	-	-	-	-	-	-
lantibiotic ABC transporter ATP-binding protein| Phosphonates import ATP-binding protein PhnC {E	-	-	-	SEQMU2_RS13875	-	-	-	-	-	-
hypothetical protein|Oleate hydratase	-	-	-	SEQMU2_RS13885	-	-	-	-	-	-
glycerophosphoryl diester phosphodiesterase|Puta tive glycerophosphoryl diester phosphodiesteras	-	-	-	SEQMU2_RS13920	-	-	-	-	-	-
hypothetical protein	-	-	-	SEQMU2_RS13935	-	-	-	-	-	-
restriction endonuclease S subunit	-	-	-	SEQMU2_RS13940	-	-	-	-	-	-
hypothetical protein|Pyruvate decarboxylase	-	-	-	SEQMU2_RS13965	-	-	-	-	-	-

Product	S. carnosus		S. equorum		S. succinus			S. xylosus		S. saprophyticus
	JCM 6069	TM300	KS1039	Mu2	14BME20	CSM 77	DSM 14617	C2a	HKUOPL8	ATCC 15305
hypothetical protein	-	-	-	SEQMU2_RS13970	-	-	-	-	-	-
hypothetical protein|Probable serine/threonine-p rotein kinase irlF	-	-	-	SEQMU2_RS13980	-	-	-	-	-	-
hypothetical protein	-	-	-	SEQMU2_RS14005	-	-	-	-	-	-
universal stress protein	-	-	-	SEQMU2_RS14020	-	-	-	-	-	-
hypothetical protein|Transcriptional activator R amA	-	-	-	SEQMU2_RS10410	-	-	-	-	-	-
integrase|ICEBs1 integrase	-	-	-	SEQMU2_RS10650	-	-	-	-	-	-
phage protein	-	-	-	SEQMU2_RS10655	-	-	-	-	-	-
transcriptional regulator	-	-	-	SEQMU2_RS10660	-	-	-	-	-	-
CRISPR-associated protein Cas2	-	-	-	SEQMU2_RS10685	-	-	-	-	-	-
hypothetical protein	-	-	-	SEQMU2_RS10690	-	-	-	-	-	-
hypothetical protein	-	-	-	SEQMU2_RS10695	-	-	-	-	-	-
hypothetical protein	-	-	-	SEQMU2_RS10700	-	-	-	-	-	-
hypothetical protein	-	-	-	SEQMU2_RS10705	-	-	-	-	-	-
hypothetical protein	-	-	-	SEQMU2_RS10710	-	-	-	-	-	-
hypothetical protein	-	-	-	SEQMU2_RS10715	-	-	-	-	-	-
hypothetical protein|Uncharacterized protein Yqa J	-	-	-	SEQMU2_RS10720	-	-	-	-	-	-
DNA recombination protein RecT|Protein RecT	-	-	-	SEQMU2_RS10725	-	-	-	-	-	-
hypothetical protein	-	-	-	SEQMU2_RS10730	-	-	-	-	-	-
hypothetical protein	-	-	-	SEQMU2_RS10745	-	-	-	-	-	-
hypothetical protein	-	-	-	SEQMU2_RS10750	-	-	-	-	-	-
single-stranded DNA-binding protein|Single-stran ded DNA-binding protein 1 {ECO:0000255|HAMAP-Rule:MF_00984 }	-	-	-	SEQMU2_RS10755	-	-	-	-	-	-
hypothetical protein	-	-	-	SEQMU2_RS10760	-	-	-	-	-	-
hypothetical protein	-	-	-	SEQMU2_RS10765	-	-	-	-	-	-
hypothetical protein	-	-	-	SEQMU2_RS10770	-	-	-	-	-	-
hypothetical protein	-	-	-	SEQMU2_RS10775	-	-	-	-	-	-
hypothetical protein	-	-	-	SEQMU2_RS10780	-	-	-	-	-	-
Holliday junction DNA helicase|Holliday junction resolvase RecU	-	-	-	SEQMU2_RS10785	-	-	-	-	-	-
hypothetical protein	-	-	-	SEQMU2_RS10790	-	-	-	-	-	-
phage protein	-	-	-	SEQMU2_RS10800	-	-	-	-	-	-
hypothetical protein	-	-	-	SEQMU2_RS10805	-	-	-	-	-	-

Product	*S. carnosus*		*S. equorum*		*S. succinus*			*S. xylosus*		*S. saprophyticus*
	**JCM 6069**	**TM300**	**KS1039**	**Mu2**	**14BME20**	**CSM 77**	**DSM 14617**	**C2a**	**HKUOPL8**	**ATCC 15305**
phage capsid protein	-	-	-	SEQMU2_RS10810	-	-	-	-	-	-
hypothetical protein	-	-	-	SEQMU2_RS10815	-	-	-	-	-	-
hypothetical protein	-	-	-	SEQMU2_RS10820	-	-	-	-	-	-
hypothetical protein	-	-	-	SEQMU2_RS10825	-	-	-	-	-	-
hypothetical protein	-	-	-	SEQMU2_RS10830	-	-	-	-	-	-
hypothetical protein	-	-	-	SEQMU2_RS10835	-	-	-	-	-	-
hypothetical protein	-	-	-	SEQMU2_RS10840	-	-	-	-	-	-
hypothetical protein	-	-	-	SEQMU2_RS10845	-	-	-	-	-	-
hypothetical protein	-	-	-	SEQMU2_RS10850	-	-	-	-	-	-
hypothetical protein	-	-	-	SEQMU2_RS10855	-	-	-	-	-	-
hypothetical protein|Uncharacterized protein CA_ P0160	-	-	-	SEQMU2_RS10860	-	-	-	-	-	-
hypothetical protein	-	-	-	SEQMU2_RS10865	-	-	-	-	-	-
hypothetical protein	-	-	-	SEQMU2_RS10870	-	-	-	-	-	-
hypothetical protein|Murein DD-endopeptidase Mep M	-	-	-	SEQMU2_RS10875	-	-	-	-	-	-
phage tail protein	-	-	-	SEQMU2_RS10880	-	-	-	-	-	-
hypothetical protein|Papilin	-	-	-	SEQMU2_RS10885	-	-	-	-	-	-
hypothetical protein	-	-	-	SEQMU2_RS10890	-	-	-	-	-	-
hypothetical protein	-	-	-	SEQMU2_RS10895	-	-	-	-	-	-
hypothetical protein	-	-	-	SEQMU2_RS10900	-	-	-	-	-	-
DNA-binding protein	-	-	-	SEQMU2_RS10910	-	-	-	-	-	-
hypothetical protein	-	-	-	SEQMU2_RS11280	-	-	-	-	-	-
hypothetical protein	-	-	-	SEQMU2_RS11325	-	-	-	-	-	-
hypothetical protein	-	-	-	SEQMU2_RS11330	-	-	-	-	-	-
hypothetical protein	-	-	-	SEQMU2_RS11335	-	-	-	-	-	-
hypothetical protein	-	-	-	SEQMU2_RS11340	-	-	-	-	-	-
hypothetical protein	-	-	-	SEQMU2_RS11345	-	-	-	-	-	-
hypothetical protein	-	-	-	SEQMU2_RS11350	-	-	-	-	-	-
phage protein	-	-	-	SEQMU2_RS11355	-	-	-	-	-	-
hypothetical protein	-	-	-	SEQMU2_RS11365	-	-	-	-	-	-
hypothetical protein	-	-	-	SEQMU2_RS11370	-	-	-	-	-	-
hypothetical protein	-	-	-	SEQMU2_RS11375	-	-	-	-	-	-

Product	S. carnosus		S. equorum		S. succinus			S. xylosus		S. saprophyticus
	JCM 6069	TM300	KS1039	Mu2	14BME20	CSM 77	DSM 14617	C2a	HKUOPL8	ATCC 15305
hypothetical protein	-	-	-	SEQMU2_RS11380	-	-	-	-	-	-
hypothetical protein	-	-	-	SEQMU2_RS12480	-	-	-	-	-	-
hypothetical protein	-	-	-	SEQMU2_RS12485	-	-	-	-	-	-
integrase|Transposase InsI for insertion sequenc e element IS30A	-	-	-	SEQMU2_RS12835	-	-	-	-	-	-
hypothetical protein|PTS system N-acetylglucosam ine-specific EIICBA component	-	-	-	SEQMU2_RS12840	-	-	-	-	-	-
ArsR family transcriptional regulator|Arsenical resistance operon repressor	-	-	-	SEQMU2_RS09195	-	-	-	-	-	-
pathogenicity island protein	-	-	-	SEQMU2_RS08255	-	-	-	-	-	-
hypothetical protein	-	-	-	SEQMU2_RS08260	-	-	-	-	-	-
hypothetical protein	-	-	-	SEQMU2_RS08270	-	-	-	-	-	-
hypothetical protein	-	-	-	SEQMU2_RS08275	-	-	-	-	-	-
hypothetical protein	-	-	-	SEQMU2_RS08280	-	-	-	-	-	-
hypothetical protein	-	-	-	SEQMU2_RS08285	-	-	-	-	-	-
hypothetical protein	-	-	-	SEQMU2_RS08295	-	-	-	-	-	-
hypothetical protein	-	-	-	SEQMU2_RS08300	-	-	-	-	-	-
fosmidomycin resistance protein|Metallothiol tra nsferase FosB {ECO:0000255|HAMAP-Rule:MF_01512}	-	-	-	SEQMU2_RS08320	-	-	-	-	-	-
glutathione transferase|Metallothiol transferase FosB 2 {ECO:0000255|HAMAP-Rule:MF_01512}	-	-	-	SEQMU2_RS08325	-	-	-	-	-	-
short-chain dehydrogenase|Tropinone reductase ho molog At1g07440 {ECO:0000305}	-	-	-	SEQMU2_RS08415	-	-	-	-	-	-
transposase	-	-	-	SEQMU2_RS08580	-	-	-	-	-	-
5-aminolevulinate synthase|5-aminolevulinate syn thase, mitochondrial	-	-	-	SEQMU2_RS08585	-	-	-	-	-	-
membrane protein	-	-	-	SEQMU2_RS08590	-	-	-	-	-	-
hypothetical protein	-	-	-	SEQMU2_RS08215	-	-	-	-	-	-
DNA-binding protein	-	-	-	SEQMU2_RS08225	-	-	-	-	-	-
hypothetical protein	-	-	-	SEQMU2_RS08235	-	-	-	-	-	-
hypothetical protein	-	-	-	SEQMU2_RS08250	-	-	-	-	-	-
lysophospholipase	-	-	-	SEQMU2_RS06735	-	-	-	-	-	-
hydrolase|Uncharacterized hydrolase YraK	-	-	-	SEQMU2_RS06750	-	-	-	-	-	-
hypothetical protein|Protein VraX	-	-	-	SEQMU2_RS06860	-	-	-	-	-	-
repressor	-	-	-	SEQMU2_RS07830	-	-	-	-	-	-
hypothetical protein	-	-	-	SEQMU2_RS07835	-	-	-	-	-	-
phage protein	-	-	-	SEQMU2_RS07845	-	-	-	-	-	-

Product	*S. carnosus*		*S. equorum*		*S. succinus*			*S. xylosus*		*S. saprophyticus*
	**JCM 6069**	**TM300**	**KS1039**	**Mu2**	**14BME20**	**CSM 77**	**DSM 14617**	**C2a**	**HKUOPL8**	**ATCC 15305**
hypothetical protein	-	-	-	SEQMU2_RS07850	-	-	-	-	-	-
hypothetical protein	-	-	-	SEQMU2_RS07855	-	-	-	-	-	-
hypothetical protein	-	-	-	SEQMU2_RS07860	-	-	-	-	-	-
hypothetical protein	-	-	-	SEQMU2_RS07865	-	-	-	-	-	-
hypothetical protein	-	-	-	SEQMU2_RS07870	-	-	-	-	-	-
hypothetical protein	-	-	-	SEQMU2_RS07900	-	-	-	-	-	-
hypothetical protein	-	-	-	SEQMU2_RS07910	-	-	-	-	-	-
hypothetical protein	-	-	-	SEQMU2_RS07915	-	-	-	-	-	-
hypothetical protein	-	-	-	SEQMU2_RS07930	-	-	-	-	-	-
hypothetical protein	-	-	-	SEQMU2_RS07955	-	-	-	-	-	-
terminase|PBSX phage terminase small subunit	-	-	-	SEQMU2_RS07995	-	-	-	-	-	-
phage protein	-	-	-	SEQMU2_RS08025	-	-	-	-	-	-
hypothetical protein	-	-	-	SEQMU2_RS08050	-	-	-	-	-	-
pathogenicity island protein	-	-	-	SEQMU2_RS06470	-	-	-	-	-	-
hypothetical protein	-	-	-	SEQMU2_RS06485	-	-	-	-	-	-
mRNA interferase PemK	-	-	-	SEQMU2_RS06495	-	-	-	-	-	-
GNAT family acetyltransferase	-	-	-	SEQMU2_RS06510	-	-	-	-	-	-
hypothetical protein	-	-	-	SEQMU2_RS06515	-	-	-	-	-	-
hypothetical protein	-	-	-	SEQMU2_RS05430	-	-	-	-	-	-
hypothetical protein	-	-	-	SEQMU2_RS05435	-	-	-	-	-	-
hypothetical protein	-	-	-	SEQMU2_RS05440	-	-	-	-	-	-
hypothetical protein	-	-	-	SEQMU2_RS05445	-	-	-	-	-	-
hypothetical protein	-	-	-	SEQMU2_RS05450	-	-	-	-	-	-
hypothetical protein|RecBCD enzyme subunit RecB {ECO:0000255|HAMAP-Rule:MF_01485}	-	-	-	SEQMU2_RS05455	-	-	-	-	-	-
membrane protein|Uncharacterized protein PA2218	-	-	-	SEQMU2_RS05770	-	-	-	-	-	-
hypothetical protein	-	-	-	SEQMU2_RS05805	-	-	-	-	-	-
hypothetical protein	-	-	-	SEQMU2_RS06135	-	-	-	-	-	-
hypothetical protein	-	-	-	SEQMU2_RS06210	-	-	-	-	-	-
hypothetical protein	-	-	-	SEQMU2_RS06215	-	-	-	-	-	-
hypothetical protein	-	-	-	SEQMU2_RS06220	-	-	-	-	-	-
DNA-binding protein	-	-	-	SEQMU2_RS06430	-	-	-	-	-	-

Product	*S. carnosus*		*S. equorum*		*S. succinus*			*S. xylosus*		*S. saprophyticus*
	**JCM 6069**	**TM300**	**KS1039**	**Mu2**	**14BME20**	**CSM 77**	**DSM 14617**	**C2a**	**HKUOPL8**	**ATCC 15305**
hypothetical protein	-	-	-	SEQMU2_RS06465	-	-	-	-	-	-
arsenical pump membrane protein|Arsenical pump m embrane protein	-	-	-	SEQMU2_RS05180	-	-	-	-	-	-
dihydrolipoamide dehydrogenase|Probable pyridine nucleotide-disulfide oxidoreductase RclA	-	-	-	SEQMU2_RS05190	-	-	-	-	-	-
transcriptional regulator	-	-	-	SEQMU2_RS05195	-	-	-	-	-	-
glycyl-glycine endopeptidase|Glycyl-glycine endo peptidase LytM	-	-	-	SEQMU2_RS05200	-	-	-	-	-	-
activator of HSP90 ATPase|Uncharacterized protei n YndB	-	-	-	SEQMU2_RS05205	-	-	-	-	-	-
glutamine amidotransferase|Uncharacterized prote ase YoaZ	-	-	-	SEQMU2_RS05215	-	-	-	-	-	-
DeoR family transcriptional regulator|Uncharacte rized HTH-type transcriptional regulator YobV	-	-	-	SEQMU2_RS05220	-	-	-	-	-	-
hypothetical protein	-	-	-	SEQMU2_RS05225	-	-	-	-	-	-
hypothetical protein	-	-	-	SEQMU2_RS05240	-	-	-	-	-	-
hypothetical protein	-	-	-	SEQMU2_RS05265	-	-	-	-	-	-
hypothetical protein	-	-	-	SEQMU2_RS05275	-	-	-	-	-	-
hypothetical protein	-	-	-	SEQMU2_RS05305	-	-	-	-	-	-
hypothetical protein	-	-	-	SEQMU2_RS05310	-	-	-	-	-	-
hypothetical protein	-	-	-	SEQMU2_RS05315	-	-	-	-	-	-
hypothetical protein	-	-	-	SEQMU2_RS05320	-	-	-	-	-	-
hypothetical protein	-	-	-	SEQMU2_RS05335	-	-	-	-	-	-
capsule biosynthesis protein CapK|Protein CapK	-	-	-	SEQMU2_RS05365	-	-	-	-	-	-
capsule biosynthesis protein CapJ|Protein CapJ	-	-	-	SEQMU2_RS05370	-	-	-	-	-	-
hypothetical protein	-	-	-	SEQMU2_RS05380	-	-	-	-	-	-
capsular polysaccharide biosynthesis protein|Cap sular polysaccharide biosynthesis protein CapF	-	-	-	SEQMU2_RS05385	-	-	-	-	-	-
abortive infection protein	-	-	-	SEQMU2_RS05035	-	-	-	-	-	-
hypothetical protein	-	-	-	SEQMU2_RS05040	-	-	-	-	-	-
hypothetical protein	-	-	-	SEQMU2_RS05045	-	-	-	-	-	-
hypothetical protein	-	-	-	SEQMU2_RS05050	-	-	-	-	-	-
hypothetical protein	-	-	-	SEQMU2_RS05060	-	-	-	-	-	-
hypothetical protein	-	-	-	SEQMU2_RS05065	-	-	-	-	-	-
hypothetical protein|General transcriptional cor epressor trfA	-	-	-	SEQMU2_RS05070	-	-	-	-	-	-
pathogenicity island protein	-	-	-	SEQMU2_RS05080	-	-	-	-	-	-
2-C-methyl-D-erythritol 4-phosphate cytidylyltra nsferase|Putative 2-C-methyl-D-erythritol 4-phosphate cyti	-	-	-	SEQMU2_RS04890	-	-	-	-	-	-

Product	*S. carnosus*		*S. equorum*		*S. succinus*			*S. xylosus*		*S. saprophyticus*
	**JCM 6069**	**TM300**	**KS1039**	**Mu2**	**14BME20**	**CSM 77**	**DSM 14617**	**C2a**	**HKUOPL8**	**ATCC 15305**
dylyltransferase 2										
hypothetical protein	-	-	-	SEQMU2_RS04905	-	-	-	-	-	-
hypothetical protein	-	-	-	SEQMU2_RS04955	-	-	-	-	-	-
hypothetical protein	-	-	-	SEQMU2_RS04035	-	-	-	-	-	-
hypothetical protein	-	-	-	SEQMU2_RS04345	-	-	-	-	-	-
hypothetical protein|Uncharacterized ATP-depende nt helicase MG140 homolog	-	-	-	SEQMU2_RS04350	-	-	-	-	-	-
hypothetical protein|Uncharacterized ATP-depende nt helicase MG140	-	-	-	SEQMU2_RS04355	-	-	-	-	-	-
hypothetical protein	-	-	-	SEQMU2_RS04360	-	-	-	-	-	-
GntR family transcriptional regulator|Uncharacte rized HTH-type transcriptional regulator YdhC	-	-	-	SEQMU2_RS04420	-	-	-	-	-	-
C4-dicarboxylate ABC transporter permease|Unchar acterized protein YgiK	-	-	-	SEQMU2_RS04425	-	-	-	-	-	-
C4-dicarboxylate ABC transporter permease|Sialic acid TRAP transporter permease protein SiaT	-	-	-	SEQMU2_RS04430	-	-	-	-	-	-
C4-dicarboxylate ABC transporter substrate-bindi ng protein|Uncharacterized protein HI_0052	-	-	-	SEQMU2_RS04435	-	-	-	-	-	-
hydrolase|Hydrolase MtnU	-	-	-	SEQMU2_RS04440	-	-	-	-	-	-
hypothetical protein	-	-	-	SEQMU2_RS04445	-	-	-	-	-	-
glycerate kinase|Glycerate 2-kinase	-	-	-	SEQMU2_RS04460	-	-	-	-	-	-
anion:sodium symporter	-	-	-	SEQMU2_RS04465	-	-	-	-	-	-
PhoB family transcriptional regulator|Transcript ional regulatory protein ResD	-	-	-	SEQMU2_RS04470	-	-	-	-	-	-
histidine kinase|Signal transduction histidine-p rotein kinase ArlS	-	-	-	SEQMU2_RS04475	-	-	-	-	-	-
hypothetical protein	-	-	-	SEQMU2_RS04480	-	-	-	-	-	-
sugar tyrosine-protein kinase|Putative exported peptide YydF	-	-	-	SEQMU2_RS04635	-	-	-	-	-	-
radical SAM peptide maturase|Putative peptide bi osynthesis protein YydG	-	-	-	SEQMU2_RS04640	-	-	-	-	-	-
hypothetical protein	-	-	-	SEQMU2_RS04720	-	-	-	-	-	-
glycosyl transferase|Uncharacterized glycosyltra nsferase MG025 homolog	-	-	-	SEQMU2_RS04725	-	-	-	-	-	-
UTP--glucose-1-phosphate uridylyltransferase|UTP --glucose-1-phosphate uridylyltransferase 1	-	-	-	SEQMU2_RS04730	-	-	-	-	-	-
esterase|Putative acetyl-hydrolase LipR	-	-	-	SEQMU2_RS04775	-	-	-	-	-	-
hypothetical protein	-	-	-	SEQMU2_RS04870	-	-	-	-	-	-
hypothetical protein|Uncharacterized glycosyltra nsferase MJ1059	-	-	-	SEQMU2_RS04875	-	-	-	-	-	-
teichoic acid biosynthesis protein|Putative ribi tolphosphotransferase	-	-	-	SEQMU2_RS04885	-	-	-	-	-	-

Product	*S. carnosus*		*S. equorum*		*S. succinus*			*S. xylosus*		*S. saprophyticus*
	**JCM 6069**	**TM300**	**KS1039**	**Mu2**	**14BME20**	**CSM 77**	**DSM 14617**	**C2a**	**HKUOPL8**	**ATCC 15305**
2-C-methyl-D-erythritol 4-phosphate cytidylyltra nsferase|Putative 2-C-methyl-D-erythritol 4-phosphate cyti dylyltransferase 2	-	-	-	SEQMU2_RS03025	-	-	-	-	-	-
Linalool 8-monooxygenase|Cytochrome P450 119	-	-	-	SEQMU2_RS00570	-	-	-	-	-	-
hypothetical protein	-	-	-	SEQMU2_RS00575	-	-	-	-	-	-
hypothetical protein|Uncharacterized ABC transpo rter ATP-binding protein YwjA	-	-	-	SEQMU2_RS00275	-	-	-	-	-	-
phage protein	-	-	-	SEQMU2_RS00285	-	-	-	-	-	-
DNA-binding protein	-	-	-	SEQMU2_RS00290	-	-	-	-	-	-
hypothetical protein	-	-	-	SEQMU2_RS00300	-	-	-	-	-	-
DNA primase	-	-	-	SEQMU2_RS00310	-	-	-	-	-	-
hypothetical protein	-	-	-	SEQMU2_RS00315	-	-	-	-	-	-
hypothetical protein	-	-	-	-	-	-	-	-	-	SSP_RS00145
site-specific DNA-methyltransferase	-	-	-	-	-	-	-	-	-	SSP_RS00150
modification methylase	-	-	-	-	-	-	-	-	-	SSP_RS00155
hypothetical protein	-	-	-	-	-	-	-	-	-	SSP_RS00165
hypothetical protein	-	-	-	-	-	-	-	-	-	SSP_RS00210
glycosyl transferase	-	-	-	-	-	-	-	-	-	SSP_RS00325
glycosyl transferase	-	-	-	-	-	-	-	-	-	SSP_RS00330
capsular polysaccharide synthesis protein	-	-	-	-	-	-	-	-	-	SSP_RS00335
glycosyl transferase	-	-	-	-	-	-	-	-	-	SSP_RS00340
polysaccharide polymerase	-	-	-	-	-	-	-	-	-	SSP_RS00350
hypothetical protein	-	-	-	-	-	-	-	-	-	SSP_RS12690
hypothetical protein	-	-	-	-	-	-	-	-	-	SSP_RS12695
HNH endonuclease	-	-	-	-	-	-	-	-	-	SSP_RS00370
ATPase	-	-	-	-	-	-	-	-	-	SSP_RS00375
hypothetical protein	-	-	-	-	-	-	-	-	-	SSP_RS12700
glycosyl transferase	-	-	-	-	-	-	-	-	-	SSP_RS00390
DUF2648 domain-containing protein	-	-	-	-	-	-	-	-	-	SSP_RS12705
L-lactate permease	-	-	-	-	-	-	-	-	-	SSP_RS00430
hypothetical protein	-	-	-	-	-	-	-	-	-	SSP_RS00435
acetyltransferase	-	-	-	-	-	-	-	-	-	SSP_RS00480
hypothetical protein	-	-	-	-	-	-	-	-	-	SSP_RS00490

Product	*S. carnosus*		*S. equorum*		*S. succinus*			*S. xylosus*		*S. saprophyticus*
	**JCM 6069**	**TM300**	**KS1039**	**Mu2**	**14BME20**	**CSM 77**	**DSM 14617**	**C2a**	**HKUOPL8**	**ATCC 15305**
hypothetical protein	-	-	-	-	-	-	-	-	-	SSP_RS00495
MBL fold metallo-hydrolase	-	-	-	-	-	-	-	-	-	SSP_RS00505
transcriptional regulator	-	-	-	-	-	-	-	-	-	SSP_RS00510
hypothetical protein	-	-	-	-	-	-	-	-	-	SSP_RS12720
SIS domain-containing protein	-	-	-	-	-	-	-	-	-	SSP_RS12725
arginase	-	-	-	-	-	-	-	-	-	SSP_RS00620
TetR family transcriptional regulator	-	-	-	-	-	-	-	-	-	SSP_RS00650
cell wall-anchored protein	-	-	-	-	-	-	-	-	-	SSP_RS12730
hypothetical protein	-	-	-	-	-	-	-	-	-	SSP_RS00730
hypothetical protein	-	-	-	-	-	-	-	-	-	SSP_RS00770
hypothetical protein	-	-	-	-	-	-	-	-	-	SSP_RS00780
hypothetical protein	-	-	-	-	-	-	-	-	-	SSP_RS00850
hypothetical protein	-	-	-	-	-	-	-	-	-	SSP_RS00885
4-carboxymuconolactone decarboxylase	-	-	-	-	-	-	-	-	-	SSP_RS00980
LytTR family transcriptional regulator	-	-	-	-	-	-	-	-	-	SSP_RS00985
NAD(P)-dependent oxidoreductase	-	-	-	-	-	-	-	-	-	SSP_RS00990
hypothetical protein	-	-	-	-	-	-	-	-	-	SSP_RS00995
MFS transporter	-	-	-	-	-	-	-	-	-	SSP_RS01000
hypothetical protein	-	-	-	-	-	-	-	-	-	SSP_RS01095
transcriptional antiterminator	-	-	-	-	-	-	-	-	-	SSP_RS01195
MFS transporter	-	-	-	-	-	-	-	-	-	SSP_RS12740
MFS transporter	-	-	-	-	-	-	-	-	-	SSP_RS12745
IS30 family transposaseIDENTICAL PARALOGS:	-	-	-	-	-	-	-	-	-	SSP_RS01630
IS30 family transposase	-	-	-	-	-	-	-	-	-	SSP_RS07225
AraC family transcriptional regulator	-	-	-	-	-	-	-	-	-	SSP_RS01675
6-phospho-beta-glucosidase	-	-	-	-	-	-	-	-	-	SSP_RS01855
transcription antiterminator	-	-	-	-	-	-	-	-	-	SSP_RS01860
beta-1,4-N-acetylgalactosaminyltransferase	-	-	-	-	-	-	-	-	-	SSP_RS01865
hypothetical protein	-	-	-	-	-	-	-	-	-	SSP_RS02425
lipase precursor	-	-	-	-	-	-	-	-	-	SSP_RS12755
ferrichrome ABC transporter substrate-binding protein	-	-	-	-	-	-	-	-	-	SSP_RS02565

Product	*S. carnosus*		*S. equorum*		*S. succinus*			*S. xylosus*		*S. saprophyticus*
	**JCM 6069**	**TM300**	**KS1039**	**Mu2**	**14BME20**	**CSM 77**	**DSM 14617**	**C2a**	**HKUOPL8**	**ATCC 15305**
transposase	-	-	-	-	-	-	-	-	-	SSP_RS02825
metal-dependent hydrolase	-	-	-	-	-	-	-	-	-	SSP_RS03090
hypothetical protein	-	-	-	-	-	-	-	-	-	SSP_RS04060
hypothetical protein	-	-	-	-	-	-	-	-	-	SSP_RS04205
MarR family transcriptional regulator	-	-	-	-	-	-	-	-	-	SSP_RS04230
type I addiction module toxin Fst family	-	-	-	-	-	-	-	-	-	SSP_RS12765
integrase	-	-	-	-	-	-	-	-	-	SSP_RS05625
autolysin	-	-	-	-	-	-	-	-	-	SSP_RS06625
hypothetical protein	-	-	-	-	-	-	-	-	-	SSP_RS06750
hypothetical protein	-	-	-	-	-	-	-	-	-	SSP_RS07030
hypothetical protein	-	-	-	-	-	-	-	-	-	SSP_RS07105
phosphonate ABC transporter permease protein PhnE	-	-	-	-	-	-	-	-	-	SSP_RS07230
phosphonate ABC transporter permease protein PhnE	-	-	-	-	-	-	-	-	-	SSP_RS07235
phosphonates import ATP-binding protein PhnC	-	-	-	-	-	-	-	-	-	SSP_RS07240
phosphate/phosphite/phosphonate ABC transporter substrate-binding protein	-	-	-	-	-	-	-	-	-	SSP_RS07245
hypothetical protein	-	-	-	-	-	-	-	-	-	SSP_RS07250
bifunctional metallophosphatase/5-nucleotidase	-	-	-	-	-	-	-	-	-	SSP_RS07255
hypothetical protein	-	-	-	-	-	-	-	-	-	SSP_RS07260
hypothetical protein	-	-	-	-	-	-	-	-	-	SSP_RS07265
LysE family translocator	-	-	-	-	-	-	-	-	-	SSP_RS08105
transposase	-	-	-	-	-	-	-	-	-	SSP_RS08120
transposase	-	-	-	-	-	-	-	-	-	SSP_RS08125
hypothetical protein	-	-	-	-	-	-	-	-	-	SSP_RS08130
IS6 family transposase	-	-	-	-	-	-	-	-	-	SSP_RS08145
PLP-dependent aminotransferase family protein	-	-	-	-	-	-	-	-	-	SSP_RS08175
transcriptional regulator	-	-	-	-	-	-	-	-	-	SSP_RS08215
aldehyde dehydrogenase	-	-	-	-	-	-	-	-	-	SSP_RS08220
sodium:proton antiporter	-	-	-	-	-	-	-	-	-	SSP_RS12810
alpha/beta hydrolase	-	-	-	-	-	-	-	-	-	SSP_RS09515
hypothetical protein	-	-	-	-	-	-	-	-	-	SSP_RS09520
hypothetical protein	-	-	-	-	-	-	-	-	-	SSP_RS09570

Product	*S. carnosus*		*S. equorum*		*S. succinus*			*S. xylosus*		*S. saprophyticus*
	**JCM 6069**	**TM300**	**KS1039**	**Mu2**	**14BME20**	**CSM 77**	**DSM 14617**	**C2a**	**HKUOPL8**	**ATCC 15305**
aminoglycoside 6-adenylyltransferase	-	-	-	-	-	-	-	-	-	SSP_RS09640
hypothetical protein	-	-	-	-	-	-	-	-	-	SSP_RS09685
hypothetical protein	-	-	-	-	-	-	-	-	-	SSP_RS09700
hypothetical protein	-	-	-	-	-	-	-	-	-	SSP_RS09735
transcriptional regulator	-	-	-	-	-	-	-	-	-	SSP_RS09745
transcriptional regulator	-	-	-	-	-	-	-	-	-	SSP_RS09750
hypothetical protein	-	-	-	-	-	-	-	-	-	SSP_RS10810
hypothetical protein	-	-	-	-	-	-	-	-	-	SSP_RS10905
hypothetical protein	-	-	-	-	-	-	-	-	-	SSP_RS10930
N-acetyltransferase	-	-	-	-	-	-	-	-	-	SSP_RS11565
hypothetical protein	-	-	-	-	-	-	-	-	-	SSP_RS11595
hypothetical protein	-	-	-	-	-	-	-	-	-	SSP_RS11665
hypothetical protein	-	-	-	-	-	-	-	-	-	SSP_RS11675
hypothetical protein	-	-	-	-	-	-	-	-	-	SSP_RS11685
membrane protein	-	-	-	-	-	-	-	-	-	SSP_RS11880
hypothetical protein	-	-	-	-	-	-	-	-	-	SSP_RS12850
hypothetical protein	-	-	-	-	-	-	-	-	-	SSP_RS12630
glycine/betaine ABC transporter	-	-	-	-	-	-	-	-	-	SSP_RS12190
hypothetical protein	-	-	-	-	-	-	-	-	-	SSP_RS12225
hypothetical protein	-	-	-	-	BK815_RS00705	-	-	-	-	-
VOC family virulence protein	-	-	-	-	BK815_RS01415	-	-	-	-	-
transcriptional regulator	-	-	-	-	BK815_RS02615	-	-	-	-	-
N-acyl homoserine lactonase family protein	-	-	-	-	BK815_RS02620	-	-	-	-	-
XRE family transcriptional regulator	-	-	-	-	BK815_RS02670	-	-	-	-	-
XRE family transcriptional regulator	-	-	-	-	BK815_RS02675	-	-	-	-	-
hypothetical protein	-	-	-	-	BK815_RS02690	-	-	-	-	-
hypothetical protein	-	-	-	-	BK815_RS02770	-	-	-	-	-
hypothetical protein	-	-	-	-	BK815_RS02775	-	-	-	-	-
hypothetical protein	-	-	-	-	BK815_RS02790	-	-	-	-	-
hypothetical protein	-	-	-	-	BK815_RS02795	-	-	-	-	-
hypothetical protein	-	-	-	-	BK815_RS02800	-	-	-	-	-

Product	*S. carnosus*		*S. equorum*		*S. succinus*			*S. xylosus*		*S. saprophyticus*
	**JCM 6069**	**TM300**	**KS1039**	**Mu2**	**14BME20**	**CSM 77**	**DSM 14617**	**C2a**	**HKUOPL8**	**ATCC 15305**
terminase	-	-	-	-	BK815_RS02810	-	-	-	-	-
hypothetical protein	-	-	-	-	BK815_RS02835	-	-	-	-	-
hypothetical protein	-	-	-	-	BK815_RS02920	-	-	-	-	-
hypothetical protein	-	-	-	-	BK815_RS02925	-	-	-	-	-
hypothetical protein	-	-	-	-	BK815_RS02930	-	-	-	-	-
hypothetical protein	-	-	-	-	BK815_RS02935	-	-	-	-	-
phosphoglucomutase	-	-	-	-	BK815_RS03790	-	-	-	-	-
LysE family translocator	-	-	-	-	BK815_RS03865	-	-	-	-	-
YqcI/YcgG family protein	-	-	-	-	BK815_RS03870	-	-	-	-	-
hypothetical protein	-	-	-	-	BK815_RS03875	-	-	-	-	-
hypothetical protein	-	-	-	-	BK815_RS03890	-	-	-	-	-
hypothetical protein	-	-	-	-	BK815_RS03895	-	-	-	-	-
hypothetical protein	-	-	-	-	BK815_RS05035	-	-	-	-	-
XRE family transcriptional regulator	-	-	-	-	BK815_RS05040	-	-	-	-	-
hypothetical protein	-	-	-	-	BK815_RS05050	-	-	-	-	-
hypothetical protein	-	-	-	-	BK815_RS06360	-	-	-	-	-
glyoxalase	-	-	-	-	BK815_RS07280	-	-	-	-	-
MFS transporter	-	-	-	-	BK815_RS08645	-	-	-	-	-
hypothetical protein	-	-	-	-	BK815_RS09185	-	-	-	-	-
nitrilotriacetate monooxygenase	-	-	-	-	BK815_RS09200	-	-	-	-	-
transcriptional regulator	-	-	-	-	BK815_RS09205	-	-	-	-	-
alpha/beta hydrolase	-	-	-	-	BK815_RS09210	-	-	-	-	-
hypothetical protein	-	-	-	-	BK815_RS09220	-	-	-	-	-
amino acid permease	-	-	-	-	BK815_RS09225	-	-	-	-	-
MarR family transcriptional regulator	-	-	-	-	BK815_RS09240	-	-	-	-	-
hypothetical protein	-	-	-	-	BK815_RS09245	-	-	-	-	-
LysR family transcriptional regulator	-	-	-	-	BK815_RS09250	-	-	-	-	-
hypothetical protein	-	-	-	-	BK815_RS09270	-	-	-	-	-
short-chain dehydrogenase	-	-	-	-	BK815_RS09275	-	-	-	-	-
TetR/AcrR family transcriptional regulator	-	-	-	-	BK815_RS09280	-	-	-	-	-
restriction endonuclease	-	-	-	-	BK815_RS09675	-	-	-	-	-

Product	S. carnosus		S. equorum		S. succinus			S. xylosus		S. saprophyticus
	**JCM 6069**	**TM300**	**KS1039**	**Mu2**	**14BME20**	**CSM 77**	**DSM 14617**	**C2a**	**HKUOPL8**	**ATCC 15305**
hypothetical protein	-	-	-	-	BK815_RS09680	-	-	-	-	-
hypothetical protein	-	-	-	-	BK815_RS09730	-	-	-	-	-
ATP-binding protein	-	-	-	-	BK815_RS09740	-	-	-	-	-
abortive phage resistance protein	-	-	-	-	BK815_RS09745	-	-	-	-	-
hypothetical protein	-	-	-	-	BK815_RS09750	-	-	-	-	-
hypothetical protein	-	-	-	-	BK815_RS09765	-	-	-	-	-
hypothetical protein	-	-	-	-	BK815_RS09935	-	-	-	-	-
hypothetical protein	-	-	-	-	BK815_RS09945	-	-	-	-	-
hypothetical protein	-	-	-	-	BK815_RS10500	-	-	-	-	-
amino acid permease	-	-	-	-	BK815_RS13005	-	-	-	-	-
DUF2335 domain-containing protein	-	-	-	-	-	A6V26_RS10985	-	-	-	-
hypothetical protein	-	-	-	-	-	A6V26_RS10990	-	-	-	-
hypothetical protein	-	-	-	-	-	A6V26_RS11025	-	-	-	-
DUF771 domain-containing protein	-	-	-	-	-	A6V26_RS11230	-	-	-	-
hypothetical protein	-	-	-	-	-	A6V26_RS11235	-	-	-	-
hypothetical protein	-	-	-	-	-	A6V26_RS11240	-	-	-	-
hypothetical protein	-	-	-	-	-	A6V26_RS11245	-	-	-	-
DUF3102 domain-containing protein	-	-	-	-	-	A6V26_RS11250	-	-	-	-
XRE family transcriptional regulator	-	-	-	-	-	A6V26_RS11255	-	-	-	-
hypothetical protein	-	-	-	-	-	A6V26_RS08895	-	-	-	-
hypothetical protein	-	-	-	-	-	A6V26_RS05010	-	-	-	-
hypothetical protein	-	-	-	-	-	A6V26_RS02460	-	-	-	-
ArgR family transcriptional regulator	-	-	-	-	-	-	AA913_RS14110	-	-	-
hypothetical protein	-	-	-	-	-	-	AA913_RS14050	-	-	-
hypothetical protein	-	-	-	-	-	-	AA913_RS14025	-	-	-
hypothetical protein	-	-	-	-	-	-	AA913_RS14000	-	-	-
hypothetical protein	-	-	-	-	-	-	AA913_RS13785	-	-	-
hypothetical protein	-	-	-	-	-	-	AA913_RS13690	-	-	-
hypothetical protein	-	-	-	-	-	-	AA913_RS13685	-	-	-
hypothetical protein	-	-	-	-	-	-	AA913_RS13655	-	-	-
DUF1474 domain-containing protein	-	-	-	-	-	-	AA913_RS13650	-	-	-

Product	*S. carnosus*		*S. equorum*		*S. succinus*			*S. xylosus*		*S. saprophyticus*
	**JCM 6069**	**TM300**	**KS1039**	**Mu2**	**14BME20**	**CSM 77**	**DSM 14617**	**C2a**	**HKUOPL8**	**ATCC 15305**
hypothetical protein	-	-	-	-	-	-	AA913_RS13645	-	-	-
conjugal transfer protein	-	-	-	-	-	-	AA913_RS13620	-	-	-
hypothetical protein	-	-	-	-	-	-	AA913_RS13530	-	-	-
hypothetical protein	-	-	-	-	-	-	AA913_RS13515	-	-	-
hypothetical protein	-	-	-	-	-	-	AA913_RS13480	-	-	-
integrase	-	-	-	-	-	-	AA913_RS13485	-	-	-
conjugal transfer protein	-	-	-	-	-	-	AA913_RS13455	-	-	-
cell division protein FtsK	-	-	-	-	-	-	AA913_RS13440	-	-	-
glutamine amidotransferase	-	-	-	-	-	-	AA913_RS13385	-	-	-
hypothetical protein	-	-	-	-	-	-	AA913_RS14405	-	-	-
IS3 family transposase	-	-	-	-	-	-	AA913_RS14195	-	-	-
hypothetical protein	-	-	-	-	-	-	AA913_RS13310	-	-	-
hypothetical protein	-	-	-	-	-	-	AA913_RS13315	-	-	-
hypothetical protein	-	-	-	-	-	-	AA913_RS13320	-	-	-
DDE transposase	-	-	-	-	-	-	AA913_RS13255	-	-	-
group II intron reverse transcriptase/maturase	-	-	-	-	-	-	AA913_RS13260	-	-	-
accessory Sec system glycosylation chaperone GtfB	-	-	-	-	-	-	AA913_RS13265	-	-	-
accessory Sec system protein Asp3	-	-	-	-	-	-	AA913_RS14185	-	-	-
hypothetical protein	-	-	-	-	-	-	AA913_RS14380	-	-	-
hypothetical protein	-	-	-	-	-	-	AA913_RS12880	-	-	-
hypothetical protein	-	-	-	-	-	-	AA913_RS10305	-	-	-
hypothetical protein	-	-	-	-	-	-	AA913_RS10310	-	-	-
hypothetical protein	-	-	-	-	-	-	AA913_RS10315	-	-	-
transcriptional regulator	-	-	-	-	-	-	AA913_RS08685	-	-	-
dihydrolipoamide dehydrogenase	-	-	-	-	-	-	AA913_RS08695	-	-	-
permease	-	-	-	-	-	-	AA913_RS08700	-	-	-
transcriptional regulator	-	-	-	-	-	-	AA913_RS08715	-	-	-
hypothetical protein	-	-	-	-	-	-	AA913_RS08720	-	-	-
hypothetical protein	-	-	-	-	-	-	AA913_RS08725	-	-	-
hypothetical protein	-	-	-	-	-	-	AA913_RS08730	-	-	-
hypothetical protein	-	-	-	-	-	-	AA913_RS08735	-	-	-

Product	*S. carnosus*		*S. equorum*		*S. succinus*			*S. xylosus*		*S. saprophyticus*
	**JCM 6069**	**TM300**	**KS1039**	**Mu2**	**14BME20**	**CSM 77**	**DSM 14617**	**C2a**	**HKUOPL8**	**ATCC 15305**
hypothetical protein	-	-	-	-	-	-	AA913_RS08740	-	-	-
hypothetical protein	-	-	-	-	-	-	AA913_RS08750	-	-	-
DUF4176 domain-containing protein	-	-	-	-	-	-	AA913_RS08755	-	-	-
AbrB/MazE/SpoVT family DNA-binding domain-containing protein	-	-	-	-	-	-	AA913_RS08760	-	-	-
ParA family protein	-	-	-	-	-	-	AA913_RS08765	-	-	-
hypothetical protein	-	-	-	-	-	-	AA913_RS08770	-	-	-
hypothetical protein	-	-	-	-	-	-	AA913_RS14160	-	-	-
hypothetical protein	-	-	-	-	-	-	AA913_RS08780	-	-	-
hypothetical protein	-	-	-	-	-	-	AA913_RS08785	-	-	-
RusA family crossover junction endodeoxyribonuclease	-	-	-	-	-	-	AA913_RS08790	-	-	-
hypothetical protein	-	-	-	-	-	-	AA913_RS08795	-	-	-
type II toxin-antitoxin system RelE/ParE family toxin	-	-	-	-	-	-	AA913_RS08800	-	-	-
prevent-host-death protein	-	-	-	-	-	-	AA913_RS08805	-	-	-
hypothetical protein	-	-	-	-	-	-	AA913_RS06120	-	-	-
autolysin	-	-	-	-	-	-	AA913_RS06140	-	-	-
DUF2479 domain-containing protein	-	-	-	-	-	-	AA913_RS14145	-	-	-
hypothetical protein	-	-	-	-	-	-	AA913_RS06165	-	-	-
hypothetical protein	-	-	-	-	-	-	AA913_RS06170	-	-	-
hypothetical protein	-	-	-	-	-	-	AA913_RS06380	-	-	-
XRE family transcriptional regulator	-	-	-	-	-	-	AA913_RS06385	-	-	-
hypothetical protein	-	-	-	-	-	-	-	SXYL_RS00145	-	-
transporter	-	-	-	-	-	-	-	SXYL_RS00160	-	-
hypothetical protein	-	-	-	-	-	-	-	SXYL_RS00175	-	-
inositol 2-dehydrogenase	-	-	-	-	-	-	-	SXYL_RS00180	-	-
hypothetical protein	-	-	-	-	-	-	-	SXYL_RS13240	-	-
hypothetical protein	-	-	-	-	-	-	-	SXYL_RS01195	-	-
hypothetical protein	-	-	-	-	-	-	-	SXYL_RS13190	-	-
MazF/PemK family toxin	-	-	-	-	-	-	-	SXYL_RS05185	-	-
hypothetical protein	-	-	-	-	-	-	-	SXYL_RS05195	-	-
phage tail family protein	-	-	-	-	-	-	-	SXYL_RS05200	-	-
hypothetical protein	-	-	-	-	-	-	-	SXYL_RS05210	-	-

Product	*S. carnosus*		*S. equorum*		*S. succinus*			*S. xylosus*		*S. saprophyticus*
	**JCM 6069**	**TM300**	**KS1039**	**Mu2**	**14BME20**	**CSM 77**	**DSM 14617**	**C2a**	**HKUOPL8**	**ATCC 15305**
hypothetical protein	-	-	-	-	-	-	-	SXYL_RS05215	-	-
hypothetical protein	-	-	-	-	-	-	-	SXYL_RS05220	-	-
hypothetical protein	-	-	-	-	-	-	-	SXYL_RS13200	-	-
hypothetical protein	-	-	-	-	-	-	-	SXYL_RS05230	-	-
hypothetical protein	-	-	-	-	-	-	-	SXYL_RS05260	-	-
hypothetical protein	-	-	-	-	-	-	-	SXYL_RS05265	-	-
hypothetical protein	-	-	-	-	-	-	-	SXYL_RS05270	-	-
hypothetical protein	-	-	-	-	-	-	-	SXYL_RS05275	-	-
hypothetical protein	-	-	-	-	-	-	-	SXYL_RS07070	-	-
hypothetical protein	-	-	-	-	-	-	-	SXYL_RS07075	-	-
hypothetical protein	-	-	-	-	-	-	-	SXYL_RS07080	-	-
hypothetical protein	-	-	-	-	-	-	-	SXYL_RS07095	-	-
hypothetical protein	-	-	-	-	-	-	-	SXYL_RS07100	-	-
hypothetical protein	-	-	-	-	-	-	-	SXYL_RS07105	-	-
hypothetical protein	-	-	-	-	-	-	-	SXYL_RS07110	-	-
hypothetical protein	-	-	-	-	-	-	-	SXYL_RS07120	-	-
hypothetical protein	-	-	-	-	-	-	-	SXYL_RS07125	-	-
hypothetical protein	-	-	-	-	-	-	-	SXYL_RS07130	-	-
hypothetical protein	-	-	-	-	-	-	-	SXYL_RS07135	-	-
arsenic transporter	-	-	-	-	-	-	-	SXYL_RS13285	-	-
hypothetical protein	-	-	-	-	-	-	-	SXYL_RS07715	-	-
hypothetical protein	-	-	-	-	-	-	-	SXYL_RS07815	-	-
hypothetical protein	-	-	-	-	-	-	-	SXYL_RS08475	-	-
hypothetical protein	-	-	-	-	-	-	-	SXYL_RS08485	-	-
hypothetical protein	-	-	-	-	-	-	-	SXYL_RS08490	-	-
hypothetical protein	-	-	-	-	-	-	-	SXYL_RS08495	-	-
hypothetical protein	-	-	-	-	-	-	-	SXYL_RS08505	-	-
phage tail tape measure protein	-	-	-	-	-	-	-	SXYL_RS13305	-	-
phage tail protein	-	-	-	-	-	-	-	SXYL_RS08580	-	-
hypothetical protein	-	-	-	-	-	-	-	SXYL_RS08595	-	-
transcriptional regulator	-	-	-	-	-	-	-	SXYL_RS08640	-	-

Product	*S. carnosus*		*S. equorum*		*S. succinus*			*S. xylosus*		*S. saprophyticus*
	**JCM 6069**	**TM300**	**KS1039**	**Mu2**	**14BME20**	**CSM 77**	**DSM 14617**	**C2a**	**HKUOPL8**	**ATCC 15305**
hypothetical protein	-	-	-	-	-	-	-	SXYL_RS08650	-	-
hypothetical protein	-	-	-	-	-	-	-	SXYL_RS08665	-	-
HNH endonuclease	-	-	-	-	-	-	-	SXYL_RS08680	-	-
hypothetical protein	-	-	-	-	-	-	-	SXYL_RS08685	-	-
hypothetical protein	-	-	-	-	-	-	-	SXYL_RS08700	-	-
hypothetical protein	-	-	-	-	-	-	-	SXYL_RS08750	-	-
hypothetical protein	-	-	-	-	-	-	-	SXYL_RS08760	-	-
oxidoreductase	-	-	-	-	-	-	-	SXYL_RS08765	-	-
helix-turn-helix domain-containing protein	-	-	-	-	-	-	-	SXYL_RS08770	-	-
hypothetical protein	-	-	-	-	-	-	-	SXYL_RS13210	-	-
integrase	-	-	-	-	-	-	-	SXYL_RS08790	-	-
hypothetical protein	-	-	-	-	-	-	-	SXYL_RS10065	-	-
hypothetical protein	-	-	-	-	-	-	-	SXYL_RS10080	-	-
hypothetical protein	-	-	-	-	-	-	-	SXYL_RS13215	-	-
hypothetical protein	-	-	-	-	-	-	-	SXYL_RS13230	-	-
hypothetical protein	-	-	-	-	-	-	-	SXYL_RS12685	-	-
hypothetical protein	-	-	-	-	-	-	-	SXYL_RS13020	-	-
LacI family transcriptional regulator	-	-	-	-	-	-	-	SXYL_RS13025	-	-
metal ABC transporter	-	-	-	-	-	-	-	SXYL_RS13030	-	-
dihydrodipicolinate synthase family protein	-	-	-	-	-	-	-	SXYL_RS13035	-	-
UDP-N-acetylglucosamine 2-epimerase (non-hydrolyzing)	-	-	-	-	-	-	-	-	BE24_RS00230	-
hypothetical protein	-	-	-	-	-	-	-	-	BE24_RS00235	-
hypothetical protein	-	-	-	-	-	-	-	-	BE24_RS00240	-
hypothetical protein	-	-	-	-	-	-	-	-	BE24_RS00270	-
ester cyclase	-	-	-	-	-	-	-	-	BE24_RS00285	-
hypothetical protein	-	-	-	-	-	-	-	-	BE24_RS01650	-
hemolytic protein	-	-	-	-	-	-	-	-	BE24_RS13600	-
N-acetylmuramoyl-L-alanine amidase	-	-	-	-	-	-	-	-	BE24_RS05055	-
hypothetical protein	-	-	-	-	-	-	-	-	BE24_RS05075	-
hypothetical protein	-	-	-	-	-	-	-	-	BE24_RS05080	-
phage tail family protein	-	-	-	-	-	-	-	-	BE24_RS05085	-

Product	*S. carnosus*		*S. equorum*		*S. succinus*			*S. xylosus*		*S. saprophyticus*
	**JCM 6069**	**TM300**	**KS1039**	**Mu2**	**14BME20**	**CSM 77**	**DSM 14617**	**C2a**	**HKUOPL8**	**ATCC 15305**
hypothetical protein	-	-	-	-	-	-	-	-	BE24_RS05180	-
hypothetical protein	-	-	-	-	-	-	-	-	BE24_RS05185	-
hypothetical protein	-	-	-	-	-	-	-	-	BE24_RS05190	-
DNA cytosine methyltransferase	-	-	-	-	-	-	-	-	BE24_RS05200	-
hypothetical protein	-	-	-	-	-	-	-	-	BE24_RS05245	-
hypothetical protein	-	-	-	-	-	-	-	-	BE24_RS05285	-
hypothetical protein	-	-	-	-	-	-	-	-	BE24_RS05290	-
DNA-binding protein	-	-	-	-	-	-	-	-	BE24_RS05300	-
hypothetical protein	-	-	-	-	-	-	-	-	BE24_RS13465	-
MBL fold metallo-hydrolase	-	-	-	-	-	-	-	-	BE24_RS13470	-
hypothetical protein	-	-	-	-	-	-	-	-	BE24_RS06060	-
kinase	-	-	-	-	-	-	-	-	BE24_RS06335	-
hypothetical protein	-	-	-	-	-	-	-	-	BE24_RS06345	-
hypothetical protein	-	-	-	-	-	-	-	-	BE24_RS07395	-
hypothetical protein	-	-	-	-	-	-	-	-	BE24_RS07475	-
hypothetical protein	-	-	-	-	-	-	-	-	BE24_RS07960	-
hypothetical protein	-	-	-	-	-	-	-	-	BE24_RS07965	-
hypothetical protein	-	-	-	-	-	-	-	-	BE24_RS07970	-
hypothetical protein	-	-	-	-	-	-	-	-	BE24_RS13625	-
hypothetical protein	-	-	-	-	-	-	-	-	BE24_RS09315	-
hypothetical protein	-	-	-	-	-	-	-	-	BE24_RS13630	-
hypothetical protein	-	-	-	-	-	-	-	-	BE24_RS13635	-
succinyl-diaminopimelate desuccinylase	-	-	-	-	-	-	-	-	BE24_RS09830	-
transcriptional regulator	-	-	-	-	-	-	-	-	BE24_RS10020	-
hypothetical protein	-	-	-	-	-	-	-	-	BE24_RS10395	-
hypothetical protein	-	-	-	-	-	-	-	-	BE24_RS13480	-
non-ribosomal peptide synthetase	-	-	-	-	-	-	-	-	BE24_RS10400	-
hypothetical protein	-	-	-	-	-	-	-	-	BE24_RS10860	-
hypothetical protein	-	-	-	-	-	-	-	-	BE24_RS10940	-
YSIRK signal domain/LPXTG anchor domain surface protein	-	-	-	-	-	-	-	-	BE24_RS13490	-
intercellular adhesin biosynthesis polysaccharide N-deacetylase	-	-	-	-	-	-	-	-	BE24_RS11195	-

Product	S. carnosus		S. equorum		S. succinus			S. xylosus		S. saprophyticus
	JCM 6069	TM300	KS1039	Mu2	14BME20	CSM 77	DSM 14617	C2a	HKUOPL8	ATCC 15305
flavin reductase	-	-	-	-	-	-	-	-	BE24_RS11520	-
acetyltransferase	-	-	-	-	-	-	-	-	BE24_RS11530	-
FAD-binding dehydrogenase	-	-	-	-	-	-	-	-	BE24_RS11535	-
DNA-binding protein	-	-	-	-	-	-	-	-	BE24_RS11610	-
DNA (cytosine-5-)-methyltransferase	-	-	-	-	-	-	-	-	BE24_RS11615	-
DNA (cytosine-5-)-methyltransferase	-	-	-	-	-	-	-	-	BE24_RS11620	-
hypothetical protein	-	-	-	-	-	-	-	-	BE24_RS13495	-
hypothetical protein	-	-	-	-	-	-	-	-	BE24_RS11630	-
LlaJI family restriction endonuclease	-	-	-	-	-	-	-	-	BE24_RS11635	-
modification methylase	-	-	-	-	-	-	-	-	BE24_RS11645	-
hypothetical protein	-	-	-	-	-	-	-	-	BE24_RS11650	-
hypothetical protein	-	-	-	-	-	-	-	-	BE24_RS11655	-
hypothetical protein	-	-	-	-	-	-	-	-	BE24_RS11660	-
hypothetical protein	-	-	-	-	-	-	-	-	BE24_RS11665	-
acetyltransferase	-	-	-	-	-	-	-	-	BE24_RS11700	-
hypothetical protein	-	-	-	-	-	-	-	-	BE24_RS11715	-
LytR family transcriptional regulator	-	-	-	-	-	-	-	-	BE24_RS11750	-
pyridoxal phosphate-dependent aminotransferase	-	-	-	-	-	-	-	-	BE24_RS11755	-
hypothetical protein	-	-	-	-	-	-	-	-	BE24_RS11785	-
hypothetical protein	-	-	-	-	-	-	-	-	BE24_RS13510	-
hypothetical protein	-	-	-	-	-	-	-	-	BE24_RS11795	-
serine acetyltransferase	-	-	-	-	-	-	-	-	BE24_RS11800	-
hypothetical protein	-	-	-	-	-	-	-	-	BE24_RS11805	-
hypothetical protein	-	-	-	-	-	-	-	-	BE24_RS11810	-
hypothetical protein	-	-	-	-	-	-	-	-	BE24_RS11835	-
hypothetical protein	-	-	-	-	-	-	-	-	BE24_RS11840	-
SAM-dependent DNA methyltransferase	-	-	-	-	-	-	-	-	BE24_RS11845	-
type I restriction-modification system specificity determinant protein	-	-	-	-	-	-	-	-	BE24_RS11850	-
DEAD/DEAH box helicase	-	-	-	-	-	-	-	-	BE24_RS11855	-
hypothetical protein	-	-	-	-	-	-	-	-	BE24_RS11905	-
hypothetical protein	-	-	-	-	-	-	-	-	BE24_RS11910	-

Product	*S. carnosus*		*S. equorum*		*S. succinus*			*S. xylosus*		*S. saprophyticus*
	**JCM 6069**	**TM300**	**KS1039**	**Mu2**	**14BME20**	**CSM 77**	**DSM 14617**	**C2a**	**HKUOPL8**	**ATCC 15305**
hypothetical protein	-	-	-	-	-	-	-	-	BE24_RS13660	-
hypothetical protein	-	-	-	-	-	-	-	-	BE24_RS13520	-
hypothetical protein	-	-	-	-	-	-	-	-	BE24_RS12495	-
hypothetical protein	-	-	-	-	-	-	-	-	BE24_RS12590	-
non-ribosomal peptide synthetase	-	-	-	-	-	-	-	-	BE24_RS13530	-
XRE family transcriptional regulator	-	-	-	-	-	-	-	-	BE24_RS13545	-
hypothetical protein	-	-	-	-	-	-	-	-	BE24_RS12885	-
phage portal protein	-	-	-	-	-	-	-	-	BE24_RS12890	-
peptidase U35	-	-	-	-	-	-	-	-	BE24_RS12895	-
phage major capsid protein	-	-	-	-	-	-	-	-	BE24_RS12900	-
phage gp6-like head-tail connector protein	-	-	-	-	-	-	-	-	BE24_RS12905	-
HNH endonuclease	-	-	-	-	-	-	-	-	BE24_RS12910	-
hypothetical protein	-	-	-	-	-	-	-	-	BE24_RS12915	-
terminase	-	-	-	-	-	-	-	-	BE24_RS12920	-
hypothetical protein	-	-	-	-	-	-	-	-	BE24_RS12930	-
hypothetical protein	-	-	-	-	-	-	-	-	BE24_RS12935	-
hypothetical protein	-	-	-	-	-	-	-	-	BE24_RS13690	-

Table S2. List of singletons generated by comparing the genomes of 10 CNS genomes

Strain	Gene locus	Product
TM300	SCA_RS00035	hypothetical protein
	SCA_RS00090	5-amino-6-(5-phosphoribosylamino)uracil reductase
	SCA_RS00095	hypothetical protein
	SCA_RS00100	oxidoreductase
	SCA_RS00110	MazF/PemK family toxin
	SCA_RS00115	hypothetical protein
	SCA_RS00120	thermonuclease
	SCA_RS00140	hypothetical protein
	SCA_RS00145	monooxygenase IsdI
	SCA_RS00150	hypothetical protein
	SCA_RS00175	phosphatase
	SCA_RS00180	hypothetical protein
	SCA_RS12605	hypothetical protein
	SCA_RS12610	hypothetical protein
	SCA_RS00250	hypothetical protein
	SCA_RS00255	NUDIX hydrolase
	SCA_RS00260	hypothetical protein
	SCA_RS00270	hypothetical protein
	SCA_RS00340	hypothetical protein
	SCA_RS00360	hypothetical protein
	SCA_RS12435	hypothetical protein
	SCA_RS00370	hypothetical protein
	SCA_RS00375	DNA-binding protein
	SCA_RS00380	type 1 glutamine amidotransferase domain-containing protein
	SCA_RS00385	membrane protein
	SCA_RS00445	bifunctional metallophosphatase/5-nucleotidase
	SCA_RS00455	hypothetical protein
	SCA_RS00460	DNA mismatch repair protein MutT
	SCA_RS00465	hypothetical protein
	SCA_RS00470	patatin
	SCA_RS00475	hypothetical protein
	SCA_RS00535	transcriptional regulator
	SCA_RS00540	hypothetical protein
	SCA_RS00555	hypothetical protein
	SCA_RS12440	lipase (fragment 1)
	SCA_RS12445	lipase (fragment 2)
	SCA_RS00645	DUF5079 domain-containing protein
	SCA_RS00650	DUF5085 domain-containing protein
	SCA_RS00655	hypothetical protein
	SCA_RS00660	hypothetical protein
	SCA_RS00665	putative ABC transporter ATP-binding protein
	SCA_RS00670	hypothetical protein
	SCA_RS00755	hypothetical protein
	SCA_RS00815	tRNA dihydrouridine synthase DusB
	SCA_RS00890	N-acetyltransferase

Strain	Gene locus	Product
	SCA_RS00985	RNA polymerase sigma factor
	SCA_RS01035	hypothetical protein
	SCA_RS01085	N-acetyltransferase
	SCA_RS01120	hydrolase
	SCA_RS01210	DedA family protein
	SCA_RS01215	hypothetical protein
	SCA_RS01265	hypothetical protein
	SCA_RS01305	prevent-host-death protein
	SCA_RS01310	Txe/YoeB family addiction module toxin
	SCA_RS01365	hypothetical protein
	SCA_RS01415	phosphatase PAP2 family protein
	SCA_RS01420	N-acetyltransferase
	SCA_RS01450	membrane protein
	SCA_RS01595	hypothetical protein
	SCA_RS01600	AraC family transcriptional regulator
	SCA_RS01605	hypothetical protein
	SCA_RS01610	hypothetical protein
	SCA_RS12625	hypothetical protein
	SCA_RS01760	hypothetical protein
	SCA_RS01810	iron-sulfur cluster repair protein ScdA
	SCA_RS01960	hypothetical protein
	SCA_RS02120	MFS transporter
	SCA_RS02145	DUF4887 domain-containing protein
	SCA_RS12635	hypothetical protein
	SCA_RS02210	hypothetical protein
	SCA_RS02215	hypothetical protein
	SCA_RS02220	superantigen-like protein
	SCA_RS02225	cold-shock protein
	SCA_RS02235	hypothetical protein
	SCA_RS02250	LemA family protein
	SCA_RS02255	hypothetical protein
	SCA_RS02260	cupin
	SCA_RS02270	hypothetical protein
	SCA_RS02355	site-specific integrase
	SCA_RS02360	hypothetical protein
	SCA_RS02365	hypothetical protein
	SCA_RS02370	transcriptional regulator
	SCA_RS02375	transcriptional regulator
	SCA_RS02380	antirepressor
	SCA_RS02385	hypothetical protein
	SCA_RS02390	hypothetical protein
	SCA_RS02395	hypothetical protein
	SCA_RS02400	hypothetical protein
	SCA_RS02405	hypothetical protein
	SCA_RS02410	hypothetical protein
	SCA_RS02425	single-stranded DNA-binding protein
	SCA_RS02430	HNH endonuclease

Strain	Gene locus	Product
	SCA_RS02440	hypothetical protein
	SCA_RS02450	hypothetical protein
	SCA_RS02460	hypothetical protein
	SCA_RS12455	hypothetical protein
	SCA_RS02470	hypothetical protein
	SCA_RS02475	hypothetical protein
	SCA_RS02480	hypothetical protein
	SCA_RS02485	dUTP pyrophosphatase
	SCA_RS02490	hypothetical protein
	SCA_RS02495	DUF1381 domain-containing protein
	SCA_RS02500	hypothetical protein
	SCA_RS02505	hypothetical protein
	SCA_RS02510	hypothetical protein
	SCA_RS02515	hypothetical protein
	SCA_RS02525	HNH endonuclease
	SCA_RS02530	hypothetical protein
	SCA_RS02535	terminase
	SCA_RS02540	phage portal protein
	SCA_RS02545	peptidase
	SCA_RS02550	phage major capsid protein
	SCA_RS02555	hypothetical protein
	SCA_RS02560	hypothetical protein
	SCA_RS02570	hypothetical protein
	SCA_RS02575	tail protein
	SCA_RS02580	tail protein
	SCA_RS02585	hypothetical protein
	SCA_RS12640	phage tail tape measure protein
	SCA_RS02595	hypothetical protein
	SCA_RS02610	hypothetical protein
	SCA_RS02615	hypothetical protein
	SCA_RS12460	hypothetical protein
	SCA_RS02645	phage holin
	SCA_RS02655	hypothetical protein
	SCA_RS02660	hypothetical protein
	SCA_RS02665	glycerophosphoryl diester phosphodiesterase
	SCA_RS02670	hypothetical protein
	SCA_RS02775	acetyltransferase
	SCA_RS02865	hypothetical protein
	SCA_RS02870	NUDIX domain-containing protein
	SCA_RS02900	amidohydrolase
	SCA_RS03060	hypothetical protein
	SCA_RS12475	hypothetical protein
	SCA_RS12480	hypothetical protein
	SCA_RS03120	MFS transporter
	SCA_RS03240	alpha/beta hydrolase
	SCA_RS03245	transcriptional regulator
	SCA_RS03250	hypothetical protein

Strain	Gene locus	Product
	SCA_RS12485	hypothetical protein
	SCA_RS12490	Fic protein family protein (fragment 2)
	SCA_RS03295	hypothetical protein
	SCA_RS03395	PTS lactose transporter subunit IIB
	SCA_RS03400	6-phospho-beta-galactosidase
	SCA_RS03415	tagatose-bisphosphate aldolase
	SCA_RS03420	tagatose-6-phosphate kinase
	SCA_RS03425	galactose-6-phosphate isomerase subunit LacB
	SCA_RS03430	galactose-6-phosphate isomerase LacA subunit
	SCA_RS03435	DeoR/GlpR transcriptional regulator
	SCA_RS03540	hypothetical protein
	SCA_RS03605	6-phospho-3-hexuloisomerase
	SCA_RS03630	hypothetical protein
	SCA_RS03700	hypothetical protein
	SCA_RS03805	ornithine cyclodeaminase
	SCA_RS03890	oxidoreductase
	SCA_RS03895	MarR family transcriptional regulator
	SCA_RS03915	hypothetical protein
	SCA_RS03935	hypothetical protein
	SCA_RS03940	hypothetical protein
	SCA_RS12495	hypothetical protein
	SCA_RS03955	hypothetical protein
	SCA_RS12500	hypothetical protein
	SCA_RS12505	hypothetical protein
	SCA_RS03985	hypothetical protein
	SCA_RS03990	hypothetical protein
	SCA_RS04140	dihydroorotate dehydrogenase electron transfer subunit
	SCA_RS04145	dihydroorotate dehydrogenase
	SCA_RS04170	capsular polysaccharide biosynthesis protein Cap8C
	SCA_RS04210	putative phosphotyrosine-protein phosphatase
	SCA_RS04215	hypothetical protein
	SCA_RS04220	hypothetical protein
	SCA_RS04270	hypothetical protein
	SCA_RS04380	hypothetical protein
	SCA_RS04385	hypothetical protein
	SCA_RS04400	AraC family transcriptional regulator
	SCA_RS04405	alpha/beta hydrolase
	SCA_RS04410	DeoR/GlpR transcriptional regulator
	SCA_RS04420	iron ABC transporter permease
	SCA_RS04435	2-aminoethylphosphonate--pyruvate transaminase
	SCA_RS04440	phosphonoacetaldehyde hydrolase
	SCA_RS04565	exotoxin-like protein
	SCA_RS04580	MFS transporter
	SCA_RS04700	hypothetical protein
	SCA_RS04720	ABC transporter ATP-binding protein
	SCA_RS04725	ABC transporter permease
	SCA_RS04730	ABC transporter permease

Strain	Gene locus	Product
	SCA_RS04825	hypothetical protein
	SCA_RS04845	iron ABC transporter substrate-binding protein
	SCA_RS04855	hypothetical protein
	SCA_RS05020	PTS glucoside EIICBA component
	SCA_RS05040	homoserine O-succinyltransferase
	SCA_RS05570	hypothetical protein
	SCA_RS05680	hypothetical protein
	SCA_RS05820	hypothetical protein
	SCA_RS06000	tRNA (N(6)-L-threonylcarbamoyladenosine(37)-C(2))-methylthiotransferase MtaB
	SCA_RS06680	hypothetical protein
	SCA_RS12515	hypothetical protein
	SCA_RS06875	ADP-ribosylglycohydrolase family protein
	SCA_RS06930	hypothetical protein
	SCA_RS06935	hypothetical protein
	SCA_RS06940	hypothetical protein
	SCA_RS06985	hypothetical protein
	SCA_RS06990	SAM-dependent methyltransferase
	SCA_RS12675	hypothetical protein
	SCA_RS12520	hypothetical protein
	SCA_RS07410	carbonic anhydrase
	SCA_RS07465	general stress protein
	SCA_RS07600	hypothetical protein
	SCA_RS07610	hypothetical protein
	SCA_RS07615	hypothetical protein
	SCA_RS07670	hypothetical protein
	SCA_RS07680	threonine aldolase
	SCA_RS07685	hypothetical protein
	SCA_RS07690	hypothetical protein
	SCA_RS07695	hypothetical protein
	SCA_RS07710	hypothetical protein
	SCA_RS07715	hypothetical protein
	SCA_RS07720	phage-associated protein
	SCA_RS07745	hypothetical protein
	SCA_RS07770	hypothetical protein
	SCA_RS07780	excisionase
	SCA_RS07790	transcriptional regulator
	SCA_RS07800	site-specific integrase
	SCA_RS07820	hypothetical protein
	SCA_RS07830	delta-hemolysin
	SCA_RS08140	DNA-directed RNA polymerase subunit beta
	SCA_RS08275	hypothetical protein
	SCA_RS08400	transposase
	SCA_RS08430	methyltransferase
	SCA_RS08625	NADP-dependent oxidoreductase
	SCA_RS08645	hypothetical protein
	SCA_RS08650	hypothetical protein

Strain	Gene locus	Product
	SCA_RS08655	histidine phosphatase family protein
	SCA_RS08855	N-acetyltransferase
	SCA_RS08895	amidohydrolase family protein
	SCA_RS08900	hypothetical protein
	SCA_RS09170	hypothetical protein
	SCA_RS09195	hypothetical protein
	SCA_RS09315	hypothetical protein
	SCA_RS09330	hypothetical protein
	SCA_RS09340	DUF805 domain-containing protein
	SCA_RS09345	DUF805 domain-containing protein
	SCA_RS09350	hypothetical protein
	SCA_RS09355	DUF805 domain-containing protein
	SCA_RS09375	hypothetical protein
	SCA_RS09395	hypothetical protein
	SCA_RS12530	putative transcriptional regulator
	SCA_RS09635	NirR protein
	SCA_RS09645	hypothetical protein
	SCA_RS09665	ferrous iron transporter B
	SCA_RS09670	hypothetical protein
	SCA_RS09735	amino acid permease
	SCA_RS09740	hypothetical protein
	SCA_RS09770	hypothetical protein
	SCA_RS09775	hypothetical protein
	SCA_RS09780	TetR family transcriptional regulator
	SCA_RS09820	carboxylesterase/lipase family protein
	SCA_RS09840	CPBP family intramembrane metalloprotease
	SCA_RS09850	hypothetical protein
	SCA_RS09865	acetylornithine deacetylase
	SCA_RS09870	hypothetical protein
	SCA_RS09900	ATP-dependent Clp protease ATP-binding subunit
	SCA_RS09945	ABC-2 transporter family protein
	SCA_RS09990	acetyltransferase
	SCA_RS10005	lactate dehydrogenase
	SCA_RS10035	hypothetical protein
	SCA_RS10050	nickel transporter NixA
	SCA_RS10055	branched-chain amino acid transporter AzlD
	SCA_RS10060	branched-chain amino acid ABC transporter permease
	SCA_RS10075	2-pyrone-4
	SCA_RS10130	hypothetical protein
	SCA_RS10145	hypothetical protein
	SCA_RS10150	hypothetical protein
	SCA_RS10155	FMN-dependent NADH-azoreductase
	SCA_RS10160	cation transporter
	SCA_RS10165	polyphosphate--AMP phosphotransferase
	SCA_RS10195	hypothetical protein
	SCA_RS10225	DNA-binding protein
	SCA_RS10235	hypothetical protein

Strain	Gene locus	Product
	SCA_RS10255	hypothetical protein
	SCA_RS10275	hypothetical protein
	SCA_RS10280	serine/threonine protein phosphatase
	SCA_RS10420	hypothetical protein
	SCA_RS12700	hypothetical protein
	SCA_RS10425	NAD-dependent dehydratase
	SCA_RS10445	DUF3106 domain-containing protein
	SCA_RS10450	hypothetical protein
	SCA_RS10475	serine protease
	SCA_RS10505	hypothetical protein
	SCA_RS10510	transcriptional regulator
	SCA_RS10515	Crp/Fnr family transcriptional regulator
	SCA_RS10520	arginine-ornithine antiporter
	SCA_RS10525	arginine deiminase
	SCA_RS10530	arginine deiminase
	SCA_RS10535	hypothetical protein
	SCA_RS10545	hypothetical protein
	SCA_RS10550	hypothetical protein
	SCA_RS10560	YSIRK signal domain/LPXTG anchor domain surface protein
	SCA_RS10590	transcriptional regulator
	SCA_RS10595	hypothetical protein
	SCA_RS10600	CoA-disulfide reductase
	SCA_RS10610	aldehyde dehydrogenase
	SCA_RS10625	amino acid permease
	SCA_RS12705	hypothetical protein
	SCA_RS10635	glycerol-3-phosphate cytidylyltransferase
	SCA_RS12710	CDP-glycerol:glycerophosphate glycerophosphotransferase
	SCA_RS10685	hypothetical protein
	SCA_RS12535	hypothetical protein
	SCA_RS10690	alanine glycine permease
	SCA_RS10700	hypothetical protein
	SCA_RS10745	hypothetical protein
	SCA_RS10760	hypothetical protein
	SCA_RS10765	hypothetical protein
	SCA_RS10770	formate dehydrogenase
	SCA_RS10775	alpha/beta hydrolase
	SCA_RS10795	DUF871 domain-containing protein
	SCA_RS10815	MOSC domain-containing protein
	SCA_RS10860	TetR/AcrR family transcriptional regulator
	SCA_RS10865	sulfatase
	SCA_RS10870	hypothetical protein
	SCA_RS10875	oligosaccharide repeat unit polymerase
	SCA_RS10880	hypothetical protein
	SCA_RS10885	sugar transferase
	SCA_RS10890	NAD(P)-dependent oxidoreductase
	SCA_RS10895	hypothetical protein
	SCA_RS10935	hypothetical protein

Strain	Gene locus	Product
	SCA_RS10940	hypothetical protein
	SCA_RS10945	hypothetical protein
	SCA_RS10955	alpha/beta hydrolase
	SCA_RS10960	TetR family transcriptional regulator
	SCA_RS10990	glycosyl transferase family 1
	SCA_RS11000	teichoic acid biosynthesis protein
	SCA_RS11005	hypothetical protein
	SCA_RS11010	hypothetical protein
	SCA_RS12540	CDP-glycerol glycerophosphotransferase family protein
	SCA_RS11025	hypothetical protein
	SCA_RS11030	AraC family transcriptional regulator
	SCA_RS11035	alpha/beta hydrolase
	SCA_RS11060	hypothetical protein
	SCA_RS12545	hypothetical protein
	SCA_RS11080	3-methyladenine DNA glycosylase 2
	SCA_RS11095	accessory Sec system translocase SecA2
	SCA_RS11100	accessory Sec system protein Asp3
	SCA_RS11105	accessory Sec system protein Asp2
	SCA_RS11110	accessory Sec system protein Asp1
	SCA_RS11115	accessory Sec system protein translocase subunit SecY2
	SCA_RS11120	hypothetical protein
	SCA_RS12560	hypothetical protein
	SCA_RS12565	hypothetical protein
	SCA_RS12570	hypothetical protein
	SCA_RS12575	hypothetical protein
	SCA_RS11135	C4-dicarboxylate ABC transporter
	SCA_RS11140	hypothetical protein
	SCA_RS11155	twin-arginine translocase TatA/TatE family subunit
	SCA_RS11160	twin arginine-targeting protein translocase TatC
	SCA_RS11165	hypothetical protein
	SCA_RS11170	deferrochelatase/peroxidase EfeB
	SCA_RS11175	EfeM/EfeO family lipoprotein
	SCA_RS11195	hypothetical protein
	SCA_RS11200	hypothetical protein
	SCA_RS12580	hypothetical protein
	SCA_RS11210	hypothetical protein
	SCA_RS11225	BioY family transporter
	SCA_RS11230	amidohydrolase
	SCA_RS11235	thiaminase II
	SCA_RS11240	hypothetical protein
	SCA_RS11265	short-chain dehydrogenase
	SCA_RS11270	TetR family transcriptional regulator
	SCA_RS11275	NmrA/HSCARG family protein
	SCA_RS11280	CPBP family intramembrane metalloprotease
	SCA_RS11290	serine/threonine protein phosphatase
	SCA_RS11295	iron ABC transporter permease
	SCA_RS11300	ABC transporter substrate-binding protein

Strain	Gene locus	Product
	SCA_RS12585	ABC transporter ATP-binding protein
	SCA_RS11310	LysR family transcriptional regulator
	SCA_RS11315	hypothetical protein
	SCA_RS11320	hypothetical protein
	SCA_RS11325	membrane protein
	SCA_RS11335	RNA-binding protein
	SCA_RS11345	carbamate kinase
	SCA_RS11350	YfcC family protein
	SCA_RS11355	hypothetical protein
	SCA_RS11360	YfcC family protein
	SCA_RS11365	carbamate kinase
	SCA_RS11370	ornithine carbamoyltransferase
	SCA_RS11380	hypothetical protein
	SCA_RS11400	hypothetical protein
	SCA_RS11405	amidohydrolase
	SCA_RS12590	hypothetical protein
	SCA_RS11420	CPBP family intramembrane metalloprotease
	SCA_RS11430	NADPH:quinone reductase
	SCA_RS11470	MFS transporter
	SCA_RS11475	hypothetical protein
	SCA_RS11480	hypothetical protein
	SCA_RS12595	hypothetical protein
	SCA_RS11505	YSIRK signal domain/LPXTG anchor domain surface protein
	SCA_RS11510	hypothetical protein
	SCA_RS11515	hypothetical protein
	SCA_RS11525	short-chain dehydrogenase
	SCA_RS11555	hypothetical protein
	SCA_RS11560	hypothetical protein
	SCA_RS11565	hypothetical protein
	SCA_RS11580	hypothetical protein
	SCA_RS11585	hypothetical protein
	SCA_RS11600	hypothetical protein
	SCA_RS11605	hypothetical protein
	SCA_RS11610	hypothetical protein
	SCA_RS11615	Superfamily I DNA and RNA helicase
	SCA_RS11620	hypothetical protein
	SCA_RS11635	hypothetical protein
	SCA_RS11640	hypothetical protein
	SCA_RS11645	hypothetical protein
	SCA_RS11650	WYL domain-containing protein
	SCA_RS11655	DUF4870 domain-containing protein
	SCA_RS11660	hypothetical protein
	SCA_RS11665	hypothetical protein
	SCA_RS11700	LlaJI family restriction endonuclease
	SCA_RS11705	hypothetical protein
	SCA_RS11710	hypothetical protein
	SCA_RS11715	zinc ribbon domain-containing protein

Strain	Gene locus	Product
	SCA_RS11720	hypothetical protein
	SCA_RS11725	zinc ribbon domain-containing protein
	SCA_RS11730	hypothetical protein
	SCA_RS11735	transcriptional regulator
	SCA_RS11745	alpha/beta hydrolase
	SCA_RS11750	succinyl-diaminopimelate desuccinylase
	SCA_RS11755	butanediol dehydrogenase
	SCA_RS11770	CPBP family intramembrane metalloprotease domain-containing protein
	SCA_RS11780	hypothetical protein
	SCA_RS12600	TetR family transcriptional regulator
	SCA_RS11800	NAD(P)-dependent oxidoreductase
	SCA_RS11805	glycosyltransferase family 1 protein
	SCA_RS11810	hypothetical protein
	SCA_RS11815	nucleotide sugar dehydrogenase
	SCA_RS11820	NAD-dependent epimerase
	SCA_RS11825	hypothetical protein
	SCA_RS11830	hypothetical protein
	SCA_RS11835	hypothetical protein
	SCA_RS11860	hypothetical protein
	SCA_RS11870	EamA family transporter
	SCA_RS11920	2-dehydropantoate 2-reductase
	SCA_RS11925	LysR family transcriptional regulator
	SCA_RS11950	hypothetical protein
	SCA_RS11965	hypothetical protein
	SCA_RS11980	hypothetical protein
	SCA_RS11990	amino acid permease
	SCA_RS11995	gamma-glutamyl-gamma-aminobutyrate hydrolase
	SCA_RS12035	hypothetical protein
	SCA_RS12040	hypothetical protein
	SCA_RS12065	histidine phosphatase family protein
	SCA_RS12080	CDP-glycerol glycerophosphotransferase
	SCA_RS12085	CDP-glycerol glycerophosphotransferase
	SCA_RS12120	fructose-bisphosphatase class II
	SCA_RS12180	hypothetical protein
	SCA_RS12185	hypothetical protein
	SCA_RS12190	ABC transporter
	SCA_RS12195	hypothetical protein
	SCA_RS12200	phage infection protein
	SCA_RS12205	N-acetylmuramoyl-L-alanine amidase
	SCA_RS12210	hypothetical protein
	SCA_RS12240	transcriptional regulator
	SCA_RS12265	hypothetical protein
	SCA_RS12275	serine protease
	SCA_RS12280	penicillin-binding protein 4
	SCA_RS12285	hypothetical protein
	SCA_RS12295	MFS transporter

Strain	Gene locus	Product
	SCA_RS12300	MFS transporter
	SCA_RS12305	HNH endonuclease
	SCA_RS12350	YitT family protein
	SCA_RS12380	hypothetical protein
	SCA_RS12395	hypothetical protein
KS1039	SE1039_RS00085	hypothetical protein
	SE1039_RS00090	hypothetical protein
	SE1039_RS00100	hypothetical protein
	SE1039_RS00105	hypothetical protein
	SE1039_RS00150	flavodoxin family protein
	SE1039_RS00155	peroxiredoxin
	SE1039_RS00170	aldo/keto reductase
	SE1039_RS00175	MerR family transcriptional regulator
	SE1039_RS00180	alpha/beta hydrolase
	SE1039_RS00205	Mph(C) family macrolide 2-phosphotransferase
	SE1039_RS00220	GntR family transcriptional regulator
	SE1039_RS00225	tartrate dehydrogenase
	SE1039_RS00230	cation transporter
	SE1039_RS00235	glycerate kinase
	SE1039_RS00240	helix-turn-helix domain-containing protein
	SE1039_RS00245	hypothetical protein
	SE1039_RS00270	hypothetical protein
	SE1039_RS00280	hypothetical protein
	SE1039_RS00295	tripartite tricarboxylate transporter substrate binding protein
	SE1039_RS00300	tripartite tricarboxylate transporter TctB family protein
	SE1039_RS00305	transporter
	SE1039_RS00390	hypothetical protein
	SE1039_RS00475	hypothetical protein
	SE1039_RS00480	general stress protein
	SE1039_RS00495	ferrochelatase
	SE1039_RS00510	3-alpha-hydroxysteroid dehydrogenase
	SE1039_RS00515	MarR family transcriptional regulator
	SE1039_RS00520	oxidoreductase
	SE1039_RS00535	hypothetical protein
	SE1039_RS00540	hypothetical protein
	SE1039_RS00545	hypothetical protein
	SE1039_RS00550	hypothetical protein
	SE1039_RS00555	ABC transporter substrate-binding protein
	SE1039_RS00560	iron ABC transporter permease
	SE1039_RS00565	ABC transporter ATP-binding protein
	SE1039_RS00610	hypothetical protein
	SE1039_RS00615	NINE protein
	SE1039_RS00620	4-phosphopantetheinyl transferase
	SE1039_RS00625	non-ribosomal peptide synthase
	SE1039_RS00710	hypothetical protein
	SE1039_RS00720	PTS sugar transporter subunit IIB
	SE1039_RS00725	PTS system

Strain	Gene locus	Product
	SE1039_RS00730	PTS lactose/cellobiose transporter subunit IIA
	SE1039_RS00735	6-phospho-beta-glucosidase
	SE1039_RS00740	transcriptional antiterminator
	SE1039_RS00815	hypothetical protein
	SE1039_RS00820	ABC transporter ATP-binding protein
	SE1039_RS00825	ABC transporter ATP-binding protein
	SE1039_RS00860	hypothetical protein
	SE1039_RS00905	hypothetical protein
	SE1039_RS00915	hypothetical protein
	SE1039_RS00920	hypothetical protein
	SE1039_RS00925	hypothetical protein
	SE1039_RS00930	hypothetical protein
	SE1039_RS00935	ABC transporter
	SE1039_RS13530	hypothetical protein
	SE1039_RS00965	hypothetical protein
	SE1039_RS00985	hypothetical protein
	SE1039_RS01160	ISL3 family transposase
	SE1039_RS01600	hypothetical protein
	SE1039_RS01605	hypothetical protein
	SE1039_RS01670	hypothetical protein
	SE1039_RS01675	hypothetical protein
	SE1039_RS01775	hypothetical protein
	SE1039_RS01815	hypothetical protein
	SE1039_RS01830	TetR family transcriptional regulator
	SE1039_RS01835	ABC transporter ATP-binding protein
	SE1039_RS01840	hypothetical protein
	SE1039_RS01845	hypothetical protein
	SE1039_RS01850	hypothetical protein
	SE1039_RS01855	TetR/AcrR family transcriptional regulator
	SE1039_RS01860	MFS transporter
	SE1039_RS01900	hypothetical protein
	SE1039_RS01905	hypothetical protein
	SE1039_RS01910	GTP pyrophosphokinase
	SE1039_RS01915	hypothetical protein
	SE1039_RS01945	hypothetical protein
	SE1039_RS01950	hypothetical protein
	SE1039_RS01955	transposon DNA-invertase
	SE1039_RS02045	sodium:alanine symporter family protein
	SE1039_RS02225	MerR family transcriptional regulator
	SE1039_RS03105	N-acetyltransferase
	SE1039_RS03115	N-acetyltransferase
	SE1039_RS03125	N-acetyltransferase
	SE1039_RS03130	cytidine deaminase
	SE1039_RS03140	hypothetical protein
	SE1039_RS03150	CPBP family intramembrane metalloprotease
	SE1039_RS03165	hypothetical protein
	SE1039_RS03175	hypothetical protein

Strain	Gene locus	Product
	SE1039_RS03190	hypothetical protein
	SE1039_RS03200	cold-shock protein
	SE1039_RS03225	tryptophan-rich sensory protein
	SE1039_RS03460	hypothetical protein
	SE1039_RS03605	MFS transporter
	SE1039_RS03610	RimJ/RimL family protein N-acetyltransferase
	SE1039_RS03950	hypothetical protein
	SE1039_RS03955	permease
	SE1039_RS03960	ArsR family transcriptional regulator
	SE1039_RS03965	arsenical resistance operon transcriptional repressor ArsD
	SE1039_RS03970	arsenical pump-driving ATPase
	SE1039_RS03975	dehydrogenase
	SE1039_RS04115	hypothetical protein
	SE1039_RS04530	hypothetical protein
	SE1039_RS04540	hypothetical protein
	SE1039_RS04545	DUF4352 domain-containing protein
	SE1039_RS13565	putative holin-like toxin
	SE1039_RS04550	hypothetical protein
	SE1039_RS04555	hypothetical protein
	SE1039_RS04780	hypothetical protein
	SE1039_RS04785	hypothetical protein
	SE1039_RS04790	XRE family transcriptional regulator
	SE1039_RS04795	helix-turn-helix domain-containing protein
	SE1039_RS04800	transcriptional regulator
	SE1039_RS04805	hypothetical protein
	SE1039_RS04810	hypothetical protein
	SE1039_RS04835	hypothetical protein
	SE1039_RS04840	chromosomal replication initiator DnaA
	SE1039_RS04845	XRE family transcriptional regulator
	SE1039_RS04850	hypothetical protein
	SE1039_RS04885	hypothetical protein
	SE1039_RS04895	hypothetical protein
	SE1039_RS04910	hypothetical protein
	SE1039_RS04915	hypothetical protein
	SE1039_RS04920	hypothetical protein
	SE1039_RS04930	hypothetical protein
	SE1039_RS04935	transcriptional regulator
	SE1039_RS04940	hypothetical protein
	SE1039_RS04945	terminase small subunit
	SE1039_RS04950	terminase
	SE1039_RS04975	hypothetical protein
	SE1039_RS05025	hypothetical protein
	SE1039_RS05060	hypothetical protein
	SE1039_RS05065	hypothetical protein
	SE1039_RS05070	ParA family protein
	SE1039_RS05075	hypothetical protein
	SE1039_RS05080	hypothetical protein

Strain	Gene locus	Product
	SE1039_RS05095	hypothetical protein
	SE1039_RS05140	hypothetical protein
	SE1039_RS05535	NAD(P)/FAD-dependent oxidoreductase
	SE1039_RS05560	AraC family transcriptional regulator
	SE1039_RS05565	hypothetical protein
	SE1039_RS05570	lipase
	SE1039_RS05575	glycosyl hydrolase family 3
	SE1039_RS05625	anion permease
	SE1039_RS05670	hypothetical protein
	SE1039_RS05675	VOC family protein
	SE1039_RS05805	hypothetical protein
	SE1039_RS05820	hypothetical protein
	SE1039_RS05915	hypothetical protein
	SE1039_RS13575	hypothetical protein
	SE1039_RS06005	hypothetical protein
	SE1039_RS06275	hypothetical protein
	SE1039_RS07235	hypothetical protein
	SE1039_RS07370	aldo/keto reductase
	SE1039_RS07405	hypothetical protein
	SE1039_RS08030	glycosyl transferase family A
	SE1039_RS08035	hypothetical protein
	SE1039_RS08180	hypothetical protein
	SE1039_RS09075	transglycosylase
	SE1039_RS09375	hypothetical protein
	SE1039_RS10840	hypothetical protein
	SE1039_RS11600	membrane protein
	SE1039_RS11675	tripartite tricarboxylate transporter TctB family protein
	SE1039_RS11680	tripartite tricarboxylate transporter substrate binding protein
	SE1039_RS11690	3-hydroxybenzoate 6-hydroxylase
	SE1039_RS11695	cupin
	SE1039_RS11700	DinB family protein
	SE1039_RS11705	2-hydroxyhepta-2
	SE1039_RS11710	IclR family transcriptional regulator
	SE1039_RS11715	MFS transporter
	SE1039_RS11765	ROK family protein
	SE1039_RS11830	5
	SE1039_RS11835	acetate--CoA ligase
	SE1039_RS11860	idonate transporter
	SE1039_RS11900	hypothetical protein
	SE1039_RS11920	N-acetyltransferase
	SE1039_RS11925	QacE family quaternary ammonium compound efflux SMR transporter
	SE1039_RS11950	ABC transporter substrate-binding protein
	SE1039_RS12120	NADH-dependent flavin oxidoreductase
	SE1039_RS13615	hypothetical protein
	SE1039_RS13620	hypothetical protein
	SE1039_RS12395	3-hydroxybutyryl-CoA dehydrogenase

Strain	Gene locus	Product
	SE1039_RS12400	citrate transporter
	SE1039_RS12405	histidinol dehydrogenase
	SE1039_RS12410	LacI family transcriptional regulator
	SE1039_RS12480	LacI family transcriptional regulator
	SE1039_RS12515	haloacid dehalogenase
	SE1039_RS12520	hypothetical protein
	SE1039_RS12530	RNA pseudouridine synthase
	SE1039_RS12550	hypothetical protein
	SE1039_RS12625	hypothetical protein
	SE1039_RS12655	siderophore biosynthesis protein SbnI
	SE1039_RS12660	diaminopimelate decarboxylase
	SE1039_RS12665	siderophore biosynthesis protein SbnG
	SE1039_RS12670	IucA/IucC family siderophore biosynthesis protein
	SE1039_RS12675	siderophore biosynthesis protein SbnE
	SE1039_RS12680	MFS transporter
	SE1039_RS12685	siderophore biosynthesis protein SbnC
	SE1039_RS12690	2,3-diaminopropionate biosynthesis protein SbnB
	SE1039_RS12695	2,3-diaminopropionate biosynthesis protein SbnA
	SE1039_RS12700	iron ABC transporter substrate-binding protein
	SE1039_RS12705	iron ABC transporter permease
	SE1039_RS12710	iron ABC transporter permease
	SE1039_RS13630	preprotein translocase subunit YajC
	SE1039_RS12715	hypothetical protein
	SE1039_RS12755	hypothetical protein
	SE1039_RS12765	hypothetical protein
	SE1039_RS12795	accessory Sec system glycosyltransferase GtfA
	SE1039_RS12800	hypothetical protein
	SE1039_RS12805	hypothetical protein
	SE1039_RS12810	peptide ABC transporter ATP-binding protein
	SE1039_RS12815	peptide ABC transporter permease
	SE1039_RS12825	sodium:pantothenate symporter
	SE1039_RS12835	hypothetical protein
	SE1039_RS12850	NADP-dependent oxidoreductase
	SE1039_RS12855	sodium:proline symporter
	SE1039_RS12875	hypothetical protein
	SE1039_RS12880	luciferase family oxidoreductase
	SE1039_RS12965	tRNA-specific adenosine deaminase
	SE1039_RS12970	hypothetical protein
	SE1039_RS12975	hypothetical protein
	SE1039_RS12985	DNA polymerase
	SE1039_RS12990	hypothetical protein
	SE1039_RS12995	hypothetical protein
	SE1039_RS13055	hypothetical protein
	SE1039_RS13060	NADP-dependent oxidoreductase
	SE1039_RS13065	oxidoreductase
	SE1039_RS13075	MerR family transcriptional regulator
	SE1039_RS13080	hypothetical protein

Strain	Gene locus	Product
	SE1039_RS13085	5-methylcytosine-specific restriction system specificity protein McrC
	SE1039_RS13090	GTPase
	SE1039_RS13095	hypothetical protein
	SE1039_RS13100	hypothetical protein
	SE1039_RS13105	hypothetical protein
	SE1039_RS13110	CRISPR-associated endoribonuclease Cas6
	SE1039_RS13115	type III-A CRISPR-associated protein Csm6
	SE1039_RS13120	type III-A CRISPR-associated RAMP protein Csm5
	SE1039_RS13125	type III-A CRISPR-associated RAMP protein Csm4
	SE1039_RS13130	type III-A CRISPR-associated RAMP protein Csm3
	SE1039_RS13135	type III-A CRISPR-associated protein Csm2
	SE1039_RS13145	CRISPR-associated endonuclease Cas2
	SE1039_RS13150	type II CRISPR-associated endonuclease Cas1
	SE1039_RS13160	hypothetical protein
	SE1039_RS13180	type I restriction endonuclease subunit S
	SE1039_RS13200	hypothetical protein
	SE1039_RS13225	hypothetical protein
	SE1039_RS13230	hypothetical protein
	SE1039_RS13235	hypothetical protein
	SE1039_RS13255	phosphoglucomutase
	SE1039_RS13265	CDP-glycerol glycerophosphotransferase
	SE1039_RS13280	hypothetical protein
	SE1039_RS13300	hypothetical protein
	SE1039_RS13305	hypothetical protein
	SE1039_RS13340	hypothetical protein
	SE1039_RS13350	hypothetical protein
	SE1039_RS13645	hypothetical protein
	SE1039_RS13360	hypothetical protein
	SE1039_RS13365	hypothetical protein
14BME20	BK815_RS00705	hypothetical protein
	BK815_RS01235	CDP-glycerol:glycerophosphate glycerophosphotransferase
	BK815_RS01255	proline dehydrogenase
	BK815_RS01260	hypothetical protein
	BK815_RS01405	hypothetical protein
	BK815_RS01410	hypothetical protein
	BK815_RS01415	VOC family virulence protein
	BK815_RS02040	MFS transporter
	BK815_RS02070	hypothetical protein
	BK815_RS02100	N-acetyltransferase
	BK815_RS02105	N-acetyltransferase
	BK815_RS02110	hypothetical protein
	BK815_RS02615	transcriptional regulator
	BK815_RS02620	N-acyl homoserine lactonase family protein
	BK815_RS02670	XRE family transcriptional regulator
	BK815_RS02675	XRE family transcriptional regulator
	BK815_RS02680	Rha family transcriptional regulator

Strain	Gene locus	Product
	BK815_RS02690	hypothetical protein
	BK815_RS02705	hypothetical protein
	BK815_RS02725	replication protein
	BK815_RS02745	hypothetical protein
	BK815_RS02750	hypothetical protein
	BK815_RS02770	hypothetical protein
	BK815_RS02775	hypothetical protein
	BK815_RS02790	hypothetical protein
	BK815_RS02795	hypothetical protein
	BK815_RS02800	hypothetical protein
	BK815_RS02810	terminase
	BK815_RS02835	hypothetical protein
	BK815_RS02885	hypothetical protein
	BK815_RS02890	hypothetical protein
	BK815_RS02920	hypothetical protein
	BK815_RS02925	hypothetical protein
	BK815_RS02930	hypothetical protein
	BK815_RS02935	hypothetical protein
	BK815_RS03440	hypothetical protein
	BK815_RS03790	phosphoglucomutase
	BK815_RS03800	hypothetical protein
	BK815_RS03805	hypothetical protein
	BK815_RS03815	hypothetical protein
	BK815_RS03820	hypothetical protein
	BK815_RS03825	hypothetical protein
	BK815_RS03830	hypothetical protein
	BK815_RS03845	hypothetical protein
	BK815_RS03855	MFS transporter
	BK815_RS03865	LysE family translocator
	BK815_RS03870	YqcI/YcgG family protein
	BK815_RS03875	hypothetical protein
	BK815_RS03890	hypothetical protein
	BK815_RS03895	hypothetical protein
	BK815_RS03905	hypothetical protein
	BK815_RS03910	hypothetical protein
	BK815_RS03930	hypothetical protein
	BK815_RS03935	hypothetical protein
	BK815_RS03940	hypothetical protein
	BK815_RS03950	MFS transporter
	BK815_RS03955	alpha-N-arabinofuranosidase
	BK815_RS04220	hypothetical protein
	BK815_RS04780	NINE protein
	BK815_RS04785	esterase
	BK815_RS04790	hypothetical protein
	BK815_RS05020	hypothetical protein
	BK815_RS05035	hypothetical protein
	BK815_RS05040	XRE family transcriptional regulator

Strain	Gene locus	Product
	BK815_RS05045	hypothetical protein
	BK815_RS05050	hypothetical protein
	BK815_RS06150	hypothetical protein
	BK815_RS06155	N-acetyltransferase
	BK815_RS06300	DoxX family protein
	BK815_RS06315	PLP-dependent aminotransferase family protein
	BK815_RS06320	EamA family transporter
	BK815_RS06335	hypothetical protein
	BK815_RS06345	hypothetical protein
	BK815_RS06360	hypothetical protein
	BK815_RS06475	SAM-dependent methyltransferase
	BK815_RS07275	hypothetical protein
	BK815_RS07280	glyoxalase
	BK815_RS07625	hypothetical protein
	BK815_RS08645	MFS transporter
	BK815_RS08650	LysR family transcriptional regulator
	BK815_RS08660	glyoxalase/bleomycin resistance/dioxygenase family protein
	BK815_RS08675	8-amino-7-oxononanoate synthase
	BK815_RS08680	6-carboxyhexanoate--CoA ligase
	BK815_RS08690	choline transporter
	BK815_RS08855	hypothetical protein
	BK815_RS08860	hypothetical protein
	BK815_RS08885	MarR family transcriptional regulator
	BK815_RS08920	hypothetical protein
	BK815_RS08925	gfo/Idh/MocA family oxidoreductase
	BK815_RS08935	myo-inosose-2 dehydratase
	BK815_RS08940	5-dehydro-2-deoxygluconokinase
	BK815_RS08945	5-deoxy-glucuronate isomerase
	BK815_RS08950	methylmalonate-semialdehyde dehydrogenase (CoA acylating)
	BK815_RS08955	DeoR/GlpR transcriptional regulator
	BK815_RS08960	isomerase
	BK815_RS08965	protein iolH
	BK815_RS08970	3D-(3,5/4)-trihydroxycyclohexane-1,2-dione acylhydrolase (decyclizing)
	BK815_RS08975	sugar porter family MFS transporter
	BK815_RS09020	ATP-grasp domain-containing protein
	BK815_RS09055	hypothetical protein
	BK815_RS09090	MFS transporter
	BK815_RS09095	sucrose-6-phosphate hydrolase
	BK815_RS09100	LacI family transcriptional regulator
	BK815_RS09105	MFS transporter
	BK815_RS09115	hypothetical protein
	BK815_RS09120	agmatinase
	BK815_RS09125	sodium:solute symporter
	BK815_RS09130	N-acetyl-L,L-diaminopimelate aminotransferase
	BK815_RS09135	hypothetical protein
	BK815_RS09145	hypothetical protein

Strain	Gene locus	Product
	BK815_RS09180	hypothetical protein
	BK815_RS09185	hypothetical protein
	BK815_RS09200	nitrilotriacetate monooxygenase
	BK815_RS09205	transcriptional regulator
	BK815_RS09210	alpha/beta hydrolase
	BK815_RS09220	hypothetical protein
	BK815_RS09225	amino acid permease
	BK815_RS09240	MarR family transcriptional regulator
	BK815_RS09245	hypothetical protein
	BK815_RS09250	LysR family transcriptional regulator
	BK815_RS09255	glycerol dehydrogenase
	BK815_RS09265	hypothetical protein
	BK815_RS09270	hypothetical protein
	BK815_RS09275	short-chain dehydrogenase
	BK815_RS09280	TetR/AcrR family transcriptional regulator
	BK815_RS09295	ATP-grasp domain-containing protein
	BK815_RS09300	hypothetical protein
	BK815_RS09305	hypothetical protein
	BK815_RS09310	beta-ketoacyl-[acyl-carrier-protein] synthase II
	BK815_RS09325	hypothetical protein
	BK815_RS09355	cold-shock protein
	BK815_RS09360	aldo/keto reductase
	BK815_RS09390	SDR family oxidoreductase
	BK815_RS09395	Rrf2 family transcriptional regulator
	BK815_RS09405	alpha/beta hydrolase
	BK815_RS09415	alpha/beta hydrolase
	BK815_RS09420	Lrp/AsnC family transcriptional regulator
	BK815_RS09430	hypothetical protein
	BK815_RS09445	6-phospho-3-hexuloisomerase
	BK815_RS09450	3-hexulose-6-phosphate synthase
	BK815_RS09460	MFS transporter
	BK815_RS09475	hypothetical protein
	BK815_RS09485	aldo/keto reductase
	BK815_RS09675	restriction endonuclease
	BK815_RS09680	hypothetical protein
	BK815_RS09730	hypothetical protein
	BK815_RS09740	ATP-binding protein
	BK815_RS09745	abortive phage resistance protein
	BK815_RS09750	hypothetical protein
	BK815_RS09765	hypothetical protein
	BK815_RS09785	hypothetical protein
	BK815_RS09825	type 1 glutamine amidotransferase
	BK815_RS09830	transcriptional regulator
	BK815_RS09835	transcriptional regulator
	BK815_RS09840	aldehyde dehydrogenase
	BK815_RS09870	non-ribosomal peptide synthetase
	BK815_RS09920	TetR/AcrR family transcriptional regulator

Strain	Gene locus	Product
	BK815_RS09935	hypothetical protein
	BK815_RS09945	hypothetical protein
	BK815_RS09975	gfo/Idh/MocA family oxidoreductase
	BK815_RS09980	hypothetical protein
	BK815_RS09985	MFS transporter
	BK815_RS10005	alcohol dehydrogenase
	BK815_RS10075	transglycosylase
	BK815_RS10090	MerR family transcriptional regulator
	BK815_RS10100	quercetin 2,3-dioxygenase
	BK815_RS10270	nitrilotriacetate monooxygenase
	BK815_RS10280	AP endonuclease
	BK815_RS10285	hypothetical protein
	BK815_RS10290	ribokinase
	BK815_RS10300	PadR family transcriptional regulator
	BK815_RS10310	DUF3237 domain-containing protein
	BK815_RS10335	ABC transporter ATP-binding protein
	BK815_RS10340	hypothetical protein
	BK815_RS10345	ABC transporter permease
	BK815_RS10350	peptide ABC transporter substrate-binding protein
	BK815_RS10355	gamma-glutamyltransferase
	BK815_RS10360	hypothetical protein
	BK815_RS10365	hypothetical protein
	BK815_RS10440	hypothetical protein
	BK815_RS10450	hypothetical protein
	BK815_RS10500	hypothetical protein
	BK815_RS10615	gamma-aminobutyrate permease
	BK815_RS10835	endonuclease
	BK815_RS10855	type I pantothenate kinase
	BK815_RS11055	HXXEE domain-containing protein
	BK815_RS11070	transcriptional regulator
	BK815_RS11425	phosphoesterase
	BK815_RS12160	hypothetical protein
	BK815_RS13000	carbon-nitrogen hydrolase family protein
	BK815_RS13005	amino acid permease
C2a	SXYL_RS00145	hypothetical protein
	SXYL_RS00155	restriction endonuclease subunit R
	SXYL_RS00160	transporter
	SXYL_RS00175	hypothetical protein
	SXYL_RS00180	inositol 2-dehydrogenase
	SXYL_RS00205	hypothetical protein
	SXYL_RS00220	flavin reductase
	SXYL_RS00225	hypothetical protein
	SXYL_RS00330	diaminopimelate epimerase
	SXYL_RS00335	ribosomal RNA methyltransferase FmrO domain protein
	SXYL_RS00340	hypothetical protein
	SXYL_RS00345	nickel ABC transporter
	SXYL_RS00350	ABC transporter permease

Strain	Gene locus	Product
	SXYL_RS00355	ABC transporter permease
	SXYL_RS00360	ABC transporter ATP-binding protein
	SXYL_RS00365	ABC transporter ATP-binding protein
	SXYL_RS00370	MFS transporter
	SXYL_RS00380	TM2 domain-containing protein
	SXYL_RS13240	hypothetical protein
	SXYL_RS00535	glyoxalase
	SXYL_RS00555	DNA-binding response regulator
	SXYL_RS00680	AraC family transcriptional regulator
	SXYL_RS00745	hypothetical protein
	SXYL_RS00850	hypothetical protein
	SXYL_RS01195	hypothetical protein
	SXYL_RS01205	IclR family transcriptional regulator
	SXYL_RS01210	gluconate permease
	SXYL_RS01215	alcohol dehydrogenase
	SXYL_RS01325	hypothetical protein
	SXYL_RS01400	MFS transporter
	SXYL_RS02195	2-dehydro-3-deoxyphosphogluconate aldolase
	SXYL_RS02200	MFS transporter
	SXYL_RS02205	uronate isomerase
	SXYL_RS13190	hypothetical protein
	SXYL_RS04720	hypothetical protein
	SXYL_RS05025	anion permease
	SXYL_RS05185	MazF/PemK family toxin
	SXYL_RS05195	hypothetical protein
	SXYL_RS05200	phage tail family protein
	SXYL_RS05210	hypothetical protein
	SXYL_RS05215	hypothetical protein
	SXYL_RS05220	hypothetical protein
	SXYL_RS13200	hypothetical protein
	SXYL_RS05230	hypothetical protein
	SXYL_RS05235	hypothetical protein
	SXYL_RS05250	CHAP domain-containing protein
	SXYL_RS05260	hypothetical protein
	SXYL_RS05265	hypothetical protein
	SXYL_RS05270	hypothetical protein
	SXYL_RS05275	hypothetical protein
	SXYL_RS06200	anion permease
	SXYL_RS07065	site-specific integrase
	SXYL_RS07070	hypothetical protein
	SXYL_RS07075	hypothetical protein
	SXYL_RS07080	hypothetical protein
	SXYL_RS07090	hypothetical protein
	SXYL_RS07095	hypothetical protein
	SXYL_RS07100	hypothetical protein
	SXYL_RS07105	hypothetical protein
	SXYL_RS07110	hypothetical protein

Strain	Gene locus	Product
	SXYL_RS07120	hypothetical protein
	SXYL_RS07125	hypothetical protein
	SXYL_RS07130	hypothetical protein
	SXYL_RS07135	hypothetical protein
	SXYL_RS07140	hypothetical protein
	SXYL_RS13285	arsenic transporter
	SXYL_RS07435	hypothetical protein
	SXYL_RS07500	hypothetical protein
	SXYL_RS07715	hypothetical protein
	SXYL_RS07815	hypothetical protein
	SXYL_RS07825	hypothetical protein
	SXYL_RS08465	GlsB/YeaQ/YmgE family stress response membrane protein
	SXYL_RS08475	hypothetical protein
	SXYL_RS08485	hypothetical protein
	SXYL_RS08490	hypothetical protein
	SXYL_RS08495	hypothetical protein
	SXYL_RS08505	hypothetical protein
	SXYL_RS08510	phage holin
	SXYL_RS13205	hypothetical protein
	SXYL_RS08535	peptidase G2
	SXYL_RS08540	hypothetical protein
	SXYL_RS08545	peptidase
	SXYL_RS13305	phage tail tape measure protein
	SXYL_RS08570	hypothetical protein
	SXYL_RS08575	hypothetical protein
	SXYL_RS08580	phage tail protein
	SXYL_RS08585	hypothetical protein
	SXYL_RS08595	hypothetical protein
	SXYL_RS08600	DNA-packaging protein
	SXYL_RS08605	phage major capsid protein
	SXYL_RS08610	peptidase U35
	SXYL_RS08615	phage portal protein
	SXYL_RS08620	hypothetical protein
	SXYL_RS08625	terminase
	SXYL_RS08630	terminase
	SXYL_RS08635	HNH endonuclease
	SXYL_RS08640	transcriptional regulator
	SXYL_RS08645	hypothetical protein
	SXYL_RS08650	hypothetical protein
	SXYL_RS08655	hypothetical protein
	SXYL_RS08665	hypothetical protein
	SXYL_RS08670	hypothetical protein
	SXYL_RS08680	HNH endonuclease
	SXYL_RS08685	hypothetical protein
	SXYL_RS08700	hypothetical protein
	SXYL_RS08750	hypothetical protein
	SXYL_RS08760	hypothetical protein

Strain	Gene locus	Product
	SXYL_RS08765	oxidoreductase
	SXYL_RS08770	helix-turn-helix domain-containing protein
	SXYL_RS08775	transcriptional regulator
	SXYL_RS13210	hypothetical protein
	SXYL_RS08790	integrase
	SXYL_RS09835	hypothetical protein
	SXYL_RS09840	N-acetyltransferase
	SXYL_RS10065	hypothetical protein
	SXYL_RS10080	hypothetical protein
	SXYL_RS13215	hypothetical protein
	SXYL_RS13330	hypothetical protein
	SXYL_RS11165	hypothetical protein
	SXYL_RS11175	hypothetical protein
	SXYL_RS11315	hypothetical protein
	SXYL_RS11320	C4-dicarboxylate ABC transporter permease
	SXYL_RS11325	hypothetical protein
	SXYL_RS11330	S-adenosylmethionine--2-demethylmenaquinone methyltransferase
	SXYL_RS11335	MurR/RpiR family transcriptional regulator
	SXYL_RS11360	YSIRK signal domain/LPXTG anchor domain surface protein
	SXYL_RS11390	hypothetical protein
	SXYL_RS11510	hypothetical protein
	SXYL_RS12335	hypothetical protein
	SXYL_RS12385	hypothetical protein
	SXYL_RS12400	transcriptional regulator
	SXYL_RS12520	hypothetical protein
	SXYL_RS12525	tripartite tricarboxylate transporter substrate binding protein
	SXYL_RS12530	hypothetical protein
	SXYL_RS13220	hypothetical protein
	SXYL_RS13230	hypothetical protein
	SXYL_RS12640	gluconate permease
	SXYL_RS12645	hypothetical protein
	SXYL_RS12650	2-hydroxy-3-oxopropionate reductase
	SXYL_RS12665	membrane protein
	SXYL_RS12680	iron-containing alcohol dehydrogenase
	SXYL_RS12685	hypothetical protein
	SXYL_RS12695	hypothetical protein
	SXYL_RS12715	zinc ribbon domain-containing protein
	SXYL_RS12720	zinc ribbon domain-containing protein
	SXYL_RS12995	magnesium transporter
	SXYL_RS13000	zinc ABC transporter substrate-binding protein
	SXYL_RS13005	metal ABC transporter permease
	SXYL_RS13010	manganese ABC transporter ATP-binding protein
	SXYL_RS13020	hypothetical protein
	SXYL_RS13025	LacI family transcriptional regulator
	SXYL_RS13030	metal ABC transporter
	SXYL_RS13035	dihydrodipicolinate synthase family protein

Strain	Gene locus	Product
	SXYL_RS13040	acyltransferase
	SXYL_RS13075	acyltransferase
	SXYL_RS13080	hypothetical protein
	SXYL_RS13085	CDP-glycerol--glycerophosphate glycerophosphotransferase
	SXYL_RS13355	CDP-glycerol:glycerophosphate glycerophosphotransferase
ATCC 15305	SSP_RS00145	hypothetical protein
	SSP_RS00150	site-specific DNA-methyltransferase
	SSP_RS00155	modification methylase
	SSP_RS00165	hypothetical protein
	SSP_RS00210	hypothetical protein
	SSP_RS00215	hypothetical protein
	SSP_RS00220	hypothetical protein
	SSP_RS00245	hypothetical protein
	SSP_RS12635	restriction endonuclease S subunit
	SSP_RS00325	glycosyl transferase
	SSP_RS00330	glycosyl transferase
	SSP_RS00335	capsular polysaccharide synthesis protein
	SSP_RS00340	glycosyl transferase
	SSP_RS00350	polysaccharide polymerase
	SSP_RS12690	hypothetical protein
	SSP_RS12695	hypothetical protein
	SSP_RS00370	HNH endonuclease
	SSP_RS00375	ATPase
	SSP_RS12700	hypothetical protein
	SSP_RS00390	glycosyl transferase
	SSP_RS12705	DUF2648 domain-containing protein
	SSP_RS00415	malate:quinone oxidoreductase
	SSP_RS00420	acetolactate synthase AlsS
	SSP_RS00430	L-lactate permease
	SSP_RS00435	hypothetical protein
	SSP_RS00480	acetyltransferase
	SSP_RS00490	hypothetical protein
	SSP_RS00495	hypothetical protein
	SSP_RS00505	MBL fold metallo-hydrolase
	SSP_RS00510	transcriptional regulator
	SSP_RS00560	transcriptional regulator
	SSP_RS00565	hypothetical protein
	SSP_RS00580	transglycosylase SceD 1
	SSP_RS12720	hypothetical protein
	SSP_RS12725	SIS domain-containing protein
	SSP_RS00620	arginase
	SSP_RS00650	TetR family transcriptional regulator
	SSP_RS12730	cell wall-anchored protein
	SSP_RS00730	hypothetical protein
	SSP_RS00770	hypothetical protein
	SSP_RS00775	hypothetical protein
	SSP_RS00780	hypothetical protein

Strain	Gene locus	Product
	SSP_RS00785	hypothetical protein
	SSP_RS00800	DUF3100 domain-containing protein
	SSP_RS00850	hypothetical protein
	SSP_RS00885	hypothetical protein
	SSP_RS00980	4-carboxymuconolactone decarboxylase
	SSP_RS00985	LytTR family transcriptional regulator
	SSP_RS00990	NAD(P)-dependent oxidoreductase
	SSP_RS00995	hypothetical protein
	SSP_RS01000	MFS transporter
	SSP_RS01095	hypothetical protein
	SSP_RS01195	transcriptional antiterminator
	SSP_RS12740	MFS transporter
	SSP_RS12745	MFS transporter
	SSP_RS01630	IS30 family transposase
	SSP_RS01675	AraC family transcriptional regulator
	SSP_RS01855	6-phospho-beta-glucosidase
	SSP_RS01860	transcription antiterminator
	SSP_RS01865	beta-1,4-N-acetylgalactosaminyltransferase
	SSP_RS02425	hypothetical protein
	SSP_RS12755	lipase precursor
	SSP_RS02565	ferrichrome ABC transporter substrate-binding protein
	SSP_RS02825	transposase
	SSP_RS03090	metal-dependent hydrolase
	SSP_RS04060	hypothetical protein
	SSP_RS04205	hypothetical protein
	SSP_RS04230	MarR family transcriptional regulator
	SSP_RS12765	type I addiction module toxin
	SSP_RS04480	hypothetical protein
	SSP_RS05625	integrase
	SSP_RS06625	autolysin
	SSP_RS06750	hypothetical protein
	SSP_RS07030	hypothetical protein
	SSP_RS07105	hypothetical protein
	SSP_RS07230	phosphonate ABC transporter
	SSP_RS07235	phosphonate ABC transporter
	SSP_RS07240	phosphonates import ATP-binding protein PhnC
	SSP_RS07245	phosphate/phosphite/phosphonate ABC transporter substrate-binding protein
	SSP_RS07250	hypothetical protein
	SSP_RS07255	bifunctional metallophosphatase/5-nucleotidase
	SSP_RS07260	hypothetical protein
	SSP_RS07265	hypothetical protein
	SSP_RS07335	DNA-binding response regulator
	SSP_RS07340	two-component sensor histidine kinase
	SSP_RS07345	ABC transporter permease
	SSP_RS07350	ABC transporter ATP-binding protein
	SSP_RS08105	LysE family translocator

Strain	Gene locus	Product
	SSP_RS08120	transposase
	SSP_RS08125	transposase
	SSP_RS08130	hypothetical protein
	SSP_RS08145	IS6 family transposase
	SSP_RS08165	3-hexulose-6-phosphate synthase 1
	SSP_RS08175	PLP-dependent aminotransferase family protein
	SSP_RS08180	EamA family transporter
	SSP_RS08200	glutamine amidotransferase
	SSP_RS08205	3-hexulose-6-phosphate synthase 2
	SSP_RS08215	transcriptional regulator
	SSP_RS08220	aldehyde dehydrogenase
	SSP_RS12810	sodium:proton antiporter
	SSP_RS08255	lipase
	SSP_RS08260	replication initiator protein A
	SSP_RS08265	IS6 family transposase
	SSP_RS09515	alpha/beta hydrolase
	SSP_RS09520	hypothetical protein
	SSP_RS09570	hypothetical protein
	SSP_RS09640	aminoglycoside 6-adenylyltransferase
	SSP_RS09665	YolD-like family protein
	SSP_RS09685	hypothetical protein
	SSP_RS09695	pathogenicity island protein
	SSP_RS09700	hypothetical protein
	SSP_RS09730	hypothetical protein
	SSP_RS09735	hypothetical protein
	SSP_RS09745	transcriptional regulator
	SSP_RS09750	transcriptional regulator
	SSP_RS10810	hypothetical protein
	SSP_RS10905	hypothetical protein
	SSP_RS10930	hypothetical protein
	SSP_RS11565	N-acetyltransferase
	SSP_RS11595	hypothetical protein
	SSP_RS11665	hypothetical protein
	SSP_RS11675	hypothetical protein
	SSP_RS11685	hypothetical protein
	SSP_RS11880	membrane protein
	SSP_RS12850	hypothetical protein
	SSP_RS12630	hypothetical protein
	SSP_RS12160	hypothetical protein
	SSP_RS12190	glycine/betaine ABC transporter
	SSP_RS12225	hypothetical protein

Table S3. Putative transposase and relaxasome identified in the 10 CNS genomes

**Product**	***S. carnosus***		***S. equorum***		***S. succinus***			***S. xylosus***		***S. saprophyticus***
	**JCM 6069**	**TM300**	**KS1039**	**Mu2**	**14BME20**	**CSM 77**	**DSM 14617**	**C2a**	**HKUOPL8**	**ATCC 15305**
Transposase gene										
DDE transposase							AA913_RS13255			
Transposase InsI for insertion sequenc e element IS30A				SEQMU2_RS12835						SSP_RS04480
IS3 family transposase							AA913_RS14195			
IS30 family transposase										SSP_RS07225
IS30 family transposase										SSP_RS01630
IS6 family transposase; IS257 Tn4003; IS431mec	BEK99_RS11640			SEQMU2_RS13865		A6V26_RS13340	AA913_RS12905			SSP_RS08265
IS6 family transposase; mobilome										SSP_RS08145
ISL3 family transposase			SE1039_RS01160							
transposase; HTH						A6V26_RS13335	AA913_RS12900			
Transposase										SSP_RS02825
transposase; HTH										SSP_RS08120
Transposase										SSP_RS08125
Transposase									BE24_RS09275	
transposase IDENTICAL PARALOGS:				SEQMU2_RS13915					BE24_RS03755	
transposase IDENTICAL PARALOGS:									BE24_RS06535	
Mobile-related gene										
mobilization protein				SEQMU2_RS14230		A6V26_RS13275	AA913_RS12950			
conjugal transfer protein							AA913_RS13620			
conjugal transfer protein							AA913_RS13455			

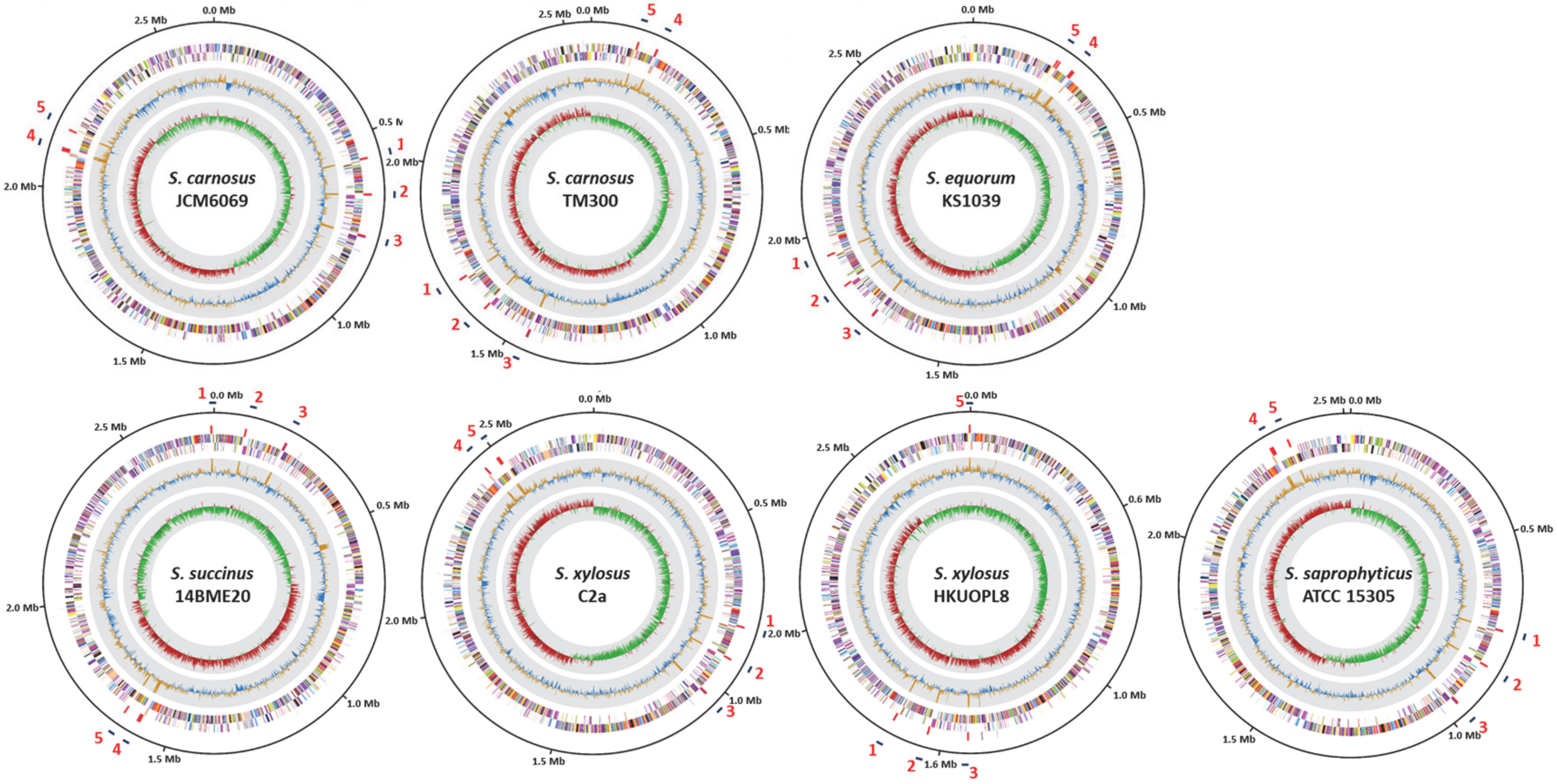

Fig. S1. Circular genome map of five coagulase-negative *Staphylococcus* complete genomes

The scales indicated the location in Mbp, starting with the initial coding region. From the outermost edge of the ring, the first circle denotes tRNAs (red) and rRNAs (blue). The second and third circles illustrate the CDSs, coloured according to COG functional categories, on the positive and complementary strand, respectively. The forth circle shows the GC contents, and the fifth circle shows the GC skew. The values were plotted as the deviation from the average GC skew of the entire sequence. Specific regions are marked by red partial fragments.

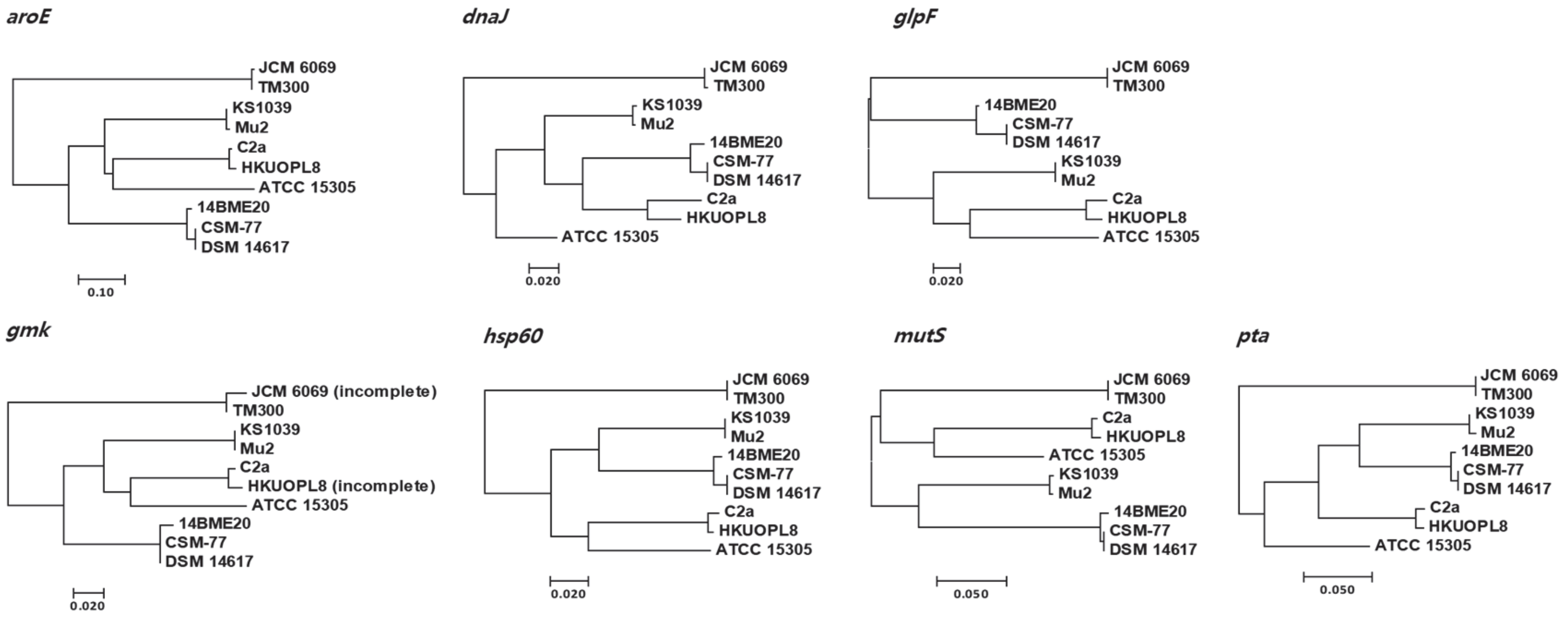

Fig. S2. Phylogenetic trees based on the partial gene sequences of *aroE, dnaJ, glpF, gmk, hsp60, mutS,* and *pta* showing the phylogenetic relationships between the 10 CNS

The phylogenetic tree was constructed using the maximum likelihood method. Branches with bootstrap values <50% were collapsed. The distance scales are shown under the trees.
